# Proteomics of Secretory and Endocytic Organelles in *Giardia lamblia*

**DOI:** 10.1371/journal.pone.0094089

**Published:** 2014-04-14

**Authors:** Petra B. Wampfler, Vinko Tosevski, Paolo Nanni, Cornelia Spycher, Adrian B. Hehl

**Affiliations:** 1 Institute of Parasitology, University of Zurich, Zurich, Switzerland; 2 Institute of Experimental Immunology, University of Zurich, Zurich, Switzerland; 3 Functional Genomics Center Zurich, Zurich, Switzerland; 4 Institute of Parasitology, University of Bern, Bern, Switzerland; Cornell University, United States of America

## Abstract

*Giardia lamblia* is a flagellated protozoan enteroparasite transmitted as an environmentally resistant cyst. Trophozoites attach to the small intestine of vertebrate hosts and proliferate by binary fission. They access nutrients directly via uptake of bulk fluid phase material into specialized endocytic organelles termed peripheral vesicles (PVs), mainly on the exposed dorsal side. When trophozoites reach the G2/M restriction point in the cell cycle they can begin another round of cell division or encyst if they encounter specific environmental cues. They induce neogenesis of Golgi-like organelles, encystation-specific vesicles (ESVs), for regulated secretion of cyst wall material. PVs and ESVs are highly simplified and thus evolutionary diverged endocytic and exocytic organelle systems with key roles in proliferation and transmission to a new host, respectively. Both organelle systems physically and functionally intersect at the endoplasmic reticulum (ER) which has catabolic as well as anabolic functions. However, the unusually high degree of sequence divergence in Giardia rapidly exhausts phylogenomic strategies to identify and characterize the molecular underpinnings of these streamlined organelles. To define the first proteome of ESVs and PVs we used a novel strategy combining flow cytometry-based organelle sorting with *in silico* filtration of mass spectrometry data. From the limited size datasets we retrieved many hypothetical but also known organelle-specific factors. In contrast to PVs, ESVs appear to maintain a strong physical and functional link to the ER including recruitment of ribosomes to organelle membranes. Overall the data provide further evidence for the formation of a cyst extracellular matrix with minimal complexity. The mass spectrometry proteomics data have been deposited to the ProteomeXchange Consortium with the dataset identifier PXD000694.

## Introduction

As the leading cause for protozoal diarrhea worldwide, the small intestinal parasite *Giardia lamblia (syn. G. duodenalis, G. intestinalis)* is an important pathogen of humans and animals causing significant morbidity and economic loss [1]. The *Giardia* life cycle is simple and consists of trophozoites, which multiply by binary fission in the gut of animal and human hosts, and an infectious cyst stage. Trophozoites attach actively to the epithelium of the small intestine and exhibit antigenic variation of variant surface proteins (VSPs) in their protein surface coat [2], [3]. Triggered by environmental cues (e.g. bile concentration, bioavailability of lipids, pH) trophozoites undergo a complex stage-differentiation process and transform to environmentally resistant cyst forms. The complete life-cycle, including cyst formation and excystation, can be reproduced *in vitro*.

Giardia belongs to the phylum Diplomonadida, unicellular eukaryotes that have undergone considerable reductive evolution resulting in minimization or even loss of most cellular systems such as mitochondria, peroxisomes, a Golgi apparatus, and a classical endo-lysosomal system. Despite this unusual organization 3 giardial organelle systems are clearly discernible: the endplasmic reticulum (ER) which extends bilaterally through the cell body [4], relic mitochondria (mitosomes), localized at the cell center but also dispersed in the cytoplasm [5], [6], and peripheral vesicles (PVs). PVs are ∼150 nm compartments with a fixed localization underlying the plasma membrane on the dorsal side and are also present in a specialized region of the ventral disk [7], [8]. PVs have been dubbed endosomal-lysosomal compartments based on localization of hydrolase activity [7], [9]–[11], their ability to acidify [10], [12], [13], and to take up exogenous ferritin as well as fluid phase markers [12], [14]–[16]. While in classical eukaryotic systems endosomes undergo organelle maturation, a similar process is not observed for PVs in Giardia. A selective pathway sorting proteins from the plasma membrane to PVs has been demonstrated [13], [17]–[19], and there is experimental evidence for a direct but selective connectivity between PVs and the ER [8]. PVs are thought to be the major route of nutrient uptake by the parasite, but the range of their functions, their morphogenesis and propagation remain unclear. Additional open questions concern the exact mechanism of bulk fluid uptake into the organelles and how endocytic cargo is sorted and trafficked to the ER.

Constitutive secretion of giardial proteins does not require a Golgi apparatus. As a consequence, secreted proteins are exported directly from the ER to target organelles such as PVs or the plasma membrane [20]. Organelles with Golgi properties (encystation-specific vesicles, ESVs) are generated *de novo* exclusively for regulated export of the cyst wall biopolymer consisting of three paralogous cyst wall proteins (CWP1–3) [21]–[23], and a unique β(1–3)-GalNAc homopolymer glycan [24], [25]. ESV formation is induced by COPII-dependent export of cyst wall proteins from ER exit sites [26], [27]. CWPs partition into two biophysically distinct phases before being sorted and secreted sequentially to build the two layers of the composite cyst wall polymer as an extracellular matrix 20–24 h post induction (p.i.) *in vitro* [28]. Although current data strongly support the hypothesis that ESVs are Golgi-derived organelles, the molecular underpinnings of ESV neogenesis and their identity as post-ER organelles remain controversial.

As with most systems and molecular machineries in diplomonads, PV and ESV organelles can be classified in terms of function but are highly divergent, i.e. there is some experimental evidence for their respective roles in the cell, but the paucity of molecular and morphological landmarks for organelle structure and function has prevented a systematic and detailed characterization. Nevertheless both organelle systems are essential and as such represent potentially vulnerable structures of this highly adapted parasite. The significant reductive evolution and sequence divergence in Giardia also means that strategies using homology-based identification and functional analysis of PV and ESV proteins [29]–[31] are biased towards few identifiable factors. The aim of this study was to identify novel factors that are specifically associated with these organelles and that may help characterize their nature, range of function, and evolutionary history in the context of the giardial ecological niche. To address these questions we developed a conceptually new approach to generate enriched organelle proteome datasets in two steps: (i) simultaneous flow cytometry-based sorting of a mixed microsome fraction containing green fluorescent ESV organelles with CWP3-GFP (green fluorescent protein) in condensed cores and red labeled peripheral vesicles (PV); (ii) mass spectrometry analysis and subtraction of overlapping hits to increase identification of organelle-specific candidates. Detailed analysis of the datasets and localization of selected candidate proteins suggests a close association of ESVs with the ER and no evidence for additional cargo or organelle-specific factors involved in their genesis and maturation. Conversely, although direct connections with the ER have been demonstrated, PVs appear to have a discrete compartment identity with a specific set of organelle proteins.

## Results

Giardia has an extensive ER [4] which stretches almost throughout the entire cytoplasm and to the cell periphery. Giardial organelle preparations for proteomic analysis are uniformly contaminated with membrane and membrane associated ER proteins [32], [33]. To overcome this we developed and implemented a new strategy to investigate the proteome of two organelle sets which are known to be in close proximity to and even in direct contact with the ER: ESV organelles [26], [27] and PV organelles [8]. After cell disruption we simultaneously enriched differentially labeled ESV and PV organelles by fluorescence-assisted organelle sorting (FAOS) from a mixed microsome fraction. We reasoned that the simultaneously sorted vesicle fractions would contain comparable amounts of mostly cytoplasmic and ER-derived unspecific proteins, and that this overlap could be subtracted from the respective mass spectrometry (MS) datasets *in silico* to reveal organelle-specific proteins.

### Enrichment and Separation of Fluorescently Labeled Organelles by Flow Cytometry

**Figure 1:**
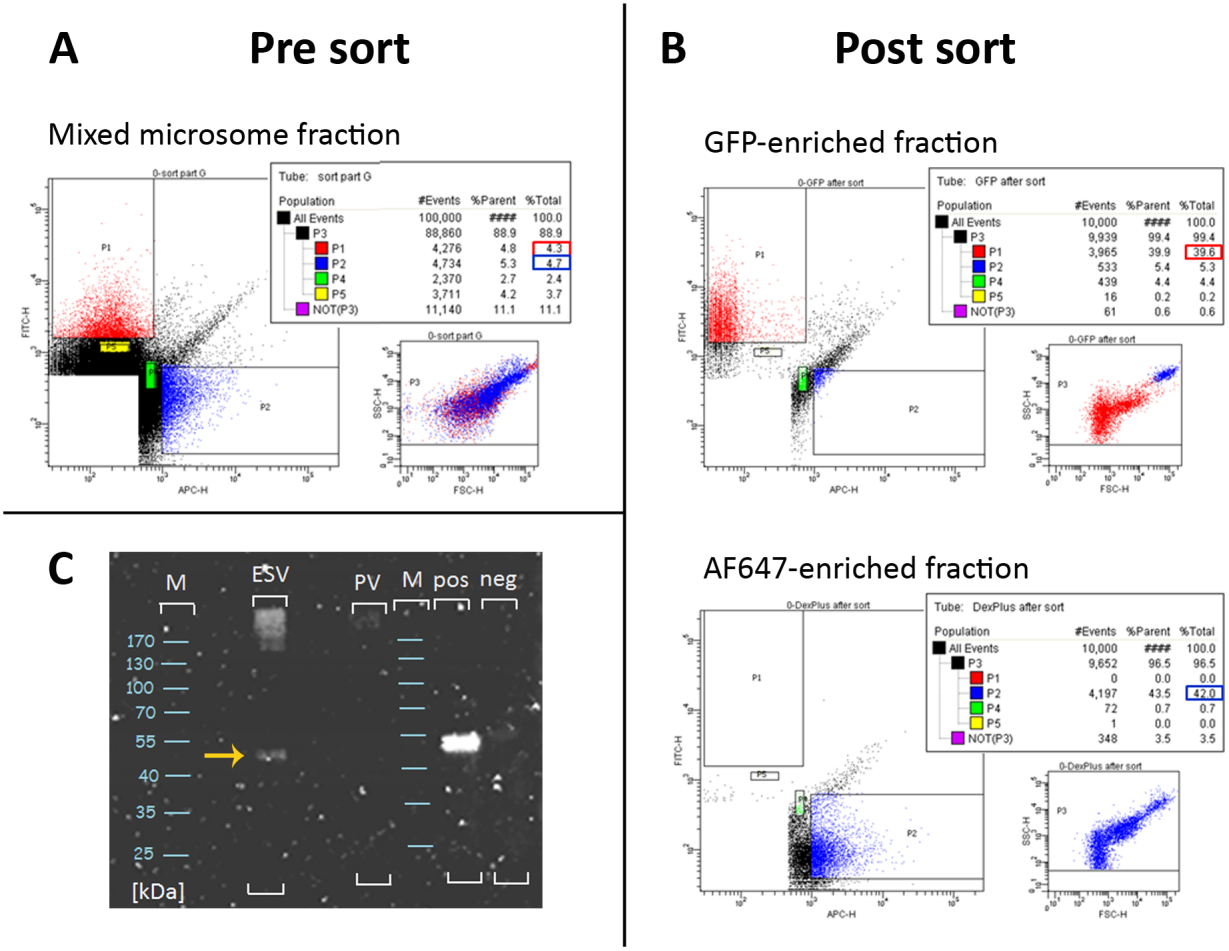
Flow cytometry based organelle sorting. **A**) Pre-sort flow cytometry analysis: 10e5 events of a mixed microsome fraction containing 4.3% GFP-positive events (ESV organelles) and 4.7% AF647-positive events (PV organelles). SSC/FSC scatter plot: P3, parent gate (organelle population); P1 (red) GFP-positive-, and P2 (blue) AF647-positive events. P4 and P5 (controls) were set to randomly collect additional material for later protein precipitation test runs. **B**) Post sort analysis shows enrichment of GFP-positive events (39.6%; B, top) and AF647-positive events (42.0%; B, bottom). **C**) Western blot analysis of enriched organelle fractions (post-sort precipitates) detecting GFP shows a distinct band for CWP3-GFP (53 kDA, arrow) in the ESV post sort fraction, but not the PV post sort fraction. *M:* protein size ladder (kDa); *pos, neg:* Positive and negative controls from an ESV-fraction and a non-ESV-fraction (control) of a sucrose density gradient centrifugation experiment performed with 13h encysting Giardia cells expressing CWP3-GFP [28].

We used flow cytometry to sort differentially labeled ESVs and PVs simultaneously from a mixed microsome fraction. The organelle fraction for sorting was prepared by mixing two microsome fractions derived from trophozoites with labelled PVs (Dextran-AlexaFluor647, AF647) and transgenic encysting cells with labelled ESVs (CWP3-GFP). Cell labeling, harvesting, and disruption were performed in three completely independent experiments (biological replicates). The sorts were performed on a BD FACSAriaIII flow cytometer using a sort precision mode of 0/32/0 to obtain maximal purity. Three gates were set: a very broad parent gate P3 in the SSC (side scatter) vs. FSC (forward scatter) plot that excluded the readily apparent measurement noise, and gates P1 and P2 in a bivariate dot plot to define the GFP-positive and AF647-positive events, respectively (Figure 1A). The target events in the mixed microsome fraction were 4.3% GFP-positives and 4.7% AF647-positives, corresponding to ESV and PV organelles, respectively.

Post-sort quality control by flow cytometry showed more than eight-fold increase of both, GFP-positive events (39.6%, Figure 1B, top) and AF647-positive events (42%, Figure 1B, bottom). In the post sort analysis 10^4^ events of the AF647-enriched fraction contained no GFP-positive events, i.e. ESV organelles (Figure 1B, bottom, gate P1). Likewise, analysis of 10^4^ events of the GFP-enriched fraction contained no AF647-positive events, i.e. PV organelles, with a scatter profile corresponding to that of the pre-sort sample (Figure 1B, top, gate P2); the cluster of events extending diagonally and between the GFP and AF647 fraction was apparent in all samples analyzed, both pre- and post-sort. Comparisons of normal buffer preparations (used across all experiments) with more meticulously prepared sample buffers and sheath fluid (manually filtered through liquid filters with a pore size of 220 nm) led us to conclude that these events do not correspond to ESV or PV target organelles, but instead predominantly represent particulates of very small size. Taken together, GFP- and AF647-positive events were completely separable, and we achieved a 100% relative enrichment using this approach.

To confirm the separation of labeled organelles, we detected GFP in post-sort precipitates (Figure 1C) using Western blot analysis. Using an anti-GFP antibody, a band between 40 and 55 kDa corresponding to the predicted CWP3-GFP fusion protein (53 kDa) was detected in the ESV-enriched fraction but not in the PV-enriched fraction. An additional GFP-signal was detected in the high molecular weight area in the ESV-enriched fraction, presumably corresponding to insoluble CWP3-GFP aggregates from condensed cores of ESVs or covalently linked homo-multimers. Taken together with the data obtained by post sort flow cytometry, we concluded that the two organelles could be quantitatively separated which was a prerequisite for the subsequent subtractive analysis.

### Mass Spectrometry Analysis of Organelle-enriched Fractions

**Figure 2:**
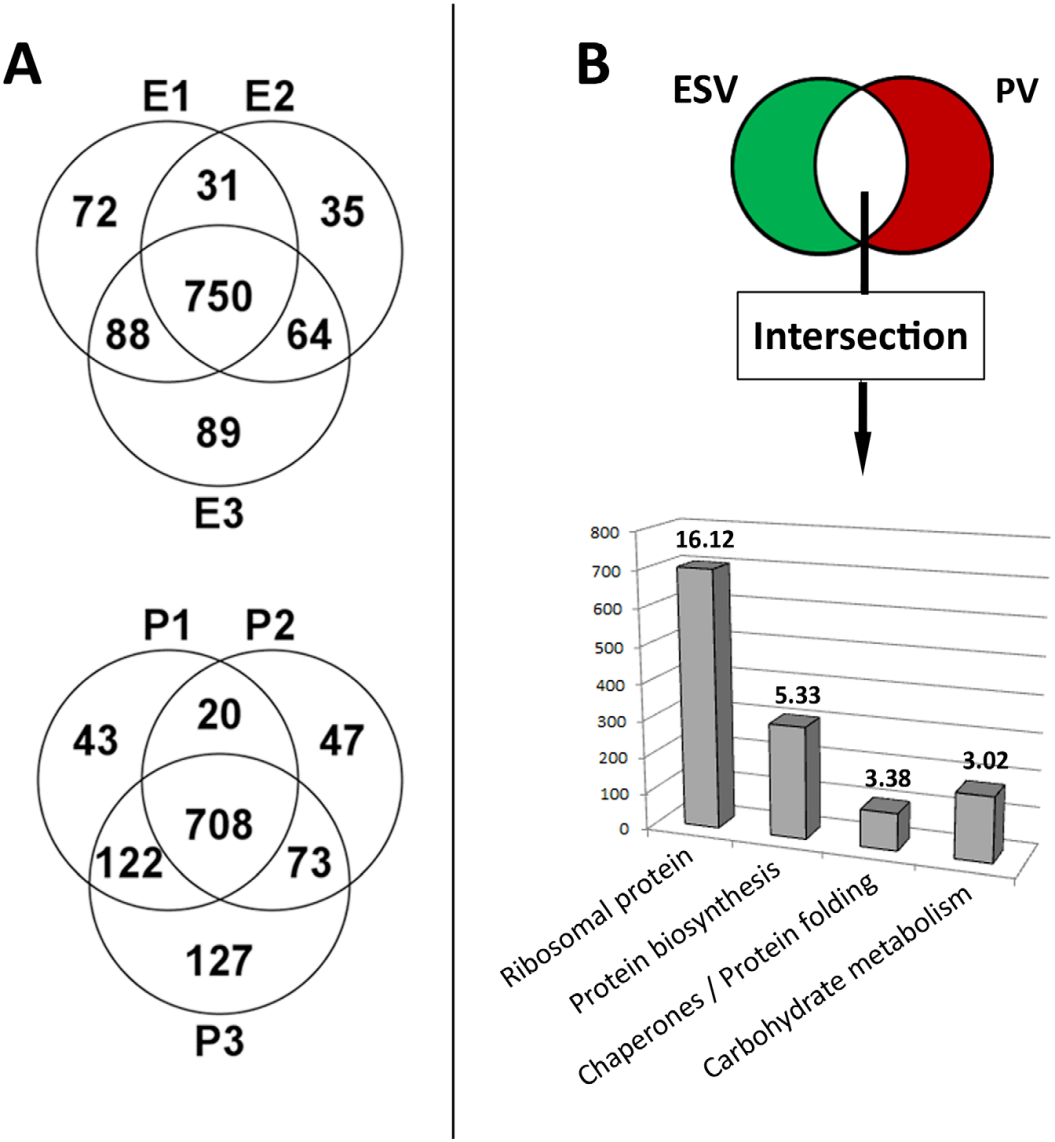
Reproducibility of biological triplicates and annotation clustering of intersection. **A**) VENN diagrams for ESV (E1, E2, E3) and PV (P1, P2, P3) mass spectrometry datasets (total n = 1281). ESV datasets (A, top) n = 1129 with overlaps. 750 proteins (66%) were detected in all three datasets. PV datasets (A, bottom) n = 1140 with overlaps. 708 proteins (62%) were detected in all three datasets. **B**) Clustering analysis of 1059 proteins defined by the E1-E3 and P1-P3 data intersection (see Figures S1 and S2) using the DAVID bioinformatics tool [34]. *X-axis:* functional clusters with an enrichment score above 3; *y-axis:* number of proteins; value in bold on top of column: enrichment score. A detailed summary of all 14 clusters can be found in the Text S2 and S3.

Sorted organelle-enriched fractions were analyzed by mass spectrometry using a shotgun approach combining 1D–SDS-PAGE and LC ESI-MS/MS (liquid chromatography electrospray ionization - tandem mass spectrometry). With a Mascot ion cut-off score of 20 for peptide-spectrum matches, a minimum of 2 unique peptides, and a protein probability of 80%, a total of 1281 proteins were identified in the combined triplicate ESV and PV fractions. After subtraction of environmental contaminations, e.g. keratins, 1213 *G. lamblia* proteins remained. A false discovery rate (FDR) of 0.0% for peptides and 0.5% for proteins was calculated by Scaffold. In the unified ESV fractions (E1+E2+E3) a total of 1129 proteins were identified; 750 (66%) thereof were detected in all three samples and 933 (83%) in at least 2 of 3 samples (Figure 2A, top). Comparable numbers were found for PV organelles: of 1140 proteins identified in total (P1+P2+P3), 708 (62%) were detected in all three samples and 923 (81%) in at least 2 of 3 samples (Figure 2A, bottom). The large overlaps of the P and E datasets, respectively, demonstrated high reproducibility between replicate experiments. For a detailed compilation of identified proteins see Table S1. All mass spectrometry proteomic datasets have been deposited to the ProteomeXchange consortium (http://proteomecentral.proteomexchange.org) and are accessible with the dataset identifier PXD000694 and DOI 10.6019/PXD000694.

### Subtractive Analysis Eliminates Many Predominantly Translation-associated and ER-derived Contaminants

Simultaneous sorting of two differentially labeled organelles (ESVs and PVs) from a mixed microsome fraction allows subtracting the unspecific background of unlabeled soluble proteins as well as small cell debris contained within the positively sorted droplets which are generated by the vibrating nozzle of the cell sorter. The premise was that by eliminating all proteins common to the ESV and PV datasets (intersection) the organelle-specificity of each dataset would increase significantly. In particular, the occurrence of ER-derived contaminants, which have been a severe problem in all previous attempts to enrich Giardia organelles, should be strongly reduced.

The total 1213 hits in all replicate mass spectrometry datasets contained 1059 putative contaminants, defined by the E1-E3 and P1-P3 data intersection, whilst 72 proteins were considered ESV-specific and 82 PV-specific (Figure 2B, top). A detailed description of the workflow and the *in silico* identification of the data intersect can be found in the Figures S1 and S2. This relatively high ratio of contaminants to organelle-specific proteins was not surprising considering the results of previous cell fractionation experiments and the analysis of organelle fractions by SDS-PAGE in this study (not shown).

### Analysis of the Eliminated ESV and PV Dataset Intersection

A more detailed analysis of the two organelle-specific and the large intersecting datasets was performed using the DAVID bioinformatics tool [34]. From 1059 proteins in the dataset intersection we removed an additional 28 with obsolete gene models. Of the 1031 remaining proteins 903 could be assigned to 14 different DAVID clusters (enrichment score >1), while 128 proteins could not be clustered. 4 of the 14 DAVID clusters showed an enrichment score of >3 (Figure 2B, bottom). The top ranking clusters were “ribosomal proteins” (enrichment score 16.12, 701 proteins), “protein biosynthesis” (enrichment score 5.33, 305 proteins), “chaperones/protein folding” (enrichment score 3.38, 101 proteins) and “carbohydrate metabolism” (enrichment score 3.02, 176 proteins). A corresponding genome-wide analysis as a reference using a total of 5150 validated genes yielded on average 79 clusters per 3′000 gene models, with the vast majority of enrichment scores <1 and none >2. See also supplementary data (Text S2 and S3) for a detailed description of all DAVID analyses results. Taken together, this demonstrates the relative enrichment for translation-associated and ER-derived factors in the data intersection, supporting the idea that subtraction of these hits will increase the specificity of organelle datasets. Conversely no obvious clusters were detected in the control datasets representing a genome-wide sampling.

### Parsing and Manual Annotation of ESV and PV Organelle Specific Proteins

**Figure 3:**
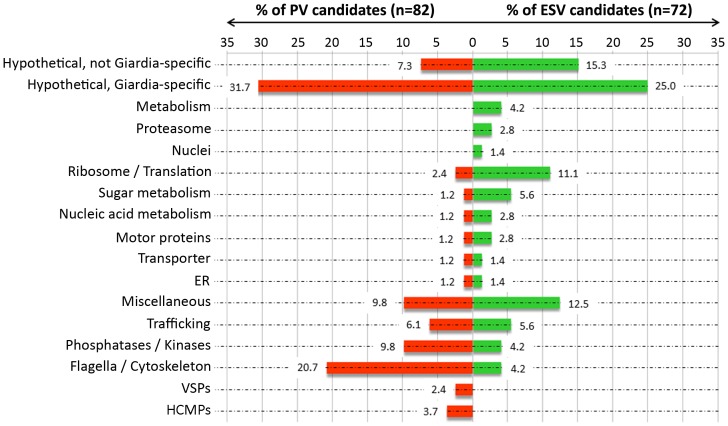
Categorization of the 72 ESV and 82 PV candidates. 17 categories based on predicted function were used. The percentage of candidates in each group is indicated at the end of the columns. ESV candidates (green) and PV candidates (red) fall into 15 and 14 groups, respectively. *ER*: endoplasmic reticulum; *VSPs*: variant surface proteins; *HCMPs*: high cysteine membrane proteins. Detailed information on the candidates in each category can be found in the Table S2.

The large majority of gene models and protein annotations in the Giardia Genome Database (GiardiaDB) are based on automated predictions. For a manual annotation of organelle-specific datasets we used function and homology prediction programs (PSORTII, TMHMM, SMART, pBLAST and HHPred) to identify putative signal peptides, transmembrane domains, protein functional domains and homologies (Table 1 and Table S2). In total we suggest re-annotation of 29 genes (listed in Table S2). For parsing, 17 categories were used (Figure 3) based on predicted function or localization according to the criteria described in this study or in previous reports [30].

**Table 1:** ESV and PV candidate list.

ESV candidates (n = 72)				PV candidates (n = 82)			
Cat.	GeneID	Product description	Loc.	Cat.	GeneID	Product description	Loc.
HGS	Gl2014	Hypothetical protein		HGS	Gl13651	Hypothetical protein	
HGS	Gl7350	Hypothetical protein	cy	HGS	Gl15918	Hypothetical protein	
HGS	Gl9007	Hypothetical protein		HGS	Gl16811*	Hypothetical protein	
HGS	Gl9157	Hypothetical protein	cy	HGS	Gl17468	Hypothetical protein	
HGS	Gl10221	Hypothetical protein	er	HGS	Gl29119	Hypothetical protein	
HGS	Gl10568	Hypothetical protein		HGS	Gl6535	Hypothetical protein	
HGS	Gl14235	Hypothetical protein		HGS	Gl9573	Hypothetical protein	
HGS	Gl14458	Hypothetical protein	pe, ex, es	HGS	Gl13774	Hypothetical protein	
HGS	Gl16522	Hypothetical protein		HGS	Gl13783	Hypothetical protein	
HGS	Gl22136	Hypothetical protein	er	HGS	Gl16926	Hypothetical protein	
HGS	Gl24453	Hypothetical protein		HGS	Gl17330	Hypothetical protein	
HGS	Gl25205	Hypothetical protein	er, es	HGS	Gl17571*	Hypothetical protein	
HGS	Gl32419	Hypothetical protein	er, ex, es	HGS	Gl24451	Hypothetical protein	
HGS	Gl87926	Hypothetical protein	vd	HGS	Gl3920	Hypothetical protein	
HGS	Gl90434	Hypothetical protein		HGS	Gl4852	Hypothetical protein	
HGS	Gl113722	Hypothetical protein		HGS	Gl5890	Hypothetical protein	
HGS	Gl137705	Hypothetical protein		HGS	Gl6334	Hypothetical protein	
HGS	Gl137712	Hypothetical protein		HGS	Gl10181	Hypothetical protein	
HNS	Gl5568	Hypothetical protein		HGS	Gl10524	Hypothetical protein	
HNS	Gl8799	Hypothetical protein		HGS	Gl10608	Hypothetical protein	
HNS	Gl11246	Hypothetical protein		HGS	Gl112893	Hypothetical protein	
HNS	Gl13262	Hypothetical protein		HGS	Gl14971	Hypothetical protein	
HNS	Gl14345	Coiled-coil protein		HGS	Gl16543	Hypothetical protein	
HNS	Gl14877	Hypothetical protein		HGS	Gl4018	Hypothetical protein	
HNS	Gl15956	WD-40 repeat protein	er	HGS	Gl4270	Hypothetical protein	pm, cy, pv?
HNS	Gl22806	Hypothetical protein		HGS	Gl17347	Hypothetical protein	
HNS	Gl23357*	Hypothetical protein		HNS	Gl91354*	Hypothetical protein	
HNS	Gl88581	Synaptic glycoprotein SC2	er	HNS	Gl10522	Hypothetical protein	
HNS	Gl94654	Hypothetical protein		HNS	Gl16237	Hypothetical protein	
MET	Gl4059	Methylthioadenosine/S adenosylhomocysteine nucleosidase		HNS	Gl16367	Hypothetical protein	
MET	Gl4507	CTP synthase/UTP-ammonia lyase		HNS	Gl16998	Hypothetical protein	
MET	Gl6757*	Isochorismatase		HNS	Gl7778	Hypothetical protein	
PRO	Gl7896	26S proteasome non-ATPase regulatory subunit 7		R/T	Gl11287	Ribosomal protein L7Ae	
PRO	Gl16823	Non ATPase subunit MPR1 of 26S proteasome		R/T	Gl14869	Ribosomal protein L24	
NUC	Gl7474	DNA-directed RNA polymerase RPB3		SUG	Gl104031	Glycogen synthase, putative	
R/T	Gl9899*	putative eIF2A		NUA	Gl16328*	Pseudouridine synthase	
R/T	Gl10341*	putative 50S ribosomal protein L1		MOT	Gl13825	Kinesin-1	
R/T	Gl10780	Ribosomal protein S27		TPO	Gl40224	Multi drug resistance (MDR) protein-like protein	er, pe
R/T	Gl11319	U3 snRNP IMP3, putative		ER	Gl14856	Signal recognition particle (SRP) receptor	
R/T	Gl11755*	30S ribosomal protein S8E		MIS	Gl3095*	Cyclin_N domain containing protein	
R/T	Gl15156	Signal recognition particle 54 kDa (SRP54) GTPase	cy, er, es	MIS	Gl8394*	RdX-domain containing protein	
R/T	Gl15546*	putative eIF3		MIS	Gl32697*	Methyltransferase-domain containing protein	
R/T	Gl40521*	putative eIF2A		MIS	Gl24979*	Thioredoxin domain containing protein	
SUG	Gl8382*	Putative UDP-GlcNAc-4′-epimerase (GALE)	er	MIS	Gl2013	Glutaredoxin-related protein	
SUG	Gl10324	Ribulose-phosphate 3-epimerase		MIS	Gl2933	Programmed cell death protein-like protein	
SUG	Gl11595*	Glycosyl transferase family 8 protein	cy	MIS	Gl5871	Developmentally regulated GTP-binding protein 1	
SUG	Gl15483*	UDP-GlcNAc transporter	er, pe, es	MIS	Gl8559	V-type ATPase, 16 kDa proteolipid subunit	er, pe
NUA	Gl16887	ATP-dependent RNA helicase HAS1, putative		TRA	Gl16521	Alpha-SNAP	pv
NUA	Gl113365	5′-3′ exoribonuclease 2		TRA	Gl15104	Sec1, putative	pv
MOT	Gl101138	Dynein heavy chain		TRA	Gl15339	Adaptor protein (AP) complex large chain subunit BetaA	er
MOT	Gl111950	Dynein heavy chain		TRA	Gl96994*	Qa3-SNARE	pv
TPO	Gl11299	Amino acid transporter, putative	er	TRA	Gl15472*	Vacuolar protein sorting (VPS) 46a	cy, pm? pv?
ER	Gl101339	FKBP-type peptidyl-prolyl cis-trans isomerase		P/K	Gl11554	Kinase, NEK	
MIS	Gl6185*	Nucleoside triphosphatase (NTPase)		P/K	Gl15935	Kinase, NEK	
MIS	Gl7207*	Calcium-binding protein	cy	P/K	Gl17231	Kinase, NEK	
MIS	Gl8524*	AAA (ATPases Associated with diverse cellular Activities) ATPase		P/K	Gl2661	Cyclin-dependent kinase (CdK) regulatory subunit	
MIS	Gl9594	Hsp70 binding protein		P/K	Gl17069	Kinase, NEK	
MIS	Gl14277	N-acetyltransferase-like protein		P/K	Gl2053	Ser/Thr protein phosphatase 4	
MIS	Gl14604*	Cytosolic Fe-S cluster assembling factor NBP35		P/K	Gl17556	Kinase, CAMK, CAMKL	
MIS	Gl32838*	Nitrogen fixation protein NifU		P/K	Gl9894	Protein phosphatase 2A (PP2A) regulatory subunit, putative	
MIS	Gl113143	Lipopolysaccharide-responsive and beige-like anchor protein		F/C	Gl11164	Protein 21.1	
MIS	Gl9058*	Putative Type 2A phosphatase-associated protein 42 (TAP42)		F/C	Gl11165	Protein 21.1	
TRA	Gl8049*	Putative importin alpha		F/C	Gl113622	Protein 21.1	
TRA	Gl15204	ERP3		F/C	Gl16532	Protein 21.1	
TRA	Gl17109	Vacuolar protein sorting 11 (VPS11)		F/C	Gl17046	Protein 21.1	
TRA	Gl137698	Sec13		F/C	Gl23492	Protein 21.1	
P/K	Gl14545*	putative Ser/Thr-protein phosphatase 2A (PP2A)		F/C	Gl6744	Centrin	
P/K	Gl14661*	Ser/Thr protein kinase		F/C	Gl95192	Protein 21.1	
P/K	Gl32312	Protein phosphatase 2C (PP2C)		F/C	Gl9750	Intraflagellar transport protein component IFT74/72	
F/C	Gl4026	Alpha-19 giardin		F/C	Gl10038	Alpha-18 giardin	
F/C	Gl15587	Protein 21.1		F/C	Gl10219	Protein 21.1	
F/C	Gl24009	Protein 21.1		F/C	Gl103807	Protein 21.1	
				F/C	Gl13766	Protein 21.1	
				F/C	Gl14926	Protein 21.1	
				F/C	Gl16220	Protein 21.1	
				F/C	Gl14745	Protein 21.1	
				F/C	Gl15428	IFT complex B	
				VSP	Gl33279	VSP	
				VSP	Gl13194	VSP AS8	
				HCM	Gl15317	HCMP Group 1	
				HCM	Gl24880	HCMP Group 2	
				HCM	Gl103454	HCMP Group 1	

72 ESV and 82 PV candidate lists. The subcellular localization of 16 (ESV) and 8 (PV) candidates determined in this study are indicated. Additional information including GenBank accession numbers can be found in Table S2. *Cat:* category; *GeneID:* Gene accession number according to the *G. lamblia* genome database; *Loc:* localization determined in this study; *n:* number; *er:* endoplasmic reticulum; *pe:* perinuclear ER; *ex:* ER exit sites; *es:* encystation-specific vesicles; *cy* cytoplasm; *vd:* ventral disc; *pv:* peripheral vesicles*; pm:* plasma membrane. *HGS:* hypothetical protein Giardia-specific; *HNS:* hypothetical protein not Giardia-specific; *MET:* metabolism; PRO: proteasome; *NUC:* nuclei; *R/T:* ribosome/translation; *SUG:* sugar metabolism; *NUA:* nucleic acid metabolism; *MOT:* motor proteins; *TPO*: transporter; *ER*: endoplasmic reticulum; *MIS:* miscellaneous; *TRA:* trafficking; *P/K:* phosphatases/kinases; *F/C:* flagella/cytoskeleton; *VSP:* variant-specific surface protein; *HCM, HCMP:* high-cysteine membrane protein, *eIF:* eukaryotic initiation factor; *snRNP:* small nuclear ribonucleoprotein; *SNAP:* soluble N-ethylmaleimide-sensitive-factor (NSF) attachment protein; *SNARE:* soluble N-ethylmaleimide-sensitive-factor (NSF) attachment receptor; *GlcNAc:* N-Acetylglucosamin; *NEK:* NimA related kinase; *: re-annotated.

Figure 3 shows a graphical representation of the parsed ESV proteins in 15 categories with 29 hypothetical proteins remaining. Eleven of those (15.3%) have predicted homologs in other species, whereas 18 (25.0%) are considered Giardia-specific. The 82 PV proteins were parsed into 14 categories, with 32 candidates designated as hypothetical proteins and 26 thereof (31.7%) considered Giardia-specific. Direct comparison of the ESV and PV datasets (Figure 3) revealed a similar proportion of proteins with no functional prediction, i.e. hypothetical proteins (40.3% and 39.0%, respectively). Parsing of the data revealed clear differences in categories, with 3 and 2 groups appearing exclusively in the ESV and PV datasets, respectively (Figure 3). Within the shared groups, the most striking differences were the overrepresentation of factors involved in protein translation (ESV: 11.1% versus PV: 2.4%) in the ESV dataset, or the enrichment of flagella/cytoskeleton proteins (ESV: 4.2% versus PVs: 20.7%) in the PV dataset.

### Detection of Known Organelle Proteins in ESV and PV Datasets

Fewer than 20 factors are known to associate to ESVs at any given point during ESV neogenesis and maturation [10], [16], [26], [29], [32], [35]–[39]. Only 3 localize exclusively to ESVs at 13h post induction (p.i.): CWP1 and the large fragment of the proteolytically processed CWP2 in the fluid phase fraction, and CWP3 together with the small fragment of CWP2 in the condensed core [28]. CWP-derived tryptic peptides are detected very inefficiently in MS most likely due to the well-documented extensive intra- and intermolecular cross-linking by disulfide and isopeptide bonds [40], [41]. The proportion of DTT (dithiothreitol)-resistant high molecular weight complexes of CWP3-GFP can be estimated in the Western blot in Figure 1C (ESV). Nevertheless, peptides derived from the organelle marker CWP3-GFP or endogenous CWP3 were detected exclusively in ESV-enriched samples by MS (average quantitative value of 2.7) albeit only at a stringency of 1 unique peptide and 50% protein probability. GFP-derived peptides were detected in all 3 ESV-enriched samples with an average quantitative value of 1.7.

Of the 72 proteins in the ESV dataset, 4 are either known or predicted to associate to ESV organelles. In particular, we identified two COPII-coat components Sec13 (Gl137698) and Erp3 (Gl15204) (trafficking proteins, Figure 3, Table 1). The association of other COPII-components with ESVs, such as GlSar1 and GlSec31, during early development was observed previously [26], [29]. Additional ESV-associated factors are represented by 2 proteasome proteins (Figure 3, Table 1). Proteasome complexes are recruited to ESV membranes during early encystation, perhaps in connection with post-ER quality control and associated degradation processes [32]. It is important to note that with the exception of CWP1–3, all other previously described ESV-associated factors are also expressed in trophozoites. Since their expression is not strictly stage-specific and they are recruited to ESV organelles from other subcellular localizations during encystation, their absence in the ESV-specific dataset but detection in the data intersection instead is not surprising.

The 15 PV proteins which have been identified in trophozoites and encysting cells thus far include soluble N-ethylmaleimide-sensitive-factor attachment receptors (SNAREs), components of the clathrin/adaptor protein (AP)-mediated trafficking machinery, Rab11, an acidic phosphatase and an encystation-specific protease termed ESCP [13], [16]–[18], [29], [42], [43]. Since most of these PV-associated proteins are involved in vesicle trafficking, they have secondary localizations, i.e. the cytoplasm, the plasma membrane, the ER or ESVs. Not surprisingly, we recovered the majority of these trafficking proteins in the data intersect. However, the Qa3-SNARE homolog syntaxin1 (Gl96994) [29], [43] was specifically included in the PV dataset. In addition to this membrane trafficking factor we recovered 3 previously described PV-associated proteins including two VSPs (Gl33279, Gl13194) [44], [45] and, when lowering the stringency to 1 unique peptide, the encystation-specific cysteine protease ESCP (Gl14566) [13].

Taken together, we detected 5 known ESV-associated proteins, including our organelle marker CWP3-GFP, in the ESV dataset, whereas 4 known PV-associated factors were contained in the PV dataset. Because of their extensive cross-linking and post translational modifications CWP1 and CWP2, comprising the fluid component of the cyst wall material (CWM) in ESVs at 13 hours p.i., were detected only with relaxed stringency.

### Subcellular Localization of Candidate PV Proteins

**Figure 4:**
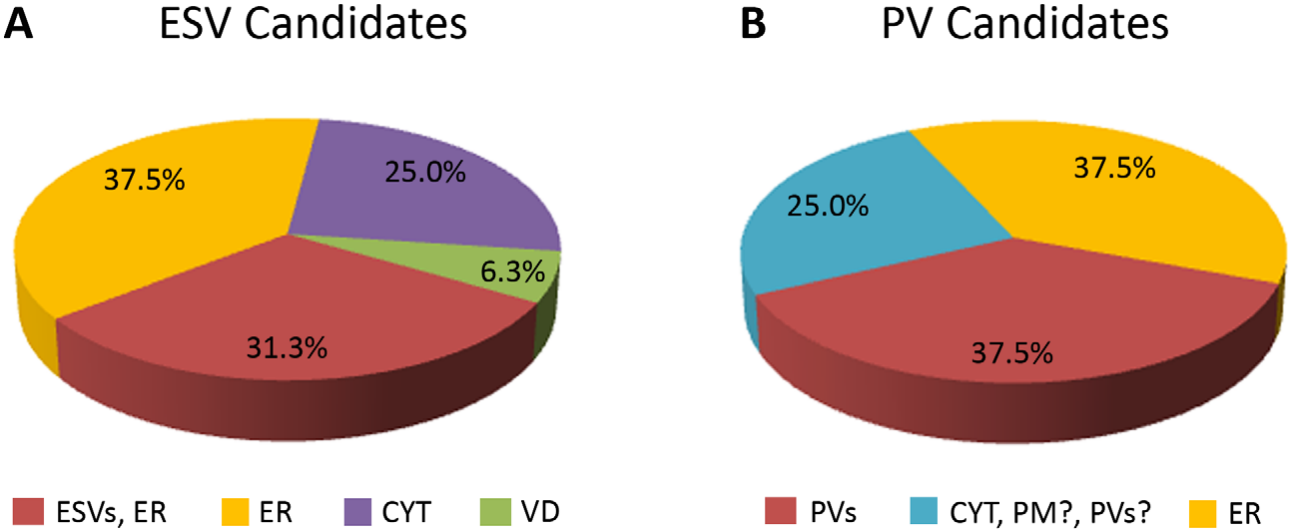
Subcellular localization of selected ESV and PV candidates. 16 (ESV candidates) and 8 (PV candidates) HA-tagged variants were localized by immunofluorescence analysis in transgenic cells. **A**) Subcellular localization of 16 ESV candidates in trophozoites at 13 hours p.i. **B**) Subcellular localization of 8 PV candidates. Detailed information on the respective candidates can be found in Table 1 and Table S2. *ER:* endoplasmic reticulum; *ESVs:* encystation-specific vesicles; *CYT*: cytoplasm; *VD*: ventral disc; *PVs:* peripheral vesicles; *PM;* plasma membrane.

**Figure 5:**
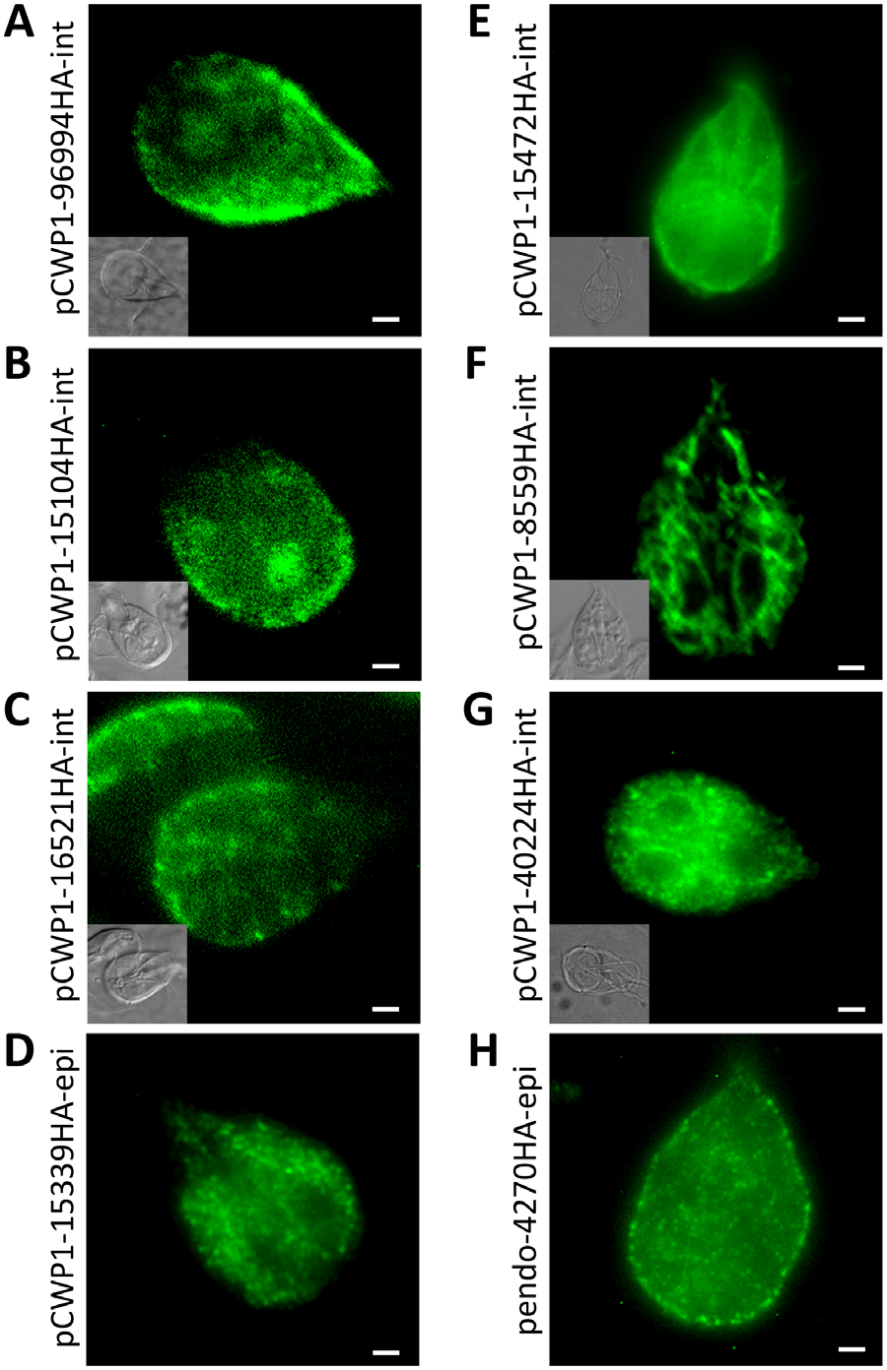
Subcellular localization of 8 selected PV candidates. Representative localization of C-terminally HA-tagged variants after constitutive expression in trophozoites (H) or 8–12 hours induced expression (A-G). **A, B, C**) Gl96994HA (Qa3-SNARE), Gl15104HA (Sec1 homolog), and Gl16521HA (putative alpha-SNAP) localize to PVs. **D**) Gl15339HA (beta adaptin) localizes to the ER. **E**) Gl15472HA (VPS46 homolog) localizes to the cytoplasm and shows intense signal near the cell surface. **F, G**) Gl8559HA (v-type ATPase) and Gl40224HA (MDR-like protein) localize to the perinuclear region and the ER in trophozoites. **H**) Gl4270HA (hypothetical protein) localizes to vesicle-like structures at the plasma membrane and in the cytoplasm. Localization to PVs cannot be excluded. *Antibodies:* anti-HA high affinity from rat, Alexa488-conjugated goat anti-rat (green), alternatively rat anti-HA-FITC (green). *pCWP1:* inducible CWP1 promoter; *pendo:* endogenous promoter; *int:* stable integration into the genome; *epi:* episomal maintenance of the plasmid. Scale bar: 1.5 µm.

As a first partial validation of the PV-derived dataset and to determine whether novel PV proteins are contained within the dataset, we expressed 8 proteins as C-terminally hemagglutinin (HA)-tagged variants in *G. lamblia* (Table 1, Figure 4b). In addition to proteins involved in membrane traffic, we focused on additional predicted transmembrane or membrane interacting proteins. We localized 3 tagged candidates involved in SNARE-mediated membrane fusion: (i) a Qa3-SNARE (Gl96994) homologous to syntaxin 1a [29], [43] (ii) a putative Sec1 protein (Gl15104) which forms heterodimers with syntaxin 1a [46] and (iii) a putative alpha-soluble N-ethylmaleimide-sensitive factor attachment protein (alpha-SNAP) (Gl16521). All 3 reporters localized predominantly to the cortical area of trophozoites, consistent with an association to PVs (Figure 5A–C and Figure S5). Our localization data of the Giardia Qa3-SNARE are in agreement with previous studies localizing this protein to PVs [43].

We localized two proteins with a predicted function in endocytic/endosomal transport: the adaptor protein (AP) large chain subunit BetaA (Gl15339) and a VPS (vacuolar protein sorting) 46a homolog (Gl15472), a putative component of a giardial endosomal sorting complex required for transport (ESCRT) III complex [47]. Localization of the tagged AP large chain subunit revealed a distribution consistent with the Giardia ER (Figure 5D). This result is partially consistent with earlier studies detecting an AP1-subunit in PVs and the ER, and the AP2-subunit u2 in PVs and the plasma membrane [17], [18]. The tagged giardial VPS46a homolog was detected in the cytoplasm, and probably localizes also to the plasma membrane and/or to PVs (Figure 5E).

Subcellular localization studies on the vacuolar ATP synthase subunit (Gl8559, Figure 5F) and the multidrug resistance (MDR)-like protein (Gl40224, Figure 5G) showed perinuclear staining and distribution typical of the Giardia ER. One hypothetical protein (Gl4270, Figure 5H) was detected in vesicle-like structures at the plasma membrane and in the cytoplasm. Whether these structures correspond indeed to PVs will require further investigation.

### Subcellular Localization of Candidate ESV Proteins

To validate the ESV-organelle dataset, 16 proteins were chosen for ectopic expression as C-terminally HA-tagged variants in *G. lamblia* (Table 1, Figure 4a) based on the following criteria: (a) Giardia-specific hypothetical proteins since they are unique to Giardia and might associate with ESVs; (b) proteins whose mRNAs were significantly upregulated during encystation [48], representing putative key factors in differentiation; and (c) proteins with predicted transmembrane domains and/or signal peptides, as they may be trafficked to ESVs. In addition, we wanted to localize proteins involved in (d) sugar metabolism or (e) sugar transport as these may represent key factors required for cyst wall glycan synthesis. Another category (f) was composed of predicted transporters which may be involved in direct import of substrates into ESV organelles from the cytoplasm. We also determined the subcellular localization of (g) a newly identified calcium-binding protein. Finally, we selected (h) a signal recognition particle component to test for recruitment of complexes for co-translational insertion of proteins across the ER and ESV membranes, as suggested by the electron microscopy data (see below in “Factors for co-translational protein insertion”).

Subcellular localization studies of 16 ESV candidate proteins revealed 11 candidates which had a distribution consistent with ER localization; 6 were detected exclusively in the ER while 5 also localized to ESVs. Four tagged candidates showed cytoplasmic localization and one candidate localized to the ventral disc (Table 1, Figure 4A).

#### A) Novel ESV proteins

**Figure 6:**
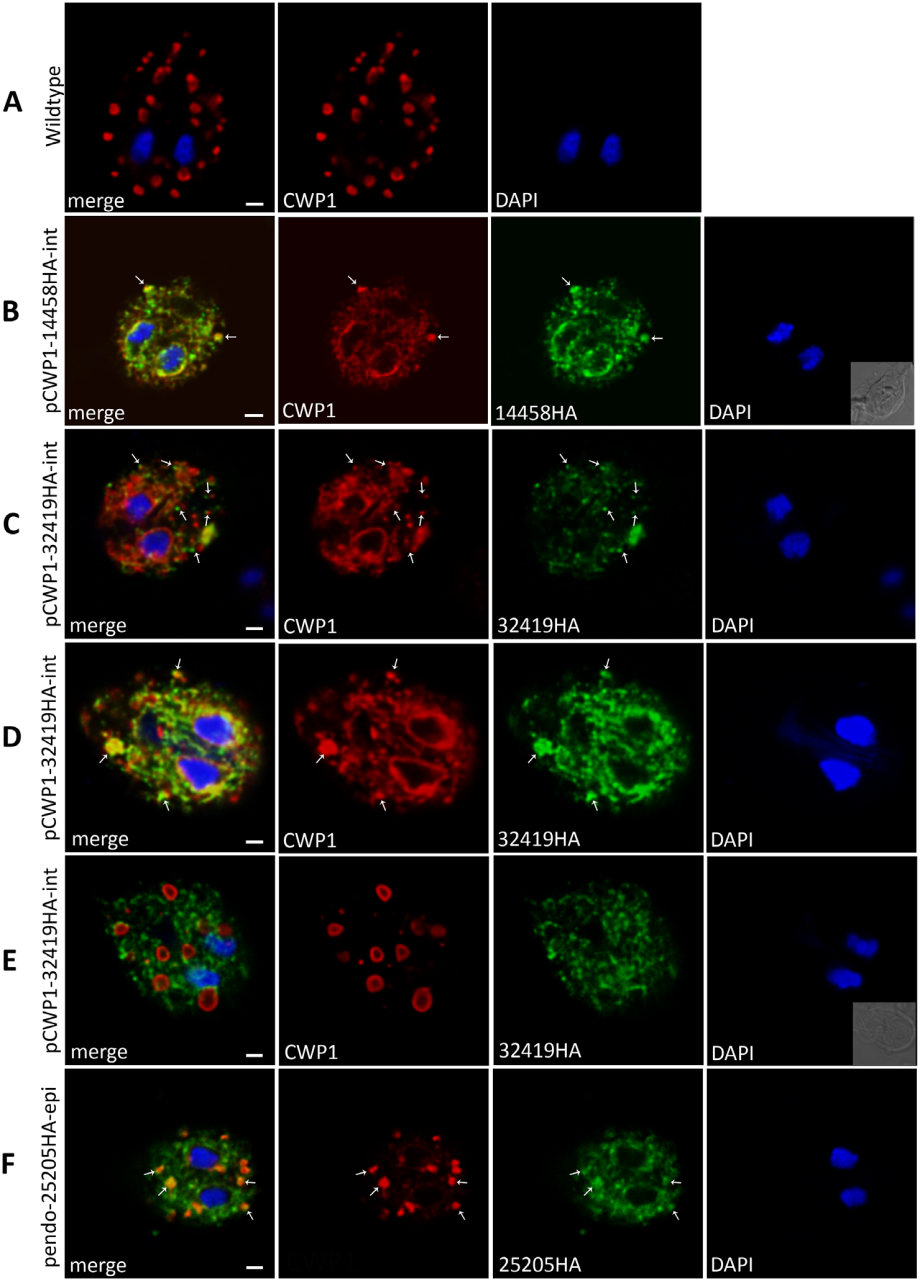
Subcellular localization of three ESV candidates: Gl14458HA, Gl32419HA, and Gl25205HA. Representative localization of C-terminally HA-tagged variants after inducible (Gl14458, Gl32419) or constitutive expression (Gl25205) by confocal microscopy at 13h p.i. Anti-CWP1 was used to detect ESV organelles. **A)** Wild type (WB) encysting trophozoite at 13h p.i.: CWP1 localized to “doughnut-shaped” ESVs which is typical for this time point [28]. No CWP1-signal in the perinuclear ER was visible. **B**) Gl14458HA at 13h p.i.: Gl14458HA was detected primarily in the perinuclear ER and overlaps with CWP1 in ESVs. Note the retention of CWP1 in the perinuclear ER and reduction of ESV numbers. In fact, the majority of induced cells in the population did not produce ESVs at all. **C, D, E**) Representative subcellular localizations of Gl32419-HA at 13h p.i. **C)** Partial signal overlap of Gl32419HA (green) with CWP1 (red) in knob-like structures, reminiscent of ESV neogenesis at ER exit sites [27]. Alternatively, Gl32419HA localized to the ER **D**) co-localizing with CWP1 primarily in the perinuclear ER and in ESVs suggesting delayed export of CWM to ESVs. **E**) In cells with canonical mature ESVs no signal overlap of Gl32419HA and CWP1 was observed. **F**) Gl25205HA at 13h p.i. was detected in the ER and in ESVs with occasional signal overlap with CWP1. *Antibodies:* anti-HA high affinity from rat, Alexa488-conjugated goat anti-rat (green), and Texas red-conjugated anti-CWP1 (red). Nuclear DNA was labeled with DAPI. *pCWP1:* inducible CWP1 promoter; *pendo:* endogenous promoter; *int:* stable integration into the genome; *epi:* episomal maintenance of the plasmid, *WB:* wildtype. Scale bar: 1.5 µm.

Only two ER-resident proteins are known to reach ESVs without being secreted to the surface of the cell: heat shock protein (Hsp) 70/Binding protein (BiP), which cycles between the ER and ESVs [32] and a subtilisin-like proprotein convertase termed gSPC [39]. Here we identify 3 additional hypothetical proteins that localize to the ER and ESVs during encystation: **(i)** At 13 h p.i., the 202 amino acid-long HA-tagged Gl14458 product was detected in the perinuclear ER, in ER-associated punctate structures reminiscent of ER exit sites (ERES), and overlapping with emerging ESVs in the cytoplasm (Figure 6B). Transgenic cells expressing Gl14458-HA showed a significant delay or, in most cells, a block of CWP1 export from the ER and a reduction in the number of ESVs compared with wild type cells (Figure 6A, B) or transgenic cells expressing unrelated HA-tagged products (data not shown). **(ii)** ORF Gl32419 is stage-specifically upregulated [49] and codes for a 564 amino acid protein of unknown function (Table S2) whose HA-tagged variant localizes in the ER, including structures reminiscent of ERES [27] as well as with maturing ESVs (Figure 6C, D). In cells with mature ESVs containing condensed cores the protein localized to ER membranes, in particular to those adjacent to ESVs (Figure 6C–E). **(iii)** The predicted Gl25205 product is a hypothetical Giardia-specific multipass membrane protein of 1246 amino acids with 14 hydrophobic domains. An epitope-tagged variant localized to the ER and in many cases also to morphologically normal ESVs at 13 hours p.i. (Figure 6F).

#### B) Factors involved in cyst wall glycan synthesis

**Figure 7:**
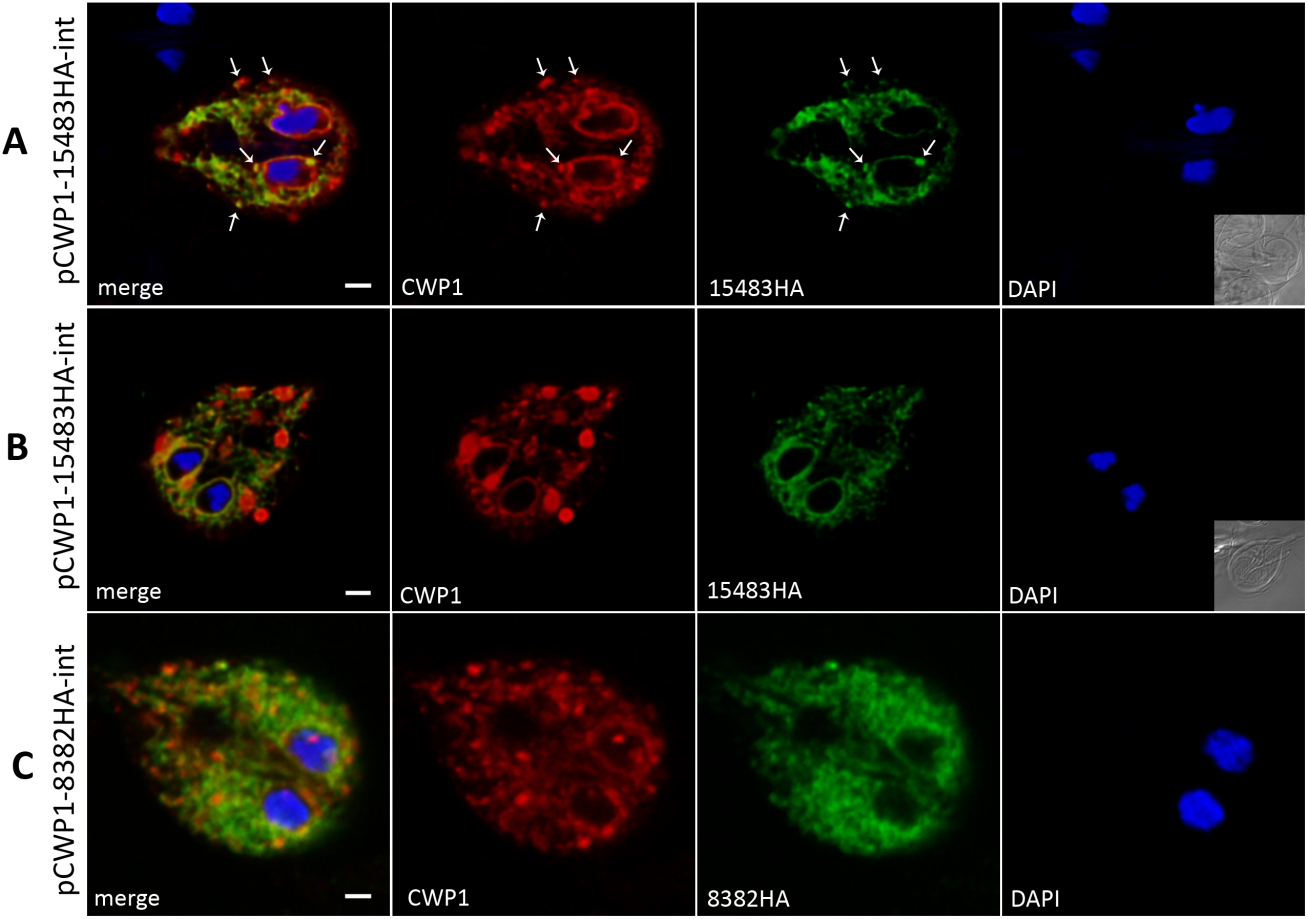
Subcellular localization of the Giardia UDP-GlcNAc transporter (Gl15483) and the putative ER-GALE (Gl8382). Representative subcellular localization of C-terminally HA-tagged variants of UDP-GlcNAc transporter (Gl15483) and the putative UDP-GlcNAc-4′-epimerase (Gl8382) in encysting transgenic cells. **A, B**) At 13h p.i.: the HA-tagged transporter was detected in the ER. Co-localization with CWP1 was observed in the perinuclear ER and in areas corresponding to distinct ER regions or early ESVs (A, arrows). CWP1 is delayed in the perinuclear ER of Gl15483HA-expressing cells where it overlaps with Gl15483HA. In mature ESVs, no co-localization of the two proteins was observed. **C)** Localization of the putative UDP-GlcNAc-4′-epimerase (Gl8382HA) at 13h p.i in the ER together with CWP1 whose export is delayed. *Antibodies:* anti-HA high affinity from rat, Alexa488-conjugated goat anti-rat (green), and Texas red-conjugated anti-CWP1 (red). Nuclear DNA was labeled with DAPI. *pCWP1:* inducible CWP1 promoter; *int:* stable integration into the genome; scale bar: 1.5 µm.

ORF Gl15483 codes for an UDP-N-acetylglucosamine (UDP-GlcNAc) sugar transporter [50] with a reported localization at the perinuclear ER and at peripheral vesicles distinct from PVs [51]. This observation is only partially consistent with our localization of an HA-tagged variant in the ER but not to other organelles in encysting trophozoites at 13h p.i. (Figure 7A, 7B). In dual labeling experiments, signal overlap of 15483-HA and CWP1 in the perinuclear ER and in areas corresponding to punctate peripheral ER regions or emerging ESVs was observed (Figure 7A, arrows). When mature ESVs were present, the overlap of the two proteins was restricted mostly to the perinuclear ER (Figure 7B). Notably, Gl15483-HA-expressing cells showed delay of CWP1 export from the ER and accumulation in the perinuclear ER at 13h p.i. (Figure 7A, 7B).

ORF Gl8382 codes for a putative UDP-GlcNAc-4′-epimerase (GALE) homolog in Giardia with an N-terminal signal sequence. Manual protein sequence analysis revealed that the giardial protein harbors all conserved motifs required for the enzyme’s function [52] (Figure S3). An HA-tagged variant localized to the ER (Figure 7C). These transgenic cells retained CWP1 in the ER at 13h p.i. and no mature ESVs were formed. The correlation, if any, between this phenotype and the predicted function of the protein, i.e. conversion of UDP-GlcNAc into the UDP-N-acetylgalactosamine (UDP-GalNAc) monomer of the cyst wall glycan [53] in the ER lumen, remains to be determined.

#### C) Factors for co-translational protein insertion

**Figure 8:**
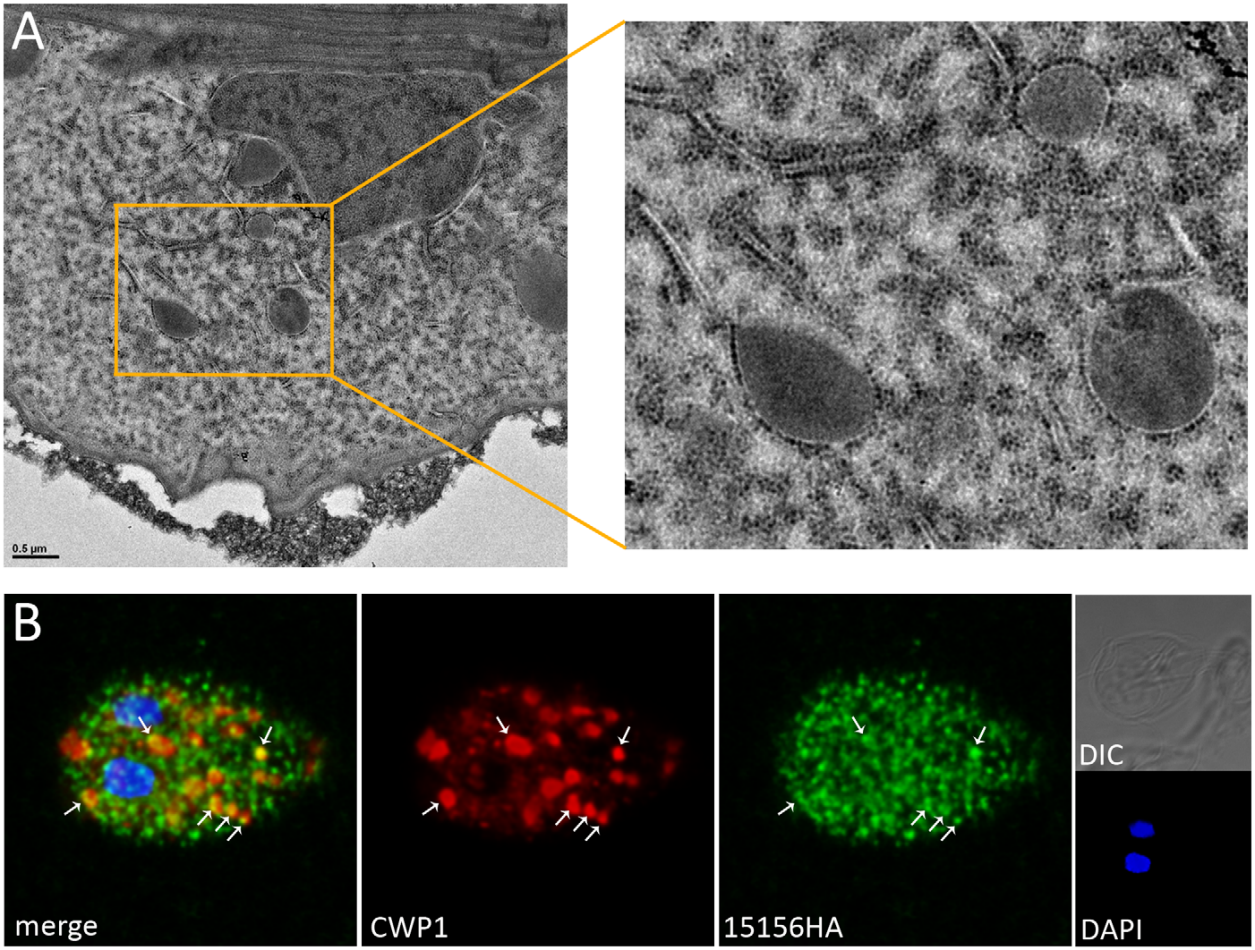
Stage-specific recruitment of ribosomes to ESV membranes. **A**) Transmission electron microscopy image of ESVs in *G. lamblia* wild type encysting cells at 7 h p.i. (magnified on the right). Recruitment of ribosomes to ER membranes (tubular structures) and to early ESVs (round, electron-dense structures) is observed. Ribosomes are visible as small, round and highly electron-dense structures arrayed along the cytoplasmic side of ESV and ER membranes. **B**) Immunofluorescence analysis of cells expressing a C-terminally HA-tagged signal recognition particle component SRP54 (line pendo-15156HA-epi). The micrograph shows punctuate localization of SRP54-HA and partial overlap with CWP1 accumulation (arrows). Cytoplasmic and ER membrane associated SRP54-HA generates a high background signal, making a detection of the protein at ESV membranes difficult. *Antibodies:* anti-HA high affinity from rat, Alexa488-conjugated goat anti-rat (green), and Texas red-conjugated anti-CWP1 (red). Nuclei were labeled with DAPI. *pCWP1:* inducible CWP1 promoter; *epi:* episomal maintenance of expression vector; scale bar: 1.5 µm.

The panel of candidate ESV proteins contains a substantial number (8, 11.1%) of ribosomal or ribosome-associated proteins (Figure 3, Table 1) whereas the PV dataset only contained 2 of these potential contaminants. Interestingly, in transmission electron microscopy (TEM) images we found that, in addition to decorating rough ER membranes, ribosomes distinctly associated with ESV membranes (Figure 8A, inset). Taken together with the identification of a component of the signal recognition particle, SRP54 (Gl15156) in the ESV dataset, this suggests that ESV cargo proteins could be inserted co-translationally directly into the ESV lumen. Since transgenic cells expressing epitope-tagged ribosomal subunits were not viable, we tested this hypothesis indirectly by expression of an epitope-tagged SRP54 variant (Table 1). The tagged product localized in a punctate, distributed pattern and signal overlap with CWP1 was detected predominantly in smaller ESVs (Figure 8B, arrows). The possibility that proteins, presumably CWPs, are co-translationally inserted directly into ESVs is intriguing but requires additional experimental testing.

In summary (see Table 1) our localization studies uncovered 5 ESV-associated proteins including 3 Giardia-specific proteins (Gl32419, Gl14458, Gl25205), the signal recognition particle subunit SRP54 (Gl15156), and an UDP-GlcNAc transporter (Gl15483). Six tagged candidates localized exclusively to the ER. Among these were an UDP-GlcNAc-4′-epimerase (GALE) eventually involved in synthesis of the CW glycan, two hypothetical proteins (Gl10221, Gl22136), a predicted synaptic protein SC2 (Gl88581) and a WD40 repeat protein (Gl15956) both of unknown function, and an amino acid transporter (Gl11299). Four tagged candidates showed cytoplasmic localization: two Giardia-specific hypothetical proteins (Gl7350, Gl9157), a calcium-binding protein (Gl7207), and a glycosyl transferase family 8 protein (Gl11595). Finally, a Giardia-specific protein of unknown function (Gl87926) was detected at the ventral disc. Taken together, this preliminary analysis suggests that i) abundant novel ESV cargo proteins are not present in mature ESVs, and ii) while large amounts of ER-derived contaminants can be eliminated by subtraction, many ER proteins remain in the dataset. The most likely explanation for this is the intimate physical contact and direct connections between the ER and ESVs as illustrated in Figure 8A, which survive cell disruption and organelle preparation.

## Discussion

ESV organelles are inducible Golgi-like membrane compartments for accumulation, processing, sorting, and export of the Giardia cyst wall material during differentiation of trophozoites into cysts [26], [28], [29]. Their *de novo* genesis and maturation to secretion-competent organelles is only partially understood: Fewer than 20 ESV-associated factors (among them the 3 CWPs) have been identified or characterized [10], [16], [26], [29], [32], [35]–[39]. However, no defining ESV-specific, peripherally associated or membrane-bound factor has been identified. Previous attempts to generate an ESV proteome using cell fractionation and density gradient centrifugation yielded datasets which revealed additional ESV factors but also contained very high levels of contaminating proteins [32] (Hehl A.B., unpublished). Here, we tested a conceptually new approach to generate highly enriched organelle proteome datasets for ESVs and PVs resulting in identification of a candidate set of 72 ESV- and 82 PV-associated proteins.

### The ER and ESVs Maintain a Broad Range of Interactions

Partial validation of the “ESV-specific” protein dataset (72 candidates) by subcellular localization studies of 16 selected ESV candidate proteins revealed 5 proteins which localized to ESVs, but also many ER proteins: 11 candidates had a distribution consistent with ER localization; 6 thereof were detected exclusively in the ER. This suggests that elements of ER organelles remain physically linked to and are co-sorted with labeled ESVs, but not PVs.

ESVs are closely associated with the ER during their neogenesis and nucleated from ERES in a COPII-dependent process [26], [27]. Although light microscopy data suggests that ESVs become physically distinct from the ER after neogenesis [27], [28] previously published electron microscopy (EM) data [20], [42], [54] and data in the present study (Figure 8A) clearly show membrane continuities between the organelle systems. The imaging data is supported by evidence for cycling of ER-resident proteins such as Hsp70/BiP between the two organelles [32]. An extensive network of tubular membrane connections mediating exchange of CWP1 between maturing ESVs makes a definition of the boundaries for ESVs even more challenging [26]: there is a possibility that this dynamic network is not restricted to ESV organelles but establishes direct connections with the ER [28]. Further, the recruitment of proteasomes [32] and ribosomes to ER and ESV membranes, as shown also in this study, is an additional common feature of the two organelles. This suggests that basic trafficking-related processes such as co-translational import of secreted proteins, folding, retro-translocation, and associated degradation processes start at the ER level but may extend beyond ESV genesis.

Consistent with the premise of subtractive elimination of ER proteins from organelle-specific factors, the MS data intersect was highly enriched in abundant ER-resident proteins, e.g. protein disulfide isomerase (PDI) 1–5, Hsp70/BiP and Hsp90/Grp94. On the other hand, many proteins in the ESV dataset showed a typical ER distribution, although 5 proteins also localized to ESVs. A likely explanation is that many tested candidates are present in ER subdomains closely associated or directly connected to ESVs. When encysting Giardia cells are subjected to cell disruption by sonication or other tested methods such as nitrogen cavitation (not shown), these ER domains remain physically linked to ESVs and are enriched accordingly in the sorting process.

Despite elimination of ∼90% of all hits in the dataset intersection, which was enriched for known ER proteins, the ESV dataset still contained a large number of proteins localizing to the ER. There are several possible explanations for this surprising finding, the most likely being the intimate association between the ER and ESVs as discussed above. The physical connections and membrane continuities may be resistant to our cell disruption protocols and may thus limit to extraction of ESVs from this subcellular context. In contrast, ER membranes and PV do not occupy the same cellular space: the ER network extends throughout the cytoplasm but not into the cortical layer just below the plasma membrane into which PVs are embedded [55]. Although the interface between the two organelle systems includes postulated direct contact points [8] the subcellular segregation of the two compartments appears to make separation by cell disruption easier.

We performed a preliminary validation of the “ESV-specific” dataset by localizing 16 tagged factors. The results suggested that the dataset contained many ER proteins rather than novel ESV factors. Based on this we propose two non-mutually exclusive interpretations for the lack of novel ESV-specific proteins in our datasets: i) The organelle enrichment strategy and post-sorting elimination of contaminants worked well for filtering out abundant generic ER proteins, but is less efficient in eliminating minor contaminants and/or proteins that remain attached to the fluorescently labeled organelles. ii) ESVs and the ER are highly distinct with respect to abundant luminal proteins such as CWPs, modifying factors, or chaperones. However, both organelles share most membrane or peripherally associated proteins. All available data on stage-specific gene expression during encystation suggests that only CWP genes are strictly stage-specifically regulated (i.e. completely “off” in trophozoites) whilst the remaining (<20) significantly modulated “encystation” genes are upregulated from a basal level in trophozoites during the first 7 hours p.i. [48]. Since CWPs as cargo proteins are certainly more abundant than ESV membrane or organelle-associated proteins, any novel representatives of the latter are difficult to identify within a still relatively high background of ER proteins. In fact, the apparent lack of abundant novel proteins in ESVs exacerbates the appearance of false-positive ER factors in the organelle-specific dataset.

### A Proposal for Cargo-driven ESV Neogenesis

The emerging picture in Giardia encystation is that, aside from synthesis of the bulk cyst wall proteins, only relatively small adjustments of expression in some genes (e.g. enzymes for the synthesis of the cyst wall glycan) are required for encysting cells to produce mature ESVs [48],[56]. This also suggests that morphogenesis of ESV organelles could be driven by accumulation of cargo rather than by specific organelle-associated factors, analogous to the formation of dense core secretory granules (DCSG) in endocrine/neuroendocrine cells [57], or in ciliates [58]. In early electron microscopy studies, ESV formation was described as aggregation of electron-dense material in ER membrane-bounded compartments, followed by growth via direct addition of newly synthesized CWPs until large organelles are formed [21], [54], [59]. A more recent model posits that ESV formation is the result of self-organizing properties, mainly of CWP3, leading to formation of a dense core [28]. The ability of CWP1 and 2 to form highly cross-linked complexes [21], [41], and of CWP1 to bind directly to the GalNAc homopolymers [60], which constitutes 60% of the cyst wall material, provides the prerequisites for distributing the extracellular matrix material evenly on the surface of the parasite before initiating polymerization. The presence of basic and simple machinery for ESV formation is supported by the observation that the expression of CWP1 and CWP2 in human embryonic kidney cells is sufficient to induce accumulation in membrane compartments and secretion of the proteins [61]. This observation is in line with granule formation in non-granule forming cells upon expression of different dense core granule cargo proteins, including pro-vasopressin, chromogranin A or von Willebrand factor [62]–[64], suggesting that the cargo proteins themselves induce the formation of their own carriers through accumulation. Thus, ESV formation might be driven by progressive accumulation of CWPs by a “sorting by retention” mechanism, while ER-resident proteins such as Hsp70/BiP are removed via low-density vesicles or tubular connections between ER and ESVs as the organelles mature [32]. Taken together, the proteome data support a scenario for ESV formation and maturation which relies strongly on inherent properties of cargo proteins and likely only few and as yet unidentified additional components, rather than on a dedicated ESV-specific, organelle-associated machinery driving morphogenesis.

Analysis of the remaining candidates in the data set might bring to light further proteins localizing to ESV organelles. However, none of these genes appear to be significantly upregulated during encystation [48]. Thus, unless significant translational control comes into play [36] we do not expect any additional highly abundant proteins to be discovered exclusively in ESVs.

Taken together, identification of one or more low abundance protein(s) which could be used as defining factors for ESVs as post ER organelles within the only regulated secretory transport pathway in Giardia, remains a significant challenge.

### Ribosomal Proteins Are Enriched in the ESV Fraction

A comparison of the 72 ESV-specific and 82 PV-specific candidates revealed a significant enrichment of translation/ribosome proteins in the former. Using transmission electron and immunofluorescence microscopy we found support for recruitment of ribosomes not only to the ER but also to ESV membranes during the differentiation process. However, additional functional verification is required, in particular to test whether co-translational insertion of proteins directed to the regulated secretory pathway may occur directly into ESVs.

In eukaryotic cells, ribosomes localize to the cytoplasm, the nuclear envelope, and the rough ER, giving the latter its typical appearance in transmission electron microscopy. While an association of ribosomes to Golgi membranes in eukaryotic cells containing a steady state Golgi organelle was not observed, giardial ribosomes can be visualized on ESV membranes. Co-translational insertion of secreted proteins directly across ESV membranes might be a consequence of the requirement for producing large quantities of CWP in the relatively short time when ESVs grow maximally. The process of CWP synthesis, translocation and folding clearly begins at the ER level from which CWPs are exported in a COPII-dependent manner [27]). However, the amount of CWPs detected in the ER drops significantly after establishment of small immature ESVs (Figure 5 in [27]). Direct co-translational import of CWPs via ESV membranes is one possible explanation for this observation. Directed translocation of proteins across ESV membranes towards the cytosol as part of a quality control system was inferred from the observation that proteasome complexes were recruited to the vicinity of developing ESV organelles [26]. Pore complexes such as Sec61, for which an alpha and a gamma subunit are annotated in the Giardia genome database, are required for co-translational insertion and are also strongly implicated in retro-translocation to the cytoplasm [65]. In support of co-translational insertion of proteins across ESV membranes, a signal recognition particle component, 54 kDa protein (Gl15156), has been localized partially to ESVs (Figure 8B). However, none of these factors are distributed in an organelle-specific manner and there is currently no possibility to design experiments allowing a dissection of the directionality of protein translocation across ESV or ER membranes.

### Is the Cyst Wall Sugar Monomer UDP-GalNAc Synthesized in the ER?

The Giardia cyst wall consists of 3 proteins (CWP1–3) and a β(1-3)-GalNAc homopolymer which makes up about 60% of the cyst wall [24], [25]. While the protein components are trafficked via ESV organelles to the surface of the cell, the place of synthesis, transport to the surface, as well as timing and manner of incorporation of the sugar components into the cyst wall remains largely unknown. The sparse literature on the subject suggests synthesis of the cyst wall monomer UDP-GalNAc from endogenous glucose by a series of stage-specifically regulated, enzymatic reactions [53]. A late step, i.e. conversion of UDP-GlcNAc into UDP-GalNAc, was proposed to be performed by a cytosolic UDP-GlcNAc-4′-epimerase or so called GALE (Gl7982). While some experimental data showed that the enzyme converted UDP-GlcNAc into UDP-GalNAc during encystation, investigation of enzyme kinetics showed that the reverse reaction towards production of UDP-GlcNAc was clearly favored, raising significant doubts about a productive synthesis of UDP-GalNAc in the cytoplasm [66].

In this study, two important proteins potentially involved in this process were identified in the ESV dataset: i) the only nucleotide sugar transporter (Gl15483) identified in the Giardia genome project [50] which specifically transports UDP-GlcNAc from the cytoplasm to the ER lumen [51], and ii) a putative UDP-GlcNAc-4′-epimerase (GALE) (Gl8382). We detected epitope-tagged variants of the epimerase in the ER, and the transporter mainly showed distribution in the perinuclear ER and early ESVs, where its signal overlapped with that of CWP1. N-glycosylation of Giardia proteins is restricted to addition of GlcNAc_1-2_ to asparagine [67]. Consistent with this, the parasite lacks genes required for synthesis of the typical eukaryotic core-oligosaccharide GlcNAc_2_Man_9_Glc_3_ and for further N-glycan processing in the ER and Golgi. While the UDP-GlcNAc-transporter Gl15483 in the ER membrane imports UDP-GlcNAc used for N-glycosylation, the presence of an ER-localized UDP-GlcNAc-4′-epimerase converting UDP-GlcNAc into UDP-GalNAc indicates involvement of the putative GALE enzyme (Gl8382) in producing the UDP-GalNAc monomer for the cyst wall glycan in the ER.

### Opportunities and Technical Limitations of Dual Organelle Sorting and in Silico Processing of Mass Spectrometry Data

In proteomic studies, the purity of the biological sample is of utmost importance for a successful analysis. One of the most crucial steps is subcellular fractionation. Despite considerable efforts to optimize protocols for purification of Giardia organelles, the levels of contaminating proteins from non-target organelles and cellular structures remain high [32], [33]. The most frequently used subcellular fractionation techniques applied in organellar proteomics are density-based gradient centrifugation, affinity-based isolation, free flow electrophoresis, and recently also flow cytometry [68]–[71]. Fluorescence-based organelle sorting by flow cytometry is challenging because of the small size of organelles which usually results in reduced fluorescence intensity. In the case of Giardia organelles, labeling with a highly expressed luminal GFP-tagged organelle marker (CWP3-GFP) in ESVs or the endocytic uptake of a fluid-phase fluorescent dye by PV organelles created unique opportunities for intense organelle labeling. This was sufficient to clearly detect and enrich the organelles simultaneously by flow cytometry despite their small size and, most importantly, to achieve a 100% relative enrichment (i.e. 100% separation) of ESVs and PVs which was a precondition for the subsequent subtractive approach. Analysis of ESV and PV-enriched samples by shotgun mass spectrometry revealed high reproducibility between 3 independent experiments.

The limited purity of organelle preparations and the high sensitivity of current mass spectrometers require additional measures to address the large quantities of false-positive hits. Researchers have developed different (*in silico*) strategies for the elimination of contaminating proteins from organellar datasets. A proteomic study on Giardia mitochondria-relic organelles (mitosomes) using gradient centrifugation took advantage of the organelle distribution into two neighboring fractions [33]. Using isobaric tags for relative and absolute quantitation (iTRAQ) mass spectrometry the relative distribution of mitosomal marker proteins between the two fractions was evaluated, and novel putative proteins were identified based on their similar distribution ratio [33]. Another study focusing on the proteome of Spironucleus hydrogenosomes utilized the distribution of the target organelle into two gradient fractions of distinct densities [72]. After mass spectrometry analysis, putative organelle-specific proteins were identified by their co-purification with organelle marker proteins. Both approaches significantly reduced the incidence of contaminants in the resulting large datasets, but the proportion of organelle-specific proteins remained low.

In our case, simultaneous enrichment of ESV and PV organelles makes subtractive approaches to identify contaminating MS hits *in silico* possible. Using this approach we removed 1059 hits which were common to both organelle fractions, including a large proportion (86%) of ribosome/translation and ER-derived contaminants which constitute a major challenge in organelle proteomic studies. Accordingly, among the 72 ESV and 82 PV candidates only a single predicted ER protein was identified in each dataset. However, preliminary evaluation revealed a large proportion of hitherto unknown ER proteins.

Two factors may contribute to the low discovery rate of organelle proteins: i) peripherally associated organelle proteins may be partially or completely lost during the purification and sorting process, ii) the subtractive approach to remove contaminating proteins likely also eliminates specific categories of organelle-associated proteins, i.e. those with secondary localizations or with large cytoplasmic pools. Examples are the small GTPases Rab1, Rab11, Arf1 and COPI components which also localize to the ER and other membranes. In addition, some peripherally associated factors are recruited to ESVs only during specific phases of the differentiation process, e.g. Rab1, COPI [26], [29], or members of the SNARE family [43].

In summary, the success of a simultaneous sorting approach strongly depends on how cleanly the differentially labeled organelles can be prepared during cell disruption and how similar their cellular context is. The major limitation in our case is the close association of ESVs but not PVs with the ER subdomains. Since the cellular context of the two analyzed organelles differs greatly, subtractive analysis appears to be more efficient for PV candidates but still led to many false-positive candidates in the ESV dataset. Thus, subtractive analysis of datasets derived from simultaneously sorted organelles is a useful strategy to discover organelle-specific factors, but the degree of success depends strongly on the feasibility of clean extraction of target organelles from their subcellular context.

## Materials and Methods

### Giardia Cell Culture and in Vitro Encystation

*Giardia lamblia* WBC6 (ATCC catalog number 50803) trophozoites were grown under microaerophilic conditions in 11 ml culture tubes (Nunc, cat. 156758) or triple flasks (Nunc, cat. 132867) containing TYI-S- 33 medium supplemented with 10% fetal bovine serum and bovine bile according to standard protocols [41]. Parasites were harvested by chilling the tubes on ice for 30 minutes (for flasks: 1 hour in ice water) to detach adherent cells and collected by centrifugation (900×*g*, 10 minutes, 4°C). Encystation was induced using the two-step method as described previously [48] by cultivating the trophozoites in bile-free medium for 44 hours and thereafter in medium (pH 7.85) containing porcine bile.

### Sample Preparation for Flow Cytometry

#### ESV-organelle staining

CWP3-GFP expressing cells [28] and WB wild type cells (control) were grown in triple flasks (2×800 ml) and kept in encystation conditions for 13 hours as described above. Cells were harvested and resuspended in 20 ml of encystation medium. To allow oxidative chromophore formation without damage to the cells, the cell suspension was chilled on ice and dispersed into a 6 well plate (Sigma, cat. Z707759) and exposed to air on ice over night. To complete GFP folding, the ice-cold cell suspension was collected from the plate and incubated in microaerophilic conditions for 30 minutes at 37°C.

#### PV-organelle labeling

Wild type trophozoites were grown in triple flasks and harvested as described above. Cells were washed twice in 10 ml 1x PBS (900×*g*, 10 minutes, 4°C) and resuspended in 500 ul supplemented PBS (5 mM glucose, 5 mM cysteine, 0.1 mM ascorbic acid) containing 4 mg/ml dextran AlexaFluor-647 (Molecular Probes Inc., cat. D22914). Endocytic uptake of the fluorescent dye by PV organelles was achieved at 37°C for 30 minutes, protected from light.

All samples were washed twice in 10 ml 1x PBS (900×*g*, 10 minutes, 4°C) and resuspended in 5 ml 1x PBS. After addition of protease inhibitor cocktail (Calbiochem, cat. 539131) and phenylmethanesulfonyl fluoride (PMSF, Sigma, cat. P7626), the cells were disrupted by four rounds of mild sonication (Branson Sonifier 250, Branson Ultrasonics Corporation, 4×60 pulses, duty cycle 20%, output control 1.5) on ice. To remove remaining intact cells and cysts, the cell suspensions were passed through a 5 um filter (MILLEX-SV 5.00 um, Millipore, cat. SLSV25LS). Prior to the sort, the two cell lysates were mixed at a ratio of 1∶4 to obtain similar numbers of target events, i.e. GFP-positive and AF647-positive events, in the mixture.

### Flow Cytometry-based Organelle Sorting

Flow cytometry-based sorting of organelles was performed on a BD FACSAriaIII™ cell sorter. For data acquisition and processing, the BD FACSDiva™ software (version 6.1.3) was used. In order to achieve maximal speed the sort was performed with using a nozzle with a 70 micrometer orifice diameter at 4.83 bar sheath pressure. GFP was excited by a 488 nm laser and emission was detected using a 525/45 band pass filter. AlexaFluor647 was excited by a 633 nm laser and emission was detected using a 670/40 band pass filter. Given the small organelle size, considerable proportion of observed events was stemming from particulate and electronic noise. All the fluorescent and light scatter parameters were estimated by the height of the voltage pulse generated by each event. The detection threshold was defined as a logical combination of green (GFP) and red (AF647) signal value using the “OR” functional operator. The organelle populations were defined by a parent gate (P3) based on FSC-H (forward scatter-height) and SSC-H (side scatter-height) (Figure 1). Yellow fluorescent protein (YFP) control beads (SHERO™ Fluorescent Nanospheres, Spherotech Inc., cat. FP-0552–2) in the size range of the organelles (400 to 600 nm) were used to estimate the level of particulate and/or electronic noise with selected instrument settings. To select for GFP-positive and AF647-positive events out of the mixed organelle population, gates P1 and P2 were set in a bivariate dot-plot. An unlabeled cell suspension was used as negative control to define the gate positions. To attain maximal purity, a sort precision mode of 0/32/0 was chosen. The sort was performed with an average event rate of 25′000 to 30′000 per second, an average sort rate of 600 events per second and a mean sort efficiency of 80%. Twenty million GFP-positive and AF647-positive events were collected in separate 5 ml polystyrene tubes (BD Biosciences, cat. 352052). For quality control of the sort, the collected material was analyzed by flow cytometry using the same settings as for the sort (Figure 1).

### Protein Precipitation

Protein precipitation was performed using the pyrogallol red molybdate (PRM) method [73]. Briefly, PRM reagent was added to the sample in a ratio of 1∶4. The samples were mixed and incubated at 25°C for 25 minutes. Proteins were pelleted at 3800×*g* for 30 minutes and dissolved in 1 ml of ddH2O. After addition of 250 ul of PRM reagent, incubation and pelleting was repeated one more time. The final pellet was resuspended in 25 ul of Laemmli buffer containing 0.5% (v/v) β-mercaptoethanol, incubated for 5 minutes in boiling water, and stored over night at −20°C.

### SDS-PAGE and Immunoblot Analysis

SDS-PAGE on a 12% polyacrylamide gel and subsequent transfer to a nitrocellulose membrane (Protran, Whatman GmbH, cat. 10401396) was performed according to standard protocols. The following antibodies were used: Anti-GFP from mouse (JL-8, Clontech, cat. 632380; 1∶2000) and a horseradish peroxidase-conjugated rabbit anti-mouse IgG (Sigma, cat. A9044, 1∶8000). Signal detection was performed using Western Lightning Chemiluminescence Reagent (PerkinElmer Life Sciences, cat. NEL100001EA). Data collection was done in a Multimage Light Cabinet with AlphaEase FC software (Alpha Innotech, San Leonardo, CA, USA).

### Mass Spectrometry Analysis and Protein Identification

Protein samples were boiled for 5 minutes and centrifuged for 1 minute at 16′100×*g* at room temperature to pellet insoluble material. The samples were separated by 1D–SDS-PAGE using precast 12% Tris-Glycine gels (Invitrogen, cat. IM6000). 4 to 8 ul of supernatant were loaded, depending on the estimated amount of protein in the respective sample determined in a preceding test run. After staining with Roti Blue (CARL ROTH, cat. A152), each gel line was cut into 21 slices. In-gel digestion of proteins using trypsin and extraction of peptides was performed according to standard protocols. Samples were analyzed on a LTQ-Orbitrap XL mass spectrometer (Thermo Fischer Scientific, Bremen, Germany) coupled to an Eksigent-Nano-HPLC system (Eksigent Technologies, Dublin, CA, USA). A detailed description of sample preparation and mass spectrometry analysis can be found in the Text S1.

The raw-files from the mass spectrometer were converted into Mascot generic files (mgf) with Mascot Distiller software 2.4.2.0 (Matrix Science Ltd., London, UK). The peak lists were searched using Mascot Server 2.3 against the *G. lamblia* database (http://tinyurl.com/37z5zqp) with a concatenated decoy database supplemented with contaminants, The Arabidopsis Information Resource (TAIR9) protein database and the Swissprot database to increase the database’s size. The final database included 79141 entries. The identification results were loaded into Scaffold 3.0 (Proteome Software, Portland, US) and filtered for a minimal mascot score of 20 for peptide probability, a protein probability greater than 80%, and a minimum of 2 unique peptides per protein. The mass spectrometry proteomics data have been deposited to the ProteomeXchange Consortium (http://www.proteomexchange.org) via the PRIDE partner repository [74] with the dataset identifier PXD000694”.

### In silico Removal and Functional Annotation Clustering of the Intersection Dataset

The final ESV and PV organelle-specific datasets were compiled by *in silico* identification and removal of the contaminating proteins. Briefly, the ESV-derived and PV-derived mass spectrometry (MS)-datasets from the three independent experiments were intersected separately. Proteins detected in both ESV and PV MS-datasets were considered as “contaminants” and removed, thus generating three independent subtractive lists enriched for putative ESV-specific hits and PV-specific hits, respectively. To enhance the stringency for detection of putative organelle-specific candidates, we accepted only ESV candidates that were detected in at least two more ESV MS-datasets than PV MS-datasets, and vice-versa. A detailed description of the procedure is attached Figure S3. The contaminating proteins defined by the data intersection were evaluated and clustered into functional groups using the DAVID bioinformatics tool (http://david.abcc.ncifcrf.gov/home.jsp) [34]. Since analysis in DAVID is restricted to 3000 genes, clustering of the Giardia genome as a control was performed by the generation of 10 independent lists each containing 3000 randomly selected Giardia genes.

### In silico Analysis Tools

Analysis of primary structure and domain architecture of ESV and PV candidates (i.e., manual annotation) was performed using the following tools and databases: PSORTII (http://psort.hgc.jp/form2.html) for prediction of subcellular localization, TMHMM (http://www.cbs.dtu.dk/services/TMHMM/) for prediction of transmembrane helices, SMART (http://smart.embl-heidelberg.de/) for prediction of patterns and functional domains, pBLAST for protein homology detection (protein blast by NCBI, http://blast.ncbi.nlm.nih.gov/Blast.cgi), HHPred (http://toolkit.tuebingen.mpg.de/hhpred) for protein homology detection based on Hidden Markov Model (HMM-HMM) comparison, and the Giardia genome database (http://giardiadb.org/giardiadb/) for changes in mRNA expression during the Giardia life cycle. For functional domains predicted by SMART we used an e-value of 10e-5 as cutoff, and for protein homologies predicted by pBLAST we accepted alignment scores above 80. Alignment scores between 50 and 80 were accepted only when the pBLAST predictions were consistent with those of HHPred. The latter was used to make pBLAST more robust; only hits with a probability above 95% were accepted.

Functional annotation clustering of the data intersect was performed using the DAVID bioinformatics tool (http://david.abcc.ncifcrf.gov/home.jsp) [34].

### Expression Constructs and Transfection

For cloning of C-terminally HA-tagged proteins in Giardia, a vector PAC-CHA with additional restriction sites was designed on the basis of the previously described vector pPacV-Integ [26]. Additional restriction sites were inserted via oligonucleotide primers. A detailed vector map can be found in the Figure S4. For each gene of interest two expression vectors were constructed, one in which expression of the gene of interest is driven by its own promoter (pendo), and another in which the gene of interest is under the control of the inducible cyst wall protein 1 promoter (pCWP1). GenBank accession numbers and a list of primers used for cloning can be found in Tables S2 and S3, respectively. For transfection, 15 ug of plasmid DNA linearized with *Swa*I was electroporated (BIO RAD Gene Pulser, 350V, 960 mF, 800 Ohm). The expression vector is targeted to the *Giardia lamblia* triose phosphate isomerase (Gl-TPI) locus by homologous recombination [75] stable transfectants are selected with the antibiotic puromycin (Sigma, cat. 7699111) at a concentration of 77 uM for 5 days. For episomal maintenance, circular plasmid DNA was electroporated and selected with puromycin.

### Immune Fluorescence Assay

Immunofluorescence analysis was performed as described previously [20]. The following antibodies were used in this work: Anti-HA high affinity from rat (Roche diagnostics AG, cat. 11867423001, 1∶50), Alexa488-conjugated goat anti-rat (Invitrogen, cat. A11006, dilution 1∶200), Texas Red-conjugated anti CWP1 (Waterborne™ Inc., cat. A300TR-R, dilution 1∶50). For microscopy cells were embedded in Vectashield (Vector Labs, Inc., cat. H-1200) containing the DNA intercalating agent 4′-6-Diamidino-2-phenylindole (DAPI) for staining of nuclear DNA. Immunofluorescence analysis was performed on the standard fluorescence microscopes Leica DM IRBE with MetaVue software version 5.0r1, or Nikon Eclipse 80i with Openlab Improvision software 5.5.2 for data collection. WCIF ImageJ was used for image processing. Alternatively, analysis was performed on a Leica SP2 AOBS confocal laser-scanning microscope (Leica Microsystems, Wetzlar, Germany) equipped with a glycerol objective (Leica, HCX PL APO CS 63×1.3 Corr).

### Transmission Electron Microscopy

Transmission electron microscopy and sample preparation was performed as described previously [29].

## Supporting Information

Figure S1 **Workflow.** CWP3-GFP expressing cells at 13 hours p.i. **(A)** and wild type trophozoites after endocytic uptake of the fluid phase dye Dextran-AlexaFluor-647 **(B)** were disrupted by sonication and passed through a 5µm filter. The cleared microsome fractions were mixed **(C)** and organelles were simultaneously enriched by flow cytometry-assisted organelle sorting (FAOS) **(D)**. Sample preparation and organelle sorting were performed in biological triplicates. Protein precipitates of organelle-enriched fractions were separated by 1D–SDS-PAGE and analyzed by mass spectrometry (MS) **(E)**, resulting in 3 ESV and PV mass spectrometry datasets, each **(F)**. Contaminating proteins were identified by intersecting the ESV and PV MS-datasets **(G)**. A detailed description of the intersection can be found in Figure S2. *In silico* data filtration, i.e. removal of the data intersection **(H)** revealed ESV-organelle **(J)** and PV-organelle **(K)** specific datasets.
(TIF)

Figure S2 **Generation of the MS data intersection. A)** Mass spectrometry datasets of ESV-enriched (E1, E2, E3) and PV-enriched (P1, P2, P3) fractions of each replicate were intersected separately (top). The numbers stand for the proteins detected by mass spectrometry. Removal of the intersection revealed proteins exclusively detected in ESV-enriched fractions (middle, left) or PV-enriched fractions (middle, right). From these lists, only proteins occurring in at least two lists were accepted (bottom, blue). The proteins were further analyzed according to their distribution pattern in the six organelle-enriched fractions (B). **B)** Schematic representation of the protein distribution pattern in ESV- and PV-enriched fractions. ESV candidates (left): proteins of type X were detected exclusively and in at least two of three ESV fractions, proteins of type Y were detected in all ESV fractions and in one PV fraction, proteins of type Z were detected in only two ESV fractions and in one PV fraction. The same is true vice-versa for PV candidates (right). Type Z proteins were removed, resulting in 72 ESV and 82 PV candidate proteins. The respective protein numbers are indicated in brackets.
(TIF)

Figure S3 **Conserved short chain dehydrogenases (SDH) motifs in Gl8382, Gl7982 and human GALE.** Protein sequences of *G. lamblia* Gl7982 (cytoplasmic GALE, [53]), *G. lamblia* Gl8382 (putative ER-GALE), and the human GALE (hGALE) were analyzed manually. All conserved sequences required for hGALE function [52] are present in both Giardia GALEs and listed in the table. A conserved PG motif, which is required for the direction of the reaction, is only present in the Giardia ER-GALE. An N-terminal integral membrane domain in the ER-GALE shifts the conserved motif positions for about 40 amino acids towards the C-terminus, compared to the cytoplasmic GALE and hGALE.
(TIF)

Figure S4 **Vector map.** Schematic depiction of the vector used for candidate cloning. *pCW1:* putative promoter region of cyst wall protein 1 (GL50803_5638); *ORF:* open reading frame; *HA:* hemagglutinin tag; *CWP 3′UTR:* 3′ untranslated region of cyst wall protein 1 (GL50803_5638); *RS1/2:* recombination sites 1 (GL50803_17200) and 2 (GL50803_93938); *GDH 5′/3′ UTR:* 5′ and 3′ untranslated regions of glutamate dehydrogenase (GL50803_21942); *Puro Res.:* puromycin N-acetyltransferase.
(TIF)

Figure S5 **Gl96994HA-expressing cells at 7h post induction of encystation.** Recombinant protein expression was detected by fluorescence microscopy with an FITC-coupled anti-HA antibody (green, A and B, middle panels). **A)** Surface proteins labeled with biotin were detected by fluorescence microscopy in fixed cells after incubation with Streptavidin-Texas Red (red, left panel). **B)** Visualization of fluid-phase endocytosis of a Dextran-Texas Red marker (red, left panel). Nuclear DNA was labeled with DAPI (blue). *pCWP1:* inducible CWP1 promoter; *TXR*: Texas Red; *FITC*: Fluorescein isothiocyanate. Scale bar: 2 µm.
(TIF)

Table S1 **Protein identification by mass spectrometry.** Raw data exported from proteome software (Scaffold version 3.0). Identification probabilities, quantification values, and number of unique peptides (worksheets 1–3) are indicated for all detected proteins. For each of the 1281 proteins identified, the following information is provided: Product description (column B) and GeneID (column C) according to the *G. lamblia* genome database (GiardiaDB), molecular weight (column D), and the T-test scores (column E). For ESV-enriched fractions (E1-E3, columns F-H) and PV-enriched fractions (P1-P3, columns I-K) the protein probabilities (first worksheet), the quantitative values (second worksheet) and the number of unique peptides (third worksheet) are indicated.
(XLS)

Table S2 **ESV and PV candidate list with additional information.** For each of the 72 ESV and 82 PV candidates, the following information is provided: protein category (column B), GeneID (column C) and product description (column E) according to the *G. lamblia* genome database, NCBI reference number (column D), manual re-annotation (column F) and the prediction tools it is based on (column G), number of transmembrane domains (column H), signal peptide (column I), significant stage-specific up-regulation of transcription (column J), localization of HA-tagged variants determined in this study (column K), and literature information (column L). *TMD:* Transmembrane domains; *SP:* Signal peptide; *ER:* Endoplasmic Reticulum; *ESV:* Encystation specific vesicles; *PV:* Peripheral vesicles; *CYT:* Cytosol; *PM:* Plasma membrane; *asterisk:* prediction tools return different results.
(XLSX)

Table S3 **Oligonucleotide primer sequences.** Primers used for cloning of expression constructs. Sequences are in 5′ to 3′ orientation, restriction sites are marked in bold. *pCWP1:* inducible promoter of *G. lamblia* cyst wall protein 1; *pendo:* endogenous promoter, *HA:* hemagglutinin tag.
(DOC)

Text S1 **Detailed description of mass spectrometry analysis.** Detailed description of SDS-PAGE, sample preparation, mass spectrometry analysis, database search and protein identification.
(DOC)

Text S2 **DAVID functional annotation clustering of data intersection.** Functional annotation clustering of the data intersection using the DAVID bioinformatics tool (http://david.abcc.ncifcrf.gov/home.jsp). For each cluster, the enrichment score is given at the top, and functional groups (left) and protein counts (right) within the respective cluster are listed. The 1059 putative contaminants of the data intersection are categorized into 56 annotation clusters.
(PDF)

Text S3 **DAVID functional annotation clustering of the** ***G. lamblia*** **genome.** Functional annotation clustering of the *G. lamblia* genome using the DAVID bioinformatics tool (http://david.abcc.ncifcrf.gov/home.jsp). For each cluster, the enrichment score is given at the top, and functional groups (left) and protein counts (right) within the respective cluster are listed. Clustering of all predicted proteins in the *G. lamblia* genome (5150 validated genes) using 10 random gene lists containing 3′000 genes each.
(PDF)

## References

[1] Adam RD (2001) Biology of Giardia lamblia. Clinical microbiology reviews 14: 447–475. 11432808 10.1128/CMR.14.3.447-475.2001 PMC88984

[2] Prucca CG, Slavin I, Quiroga R, Elias EV, Rivero FD, et al (2008) Antigenic variation in Giardia lamblia is regulated by RNA interference. Nature 456: 750–754. 19079052 10.1038/nature07585

[3] Nash TE (1989) Antigenic variation in Giardia lamblia. Exp Parasitol 68: 238–241. 2647510 10.1016/0014-4894(89)90104-5

[4] Soltys BJ, Falah M, Gupta RS (1996) Identification of endoplasmic reticulum in the primitive eukaryote Giardia lamblia using cryoelectron microscopy and antibody to Bip. J Cell Sci 109 (Pt 7): 1909–1917. 10.1242/jcs.109.7.1909 8832413

[5] Tovar J, Leon-Avila G, Sanchez LB, Sutak R, Tachezy J, et al (2003) Mitochondrial remnant organelles of Giardia function in iron-sulphur protein maturation. Nature 426: 172–176. 14614504 10.1038/nature01945

[6] Regoes A, Hehl AB (2005) SNAP-tag mediated live cell labeling as an alternative to GFP in anaerobic organisms. Biotechniques 39: 809–810, 812. 10.2144/000112054 16382896

[7] Feely DE, Dyer JK (1987) Localization of acid phosphatase activity in Giardia lamblia and Giardia muris trophozoites. J Protozool 34: 80–83. 3572844 10.1111/j.1550-7408.1987.tb03137.x

[8] Abodeely M, DuBois KN, Hehl A, Stefanic S, Sajid M, et al (2009) A contiguous compartment functions as endoplasmic reticulum and endosome/lysosome in Giardia lamblia. Eukaryot Cell 8: 1665–1676. 19749174 10.1128/EC.00123-09 PMC2772394

[9] Lindmark DG (1988) Giardia lamblia: localization of hydrolase activities in lysosome-like organelles of trophozoites. Exp Parasitol 65: 141–147. 3276550 10.1016/0014-4894(88)90116-6

[10] Touz MC, Nores MJ, Slavin I, Carmona C, Conrad JT, et al (2002) The activity of a developmentally regulated cysteine proteinase is required for cyst wall formation in the primitive eukaryote Giardia lamblia. The Journal of biological chemistry 277: 8474–8481. 11773053 10.1074/jbc.M110250200

[11] Ward W, Alvarado L, Rawlings ND, Engel JC, Franklin C, et al (1997) A primitive enzyme for a primitive cell: the protease required for excystation of Giardia. Cell 89: 437–444. 9150143 10.1016/s0092-8674(00)80224-x

[12] Lanfredi-Rangel A, Attias M, de Carvalho TM, Kattenbach WM, De Souza W (1998) The peripheral vesicles of trophozoites of the primitive protozoan Giardia lamblia may correspond to early and late endosomes and to lysosomes. J Struct Biol 123: 225–235. 9878577 10.1006/jsbi.1998.4035

[13] Touz MC, Lujan HD, Hayes SF, Nash TE (2003) Sorting of encystation-specific cysteine protease to lysosome-like peripheral vacuoles in Giardia lamblia requires a conserved tyrosine-based motif. The Journal of biological chemistry 278: 6420–6426. 12466276 10.1074/jbc.M208354200

[14] Bockman DE, Winborn WB (1968) Electron microscopic localization of exogenous ferritin within vacuoles of Giardia muris. J Protozool 15: 26–30. 5643476 10.1111/j.1550-7408.1968.tb02085.x

[15] Touz MC, Rivero MR, Miras SL, Bonifacino JS (2012) Lysosomal protein trafficking in Giardia lamblia: common and distinct features. Front Biosci (Elite Ed) 4: 1898–1909. 22202006 10.2741/511 PMC3257179

[16] Gaechter V, Schraner E, Wild P, Hehl AB (2008) The single dynamin family protein in the primitive protozoan Giardia lamblia is essential for stage conversion and endocytic transport. Traffic 9: 57–71. 17892527 10.1111/j.1600-0854.2007.00657.x

[17] Touz MC, Kulakova L, Nash TE (2004) Adaptor protein complex 1 mediates the transport of lysosomal proteins from a Golgi-like organelle to peripheral vacuoles in the primitive eukaryote Giardia lamblia. Mol Biol Cell 15: 3053–3060. 15107467 10.1091/mbc.E03-10-0744 PMC452563

[18] Rivero MR, Vranych CV, Bisbal M, Maletto BA, Ropolo AS, et al (2010) Adaptor protein 2 regulates receptor-mediated endocytosis and cyst formation in Giardia lamblia. Biochem J 428: 33–45. 20199400 10.1042/BJ20100096 PMC2861151

[19] Rivero MR, Miras SL, Quiroga R, Ropolo AS, Touz MC (2011) Giardia lamblia low-density lipoprotein receptor-related protein is involved in selective lipoprotein endocytosis and parasite replication. Mol Microbiol 79: 1204–1219. 21205007 10.1111/j.1365-2958.2010.07512.x PMC3043124

[20] Marti M, Li Y, Schraner EM, Wild P, Kohler P, et al (2003) The secretory apparatus of an ancient eukaryote: protein sorting to separate export pathways occurs before formation of transient Golgi-like compartments. Mol Biol Cell 14: 1433–1447. 12686599 10.1091/mbc.E02-08-0467 PMC153112

[21] Lujan HD, Mowatt MR, Conrad JT, Bowers B, Nash TE (1995) Identification of a novel Giardia lamblia cyst wall protein with leucine-rich repeats. Implications for secretory granule formation and protein assembly into the cyst wall. The Journal of biological chemistry 270: 29307–29313. 7493963 10.1074/jbc.270.49.29307

[22] Mowatt MR, Lujan HD, Cotten DB, Bowers B, Yee J, et al (1995) Developmentally regulated expression of a Giardia lamblia cyst wall protein gene. Mol Microbiol 15: 955–963. 7596296 10.1111/j.1365-2958.1995.tb02364.x

[23] Sun CH, McCaffery JM, Reiner DS, Gillin FD (2003) Mining the Giardia lamblia genome for new cyst wall proteins. The Journal of biological chemistry 278: 21701–21708. 12686559 10.1074/jbc.M302023200

[24] Gerwig GJ, van Kuik JA, Leeflang BR, Kamerling JP, Vliegenthart JF, et al (2002) The Giardia intestinalis filamentous cyst wall contains a novel beta(1–3)-N-acetyl-D-galactosamine polymer: a structural and conformational study. Glycobiology 12: 499–505. 12145190 10.1093/glycob/cwf059

[25] Jarroll EL, Manning P, Lindmark DG, Coggins JR, Erlandsen SL (1989) Giardia cyst wall-specific carbohydrate: evidence for the presence of galactosamine. Mol Biochem Parasitol 32: 121–131. 2927442 10.1016/0166-6851(89)90063-7

[26] Stefanic S, Morf L, Kulangara C, Regos A, Sonda S, et al (2009) Neogenesis and maturation of transient Golgi-like cisternae in a simple eukaryote. J Cell Sci 122: 2846–2856. 19622633 10.1242/jcs.049411

[27] Faso C, Konrad C, Schraner EM, Hehl AB (2012) Export of cyst wall material and Golgi organelle neogenesis in Giardia lamblia depend on endoplasmic reticulum exit sites. Cellular microbiology. 10.1111/cmi.12054 23094658

[28] Konrad C, Spycher C, Hehl AB (2010) Selective condensation drives partitioning and sequential secretion of cyst wall proteins in differentiating Giardia lamblia. PLoS Pathog 6: e1000835. 20386711 10.1371/journal.ppat.1000835 PMC2851657

[29] Marti M, Regos A, Li Y, Schraner EM, Wild P, et al (2003) An ancestral secretory apparatus in the protozoan parasite Giardia intestinalis. The Journal of biological chemistry 278: 24837–24848. 12711599 10.1074/jbc.M302082200

[30] Morrison HG, McArthur AG, Gillin FD, Aley SB, Adam RD, et al (2007) Genomic minimalism in the early diverging intestinal parasite Giardia lamblia. Science (New York, NY 317: 1921–1926. 10.1126/science.1143837 17901334

[31] Dacks JB, Walker G, Field MC (2008) Implications of the new eukaryotic systematics for parasitologists. Parasitol Int 57: 97–104. 18180199 10.1016/j.parint.2007.11.004

[32] Stefanic S, Palm D, Svard SG, Hehl AB (2006) Organelle proteomics reveals cargo maturation mechanisms associated with Golgi-like encystation vesicles in the early-diverged protozoan Giardia lamblia. The Journal of biological chemistry 281: 7595–7604. 16407213 10.1074/jbc.M510940200

[33] Jedelsky PL, Dolezal P, Rada P, Pyrih J, Smid O, et al (2011) The minimal proteome in the reduced mitochondrion of the parasitic protist Giardia intestinalis. PLoS One 6: e17285. 21390322 10.1371/journal.pone.0017285 PMC3044749

[34] Huang da W, Sherman BT, Lempicki RA (2009) Systematic and integrative analysis of large gene lists using DAVID bioinformatics resources. Nat Protoc 4: 44–57. 19131956 10.1038/nprot.2008.211

[35] Davids BJ, Reiner DS, Birkeland SR, Preheim SP, Cipriano MJ, et al (2006) A new family of giardial cysteine-rich non-VSP protein genes and a novel cyst protein. PLoS One 1: e44. 17183673 10.1371/journal.pone.0000044 PMC1762436

[36] Chiu PW, Huang YC, Pan YJ, Wang CH, Sun CH (2010) A novel family of cyst proteins with epidermal growth factor repeats in Giardia lamblia. PLoS neglected tropical diseases 4: e677. 20485485 10.1371/journal.pntd.0000677 PMC2867935

[37] DuBois KN, Abodeely M, Sakanari J, Craik CS, Lee M, et al (2008) Identification of the major cysteine protease of Giardia and its role in encystation. The Journal of biological chemistry 283: 18024–18031. 18445589 10.1074/jbc.M802133200 PMC2440617

[38] Castillo-Romero A, Leon-Avila G, Wang CC, Perez Rangel A, Camacho Nuez M, et al (2010) Rab11 and actin cytoskeleton participate in Giardia lamblia encystation, guiding the specific vesicles to the cyst wall. PLoS neglected tropical diseases 4: e697. 20532229 10.1371/journal.pntd.0000697 PMC2879372

[39] Davids BJ, Gilbert MA, Liu Q, Reiner DS, Smith AJ, et al (2011) An atypical proprotein convertase in Giardia lamblia differentiation. Mol Biochem Parasitol 175: 169–180. 21075147 10.1016/j.molbiopara.2010.11.008 PMC3018286

[40] Davids BJ, Mehta K, Fesus L, McCaffery JM, Gillin FD (2004) Dependence of Giardia lamblia encystation on novel transglutaminase activity. Mol Biochem Parasitol 136: 173–180. 15478797 10.1016/j.molbiopara.2004.03.011

[41] Hehl AB, Marti M, Kohler P (2000) Stage-specific expression and targeting of cyst wall protein-green fluorescent protein chimeras in Giardia. Mol Biol Cell 11: 1789–1800. 10793152 10.1091/mbc.11.5.1789 PMC14884

[42] Hehl AB, Marti M (2004) Secretory protein trafficking in Giardia intestinalis. Mol Microbiol 53: 19–28. 15225300 10.1111/j.1365-2958.2004.04115.x

[43] Elias EV, Quiroga R, Gottig N, Nakanishi H, Nash TE, et al (2008) Characterization of SNAREs determines the absence of a typical Golgi apparatus in the ancient eukaryote Giardia lamblia. The Journal of biological chemistry 283: 35996–36010. 18930915 10.1074/jbc.M806545200 PMC2602913

[44] McCaffery JM, Faubert GM, Gillin FD (1994) Giardia lamblia: traffic of a trophozoite variant surface protein and a major cyst wall epitope during growth, encystation, and antigenic switching. Exp Parasitol 79: 236–249. 7525336 10.1006/expr.1994.1087

[45] Svard SG, Meng TC, Hetsko ML, McCaffery JM, Gillin FD (1998) Differentiation-associated surface antigen variation in the ancient eukaryote Giardia lamblia. Mol Microbiol 30: 979–989. 9988475 10.1046/j.1365-2958.1998.01125.x

[46] Hong W, Lev S (2013) Tethering the assembly of SNARE complexes. Trends Cell Biol. 10.1016/j.tcb.2013.09.006 24119662

[47] Leung KF, Dacks JB, Field MC (2008) Evolution of the multivesicular body ESCRT machinery; retention across the eukaryotic lineage. Traffic 9: 1698–1716. 18637903 10.1111/j.1600-0854.2008.00797.x

[48] Morf L, Spycher C, Rehrauer H, Fournier CA, Morrison HG, et al (2010) The transcriptional response to encystation stimuli in Giardia lamblia is restricted to a small set of genes. Eukaryot Cell 9: 1566–1576. 20693303 10.1128/EC.00100-10 PMC2950437

[49] Birkeland SR, Preheim SP, Davids BJ, Cipriano MJ, Palm D, et al (2010) Transcriptome analyses of the Giardia lamblia life cycle. Mol Biochem Parasitol 174: 62–65. 20570699 10.1016/j.molbiopara.2010.05.010 PMC2972195

[50] McArthur AG, Morrison HG, Nixon JE, Passamaneck NQ, Kim U, et al (2000) The Giardia genome project database. FEMS Microbiol Lett 189: 271–273. 10930750 10.1111/j.1574-6968.2000.tb09242.x

[51] Banerjee S, Cui J, Robbins PW, Samuelson J (2008) Use of Giardia, which appears to have a single nucleotide-sugar transporter for UDP-GlcNAc, to identify the UDP-Glc transporter of Entamoeba. Mol Biochem Parasitol 159: 44–53. 18346800 10.1016/j.molbiopara.2008.01.004 PMC4258307

[52] Oppermann U, Filling C, Hult M, Shafqat N, Wu X, et al (2003) Short-chain dehydrogenases/reductases (SDR): the 2002 update. Chemico-biological interactions 143–144: 247–253. 10.1016/s0009-2797(02)00164-3 12604210

[53] Macechko PT, Steimle PA, Lindmark DG, Erlandsen SL, Jarroll EL (1992) Galactosamine-synthesizing enzymes are induced when Giardia encyst. Mol Biochem Parasitol 56: 301–309. 1484552 10.1016/0166-6851(92)90179-n

[54] Lanfredi-Rangel A, Attias M, Reiner DS, Gillin FD, De Souza W (2003) Fine structure of the biogenesis of Giardia lamblia encystation secretory vesicles. J Struct Biol 143: 153–163. 12972352 10.1016/s1047-8477(03)00123-0

[55] Faso C, Hehl AB (2011) Membrane trafficking and organelle biogenesis in Giardia lamblia: use it or lose it. Int J Parasitol 41: 471–480. 21296082 10.1016/j.ijpara.2010.12.014

[56] Faso C, Bischof S, Hehl AB (2013) The proteome landscape of Giardia lamblia encystation. PLoS One in press. 10.1371/journal.pone.0083207 PMC3877021 24391747

[57] Vazquez-Martinez R, Diaz-Ruiz A, Almabouada F, Rabanal-Ruiz Y, Gracia-Navarro F, et al (2012) Revisiting the regulated secretory pathway: from frogs to human. Gen Comp Endocrinol 175: 1–9. 21907200 10.1016/j.ygcen.2011.08.017

[58] Turkewitz AP (2004) Out with a bang! Tetrahymena as a model system to study secretory granule biogenesis. Traffic 5: 63–68. 14690495 10.1046/j.1600-0854.2003.00155.x

[59] Reiner DS, McCaffery M, Gillin FD (1990) Sorting of cyst wall proteins to a regulated secretory pathway during differentiation of the primitive eukaryote, Giardia lamblia. Eur J Cell Biol 53: 142–153. 2076701

[60] Chatterjee A, Carpentieri A, Ratner DM, Bullitt E, Costello CE, et al (2010) Giardia cyst wall protein 1 is a lectin that binds to curled fibrils of the GalNAc homopolymer. PLoS Pathog 6: e1001059. 20808847 10.1371/journal.ppat.1001059 PMC2924369

[61] Abdul-Wahid A, Faubert GM (2004) Similarity in cyst wall protein (CWP) trafficking between encysting Giardia duodenalis trophozoites and CWP-expressing human embryonic kidney-293 cells. Biochemical and biophysical research communications 324: 1069–1080. 15485664 10.1016/j.bbrc.2004.09.167

[62] Beuret N, Stettler H, Renold A, Rutishauser J, Spiess M (2004) Expression of regulated secretory proteins is sufficient to generate granule-like structures in constitutively secreting cells. The Journal of biological chemistry 279: 20242–20249. 14996840 10.1074/jbc.M310613200

[63] Kim T, Tao-Cheng JH, Eiden LE, Loh YP (2001) Chromogranin A, an “on/off” switch controlling dense-core secretory granule biogenesis. Cell 106: 499–509. 11525735 10.1016/s0092-8674(01)00459-7

[64] Voorberg J, Fontijn R, Calafat J, Janssen H, van Mourik JA, et al (1993) Biogenesis of von Willebrand factor-containing organelles in heterologous transfected CV-1 cells. EMBO J 12: 749–758. 8440262 10.1002/j.1460-2075.1993.tb05709.x PMC413262

[65] Scott DC, Schekman R (2008) Role of Sec61p in the ER-associated degradation of short-lived transmembrane proteins. J Cell Biol 181: 1095–1105. 18573918 10.1083/jcb.200804053 PMC2442213

[66] Lopez AB, Sener K, Trosien J, Jarroll EL, van Keulen H (2007) UDP-N-acetylglucosamine 4′-epimerase from the intestinal protozoan Giardia intestinalis lacks UDP-glucose 4′-epimerase activity. The Journal of eukaryotic microbiology 54: 154–160. 17403156 10.1111/j.1550-7408.2007.00246.x

[67] Samuelson J, Banerjee S, Magnelli P, Cui J, Kelleher DJ, et al (2005) The diversity of dolichol-linked precursors to Asn-linked glycans likely results from secondary loss of sets of glycosyltransferases. Proc Natl Acad Sci U S A 102: 1548–1553. 15665075 10.1073/pnas.0409460102 PMC545090

[68] Cao Z, Li C, Higginbotham JN, Franklin JL, Tabb DL, et al (2008) Use of fluorescence-activated vesicle sorting for isolation of Naked2-associated, basolaterally targeted exocytic vesicles for proteomics analysis. Mol Cell Proteomics 7: 1651–1667. 18504258 10.1074/mcp.M700155-MCP200 PMC2528074

[69] Gauthier DJ, Sobota JA, Ferraro F, Mains RE, Lazure C (2008) Flow cytometry-assisted purification and proteomic analysis of the corticotropes dense-core secretory granules. Proteomics 8: 3848–3861. 18704904 10.1002/pmic.200700969 PMC2989539

[70] Brunner Y, Schvartz D, Coute Y, Sanchez JC (2009) Proteomics of regulated secretory organelles. Mass spectrometry reviews 28: 844–867. 19301366 10.1002/mas.20211

[71] Lee YH, Tan HT, Chung MC (2010) Subcellular fractionation methods and strategies for proteomics. Proteomics 10: 3935–3956. 21080488 10.1002/pmic.201000289

[72] Jerlstrom-Hultqvist J, Einarsson E, Xu F, Hjort K, Ek B, et al (2013) Hydrogenosomes in the diplomonad Spironucleus salmonicida. Nat Commun 4: 2493. 24042146 10.1038/ncomms3493 PMC3778541

[73] Aguilar RM, Bustamante JJ, Hernandez PG, Martinez AO, Haro LS (1999) Precipitation of dilute chromatographic samples (ng/ml) containing interfering substances for SDS-PAGE. Analytical biochemistry 267: 344–350. 10036140 10.1006/abio.1998.3018

[74] Vizcaino JA, Cote RG, Csordas A, Dianes JA, Fabregat A, et al (2013) The PRoteomics IDEntifications (PRIDE) database and associated tools: status in 2013. Nucleic Acids Res 41: D1063–1069. 23203882 10.1093/nar/gks1262 PMC3531176

[75] Jimenez-Garcia LF, Zavala G, Chavez-Munguia B, Ramos-Godinez Mdel P, Lopez-Velazquez G, et al (2008) Identification of nucleoli in the early branching protist Giardia duodenalis. Int J Parasitol 38: 1297–1304. 18625508 10.1016/j.ijpara.2008.04.012

