# Supplementary figures and images for: Proteomics of Secretory and Endocytic Organelles in Giardia lamblia
Source: PLoS One. 2014 Apr 14;9(4):e94089. doi: 10.1371/journal.pone.0094089 (PMC3986054; doi:10.1371/journal.pone.0094089)

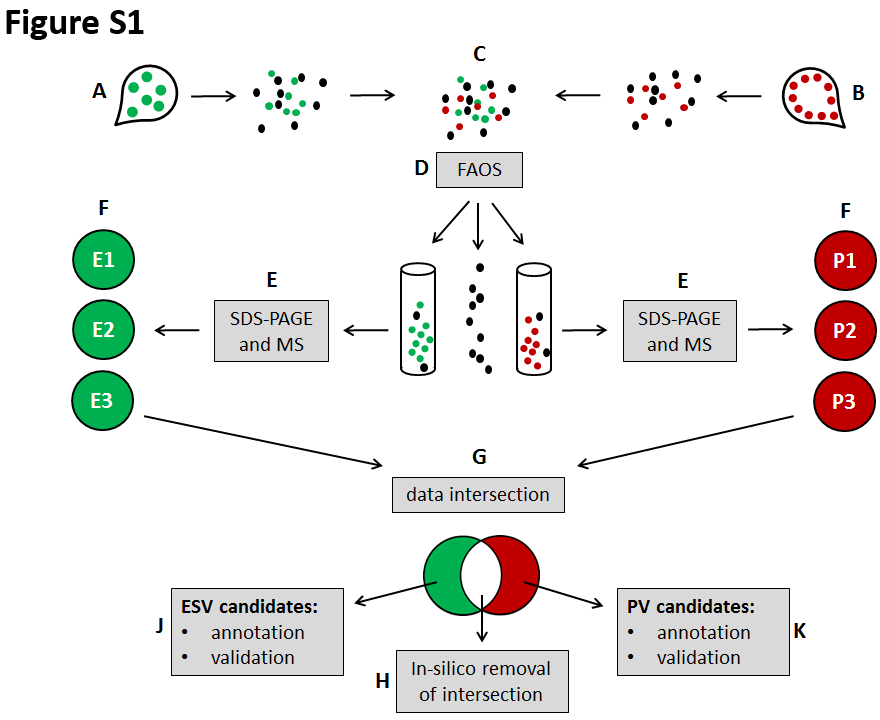

Supplement: Figure S1 — Workflow. CWP3-GFP expressing cells at 13 hours p.i. (A) and wild type trophozoites after endocytic uptake of the fluid phase dye Dextran-AlexaFluor-647 (B) were disrupted by sonication and passed through a 5µm filter. The cleared microsome fractions were mixed (C) and organelles were simultaneously enriched by flow cytometry-assisted organelle sorting (FAOS) (D). Sample preparation and organelle sorting were performed in biological triplicates. Protein precipitates of organelle-enriched fractions were separated by 1D–SDS-PAGE and analyzed by mass spectrometry (MS) (E), resulting in 3 ESV and PV mass spectrometry datasets, each (F). Contaminating proteins were identified by intersecting the ESV and PV MS-datasets (G). A detailed description of the intersection can be found in Figure S2. In silico data filtration, i.e. removal of the data intersection (H) revealed ESV-organelle (J) and PV-organelle (K) specific datasets. (TIF) [file pone.0094089.s001.tif]

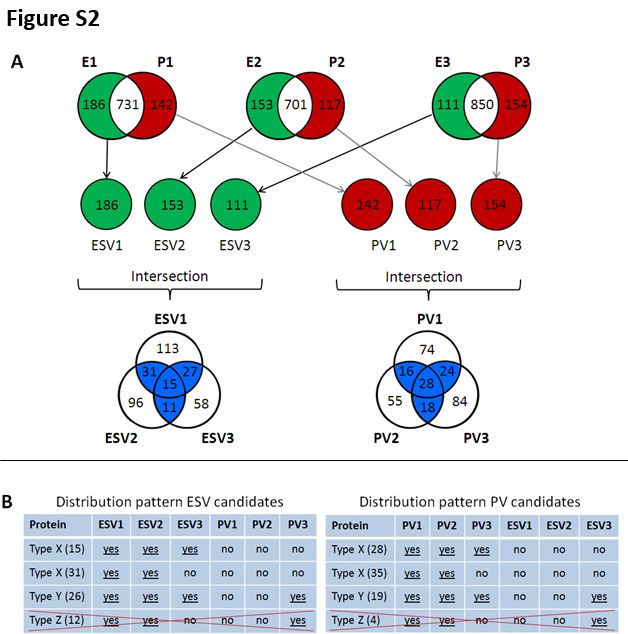

Supplement: Figure S2 — Generation of the MS data intersection. A) Mass spectrometry datasets of ESV-enriched (E1, E2, E3) and PV-enriched (P1, P2, P3) fractions of each replicate were intersected separately (top). The numbers stand for the proteins detected by mass spectrometry. Removal of the intersection revealed proteins exclusively detected in ESV-enriched fractions (middle, left) or PV-enriched fractions (middle, right). From these lists, only proteins occurring in at least two lists were accepted (bottom, blue). The proteins were further analyzed according to their distribution pattern in the six organelle-enriched fractions (B). B) Schematic representation of the protein distribution pattern in ESV- and PV-enriched fractions. ESV candidates (left): proteins of type X were detected exclusively and in at least two of three ESV fractions, proteins of type Y were detected in all ESV fractions and in one PV fraction, proteins of type Z were detected in only two ESV fractions and in one PV fraction. The same is true vice-versa for PV candidates (right). Type Z proteins were removed, resulting in 72 ESV and 82 PV candidate proteins. The respective protein numbers are indicated in brackets. (TIF) [file pone.0094089.s002.tif]

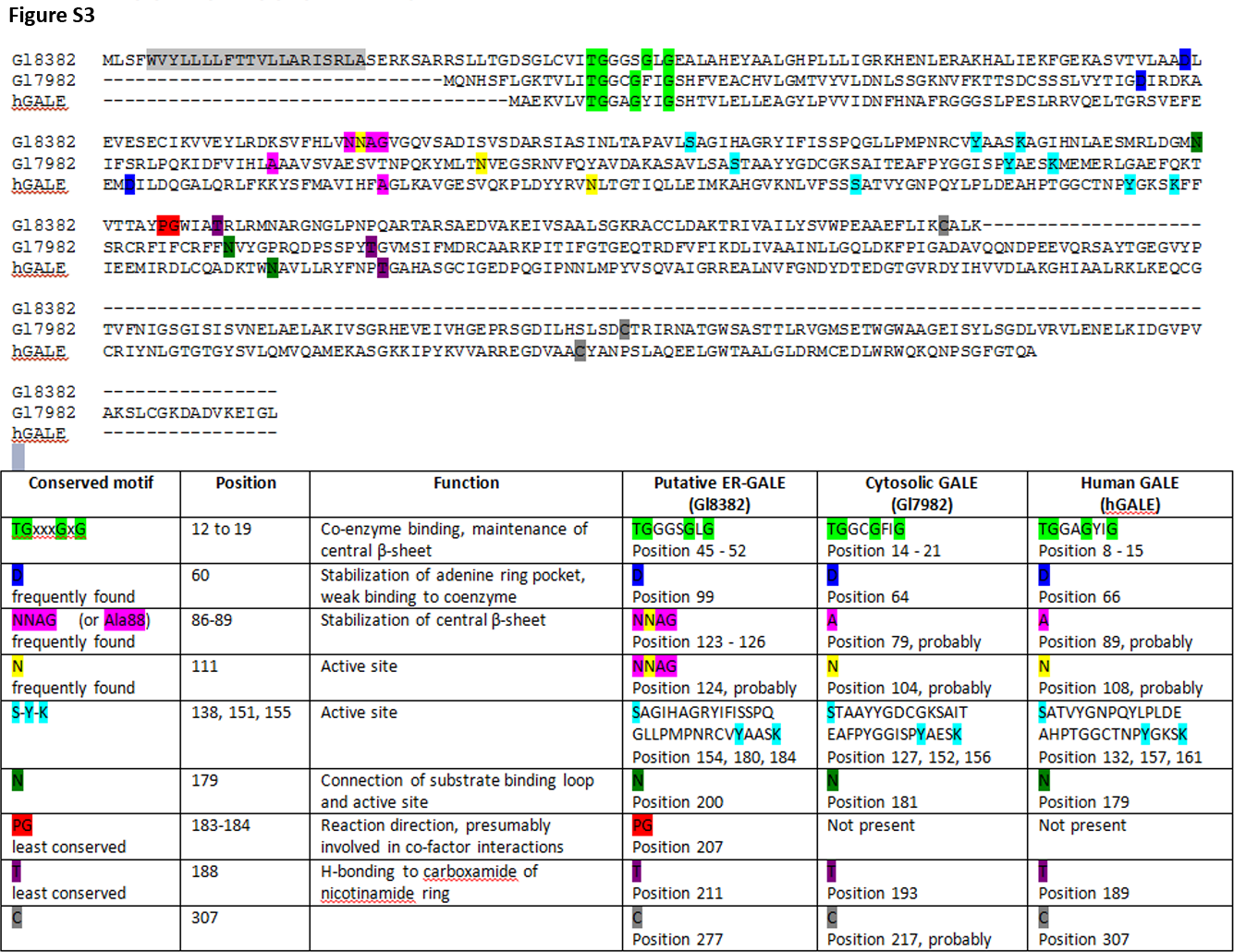

Supplement: Figure S3 — Conserved short chain dehydrogenases (SDH) motifs in Gl8382, Gl7982 and human GALE. Protein sequences of G. lamblia Gl7982 (cytoplasmic GALE, [53]), G. lamblia Gl8382 (putative ER-GALE), and the human GALE (hGALE) were analyzed manually. All conserved sequences required for hGALE function [52] are present in both Giardia GALEs and listed in the table. A conserved PG motif, which is required for the direction of the reaction, is only present in the Giardia ER-GALE. An N-terminal integral membrane domain in the ER-GALE shifts the conserved motif positions for about 40 amino acids towards the C-terminus, compared to the cytoplasmic GALE and hGALE. (TIF) [file pone.0094089.s003.tif]

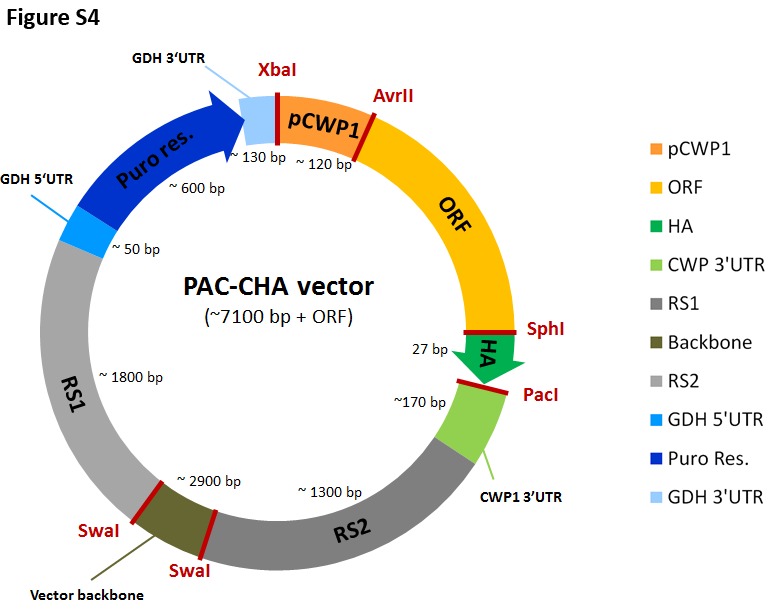

Supplement: Figure S4 — Vector map. Schematic depiction of the vector used for candidate cloning. pCW1: putative promoter region of cyst wall protein 1 (GL50803_5638); ORF: open reading frame; HA: hemagglutinin tag; CWP 3′UTR: 3′ untranslated region of cyst wall protein 1 (GL50803_5638); RS1/2: recombination sites 1 (GL50803_17200) and 2 (GL50803_93938); GDH 5′/3′ UTR: 5′ and 3′ untranslated regions of glutamate dehydrogenase (GL50803_21942); Puro Res.: puromycin N-acetyltransferase. (TIF) [file pone.0094089.s004.tif]

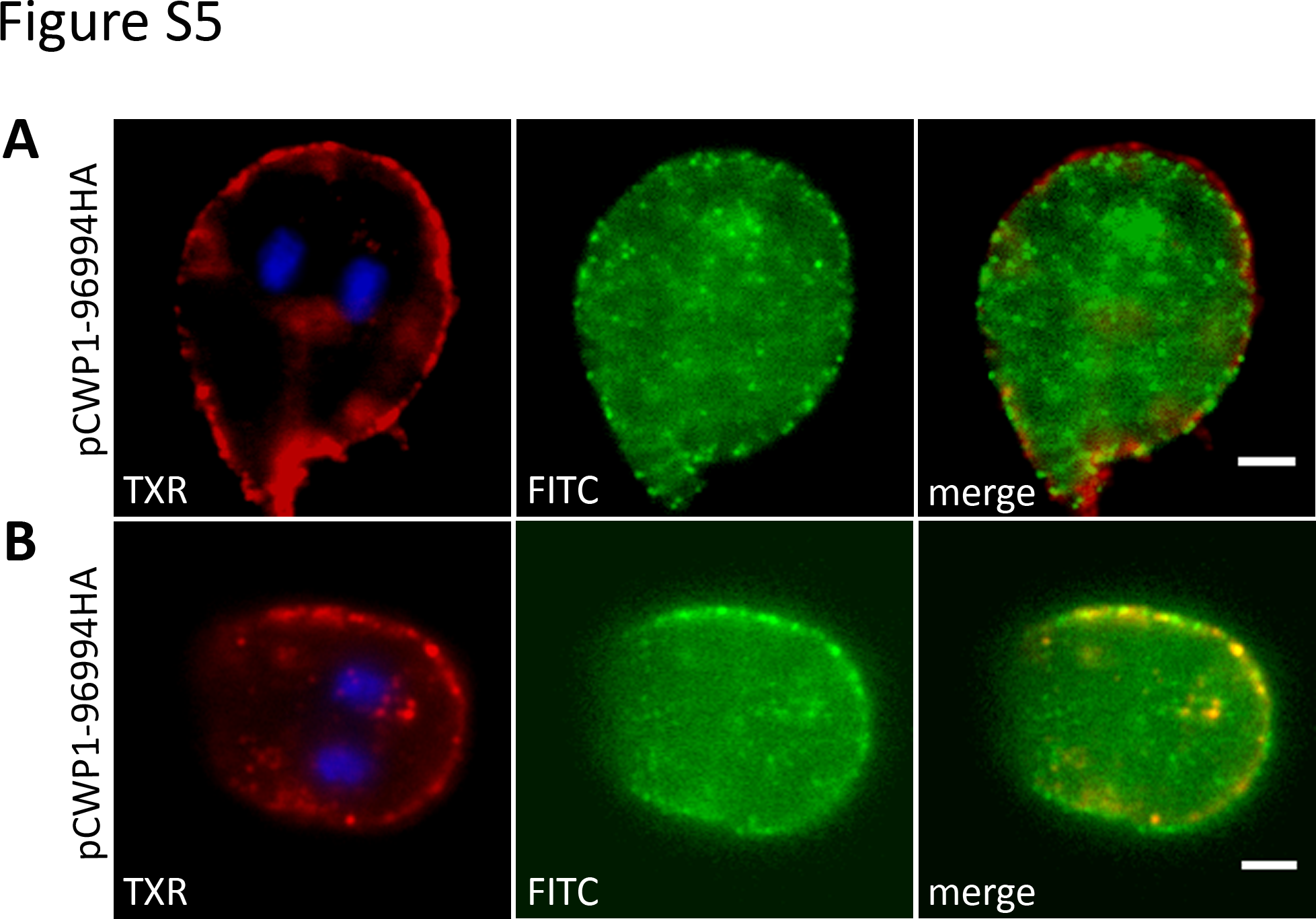

Supplement: Figure S5 — Gl96994HA-expressing cells at 7h post induction of encystation. Recombinant protein expression was detected by fluorescence microscopy with an FITC-coupled anti-HA antibody (green, A and B, middle panels). A) Surface proteins labeled with biotin were detected by fluorescence microscopy in fixed cells after incubation with Streptavidin-Texas Red (red, left panel). B) Visualization of fluid-phase endocytosis of a Dextran-Texas Red marker (red, left panel). Nuclear DNA was labeled with DAPI (blue). pCWP1: inducible CWP1 promoter; TXR: Texas Red; FITC: Fluorescein isothiocyanate. Scale bar: 2 µm. (TIF) [file pone.0094089.s005.tif]
