# Supplementary material for: Proteomics of Secretory and Endocytic Organelles in Giardia lamblia
Source: PLoS One. 2014 Apr 14;9(4):e94089. doi: 10.1371/journal.pone.0094089 (PMC3986054; doi:10.1371/journal.pone.0094089)
Supplement: Table S3 — Oligonucleotide primer sequences. Primers used for cloning of expression constructs. Sequences are in 5′ to 3′ orientation, restriction sites are marked in bold. pCWP1: inducible promoter of G. lamblia cyst wall protein 1; pendo: endogenous promoter, HA: hemagglutinin tag. (DOC) [file pone.0094089.s008.doc]

**Table S3: Oligonucleotides**

*pCWP1:* inducible promoter; *pendo:* endogenous promoter

| **Construct** | **Forward primer (5’ to 3’ direction)** | | **Reverse primer (5’ to 3’ direction)** | |
| --- | --- | --- | --- | --- |
| pCWP1-15483HA | 15483_s_NsiI | CG**ATGCAT**AACGAGACTGTCCGTTTCCTTC | 15483_as_HA_PacI | CG**TTAATTAA**TCACGCGTAGTCTGGGACATCGTATGGGTAATCTTGTTTTTTATTCTTACTGCG |
| pCWP1-10221HA | 10221_s_AvrII | GC**CCTAGG**ATGGAAAAATACCACGGGCTG | 10221_as_SphI | GA**GCATGC**TTCACTGTGTATGGCCGCGAC |
| Pendo-10221HA | p10221_s_XbaI | CG**TCTAGA**GAGTATCTGCACCTCCAGTG | 10221_as_SphI | GA**GCATGC**TTCACTGTGTATGGCCGCGAC |
| pCWP1-88581HA | 88581_s_AvrII | GC**CCTAGG**ATGAAGATTTCCTTGCTCTGTTC | 88581_as_SphI | GA**GCATGC**GAGGAAGGGGAAAATGCAGAAC |
| Pendo-88581HA | p88581_s_XbaI | CG**TCTAGA**GTTTAACCTAATGACCGTTATTG | 88581_as_SphI | GA**GCATGC**GAGGAAGGGGAAAATGCAGAAC |
| pCWP1-22135HA | 22136_s_AvrII | GC**CCTAGG**ATGACTGATACCGAGTCACAG | 22136_as_SphI | GA**GCATGC**GAAGCCACTGGCAATATTAATAAAAAG |
| Pendo-22135HA | p22136_s_XbaI | CG**TCTAGA**CAGAATCCAAGATGCTGGTC | 22136_as_SphI | GA**GCATGC**GAAGCCACTGGCAATATTAATAAAAAG |
| pCWP1-11299HA | 11299_s_AvrII | GC**CCTAGG**ATGCAAGTCGAAGACCCTGGTG | 11299_as_SphI | GA**GCATGC**TTTTTTATTGTACGGACAATTATCAAC |
| Pendo-11299HA | p11299_s_XbaI | CG**TCTAGA**CTGTAGTGGAGTTGTCGGTC | 11299_as_SphI | GA**GCATGC**TTTTTTATTGTACGGACAATTATCAAC |
| pCWP1-11595HA | 11595_Bcl_s | CA**TGATCA**ATGACGACCGCGGCGTC | 11595_SphI_as | GA**GCATGC**ATCCTTGCGTGTTCTAGCTC |
| Pendo-11595HA | p11595_XbaI_s | CG**TCTAGA**CTTGCCCAAGCAGCGGAC | 11595_SphI_as | GA**GCATGC**ATCCTTGCGTGTTCTAGCTC |
| pCWP1-32419HA | 32419_AvrII_s | GC**CCTAGG**ATGAAGCACATAGGTCTCCAC | 32419_SphI_as | GA**GCATGC**GCGAAGAGAAAGGGGCTGC |
| pendo-32419HA | P32419_Xba_for | GC**TCTAG**AGCGCATGCTAGGAATCGC | 32419_SphI_as | GA**GCATGC**GCGAAGAGAAAGGGGCTGC |
| pCWP1-15956HA | 15956_AvrII_s | GC**CCTAGG**ATGGTTCTTCAACTCCGCAG | 15956_PacI_HA_as | CG**TTAATTAA**TCACGCGTAGTCTGGGACATCGTATGGGTACATTGACCACACCATGACAC |
| Pendo-15956HA | p15956_XbaI_s | CG**TCTAGA**CAGTTTGCCCAACACGCTTC | 15956_PacI_HA_as | CG**TTAATTAA**TCACGCGTAGTCTGGGACATCGTATGGGTACATTGACCACACCATGACAC |
| pCWP1-7207HA | 7207_AvrII_s | GC**CCTAGG**ATGCAACAGCAGCAAGACCCAC | 7207_SphI_as | GA**GCATGC**TAAACGATATTGGAGAACACTCTC |
| Pendo-7207HA | 7207_SpeI_s | GC**ACTAGT**CAGATTCTGTGCTATTTGAATAAG | 7207_SphI_as | GA**GCATGC**TAAACGATATTGGAGAACACTCTC |
| pCWP1-7350 | 7350_AvrII_s | GC**CCTAGG**ATGGTGAAAGTGGCCGAAG | 7350_HA_PacI_as | CGTTAATTAATCACGCGTAGTCTGGGACATCGTATGGGTAGGAAACACACAAAGCAGGAATTC |
| Pendo-7350 | 7350_SpeI_s | GC**ACTAGT**CTCACGCGAACTGTAGCCTG | 7350_HA_PacI_as | CGTTAATTAATCACGCGTAGTCTGGGACATCGTATGGGTAGGAAACACACAAAGCAGGAATTC |
| pCWP1-14458HA | 14458_AvrII_s | GC**CCTAGG**ATGAGCAAACATCTGGCCATTG | 14458_SphI_as | GA**GCATGC**CTTGCAAAGGATCTTCCTCATAG |
| Pendo-14458HA | 14458_XbaI_s | CG**TCTAGA**CTTTGGCGGCTTTATGGCAG | 14458_SphI_as | GA**GCATGC**CTTGCAAAGGATCTTCCTCATAG |
| pCWP1-25205HA | 25205_AvrII_s | GC**CCTAGG**ATGGCCGGAAATTACCCTGTC | 25205_HA_PacI_as | CGTTAATTAATCACGCGTAGTCTGGGACATCGTATGGGTAGATTTCCATTAGGCCATAATG |
| Pendo-25205HA | 25205_SpeI_s | GC**ACTAGT**GTACGTTCACACTCTTTACCAG | 25205_HA_PacI_as | CGTTAATTAATCACGCGTAGTCTGGGACATCGTATGGGTAGATTTCCATTAGGCCATAATG |
| Pendo-15156HA | p15156_XbaI_s | CG**TCTAGA**GTACGCTTTTGGCGAGTCAGAG | 15156_SphI_as | GA**GCATGC**CTTTGACATCTTTGCCTTCTGAATC |
| Pendo-10780HA | p10780_XbaI_s | CG**TCTAGA**GTTTCCGGAATAATGTTTGTCAG | 10780_SphI_as | GA**GCATGC**CTTCTGTCTGTACCTCTCTAC |
| pCWP1-9157HA | 9157_SpeI_s | GC**ACTAGT**ATGAACAGCGGGGCTGACTCTG | 9157_SphI_as | GA**GCATGC**TGCGGATACTTCGTAGTTCCGTAG |
| Pendo-9157HA | p9157_XbaI_s | CG**TCTAGA**GATCAGCGCGTCTAATGGCGTG | 9157_SphI_as | GA**GCATGC**TGCGGATACTTCGTAGTTCCGTAG |
| pCWP1-87926HA | 87926_AvrII_s | GC**CCTAGG**ATGTTTGCGGCAGACTCTCAAAAC | 87926_SphI_as | GA**GCATGC**CTCGCCCTTCTCCCTCAAG |
| Pendo-87926HA | p87926_AvrII_s | GC**CCTAGG**GTCGTGAATGACGATGAGACTG | 87926_SphI_as | GA**GCATGC**CTCGCCCTTCTCCCTCAAG |
| Pendo-8382HA | 8382_SpeI_s | GC**ACTAGT**ATGCTGTCTTTCTGGGTTTATC | 8382_HA_PacI_as | CG**TTAATTAA**TCACGCGTAGTCTGGGACATCGTATGGGTACTTAAGAGCACACTTTATTAGGAAC |
| Pendo-7982HA | 7982_SpeI_s | GC**ACTAGT**ATGCAGAACCACTCGTTCCTC | 7982_HA_PacI_as | CG**TTAATTAA**TCACGCGTAGTCTGGGACATCGTATGGGTATAGACCAATCTCCTTGACATC |
| pCWP1-96994HA | 96994_AvrII_s | AC**CCTAGG**ATGACCGATTTTG ATGCACC | 96994_Sph1_as | GT**GCATGC**CTTTTTCACGGCACGGATG |
| pCWP1-15104HA | 15104_AvrII_s | CA**CCTAGG**GCCATTGAAACTTGAGGGAG | 15104_Sph1_as | GT**GCATGC**TGCTTTGAGGAACACCCCC |
| pCWP1-16521HA | 16521_AvrII_s | CG**CCTAGG**ATGAGTTATGCAAAGCAGGCG | 16521_Sph1_as | CT**GCATGC**CAACAACTCGCCGATAGCG |
| pCWP1-15472HA | 15472_AvrII_s | GC**CCTAGG**ATGGGGAAGATAGATCTTACC | 15472_Sph1_as | CG**GCATGC**CTCGACGCTCTGGGCCA |
| pCWP1-15339HA | 15339_AvrII_s | CG**CCTAGG**ATGGGCTCAACAACAAACATT | 15339_HA_PacI_as | GC**TTAATTAA**TCACGCGTAGTCTGGGACATCGTATGGGTAATTTCTGTTCAGGCCAGTAAC |
| Pendo-4270HA | 4270_Xba1_s | GC**TCTAGA**TGGCACAGAGCGC GTCAAC | 4270_HA_PacI_as | GC**TTAATTAA**CTACGCGTAGTCTGGGACATCGTATGGGTAGATCGGTAAATCCACAATATATGC |
| pCWP1-8559HA | 8559_NsiI_s | CG**ATGCAT**ATGTCGACAACATCCAGGAATC | 8559_HA_PacI_as | CG**TTAATTAA**TCACGCGTAGTCTGGGACATCGTATGGGTACTGCTGCACGCAAATGTTCC |
| pCWP1-40224HA | 40224_AvrII_s | GC**CCTAGG**ATGAAGTCTGCGTTCAACCC | 40224_Sph1_as | GC**GCATGC**TAAGAACTTAGGTTGATACAGC |
