# Supplementary material for: Proteomics of Secretory and Endocytic Organelles in Giardia lamblia
Source: PLoS One. 2014 Apr 14;9(4):e94089. doi: 10.1371/journal.pone.0094089 (PMC3986054; doi:10.1371/journal.pone.0094089)
Supplement: Text S1 — Detailed description of mass spectrometry analysis. Detailed description of SDS-PAGE, sample preparation, mass spectrometry analysis, database search and protein identification. (DOC) [file pone.0094089.s009.doc]

**Detailed description of mass spectrometry analysis**

**SDS-PAGE and sample preparation for mass spectrometry**

After thawing, protein samples was boiled for 5 minutes and centrifuged for 1 minute at 16’100xg at room temperature to pellet undissolved material. Samples were separated by 1D-SDS-PAGE using precast 12% Tris-Glycine gels (Invitrogen, IM6000). 4 a- 8 ul of supernatant were loaded, depending on the estimated amount of protein in the respective sample determined in a preceding test run. The gel was incubated in fixing solution (20% (v/v) methanol, 1% (v/v) 85% phosphoric acid, 79% water) for 15 minutes and stained in Roti Blue (20% (v/v) methanol, 20% (v/v) Roti Blue concentrate, 60% water) over night. After destaining the gel for 3 x 5 minutes in washing solution (25% (v/v) methanol, 75% water), each gel band was cut into 21 slices. Reduction of disulfide bridges was performed by covering the vacuum dried gel pieces with sufficient 10mM DTT in 25mM ammonium bicarbonate, pH 8, and incubation for 45 minutes at 60°C. For alkylation of cysteines DTT was removed, and 50mM iodacetamide in 25mM ammonium bicarbonate, pH8, was added for one hour at room temperature. Gel pieces were washed twice in 50% acetonitrile and airdried. In-gel digestion was performed in 25mM ammonium bicarbonate pH 8 containing 50 ng trypsin (Roche, 03708985001). After 5 minutes, gel pieces were overlaid with 25mM ammonium bicarbonate and incubated over night at 37°C. Peptides were extracted by addition of 50% acetonitrile / 5% trifluoroacetic acid to the gel pieces and subsequent removal of supernatant. The step was repeated twice. Peptides were dried and desalted using C18 ZipTips (Millipore, ZTC18S960) and the following solutions: wetting solution (100% Ethanol), washing solution (3% acetonitrile; 0.1% trifluoroacetic acid) and elution solution (80% acetonitrile; 0.1% trifluoroacetic acid). After drying, peptides were resuspended in 3% acetonitrile and 0.2% formic acid and incubated for 15 minutes at room temperature.

**Mass spectrometry**

Mass Spectrometry analysis was performed at the Functional Genomics Center Zurich. Samples were analyzed on a LTQ-Orbitrap XL mass spectrometer (Thermo Fischer Scientific, Bremen, Germany) coupled to an Eksigent-Nano-HPLC system (Eksigent Technologies, Dublin, CA, USA). Solvent composition at the two channels was 0.2% formic acid, 1% acetonitrile for channel A and 0.2% formic acid, 80% acetonitrile for channel B. Peptides were resuspended in 3% acetonitrile and 0.2% formic acid and loaded on a self-made tip column (75 µm × 70 mm) packed with reverse phase C18 material (AQ, 3 μm 200 Å, Bischoff GmbH, Leonberg, Germany) and eluted with a flow rate of 200 nl per min by a gradient from 3 to 10% of B in 5 min, 48% B in 55 min, 97% B in 60 min.  Full-scan MS spectra (300−2000 m/z) were acquired with a resolution of 60000 at 400 m/z after accumulation to a target value of 500000.

Collision induced dissociation (CID) MS/MS spectra were recorded in data dependent manner in the ion trap from the six most intense signals above a threshold of 500, using a normalized collision energy of 35% and an activation time of 30 ms. Charge state screening was enabled and singly charge states were rejected. Precursor masses selected twice for MS/MS were excluded for further selection for 120s. The exclusion window was set to 20 ppm, while the size of the exclusion list was set to a maximum of 500 entries. Samples were acquired using internal lock mass calibration set on m/z 429.088735 and 445.120025.

**Database search and protein identification**

The raw-files from the mass spectrometer were converted into Mascot generic files (mgf) with Mascot Distiller software 2.4.2.0 (Matrix Science Ltd.,London, UK). The peak lists were searched using Mascot Server 2.3 against the *Giardia lamblia* database (<http://tinyurl.com/37z5zqp>) with a concatenated decoy database supplemented with contaminants and The Arabidopsis Information Resource (TAIR9) protein database and the Swissprot database (to increase the database’s size). The final database included 79141 entries. The parameters for precursor tolerance and fragment ion tolerance were set to ± 5 ppm and ± 0.8 Da, respectively. The identification results were loaded into Scaffold 3.0 (Proteome Software, Portland, US) and filtered for a minimal mascot score of 20 for peptide probability, a protein probability greater than 80% and a minimum of 2 unique peptides per protein.
