# Supplementary material for: Proteomics of Secretory and Endocytic Organelles in Giardia lamblia
Source: PLoS One. 2014 Apr 14;9(4):e94089. doi: 10.1371/journal.pone.0094089 (PMC3986054; doi:10.1371/journal.pone.0094089)
Supplement: Text S2 — DAVID functional annotation clustering of data intersection. Functional annotation clustering of the data intersection using the DAVID bioinformatics tool (http://david.abcc.ncifcrf.gov/home.jsp). For each cluster, the enrichment score is given at the top, and functional groups (left) and protein counts (right) within the respective cluster are listed. The 1059 putative contaminants of the data intersection are categorized into 56 annotation clusters. (PDF) [file pone.0094089.s010.pdf]

DAVID Bioinformatics Resources 6.7
National Institute of Allergy and Infectious Diseases (NIAID), NIH

Functional Annotation Clustering

Help and Manual

Current Gene List: Contaminants
Current Background: Giardia intestinalis
1031 DAVID IDs

Options   Classification Stringency   Medium

Rerun using options   Create Sublist

56 Cluster(s)   Download File

Category	Term		Count	P_Value	Benjamini
Annotation Cluster 1	Enrichment Score: 16.12		Count	P_Value	Benjamini
GOTERM_BP_FAT	translation	RT	101	1.2E-29	5.0E-27
SP_PIR_KEYWORDS	ribosomal protein	RT	68	1.8E-27	1.8E-25
GOTERM_MF_FAT	structural constituent of ribosome	RT	65	3.2E-24	1.2E-21
GOTERM_MF_FAT	structural molecule activity	RT	72	7.1E-22	1.3E-19
GOTERM_CC_FAT	ribosome	RT	70	5.2E-19	5.6E-17
KEGG_PATHWAY	Ribosome	RT	65	3.1E-13	1.4E-11
GOTERM_CC_FAT	ribonucleoprotein complex	RT	72	5.0E-8	2.7E-6
GOTERM_CC_FAT	intracellular non-membrane-bounded organelle	RT	94	4.6E-4	1.2E-2
GOTERM_CC_FAT	non-membrane-bounded organelle	RT	94	4.6E-4	1.2E-2
Annotation Cluster 2	Enrichment Score: 5.33		Count	P_Value	Benjamini
SP_PIR_KEYWORDS	Aminoacyl-tRNA synthetase	RT	23	4.2E-10	2.0E-8
GOTERM_MF_FAT	aminoacyl-tRNA ligase activity	RT	24	6.5E-10	7.7E-8
GOTERM_MF_FAT	ligase activity, forming aminoacyl-tRNA and related compounds	RT	24	6.5E-10	7.7E-8
GOTERM_MF_FAT	ligase activity, forming carbon-oxygen bonds	RT	24	6.5E-10	7.7E-8
GOTERM_BP_FAT	tRNA aminoacylation	RT	23	2.8E-8	6.0E-6
GOTERM_BP_FAT	amino acid activation	RT	23	2.8E-8	6.0E-6
GOTERM_BP_FAT	tRNA aminoacylation for protein translation	RT	22	6.7E-8	9.6E-6
KEGG_PATHWAY	Aminoacyl-tRNA biosynthesis	RT	21	1.3E-4	3.0E-3
INTERPRO	Aminoacyl-tRNA synthetase, class II, conserved region	RT	9	3.3E-4	3.7E-2
SP_PIR_KEYWORDS	protein biosynthesis	RT	10	3.5E-4	5.0E-3
GOTERM_BP_FAT	tRNA metabolic process	RT	27	1.4E-3	7.3E-2
INTERPRO	Aminoacyl-tRNA synthetase, class I, conserved site	RT	8	3.5E-3	2.1E-1
GOTERM_BP_FAT	ncRNA metabolic process	RT	30	1.8E-2	4.1E-1
SP_PIR_KEYWORDS	ligase	RT	27	2.1E-2	1.2E-1
INTERPRO	Rossmann-like alpha/beta/alpha sandwich fold	RT	10	7.0E-2	9.3E-1
Annotation Cluster 3	Enrichment Score: 3.38		Count	P_Value	Benjamini
SP_PIR_KEYWORDS	cytoplasm	RT	17	1.6E-6	5.1E-5
SP_PIR_KEYWORDS	Chaperone	RT	12	4.6E-5	9.1E-4
GOTERM_MF_FAT	unfolded protein binding	RT	17	1.7E-4	1.2E-2
INTERPRO	Chaperonin Cpn60/TCP-1	RT	9	3.3E-4	3.7E-2
GOTERM_BP_FAT	protein folding	RT	22	1.0E-3	5.9E-2
INTERPRO	Chaperone, tailless complex polypeptide 1	RT	8	1.0E-3	9.8E-2
INTERPRO	Chaperonin TCP-1, conserved site	RT	8	3.5E-3	2.1E-1
PIR_SUPERFAMILY	PIRSF002584:molecular chaperone t-complex-type	RT	8	6.6E-2	1.0E0
Annotation Cluster 4	Enrichment Score: 3.02		Count	P_Value	Benjamini
GOTERM_BP_FAT	monosaccharide metabolic process	RT	21	5.3E-6	5.7E-4
GOTERM_BP_FAT	hexose metabolic process	RT	18	5.5E-5	4.7E-3
GOTERM_BP_FAT	glucose metabolic process	RT	18	5.5E-5	4.7E-3
GOTERM_BP_FAT	alcohol catabolic process	RT	13	7.0E-4	4.9E-2
GOTERM_BP_FAT	monosaccharide catabolic process	RT	13	7.0E-4	4.9E-2
GOTERM_BP_FAT	hexose catabolic process	RT	13	7.0E-4	4.9E-2
GOTERM_BP_FAT	cellular carbohydrate catabolic process	RT	13	7.0E-4	4.9E-2
GOTERM_BP_FAT	glucose catabolic process	RT	13	7.0E-4	4.9E-2

GOTERM_BP_FAT	carbohydrate catabolic process	RT	13	7.0E-4	4.9E-2
GOTERM_BP_FAT	generation of precursor metabolites and energy	RT	19	2.9E-3	1.2E-1
GOTERM_BP_FAT	glycolysis	RT	10	6.7E-3	2.0E-1
SP_PIR_KEYWORDS	glycolysis	RT	5	1.0E-1	3.7E-1
KEGG_PATHWAY	Glycolysis / Gluconeogenesis	RT	7	1.4E-1	8.1E-1
Annotation Cluster 5	Enrichment Score: 2.71		Count	P_Value	Benjamini
SMART	ANX	RT	11	1.2E-4	5.4E-3
INTERPRO	Alpha giardin	RT	11	1.5E-4	3.3E-2
INTERPRO	Annexin	RT	13	1.8E-4	2.3E-2
GOTERM_MF_FAT	calcium-dependent phospholipid binding	RT	17	4.3E-4	2.5E-2
SP_PIR_KEYWORDS	annexin	RT	13	7.8E-4	8.5E-3
INTERPRO	Annexin repeat	RT	11	1.2E-3	1.1E-1
GOTERM_MF_FAT	phospholipid binding	RT	18	3.4E-3	1.6E-1
GOTERM_MF_FAT	calcium ion binding	RT	22	5.2E-2	8.0E-1
GOTERM_MF_FAT	lipid binding	RT	19	5.4E-2	7.6E-1
GOTERM_BP_FAT	cytoskeleton organization	RT	11	6.5E-2	7.8E-1
Annotation Cluster 6	Enrichment Score: 2.26		Count	P_Value	Benjamini
INTERPRO	Proteasome, subunit alpha/beta	RT	14	1.0E-6	9.2E-4
SP_PIR_KEYWORDS	proteasome	RT	18	2.6E-6	6.5E-5
INTERPRO	Proteasome, alpha and beta subunits	RT	13	3.3E-6	1.5E-3
GOTERM_MF_FAT	threonine-type endopeptidase activity	RT	14	1.4E-5	1.2E-3
GOTERM_MF_FAT	threonine-type peptidase activity	RT	14	1.4E-5	1.2E-3
GOTERM_CC_FAT	proteasome complex	RT	19	3.8E-4	1.3E-2
GOTERM_CC_FAT	proteasome core complex	RT	14	5.7E-4	1.2E-2
KEGG_PATHWAY	Proteasome	RT	25	1.3E-3	1.9E-2
GOTERM_CC_FAT	cytosol	RT	23	2.0E-3	3.5E-2
INTERPRO	Proteasome, alpha-subunit, conserved site	RT	7	3.1E-3	2.3E-1
SP_PIR_KEYWORDS	threonine protease	RT	6	1.8E-2	1.2E-1
GOTERM_BP_FAT	protein catabolic process	RT	23	2.3E-2	4.6E-1
GOTERM_MF_FAT	peptidase activity	RT	38	2.5E-2	6.0E-1
SP_PIR_KEYWORDS	Protease	RT	19	2.8E-2	1.4E-1
GOTERM_BP_FAT	macromolecule catabolic process	RT	23	3.3E-2	5.7E-1
GOTERM_MF_FAT	peptidase activity, acting on L-amino acid peptides	RT	32	5.7E-2	7.3E-1
GOTERM_MF_FAT	endopeptidase activity	RT	22	1.1E-1	8.4E-1
GOTERM_BP_FAT	proteolysis involved in cellular protein catabolic process	RT	18	1.4E-1	9.2E-1
GOTERM_BP_FAT	cellular protein catabolic process	RT	18	1.4E-1	9.2E-1
GOTERM_BP_FAT	ubiquitin-dependent protein catabolic process	RT	9	1.6E-1	9.5E-1
PIR_SUPERFAMILY	PIRSF001212:multicatalytic endopeptidase complex chain C9	RT	6	1.7E-1	1.0E0
GOTERM_BP_FAT	cellular macromolecule catabolic process	RT	18	1.8E-1	9.5E-1
GOTERM_BP_FAT	proteolysis	RT	36	6.2E-1	1.0E0
GOTERM_BP_FAT	modification-dependent protein catabolic process	RT	11	6.7E-1	1.0E0
GOTERM_BP_FAT	modification-dependent macromolecule catabolic process	RT	11	6.7E-1	1.0E0
Annotation Cluster 7	Enrichment Score: 2.15		Count	P_Value	Benjamini
INTERPRO	Thioredoxin-like	RT	12	5.0E-5	1.5E-2
INTERPRO	Thioredoxin fold	RT	16	1.6E-4	2.8E-2
GOTERM_BP_FAT	cellular homeostasis	RT	15	3.5E-3	1.3E-1
GOTERM_BP_FAT	cell redox homeostasis	RT	14	6.6E-3	2.1E-1
GOTERM_BP_FAT	homeostatic process	RT	15	7.4E-3	2.0E-1
INTERPRO	Thioredoxin domain	RT	6	9.4E-3	4.1E-1
INTERPRO	Thioredoxin, core	RT	6	2.5E-2	7.4E-1
INTERPRO	Thioredoxin-like subdomain	RT	5	2.7E-2	7.5E-1
GOTERM_MF_FAT	antioxidant activity	RT	6	6.1E-2	7.1E-1
INTERPRO	Thioredoxin, conserved site	RT	5	6.3E-2	9.2E-1
INTERPRO	Alkyl hydroperoxide reductase/ Thiol specific antioxidant/ Mal allergen	RT	5	6.3E-2	9.2E-1
Annotation Cluster 8	Enrichment Score: 2.05		Count	P_Value	Benjamini
INTERPRO	Aminoacyl-tRNA synthetase, class II, conserved region	RT	9	3.3E-4	3.7E-2

	Category	Term		Count	P_Value	Benjamini
	INTERPRO	Aminoacyl-tRNA synthetase, class II (G, H, P and S), conserved region	RT	5	2.7E-2	7.5E-1
	INTERPRO	Anticodon-binding	RT	4	7.7E-2	9.4E-1
Annotation Cluster 9		Enrichment Score: 1.96		Count	P_Value	Benjamini
	SMART	AAA	RT	29	6.7E-4	2.0E-2
	INTERPRO	ATPase, AAA-type, conserved site	RT	10	6.5E-3	3.2E-1
	INTERPRO	26S proteasome subunit P45	RT	5	2.7E-2	7.5E-1
	INTERPRO	ATPase, AAA-type, core	RT	13	3.4E-2	8.1E-1
	INTERPRO	ATPase, AAA+ type, core	RT	29	3.7E-2	8.2E-1
Annotation Cluster 10		Enrichment Score: 1.24		Count	P_Value	Benjamini
	INTERPRO	Aminoacyl-tRNA synthetase, class II, conserved region	RT	9	3.3E-4	3.7E-2
	INTERPRO	Nucleic acid binding, OB-fold, tRNA/helicase-type	RT	4	7.7E-2	9.4E-1
	INTERPRO	Aminoacyl-tRNA synthetase, class II (D, K and N)	RT	3	2.0E-1	1.0E0
	INTERPRO	Aminoacyl-tRNA synthetase, class II (D, K and N)-like	RT	3	2.0E-1	1.0E0
	PIR_SUPERFAMILY	PIRSF001525:lysine-tRNA ligase	RT	3	5.8E-1	1.0E0
Annotation Cluster 11		Enrichment Score: 1.2		Count	P_Value	Benjamini
	GOTERM_MF_FAT	translation factor activity, nucleic acid binding	RT	19	1.4E-2	4.2E-1
	SP_PIR_KEYWORDS	Initiation factor	RT	11	6.5E-2	2.6E-1
	GOTERM_MF_FAT	translation initiation factor activity	RT	14	7.0E-2	7.5E-1
	GOTERM_BP_FAT	translational initiation	RT	7	2.6E-1	9.8E-1
Annotation Cluster 12		Enrichment Score: 1.08		Count	P_Value	Benjamini
	GOTERM_MF_FAT	cofactor binding	RT	21	7.5E-3	2.9E-1
	GOTERM_MF_FAT	vitamin B6 binding	RT	8	2.9E-2	6.2E-1
	GOTERM_MF_FAT	pyridoxal phosphate binding	RT	8	2.9E-2	6.2E-1
	SP_PIR_KEYWORDS	Aminotransferase	RT	5	4.6E-2	2.0E-1
	INTERPRO	Pyridoxal phosphate-dependent transferase, major region, subdomain 1	RT	6	5.1E-2	8.8E-1
	GOTERM_MF_FAT	vitamin binding	RT	8	5.6E-2	7.5E-1
	GOTERM_MF_FAT	transaminase activity	RT	5	5.8E-2	7.2E-1
	INTERPRO	Pyridoxal phosphate-dependent transferase, major region, subdomain 2	RT	3	2.0E-1	1.0E0
	INTERPRO	Aminotransferase, class V/Cysteine desulfurase	RT	3	2.0E-1	1.0E0
	GOTERM_MF_FAT	transferase activity, transferring nitrogenous groups	RT	5	2.1E-1	9.5E-1
	INTERPRO	Aminotransferase, class I and II	RT	3	4.5E-1	1.0E0
	KEGG_PATHWAY	Alanine, aspartate and glutamate metabolism	RT	3	6.2E-1	9.9E-1
Annotation Cluster 13		Enrichment Score: 1.03		Count	P_Value	Benjamini
	SP_PIR_KEYWORDS	lyase	RT	11	4.4E-2	2.0E-1
	GOTERM_MF_FAT	carbon-nitrogen lyase activity	RT	4	1.3E-1	8.6E-1
	GOTERM_MF_FAT	ammonia-lyase activity	RT	4	1.3E-1	8.6E-1
Annotation Cluster 14		Enrichment Score: 1.03		Count	P_Value	Benjamini
	SP_PIR_KEYWORDS	gtp-binding	RT	21	2.1E-2	1.3E-1
	INTERPRO	Small GTP-binding protein	RT	11	3.8E-2	8.2E-1
	GOTERM_MF_FAT	guanyl ribonucleotide binding	RT	24	5.4E-2	7.8E-1
	GOTERM_MF_FAT	GTP binding	RT	24	5.4E-2	7.8E-1
	GOTERM_MF_FAT	guanyl nucleotide binding	RT	24	5.4E-2	7.8E-1
	GOTERM_MF_FAT	GTPase activity	RT	9	2.3E-1	9.6E-1
	GOTERM_BP_FAT	small GTPase mediated signal transduction	RT	9	3.1E-1	9.9E-1
	GOTERM_BP_FAT	intracellular signaling cascade	RT	9	6.8E-1	1.0E0
Annotation Cluster 15		Enrichment Score: 0.98		Count	P_Value	Benjamini
	SP_PIR_KEYWORDS	threonine protease	RT	6	1.8E-2	1.2E-1
	INTERPRO	Proteasome, beta-type subunit, conserved site	RT	4	7.7E-2	9.4E-1
	SP_PIR_KEYWORDS	nucleus	RT	10	8.6E-1	9.9E-1
Annotation Cluster 16		Enrichment Score: 0.95		Count	P_Value	Benjamini
	COG_ONTOLOGY	Cytoskeleton	RT	12	3.3E-2	2.8E-1
	INTERPRO	ATPase associated with various cellular activities, AAA-5	RT	5	6.3E-2	9.2E-1
	INTERPRO	Dynein heavy chain, N-terminal region 2	RT	7	6.8E-2	9.3E-1
	INTERPRO	Dynein heavy chain, N-terminal region 1	RT	4	7.7E-2	9.4E-1
	SP_PIR_KEYWORDS	coiled coil	RT	4	1.1E-1	3.8E-1
	INTERPRO	Dynein heavy chain	RT	6	2.5E-1	1.0E0

GOTERM_CC_FAT	dynein complex	RT	6	7.9E-1	1.0E0
Annotation Cluster 17	Enrichment Score: 0.89		Count	P_Value	Benjamini
GOTERM_BP_FAT	translational elongation	RT	6	1.0E-1	8.6E-1
GOTERM_MF_FAT	translation elongation factor activity	RT	6	1.2E-1	8.5E-1
SP_PIR_KEYWORDS	elongation factor	RT	5	1.8E-1	5.0E-1
Annotation Cluster 18	Enrichment Score: 0.85		Count	P_Value	Benjamini
GOTERM_MF_FAT	electron carrier activity	RT	13	7.8E-2	7.7E-1
INTERPRO	4Fe-4S ferredoxin, iron-sulphur binding, conserved site	RT	5	1.1E-1	9.8E-1
GOTERM_MF_FAT	4 iron, 4 sulfur cluster binding	RT	6	1.2E-1	8.5E-1
GOTERM_MF_FAT	metal cluster binding	RT	11	1.4E-1	8.6E-1
GOTERM_MF_FAT	iron-sulfur cluster binding	RT	11	1.4E-1	8.6E-1
INTERPRO	4Fe-4S ferredoxin, iron-sulphur binding, subgroup	RT	5	2.5E-1	1.0E0
INTERPRO	4Fe-4S ferredoxin, iron-sulpur binding domain	RT	5	2.5E-1	1.0E0
Annotation Cluster 19	Enrichment Score: 0.84		Count	P_Value	Benjamini
GOTERM_BP_FAT	alcohol biosynthetic process	RT	5	9.3E-2	8.5E-1
GOTERM_BP_FAT	cellular carbohydrate biosynthetic process	RT	6	1.0E-1	8.6E-1
GOTERM_BP_FAT	carbohydrate biosynthetic process	RT	6	1.0E-1	8.6E-1
GOTERM_BP_FAT	pyruvate metabolic process	RT	4	1.9E-1	9.6E-1
GOTERM_BP_FAT	monosaccharide biosynthetic process	RT	4	1.9E-1	9.6E-1
GOTERM_BP_FAT	gluconeogenesis	RT	4	1.9E-1	9.6E-1
GOTERM_BP_FAT	hexose biosynthetic process	RT	4	1.9E-1	9.6E-1
Annotation Cluster 20	Enrichment Score: 0.78		Count	P_Value	Benjamini
SMART	RAB	RT	4	3.7E-2	5.8E-1
INTERPRO	Small GTP-binding protein	RT	11	3.8E-2	8.2E-1
INTERPRO	Ras small GTPase, Rab type	RT	4	7.7E-2	9.4E-1
INTERPRO	Ras	RT	7	1.0E-1	9.7E-1
GOTERM_BP_FAT	small GTPase mediated signal transduction	RT	9	3.1E-1	9.9E-1
INTERPRO	Ras GTPase	RT	6	3.2E-1	1.0E0
GOTERM_BP_FAT	intracellular signaling cascade	RT	9	6.8E-1	1.0E0
PIR_SUPERFAMILY	PIRSF001710:Ras-related protein Rab	RT	4	7.5E-1	1.0E0
Annotation Cluster 21	Enrichment Score: 0.75		Count	P_Value	Benjamini
INTERPRO	FAD-dependent pyridine nucleotide-disulphide oxidoreductase	RT	4	7.7E-2	9.4E-1
INTERPRO	Pyridine nucleotide-disulphide oxidoreductase, NAD-binding region	RT	3	2.0E-1	1.0E0
GOTERM_MF_FAT	FAD binding	RT	4	3.7E-1	9.9E-1
Annotation Cluster 22	Enrichment Score: 0.7		Count	P_Value	Benjamini
SMART	DnaJ	RT	5	8.0E-2	7.2E-1
INTERPRO	Molecular chaperone, heat shock protein, Hsp40, DnaJ	RT	5	1.1E-1	9.8E-1
INTERPRO	Heat shock protein DnaJ, N-terminal	RT	5	1.8E-1	1.0E0
INTERPRO	Chaperone DnaJ, C-terminal	RT	3	2.0E-1	1.0E0
INTERPRO	Heat shock protein DnaJ	RT	3	2.0E-1	1.0E0
GOTERM_MF_FAT	heat shock protein binding	RT	5	3.1E-1	9.7E-1
PIR_SUPERFAMILY	PIRSF002585:heat shock protein DnaJ	RT	3	5.8E-1	1.0E0
Annotation Cluster 23	Enrichment Score: 0.63		Count	P_Value	Benjamini
SMART	S_TKc	RT	28	2.8E-5	2.5E-3
SP_PIR_KEYWORDS	nucleotide-binding	RT	118	1.1E-4	1.8E-3
INTERPRO	Serine/threonine protein kinase	RT	28	3.3E-3	2.2E-1
SP_PIR_KEYWORDS	atp-binding	RT	102	1.7E-2	1.3E-1
INTERPRO	Protein kinase, ATP binding site	RT	31	8.3E-2	9.5E-1
SP_PIR_KEYWORDS	serine/threonine-protein kinase	RT	26	1.3E-1	4.2E-1
INTERPRO	Serine/threonine protein kinase, active site	RT	30	2.6E-1	1.0E0
GOTERM_MF_FAT	protein serine/threonine kinase activity	RT	37	6.4E-1	1.0E0
GOTERM_MF_FAT	purine nucleotide binding	RT	198	8.7E-1	1.0E0
GOTERM_MF_FAT	purine ribonucleotide binding	RT	194	9.0E-1	1.0E0
GOTERM_MF_FAT	ribonucleotide binding	RT	194	9.0E-1	1.0E0
GOTERM_MF_FAT	nucleotide binding	RT	209	9.1E-1	1.0E0
INTERPRO	Serine/threonine protein kinase-related	RT	60	9.1E-1	1.0E0
GOTERM_MF_FAT	purine nucleoside binding	RT	174	9.7E-1	1.0E0

GOTERM_MF_FAT	adenyl nucleotide binding	RT	174	9.7E-1	1.0E0
INTERPRO	Protein kinase, core	RT	65	9.7E-1	1.0E0
GOTERM_MF_FAT	nucleoside binding	RT	175	9.8E-1	1.0E0
GOTERM_MF_FAT	adenyl ribonucleotide binding	RT	170	9.8E-1	1.0E0
GOTERM_MF_FAT	ATP binding	RT	170	9.8E-1	1.0E0
SP_PIR_KEYWORDS	kinase	RT	90	1.0E0	1.0E0
GOTERM_MF_FAT	protein kinase activity	RT	66	1.0E0	1.0E0
GOTERM_BP_FAT	phosphorylation	RT	74	1.0E0	1.0E0
GOTERM_BP_FAT	phosphate metabolic process	RT	78	1.0E0	1.0E0
GOTERM_BP_FAT	phosphorus metabolic process	RT	78	1.0E0	1.0E0
GOTERM_BP_FAT	protein amino acid phosphorylation	RT	65	1.0E0	1.0E0
Annotation Cluster 24	Enrichment Score: 0.6	G	Count	P_Value	Benjamini
GOTERM_MF_FAT	intramolecular transferase activity, phosphotransferases	RT	4	1.3E-1	8.6E-1
INTERPRO	Alpha-D-phosphohexomutase, alpha/beta/alpha domain I	RT	3	2.0E-1	1.0E0
GOTERM_MF_FAT	intramolecular transferase activity	RT	5	6.0E-1	1.0E0
Annotation Cluster 25	Enrichment Score: 0.5	G	Count	P_Value	Benjamini
GOTERM_BP_FAT	establishment of protein localization	RT	19	7.4E-2	8.1E-1
GOTERM_BP_FAT	protein transport	RT	19	7.4E-2	8.1E-1
GOTERM_BP_FAT	vesicle-mediated transport	RT	17	8.1E-2	8.2E-1
GOTERM_BP_FAT	protein localization	RT	19	1.0E-1	8.6E-1
GOTERM_BP_FAT	cellular protein localization	RT	11	4.8E-1	1.0E0
GOTERM_BP_FAT	cellular macromolecule localization	RT	11	4.8E-1	1.0E0
GOTERM_BP_FAT	intracellular protein transport	RT	11	4.8E-1	1.0E0
GOTERM_BP_FAT	intracellular transport	RT	13	4.8E-1	1.0E0
GOTERM_CC_FAT	coated membrane	RT	7	7.7E-1	1.0E0
GOTERM_CC_FAT	membrane coat	RT	7	7.7E-1	1.0E0
GOTERM_CC_FAT	Golgi apparatus	RT	5	8.8E-1	1.0E0
GOTERM_CC_FAT	endomembrane system	RT	6	9.6E-1	1.0E0
Annotation Cluster 26	Enrichment Score: 0.48	G	Count	P_Value	Benjamini
GOTERM_BP_FAT	generation of precursor metabolites and energy	RT	19	2.9E-3	1.2E-1
GOTERM_MF_FAT	cation-transporting ATPase activity	RT	7	1.0E-1	8.5E-1
GOTERM_MF_FAT	proton-transporting ATPase activity, rotational mechanism	RT	5	2.1E-1	9.5E-1
GOTERM_BP_FAT	nucleobase, nucleoside and nucleotide biosynthetic process	RT	21	2.2E-1	9.8E-1
GOTERM_BP_FAT	nucleobase, nucleoside, nucleotide and nucleic acid biosynthetic process	RT	21	2.2E-1	9.8E-1
GOTERM_BP_FAT	nitrogen compound biosynthetic process	RT	24	2.3E-1	9.7E-1
GOTERM_BP_FAT	hydrogen transport	RT	9	2.3E-1	9.7E-1
GOTERM_BP_FAT	proton transport	RT	9	2.3E-1	9.7E-1
GOTERM_BP_FAT	oxidative phosphorylation	RT	8	2.5E-1	9.8E-1
GOTERM_BP_FAT	ATP synthesis coupled proton transport	RT	8	2.5E-1	9.8E-1
GOTERM_BP_FAT	energy coupled proton transport, down electrochemical gradient	RT	8	2.5E-1	9.8E-1
GOTERM_MF_FAT	hydrolase activity, acting on acid anhydrides, catalyzing transmembrane movement of substances	RT	14	2.5E-1	9.6E-1
GOTERM_BP_FAT	purine nucleotide biosynthetic process	RT	15	2.6E-1	9.8E-1
GOTERM_MF_FAT	ATPase activity, coupled to transmembrane movement of ions	RT	10	2.6E-1	9.7E-1
GOTERM_MF_FAT	monovalent inorganic cation transmembrane transporter activity	RT	8	2.7E-1	9.6E-1
GOTERM_MF_FAT	hydrogen ion transmembrane transporter activity	RT	8	2.7E-1	9.6E-1
GOTERM_BP_FAT	nucleoside triphosphate metabolic process	RT	14	2.8E-1	9.8E-1
GOTERM_BP_FAT	nucleoside triphosphate biosynthetic process	RT	14	2.8E-1	9.8E-1
GOTERM_BP_FAT	purine nucleoside triphosphate metabolic process	RT	14	2.8E-1	9.8E-1
GOTERM_BP_FAT	purine nucleoside triphosphate biosynthetic process	RT	14	2.8E-1	9.8E-1
GOTERM_BP_FAT	ribonucleoside triphosphate biosynthetic process	RT	14	2.8E-1	9.8E-1
GOTERM_BP_FAT	purine ribonucleoside triphosphate metabolic process	RT	14	2.8E-1	9.8E-1
GOTERM_BP_FAT	purine ribonucleoside triphosphate biosynthetic process	RT	14	2.8E-1	9.8E-1
GOTERM_BP_FAT	ribonucleoside triphosphate metabolic process	RT	14	2.8E-1	9.8E-1

GOTERM_MF_FAT	inorganic cation transmembrane transporter activity	RT	9	3.0E-1	9.7E-1
GOTERM_BP_FAT	monovalent inorganic cation transport	RT	9	3.1E-1	9.9E-1
GOTERM_BP_FAT	purine nucleotide metabolic process	RT	15	3.1E-1	9.9E-1
INTERPRO	Haloacid dehalogenase-like hydrolase	RT	5	3.3E-1	1.0E0
GOTERM_BP_FAT	ion transmembrane transport	RT	8	3.3E-1	9.9E-1
GOTERM_BP_FAT	ribonucleotide metabolic process	RT	14	3.3E-1	9.9E-1
GOTERM_BP_FAT	purine ribonucleotide metabolic process	RT	14	3.3E-1	9.9E-1
GOTERM_BP_FAT	purine ribonucleotide biosynthetic process	RT	14	3.3E-1	9.9E-1
GOTERM_BP_FAT	ribonucleotide biosynthetic process	RT	14	3.3E-1	9.9E-1
GOTERM_CC_FAT	proton-transporting two-sector ATPase complex, catalytic domain	RT	6	3.4E-1	9.9E-1
GOTERM_MF_FAT	primary active transmembrane transporter activity	RT	13	3.8E-1	9.9E-1
GOTERM_MF_FAT	P-P-bond-hydrolysis-driven transmembrane transporter activity	RT	13	3.8E-1	9.9E-1
GOTERM_MF_FAT	ATPase activity, coupled to transmembrane movement of substances	RT	12	4.2E-1	9.9E-1
GOTERM_MF_FAT	ATPase activity, coupled to movement of substances	RT	12	4.2E-1	9.9E-1
GOTERM_BP_FAT	nucleotide biosynthetic process	RT	18	4.2E-1	1.0E0
GOTERM_BP_FAT	ATP metabolic process	RT	12	4.5E-1	1.0E0
GOTERM_BP_FAT	ATP biosynthetic process	RT	12	4.5E-1	1.0E0
GOTERM_CC_FAT	proton-transporting two-sector ATPase complex	RT	9	4.7E-1	1.0E0
KEGG_PATHWAY	Oxidative phosphorylation	RT	9	5.3E-1	9.8E-1
SP_PIR_KEYWORDS	ion transport	RT	3	5.4E-1	8.9E-1
GOTERM_BP_FAT	cation transport	RT	11	5.4E-1	1.0E0
GOTERM_BP_FAT	ion transport	RT	11	5.4E-1	1.0E0
INTERPRO	ATPase, P-type, ATPase-associated region	RT	4	6.6E-1	1.0E0
GOTERM_BP_FAT	transmembrane transport	RT	9	6.8E-1	1.0E0
INTERPRO	ATPase, P-type, K/Mg/Cd/Cu/Zn/Na/Ca/Na/H-transporter	RT	4	7.2E-1	1.0E0
INTERPRO	ATPase, P-type phosphorylation site	RT	3	7.3E-1	1.0E0
GOTERM_MF_FAT	ATPase activity	RT	32	7.6E-1	1.0E0
GOTERM_MF_FAT	ATPase activity, coupled to transmembrane movement of ions, phosphorylative mechanism	RT	4	8.6E-1	1.0E0
GOTERM_CC_FAT	proton-transporting V-type ATPase complex	RT	4	9.0E-1	1.0E0
SP_PIR_KEYWORDS	transmembrane	RT	8	9.2E-1	1.0E0
GOTERM_MF_FAT	ATPase activity, coupled	RT	19	9.8E-1	1.0E0
Annotation Cluster 27	Enrichment Score: 0.46		Count	P_Value	Benjamini
SP_PIR_KEYWORDS	Rotamase	RT	5	2.6E-1	6.3E-1
GOTERM_MF_FAT	cis-trans isomerase activity	RT	5	3.1E-1	9.7E-1
GOTERM_MF_FAT	peptidyl-prolyl cis-trans isomerase activity	RT	5	3.1E-1	9.7E-1
INTERPRO	Peptidyl-prolyl cis-trans isomerase, FKBP-type	RT	3	5.6E-1	1.0E0
Annotation Cluster 28	Enrichment Score: 0.45		Count	P_Value	Benjamini
GOTERM_MF_FAT	GTPase activity	RT	9	2.3E-1	9.6E-1
INTERPRO	Protein synthesis factor, GTP-binding	RT	4	4.2E-1	1.0E0
INTERPRO	Translation elongation factor EFTu/EF1A, domain 2	RT	3	4.5E-1	1.0E0
Annotation Cluster 29	Enrichment Score: 0.42		Count	P_Value	Benjamini
SMART	ARF	RT	3	2.2E-1	9.2E-1
GOTERM_BP_FAT	small GTPase mediated signal transduction	RT	9	3.1E-1	9.9E-1
INTERPRO	ARF/SAR superfamily	RT	4	3.3E-1	1.0E0
INTERPRO	ADP-ribosylation factor	RT	3	3.3E-1	1.0E0
PIR_SUPERFAMILY	PIRSF001711:ADP-ribosylation factor	RT	4	6.0E-1	1.0E0
GOTERM_BP_FAT	intracellular signaling cascade	RT	9	6.8E-1	1.0E0
Annotation Cluster 30	Enrichment Score: 0.35		Count	P_Value	Benjamini
SMART	PP2Ac	RT	7	9.6E-2	7.3E-1
INTERPRO	Serine/threonine-specific protein phosphatase and bis(5-nucleosyl)-tetraphosphatase	RT	7	2.5E-1	1.0E0
SP_PIR_KEYWORDS	iron	RT	8	5.3E-1	8.9E-1
GOTERM_MF_FAT	iron ion binding	RT	12	5.8E-1	1.0E0
INTERPRO	Metallophosphoesterase	RT	7	6.0E-1	1.0E0
GOTERM_MF_FAT	phosphatase activity	RT	16	6.5E-1	1.0E0

GOTERM_MF_FAT	phosphoprotein phosphatase activity	RT	10	7.2E-1	1.0E0
PIR_SUPERFAMILY	PIRSF000908:serine/threonine-protein phosphatase, conventional type	RT	5	7.5E-1	1.0E0
Annotation Cluster 31	Enrichment Score: 0.35		Count	P_Value	Benjamini
GOTERM_BP_FAT	ribonucleoside metabolic process	RT	5	3.0E-1	9.9E-1
GOTERM_BP_FAT	pyrimidine nucleoside metabolic process	RT	3	3.6E-1	9.9E-1
GOTERM_BP_FAT	pyrimidine ribonucleoside metabolic process	RT	3	3.6E-1	9.9E-1
KEGG_PATHWAY	Pyrimidine metabolism	RT	7	1.0E0	1.0E0
Annotation Cluster 32	Enrichment Score: 0.34		Count	P_Value	Benjamini
INTERPRO	ABC transporter-like	RT	10	2.0E-1	1.0E0
INTERPRO	ABC transporter, conserved site	RT	7	6.5E-1	1.0E0
COG_ONTOLOGY	Defense mechanisms	RT	7	7.4E-1	9.3E-1
Annotation Cluster 33	Enrichment Score: 0.28		Count	P_Value	Benjamini
SMART	Kelch	RT	3	4.0E-1	9.9E-1
INTERPRO	Kelch repeat type 1	RT	3	5.6E-1	1.0E0
INTERPRO	Kelch-type beta propeller	RT	3	6.5E-1	1.0E0
Annotation Cluster 34	Enrichment Score: 0.25		Count	P_Value	Benjamini
SMART	TPR	RT	8	2.8E-1	9.5E-1
INTERPRO	Tetratricopeptide-like helical	RT	10	5.7E-1	1.0E0
INTERPRO	Tetratricopeptide repeat	RT	8	5.7E-1	1.0E0
SP_PIR_KEYWORDS	tpr repeat	RT	4	6.3E-1	9.3E-1
INTERPRO	Tetratricopeptide TPR-1	RT	6	7.3E-1	1.0E0
INTERPRO	Tetratricopeptide region	RT	7	7.4E-1	1.0E0
Annotation Cluster 35	Enrichment Score: 0.22		Count	P_Value	Benjamini
GOTERM_MF_FAT	nucleobase, nucleoside, nucleotide kinase activity	RT	6	2.7E-1	9.7E-1
GOTERM_MF_FAT	phosphotransferase activity, phosphate group as acceptor	RT	4	4.9E-1	1.0E0
KEGG_PATHWAY	Pyrimidine metabolism	RT	7	1.0E0	1.0E0
KEGG_PATHWAY	Purine metabolism	RT	6	1.0E0	1.0E0
Annotation Cluster 36	Enrichment Score: 0.19		Count	P_Value	Benjamini
GOTERM_BP_FAT	pyridine nucleotide metabolic process	RT	4	4.7E-1	1.0E0
GOTERM_BP_FAT	oxidoreduction coenzyme metabolic process	RT	4	4.7E-1	1.0E0
GOTERM_BP_FAT	nicotinamide metabolic process	RT	3	6.8E-1	1.0E0
GOTERM_BP_FAT	secondary metabolic process	RT	3	6.8E-1	1.0E0
GOTERM_BP_FAT	nicotinamide nucleotide metabolic process	RT	3	6.8E-1	1.0E0
GOTERM_BP_FAT	alkaloid metabolic process	RT	3	6.8E-1	1.0E0
GOTERM_BP_FAT	cellular amide metabolic process	RT	3	6.8E-1	1.0E0
GOTERM_BP_FAT	cofactor metabolic process	RT	5	7.9E-1	1.0E0
GOTERM_BP_FAT	coenzyme metabolic process	RT	4	8.5E-1	1.0E0
Annotation Cluster 37	Enrichment Score: 0.16		Count	P_Value	Benjamini
SP_PIR_KEYWORDS	Acyltransferase	RT	5	5.3E-1	8.9E-1
GOTERM_MF_FAT	N-acyltransferase activity	RT	4	7.6E-1	1.0E0
INTERPRO	Acyl-CoA N-acyltransferase	RT	3	8.4E-1	1.0E0
Annotation Cluster 38	Enrichment Score: 0.14		Count	P_Value	Benjamini
SMART	EFh	RT	4	4.6E-1	9.9E-1
INTERPRO	Calcium-binding EF-hand	RT	4	6.6E-1	1.0E0
INTERPRO	EF hand	RT	4	6.6E-1	1.0E0
INTERPRO	EF-HAND 1	RT	6	7.3E-1	1.0E0
INTERPRO	EF-Hand type	RT	5	8.1E-1	1.0E0
INTERPRO	EF-HAND 2	RT	4	9.1E-1	1.0E0
SP_PIR_KEYWORDS	calcium	RT	4	9.2E-1	1.0E0
Annotation Cluster 39	Enrichment Score: 0.12		Count	P_Value	Benjamini
GOTERM_MF_FAT	GTPase activity	RT	9	2.3E-1	9.6E-1
INTERPRO	Tubulin/FtsZ, 2-layer sandwich domain	RT	3	6.5E-1	1.0E0
INTERPRO	Tubulin, conserved site	RT	3	7.3E-1	1.0E0
INTERPRO	Tubulin	RT	3	7.3E-1	1.0E0
INTERPRO	Tubulin/FtsZ, GTPase domain	RT	3	7.3E-1	1.0E0
SP_PIR_KEYWORDS	microtubule	RT	9	8.9E-1	9.9E-1
GOTERM_BP_FAT	cellular protein complex assembly	RT	4	8.9E-1	1.0E0
GOTERM_BP_FAT	protein polymerization	RT	3	9.0E-1	1.0E0

GOTERM_BP_FAT	protein complex assembly	RT	4	9.5E-1	1.0E0
GOTERM_BP_FAT	protein complex biogenesis	RT	4	9.5E-1	1.0E0
PIR_SUPERFAMILY	PIRSF002306:tubulin	RT	3	9.7E-1	1.0E0
GOTERM_CC_FAT	microtubule	RT	9	1.0E0	1.0E0
Annotation Cluster 40	Enrichment Score: 0.11		Count	P_Value	Benjamini
GOTERM_BP_FAT	nucleobase, nucleoside and nucleotide catabolic process	RT	3	7.8E-1	1.0E0
GOTERM_BP_FAT	nucleobase, nucleoside, nucleotide and nucleic acid catabolic process	RT	3	7.8E-1	1.0E0
GOTERM_BP_FAT	nitrogen compound catabolic process	RT	3	7.8E-1	1.0E0
Annotation Cluster 41	Enrichment Score: 0.09		Count	P_Value	Benjamini
INTERPRO	Dynein heavy chain	RT	6	2.5E-1	1.0E0
INTERPRO	Kinesin, motor region, conserved site	RT	6	6.4E-1	1.0E0
GOTERM_CC_FAT	microtubule associated complex	RT	8	6.7E-1	1.0E0
SMART	KISc	RT	6	7.9E-1	1.0E0
GOTERM_CC_FAT	dynein complex	RT	6	7.9E-1	1.0E0
GOTERM_MF_FAT	microtubule motor activity	RT	14	8.0E-1	1.0E0
GOTERM_MF_FAT	motor activity	RT	14	8.0E-1	1.0E0
SP_PIR_KEYWORDS	microtubule	RT	9	8.9E-1	9.9E-1
INTERPRO	Kinesin, motor region	RT	6	9.4E-1	1.0E0
SP_PIR_KEYWORDS	motor protein	RT	6	9.4E-1	1.0E0
GOTERM_BP_FAT	microtubule-based process	RT	17	9.7E-1	1.0E0
GOTERM_BP_FAT	microtubule-based movement	RT	14	9.8E-1	1.0E0
GOTERM_CC_FAT	cytoskeleton	RT	19	1.0E0	1.0E0
GOTERM_CC_FAT	microtubule cytoskeleton	RT	17	1.0E0	1.0E0
GOTERM_CC_FAT	cytoskeletal part	RT	17	1.0E0	1.0E0
GOTERM_CC_FAT	microtubule	RT	9	1.0E0	1.0E0
Annotation Cluster 42	Enrichment Score: 0.08		Count	P_Value	Benjamini
GOTERM_MF_FAT	N-acyltransferase activity	RT	4	7.6E-1	1.0E0
INTERPRO	GCN5-related N-acetyltransferase	RT	3	7.9E-1	1.0E0
GOTERM_MF_FAT	N-acetyltransferase activity	RT	3	8.9E-1	1.0E0
GOTERM_MF_FAT	acetyltransferase activity	RT	3	9.2E-1	1.0E0
Annotation Cluster 43	Enrichment Score: 0.07		Count	P_Value	Benjamini
INTERPRO	Clathrin/coatomer adaptor, adaptin-like, N-terminal	RT	3	4.5E-1	1.0E0
GOTERM_CC_FAT	coated membrane	RT	7	7.7E-1	1.0E0
GOTERM_CC_FAT	membrane coat	RT	7	7.7E-1	1.0E0
GOTERM_CC_FAT	Golgi apparatus	RT	5	8.8E-1	1.0E0
GOTERM_CC_FAT	organelle membrane	RT	5	8.8E-1	1.0E0
GOTERM_CC_FAT	vesicle membrane	RT	3	8.8E-1	1.0E0
GOTERM_CC_FAT	cytoplasmic membrane-bounded vesicle	RT	3	8.8E-1	1.0E0
GOTERM_CC_FAT	cytoplasmic vesicle part	RT	3	8.8E-1	1.0E0
GOTERM_CC_FAT	vesicle	RT	3	8.8E-1	1.0E0
GOTERM_CC_FAT	membrane-bounded vesicle	RT	3	8.8E-1	1.0E0
GOTERM_CC_FAT	cytoplasmic vesicle	RT	3	8.8E-1	1.0E0
GOTERM_CC_FAT	coated vesicle	RT	3	8.8E-1	1.0E0
GOTERM_CC_FAT	vesicle coat	RT	3	8.8E-1	1.0E0
GOTERM_CC_FAT	coated vesicle membrane	RT	3	8.8E-1	1.0E0
GOTERM_CC_FAT	cytoplasmic vesicle membrane	RT	3	8.8E-1	1.0E0
GOTERM_CC_FAT	endomembrane system	RT	6	9.6E-1	1.0E0
GOTERM_CC_FAT	Golgi apparatus part	RT	3	9.6E-1	1.0E0
Annotation Cluster 44	Enrichment Score: 0.07		Count	P_Value	Benjamini
SP_PIR_KEYWORDS	membrane	RT	10	5.8E-1	9.1E-1
SP_PIR_KEYWORDS	transmembrane	RT	8	9.2E-1	1.0E0
GOTERM_CC_FAT	intrinsic to membrane	RT	15	1.0E0	1.0E0
GOTERM_CC_FAT	integral to membrane	RT	15	1.0E0	1.0E0
Annotation Cluster 45	Enrichment Score: 0.06		Count	P_Value	Benjamini
GOTERM_BP_FAT	cellular macromolecular complex subunit organization	RT	11	6.1E-1	1.0E0
INTERPRO	Histone core	RT	4	6.6E-1	1.0E0
GOTERM_BP_FAT	cellular macromolecular complex assembly	RT	10	7.0E-1	1.0E0

	GOTERM_BP_FAT	macromolecular complex subunit organization	RT			11	7.2E-1	1.0E0
	SP_PIR_KEYWORDS	nucleosome core	RT			3	7.4E-1	9.7E-1
	SP_PIR_KEYWORDS	chromosomal protein	RT			4	7.7E-1	9.7E-1
	GOTERM_BP_FAT	macromolecular complex assembly	RT			10	8.0E-1	1.0E0
	INTERPRO	Histone-fold	RT			4	8.2E-1	1.0E0
	SP_PIR_KEYWORDS	nucleus	RT			10	8.6E-1	9.9E-1
	GOTERM_BP_FAT	DNA packaging	RT			4	8.9E-1	1.0E0
	GOTERM_BP_FAT	nucleosome assembly	RT			4	8.9E-1	1.0E0
	GOTERM_BP_FAT	protein-DNA complex assembly	RT			4	8.9E-1	1.0E0
	GOTERM_BP_FAT	chromatin assembly	RT			4	8.9E-1	1.0E0
	GOTERM_BP_FAT	nucleosome organization	RT			4	8.9E-1	1.0E0
	GOTERM_BP_FAT	chromatin assembly or disassembly	RT			4	9.5E-1	1.0E0
	GOTERM_CC_FAT	nucleosome	RT			4	9.6E-1	1.0E0
	GOTERM_BP_FAT	chromatin organization	RT			4	9.8E-1	1.0E0
	GOTERM_CC_FAT	chromatin	RT			4	9.8E-1	1.0E0
	GOTERM_CC_FAT	protein-DNA complex	RT			4	9.9E-1	1.0E0
	GOTERM_BP_FAT	chromosome organization	RT			4	1.0E0	1.0E0
	GOTERM_CC_FAT	chromosomal part	RT			4	1.0E0	1.0E0
	SP_PIR_KEYWORDS	dna-binding	RT			5	1.0E0	1.0E0
	GOTERM_CC_FAT	chromosome	RT			4	1.0E0	1.0E0
	GOTERM_MF_FAT	DNA binding	RT			7	1.0E0	1.0E0
	Annotation Cluster 46	Enrichment Score: 0.06	G			Count	P_Value	Benjamini
	INTERPRO	EGF	RT			3	6.5E-1	1.0E0
	SP_PIR_KEYWORDS	egf-like domain	RT			6	7.2E-1	9.6E-1
	INTERPRO	EGF, extracellular	RT			6	8.4E-1	1.0E0
	INTERPRO	EGF-like, type 3	RT			7	9.4E-1	1.0E0
	INTERPRO	EGF-like region, conserved site	RT			16	1.0E0	1.0E0
	SMART	EGF	RT			20	1.0E0	1.0E0
	INTERPRO	EGF-like	RT			20	1.0E0	1.0E0
	Annotation Cluster 47	Enrichment Score: 0.06	G			Count	P_Value	Benjamini
	GOTERM_BP_FAT	tRNA modification	RT			3	5.4E-1	1.0E0
	GOTERM_BP_FAT	RNA modification	RT			5	9.4E-1	1.0E0
	GOTERM_BP_FAT	tRNA processing	RT			4	9.9E-1	1.0E0
	GOTERM_BP_FAT	ncRNA processing	RT			7	9.9E-1	1.0E0
	GOTERM_BP_FAT	RNA processing	RT			12	1.0E0	1.0E0
	Annotation Cluster 48	Enrichment Score: 0.05	G			Count	P_Value	Benjamini
	SMART	Pept_C1	RT			6	7.6E-1	1.0E0
	INTERPRO	Peptidase, cysteine peptidase active site	RT			8	8.1E-1	1.0E0
	INTERPRO	Peptidase C1A, papain	RT			6	9.3E-1	1.0E0
	INTERPRO	Peptidase C1A, papain C-terminal	RT			6	9.3E-1	1.0E0
	GOTERM_MF_FAT	cysteine-type endopeptidase activity	RT			6	9.7E-1	1.0E0
	GOTERM_MF_FAT	cysteine-type peptidase activity	RT			6	9.9E-1	1.0E0
	Annotation Cluster 49	Enrichment Score: 0.03	G			Count	P_Value	Benjamini
	GOTERM_BP_FAT	ribosome biogenesis	RT			7	8.9E-1	1.0E0
	GOTERM_BP_FAT	ribonucleoprotein complex biogenesis	RT			7	8.9E-1	1.0E0
	GOTERM_BP_FAT	rRNA metabolic process	RT			3	9.9E-1	1.0E0
	GOTERM_BP_FAT	rRNA processing	RT			3	9.9E-1	1.0E0
	Annotation Cluster 50	Enrichment Score: 0.02	G			Count	P_Value	Benjamini
	INTERPRO	RNA helicase, DEAD-box type, Q motif	RT			5	7.7E-1	1.0E0
	INTERPRO	DNA/RNA helicase, DEAD/DEAH box type, N-terminal	RT			7	9.1E-1	1.0E0
	SMART	DEXDc	RT			7	9.4E-1	1.0E0
	SMART	HELICc	RT			7	9.5E-1	1.0E0
	GOTERM_MF_FAT	ATPase activity, coupled	RT			19	9.8E-1	1.0E0
	INTERPRO	DEAD-like helicase, N-terminal	RT			7	9.9E-1	1.0E0
	INTERPRO	Helicase, superfamily 1 and 2, ATP-binding	RT			7	9.9E-1	1.0E0
	INTERPRO	DNA/RNA helicase, C-terminal	RT			7	1.0E0	1.0E0
	GOTERM_MF_FAT	purine NTP-dependent helicase activity	RT			7	1.0E0	1.0E0
		ATP-dependent helicase activity	RT			7	1.0E0	1.0E0

Category	Term		Count	P_Value	Benjamini
GOTERM_MF_FAT					
SP_PIR_KEYWORDS	helicase	RT	7	1.0E0	1.0E0
GOTERM_MF_FAT	helicase activity	RT	9	1.0E0	1.0E0
Annotation Cluster 51	Enrichment Score: 0.02	G	Count	P_Value	Benjamini
INTERPRO	WD40 repeat, conserved site	RT	7	8.9E-1	1.0E0
SMART	WD40	RT	13	9.3E-1	1.0E0
INTERPRO	WD40 repeat 2	RT	9	9.6E-1	1.0E0
INTERPRO	WD40 repeat, region	RT	9	9.8E-1	1.0E0
INTERPRO	WD40 repeat, subgroup	RT	11	9.8E-1	1.0E0
SP_PIR_KEYWORDS	wd repeat	RT	10	9.9E-1	1.0E0
INTERPRO	WD40/YVTN repeat-like	RT	13	1.0E0	1.0E0
INTERPRO	WD40 repeat	RT	13	1.0E0	1.0E0
Annotation Cluster 52	Enrichment Score: 0	G	Count	P_Value	Benjamini
SMART	ANK	RT	85	9.7E-1	1.0E0
INTERPRO	Ankyrin	RT	85	1.0E0	1.0E0
SP_PIR_KEYWORDS	ank repeat	RT	62	1.0E0	1.0E0
Annotation Cluster 53	Enrichment Score: 0	G	Count	P_Value	Benjamini
GOTERM_MF_FAT	ion binding	RT	74	1.0E0	1.0E0
GOTERM_MF_FAT	cation binding	RT	74	1.0E0	1.0E0
GOTERM_MF_FAT	metal ion binding	RT	71	1.0E0	1.0E0
GOTERM_MF_FAT	transition metal ion binding	RT	34	1.0E0	1.0E0
Annotation Cluster 54	Enrichment Score: 0	G	Count	P_Value	Benjamini
GOTERM_BP_FAT	cellular response to stress	RT	4	1.0E0	1.0E0
GOTERM_BP_FAT	DNA repair	RT	3	1.0E0	1.0E0
GOTERM_BP_FAT	response to DNA damage stimulus	RT	3	1.0E0	1.0E0
GOTERM_BP_FAT	DNA metabolic process	RT	4	1.0E0	1.0E0
Annotation Cluster 55	Enrichment Score: 0	G	Count	P_Value	Benjamini
KEGG_PATHWAY	Mismatch repair	RT	3	1.0E0	1.0E0
KEGG_PATHWAY	Nucleotide excision repair	RT	3	1.0E0	1.0E0
KEGG_PATHWAY	DNA replication	RT	3	1.0E0	1.0E0
Annotation Cluster 56	Enrichment Score: 0	G	Count	P_Value	Benjamini
SMART	EGF	RT	20	1.0E0	1.0E0
SMART	FU	RT	13	1.0E0	1.0E0
INTERPRO	EGF-like	RT	20	1.0E0	1.0E0
INTERPRO	Furin-like repeat	RT	13	1.0E0	1.0E0
INTERPRO	Giardia variant-specific surface protein	RT	11	1.0E0	1.0E0

128 terms were not clustered.

Please cite *Nature Protocols 2009; 4(1):44 & Genome Biology 2003; 4(5):P3* within any publication that makes use of any methods inspired by **DAVID**.

SAIC Frederick A Division of Science Applications International Corporation · NIAID · ABCC · NCI-Frederick · National Institutes of Health · FirstGov Your First Click to the U.S. Government · Department of Health and Human Services

Term of Service | Contact Us | Site Map
