# Supplementary material for: Proteomics of Secretory and Endocytic Organelles in Giardia lamblia
Source: PLoS One. 2014 Apr 14;9(4):e94089. doi: 10.1371/journal.pone.0094089 (PMC3986054; doi:10.1371/journal.pone.0094089)
Supplement: Text S3 — DAVID functional annotation clustering of the G. lamblia genome. Functional annotation clustering of the G. lamblia genome using the DAVID bioinformatics tool (http://david.abcc.ncifcrf.gov/home.jsp). For each cluster, the enrichment score is given at the top, and functional groups (left) and protein counts (right) within the respective cluster are listed. Clustering of all predicted proteins in the G. lamblia genome (5150 validated genes) using 10 random gene lists containing 3′000 genes each. (PDF) [file pone.0094089.s011.pdf]

DAVID Bioinformatics Resources 6.7
National Institute of Allergy and Infectious Diseases (NIAID), NIH

Functional Annotation Clustering

Help and Manual

Current Gene List: 3_Random1
Current Background: Giardia intestinalis
3000 DAVID IDs

Options   Classification Stringency   Medium
Rerun using options   Create Sublist

73 Cluster(s)   Download File

Category	Term		Count	P_Value	Benjamini
Annotation Cluster 1	Enrichment Score: 0.91		Count	P_Value	Benjamini
SP_PIR_KEYWORDS	ank repeat	RT	192	8.8E-2	1.0E0
INTERPRO	Ankyrin	RT	265	1.2E-1	1.0E0
SMART	ANK	RT	265	1.7E-1	1.0E0
Annotation Cluster 2	Enrichment Score: 0.62		Count	P_Value	Benjamini
GOTERM_MF_FAT	protein serine/threonine kinase activity	RT	72	1.3E-2	1.0E0
INTERPRO	Serine/threonine protein kinase, active site	RT	60	7.0E-2	1.0E0
GOTERM_MF_FAT	purine nucleotide binding	RT	344	1.2E-1	1.0E0
GOTERM_MF_FAT	ribonucleotide binding	RT	339	1.5E-1	1.0E0
GOTERM_MF_FAT	purine ribonucleotide binding	RT	339	1.5E-1	1.0E0
GOTERM_MF_FAT	adenyl nucleotide binding	RT	313	2.1E-1	1.0E0
GOTERM_MF_FAT	purine nucleoside binding	RT	313	2.1E-1	1.0E0
GOTERM_MF_FAT	nucleotide binding	RT	361	2.3E-1	1.0E0
GOTERM_MF_FAT	adenyl ribonucleotide binding	RT	308	2.4E-1	1.0E0
GOTERM_MF_FAT	ATP binding	RT	308	2.4E-1	1.0E0
GOTERM_MF_FAT	nucleoside binding	RT	314	2.5E-1	1.0E0
SP_PIR_KEYWORDS	kinase	RT	200	2.7E-1	1.0E0
GOTERM_BP_FAT	protein amino acid phosphorylation	RT	157	2.7E-1	1.0E0
GOTERM_BP_FAT	phosphorylation	RT	167	3.2E-1	1.0E0
INTERPRO	Serine/threonine protein kinase	RT	37	3.2E-1	1.0E0
GOTERM_BP_FAT	phosphorus metabolic process	RT	176	3.2E-1	1.0E0
GOTERM_BP_FAT	phosphate metabolic process	RT	176	3.2E-1	1.0E0
INTERPRO	Protein kinase, ATP binding site	RT	51	3.4E-1	1.0E0
GOTERM_MF_FAT	protein kinase activity	RT	159	3.5E-1	1.0E0
SMART	S_TKc	RT	37	3.5E-1	1.0E0
INTERPRO	Serine/threonine protein kinase-related	RT	139	3.7E-1	1.0E0
SP_PIR_KEYWORDS	atp-binding	RT	154	4.3E-1	1.0E0
INTERPRO	Protein kinase, core	RT	156	4.9E-1	1.0E0
SP_PIR_KEYWORDS	serine/threonine-protein kinase	RT	37	5.6E-1	1.0E0
SP_PIR_KEYWORDS	nucleotide-binding	RT	158	6.2E-1	1.0E0
Annotation Cluster 3	Enrichment Score: 0.6		Count	P_Value	Benjamini
GOTERM_BP_FAT	hexose catabolic process	RT	11	2.0E-1	1.0E0
GOTERM_BP_FAT	carbohydrate catabolic process	RT	11	2.0E-1	1.0E0
GOTERM_BP_FAT	monosaccharide catabolic process	RT	11	2.0E-1	1.0E0
GOTERM_BP_FAT	alcohol catabolic process	RT	11	2.0E-1	1.0E0
GOTERM_BP_FAT	cellular carbohydrate catabolic process	RT	11	2.0E-1	1.0E0
GOTERM_BP_FAT	glucose catabolic process	RT	11	2.0E-1	1.0E0
GOTERM_BP_FAT	glycolysis	RT	9	2.2E-1	1.0E0
GOTERM_BP_FAT	monosaccharide metabolic process	RT	16	2.7E-1	1.0E0
GOTERM_BP_FAT	hexose metabolic process	RT	14	3.0E-1	1.0E0
GOTERM_BP_FAT	glucose metabolic process	RT	14	3.0E-1	1.0E0
GOTERM_BP_FAT	generation of precursor metabolites and energy	RT	17	3.8E-1	1.0E0
SP_PIR_KEYWORDS	glycolysis	RT	5	5.2E-1	1.0E0
Annotation Cluster 4	Enrichment Score: 0.59		Count	P_Value	Benjamini
GOTERM_MF_FAT	ion binding	RT	158	1.1E-1	1.0E0
GOTERM_MF_FAT	cation binding	RT	158	1.1E-1	1.0E0

GOTERM_MF_FAT	metal ion binding	RT			156	1.2E-1	1.0E0
GOTERM_MF_FAT	transition metal ion binding	RT			115	1.7E-1	1.0E0
GOTERM_MF_FAT	zinc ion binding	RT			92	2.6E-1	1.0E0
INTERPRO	Zinc finger, RING-type	RT			43	2.7E-1	1.0E0
SMART	RING	RT			43	3.0E-1	1.0E0
SP_PIR_KEYWORDS	zinc-finger	RT			39	3.5E-1	1.0E0
INTERPRO	Zinc finger, C3HC4 RING-type	RT			22	4.9E-1	1.0E0
SP_PIR_KEYWORDS	zinc	RT			50	6.0E-1	1.0E0
SP_PIR_KEYWORDS	metal-binding	RT			59	6.3E-1	9.9E-1
Annotation Cluster 5	Enrichment Score: 0.54	G			Count	P_Value	Benjamini
INTERPRO	ARF/SAR superfamily	RT			7	1.4E-1	1.0E0
PIR_SUPERFAMILY	PIRSF001711:ADP-ribosylation factor	RT			5	2.4E-1	1.0E0
INTERPRO	ADP-ribosylation factor	RT			4	4.4E-1	1.0E0
SMART	ARF	RT			4	4.5E-1	1.0E0
Annotation Cluster 6	Enrichment Score: 0.49	G			Count	P_Value	Benjamini
INTERPRO	Zinc finger, C2H2-like	RT			13	3.2E-1	1.0E0
INTERPRO	Zinc finger, C2H2-type	RT			13	3.2E-1	1.0E0
SMART	ZnF_C2H2	RT			13	3.3E-1	1.0E0
Annotation Cluster 7	Enrichment Score: 0.47	G			Count	P_Value	Benjamini
INTERPRO	EGF-like	RT			118	1.8E-1	1.0E0
SMART	EGF	RT			118	2.3E-1	1.0E0
INTERPRO	Giardia variant-specific surface protein	RT			140	4.4E-1	1.0E0
INTERPRO	Furin-like repeat	RT			109	4.8E-1	1.0E0
SMART	FU	RT			109	5.5E-1	1.0E0
Annotation Cluster 8	Enrichment Score: 0.45	G			Count	P_Value	Benjamini
GOTERM_CC_FAT	endomembrane system	RT			12	2.4E-1	1.0E0
GOTERM_CC_FAT	organelle envelope	RT			5	2.8E-1	1.0E0
GOTERM_CC_FAT	envelope	RT			5	2.8E-1	1.0E0
GOTERM_CC_FAT	nuclear pore	RT			5	2.8E-1	1.0E0
GOTERM_CC_FAT	nuclear envelope	RT			5	2.8E-1	1.0E0
GOTERM_CC_FAT	pore complex	RT			5	2.8E-1	1.0E0
GOTERM_BP_FAT	nuclear transport	RT			3	7.8E-1	1.0E0
GOTERM_BP_FAT	nucleocytoplasmic transport	RT			3	7.8E-1	1.0E0
Annotation Cluster 9	Enrichment Score: 0.42	G			Count	P_Value	Benjamini
INTERPRO	Small GTP-binding protein	RT			15	2.0E-1	1.0E0
GOTERM_BP_FAT	small GTPase mediated signal transduction	RT			12	2.4E-1	1.0E0
GOTERM_MF_FAT	GTP binding	RT			31	2.9E-1	1.0E0
GOTERM_MF_FAT	guanyl nucleotide binding	RT			31	2.9E-1	1.0E0
GOTERM_MF_FAT	guanyl ribonucleotide binding	RT			31	2.9E-1	1.0E0
GOTERM_BP_FAT	intracellular signaling cascade	RT			14	4.0E-1	1.0E0
INTERPRO	Ras GTPase	RT			9	5.2E-1	1.0E0
INTERPRO	Ras	RT			8	6.2E-1	1.0E0
SP_PIR_KEYWORDS	gtp-binding	RT			24	6.4E-1	9.9E-1
PIR_SUPERFAMILY	PIRSF001710:Ras-related protein Rab	RT			4	7.3E-1	1.0E0
Annotation Cluster 10	Enrichment Score: 0.39	G			Count	P_Value	Benjamini
GOTERM_BP_FAT	mRNA metabolic process	RT			6	1.8E-1	1.0E0
GOTERM_BP_FAT	mRNA processing	RT			5	2.8E-1	1.0E0
GOTERM_BP_FAT	mRNA capping	RT			3	6.0E-1	1.0E0
GOTERM_BP_FAT	RNA capping	RT			3	8.9E-1	1.0E0
Annotation Cluster 11	Enrichment Score: 0.39	G			Count	P_Value	Benjamini
INTERPRO	Metallophosphoesterase	RT			16	1.6E-1	1.0E0
GOTERM_MF_FAT	phosphatase activity	RT			28	3.6E-1	1.0E0
GOTERM_MF_FAT	iron ion binding	RT			20	4.1E-1	1.0E0
GOTERM_MF_FAT	phosphoprotein phosphatase activity	RT			18	4.4E-1	1.0E0
PIR_SUPERFAMILY	PIRSF000908:serine/threonine-protein phosphatase, conventional type	RT			6	4.6E-1	1.0E0
INTERPRO	Serine/threonine-specific protein phosphatase and bis(5-nucleosyl)-tetraphosphatase	RT			10	5.5E-1	1.0E0
SP_PIR_KEYWORDS	iron	RT			13	5.6E-1	1.0E0
	PP2Ac	RT			10	5.7E-1	1.0E0

SMART					
Annotation Cluster 12	Enrichment Score: 0.38		Count	P_Value	Benjamini
INTERPRO	EGF, extracellular	RT	17	1.9E-1	1.0E0
SP_PIR_KEYWORDS	egf-like domain	RT	11	6.1E-1	1.0E0
INTERPRO	EGF-like, type 3	RT	19	6.6E-1	1.0E0
Annotation Cluster 13	Enrichment Score: 0.37		Count	P_Value	Benjamini
GOTERM_BP_FAT	establishment of protein localization	RT	22	2.6E-1	1.0E0
GOTERM_BP_FAT	protein transport	RT	22	2.6E-1	1.0E0
GOTERM_BP_FAT	cellular macromolecule localization	RT	16	2.7E-1	1.0E0
GOTERM_BP_FAT	cellular protein localization	RT	16	2.7E-1	1.0E0
GOTERM_BP_FAT	intracellular protein transport	RT	16	2.7E-1	1.0E0
GOTERM_BP_FAT	protein localization	RT	22	3.3E-1	1.0E0
GOTERM_BP_FAT	intracellular transport	RT	18	4.1E-1	1.0E0
GOTERM_CC_FAT	membrane coat	RT	9	6.1E-1	1.0E0
GOTERM_CC_FAT	coated membrane	RT	9	6.1E-1	1.0E0
GOTERM_CC_FAT	Golgi apparatus	RT	7	6.9E-1	1.0E0
GOTERM_BP_FAT	vesicle-mediated transport	RT	15	8.7E-1	1.0E0
INTERPRO	Clathrin/coatomer adaptor, adaptin-like, N-terminal	RT	3	9.0E-1	1.0E0
Annotation Cluster 14	Enrichment Score: 0.36		Count	P_Value	Benjamini
INTERPRO	DNA/RNA helicase, DEAD/DEAH box type, N-terminal	RT	21	2.3E-1	1.0E0
GOTERM_MF_FAT	purine NTP-dependent helicase activity	RT	25	2.9E-1	1.0E0
GOTERM_MF_FAT	ATP-dependent helicase activity	RT	25	2.9E-1	1.0E0
GOTERM_MF_FAT	ATPase activity	RT	58	3.0E-1	1.0E0
INTERPRO	DNA/RNA helicase, C-terminal	RT	28	3.9E-1	1.0E0
SMART	HELICc	RT	28	4.3E-1	1.0E0
INTERPRO	Helicase, superfamily 1 and 2, ATP-binding	RT	27	4.4E-1	1.0E0
GOTERM_MF_FAT	ATPase activity, coupled	RT	42	4.8E-1	1.0E0
SP_PIR_KEYWORDS	helicase	RT	27	5.6E-1	1.0E0
INTERPRO	DEAD-like helicase, N-terminal	RT	26	5.7E-1	1.0E0
SMART	DEXDc	RT	26	6.0E-1	1.0E0
GOTERM_MF_FAT	helicase activity	RT	31	6.7E-1	1.0E0
INTERPRO	RNA helicase, DEAD-box type, Q motif	RT	10	7.6E-1	1.0E0
Annotation Cluster 15	Enrichment Score: 0.35		Count	P_Value	Benjamini
GOTERM_CC_FAT	endoplasmic reticulum	RT	6	1.9E-1	1.0E0
GOTERM_CC_FAT	organelle membrane	RT	8	4.5E-1	1.0E0
GOTERM_CC_FAT	nuclear envelope-endoplasmic reticulum network	RT	3	6.0E-1	1.0E0
GOTERM_CC_FAT	endoplasmic reticulum membrane	RT	3	6.0E-1	1.0E0
GOTERM_CC_FAT	endoplasmic reticulum part	RT	3	6.0E-1	1.0E0
Annotation Cluster 16	Enrichment Score: 0.33		Count	P_Value	Benjamini
GOTERM_CC_FAT	proteasome complex	RT	16	1.2E-1	1.0E0
GOTERM_CC_FAT	proteasome core complex	RT	11	2.0E-1	1.0E0
GOTERM_MF_FAT	threonine-type endopeptidase activity	RT	11	2.2E-1	1.0E0
GOTERM_MF_FAT	threonine-type peptidase activity	RT	11	2.2E-1	1.0E0
GOTERM_CC_FAT	cytosol	RT	20	2.2E-1	1.0E0
SP_PIR_KEYWORDS	proteasome	RT	15	2.2E-1	1.0E0
INTERPRO	Proteasome, subunit alpha/beta	RT	11	2.3E-1	1.0E0
KEGG_PATHWAY	Proteasome	RT	19	2.7E-1	1.0E0
INTERPRO	Proteasome, alpha and beta subunits	RT	10	3.0E-1	1.0E0
PIR_SUPERFAMILY	PIRSF001212:multicatalytic endopeptidase complex chain C9	RT	5	4.3E-1	1.0E0
INTERPRO	Proteasome, alpha-subunit, conserved site	RT	5	6.7E-1	1.0E0
GOTERM_BP_FAT	cellular macromolecule catabolic process	RT	19	7.3E-1	1.0E0
GOTERM_BP_FAT	proteolysis	RT	48	7.8E-1	1.0E0
GOTERM_BP_FAT	ubiquitin-dependent protein catabolic process	RT	8	7.8E-1	1.0E0
GOTERM_BP_FAT	cellular protein catabolic process	RT	18	7.8E-1	1.0E0
GOTERM_BP_FAT	proteolysis involved in cellular protein catabolic process	RT	18	7.8E-1	1.0E0
GOTERM_BP_FAT	macromolecule catabolic process	RT	21	8.0E-1	1.0E0
SP_PIR_KEYWORDS	threonine protease	RT	4	8.1E-1	9.9E-1

Category	Term		Count	P_Value	Benjamini
GOTERM_BP_FAT	protein catabolic process	RT	20	8.4E-1	1.0E0
GOTERM_BP_FAT	modification-dependent protein catabolic process	RT	12	9.5E-1	1.0E0
GOTERM_BP_FAT	modification-dependent macromolecule catabolic process	RT	12	9.5E-1	1.0E0
SP_PIR_KEYWORDS	ubl conjugation pathway	RT	4	1.0E0	1.0E0
Annotation Cluster 17	Enrichment Score: 0.33		Count	P_Value	Benjamini
INTERPRO	Kelch-type beta propeller	RT	6	3.8E-1	1.0E0
INTERPRO	Kelch repeat type 1	RT	5	5.1E-1	1.0E0
SMART	Kelch	RT	5	5.2E-1	1.0E0
Annotation Cluster 18	Enrichment Score: 0.32		Count	P_Value	Benjamini
GOTERM_MF_FAT	pyridoxal phosphate binding	RT	8	3.2E-1	1.0E0
GOTERM_MF_FAT	vitamin B6 binding	RT	8	3.2E-1	1.0E0
GOTERM_MF_FAT	vitamin binding	RT	8	4.6E-1	1.0E0
INTERPRO	Pyridoxal phosphate-dependent transferase, major region, subdomain 1	RT	6	5.5E-1	1.0E0
INTERPRO	Aminotransferase, class I and II	RT	4	6.5E-1	1.0E0
GOTERM_MF_FAT	transferase activity, transferring nitrogenous groups	RT	5	6.6E-1	1.0E0
Annotation Cluster 19	Enrichment Score: 0.28		Count	P_Value	Benjamini
GOTERM_CC_FAT	intrinsic to membrane	RT	40	4.0E-1	1.0E0
GOTERM_CC_FAT	integral to membrane	RT	40	4.0E-1	1.0E0
SP_PIR_KEYWORDS	membrane	RT	17	5.7E-1	1.0E0
SP_PIR_KEYWORDS	transmembrane	RT	17	8.0E-1	9.9E-1
Annotation Cluster 20	Enrichment Score: 0.27		Count	P_Value	Benjamini
GOTERM_BP_FAT	nitrogen compound catabolic process	RT	5	4.8E-1	1.0E0
GOTERM_BP_FAT	nucleobase, nucleoside and nucleotide catabolic process	RT	5	4.8E-1	1.0E0
GOTERM_BP_FAT	nucleobase, nucleoside, nucleotide and nucleic acid catabolic process	RT	5	4.8E-1	1.0E0
GOTERM_BP_FAT	nucleotide catabolic process	RT	3	7.8E-1	1.0E0
Annotation Cluster 21	Enrichment Score: 0.26		Count	P_Value	Benjamini
GOTERM_MF_FAT	nucleoside-triphosphatase regulator activity	RT	10	2.9E-1	1.0E0
GOTERM_BP_FAT	regulation of small GTPase mediated signal transduction	RT	7	4.0E-1	1.0E0
GOTERM_BP_FAT	regulation of Ras protein signal transduction	RT	7	4.0E-1	1.0E0
GOTERM_MF_FAT	enzyme activator activity	RT	7	4.2E-1	1.0E0
GOTERM_MF_FAT	GTPase regulator activity	RT	8	4.6E-1	1.0E0
GOTERM_MF_FAT	small GTPase regulator activity	RT	8	4.6E-1	1.0E0
GOTERM_BP_FAT	regulation of hydrolase activity	RT	6	5.2E-1	1.0E0
GOTERM_BP_FAT	regulation of GTPase activity	RT	6	5.2E-1	1.0E0
INTERPRO	RabGAP/TBC	RT	3	6.2E-1	1.0E0
GOTERM_BP_FAT	regulation of ARF protein signal transduction	RT	4	6.2E-1	1.0E0
SMART	TBC	RT	3	6.3E-1	1.0E0
GOTERM_BP_FAT	regulation of Ras GTPase activity	RT	5	6.4E-1	1.0E0
GOTERM_MF_FAT	GTPase activator activity	RT	5	6.6E-1	1.0E0
GOTERM_BP_FAT	regulation of Rab GTPase activity	RT	3	7.8E-1	1.0E0
GOTERM_BP_FAT	regulation of Rab protein signal transduction	RT	3	7.8E-1	1.0E0
GOTERM_MF_FAT	Ras GTPase activator activity	RT	3	7.9E-1	1.0E0
GOTERM_MF_FAT	Rab GTPase activator activity	RT	3	7.9E-1	1.0E0
Annotation Cluster 22	Enrichment Score: 0.24		Count	P_Value	Benjamini
GOTERM_BP_FAT	chromosome organization	RT	14	2.1E-1	1.0E0
GOTERM_BP_FAT	cellular macromolecular complex subunit organization	RT	17	3.0E-1	1.0E0
GOTERM_BP_FAT	cellular macromolecular complex assembly	RT	16	3.6E-1	1.0E0
INTERPRO	Histone-fold	RT	10	4.3E-1	1.0E0
GOTERM_BP_FAT	macromolecular complex subunit organization	RT	17	4.7E-1	1.0E0
GOTERM_BP_FAT	chromatin assembly or disassembly	RT	9	4.8E-1	1.0E0
GOTERM_BP_FAT	chromatin organization	RT	10	5.0E-1	1.0E0
GOTERM_BP_FAT	macromolecular complex assembly	RT	16	5.4E-1	1.0E0
PIR_SUPERFAMILY	PIRSF002052:histone H4	RT	3	5.6E-1	1.0E0
GOTERM_CC_FAT	chromatin	RT	8	5.8E-1	1.0E0
INTERPRO	Histone H4	RT	3	6.2E-1	1.0E0
SMART	H4	RT	3	6.3E-1	1.0E0

GOTERM_CC_FAT	chromosome	RT	16	6.4E-1	1.0E0
SP_PIR_KEYWORDS	dna-binding	RT	21	6.6E-1	9.9E-1
GOTERM_BP_FAT	protein-DNA complex assembly	RT	7	6.8E-1	1.0E0
GOTERM_BP_FAT	chromatin assembly	RT	7	6.8E-1	1.0E0
GOTERM_BP_FAT	nucleosome organization	RT	7	6.8E-1	1.0E0
GOTERM_BP_FAT	DNA packaging	RT	7	6.8E-1	1.0E0
GOTERM_BP_FAT	nucleosome assembly	RT	7	6.8E-1	1.0E0
GOTERM_CC_FAT	nucleosome	RT	7	6.9E-1	1.0E0
SP_PIR_KEYWORDS	nucleosome core	RT	5	6.9E-1	9.9E-1
INTERPRO	Histone core	RT	7	7.1E-1	1.0E0
SP_PIR_KEYWORDS	chromosomal protein	RT	7	7.3E-1	9.9E-1
SP_PIR_KEYWORDS	nucleus	RT	19	8.2E-1	9.9E-1
GOTERM_CC_FAT	protein-DNA complex	RT	7	8.6E-1	1.0E0
GOTERM_CC_FAT	chromosomal part	RT	9	8.6E-1	1.0E0
Annotation Cluster 23	Enrichment Score: 0.22		Count	P_Value	Benjamini
INTERPRO	Like-Sm ribonucleoprotein, core	RT	8	4.8E-1	1.0E0
INTERPRO	Like-Sm ribonucleoprotein, eukaryotic and archaea-type, core	RT	5	6.7E-1	1.0E0
SMART	Sm	RT	5	6.9E-1	1.0E0
Annotation Cluster 24	Enrichment Score: 0.21		Count	P_Value	Benjamini
KEGG_PATHWAY	Purine metabolism	RT	21	3.1E-1	1.0E0
KEGG_PATHWAY	RNA polymerase	RT	12	4.5E-1	1.0E0
KEGG_PATHWAY	Pyrimidine metabolism	RT	20	5.4E-1	1.0E0
GOTERM_MF_FAT	DNA-directed RNA polymerase activity	RT	14	6.2E-1	1.0E0
GOTERM_BP_FAT	transcription	RT	15	6.9E-1	1.0E0
SP_PIR_KEYWORDS	nucleotidyltransferase	RT	15	7.0E-1	9.9E-1
GOTERM_MF_FAT	RNA polymerase activity	RT	14	7.1E-1	1.0E0
SP_PIR_KEYWORDS	dna-directed rna polymerase	RT	8	8.3E-1	9.9E-1
GOTERM_MF_FAT	nucleotidyltransferase activity	RT	37	9.2E-1	1.0E0
Annotation Cluster 25	Enrichment Score: 0.2		Count	P_Value	Benjamini
GOTERM_BP_FAT	lipoprotein biosynthetic process	RT	4	6.2E-1	1.0E0
GOTERM_BP_FAT	lipoprotein metabolic process	RT	4	6.2E-1	1.0E0
GOTERM_BP_FAT	protein amino acid lipidation	RT	4	6.2E-1	1.0E0
Annotation Cluster 26	Enrichment Score: 0.2		Count	P_Value	Benjamini
GOTERM_MF_FAT	intramolecular transferase activity, phosphotransferases	RT	4	4.3E-1	1.0E0
INTERPRO	Alpha-D-phosphohexomutase, alpha/beta/alpha domain I	RT	3	6.2E-1	1.0E0
GOTERM_MF_FAT	intramolecular transferase activity	RT	5	9.6E-1	1.0E0
Annotation Cluster 27	Enrichment Score: 0.18		Count	P_Value	Benjamini
GOTERM_MF_FAT	electron carrier activity	RT	16	3.0E-1	1.0E0
INTERPRO	4Fe-4S ferredoxin, iron-sulphur binding, conserved site	RT	5	6.7E-1	1.0E0
INTERPRO	4Fe-4S ferredoxin, iron-sulpur binding domain	RT	6	6.9E-1	1.0E0
INTERPRO	4Fe-4S ferredoxin, iron-sulphur binding, subgroup	RT	6	6.9E-1	1.0E0
GOTERM_MF_FAT	4 iron, 4 sulfur cluster binding	RT	5	7.9E-1	1.0E0
GOTERM_MF_FAT	iron-sulfur cluster binding	RT	11	8.2E-1	1.0E0
GOTERM_MF_FAT	metal cluster binding	RT	11	8.2E-1	1.0E0
Annotation Cluster 28	Enrichment Score: 0.18		Count	P_Value	Benjamini
INTERPRO	RNA polymerase Rpb1, domain 3	RT	3	6.2E-1	1.0E0
INTERPRO	RNA polymerase, alpha subunit	RT	3	6.2E-1	1.0E0
INTERPRO	RNA polymerase, N-terminal	RT	3	6.2E-1	1.0E0
INTERPRO	RNA polymerase Rpb1, domain 4	RT	3	6.2E-1	1.0E0
INTERPRO	RNA polymerase Rpb1, domain 1	RT	3	6.2E-1	1.0E0
INTERPRO	RNA polymerase Rpb1, domain 5	RT	3	6.2E-1	1.0E0
SMART	RPOLA_N	RT	3	6.3E-1	1.0E0
SP_PIR_KEYWORDS	Transcription	RT	5	8.1E-1	9.9E-1
SP_PIR_KEYWORDS	dna-directed rna polymerase	RT	8	8.3E-1	9.9E-1
Annotation Cluster 29	Enrichment Score: 0.17		Count	P_Value	Benjamini
GOTERM_BP_FAT	one-carbon metabolic process	RT	8	4.4E-1	1.0E0
INTERPRO	Ribosomal RNA methyltransferase RrmJ/FtsJ	RT	4	4.4E-1	1.0E0

GOTERM_BP_FAT	methylation	RT	7	5.5E-1	1.0E0
GOTERM_BP_FAT	RNA methylation	RT	3	8.9E-1	1.0E0
GOTERM_BP_FAT	biopolymer methylation	RT	3	9.4E-1	1.0E0
GOTERM_BP_FAT	RNA modification	RT	5	1.0E0	1.0E0
Annotation Cluster 30	Enrichment Score: 0.17		Count	P_Value	Benjamini
SP_PIR_KEYWORDS	Initiation factor	RT	12	6.4E-1	9.9E-1
GOTERM_MF_FAT	translation initiation factor activity	RT	15	6.4E-1	1.0E0
GOTERM_BP_FAT	translational initiation	RT	7	6.8E-1	1.0E0
GOTERM_MF_FAT	translation factor activity, nucleic acid binding	RT	18	7.6E-1	1.0E0
Annotation Cluster 31	Enrichment Score: 0.17		Count	P_Value	Benjamini
INTERPRO	ABC transporter, conserved site	RT	15	3.9E-1	1.0E0
INTERPRO	ABC transporter-like	RT	15	4.9E-1	1.0E0
INTERPRO	ATPase, AAA-type, core	RT	14	8.0E-1	1.0E0
INTERPRO	ATPase, AAA+ type, core	RT	39	8.2E-1	1.0E0
SMART	AAA	RT	39	8.5E-1	1.0E0
COG_ONTOLOGY	Defense mechanisms	RT	11	9.2E-1	1.0E0
Annotation Cluster 32	Enrichment Score: 0.17		Count	P_Value	Benjamini
INTERPRO	Tetratricopeptide region	RT	16	4.2E-1	1.0E0
INTERPRO	Tetratricopeptide-like helical	RT	19	5.8E-1	1.0E0
INTERPRO	Tetratricopeptide repeat	RT	14	7.3E-1	1.0E0
SMART	TPR	RT	14	7.5E-1	1.0E0
INTERPRO	Tetratricopeptide TPR-1	RT	11	8.3E-1	1.0E0
SP_PIR_KEYWORDS	tpr repeat	RT	5	8.9E-1	1.0E0
Annotation Cluster 33	Enrichment Score: 0.16		Count	P_Value	Benjamini
GOTERM_MF_FAT	NAD or NADH binding	RT	6	5.4E-1	1.0E0
GOTERM_BP_FAT	regulation of gene expression, epigenetic	RT	4	6.2E-1	1.0E0
GOTERM_BP_FAT	negative regulation of transcription	RT	4	6.2E-1	1.0E0
GOTERM_BP_FAT	chromatin silencing	RT	4	6.2E-1	1.0E0
GOTERM_BP_FAT	negative regulation of nucleobase, nucleoside, nucleotide and nucleic acid metabolic process	RT	4	6.2E-1	1.0E0
GOTERM_BP_FAT	negative regulation of transcription, DNA-dependent	RT	4	6.2E-1	1.0E0
GOTERM_BP_FAT	negative regulation of gene expression, epigenetic	RT	4	6.2E-1	1.0E0
GOTERM_BP_FAT	negative regulation of macromolecule biosynthetic process	RT	4	6.2E-1	1.0E0
GOTERM_BP_FAT	negative regulation of biosynthetic process	RT	4	6.2E-1	1.0E0
GOTERM_BP_FAT	negative regulation of RNA metabolic process	RT	4	6.2E-1	1.0E0
GOTERM_BP_FAT	negative regulation of nitrogen compound metabolic process	RT	4	6.2E-1	1.0E0
GOTERM_BP_FAT	negative regulation of cellular biosynthetic process	RT	4	6.2E-1	1.0E0
GOTERM_MF_FAT	NAD binding	RT	4	6.4E-1	1.0E0
INTERPRO	NAD-dependent histone deacetylase, silent information regulator Sir2	RT	4	6.5E-1	1.0E0
GOTERM_BP_FAT	protein amino acid deacetylation	RT	4	7.7E-1	1.0E0
GOTERM_BP_FAT	regulation of transcription, DNA-dependent	RT	7	7.8E-1	1.0E0
GOTERM_BP_FAT	regulation of RNA metabolic process	RT	7	7.8E-1	1.0E0
GOTERM_MF_FAT	hydrolase activity, acting on carbon-nitrogen (but not peptide) bonds, in linear amides	RT	5	7.9E-1	1.0E0
GOTERM_BP_FAT	gene silencing	RT	4	8.7E-1	1.0E0
GOTERM_BP_FAT	negative regulation of macromolecule metabolic process	RT	4	8.7E-1	1.0E0
GOTERM_BP_FAT	negative regulation of gene expression	RT	4	8.7E-1	1.0E0
GOTERM_BP_FAT	regulation of transcription	RT	15	9.3E-1	1.0E0
Annotation Cluster 34	Enrichment Score: 0.16		Count	P_Value	Benjamini
GOTERM_BP_FAT	generation of precursor metabolites and energy	RT	17	3.8E-1	1.0E0
GOTERM_MF_FAT	inorganic cation transmembrane transporter activity	RT	12	4.8E-1	1.0E0
GOTERM_CC_FAT	proton-transporting two-sector ATPase complex, proton-transporting domain	RT	5	4.8E-1	1.0E0
GOTERM_BP_FAT	purine ribonucleotide metabolic process	RT	17	5.6E-1	1.0E0
GOTERM_BP_FAT	purine ribonucleotide biosynthetic process	RT	17	5.6E-1	1.0E0
GOTERM_BP_FAT	ribonucleotide biosynthetic process	RT	17	5.6E-1	1.0E0
GOTERM_BP_FAT	ribonucleotide metabolic process	RT	17	5.6E-1	1.0E0

GOTERM_BP_FAT	transmembrane transport	RT	13	5.7E-1	1.0E0
GOTERM_BP_FAT	purine nucleotide metabolic process	RT	18	5.8E-1	1.0E0
GOTERM_BP_FAT	nucleotide biosynthetic process	RT	23	5.8E-1	1.0E0
GOTERM_MF_FAT	di-, tri-valent inorganic cation transmembrane transporter activity	RT	3	6.1E-1	1.0E0
GOTERM_MF_FAT	primary active transmembrane transporter activity	RT	18	6.1E-1	1.0E0
GOTERM_MF_FAT	P-P-bond-hydrolysis-driven transmembrane transporter activity	RT	18	6.1E-1	1.0E0
INTERPRO	ATPase, P-type cation-transporter, N-terminal	RT	3	6.2E-1	1.0E0
INTERPRO	ATPase, P-type cation-transporter, C-terminal	RT	3	6.2E-1	1.0E0
GOTERM_BP_FAT	purine nucleoside triphosphate metabolic process	RT	16	6.3E-1	1.0E0
GOTERM_BP_FAT	purine nucleoside triphosphate biosynthetic process	RT	16	6.3E-1	1.0E0
GOTERM_BP_FAT	nucleoside triphosphate metabolic process	RT	16	6.3E-1	1.0E0
GOTERM_BP_FAT	nucleoside triphosphate biosynthetic process	RT	16	6.3E-1	1.0E0
GOTERM_BP_FAT	purine ribonucleoside triphosphate metabolic process	RT	16	6.3E-1	1.0E0
GOTERM_BP_FAT	purine ribonucleoside triphosphate biosynthetic process	RT	16	6.3E-1	1.0E0
GOTERM_BP_FAT	ribonucleoside triphosphate metabolic process	RT	16	6.3E-1	1.0E0
GOTERM_BP_FAT	ribonucleoside triphosphate biosynthetic process	RT	16	6.3E-1	1.0E0
GOTERM_CC_FAT	proton-transporting V-type ATPase, V0 domain	RT	4	6.3E-1	1.0E0
GOTERM_MF_FAT	metal ion transmembrane transporter activity	RT	4	6.4E-1	1.0E0
GOTERM_BP_FAT	purine nucleotide biosynthetic process	RT	17	6.4E-1	1.0E0
GOTERM_BP_FAT	nitrogen compound biosynthetic process	RT	28	6.5E-1	1.0E0
GOTERM_BP_FAT	nucleobase, nucleoside and nucleotide biosynthetic process	RT	24	6.6E-1	1.0E0
GOTERM_BP_FAT	nucleobase, nucleoside, nucleotide and nucleic acid biosynthetic process	RT	24	6.6E-1	1.0E0
GOTERM_BP_FAT	ion transport	RT	14	6.8E-1	1.0E0
GOTERM_BP_FAT	cation transport	RT	14	6.8E-1	1.0E0
INTERPRO	ATPase, P-type, ATPase-associated region	RT	7	7.1E-1	1.0E0
GOTERM_MF_FAT	monovalent inorganic cation transmembrane transporter activity	RT	9	7.2E-1	1.0E0
GOTERM_MF_FAT	hydrogen ion transmembrane transporter activity	RT	9	7.2E-1	1.0E0
GOTERM_MF_FAT	ATPase activity, coupled to transmembrane movement of substances	RT	16	7.3E-1	1.0E0
GOTERM_MF_FAT	ATPase activity, coupled to movement of substances	RT	16	7.3E-1	1.0E0
GOTERM_BP_FAT	ATP biosynthetic process	RT	14	7.5E-1	1.0E0
GOTERM_BP_FAT	ATP metabolic process	RT	14	7.5E-1	1.0E0
GOTERM_BP_FAT	ion transmembrane transport	RT	8	7.8E-1	1.0E0
GOTERM_MF_FAT	cation-transporting ATPase activity	RT	6	7.9E-1	1.0E0
GOTERM_MF_FAT	ATPase activity, coupled to transmembrane movement of ions, phosphorylative mechanism	RT	7	8.0E-1	1.0E0
INTERPRO	Haloacid dehalogenase-like hydrolase	RT	6	8.0E-1	1.0E0
INTERPRO	ATPase, P-type, K/Mg/Cd/Cu/Zn/Na/Ca/Na/H-transporter	RT	7	8.1E-1	1.0E0
GOTERM_MF_FAT	ATPase activity, coupled to transmembrane movement of ions	RT	11	8.2E-1	1.0E0
GOTERM_MF_FAT	hydrolase activity, acting on acid anhydrides, catalyzing transmembrane movement of substances	RT	16	8.5E-1	1.0E0
GOTERM_BP_FAT	hydrogen transport	RT	8	8.5E-1	1.0E0
GOTERM_BP_FAT	proton transport	RT	8	8.5E-1	1.0E0
GOTERM_BP_FAT	oxidative phosphorylation	RT	7	8.5E-1	1.0E0
GOTERM_BP_FAT	ATP synthesis coupled proton transport	RT	7	8.5E-1	1.0E0
GOTERM_BP_FAT	energy coupled proton transport, down electrochemical gradient	RT	7	8.5E-1	1.0E0
KEGG_PATHWAY	Oxidative phosphorylation	RT	7	8.6E-1	1.0E0
GOTERM_CC_FAT	proton-transporting two-sector ATPase complex	RT	8	8.6E-1	1.0E0
GOTERM_CC_FAT	proton-transporting V-type ATPase complex	RT	5	8.7E-1	1.0E0
GOTERM_BP_FAT	monovalent inorganic cation transport	RT	8	9.0E-1	1.0E0
SP_PIR_KEYWORDS	ion transport	RT	3	9.1E-1	9.9E-1
INTERPRO	ATPase, P-type phosphorylation site	RT	4	9.4E-1	1.0E0
GOTERM_MF_FAT	proton-transporting ATPase activity, rotational mechanism	RT	3	9.7E-1	1.0E0

GOTERM_CC_FAT	proton-transporting two-sector ATPase complex, catalytic domain	RT			3	9.9E-1	1.0E0
Annotation Cluster 35	Enrichment Score: 0.15	G			Count	P_Value	Benjamini
GOTERM_MF_FAT	protein kinase regulator activity	RT			5	6.6E-1	1.0E0
GOTERM_MF_FAT	kinase regulator activity	RT			5	6.6E-1	1.0E0
GOTERM_MF_FAT	cAMP-dependent protein kinase regulator activity	RT			3	7.9E-1	1.0E0
Annotation Cluster 36	Enrichment Score: 0.15	G			Count	P_Value	Benjamini
GOTERM_CC_FAT	endomembrane system	RT			12	2.4E-1	1.0E0
GOTERM_CC_FAT	organelle membrane	RT			8	4.5E-1	1.0E0
GOTERM_CC_FAT	coated membrane	RT			9	6.1E-1	1.0E0
GOTERM_CC_FAT	membrane coat	RT			9	6.1E-1	1.0E0
GOTERM_CC_FAT	transport vesicle membrane	RT			4	6.3E-1	1.0E0
GOTERM_CC_FAT	transport vesicle	RT			4	6.3E-1	1.0E0
GOTERM_CC_FAT	Golgi apparatus	RT			7	6.9E-1	1.0E0
GOTERM_CC_FAT	cytoplasmic vesicle part	RT			4	7.8E-1	1.0E0
GOTERM_CC_FAT	coated vesicle membrane	RT			4	7.8E-1	1.0E0
GOTERM_CC_FAT	coated vesicle	RT			4	7.8E-1	1.0E0
GOTERM_CC_FAT	cytoplasmic vesicle membrane	RT			4	7.8E-1	1.0E0
GOTERM_CC_FAT	vesicle coat	RT			4	7.8E-1	1.0E0
GOTERM_CC_FAT	cytoplasmic vesicle	RT			4	7.8E-1	1.0E0
GOTERM_CC_FAT	vesicle	RT			4	7.8E-1	1.0E0
GOTERM_CC_FAT	membrane-bounded vesicle	RT			4	7.8E-1	1.0E0
GOTERM_CC_FAT	cytoplasmic membrane-bounded vesicle	RT			4	7.8E-1	1.0E0
GOTERM_CC_FAT	vesicle membrane	RT			4	7.8E-1	1.0E0
GOTERM_CC_FAT	ER to Golgi transport vesicle	RT			3	7.9E-1	1.0E0
GOTERM_CC_FAT	COPII vesicle coat	RT			3	7.9E-1	1.0E0
GOTERM_CC_FAT	ER to Golgi transport vesicle membrane	RT			3	7.9E-1	1.0E0
GOTERM_CC_FAT	Golgi apparatus part	RT			4	9.3E-1	1.0E0
GOTERM_BP_FAT	Golgi vesicle transport	RT			3	9.4E-1	1.0E0
GOTERM_BP_FAT	ER to Golgi vesicle-mediated transport	RT			3	9.4E-1	1.0E0
Annotation Cluster 37	Enrichment Score: 0.15	G			Count	P_Value	Benjamini
INTERPRO	Protein-tyrosine phosphatase, active site	RT			5	5.1E-1	1.0E0
INTERPRO	Protein-tyrosine phosphatase	RT			4	6.5E-1	1.0E0
INTERPRO	Dual-specific/protein-tyrosine phosphatase, conserved region	RT			4	6.5E-1	1.0E0
GOTERM_BP_FAT	dephosphorylation	RT			9	7.9E-1	1.0E0
GOTERM_BP_FAT	protein amino acid dephosphorylation	RT			7	8.5E-1	1.0E0
GOTERM_MF_FAT	protein tyrosine phosphatase activity	RT			4	8.8E-1	1.0E0
Annotation Cluster 38	Enrichment Score: 0.14	G			Count	P_Value	Benjamini
GOTERM_MF_FAT	calcium-dependent phospholipid binding	RT			14	5.3E-1	1.0E0
GOTERM_MF_FAT	lipid binding	RT			22	5.3E-1	1.0E0
INTERPRO	Alpha giardin	RT			8	6.2E-1	1.0E0
GOTERM_MF_FAT	calcium ion binding	RT			25	6.5E-1	1.0E0
GOTERM_BP_FAT	cytoskeleton organization	RT			9	7.9E-1	1.0E0
INTERPRO	Annexin repeat	RT			8	8.2E-1	1.0E0
INTERPRO	Annexin	RT			9	8.2E-1	1.0E0
SMART	ANX	RT			8	8.3E-1	1.0E0
SP_PIR_KEYWORDS	annexin	RT			9	8.4E-1	9.9E-1
GOTERM_MF_FAT	phospholipid binding	RT			14	8.8E-1	1.0E0
Annotation Cluster 39	Enrichment Score: 0.13	G			Count	P_Value	Benjamini
GOTERM_MF_FAT	endopeptidase activity	RT			29	3.8E-1	1.0E0
GOTERM_MF_FAT	peptidase activity, acting on L-amino acid peptides	RT			40	5.6E-1	1.0E0
GOTERM_MF_FAT	peptidase activity	RT			46	5.9E-1	1.0E0
GOTERM_BP_FAT	proteolysis	RT			48	7.8E-1	1.0E0
INTERPRO	Peptidase C1A, papain C-terminal	RT			15	8.1E-1	1.0E0
INTERPRO	Peptidase C1A, papain	RT			15	8.1E-1	1.0E0
SMART	Pept_C1	RT			15	8.3E-1	1.0E0
GOTERM_MF_FAT	cysteine-type endopeptidase activity	RT			13	8.3E-1	1.0E0
GOTERM_MF_FAT	cysteine-type peptidase activity	RT			16	8.5E-1	1.0E0

					Count	P_Value	Benjamini
INTERPRO	Peptidase, cysteine peptidase active site	RT			16	8.6E-1	1.0E0
SP_PIR_KEYWORDS	Protease	RT			19	9.0E-1	1.0E0
SP_PIR_KEYWORDS	thiol protease	RT			5	9.4E-1	1.0E0
Annotation Cluster 40	Enrichment Score: 0.13	G			Count	P_Value	Benjamini
KEGG_PATHWAY	Mismatch repair	RT			9	6.0E-1	1.0E0
KEGG_PATHWAY	DNA replication	RT			12	7.4E-1	1.0E0
KEGG_PATHWAY	Nucleotide excision repair	RT			8	9.1E-1	1.0E0
Annotation Cluster 41	Enrichment Score: 0.13	G			Count	P_Value	Benjamini
SP_PIR_KEYWORDS	coiled coil	RT			4	4.6E-1	1.0E0
INTERPRO	ATPase associated with various cellular activities, AAA-5	RT			5	5.1E-1	1.0E0
COG_ONTOLOGY	Cytoskeleton	RT			11	8.8E-1	1.0E0
GOTERM_CC_FAT	dynein complex	RT			6	9.2E-1	1.0E0
INTERPRO	Dynein heavy chain	RT			6	9.3E-1	1.0E0
INTERPRO	Dynein heavy chain, N-terminal region 2	RT			4	9.9E-1	1.0E0
Annotation Cluster 42	Enrichment Score: 0.12	G			Count	P_Value	Benjamini
SP_PIR_KEYWORDS	magnesium	RT			9	4.1E-1	1.0E0
GOTERM_BP_FAT	dephosphorylation	RT			9	7.9E-1	1.0E0
GOTERM_MF_FAT	protein serine/threonine phosphatase activity	RT			3	7.9E-1	1.0E0
INTERPRO	Protein phosphatase 2C, N-terminal	RT			4	8.0E-1	1.0E0
INTERPRO	Protein phosphatase 2C-related	RT			4	8.0E-1	1.0E0
INTERPRO	Protein phosphatase 2C	RT			4	8.0E-1	1.0E0
INTERPRO	Protein phosphatase 2C, manganese/magnesium aspartate binding site	RT			3	8.0E-1	1.0E0
SMART	PP2C_SIG	RT			4	8.0E-1	1.0E0
SMART	PP2Cc	RT			4	8.0E-1	1.0E0
SP_PIR_KEYWORDS	protein phosphatase	RT			3	8.1E-1	9.9E-1
GOTERM_BP_FAT	protein amino acid dephosphorylation	RT			7	8.5E-1	1.0E0
GOTERM_CC_FAT	protein serine/threonine phosphatase complex	RT			4	8.7E-1	1.0E0
Annotation Cluster 43	Enrichment Score: 0.11	G			Count	P_Value	Benjamini
GOTERM_BP_FAT	cellular protein complex assembly	RT			8	4.4E-1	1.0E0
GOTERM_BP_FAT	protein complex biogenesis	RT			8	6.9E-1	1.0E0
GOTERM_BP_FAT	protein complex assembly	RT			8	6.9E-1	1.0E0
GOTERM_BP_FAT	protein polymerization	RT			5	7.7E-1	1.0E0
INTERPRO	Tubulin	RT			5	8.0E-1	1.0E0
INTERPRO	Tubulin/FtsZ, GTPase domain	RT			5	8.0E-1	1.0E0
INTERPRO	Tubulin, conserved site	RT			5	8.0E-1	1.0E0
PIR_SUPERFAMILY	PIRSF002306:tubulin	RT			4	8.4E-1	1.0E0
INTERPRO	Tubulin/FtsZ, 2-layer sandwich domain	RT			4	8.9E-1	1.0E0
GOTERM_MF_FAT	GTPase activity	RT			8	9.5E-1	1.0E0
INTERPRO	Beta tubulin, autoregulation binding site	RT			3	9.5E-1	1.0E0
Annotation Cluster 44	Enrichment Score: 0.11	G			Count	P_Value	Benjamini
GOTERM_BP_FAT	glycerophospholipid metabolic process	RT			5	6.4E-1	1.0E0
GOTERM_BP_FAT	glycerolipid metabolic process	RT			5	6.4E-1	1.0E0
GOTERM_BP_FAT	phosphoinositide metabolic process	RT			5	6.4E-1	1.0E0
GOTERM_BP_FAT	phospholipid metabolic process	RT			7	7.8E-1	1.0E0
GOTERM_BP_FAT	organophosphate metabolic process	RT			7	8.5E-1	1.0E0
GOTERM_BP_FAT	phospholipid biosynthetic process	RT			4	8.7E-1	1.0E0
GOTERM_MF_FAT	inositol or phosphatidylinositol kinase activity	RT			3	8.9E-1	1.0E0
GOTERM_BP_FAT	lipid biosynthetic process	RT			7	9.1E-1	1.0E0
Annotation Cluster 45	Enrichment Score: 0.11	G			Count	P_Value	Benjamini
GOTERM_BP_FAT	cell cycle	RT			5	7.7E-1	1.0E0
GOTERM_BP_FAT	M phase	RT			4	7.7E-1	1.0E0
GOTERM_BP_FAT	cell cycle process	RT			4	7.7E-1	1.0E0
GOTERM_BP_FAT	cell cycle phase	RT			4	7.7E-1	1.0E0
GOTERM_BP_FAT	organelle fission	RT			3	7.8E-1	1.0E0
GOTERM_BP_FAT	mitosis	RT			3	7.8E-1	1.0E0
GOTERM_BP_FAT	mitotic cell cycle	RT			3	7.8E-1	1.0E0
GOTERM_BP_FAT	M phase of mitotic cell cycle	RT			3	7.8E-1	1.0E0

GOTERM_BP_FAT	nuclear division	RT	3	7.8E-1	1.0E0
Annotation Cluster 46	Enrichment Score: 0.11		Count	P_Value	Benjamini
GOTERM_BP_FAT	rRNA metabolic process	RT	8	6.9E-1	1.0E0
GOTERM_BP_FAT	rRNA processing	RT	8	6.9E-1	1.0E0
GOTERM_BP_FAT	ncRNA processing	RT	16	7.7E-1	1.0E0
GOTERM_BP_FAT	ribosome biogenesis	RT	11	8.0E-1	1.0E0
GOTERM_BP_FAT	ribonucleoprotein complex biogenesis	RT	11	8.0E-1	1.0E0
GOTERM_BP_FAT	tRNA processing	RT	9	8.5E-1	1.0E0
GOTERM_BP_FAT	RNA processing	RT	23	9.2E-1	1.0E0
Annotation Cluster 47	Enrichment Score: 0.1		Count	P_Value	Benjamini
INTERPRO	Chaperonin TCP-1, conserved site	RT	6	6.9E-1	1.0E0
SP_PIR_KEYWORDS	cytoplasm	RT	11	7.1E-1	9.9E-1
PIR_SUPERFAMILY	PIRSF002584:molecular chaperone t-complex-type	RT	5	7.3E-1	1.0E0
GOTERM_MF_FAT	unfolded protein binding	RT	12	7.6E-1	1.0E0
INTERPRO	Chaperone, tailless complex polypeptide 1	RT	5	8.0E-1	1.0E0
INTERPRO	Chaperonin Cpn60/TCP-1	RT	5	8.8E-1	1.0E0
SP_PIR_KEYWORDS	Chaperone	RT	5	9.8E-1	1.0E0
Annotation Cluster 48	Enrichment Score: 0.1		Count	P_Value	Benjamini
GOTERM_BP_FAT	cellular response to stress	RT	12	7.3E-1	1.0E0
GOTERM_BP_FAT	DNA repair	RT	11	8.0E-1	1.0E0
GOTERM_BP_FAT	response to DNA damage stimulus	RT	11	8.0E-1	1.0E0
GOTERM_BP_FAT	DNA metabolic process	RT	31	8.6E-1	1.0E0
Annotation Cluster 49	Enrichment Score: 0.09		Count	P_Value	Benjamini
GOTERM_MF_FAT	transferase activity, transferring nitrogenous groups	RT	5	6.6E-1	1.0E0
GOTERM_MF_FAT	transaminase activity	RT	3	8.9E-1	1.0E0
SP_PIR_KEYWORDS	Aminotransferase	RT	3	9.1E-1	9.9E-1
Annotation Cluster 50	Enrichment Score: 0.09		Count	P_Value	Benjamini
SP_PIR_KEYWORDS	heme	RT	4	6.7E-1	9.9E-1
INTERPRO	Cytochrome b5, heme-binding site	RT	3	8.0E-1	1.0E0
GOTERM_MF_FAT	heme binding	RT	4	8.8E-1	1.0E0
GOTERM_MF_FAT	tetrapyrrole binding	RT	4	8.8E-1	1.0E0
INTERPRO	Cytochrome b5	RT	3	9.0E-1	1.0E0
Annotation Cluster 51	Enrichment Score: 0.08		Count	P_Value	Benjamini
INTERPRO	EGF-like, type 3	RT	19	6.6E-1	1.0E0
INTERPRO	EGF-like, laminin	RT	13	9.3E-1	1.0E0
SMART	EGF_Lam	RT	13	9.4E-1	1.0E0
Annotation Cluster 52	Enrichment Score: 0.06		Count	P_Value	Benjamini
INTERPRO	EF-HAND 1	RT	13	5.3E-1	1.0E0
INTERPRO	EF-HAND 2	RT	9	8.8E-1	1.0E0
SP_PIR_KEYWORDS	calcium	RT	8	8.9E-1	1.0E0
INTERPRO	EF hand	RT	5	9.6E-1	1.0E0
INTERPRO	Calcium-binding EF-hand	RT	5	9.6E-1	1.0E0
SMART	EFh	RT	5	9.7E-1	1.0E0
INTERPRO	EF-Hand type	RT	8	9.7E-1	1.0E0
Annotation Cluster 53	Enrichment Score: 0.06		Count	P_Value	Benjamini
GOTERM_BP_FAT	ncRNA metabolic process	RT	28	8.0E-1	1.0E0
GOTERM_BP_FAT	tRNA aminoacylation for protein translation	RT	12	8.0E-1	1.0E0
GOTERM_MF_FAT	aminoacyl-tRNA ligase activity	RT	13	8.3E-1	1.0E0
GOTERM_MF_FAT	ligase activity, forming carbon-oxygen bonds	RT	13	8.3E-1	1.0E0
GOTERM_MF_FAT	ligase activity, forming aminoacyl-tRNA and related compounds	RT	13	8.3E-1	1.0E0
GOTERM_BP_FAT	tRNA metabolic process	RT	21	8.5E-1	1.0E0
GOTERM_BP_FAT	amino acid activation	RT	12	8.6E-1	1.0E0
GOTERM_BP_FAT	tRNA aminoacylation	RT	12	8.6E-1	1.0E0
KEGG_PATHWAY	Aminoacyl-tRNA biosynthesis	RT	11	8.7E-1	1.0E0
SP_PIR_KEYWORDS	Aminoacyl-tRNA synthetase	RT	12	9.0E-1	1.0E0
INTERPRO	Aminoacyl-tRNA synthetase, class II, conserved region	RT	4	9.7E-1	1.0E0
SP_PIR_KEYWORDS	ligase	RT	25	9.9E-1	1.0E0

					Count	P_Value	Benjamini
Annotation Cluster 54		Enrichment Score: 0.06			Count	P_Value	Benjamini
GOTERM_BP_FAT	dephosphorylation	RT			9	7.9E-1	1.0E0
GOTERM_BP_FAT	protein amino acid dephosphorylation	RT			7	8.5E-1	1.0E0
GOTERM_MF_FAT	protein tyrosine/serine/threonine phosphatase activity	RT			3	8.9E-1	1.0E0
INTERPRO	Protein-tyrosine phosphatase, dual specificity	RT			3	9.0E-1	1.0E0
INTERPRO	Dual specificity phosphatase, catalytic domain	RT			3	9.0E-1	1.0E0
Annotation Cluster 55		Enrichment Score: 0.06			Count	P_Value	Benjamini
INTERPRO	Myb transcription factor	RT			3	8.0E-1	1.0E0
INTERPRO	Myb-type HTH DNA-binding domain	RT			3	8.0E-1	1.0E0
INTERPRO	Myb, DNA-binding	RT			3	9.0E-1	1.0E0
INTERPRO	SANT, DNA-binding	RT			5	9.3E-1	1.0E0
SMART	SANT	RT			5	9.4E-1	1.0E0
Annotation Cluster 56		Enrichment Score: 0.05			Count	P_Value	Benjamini
INTERPRO	Na+ driven multidrug efflux pump	RT			3	8.0E-1	1.0E0
GOTERM_BP_FAT	multidrug transport	RT			3	8.9E-1	1.0E0
GOTERM_BP_FAT	response to drug	RT			3	8.9E-1	1.0E0
GOTERM_BP_FAT	drug transport	RT			3	8.9E-1	1.0E0
GOTERM_MF_FAT	drug transporter activity	RT			3	8.9E-1	1.0E0
INTERPRO	Multi antimicrobial extrusion protein MatE	RT			3	9.0E-1	1.0E0
GOTERM_MF_FAT	antiporter activity	RT			3	9.5E-1	1.0E0
Annotation Cluster 57		Enrichment Score: 0.05			Count	P_Value	Benjamini
GOTERM_BP_FAT	vitamin metabolic process	RT			3	8.9E-1	1.0E0
GOTERM_BP_FAT	water-soluble vitamin metabolic process	RT			3	8.9E-1	1.0E0
GOTERM_BP_FAT	water-soluble vitamin biosynthetic process	RT			3	8.9E-1	1.0E0
GOTERM_BP_FAT	vitamin biosynthetic process	RT			3	8.9E-1	1.0E0
Annotation Cluster 58		Enrichment Score: 0.05			Count	P_Value	Benjamini
INTERPRO	Thioredoxin domain	RT			4	8.0E-1	1.0E0
INTERPRO	Thioredoxin fold	RT			12	8.4E-1	1.0E0
GOTERM_BP_FAT	cellular homeostasis	RT			10	8.6E-1	1.0E0
INTERPRO	Thioredoxin-like	RT			7	8.8E-1	1.0E0
GOTERM_MF_FAT	antioxidant activity	RT			4	8.8E-1	1.0E0
INTERPRO	Thioredoxin-like subdomain	RT			3	9.0E-1	1.0E0
GOTERM_BP_FAT	homeostatic process	RT			10	9.0E-1	1.0E0
GOTERM_BP_FAT	cell redox homeostasis	RT			9	9.0E-1	1.0E0
INTERPRO	Alkyl hydroperoxide reductase/ Thiol specific antioxidant/ Mal allergen	RT			3	9.5E-1	1.0E0
INTERPRO	Thioredoxin, core	RT			3	9.8E-1	1.0E0
Annotation Cluster 59		Enrichment Score: 0.05			Count	P_Value	Benjamini
GOTERM_CC_FAT	membrane-enclosed lumen	RT			3	8.9E-1	1.0E0
GOTERM_CC_FAT	organelle lumen	RT			3	8.9E-1	1.0E0
GOTERM_CC_FAT	nuclear lumen	RT			3	8.9E-1	1.0E0
GOTERM_CC_FAT	intracellular organelle lumen	RT			3	8.9E-1	1.0E0
Annotation Cluster 60		Enrichment Score: 0.05			Count	P_Value	Benjamini
GOTERM_BP_FAT	carboxylic acid biosynthetic process	RT			4	8.7E-1	1.0E0
GOTERM_BP_FAT	organic acid biosynthetic process	RT			4	8.7E-1	1.0E0
GOTERM_BP_FAT	cellular amino acid biosynthetic process	RT			3	8.9E-1	1.0E0
GOTERM_BP_FAT	amine biosynthetic process	RT			3	9.4E-1	1.0E0
Annotation Cluster 61		Enrichment Score: 0.05			Count	P_Value	Benjamini
INTERPRO	von Willebrand factor, type A	RT			4	8.9E-1	1.0E0
SMART	VWA	RT			4	8.9E-1	1.0E0
INTERPRO	Copine	RT			3	9.0E-1	1.0E0
Annotation Cluster 62		Enrichment Score: 0.05			Count	P_Value	Benjamini
GOTERM_CC_FAT	cytoskeleton	RT			30	7.5E-1	1.0E0
GOTERM_CC_FAT	microtubule	RT			18	8.0E-1	1.0E0
GOTERM_CC_FAT	cytoskeletal part	RT			28	8.3E-1	1.0E0
GOTERM_CC_FAT	microtubule cytoskeleton	RT			28	8.3E-1	1.0E0
SP_PIR_KEYWORDS	microtubule	RT			17	8.9E-1	1.0E0
GOTERM_BP_FAT	microtubule-based process	RT			27	9.2E-1	1.0E0

Category	Term		Count	P_Value	Benjamini
GOTERM_MF_FAT	motor activity	RT	21	9.3E-1	1.0E0
GOTERM_MF_FAT	microtubule motor activity	RT	21	9.3E-1	1.0E0
SP_PIR_KEYWORDS	motor protein	RT	12	9.4E-1	1.0E0
GOTERM_BP_FAT	microtubule-based movement	RT	23	9.4E-1	1.0E0
INTERPRO	Kinesin, motor region	RT	13	9.7E-1	1.0E0
INTERPRO	Kinesin, motor region, conserved site	RT	8	9.7E-1	1.0E0
SMART	KISc	RT	13	9.7E-1	1.0E0
Annotation Cluster 63	Enrichment Score: 0.05		Count	P_Value	Benjamini
GOTERM_BP_FAT	cofactor metabolic process	RT	7	7.8E-1	1.0E0
GOTERM_BP_FAT	cofactor biosynthetic process	RT	5	8.6E-1	1.0E0
GOTERM_BP_FAT	coenzyme metabolic process	RT	5	9.2E-1	1.0E0
GOTERM_BP_FAT	pyridine nucleotide metabolic process	RT	3	9.4E-1	1.0E0
GOTERM_BP_FAT	oxidoreduction coenzyme metabolic process	RT	3	9.4E-1	1.0E0
GOTERM_BP_FAT	coenzyme biosynthetic process	RT	3	9.7E-1	1.0E0
Annotation Cluster 64	Enrichment Score: 0.04		Count	P_Value	Benjamini
GOTERM_BP_FAT	RNA-dependent DNA replication	RT	4	8.7E-1	1.0E0
INTERPRO	RNA-directed DNA polymerase (reverse transcriptase)	RT	4	8.9E-1	1.0E0
GOTERM_MF_FAT	RNA-directed DNA polymerase activity	RT	11	9.6E-1	1.0E0
Annotation Cluster 65	Enrichment Score: 0.04		Count	P_Value	Benjamini
GOTERM_BP_FAT	lipid transport	RT	3	8.9E-1	1.0E0
GOTERM_BP_FAT	lipid localization	RT	3	8.9E-1	1.0E0
GOTERM_MF_FAT	lipid transporter activity	RT	3	9.5E-1	1.0E0
Annotation Cluster 66	Enrichment Score: 0.04		Count	P_Value	Benjamini
GOTERM_MF_FAT	ubiquitin-protein ligase activity	RT	4	6.4E-1	1.0E0
GOTERM_MF_FAT	ligase activity, forming carbon-nitrogen bonds	RT	16	9.2E-1	1.0E0
GOTERM_MF_FAT	acid-amino acid ligase activity	RT	13	9.4E-1	1.0E0
GOTERM_MF_FAT	small conjugating protein ligase activity	RT	6	9.5E-1	1.0E0
KEGG_PATHWAY	Ubiquitin mediated proteolysis	RT	5	9.6E-1	1.0E0
INTERPRO	Ubiquitin-conjugating enzyme/RWD-like	RT	5	9.8E-1	1.0E0
INTERPRO	Ubiquitin-conjugating enzyme, E2	RT	5	9.8E-1	1.0E0
SMART	UBCc	RT	5	9.8E-1	1.0E0
SP_PIR_KEYWORDS	ligase	RT	25	9.9E-1	1.0E0
Annotation Cluster 67	Enrichment Score: 0.02		Count	P_Value	Benjamini
GOTERM_CC_FAT	ribonucleoprotein complex	RT	53	8.8E-1	1.0E0
GOTERM_CC_FAT	non-membrane-bounded organelle	RT	85	9.2E-1	1.0E0
GOTERM_CC_FAT	intracellular non-membrane-bounded organelle	RT	85	9.2E-1	1.0E0
GOTERM_MF_FAT	structural molecule activity	RT	42	9.5E-1	1.0E0
KEGG_PATHWAY	Ribosome	RT	34	9.5E-1	1.0E0
GOTERM_MF_FAT	structural constituent of ribosome	RT	34	9.6E-1	1.0E0
GOTERM_BP_FAT	translation	RT	56	9.6E-1	1.0E0
GOTERM_CC_FAT	ribosome	RT	37	9.7E-1	1.0E0
SP_PIR_KEYWORDS	ribosomal protein	RT	36	9.7E-1	1.0E0
Annotation Cluster 68	Enrichment Score: 0.02		Count	P_Value	Benjamini
GOTERM_MF_FAT	heat shock protein binding	RT	4	9.3E-1	1.0E0
INTERPRO	Heat shock protein DnaJ, N-terminal	RT	4	9.4E-1	1.0E0
SMART	DnaJ	RT	4	9.4E-1	1.0E0
INTERPRO	Molecular chaperone, heat shock protein, Hsp40, DnaJ	RT	3	9.8E-1	1.0E0
Annotation Cluster 69	Enrichment Score: 0.02		Count	P_Value	Benjamini
GOTERM_MF_FAT	acetyltransferase activity	RT	5	9.2E-1	1.0E0
INTERPRO	Acyl-CoA N-acyltransferase	RT	5	9.3E-1	1.0E0
GOTERM_MF_FAT	N-acetyltransferase activity	RT	4	9.6E-1	1.0E0
INTERPRO	GCN5-related N-acetyltransferase	RT	4	9.7E-1	1.0E0
GOTERM_MF_FAT	N-acyltransferase activity	RT	4	9.8E-1	1.0E0
Annotation Cluster 70	Enrichment Score: 0.01		Count	P_Value	Benjamini
INTERPRO	WD40 repeat 2	RT	21	9.6E-1	1.0E0
INTERPRO	WD40 repeat, region	RT	23	9.6E-1	1.0E0
INTERPRO	WD40 repeat	RT	36	9.8E-1	1.0E0
SMART	WD40	RT	36	9.9E-1	1.0E0

SP_PIR_KEYWORDS	wd repeat	RT			23	9.9E-1	1.0E0
INTERPRO	WD40 repeat, subgroup	RT			26	9.9E-1	1.0E0
INTERPRO	WD40/YVTN repeat-like	RT			34	9.9E-1	1.0E0
INTERPRO	WD40 repeat, conserved site	RT			9	1.0E0	1.0E0
Annotation Cluster 71	Enrichment Score: 0.01	G			Count	P_Value	Benjamini
GOTERM_MF_FAT	RNA-directed DNA polymerase activity	RT			11	9.6E-1	1.0E0
SP_PIR_KEYWORDS	Endonuclease	RT			14	9.7E-1	1.0E0
GOTERM_MF_FAT	nuclease activity	RT			23	9.8E-1	1.0E0
GOTERM_MF_FAT	DNA polymerase activity	RT			15	9.8E-1	1.0E0
SP_PIR_KEYWORDS	rna-directed dna polymerase	RT			10	9.8E-1	1.0E0
GOTERM_MF_FAT	endonuclease activity	RT			14	9.9E-1	1.0E0
Annotation Cluster 72	Enrichment Score: 0.01	G			Count	P_Value	Benjamini
INTERPRO	RNA recognition motif, RNP-1	RT			6	9.8E-1	1.0E0
SMART	RRM	RT			6	9.8E-1	1.0E0
INTERPRO	Nucleotide-binding, alpha-beta plait	RT			6	9.9E-1	1.0E0
Annotation Cluster 73	Enrichment Score: 0.01	G			Count	P_Value	Benjamini
GOTERM_MF_FAT	cis-trans isomerase activity	RT			3	9.9E-1	1.0E0
GOTERM_MF_FAT	peptidyl-prolyl cis-trans isomerase activity	RT			3	9.9E-1	1.0E0
SP_PIR_KEYWORDS	Rotamase	RT			3	9.9E-1	1.0E0

219 terms were not clustered.

Please cite *Nature Protocols 2009; 4(1):44 & Genome Biology 2003; 4(5):P3* within any publication that makes use of any methods inspired by **DAVID**.

Term of Service | Contact Us | Site Map

DAVID Bioinformatics Resources 6.7
National Institute of Allergy and Infectious Diseases (NIAID), NIH

Functional Annotation Clustering

Help and Manual

Current Gene List: 3_Random2
Current Background: Giardia intestinalis
3000 DAVID IDs

Options   Classification Stringency   Medium
Rerun using options   Create Sublist

79 Cluster(s)   Download File

Annotation Cluster 1	Enrichment Score: 1.83	Count	P_Value	Benjamini
INTERPRO	Tetratricopeptide TPR-1 RT	19	2.0E-3	8.9E-1
INTERPRO	Tetratricopeptide repeat RT	21	7.6E-3	9.9E-1
SMART	TPR RT	21	1.1E-2	8.2E-1
INTERPRO	Tetratricopeptide-like helical RT	25	1.9E-2	1.0E0
SP_PIR_KEYWORDS	tpr repeat RT	9	5.2E-2	1.0E0
INTERPRO	Tetratricopeptide region RT	19	6.4E-2	1.0E0
Annotation Cluster 2	Enrichment Score: 1.1	Count	P_Value	Benjamini
GOTERM_BP_FAT	cellular response to stress RT	17	4.8E-2	1.0E0
GOTERM_BP_FAT	response to DNA damage stimulus RT	16	6.7E-2	1.0E0
GOTERM_BP_FAT	DNA repair RT	16	6.7E-2	1.0E0
GOTERM_BP_FAT	DNA metabolic process RT	38	1.9E-1	1.0E0
Annotation Cluster 3	Enrichment Score: 0.65	Count	P_Value	Benjamini
GOTERM_CC_FAT	chromosome RT	21	3.2E-2	9.8E-1
GOTERM_CC_FAT	chromosomal part RT	14	6.0E-2	9.8E-1
GOTERM_BP_FAT	cellular macromolecular complex subunit organization RT	19	7.9E-2	1.0E0
GOTERM_CC_FAT	protein-DNA complex RT	11	9.5E-2	9.8E-1
GOTERM_BP_FAT	macromolecular complex subunit organization RT	20	9.6E-2	1.0E0
GOTERM_BP_FAT	cellular macromolecular complex assembly RT	18	1.1E-1	1.0E0
GOTERM_BP_FAT	macromolecular complex assembly RT	19	1.2E-1	1.0E0
GOTERM_CC_FAT	chromatin RT	10	1.4E-1	9.9E-1
GOTERM_CC_FAT	nucleosome RT	9	2.0E-1	9.9E-1
GOTERM_BP_FAT	chromatin assembly RT	9	2.1E-1	1.0E0
GOTERM_BP_FAT	protein-DNA complex assembly RT	9	2.1E-1	1.0E0
GOTERM_BP_FAT	nucleosome organization RT	9	2.1E-1	1.0E0
GOTERM_BP_FAT	nucleosome assembly RT	9	2.1E-1	1.0E0
GOTERM_BP_FAT	DNA packaging RT	9	2.1E-1	1.0E0
INTERPRO	Histone-fold RT	11	2.3E-1	1.0E0
SP_PIR_KEYWORDS	chromosomal protein RT	9	2.4E-1	9.9E-1
SP_PIR_KEYWORDS	nucleus RT	23	2.5E-1	9.8E-1
INTERPRO	Histone core RT	9	2.5E-1	1.0E0
GOTERM_BP_FAT	chromatin assembly or disassembly RT	10	2.6E-1	1.0E0
INTERPRO	Histone H3 RT	4	4.4E-1	1.0E0
SP_PIR_KEYWORDS	dna-binding RT	22	4.5E-1	9.9E-1
SMART	H3 RT	4	4.6E-1	1.0E0
GOTERM_BP_FAT	chromatin organization RT	10	4.9E-1	1.0E0
GOTERM_BP_FAT	chromosome organization RT	12	5.4E-1	1.0E0
INTERPRO	Histone H4 RT	3	6.2E-1	1.0E0
PIR_SUPERFAMILY	PIRSF002052:histone H4 RT	3	6.2E-1	1.0E0
SMART	H4 RT	3	6.4E-1	1.0E0
SP_PIR_KEYWORDS	nucleosome core RT	5	6.6E-1	9.8E-1
Annotation Cluster 4	Enrichment Score: 0.61	Count	P_Value	Benjamini
SP_PIR_KEYWORDS	egf-like domain RT	13	2.0E-1	1.0E0
INTERPRO	EGF-like, type 3 RT	22	2.4E-1	1.0E0
INTERPRO	EGF, extracellular RT	16	3.1E-1	1.0E0
Annotation Cluster 5	Enrichment Score: 0.6	Count	P_Value	Benjamini

GOTERM_MF_FAT	purine nucleotide binding	RT	338	1.3E-1	1.0E0
GOTERM_MF_FAT	purine ribonucleotide binding	RT	333	1.5E-1	1.0E0
GOTERM_MF_FAT	ribonucleotide binding	RT	333	1.5E-1	1.0E0
GOTERM_MF_FAT	purine nucleoside binding	RT	309	1.7E-1	1.0E0
GOTERM_MF_FAT	adenyl nucleotide binding	RT	309	1.7E-1	1.0E0
GOTERM_BP_FAT	protein amino acid phosphorylation	RT	158	1.9E-1	1.0E0
GOTERM_MF_FAT	adenyl ribonucleotide binding	RT	304	2.0E-1	1.0E0
GOTERM_MF_FAT	ATP binding	RT	304	2.0E-1	1.0E0
GOTERM_BP_FAT	phosphorylation	RT	168	2.2E-1	1.0E0
GOTERM_MF_FAT	nucleoside binding	RT	309	2.4E-1	1.0E0
GOTERM_BP_FAT	phosphorus metabolic process	RT	176	2.7E-1	1.0E0
GOTERM_BP_FAT	phosphate metabolic process	RT	176	2.7E-1	1.0E0
GOTERM_MF_FAT	protein kinase activity	RT	158	2.7E-1	1.0E0
GOTERM_MF_FAT	nucleotide binding	RT	353	2.9E-1	1.0E0
INTERPRO	Protein kinase, core	RT	157	3.9E-1	1.0E0
SP_PIR_KEYWORDS	atp-binding	RT	150	4.2E-1	9.9E-1
SP_PIR_KEYWORDS	kinase	RT	190	4.9E-1	9.9E-1
INTERPRO	Serine/threonine protein kinase-related	RT	135	5.4E-1	1.0E0
SP_PIR_KEYWORDS	nucleotide-binding	RT	155	5.5E-1	9.9E-1
Annotation Cluster 6	Enrichment Score: 0.56		Count	P_Value	Benjamini
SP_PIR_KEYWORDS	helicase	RT	31	9.4E-2	9.9E-1
GOTERM_MF_FAT	ATPase activity, coupled	RT	46	1.1E-1	1.0E0
GOTERM_MF_FAT	ATP-dependent helicase activity	RT	26	1.6E-1	1.0E0
GOTERM_MF_FAT	purine NTP-dependent helicase activity	RT	26	1.6E-1	1.0E0
GOTERM_MF_FAT	helicase activity	RT	35	2.0E-1	1.0E0
INTERPRO	DEAD-like helicase, N-terminal	RT	28	3.1E-1	1.0E0
INTERPRO	DNA/RNA helicase, DEAD/DEAH box type, N-terminal	RT	20	3.4E-1	1.0E0
GOTERM_MF_FAT	ATPase activity	RT	56	3.8E-1	1.0E0
SMART	DEXDc	RT	28	3.8E-1	1.0E0
INTERPRO	Helicase, superfamily 1 and 2, ATP-binding	RT	27	4.3E-1	1.0E0
INTERPRO	DNA/RNA helicase, C-terminal	RT	27	5.0E-1	1.0E0
INTERPRO	RNA helicase, DEAD-box type, Q motif	RT	11	5.7E-1	1.0E0
SMART	HELICc	RT	27	5.8E-1	1.0E0
Annotation Cluster 7	Enrichment Score: 0.48		Count	P_Value	Benjamini
GOTERM_MF_FAT	peptidyl-prolyl cis-trans isomerase activity	RT	7	2.5E-1	1.0E0
GOTERM_MF_FAT	cis-trans isomerase activity	RT	7	2.5E-1	1.0E0
SP_PIR_KEYWORDS	Rotamase	RT	7	2.6E-1	9.8E-1
SP_PIR_KEYWORDS	Isomerase	RT	20	4.0E-1	9.9E-1
GOTERM_BP_FAT	protein folding	RT	19	4.1E-1	1.0E0
INTERPRO	Peptidyl-prolyl cis-trans isomerase, FKBP-type	RT	5	5.0E-1	1.0E0
Annotation Cluster 8	Enrichment Score: 0.43		Count	P_Value	Benjamini
GOTERM_MF_FAT	lipid binding	RT	26	7.6E-2	1.0E0
GOTERM_BP_FAT	cytoskeleton organization	RT	12	2.3E-1	1.0E0
GOTERM_MF_FAT	phospholipid binding	RT	18	3.1E-1	1.0E0
INTERPRO	Alpha giardin	RT	9	3.8E-1	1.0E0
INTERPRO	Annexin repeat	RT	10	4.2E-1	1.0E0
SMART	ANX	RT	10	4.7E-1	1.0E0
GOTERM_MF_FAT	calcium-dependent phospholipid binding	RT	14	4.9E-1	1.0E0
GOTERM_MF_FAT	calcium ion binding	RT	25	6.0E-1	1.0E0
SP_PIR_KEYWORDS	annexin	RT	10	6.4E-1	9.9E-1
INTERPRO	Annexin	RT	10	6.5E-1	1.0E0
Annotation Cluster 9	Enrichment Score: 0.41		Count	P_Value	Benjamini
GOTERM_BP_FAT	translational elongation	RT	7	1.1E-1	1.0E0
SP_PIR_KEYWORDS	elongation factor	RT	5	6.6E-1	9.8E-1
GOTERM_MF_FAT	translation elongation factor activity	RT	5	7.7E-1	1.0E0
Annotation Cluster 10	Enrichment Score: 0.41		Count	P_Value	Benjamini
GOTERM_MF_FAT	tubulin-tyrosine ligase activity	RT	6	3.5E-1	1.0E0
	Tubulin-tyrosine ligase	RT	6	3.7E-1	1.0E0

INTERPRO					
GOTERM_MF_FAT	acid-amino acid ligase activity	RT	17	4.6E-1	1.0E0
Annotation Cluster 11	Enrichment Score: 0.39		Count	P_Value	Benjamini
GOTERM_CC_FAT	cell projection	RT	4	4.0E-1	1.0E0
GOTERM_CC_FAT	flagellum	RT	4	4.0E-1	1.0E0
SP_PIR_KEYWORDS	flagellum	RT	4	4.3E-1	9.9E-1
Annotation Cluster 12	Enrichment Score: 0.37		Count	P_Value	Benjamini
GOTERM_BP_FAT	intracellular transport	RT	19	2.5E-1	1.0E0
GOTERM_BP_FAT	intracellular protein transport	RT	16	2.6E-1	1.0E0
GOTERM_BP_FAT	cellular macromolecule localization	RT	16	2.6E-1	1.0E0
GOTERM_BP_FAT	cellular protein localization	RT	16	2.6E-1	1.0E0
GOTERM_BP_FAT	establishment of protein localization	RT	20	5.1E-1	1.0E0
GOTERM_BP_FAT	protein transport	RT	20	5.1E-1	1.0E0
GOTERM_CC_FAT	coated membrane	RT	9	5.6E-1	1.0E0
GOTERM_CC_FAT	membrane coat	RT	9	5.6E-1	1.0E0
GOTERM_BP_FAT	protein localization	RT	20	5.9E-1	1.0E0
GOTERM_BP_FAT	vesicle-mediated transport	RT	15	8.6E-1	1.0E0
Annotation Cluster 13	Enrichment Score: 0.36		Count	P_Value	Benjamini
GOTERM_MF_FAT	DNA helicase activity	RT	6	1.8E-1	1.0E0
GOTERM_MF_FAT	DNA-dependent ATPase activity	RT	7	2.5E-1	1.0E0
GOTERM_MF_FAT	ATP-dependent DNA helicase activity	RT	4	4.2E-1	1.0E0
INTERPRO	Helicase, superfamily 1 and 2, ATP-binding, DinG/Rad3-type	RT	3	6.2E-1	1.0E0
INTERPRO	DEAD2	RT	3	6.2E-1	1.0E0
INTERPRO	Helicase, ATP-dependent, c2 type	RT	3	6.2E-1	1.0E0
SMART	HELICc2	RT	3	6.4E-1	1.0E0
Annotation Cluster 14	Enrichment Score: 0.33		Count	P_Value	Benjamini
INTERPRO	Ankyrin	RT	257	3.2E-1	1.0E0
SP_PIR_KEYWORDS	ank repeat	RT	176	5.3E-1	9.9E-1
SMART	ANK	RT	257	6.0E-1	1.0E0
Annotation Cluster 15	Enrichment Score: 0.33		Count	P_Value	Benjamini
GOTERM_BP_FAT	cellular macromolecular complex assembly	RT	18	1.1E-1	1.0E0
GOTERM_BP_FAT	cellular protein complex assembly	RT	8	4.3E-1	1.0E0
GOTERM_BP_FAT	protein complex assembly	RT	9	4.6E-1	1.0E0
GOTERM_BP_FAT	protein complex biogenesis	RT	9	4.6E-1	1.0E0
GOTERM_BP_FAT	protein polymerization	RT	6	5.1E-1	1.0E0
INTERPRO	Tubulin, conserved site	RT	6	5.4E-1	1.0E0
INTERPRO	Tubulin	RT	6	5.4E-1	1.0E0
INTERPRO	Tubulin/FtsZ, GTPase domain	RT	6	5.4E-1	1.0E0
INTERPRO	Tubulin/FtsZ, 2-layer sandwich domain	RT	5	6.7E-1	1.0E0
PIR_SUPERFAMILY	PIRSF002306:tubulin	RT	5	6.7E-1	1.0E0
GOTERM_MF_FAT	GTPase activity	RT	10	7.1E-1	1.0E0
Annotation Cluster 16	Enrichment Score: 0.32		Count	P_Value	Benjamini
GOTERM_BP_FAT	protein localization in organelle	RT	5	2.8E-1	1.0E0
GOTERM_BP_FAT	protein targeting	RT	5	2.8E-1	1.0E0
KEGG_PATHWAY	Protein export	RT	3	5.7E-1	1.0E0
GOTERM_BP_FAT	protein targeting to ER	RT	3	5.9E-1	1.0E0
GOTERM_BP_FAT	SRP-dependent cotranslational protein targeting to membrane	RT	3	5.9E-1	1.0E0
GOTERM_BP_FAT	cotranslational protein targeting to membrane	RT	3	5.9E-1	1.0E0
GOTERM_BP_FAT	protein targeting to membrane	RT	3	5.9E-1	1.0E0
Annotation Cluster 17	Enrichment Score: 0.32		Count	P_Value	Benjamini
INTERPRO	EGF-like	RT	115	3.0E-1	1.0E0
INTERPRO	Giardia variant-specific surface protein	RT	141	3.4E-1	1.0E0
SMART	EGF	RT	115	4.6E-1	1.0E0
INTERPRO	Furin-like repeat	RT	105	6.8E-1	1.0E0
SMART	FU	RT	105	8.2E-1	1.0E0
Annotation Cluster 18	Enrichment Score: 0.31		Count	P_Value	Benjamini
GOTERM_BP_FAT	nucleotide biosynthetic process	RT	26	2.0E-1	1.0E0
GOTERM_BP_FAT	ATP metabolic process	RT	17	2.8E-1	1.0E0

	GOTERM_BP_FAT	ATP biosynthetic process	RT		17	2.8E-1	1.0E0
	GOTERM_BP_FAT	nitrogen compound biosynthetic process	RT		31	2.9E-1	1.0E0
	GOTERM_MF_FAT	ATPase activity, coupled to transmembrane movement of ions	RT		14	3.0E-1	1.0E0
	GOTERM_MF_FAT	ATPase activity, coupled to transmembrane movement of ions, phosphorylative mechanism	RT		9	3.4E-1	1.0E0
	GOTERM_BP_FAT	cation transport	RT		16	3.4E-1	1.0E0
	GOTERM_BP_FAT	ion transport	RT		16	3.4E-1	1.0E0
	INTERPRO	ATPase, P-type, K/Mg/Cd/Cu/Zn/Na/Ca/Na/H-transporter	RT		9	3.8E-1	1.0E0
	GOTERM_BP_FAT	transmembrane transport	RT		14	3.8E-1	1.0E0
	GOTERM_BP_FAT	purine ribonucleotide biosynthetic process	RT		18	3.9E-1	1.0E0
	GOTERM_BP_FAT	purine ribonucleotide metabolic process	RT		18	3.9E-1	1.0E0
	GOTERM_BP_FAT	ribonucleotide biosynthetic process	RT		18	3.9E-1	1.0E0
	GOTERM_BP_FAT	ribonucleotide metabolic process	RT		18	3.9E-1	1.0E0
	GOTERM_BP_FAT	nucleobase, nucleoside and nucleotide biosynthetic process	RT		26	3.9E-1	1.0E0
	GOTERM_BP_FAT	nucleobase, nucleoside, nucleotide and nucleic acid biosynthetic process	RT		26	3.9E-1	1.0E0
	KEGG_PATHWAY	Oxidative phosphorylation	RT		9	4.2E-1	1.0E0
	GOTERM_MF_FAT	inorganic cation transmembrane transporter activity	RT		12	4.4E-1	1.0E0
	GOTERM_BP_FAT	nucleoside triphosphate metabolic process	RT		17	4.6E-1	1.0E0
	GOTERM_BP_FAT	ribonucleoside triphosphate metabolic process	RT		17	4.6E-1	1.0E0
	GOTERM_BP_FAT	purine nucleoside triphosphate biosynthetic process	RT		17	4.6E-1	1.0E0
	GOTERM_BP_FAT	nucleoside triphosphate biosynthetic process	RT		17	4.6E-1	1.0E0
	GOTERM_BP_FAT	purine nucleoside triphosphate metabolic process	RT		17	4.6E-1	1.0E0
	GOTERM_BP_FAT	purine ribonucleoside triphosphate biosynthetic process	RT		17	4.6E-1	1.0E0
	GOTERM_BP_FAT	purine ribonucleoside triphosphate metabolic process	RT		17	4.6E-1	1.0E0
	GOTERM_BP_FAT	ribonucleoside triphosphate biosynthetic process	RT		17	4.6E-1	1.0E0
	GOTERM_CC_FAT	proton-transporting two-sector ATPase complex	RT		10	4.7E-1	1.0E0
	INTERPRO	ATPase, P-type, ATPase-associated region	RT		8	4.7E-1	1.0E0
	GOTERM_BP_FAT	purine nucleotide biosynthetic process	RT		18	4.8E-1	1.0E0
	GOTERM_BP_FAT	hydrogen transport	RT		10	4.9E-1	1.0E0
	GOTERM_BP_FAT	proton transport	RT		10	4.9E-1	1.0E0
	GOTERM_MF_FAT	cation-transporting ATPase activity	RT		7	5.4E-1	1.0E0
	GOTERM_MF_FAT	ATPase activity, coupled to transmembrane movement of substances	RT		17	5.5E-1	1.0E0
	GOTERM_MF_FAT	ATPase activity, coupled to movement of substances	RT		17	5.5E-1	1.0E0
	GOTERM_BP_FAT	purine nucleotide metabolic process	RT		18	5.6E-1	1.0E0
	GOTERM_MF_FAT	primary active transmembrane transporter activity	RT		18	5.7E-1	1.0E0
	GOTERM_MF_FAT	P-P-bond-hydrolysis-driven transmembrane transporter activity	RT		18	5.7E-1	1.0E0
	GOTERM_MF_FAT	hydrolase activity, acting on acid anhydrides, catalyzing transmembrane movement of substances	RT		18	5.7E-1	1.0E0
	GOTERM_BP_FAT	ion transmembrane transport	RT		9	5.9E-1	1.0E0
	GOTERM_CC_FAT	proton-transporting V-type ATPase, V0 domain	RT		4	6.0E-1	1.0E0
	GOTERM_BP_FAT	monovalent inorganic cation transport	RT		10	6.0E-1	1.0E0
	SP_PIR_KEYWORDS	Hydrogen ion transport	RT		3	6.1E-1	9.9E-1
	GOTERM_CC_FAT	proton-transporting V-type ATPase complex	RT		6	6.3E-1	1.0E0
	GOTERM_MF_FAT	proton-transporting ATPase activity, rotational mechanism	RT		5	6.4E-1	1.0E0
	GOTERM_BP_FAT	generation of precursor metabolites and energy	RT		15	6.8E-1	1.0E0
	GOTERM_BP_FAT	oxidative phosphorylation	RT		8	6.8E-1	1.0E0
	GOTERM_BP_FAT	ATP synthesis coupled proton transport	RT		8	6.8E-1	1.0E0
	GOTERM_BP_FAT	energy coupled proton transport, down electrochemical gradient	RT		8	6.8E-1	1.0E0
	GOTERM_MF_FAT	hydrogen ion transmembrane transporter activity	RT		9	7.0E-1	1.0E0
	GOTERM_MF_FAT	monovalent inorganic cation transmembrane transporter activity	RT		9	7.0E-1	1.0E0
	GOTERM_CC_FAT	proton-transporting two-sector ATPase complex, catalytic domain	RT		5	7.5E-1	1.0E0
	GOTERM_CC_FAT	proton-transporting two-sector ATPase complex, proton-transporting domain	RT		4	7.5E-1	1.0E0

SP_PIR_KEYWORDS	ion transport	RT	3	8.9E-1	9.9E-1
SP_PIR_KEYWORDS	transport	RT	6	9.2E-1	9.9E-1
Annotation Cluster 19	Enrichment Score: 0.31		Count	P_Value	Benjamini
GOTERM_MF_FAT	cysteine-type endopeptidase activity	RT	17	2.1E-1	1.0E0
GOTERM_MF_FAT	cysteine-type peptidase activity	RT	20	2.8E-1	1.0E0
SP_PIR_KEYWORDS	thiol protease	RT	8	3.2E-1	9.9E-1
GOTERM_MF_FAT	endopeptidase activity	RT	28	4.4E-1	1.0E0
INTERPRO	Peptidase, cysteine peptidase active site	RT	19	4.8E-1	1.0E0
INTERPRO	Peptidase C1A, papain C-terminal	RT	17	5.2E-1	1.0E0
INTERPRO	Peptidase C1A, papain	RT	17	5.2E-1	1.0E0
SMART	Pept_C1	RT	17	5.9E-1	1.0E0
GOTERM_BP_FAT	proteolysis	RT	49	6.8E-1	1.0E0
GOTERM_MF_FAT	peptidase activity, acting on L-amino acid peptides	RT	38	6.8E-1	1.0E0
GOTERM_MF_FAT	peptidase activity	RT	43	7.7E-1	1.0E0
SP_PIR_KEYWORDS	Protease	RT	19	8.6E-1	9.9E-1
Annotation Cluster 20	Enrichment Score: 0.29		Count	P_Value	Benjamini
INTERPRO	EGF-like, type 3	RT	22	2.4E-1	1.0E0
INTERPRO	EGF-like, laminin	RT	15	7.3E-1	1.0E0
SMART	EGF_Lam	RT	15	7.8E-1	1.0E0
Annotation Cluster 21	Enrichment Score: 0.27		Count	P_Value	Benjamini
SP_PIR_KEYWORDS	cyclin	RT	8	3.2E-1	9.9E-1
INTERPRO	Cyclin, C-terminal	RT	4	4.4E-1	1.0E0
INTERPRO	Cyclin, N-terminal	RT	5	5.0E-1	1.0E0
INTERPRO	Cyclin	RT	4	6.5E-1	1.0E0
SMART	CYCLIN	RT	4	6.7E-1	1.0E0
INTERPRO	Cyclin-related	RT	4	7.9E-1	1.0E0
Annotation Cluster 22	Enrichment Score: 0.26		Count	P_Value	Benjamini
GOTERM_BP_FAT	nuclear transport	RT	4	4.1E-1	1.0E0
GOTERM_BP_FAT	nucleocytoplasmic transport	RT	4	4.1E-1	1.0E0
GOTERM_CC_FAT	endomembrane system	RT	10	5.8E-1	1.0E0
GOTERM_CC_FAT	envelope	RT	4	6.0E-1	1.0E0
GOTERM_CC_FAT	pore complex	RT	4	6.0E-1	1.0E0
GOTERM_CC_FAT	organelle envelope	RT	4	6.0E-1	1.0E0
GOTERM_CC_FAT	nuclear envelope	RT	4	6.0E-1	1.0E0
GOTERM_CC_FAT	nuclear pore	RT	4	6.0E-1	1.0E0
Annotation Cluster 23	Enrichment Score: 0.26		Count	P_Value	Benjamini
SP_PIR_KEYWORDS	ligase	RT	36	2.1E-1	1.0E0
SP_PIR_KEYWORDS	protein biosynthesis	RT	8	3.2E-1	9.9E-1
KEGG_PATHWAY	Aminoacyl-tRNA biosynthesis	RT	14	3.3E-1	1.0E0
GOTERM_BP_FAT	tRNA aminoacylation for protein translation	RT	14	4.8E-1	1.0E0
GOTERM_BP_FAT	amino acid activation	RT	14	5.8E-1	1.0E0
GOTERM_BP_FAT	tRNA aminoacylation	RT	14	5.8E-1	1.0E0
SP_PIR_KEYWORDS	Aminoacyl-tRNA synthetase	RT	14	6.2E-1	9.9E-1
GOTERM_MF_FAT	ligase activity, forming aminoacyl-tRNA and related compounds	RT	14	6.7E-1	1.0E0
GOTERM_MF_FAT	ligase activity, forming carbon-oxygen bonds	RT	14	6.7E-1	1.0E0
GOTERM_MF_FAT	aminoacyl-tRNA ligase activity	RT	14	6.7E-1	1.0E0
INTERPRO	Aminoacyl-tRNA synthetase, class II, conserved region	RT	6	6.9E-1	1.0E0
GOTERM_BP_FAT	tRNA metabolic process	RT	20	9.0E-1	1.0E0
GOTERM_BP_FAT	ncRNA metabolic process	RT	25	9.5E-1	1.0E0
Annotation Cluster 24	Enrichment Score: 0.25		Count	P_Value	Benjamini
GOTERM_CC_FAT	non-membrane-bounded organelle	RT	94	1.4E-1	9.8E-1
GOTERM_CC_FAT	intracellular non-membrane-bounded organelle	RT	94	1.4E-1	9.8E-1
GOTERM_MF_FAT	structural molecule activity	RT	46	7.1E-1	1.0E0
KEGG_PATHWAY	Ribosome	RT	36	7.7E-1	1.0E0
GOTERM_CC_FAT	ribosome	RT	40	7.9E-1	1.0E0
GOTERM_BP_FAT	translation	RT	60	8.2E-1	1.0E0
GOTERM_CC_FAT	ribonucleoprotein complex	RT	52	8.6E-1	1.0E0

GOTERM_MF_FAT	structural constituent of ribosome	RT			35	9.1E-1	1.0E0
SP_PIR_KEYWORDS	ribosomal protein	RT			37	9.2E-1	9.9E-1
Annotation Cluster 25	Enrichment Score: 0.24				Count	P_Value	Benjamini
GOTERM_BP_FAT	cell cycle	RT			7	2.4E-1	1.0E0
GOTERM_BP_FAT	M phase	RT			5	4.7E-1	1.0E0
GOTERM_BP_FAT	cell cycle process	RT			5	4.7E-1	1.0E0
GOTERM_BP_FAT	cell cycle phase	RT			5	4.7E-1	1.0E0
GOTERM_BP_FAT	M phase of mitotic cell cycle	RT			3	7.8E-1	1.0E0
GOTERM_BP_FAT	mitotic cell cycle	RT			3	7.8E-1	1.0E0
GOTERM_BP_FAT	nuclear division	RT			3	7.8E-1	1.0E0
GOTERM_BP_FAT	organelle fission	RT			3	7.8E-1	1.0E0
GOTERM_BP_FAT	mitosis	RT			3	7.8E-1	1.0E0
Annotation Cluster 26	Enrichment Score: 0.23				Count	P_Value	Benjamini
PIR_SUPERFAMILY	PIRSF000908:serine/threonine-protein phosphatase, conventional type	RT			7	2.8E-1	1.0E0
INTERPRO	Metallophosphoesterase	RT			14	4.4E-1	1.0E0
INTERPRO	Serine/threonine-specific protein phosphatase and bis(5-nucleosyl)-tetraphosphatase	RT			10	5.4E-1	1.0E0
SMART	PP2Ac	RT			10	5.9E-1	1.0E0
GOTERM_MF_FAT	phosphatase activity	RT			25	6.6E-1	1.0E0
GOTERM_MF_FAT	phosphoprotein phosphatase activity	RT			16	7.0E-1	1.0E0
SP_PIR_KEYWORDS	iron	RT			11	8.2E-1	9.9E-1
GOTERM_MF_FAT	iron ion binding	RT			15	9.3E-1	1.0E0
Annotation Cluster 27	Enrichment Score: 0.23				Count	P_Value	Benjamini
GOTERM_MF_FAT	ATPase activity, coupled to transmembrane movement of ions, phosphorylative mechanism	RT			9	3.4E-1	1.0E0
INTERPRO	ATPase, P-type, K/Mg/Cd/Cu/Zn/Na/Ca/Na/H-transporter	RT			9	3.8E-1	1.0E0
GOTERM_MF_FAT	lipid transporter activity	RT			5	4.7E-1	1.0E0
INTERPRO	ATPase, P-type, ATPase-associated region	RT			8	4.7E-1	1.0E0
INTERPRO	Haloacid dehalogenase-like hydrolase	RT			7	5.8E-1	1.0E0
GOTERM_MF_FAT	di-, tri-valent inorganic cation transmembrane transporter activity	RT			3	6.0E-1	1.0E0
GOTERM_BP_FAT	lipid transport	RT			4	6.2E-1	1.0E0
GOTERM_BP_FAT	lipid localization	RT			4	6.2E-1	1.0E0
GOTERM_MF_FAT	metal ion transmembrane transporter activity	RT			4	6.2E-1	1.0E0
GOTERM_MF_FAT	aminophospholipid transporter activity	RT			4	6.2E-1	1.0E0
GOTERM_MF_FAT	phospholipid transporter activity	RT			4	6.2E-1	1.0E0
GOTERM_MF_FAT	phospholipid-translocating ATPase activity	RT			4	6.2E-1	1.0E0
GOTERM_MF_FAT	magnesium ion binding	RT			9	7.0E-1	1.0E0
GOTERM_BP_FAT	phospholipid transport	RT			3	7.8E-1	1.0E0
INTERPRO	ATPase, P-type phosphorylation site	RT			5	7.9E-1	1.0E0
INTERPRO	ATPase, P-type, phospholipid-translocating, flippase	RT			3	8.0E-1	1.0E0
Annotation Cluster 28	Enrichment Score: 0.23				Count	P_Value	Benjamini
SP_PIR_KEYWORDS	protein biosynthesis	RT			8	3.2E-1	9.9E-1
INTERPRO	Aminoacyl-tRNA synthetase, class II (D, K and N)	RT			3	6.2E-1	1.0E0
INTERPRO	Aminoacyl-tRNA synthetase, class II (D, K and N)-like	RT			3	6.2E-1	1.0E0
PIR_SUPERFAMILY	PIRSF001525:lysine-tRNA ligase	RT			3	6.2E-1	1.0E0
INTERPRO	Aminoacyl-tRNA synthetase, class II, conserved region	RT			6	6.9E-1	1.0E0
INTERPRO	Nucleic acid binding, OB-fold, tRNA/helicase-type	RT			3	8.0E-1	1.0E0
Annotation Cluster 29	Enrichment Score: 0.22				Count	P_Value	Benjamini
INTERPRO	Haloacid dehalogenase-like hydrolase	RT			7	5.8E-1	1.0E0
GOTERM_MF_FAT	phosphoglycolate phosphatase activity	RT			3	6.0E-1	1.0E0
INTERPRO	HAD-superfamily hydrolase, subfamily IA, variant 1	RT			3	6.2E-1	1.0E0
Annotation Cluster 30	Enrichment Score: 0.19				Count	P_Value	Benjamini
GOTERM_CC_FAT	microtubule cytoskeleton	RT			31	4.2E-1	1.0E0
GOTERM_CC_FAT	cytoskeletal part	RT			31	4.2E-1	1.0E0
GOTERM_CC_FAT	cytoskeleton	RT			32	4.4E-1	1.0E0
GOTERM_BP_FAT	microtubule-based process	RT			32	4.9E-1	1.0E0
	microtubule	RT			19	6.2E-1	1.0E0

Category	Term		Count	P_Value	Benjamini
GOTERM_CC_FAT					
SP_PIR_KEYWORDS	microtubule	RT	19	6.3E-1	9.9E-1
GOTERM_BP_FAT	microtubule-based movement	RT	26	7.2E-1	1.0E0
GOTERM_MF_FAT	motor activity	RT	23	7.6E-1	1.0E0
GOTERM_MF_FAT	microtubule motor activity	RT	23	7.6E-1	1.0E0
INTERPRO	Kinesin, motor region, conserved site	RT	10	8.2E-1	1.0E0
SP_PIR_KEYWORDS	motor protein	RT	13	8.3E-1	9.9E-1
INTERPRO	Kinesin, motor region	RT	15	8.5E-1	1.0E0
SMART	KISc	RT	15	8.9E-1	1.0E0
Annotation Cluster 31	Enrichment Score: 0.19		Count	P_Value	Benjamini
GOTERM_MF_FAT	phosphoinositide binding	RT	4	6.2E-1	1.0E0
INTERPRO	Phox-like	RT	4	6.5E-1	1.0E0
SMART	PX	RT	4	6.7E-1	1.0E0
Annotation Cluster 32	Enrichment Score: 0.19		Count	P_Value	Benjamini
GOTERM_MF_FAT	zinc ion binding	RT	88	4.2E-1	1.0E0
GOTERM_MF_FAT	ion binding	RT	146	5.4E-1	1.0E0
GOTERM_MF_FAT	cation binding	RT	146	5.4E-1	1.0E0
GOTERM_MF_FAT	transition metal ion binding	RT	106	5.7E-1	1.0E0
GOTERM_MF_FAT	metal ion binding	RT	143	6.1E-1	1.0E0
SP_PIR_KEYWORDS	zinc	RT	48	6.5E-1	9.9E-1
SP_PIR_KEYWORDS	zinc-finger	RT	35	6.6E-1	9.8E-1
INTERPRO	Zinc finger, RING-type	RT	38	7.2E-1	1.0E0
SMART	RING	RT	38	8.0E-1	1.0E0
SP_PIR_KEYWORDS	metal-binding	RT	54	8.5E-1	9.9E-1
INTERPRO	Zinc finger, C3HC4 RING-type	RT	17	9.5E-1	1.0E0
Annotation Cluster 33	Enrichment Score: 0.18		Count	P_Value	Benjamini
COG_ONTOLOGY	Nucleotide transport and metabolism	RT	6	4.3E-1	1.0E0
INTERPRO	5'-Nucleotidase and apyrase	RT	3	6.2E-1	1.0E0
INTERPRO	Predicted phosphoesterase, C1039.02 type	RT	3	6.2E-1	1.0E0
PIR_SUPERFAMILY	PIRSF017316:phosphoesterase, C1039.02 type	RT	3	6.2E-1	1.0E0
PIR_SUPERFAMILY	PIRSF017316:Pesterase_C1039	RT	3	6.2E-1	1.0E0
GOTERM_BP_FAT	nitrogen compound catabolic process	RT	4	7.7E-1	1.0E0
GOTERM_BP_FAT	nucleobase, nucleoside and nucleotide catabolic process	RT	4	7.7E-1	1.0E0
GOTERM_BP_FAT	nucleobase, nucleoside, nucleotide and nucleic acid catabolic process	RT	4	7.7E-1	1.0E0
GOTERM_BP_FAT	nucleotide catabolic process	RT	3	7.8E-1	1.0E0
Annotation Cluster 34	Enrichment Score: 0.18		Count	P_Value	Benjamini
GOTERM_MF_FAT	ligase activity, forming carbon-nitrogen bonds	RT	21	3.1E-1	1.0E0
GOTERM_MF_FAT	acid-amino acid ligase activity	RT	17	4.6E-1	1.0E0
GOTERM_MF_FAT	small conjugating protein ligase activity	RT	8	6.8E-1	1.0E0
SP_PIR_KEYWORDS	ubl conjugation pathway	RT	8	7.1E-1	9.9E-1
INTERPRO	Ubiquitin-conjugating enzyme, E2	RT	7	8.0E-1	1.0E0
INTERPRO	Ubiquitin-conjugating enzyme/RWD-like	RT	7	8.0E-1	1.0E0
SMART	UBCc	RT	7	8.3E-1	1.0E0
PIR_SUPERFAMILY	PIRSF001567:ubiquitin-protein ligase E2	RT	3	9.5E-1	1.0E0
Annotation Cluster 35	Enrichment Score: 0.17		Count	P_Value	Benjamini
GOTERM_MF_FAT	guanyl ribonucleotide binding	RT	29	4.6E-1	1.0E0
GOTERM_MF_FAT	GTP binding	RT	29	4.6E-1	1.0E0
GOTERM_MF_FAT	guanyl nucleotide binding	RT	29	4.6E-1	1.0E0
SP_PIR_KEYWORDS	gtp-binding	RT	24	5.6E-1	9.9E-1
GOTERM_MF_FAT	GTPase activity	RT	10	7.1E-1	1.0E0
GOTERM_BP_FAT	small GTPase mediated signal transduction	RT	9	7.8E-1	1.0E0
INTERPRO	Small GTP-binding protein	RT	11	8.3E-1	1.0E0
INTERPRO	Ras GTPase	RT	7	8.7E-1	1.0E0
INTERPRO	Ras	RT	6	9.2E-1	1.0E0
GOTERM_BP_FAT	intracellular signaling cascade	RT	10	9.3E-1	1.0E0
Annotation Cluster 36	Enrichment Score: 0.15		Count	P_Value	Benjamini
INTERPRO	Clathrin/coatomer adaptor, adaptin-like, N-terminal	RT	5	3.0E-1	1.0E0

GOTERM_CC_FAT	organelle membrane	RT	8	4.1E-1	1.0E0
GOTERM_CC_FAT	coated membrane	RT	9	5.6E-1	1.0E0
GOTERM_CC_FAT	membrane coat	RT	9	5.6E-1	1.0E0
GOTERM_CC_FAT	endomembrane system	RT	10	5.8E-1	1.0E0
GOTERM_CC_FAT	Golgi apparatus part	RT	5	7.5E-1	1.0E0
GOTERM_CC_FAT	vesicle membrane	RT	4	7.5E-1	1.0E0
GOTERM_CC_FAT	cytoplasmic vesicle part	RT	4	7.5E-1	1.0E0
GOTERM_CC_FAT	vesicle	RT	4	7.5E-1	1.0E0
GOTERM_CC_FAT	cytoplasmic vesicle	RT	4	7.5E-1	1.0E0
GOTERM_CC_FAT	cytoplasmic vesicle membrane	RT	4	7.5E-1	1.0E0
GOTERM_CC_FAT	coated vesicle membrane	RT	4	7.5E-1	1.0E0
GOTERM_CC_FAT	membrane-bounded vesicle	RT	4	7.5E-1	1.0E0
GOTERM_CC_FAT	coated vesicle	RT	4	7.5E-1	1.0E0
GOTERM_CC_FAT	vesicle coat	RT	4	7.5E-1	1.0E0
GOTERM_CC_FAT	cytoplasmic membrane-bounded vesicle	RT	4	7.5E-1	1.0E0
GOTERM_CC_FAT	Golgi apparatus	RT	6	8.4E-1	1.0E0
GOTERM_BP_FAT	vesicle-mediated transport	RT	15	8.6E-1	1.0E0
GOTERM_CC_FAT	transport vesicle membrane	RT	3	8.8E-1	1.0E0
GOTERM_CC_FAT	transport vesicle	RT	3	8.8E-1	1.0E0
GOTERM_BP_FAT	Golgi vesicle transport	RT	3	9.4E-1	1.0E0
GOTERM_BP_FAT	ER to Golgi vesicle-mediated transport	RT	3	9.4E-1	1.0E0
Annotation Cluster 37	Enrichment Score: 0.15		Count	P_Value	Benjamini
GOTERM_BP_FAT	carbohydrate biosynthetic process	RT	5	6.4E-1	1.0E0
GOTERM_BP_FAT	cellular carbohydrate biosynthetic process	RT	5	6.4E-1	1.0E0
GOTERM_BP_FAT	alcohol biosynthetic process	RT	3	8.8E-1	1.0E0
Annotation Cluster 38	Enrichment Score: 0.14		Count	P_Value	Benjamini
INTERPRO	Pyridine nucleotide-disulphide oxidoreductase, NAD-binding region	RT	3	6.2E-1	1.0E0
GOTERM_MF_FAT	FAD binding	RT	4	7.7E-1	1.0E0
INTERPRO	FAD-dependent pyridine nucleotide-disulphide oxidoreductase	RT	3	8.0E-1	1.0E0
Annotation Cluster 39	Enrichment Score: 0.14		Count	P_Value	Benjamini
GOTERM_BP_FAT	regulation of transcription	RT	18	6.4E-1	1.0E0
GOTERM_BP_FAT	regulation of RNA metabolic process	RT	7	7.7E-1	1.0E0
GOTERM_BP_FAT	regulation of transcription, DNA-dependent	RT	7	7.7E-1	1.0E0
Annotation Cluster 40	Enrichment Score: 0.13		Count	P_Value	Benjamini
INTERPRO	WD40 repeat	RT	43	6.3E-1	1.0E0
INTERPRO	WD40 repeat, region	RT	27	7.0E-1	1.0E0
INTERPRO	WD40/YVTN repeat-like	RT	41	7.0E-1	1.0E0
SMART	WD40	RT	43	7.2E-1	1.0E0
INTERPRO	WD40 repeat 2	RT	24	7.7E-1	1.0E0
SP_PIR_KEYWORDS	wd repeat	RT	27	7.8E-1	9.9E-1
INTERPRO	WD40 repeat, subgroup	RT	31	7.9E-1	1.0E0
INTERPRO	WD40 repeat, conserved site	RT	14	9.5E-1	1.0E0
Annotation Cluster 41	Enrichment Score: 0.12		Count	P_Value	Benjamini
GOTERM_MF_FAT	electron carrier activity	RT	16	2.6E-1	1.0E0
INTERPRO	4Fe-4S ferredoxin, iron-sulphur binding, subgroup	RT	5	8.8E-1	1.0E0
INTERPRO	4Fe-4S ferredoxin, iron-sulpur binding domain	RT	5	8.8E-1	1.0E0
INTERPRO	4Fe-4S ferredoxin, iron-sulphur binding, conserved site	RT	4	8.8E-1	1.0E0
GOTERM_MF_FAT	4 iron, 4 sulfur cluster binding	RT	4	9.3E-1	1.0E0
GOTERM_MF_FAT	iron-sulfur cluster binding	RT	9	9.6E-1	1.0E0
GOTERM_MF_FAT	metal cluster binding	RT	9	9.6E-1	1.0E0
Annotation Cluster 42	Enrichment Score: 0.11		Count	P_Value	Benjamini
INTERPRO	Serine/threonine protein kinase-related	RT	135	5.4E-1	1.0E0
INTERPRO	Protein kinase, ATP binding site	RT	48	5.9E-1	1.0E0
GOTERM_MF_FAT	protein serine/threonine kinase activity	RT	57	7.5E-1	1.0E0
INTERPRO	Serine/threonine protein kinase, active site	RT	48	8.6E-1	1.0E0
SP_PIR_KEYWORDS	serine/threonine-protein kinase	RT	32	8.8E-1	9.9E-1
INTERPRO	Serine/threonine protein kinase	RT	30	9.1E-1	1.0E0

SMART	S_TKc	RT			30	9.5E-1	1.0E0
Annotation Cluster 43	Enrichment Score: 0.11				Count	P_Value	Benjamini
GOTERM_BP_FAT	vitamin metabolic process	RT			4	6.2E-1	1.0E0
GOTERM_BP_FAT	water-soluble vitamin metabolic process	RT			4	6.2E-1	1.0E0
GOTERM_BP_FAT	vitamin biosynthetic process	RT			4	6.2E-1	1.0E0
GOTERM_BP_FAT	water-soluble vitamin biosynthetic process	RT			4	6.2E-1	1.0E0
GOTERM_BP_FAT	coenzyme metabolic process	RT			6	7.7E-1	1.0E0
GOTERM_BP_FAT	oxidoreduction coenzyme metabolic process	RT			4	7.7E-1	1.0E0
GOTERM_BP_FAT	pyridine nucleotide metabolic process	RT			4	7.7E-1	1.0E0
GOTERM_BP_FAT	cofactor metabolic process	RT			7	7.7E-1	1.0E0
GOTERM_BP_FAT	pyridine nucleotide biosynthetic process	RT			3	7.8E-1	1.0E0
GOTERM_BP_FAT	cofactor biosynthetic process	RT			5	8.5E-1	1.0E0
GOTERM_BP_FAT	coenzyme biosynthetic process	RT			4	8.6E-1	1.0E0
GOTERM_BP_FAT	secondary metabolic process	RT			3	8.8E-1	1.0E0
GOTERM_BP_FAT	nicotinamide nucleotide metabolic process	RT			3	8.8E-1	1.0E0
GOTERM_BP_FAT	nicotinamide metabolic process	RT			3	8.8E-1	1.0E0
GOTERM_BP_FAT	alkaloid metabolic process	RT			3	8.8E-1	1.0E0
GOTERM_BP_FAT	cellular amide metabolic process	RT			3	8.8E-1	1.0E0
Annotation Cluster 44	Enrichment Score: 0.11				Count	P_Value	Benjamini
GOTERM_BP_FAT	cellular amino acid biosynthetic process	RT			4	6.2E-1	1.0E0
GOTERM_BP_FAT	amine biosynthetic process	RT			4	7.7E-1	1.0E0
GOTERM_BP_FAT	organic acid biosynthetic process	RT			4	8.6E-1	1.0E0
GOTERM_BP_FAT	carboxylic acid biosynthetic process	RT			4	8.6E-1	1.0E0
Annotation Cluster 45	Enrichment Score: 0.11				Count	P_Value	Benjamini
INTERPRO	Chaperonin TCP-1, conserved site	RT			6	6.9E-1	1.0E0
INTERPRO	Chaperonin Cpn60/TCP-1	RT			6	6.9E-1	1.0E0
INTERPRO	Chaperone, tailless complex polypeptide 1	RT			5	7.9E-1	1.0E0
SP_PIR_KEYWORDS	Chaperone	RT			7	7.9E-1	9.9E-1
PIR_SUPERFAMILY	PIRSF002584:molecular chaperone t-complex-type	RT			5	8.0E-1	1.0E0
SP_PIR_KEYWORDS	cytoplasm	RT			10	8.1E-1	9.9E-1
GOTERM_MF_FAT	unfolded protein binding	RT			11	8.5E-1	1.0E0
Annotation Cluster 46	Enrichment Score: 0.11				Count	P_Value	Benjamini
GOTERM_CC_FAT	spindle pole	RT			3	7.7E-1	1.0E0
GOTERM_CC_FAT	spindle	RT			3	7.7E-1	1.0E0
GOTERM_CC_FAT	microtubule organizing center	RT			3	7.7E-1	1.0E0
GOTERM_BP_FAT	microtubule cytoskeleton organization	RT			3	7.8E-1	1.0E0
INTERPRO	Spc97/Spc98	RT			3	8.0E-1	1.0E0
Annotation Cluster 47	Enrichment Score: 0.11				Count	P_Value	Benjamini
GOTERM_BP_FAT	secretion by cell	RT			3	7.8E-1	1.0E0
GOTERM_BP_FAT	exocytosis	RT			3	7.8E-1	1.0E0
GOTERM_BP_FAT	vesicle docking during exocytosis	RT			3	7.8E-1	1.0E0
GOTERM_BP_FAT	vesicle docking	RT			3	7.8E-1	1.0E0
GOTERM_BP_FAT	secretion	RT			3	7.8E-1	1.0E0
GOTERM_BP_FAT	membrane docking	RT			3	7.8E-1	1.0E0
INTERPRO	Sec1-like protein	RT			3	8.0E-1	1.0E0
Annotation Cluster 48	Enrichment Score: 0.11				Count	P_Value	Benjamini
INTERPRO	Aminotransferase, class V/Cysteine desulfurase	RT			3	6.2E-1	1.0E0
GOTERM_MF_FAT	vitamin B6 binding	RT			6	7.7E-1	1.0E0
GOTERM_MF_FAT	pyridoxal phosphate binding	RT			6	7.7E-1	1.0E0
GOTERM_MF_FAT	vitamin binding	RT			6	8.5E-1	1.0E0
INTERPRO	Pyridoxal phosphate-dependent transferase, major region, subdomain 1	RT			4	9.4E-1	1.0E0
Annotation Cluster 49	Enrichment Score: 0.11				Count	P_Value	Benjamini
GOTERM_MF_FAT	RNA polymerase activity	RT			15	5.1E-1	1.0E0
GOTERM_MF_FAT	DNA-directed RNA polymerase activity	RT			14	5.9E-1	1.0E0
SP_PIR_KEYWORDS	dna-directed rna polymerase	RT			9	6.2E-1	9.9E-1
KEGG_PATHWAY	RNA polymerase	RT			9	8.7E-1	1.0E0
SP_PIR_KEYWORDS	nucleotidyltransferase	RT			13	8.8E-1	9.9E-1
GOTERM_BP_FAT	transcription	RT			13	8.9E-1	1.0E0

KEGG_PATHWAY	Pyrimidine metabolism	RT	15	9.5E-1	1.0E0
GOTERM_MF_FAT	nucleotidyltransferase activity	RT	35	9.6E-1	1.0E0
KEGG_PATHWAY	Purine metabolism	RT	14	9.7E-1	1.0E0
Annotation Cluster 50	Enrichment Score: 0.1		Count	P_Value	Benjamini
SP_PIR_KEYWORDS	transmembrane	RT	17	7.4E-1	9.9E-1
SP_PIR_KEYWORDS	membrane	RT	15	7.9E-1	9.9E-1
INTERPRO	Amino acid transporter, transmembrane	RT	6	8.0E-1	1.0E0
GOTERM_CC_FAT	intrinsic to membrane	RT	35	8.1E-1	1.0E0
GOTERM_CC_FAT	integral to membrane	RT	35	8.1E-1	1.0E0
Annotation Cluster 51	Enrichment Score: 0.1		Count	P_Value	Benjamini
GOTERM_MF_FAT	thiolester hydrolase activity	RT	5	7.7E-1	1.0E0
GOTERM_MF_FAT	ubiquitin thiolesterase activity	RT	4	7.7E-1	1.0E0
INTERPRO	Peptidase C19, ubiquitin carboxyl-terminal hydrolase 2	RT	4	7.9E-1	1.0E0
INTERPRO	Peptidase C19, ubiquitin carboxyl-terminal hydrolase 2, conserved site	RT	3	8.0E-1	1.0E0
GOTERM_BP_FAT	ubiquitin-dependent protein catabolic process	RT	7	9.0E-1	1.0E0
Annotation Cluster 52	Enrichment Score: 0.09		Count	P_Value	Benjamini
INTERPRO	AMP-dependent synthetase and ligase	RT	5	6.7E-1	1.0E0
COG_ONTOLOGY	Lipid metabolism	RT	7	7.8E-1	1.0E0
GOTERM_MF_FAT	long-chain-fatty-acid-CoA ligase activity	RT	3	8.8E-1	1.0E0
GOTERM_MF_FAT	fatty-acid ligase activity	RT	3	8.8E-1	1.0E0
GOTERM_MF_FAT	ligase activity, forming carbon-sulfur bonds	RT	3	8.8E-1	1.0E0
Annotation Cluster 53	Enrichment Score: 0.09		Count	P_Value	Benjamini
KEGG_PATHWAY	Glycolysis / Gluconeogenesis	RT	5	6.0E-1	1.0E0
GOTERM_BP_FAT	hexose metabolic process	RT	12	6.4E-1	1.0E0
GOTERM_BP_FAT	glucose metabolic process	RT	12	6.4E-1	1.0E0
GOTERM_BP_FAT	monosaccharide metabolic process	RT	13	7.3E-1	1.0E0
GOTERM_BP_FAT	hexose catabolic process	RT	7	9.0E-1	1.0E0
GOTERM_BP_FAT	carbohydrate catabolic process	RT	7	9.0E-1	1.0E0
GOTERM_BP_FAT	alcohol catabolic process	RT	7	9.0E-1	1.0E0
GOTERM_BP_FAT	monosaccharide catabolic process	RT	7	9.0E-1	1.0E0
GOTERM_BP_FAT	cellular carbohydrate catabolic process	RT	7	9.0E-1	1.0E0
GOTERM_BP_FAT	glucose catabolic process	RT	7	9.0E-1	1.0E0
SP_PIR_KEYWORDS	glycolysis	RT	3	9.5E-1	1.0E0
GOTERM_BP_FAT	glycolysis	RT	5	9.5E-1	1.0E0
Annotation Cluster 54	Enrichment Score: 0.08		Count	P_Value	Benjamini
GOTERM_CC_FAT	microtubule associated complex	RT	9	6.7E-1	1.0E0
GOTERM_CC_FAT	dynein complex	RT	6	9.0E-1	1.0E0
INTERPRO	Dynein heavy chain	RT	6	9.2E-1	1.0E0
Annotation Cluster 55	Enrichment Score: 0.08		Count	P_Value	Benjamini
INTERPRO	Kelch repeat type 1	RT	4	7.9E-1	1.0E0
SMART	Kelch	RT	4	8.1E-1	1.0E0
INTERPRO	Kelch-type beta propeller	RT	4	8.8E-1	1.0E0
Annotation Cluster 56	Enrichment Score: 0.08		Count	P_Value	Benjamini
GOTERM_BP_FAT	proteolysis	RT	49	6.8E-1	1.0E0
GOTERM_BP_FAT	macromolecule catabolic process	RT	22	6.8E-1	1.0E0
SP_PIR_KEYWORDS	ubl conjugation pathway	RT	8	7.1E-1	9.9E-1
GOTERM_BP_FAT	cellular macromolecule catabolic process	RT	19	7.2E-1	1.0E0
GOTERM_BP_FAT	protein catabolic process	RT	21	7.4E-1	1.0E0
KEGG_PATHWAY	Proteasome	RT	15	7.7E-1	1.0E0
GOTERM_BP_FAT	proteolysis involved in cellular protein catabolic process	RT	18	7.7E-1	1.0E0
GOTERM_BP_FAT	cellular protein catabolic process	RT	18	7.7E-1	1.0E0
GOTERM_BP_FAT	modification-dependent protein catabolic process	RT	14	8.0E-1	1.0E0
GOTERM_BP_FAT	modification-dependent macromolecule catabolic process	RT	14	8.0E-1	1.0E0
GOTERM_CC_FAT	proteasome complex	RT	11	8.4E-1	1.0E0
INTERPRO	Proteasome, alpha and beta subunits	RT	7	8.7E-1	1.0E0
GOTERM_CC_FAT	proteasome core complex	RT	7	8.9E-1	1.0E0
GOTERM_BP_FAT	ubiquitin-dependent protein catabolic process	RT	7	9.0E-1	1.0E0

Category	Term		Count	P_Value	Benjamini
GOTERM_MF_FAT	threonine-type peptidase activity	RT	7	9.0E-1	1.0E0
GOTERM_MF_FAT	threonine-type endopeptidase activity	RT	7	9.0E-1	1.0E0
SP_PIR_KEYWORDS	proteasome	RT	10	9.1E-1	9.9E-1
INTERPRO	Proteasome, subunit alpha/beta	RT	7	9.2E-1	1.0E0
SP_PIR_KEYWORDS	threonine protease	RT	3	9.5E-1	1.0E0
PIR_SUPERFAMILY	PIRSF001212:multicatalytic endopeptidase complex chain C9	RT	3	9.5E-1	1.0E0
GOTERM_CC_FAT	cytosol	RT	13	9.6E-1	1.0E0
INTERPRO	Proteasome, alpha-subunit, conserved site	RT	3	9.8E-1	1.0E0
Annotation Cluster 57	Enrichment Score: 0.08		Count	P_Value	Benjamini
GOTERM_BP_FAT	small GTPase mediated signal transduction	RT	9	7.8E-1	1.0E0
INTERPRO	ADP-ribosylation factor	RT	3	8.0E-1	1.0E0
SMART	ARF	RT	3	8.1E-1	1.0E0
INTERPRO	ARF/SAR superfamily	RT	4	8.8E-1	1.0E0
PIR_SUPERFAMILY	PIRSF001711:ADP-ribosylation factor	RT	3	9.0E-1	1.0E0
Annotation Cluster 58	Enrichment Score: 0.08		Count	P_Value	Benjamini
GOTERM_MF_FAT	cAMP-dependent protein kinase regulator activity	RT	3	7.8E-1	1.0E0
GOTERM_MF_FAT	protein kinase regulator activity	RT	4	8.7E-1	1.0E0
GOTERM_MF_FAT	kinase regulator activity	RT	4	8.7E-1	1.0E0
Annotation Cluster 59	Enrichment Score: 0.07		Count	P_Value	Benjamini
INTERPRO	ATPase, AAA-type, core	RT	14	7.9E-1	1.0E0
INTERPRO	ATPase, AAA+ type, core	RT	38	8.7E-1	1.0E0
SMART	AAA	RT	38	9.2E-1	1.0E0
Annotation Cluster 60	Enrichment Score: 0.06		Count	P_Value	Benjamini
GOTERM_MF_FAT	translation factor activity, nucleic acid binding	RT	17	8.2E-1	1.0E0
SP_PIR_KEYWORDS	Initiation factor	RT	10	8.7E-1	9.9E-1
GOTERM_MF_FAT	translation initiation factor activity	RT	12	9.3E-1	1.0E0
Annotation Cluster 61	Enrichment Score: 0.06		Count	P_Value	Benjamini
INTERPRO	Zinc finger, C2H2-like	RT	10	8.2E-1	1.0E0
SMART	ZnF_C2H2	RT	10	8.5E-1	1.0E0
INTERPRO	Zinc finger, C2H2-type	RT	8	9.7E-1	1.0E0
Annotation Cluster 62	Enrichment Score: 0.05		Count	P_Value	Benjamini
GOTERM_MF_FAT	phosphoprotein phosphatase activity	RT	16	7.0E-1	1.0E0
GOTERM_MF_FAT	protein tyrosine phosphatase activity	RT	4	8.7E-1	1.0E0
INTERPRO	Dual-specific/protein-tyrosine phosphatase, conserved region	RT	3	9.0E-1	1.0E0
INTERPRO	Protein-tyrosine phosphatase	RT	3	9.0E-1	1.0E0
GOTERM_BP_FAT	protein amino acid dephosphorylation	RT	6	9.4E-1	1.0E0
INTERPRO	Protein-tyrosine phosphatase, active site	RT	3	9.5E-1	1.0E0
GOTERM_BP_FAT	dephosphorylation	RT	7	9.6E-1	1.0E0
Annotation Cluster 63	Enrichment Score: 0.05		Count	P_Value	Benjamini
GOTERM_BP_FAT	mismatch repair	RT	3	8.8E-1	1.0E0
GOTERM_MF_FAT	double-stranded DNA binding	RT	3	8.8E-1	1.0E0
GOTERM_MF_FAT	structure-specific DNA binding	RT	3	8.8E-1	1.0E0
GOTERM_MF_FAT	mismatched DNA binding	RT	3	8.8E-1	1.0E0
Annotation Cluster 64	Enrichment Score: 0.05		Count	P_Value	Benjamini
GOTERM_BP_FAT	isoprenoid metabolic process	RT	3	8.8E-1	1.0E0
GOTERM_BP_FAT	isoprenoid biosynthetic process	RT	3	8.8E-1	1.0E0
GOTERM_BP_FAT	lipid biosynthetic process	RT	7	9.0E-1	1.0E0
Annotation Cluster 65	Enrichment Score: 0.05		Count	P_Value	Benjamini
GOTERM_MF_FAT	nucleoside-triphosphatase regulator activity	RT	8	6.8E-1	1.0E0
GOTERM_MF_FAT	small GTPase regulator activity	RT	6	8.5E-1	1.0E0
GOTERM_MF_FAT	GTPase regulator activity	RT	6	8.5E-1	1.0E0
GOTERM_BP_FAT	regulation of small GTPase mediated signal transduction	RT	5	8.5E-1	1.0E0
GOTERM_BP_FAT	regulation of Ras protein signal transduction	RT	5	8.5E-1	1.0E0
GOTERM_BP_FAT	regulation of ARF protein signal transduction	RT	3	8.8E-1	1.0E0
GOTERM_MF_FAT	enzyme activator activity	RT	4	9.6E-1	1.0E0
GOTERM_BP_FAT	regulation of Ras GTPase activity	RT	3	9.7E-1	1.0E0
GOTERM_MF_FAT	GTPase activator activity	RT	3	9.7E-1	1.0E0
GOTERM_BP_FAT	regulation of GTPase activity	RT	3	9.9E-1	1.0E0

GOTERM_BP_FAT	regulation of hydrolase activity	RT	3	9.9E-1	1.0E0
Annotation Cluster 66	Enrichment Score: 0.05		Count	P_Value	Benjamini
INTERPRO	Thioredoxin, conserved site	RT	4	7.9E-1	1.0E0
INTERPRO	Thioredoxin domain	RT	4	7.9E-1	1.0E0
INTERPRO	Thioredoxin, core	RT	4	8.8E-1	1.0E0
INTERPRO	Thioredoxin-like subdomain	RT	3	9.0E-1	1.0E0
GOTERM_BP_FAT	cell redox homeostasis	RT	9	9.0E-1	1.0E0
INTERPRO	Thioredoxin fold	RT	11	9.2E-1	1.0E0
GOTERM_BP_FAT	cellular homeostasis	RT	9	9.3E-1	1.0E0
GOTERM_BP_FAT	homeostatic process	RT	9	9.6E-1	1.0E0
INTERPRO	Thioredoxin-like	RT	5	9.9E-1	1.0E0
Annotation Cluster 67	Enrichment Score: 0.05		Count	P_Value	Benjamini
GOTERM_BP_FAT	response to drug	RT	3	8.8E-1	1.0E0
GOTERM_BP_FAT	multidrug transport	RT	3	8.8E-1	1.0E0
GOTERM_BP_FAT	drug transport	RT	3	8.8E-1	1.0E0
GOTERM_MF_FAT	drug transporter activity	RT	3	8.8E-1	1.0E0
INTERPRO	Multi antimicrobial extrusion protein MatE	RT	3	9.0E-1	1.0E0
GOTERM_MF_FAT	antiporter activity	RT	3	9.4E-1	1.0E0
Annotation Cluster 68	Enrichment Score: 0.05		Count	P_Value	Benjamini
GOTERM_BP_FAT	RNA-dependent DNA replication	RT	4	8.6E-1	1.0E0
INTERPRO	RNA-directed DNA polymerase (reverse transcriptase)	RT	4	8.8E-1	1.0E0
INTERPRO	RNA-directed DNA polymerase (reverse transcriptase), related	RT	3	9.0E-1	1.0E0
GOTERM_MF_FAT	RNA-directed DNA polymerase activity	RT	11	9.5E-1	1.0E0
Annotation Cluster 69	Enrichment Score: 0.05		Count	P_Value	Benjamini
GOTERM_MF_FAT	exopeptidase activity	RT	6	8.5E-1	1.0E0
GOTERM_MF_FAT	metalloexopeptidase activity	RT	4	8.7E-1	1.0E0
GOTERM_MF_FAT	metallopeptidase activity	RT	5	9.8E-1	1.0E0
Annotation Cluster 70	Enrichment Score: 0.04		Count	P_Value	Benjamini
GOTERM_BP_FAT	organophosphate metabolic process	RT	7	8.5E-1	1.0E0
GOTERM_BP_FAT	phospholipid biosynthetic process	RT	4	8.6E-1	1.0E0
GOTERM_BP_FAT	lipid biosynthetic process	RT	7	9.0E-1	1.0E0
GOTERM_BP_FAT	phospholipid metabolic process	RT	6	9.1E-1	1.0E0
GOTERM_BP_FAT	glycerophospholipid metabolic process	RT	3	9.7E-1	1.0E0
GOTERM_BP_FAT	glycerolipid metabolic process	RT	3	9.7E-1	1.0E0
GOTERM_BP_FAT	phosphoinositide metabolic process	RT	3	9.7E-1	1.0E0
Annotation Cluster 71	Enrichment Score: 0.04		Count	P_Value	Benjamini
INTERPRO	EF hand	RT	6	8.7E-1	1.0E0
INTERPRO	EF-HAND 2	RT	9	8.7E-1	1.0E0
INTERPRO	EF-Hand type	RT	9	9.2E-1	1.0E0
INTERPRO	EF-HAND 1	RT	10	9.2E-1	1.0E0
SP_PIR_KEYWORDS	calcium	RT	7	9.5E-1	1.0E0
INTERPRO	Calcium-binding EF-hand	RT	5	9.6E-1	1.0E0
SMART	EFh	RT	5	9.7E-1	1.0E0
Annotation Cluster 72	Enrichment Score: 0.04		Count	P_Value	Benjamini
INTERPRO	Molecular chaperone, heat shock protein, Hsp40, DnaJ	RT	4	8.8E-1	1.0E0
GOTERM_MF_FAT	heat shock protein binding	RT	4	9.3E-1	1.0E0
INTERPRO	Heat shock protein DnaJ, N-terminal	RT	4	9.4E-1	1.0E0
SMART	DnaJ	RT	4	9.5E-1	1.0E0
Annotation Cluster 73	Enrichment Score: 0.03		Count	P_Value	Benjamini
INTERPRO	Nucleotide-binding, alpha-beta plait	RT	8	9.2E-1	1.0E0
INTERPRO	RNA recognition motif, RNP-1	RT	7	9.2E-1	1.0E0
SMART	RRM	RT	7	9.4E-1	1.0E0
Annotation Cluster 74	Enrichment Score: 0.03		Count	P_Value	Benjamini
INTERPRO	Acyl-CoA N-acyltransferase	RT	6	8.0E-1	1.0E0
SP_PIR_KEYWORDS	Acyltransferase	RT	6	8.7E-1	9.9E-1
GOTERM_MF_FAT	N-acyltransferase activity	RT	4	9.8E-1	1.0E0
GOTERM_MF_FAT	acetyltransferase activity	RT	4	9.8E-1	1.0E0
GOTERM_MF_FAT	N-acetyltransferase activity	RT	3	9.9E-1	1.0E0

Category	Term		Count	P_Value	Benjamini
INTERPRO	GCN5-related N-acetyltransferase	RT	3	9.9E-1	1.0E0
Annotation Cluster 75	Enrichment Score: 0.03	G	Count	P_Value	Benjamini
INTERPRO	Like-Sm ribonucleoprotein, core	RT	6	8.7E-1	1.0E0
INTERPRO	Like-Sm ribonucleoprotein, eukaryotic and archaea-type, core	RT	3	9.8E-1	1.0E0
SMART	Sm	RT	3	9.8E-1	1.0E0
Annotation Cluster 76	Enrichment Score: 0.01	G	Count	P_Value	Benjamini
GOTERM_MF_FAT	RNA-directed DNA polymerase activity	RT	11	9.5E-1	1.0E0
SP_PIR_KEYWORDS	Endonuclease	RT	14	9.6E-1	1.0E0
GOTERM_MF_FAT	nuclease activity	RT	23	9.7E-1	1.0E0
GOTERM_MF_FAT	endonuclease activity	RT	15	9.8E-1	1.0E0
GOTERM_MF_FAT	DNA polymerase activity	RT	15	9.8E-1	1.0E0
SP_PIR_KEYWORDS	rna-directed dna polymerase	RT	10	9.8E-1	1.0E0
Annotation Cluster 77	Enrichment Score: 0.01	G	Count	P_Value	Benjamini
GOTERM_BP_FAT	ribosome biogenesis	RT	9	9.6E-1	1.0E0
GOTERM_BP_FAT	ribonucleoprotein complex biogenesis	RT	9	9.6E-1	1.0E0
GOTERM_BP_FAT	rRNA processing	RT	5	9.8E-1	1.0E0
GOTERM_BP_FAT	rRNA metabolic process	RT	5	9.8E-1	1.0E0
GOTERM_BP_FAT	ncRNA processing	RT	11	1.0E0	1.0E0
Annotation Cluster 78	Enrichment Score: 0.01	G	Count	P_Value	Benjamini
INTERPRO	Ubiquitin	RT	3	9.8E-1	1.0E0
INTERPRO	Ubiquitin supergroup	RT	5	9.8E-1	1.0E0
SMART	UBQ	RT	3	9.8E-1	1.0E0
Annotation Cluster 79	Enrichment Score: 0.01	G	Count	P_Value	Benjamini
GOTERM_BP_FAT	ncRNA metabolic process	RT	25	9.5E-1	1.0E0
GOTERM_BP_FAT	tRNA processing	RT	6	9.9E-1	1.0E0
GOTERM_BP_FAT	ncRNA processing	RT	11	1.0E0	1.0E0
GOTERM_BP_FAT	RNA processing	RT	16	1.0E0	1.0E0

209 terms were not clustered.

Please cite *Nature Protocols 2009; 4(1):44 & Genome Biology 2003; 4(5):P3* within any publication that makes use of any methods inspired by **DAVID**.

Term of Service | Contact Us | Site Map

DAVID Bioinformatics Resources 6.7
National Institute of Allergy and Infectious Diseases (NIAID), NIH

Functional Annotation Clustering

Help and Manual

Current Gene List: 3_Random3
Current Background: Giardia intestinalis
3000 DAVID IDs

Options Classification Stringency Medium

Rerun using options | Create Sublist

75 Cluster(s) Download File

				Count	P_Value	Benjamini
Annotation Cluster 1		Enrichment Score: 0.85		Count	P_Value	Benjamini
INTERPRO	EGF-like, laminin	RT		20	8.3E-2	1.0E0
SMART	EGF_Lam	RT		20	9.0E-2	1.0E0
INTERPRO	EGF-like, type 3	RT		21	3.7E-1	1.0E0
Annotation Cluster 2		Enrichment Score: 0.85		Count	P_Value	Benjamini
GOTERM_MF_FAT	ATPase activity, coupled	RT		50	2.5E-2	1.0E0
SP_PIR_KEYWORDS	helicase	RT		32	6.0E-2	1.0E0
GOTERM_MF_FAT	helicase activity	RT		38	7.5E-2	1.0E0
GOTERM_MF_FAT	ATPase activity	RT		62	1.0E-1	1.0E0
INTERPRO	Helicase, superfamily 1 and 2, ATP-binding	RT		30	1.3E-1	1.0E0
INTERPRO	DNA/RNA helicase, C-terminal	RT		30	1.8E-1	1.0E0
SMART	HELICc	RT		30	1.9E-1	1.0E0
INTERPRO	RNA helicase, DEAD-box type, Q motif	RT		13	2.1E-1	1.0E0
INTERPRO	DEAD-like helicase, N-terminal	RT		29	2.1E-1	1.0E0
GOTERM_MF_FAT	purine NTP-dependent helicase activity	RT		26	2.1E-1	1.0E0
GOTERM_MF_FAT	ATP-dependent helicase activity	RT		26	2.1E-1	1.0E0
SMART	DEXDc	RT		29	2.3E-1	1.0E0
INTERPRO	DNA/RNA helicase, DEAD/DEAH box type, N-terminal	RT		20	3.4E-1	1.0E0
Annotation Cluster 3		Enrichment Score: 0.67		Count	P_Value	Benjamini
GOTERM_BP_FAT	monosaccharide metabolic process	RT		17	1.2E-1	1.0E0
GOTERM_BP_FAT	hexose metabolic process	RT		15	1.4E-1	1.0E0
GOTERM_BP_FAT	glucose metabolic process	RT		15	1.4E-1	1.0E0
GOTERM_BP_FAT	hexose catabolic process	RT		11	1.8E-1	1.0E0
GOTERM_BP_FAT	monosaccharide catabolic process	RT		11	1.8E-1	1.0E0
GOTERM_BP_FAT	glucose catabolic process	RT		11	1.8E-1	1.0E0
GOTERM_BP_FAT	cellular carbohydrate catabolic process	RT		11	1.8E-1	1.0E0
GOTERM_BP_FAT	alcohol catabolic process	RT		11	1.8E-1	1.0E0
GOTERM_BP_FAT	carbohydrate catabolic process	RT		11	1.8E-1	1.0E0
GOTERM_BP_FAT	generation of precursor metabolites and energy	RT		17	3.4E-1	1.0E0
GOTERM_BP_FAT	glycolysis	RT		8	4.1E-1	1.0E0
SP_PIR_KEYWORDS	glycolysis	RT		4	7.9E-1	1.0E0
Annotation Cluster 4		Enrichment Score: 0.66		Count	P_Value	Benjamini
INTERPRO	EF-HAND 1	RT		16	9.1E-2	1.0E0
INTERPRO	EF-HAND 2	RT		14	9.3E-2	1.0E0
INTERPRO	EF-Hand type	RT		14	1.6E-1	1.0E0
SP_PIR_KEYWORDS	calcium	RT		12	1.7E-1	1.0E0
GOTERM_MF_FAT	calcium ion binding	RT		29	2.2E-1	1.0E0
INTERPRO	Calcium-binding EF-hand	RT		8	4.8E-1	1.0E0
INTERPRO	EF hand	RT		8	4.8E-1	1.0E0
SMART	EFh	RT		8	4.9E-1	1.0E0
Annotation Cluster 5		Enrichment Score: 0.6		Count	P_Value	Benjamini
GOTERM_MF_FAT	DNA polymerase activity	RT		26	4.6E-2	1.0E0
GOTERM_MF_FAT	nucleotidyltransferase activity	RT		47	1.9E-1	1.0E0
GOTERM_MF_FAT	RNA-directed DNA polymerase activity	RT		17	2.6E-1	1.0E0
SP_PIR_KEYWORDS	rna-directed dna polymerase	RT		16	3.2E-1	1.0E0
GOTERM_MF_FAT	endonuclease activity	RT		22	4.0E-1	1.0E0

					Count	P_Value	Benjamini
	GOTERM_MF_FAT	nuclease activity	RT		31	4.6E-1	1.0E0
	SP_PIR_KEYWORDS	Endonuclease	RT		19	4.9E-1	9.9E-1
	Annotation Cluster 6	Enrichment Score: 0.57	G		Count	P_Value	Benjamini
	INTERPRO	WD40 repeat, subgroup	RT		38	1.3E-1	1.0E0
	INTERPRO	WD40 repeat 2	RT		29	2.1E-1	1.0E0
	INTERPRO	WD40 repeat, region	RT		31	2.5E-1	1.0E0
	INTERPRO	WD40/YVTN repeat-like	RT		46	2.5E-1	1.0E0
	SP_PIR_KEYWORDS	wd repeat	RT		32	2.7E-1	1.0E0
	INTERPRO	WD40 repeat	RT		46	3.5E-1	1.0E0
	SMART	WD40	RT		46	3.8E-1	1.0E0
	INTERPRO	WD40 repeat, conserved site	RT		19	4.0E-1	1.0E0
	Annotation Cluster 7	Enrichment Score: 0.5	G		Count	P_Value	Benjamini
	GOTERM_BP_FAT	nucleobase, nucleoside and nucleotide biosynthetic process	RT		30	5.0E-2	1.0E0
	GOTERM_BP_FAT	nucleobase, nucleoside, nucleotide and nucleic acid biosynthetic process	RT		30	5.0E-2	1.0E0
	GOTERM_BP_FAT	nucleotide biosynthetic process	RT		27	1.0E-1	1.0E0
	GOTERM_BP_FAT	nitrogen compound biosynthetic process	RT		33	1.0E-1	1.0E0
	GOTERM_BP_FAT	nucleoside triphosphate biosynthetic process	RT		19	1.6E-1	1.0E0
	GOTERM_BP_FAT	nucleoside triphosphate metabolic process	RT		19	1.6E-1	1.0E0
	GOTERM_BP_FAT	ribonucleoside triphosphate biosynthetic process	RT		19	1.6E-1	1.0E0
	GOTERM_BP_FAT	purine ribonucleoside triphosphate metabolic process	RT		19	1.6E-1	1.0E0
	GOTERM_BP_FAT	ribonucleoside triphosphate metabolic process	RT		19	1.6E-1	1.0E0
	GOTERM_BP_FAT	purine nucleoside triphosphate metabolic process	RT		19	1.6E-1	1.0E0
	GOTERM_BP_FAT	purine nucleoside triphosphate biosynthetic process	RT		19	1.6E-1	1.0E0
	GOTERM_BP_FAT	purine ribonucleoside triphosphate biosynthetic process	RT		19	1.6E-1	1.0E0
	GOTERM_BP_FAT	purine nucleotide biosynthetic process	RT		20	1.8E-1	1.0E0
	GOTERM_MF_FAT	ATPase activity, coupled to transmembrane movement of substances	RT		20	2.0E-1	1.0E0
	GOTERM_MF_FAT	ATPase activity, coupled to movement of substances	RT		20	2.0E-1	1.0E0
	GOTERM_MF_FAT	P-P-bond-hydrolysis-driven transmembrane transporter activity	RT		21	2.2E-1	1.0E0
	GOTERM_MF_FAT	primary active transmembrane transporter activity	RT		21	2.2E-1	1.0E0
	GOTERM_MF_FAT	hydrolase activity, acting on acid anhydrides, catalyzing transmembrane movement of substances	RT		21	2.2E-1	1.0E0
	GOTERM_BP_FAT	purine ribonucleotide metabolic process	RT		19	2.3E-1	1.0E0
	GOTERM_BP_FAT	purine ribonucleotide biosynthetic process	RT		19	2.3E-1	1.0E0
	GOTERM_BP_FAT	ribonucleotide biosynthetic process	RT		19	2.3E-1	1.0E0
	GOTERM_BP_FAT	ribonucleotide metabolic process	RT		19	2.3E-1	1.0E0
	GOTERM_BP_FAT	purine nucleotide metabolic process	RT		20	2.5E-1	1.0E0
	GOTERM_BP_FAT	ATP metabolic process	RT		17	2.6E-1	1.0E0
	GOTERM_BP_FAT	ATP biosynthetic process	RT		17	2.6E-1	1.0E0
	GOTERM_BP_FAT	generation of precursor metabolites and energy	RT		17	3.4E-1	1.0E0
	GOTERM_MF_FAT	ATPase activity, coupled to transmembrane movement of ions	RT		14	3.5E-1	1.0E0
	GOTERM_BP_FAT	transmembrane transport	RT		14	3.6E-1	1.0E0
	INTERPRO	ATPase, P-type, K/Mg/Cd/Cu/Zn/Na/Ca/Na/H-transporter	RT		9	3.8E-1	1.0E0
	GOTERM_MF_FAT	ATPase activity, coupled to transmembrane movement of ions, phosphorylative mechanism	RT		9	3.9E-1	1.0E0
	GOTERM_BP_FAT	hydrogen transport	RT		10	4.7E-1	1.0E0
	GOTERM_BP_FAT	proton transport	RT		10	4.7E-1	1.0E0
	INTERPRO	ATPase, P-type, ATPase-associated region	RT		8	4.8E-1	1.0E0
	GOTERM_MF_FAT	inorganic cation transmembrane transporter activity	RT		12	5.0E-1	1.0E0
	GOTERM_CC_FAT	proton-transporting two-sector ATPase complex	RT		10	5.5E-1	1.0E0
	GOTERM_MF_FAT	hydrogen ion transmembrane transporter activity	RT		10	5.5E-1	1.0E0
	GOTERM_MF_FAT	monovalent inorganic cation transmembrane transporter activity	RT		10	5.5E-1	1.0E0
	GOTERM_BP_FAT	ion transmembrane transport	RT		9	5.6E-1	1.0E0
	GOTERM_BP_FAT	monovalent inorganic cation transport	RT		10	5.8E-1	1.0E0

GOTERM_BP_FAT	oxidative phosphorylation	RT		8	6.6E-1	1.0E0
GOTERM_BP_FAT	ATP synthesis coupled proton transport	RT		8	6.6E-1	1.0E0
GOTERM_BP_FAT	energy coupled proton transport, down electrochemical gradient	RT		8	6.6E-1	1.0E0
GOTERM_MF_FAT	proton-transporting ATPase activity, rotational mechanism	RT		5	6.7E-1	1.0E0
GOTERM_CC_FAT	proton-transporting V-type ATPase complex	RT		6	7.0E-1	1.0E0
KEGG_PATHWAY	Oxidative phosphorylation	RT		8	7.2E-1	1.0E0
GOTERM_BP_FAT	ion transport	RT		13	7.8E-1	1.0E0
GOTERM_BP_FAT	cation transport	RT		13	7.8E-1	1.0E0
GOTERM_CC_FAT	proton-transporting two-sector ATPase complex, proton-transporting domain	RT		4	8.0E-1	1.0E0
GOTERM_CC_FAT	proton-transporting two-sector ATPase complex, catalytic domain	RT		5	8.0E-1	1.0E0
GOTERM_MF_FAT	cation-transporting ATPase activity	RT		6	8.0E-1	1.0E0
GOTERM_CC_FAT	proton-transporting V-type ATPase, V0 domain	RT		3	9.0E-1	1.0E0
Annotation Cluster 8	Enrichment Score: 0.46	G		Count	P_Value	Benjamini
INTERPRO	Pumilio RNA-binding region	RT		5	3.0E-1	1.0E0
SMART	Pumilio	RT		5	3.1E-1	1.0E0
INTERPRO	Armadillo-like helical	RT		14	4.5E-1	1.0E0
Annotation Cluster 9	Enrichment Score: 0.43	G		Count	P_Value	Benjamini
GOTERM_CC_FAT	intrinsic to membrane	RT		43	1.9E-1	1.0E0
GOTERM_CC_FAT	integral to membrane	RT		43	1.9E-1	1.0E0
SP_PIR_KEYWORDS	membrane	RT		18	3.8E-1	1.0E0
SP_PIR_KEYWORDS	transmembrane	RT		18	6.4E-1	1.0E0
INTERPRO	Amino acid transporter, transmembrane	RT		6	8.0E-1	1.0E0
Annotation Cluster 10	Enrichment Score: 0.39	G		Count	P_Value	Benjamini
GOTERM_MF_FAT	endopeptidase activity	RT		31	2.0E-1	1.0E0
GOTERM_MF_FAT	peptidase activity	RT		51	2.0E-1	1.0E0
GOTERM_BP_FAT	proteolysis	RT		54	2.1E-1	1.0E0
GOTERM_MF_FAT	peptidase activity, acting on L-amino acid peptides	RT		44	2.3E-1	1.0E0
SP_PIR_KEYWORDS	Protease	RT		24	2.9E-1	1.0E0
INTERPRO	Peptidase, cysteine peptidase active site	RT		19	4.9E-1	1.0E0
INTERPRO	Peptidase C1A, papain C-terminal	RT		16	6.8E-1	1.0E0
INTERPRO	Peptidase C1A, papain	RT		16	6.8E-1	1.0E0
SMART	Pept_C1	RT		16	7.0E-1	1.0E0
GOTERM_MF_FAT	cysteine-type endopeptidase activity	RT		14	7.3E-1	1.0E0
GOTERM_MF_FAT	cysteine-type peptidase activity	RT		17	7.7E-1	1.0E0
Annotation Cluster 11	Enrichment Score: 0.38	G		Count	P_Value	Benjamini
INTERPRO	Small GTP-binding protein	RT		15	1.9E-1	1.0E0
GOTERM_MF_FAT	guanyl nucleotide binding	RT		31	3.2E-1	1.0E0
GOTERM_MF_FAT	GTP binding	RT		31	3.2E-1	1.0E0
GOTERM_MF_FAT	guanyl ribonucleotide binding	RT		31	3.2E-1	1.0E0
GOTERM_BP_FAT	intracellular signaling cascade	RT		14	3.6E-1	1.0E0
GOTERM_BP_FAT	small GTPase mediated signal transduction	RT		11	3.8E-1	1.0E0
SP_PIR_KEYWORDS	gtp-binding	RT		25	4.7E-1	9.9E-1
INTERPRO	Ras	RT		8	6.1E-1	1.0E0
INTERPRO	Ras GTPase	RT		8	7.2E-1	1.0E0
PIR_SUPERFAMILY	PIRSF001710:Ras-related protein Rab	RT		4	7.7E-1	1.0E0
Annotation Cluster 12	Enrichment Score: 0.38	G		Count	P_Value	Benjamini
GOTERM_BP_FAT	pyridine nucleotide metabolic process	RT		6	1.7E-1	1.0E0
GOTERM_BP_FAT	oxidoreduction coenzyme metabolic process	RT		6	1.7E-1	1.0E0
GOTERM_BP_FAT	secondary metabolic process	RT		5	2.6E-1	1.0E0
GOTERM_BP_FAT	cellular amide metabolic process	RT		5	2.6E-1	1.0E0
GOTERM_BP_FAT	nicotinamide metabolic process	RT		5	2.6E-1	1.0E0
GOTERM_BP_FAT	alkaloid metabolic process	RT		5	2.6E-1	1.0E0
GOTERM_BP_FAT	nicotinamide nucleotide metabolic process	RT		5	2.6E-1	1.0E0
GOTERM_BP_FAT	coenzyme metabolic process	RT		8	2.8E-1	1.0E0
GOTERM_BP_FAT	pyridine nucleotide biosynthetic process	RT		4	4.0E-1	1.0E0
GOTERM_BP_FAT	cofactor metabolic process	RT		8	5.4E-1	1.0E0
GOTERM_BP_FAT	nicotinamide nucleotide biosynthetic process	RT		3	5.8E-1	1.0E0

GOTERM_BP_FAT	NAD biosynthetic process	RT	3	5.8E-1	1.0E0
GOTERM_BP_FAT	NAD metabolic process	RT	3	5.8E-1	1.0E0
GOTERM_BP_FAT	vitamin metabolic process	RT	4	6.0E-1	1.0E0
GOTERM_BP_FAT	water-soluble vitamin metabolic process	RT	4	6.0E-1	1.0E0
GOTERM_BP_FAT	water-soluble vitamin biosynthetic process	RT	4	6.0E-1	1.0E0
GOTERM_BP_FAT	vitamin biosynthetic process	RT	4	6.0E-1	1.0E0
GOTERM_BP_FAT	coenzyme biosynthetic process	RT	5	6.2E-1	1.0E0
GOTERM_BP_FAT	cofactor biosynthetic process	RT	5	8.4E-1	1.0E0
INTERPRO	Rossmann-like alpha/beta/alpha sandwich fold	RT	9	9.5E-1	1.0E0
Annotation Cluster 13	Enrichment Score: 0.35		Count	P_Value	Benjamini
SP_PIR_KEYWORDS	flagellum	RT	4	4.4E-1	1.0E0
GOTERM_CC_FAT	cell projection	RT	4	4.4E-1	1.0E0
GOTERM_CC_FAT	flagellum	RT	4	4.4E-1	1.0E0
Annotation Cluster 14	Enrichment Score: 0.32		Count	P_Value	Benjamini
INTERPRO	EGF, extracellular	RT	16	3.1E-1	1.0E0
INTERPRO	EGF-like, type 3	RT	21	3.7E-1	1.0E0
SP_PIR_KEYWORDS	egf-like domain	RT	8	9.5E-1	1.0E0
Annotation Cluster 15	Enrichment Score: 0.31		Count	P_Value	Benjamini
INTERPRO	Ankyrin	RT	254	4.6E-1	1.0E0
SP_PIR_KEYWORDS	ank repeat	RT	179	4.8E-1	9.9E-1
SMART	ANK	RT	254	5.6E-1	1.0E0
Annotation Cluster 16	Enrichment Score: 0.28		Count	P_Value	Benjamini
INTERPRO	EGF-like	RT	114	3.6E-1	1.0E0
SMART	EGF	RT	114	4.1E-1	1.0E0
INTERPRO	Giardia variant-specific surface protein	RT	136	6.2E-1	1.0E0
INTERPRO	Furin-like repeat	RT	106	6.3E-1	1.0E0
SMART	FU	RT	106	6.9E-1	1.0E0
Annotation Cluster 17	Enrichment Score: 0.28		Count	P_Value	Benjamini
GOTERM_MF_FAT	calcium ion binding	RT	29	2.2E-1	1.0E0
INTERPRO	Alpha giardin	RT	9	3.8E-1	1.0E0
INTERPRO	Annexin repeat	RT	10	4.2E-1	1.0E0
SMART	ANX	RT	10	4.4E-1	1.0E0
INTERPRO	Annexin	RT	11	4.6E-1	1.0E0
SP_PIR_KEYWORDS	annexin	RT	11	4.6E-1	1.0E0
GOTERM_BP_FAT	cytoskeleton organization	RT	9	7.6E-1	1.0E0
GOTERM_MF_FAT	calcium-dependent phospholipid binding	RT	12	8.4E-1	1.0E0
GOTERM_MF_FAT	lipid binding	RT	19	8.8E-1	1.0E0
GOTERM_MF_FAT	phospholipid binding	RT	13	9.5E-1	1.0E0
Annotation Cluster 18	Enrichment Score: 0.27		Count	P_Value	Benjamini
GOTERM_BP_FAT	microtubule-based movement	RT	30	2.4E-1	1.0E0
GOTERM_MF_FAT	motor activity	RT	27	3.7E-1	1.0E0
GOTERM_MF_FAT	microtubule motor activity	RT	27	3.7E-1	1.0E0
INTERPRO	Dynein heavy chain	RT	9	3.8E-1	1.0E0
GOTERM_CC_FAT	dynein complex	RT	9	3.8E-1	1.0E0
COG_ONTOLOGY	Cytoskeleton	RT	14	5.2E-1	9.9E-1
GOTERM_CC_FAT	microtubule associated complex	RT	10	5.5E-1	1.0E0
GOTERM_BP_FAT	microtubule-based process	RT	31	5.6E-1	1.0E0
SP_PIR_KEYWORDS	motor protein	RT	15	5.8E-1	1.0E0
GOTERM_CC_FAT	microtubule	RT	20	6.0E-1	1.0E0
INTERPRO	Kinesin, motor region	RT	17	6.1E-1	1.0E0
SMART	KISc	RT	17	6.3E-1	1.0E0
SP_PIR_KEYWORDS	microtubule	RT	19	6.5E-1	1.0E0
INTERPRO	Kinesin, motor region, conserved site	RT	11	6.7E-1	1.0E0
GOTERM_CC_FAT	cytoskeletal part	RT	30	7.0E-1	1.0E0
GOTERM_CC_FAT	microtubule cytoskeleton	RT	30	7.0E-1	1.0E0
INTERPRO	Dynein heavy chain, N-terminal region 2	RT	7	7.1E-1	1.0E0
GOTERM_CC_FAT	cytoskeleton	RT	30	8.1E-1	1.0E0
Annotation Cluster 19	Enrichment Score: 0.26		Count	P_Value	Benjamini

Category	Term		Count	P_Value	Benjamini
GOTERM_BP_FAT	intracellular transport	RT	18	3.6E-1	1.0E0
GOTERM_BP_FAT	cellular protein localization	RT	15	3.8E-1	1.0E0
GOTERM_BP_FAT	cellular macromolecule localization	RT	15	3.8E-1	1.0E0
GOTERM_BP_FAT	intracellular protein transport	RT	15	3.8E-1	1.0E0
GOTERM_BP_FAT	vesicle-mediated transport	RT	17	6.0E-1	1.0E0
GOTERM_CC_FAT	coated membrane	RT	9	6.4E-1	1.0E0
GOTERM_CC_FAT	membrane coat	RT	9	6.4E-1	1.0E0
GOTERM_BP_FAT	protein localization	RT	19	6.9E-1	1.0E0
GOTERM_CC_FAT	Golgi apparatus	RT	7	7.1E-1	1.0E0
GOTERM_BP_FAT	protein transport	RT	18	7.5E-1	1.0E0
GOTERM_BP_FAT	establishment of protein localization	RT	18	7.5E-1	1.0E0
Annotation Cluster 20	Enrichment Score: 0.26	G	Count	P_Value	Benjamini
INTERPRO	Aldolase-type TIM barrel	RT	7	4.3E-1	1.0E0
GOTERM_MF_FAT	FAD binding	RT	5	5.1E-1	1.0E0
PIR_SUPERFAMILY	PIRSF006621:Dus	RT	3	6.0E-1	1.0E0
INTERPRO	tRNA-dihydrouridine synthase	RT	3	6.2E-1	1.0E0
GOTERM_MF_FAT	tRNA dihydrouridine synthase activity	RT	3	6.2E-1	1.0E0
Annotation Cluster 21	Enrichment Score: 0.25	G	Count	P_Value	Benjamini
INTERPRO	Protein phosphatase 2C, manganese/magnesium aspartate binding site	RT	4	4.4E-1	1.0E0
SP_PIR_KEYWORDS	protein phosphatase	RT	4	4.4E-1	1.0E0
GOTERM_MF_FAT	protein serine/threonine phosphatase activity	RT	4	4.4E-1	1.0E0
INTERPRO	Protein phosphatase 2C, N-terminal	RT	5	5.0E-1	1.0E0
INTERPRO	Protein phosphatase 2C	RT	5	5.0E-1	1.0E0
INTERPRO	Protein phosphatase 2C-related	RT	5	5.0E-1	1.0E0
SMART	PP2C_SIG	RT	5	5.1E-1	1.0E0
SMART	PP2Cc	RT	5	5.1E-1	1.0E0
GOTERM_MF_FAT	magnesium ion binding	RT	10	5.5E-1	1.0E0
SP_PIR_KEYWORDS	magnesium	RT	8	6.1E-1	1.0E0
GOTERM_BP_FAT	dephosphorylation	RT	9	7.6E-1	1.0E0
GOTERM_BP_FAT	protein amino acid dephosphorylation	RT	7	8.3E-1	1.0E0
GOTERM_CC_FAT	protein serine/threonine phosphatase complex	RT	4	8.9E-1	1.0E0
Annotation Cluster 22	Enrichment Score: 0.25	G	Count	P_Value	Benjamini
GOTERM_MF_FAT	DNA-dependent ATPase activity	RT	7	2.8E-1	1.0E0
GOTERM_MF_FAT	DNA helicase activity	RT	4	8.0E-1	1.0E0
GOTERM_MF_FAT	ATP-dependent DNA helicase activity	RT	3	8.0E-1	1.0E0
Annotation Cluster 23	Enrichment Score: 0.25	G	Count	P_Value	Benjamini
GOTERM_BP_FAT	regulation of transcription, DNA-dependent	RT	8	5.4E-1	1.0E0
GOTERM_BP_FAT	regulation of RNA metabolic process	RT	8	5.4E-1	1.0E0
GOTERM_BP_FAT	regulation of transcription	RT	18	6.1E-1	1.0E0
Annotation Cluster 24	Enrichment Score: 0.25	G	Count	P_Value	Benjamini
GOTERM_BP_FAT	tRNA processing	RT	13	1.6E-1	1.0E0
GOTERM_BP_FAT	ncRNA processing	RT	17	6.0E-1	1.0E0
GOTERM_BP_FAT	tRNA metabolic process	RT	21	8.1E-1	1.0E0
GOTERM_BP_FAT	RNA processing	RT	24	8.2E-1	1.0E0
GOTERM_BP_FAT	ncRNA metabolic process	RT	25	9.4E-1	1.0E0
Annotation Cluster 25	Enrichment Score: 0.24	G	Count	P_Value	Benjamini
GOTERM_MF_FAT	DNA-directed DNA polymerase activity	RT	9	1.4E-1	1.0E0
INTERPRO	DNA-directed DNA polymerase, family B	RT	4	6.5E-1	1.0E0
SP_PIR_KEYWORDS	dna-directed dna polymerase	RT	4	6.5E-1	1.0E0
SMART	POLBc	RT	4	6.6E-1	1.0E0
INTERPRO	DNA-directed DNA polymerase, family B, conserved region2	RT	3	8.0E-1	1.0E0
INTERPRO	DNA-directed DNA polymerase, family B, conserved site	RT	3	8.0E-1	1.0E0
SP_PIR_KEYWORDS	dna replication	RT	6	8.0E-1	9.9E-1
Annotation Cluster 26	Enrichment Score: 0.24	G	Count	P_Value	Benjamini
INTERPRO	Nucleotide-binding, alpha-beta plait	RT	11	4.6E-1	1.0E0
INTERPRO	RNA recognition motif, RNP-1	RT	9	6.3E-1	1.0E0
SMART	RRM	RT	9	6.5E-1	1.0E0

			Count	P_Value	Benjamini
Annotation Cluster 27	Enrichment Score: 0.23		Count	P_Value	Benjamini
GOTERM_BP_FAT	phospholipid metabolic process	RT	9	3.2E-1	1.0E0
GOTERM_BP_FAT	phospholipid biosynthetic process	RT	6	3.3E-1	1.0E0
GOTERM_BP_FAT	lipid biosynthetic process	RT	10	3.5E-1	1.0E0
GOTERM_BP_FAT	organophosphate metabolic process	RT	9	4.4E-1	1.0E0
INTERPRO	CDP-alcohol phosphatidyltransferase	RT	3	6.2E-1	1.0E0
GOTERM_MF_FAT	phosphotransferase activity, for other substituted phosphate groups	RT	3	8.0E-1	1.0E0
GOTERM_BP_FAT	glycerolipid metabolic process	RT	4	8.6E-1	1.0E0
GOTERM_BP_FAT	phosphoinositide metabolic process	RT	4	8.6E-1	1.0E0
GOTERM_BP_FAT	glycerophospholipid metabolic process	RT	4	8.6E-1	1.0E0
GOTERM_MF_FAT	inositol or phosphatidylinositol kinase activity	RT	3	9.0E-1	1.0E0
Annotation Cluster 28	Enrichment Score: 0.22		Count	P_Value	Benjamini
SP_PIR_KEYWORDS	dna-binding	RT	24	2.3E-1	1.0E0
GOTERM_CC_FAT	chromosomal part	RT	12	3.8E-1	1.0E0
GOTERM_BP_FAT	chromatin assembly or disassembly	RT	9	4.4E-1	1.0E0
GOTERM_CC_FAT	protein-DNA complex	RT	9	5.2E-1	1.0E0
GOTERM_CC_FAT	chromosome	RT	17	5.4E-1	1.0E0
GOTERM_BP_FAT	cellular macromolecular complex subunit organization	RT	15	5.7E-1	1.0E0
GOTERM_BP_FAT	macromolecular complex subunit organization	RT	16	5.8E-1	1.0E0
GOTERM_CC_FAT	chromatin	RT	8	6.2E-1	1.0E0
INTERPRO	Histone-fold	RT	9	6.3E-1	1.0E0
GOTERM_BP_FAT	cellular macromolecular complex assembly	RT	14	6.4E-1	1.0E0
GOTERM_BP_FAT	protein-DNA complex assembly	RT	7	6.5E-1	1.0E0
GOTERM_BP_FAT	DNA packaging	RT	7	6.5E-1	1.0E0
GOTERM_BP_FAT	nucleosome assembly	RT	7	6.5E-1	1.0E0
GOTERM_BP_FAT	nucleosome organization	RT	7	6.5E-1	1.0E0
GOTERM_BP_FAT	chromatin assembly	RT	7	6.5E-1	1.0E0
GOTERM_BP_FAT	macromolecular complex assembly	RT	15	6.5E-1	1.0E0
SP_PIR_KEYWORDS	nucleosome core	RT	5	6.7E-1	1.0E0
GOTERM_BP_FAT	chromatin organization	RT	9	6.7E-1	1.0E0
INTERPRO	Histone core	RT	7	7.1E-1	1.0E0
SP_PIR_KEYWORDS	chromosomal protein	RT	7	7.1E-1	1.0E0
GOTERM_CC_FAT	nucleosome	RT	7	7.1E-1	1.0E0
SP_PIR_KEYWORDS	nucleus	RT	19	7.8E-1	1.0E0
GOTERM_BP_FAT	chromosome organization	RT	10	8.3E-1	1.0E0
Annotation Cluster 29	Enrichment Score: 0.22		Count	P_Value	Benjamini
INTERPRO	ATPase, AAA+ type, core	RT	42	5.6E-1	1.0E0
SMART	AAA	RT	42	6.0E-1	1.0E0
INTERPRO	ATPase, AAA-type, core	RT	15	6.6E-1	1.0E0
Annotation Cluster 30	Enrichment Score: 0.21		Count	P_Value	Benjamini
GOTERM_MF_FAT	intramolecular transferase activity	RT	8	4.8E-1	1.0E0
PIR_SUPERFAMILY	PIRSF001430:tRNA_psdUrid_synth	RT	3	6.0E-1	1.0E0
INTERPRO	Pseudouridine synthase I, TruA	RT	3	6.2E-1	1.0E0
GOTERM_BP_FAT	pseudouridine synthesis	RT	5	6.2E-1	1.0E0
GOTERM_MF_FAT	pseudouridine synthase activity	RT	5	6.7E-1	1.0E0
GOTERM_BP_FAT	RNA modification	RT	9	7.6E-1	1.0E0
Annotation Cluster 31	Enrichment Score: 0.21		Count	P_Value	Benjamini
INTERPRO	ARF/SAR superfamily	RT	6	3.8E-1	1.0E0
PIR_SUPERFAMILY	PIRSF001711:ADP-ribosylation factor	RT	4	6.3E-1	1.0E0
INTERPRO	ADP-ribosylation factor	RT	3	8.0E-1	1.0E0
SMART	ARF	RT	3	8.0E-1	1.0E0
Annotation Cluster 32	Enrichment Score: 0.2		Count	P_Value	Benjamini
GOTERM_BP_FAT	nucleoside metabolic process	RT	9	4.4E-1	1.0E0
GOTERM_BP_FAT	ribonucleoside metabolic process	RT	5	6.2E-1	1.0E0
GOTERM_BP_FAT	purine nucleoside metabolic process	RT	3	7.7E-1	1.0E0
GOTERM_BP_FAT	purine ribonucleoside metabolic process	RT	3	7.7E-1	1.0E0
Annotation Cluster 33	Enrichment Score: 0.2		Count	P_Value	Benjamini
GOTERM_BP_FAT	cellular response to stress	RT	13	5.3E-1	1.0E0

GOTERM_BP_FAT	mismatch repair	RT		4	6.0E-1	1.0E0
GOTERM_BP_FAT	DNA repair	RT		12	6.1E-1	1.0E0
GOTERM_BP_FAT	response to DNA damage stimulus	RT		12	6.1E-1	1.0E0
GOTERM_MF_FAT	double-stranded DNA binding	RT		4	6.5E-1	1.0E0
GOTERM_MF_FAT	structure-specific DNA binding	RT		4	6.5E-1	1.0E0
GOTERM_MF_FAT	mismatched DNA binding	RT		4	6.5E-1	1.0E0
KEGG_PATHWAY	Mismatch repair	RT		8	8.1E-1	9.9E-1
Annotation Cluster 34	Enrichment Score: 0.18	G		Count	P_Value	Benjamini
INTERPRO	ATPase, P-type, K/Mg/Cd/Cu/Zn/Na/Ca/Na/H-transporter	RT		9	3.8E-1	1.0E0
GOTERM_MF_FAT	ATPase activity, coupled to transmembrane movement of ions, phosphorylative mechanism	RT		9	3.9E-1	1.0E0
INTERPRO	ATPase, P-type, ATPase-associated region	RT		8	4.8E-1	1.0E0
GOTERM_BP_FAT	lipid transport	RT		4	6.0E-1	1.0E0
GOTERM_BP_FAT	lipid localization	RT		4	6.0E-1	1.0E0
GOTERM_BP_FAT	phospholipid transport	RT		3	7.7E-1	1.0E0
INTERPRO	ATPase, P-type phosphorylation site	RT		5	7.9E-1	1.0E0
GOTERM_MF_FAT	lipid transporter activity	RT		4	8.0E-1	1.0E0
INTERPRO	ATPase, P-type, phospholipid-translocating, flippase	RT		3	8.0E-1	1.0E0
GOTERM_MF_FAT	phospholipid-translocating ATPase activity	RT		3	9.0E-1	1.0E0
GOTERM_MF_FAT	phospholipid transporter activity	RT		3	9.0E-1	1.0E0
GOTERM_MF_FAT	aminophospholipid transporter activity	RT		3	9.0E-1	1.0E0
Annotation Cluster 35	Enrichment Score: 0.18	G		Count	P_Value	Benjamini
GOTERM_BP_FAT	proteolysis	RT		54	2.1E-1	1.0E0
GOTERM_BP_FAT	cellular macromolecule catabolic process	RT		20	5.6E-1	1.0E0
GOTERM_BP_FAT	ubiquitin-dependent protein catabolic process	RT		9	5.6E-1	1.0E0
GOTERM_BP_FAT	proteolysis involved in cellular protein catabolic process	RT		19	6.2E-1	1.0E0
GOTERM_BP_FAT	cellular protein catabolic process	RT		19	6.2E-1	1.0E0
INTERPRO	Proteasome, subunit alpha/beta	RT		9	6.3E-1	1.0E0
GOTERM_MF_FAT	threonine-type peptidase activity	RT		9	6.4E-1	1.0E0
GOTERM_MF_FAT	threonine-type endopeptidase activity	RT		9	6.4E-1	1.0E0
GOTERM_CC_FAT	proteasome core complex	RT		9	6.4E-1	1.0E0
GOTERM_BP_FAT	modification-dependent protein catabolic process	RT		15	6.5E-1	1.0E0
GOTERM_BP_FAT	modification-dependent macromolecule catabolic process	RT		15	6.5E-1	1.0E0
GOTERM_BP_FAT	macromolecule catabolic process	RT		22	6.5E-1	1.0E0
INTERPRO	Proteasome, alpha-subunit, conserved site	RT		5	6.7E-1	1.0E0
GOTERM_BP_FAT	protein catabolic process	RT		21	7.1E-1	1.0E0
INTERPRO	Proteasome, alpha and beta subunits	RT		8	7.2E-1	1.0E0
GOTERM_CC_FAT	proteasome complex	RT		12	7.8E-1	1.0E0
SP_PIR_KEYWORDS	threonine protease	RT		4	7.9E-1	1.0E0
GOTERM_CC_FAT	cytosol	RT		16	8.2E-1	1.0E0
SP_PIR_KEYWORDS	proteasome	RT		11	8.3E-1	9.9E-1
KEGG_PATHWAY	Proteasome	RT		15	8.6E-1	1.0E0
SP_PIR_KEYWORDS	ubl conjugation pathway	RT		7	8.8E-1	1.0E0
PIR_SUPERFAMILY	PIRSF001212:multicatalytic endopeptidase complex chain C9	RT		3	9.4E-1	1.0E0
Annotation Cluster 36	Enrichment Score: 0.17	G		Count	P_Value	Benjamini
INTERPRO	Tetratricopeptide-like helical	RT		20	4.3E-1	1.0E0
INTERPRO	Tetratricopeptide region	RT		15	5.7E-1	1.0E0
INTERPRO	Tetratricopeptide repeat	RT		14	7.2E-1	1.0E0
SMART	TPR	RT		14	7.4E-1	1.0E0
INTERPRO	Tetratricopeptide TPR-1	RT		11	8.3E-1	1.0E0
SP_PIR_KEYWORDS	tpr repeat	RT		5	8.8E-1	9.9E-1
Annotation Cluster 37	Enrichment Score: 0.16	G		Count	P_Value	Benjamini
GOTERM_MF_FAT	metallopeptidase activity	RT		10	3.0E-1	1.0E0
GOTERM_MF_FAT	metalloexopeptidase activity	RT		5	6.7E-1	1.0E0
GOTERM_MF_FAT	exopeptidase activity	RT		7	7.1E-1	1.0E0

GOTERM_MF_FAT	metallocarboxypeptidase activity	RT	3	8.0E-1	1.0E0
GOTERM_MF_FAT	carboxypeptidase activity	RT	3	8.0E-1	1.0E0
INTERPRO	Peptidase M14, carboxypeptidase A	RT	3	9.0E-1	1.0E0
SMART	Zn_pept	RT	3	9.0E-1	1.0E0
Annotation Cluster 38	Enrichment Score: 0.16		Count	P_Value	Benjamini
GOTERM_MF_FAT	translation factor activity, nucleic acid binding	RT	19	6.6E-1	1.0E0
GOTERM_MF_FAT	translation initiation factor activity	RT	15	6.7E-1	1.0E0
SP_PIR_KEYWORDS	Initiation factor	RT	11	7.6E-1	1.0E0
Annotation Cluster 39	Enrichment Score: 0.16		Count	P_Value	Benjamini
GOTERM_BP_FAT	alcohol biosynthetic process	RT	4	6.0E-1	1.0E0
GOTERM_BP_FAT	cellular carbohydrate biosynthetic process	RT	5	6.2E-1	1.0E0
GOTERM_BP_FAT	carbohydrate biosynthetic process	RT	5	6.2E-1	1.0E0
GOTERM_BP_FAT	monosaccharide biosynthetic process	RT	3	7.7E-1	1.0E0
GOTERM_BP_FAT	pyruvate metabolic process	RT	3	7.7E-1	1.0E0
GOTERM_BP_FAT	hexose biosynthetic process	RT	3	7.7E-1	1.0E0
GOTERM_BP_FAT	gluconeogenesis	RT	3	7.7E-1	1.0E0
Annotation Cluster 40	Enrichment Score: 0.14		Count	P_Value	Benjamini
GOTERM_BP_FAT	phosphorus metabolic process	RT	168	5.8E-1	1.0E0
GOTERM_BP_FAT	phosphate metabolic process	RT	168	5.8E-1	1.0E0
GOTERM_BP_FAT	phosphorylation	RT	159	5.8E-1	1.0E0
GOTERM_MF_FAT	nucleotide binding	RT	356	5.9E-1	1.0E0
GOTERM_BP_FAT	protein amino acid phosphorylation	RT	148	6.1E-1	1.0E0
GOTERM_MF_FAT	purine nucleotide binding	RT	334	6.4E-1	1.0E0
GOTERM_MF_FAT	purine ribonucleotide binding	RT	328	7.1E-1	1.0E0
GOTERM_MF_FAT	ribonucleotide binding	RT	328	7.1E-1	1.0E0
GOTERM_MF_FAT	nucleoside binding	RT	306	7.3E-1	1.0E0
GOTERM_MF_FAT	adenyl nucleotide binding	RT	303	7.6E-1	1.0E0
GOTERM_MF_FAT	purine nucleoside binding	RT	303	7.6E-1	1.0E0
GOTERM_MF_FAT	ATP binding	RT	297	8.2E-1	1.0E0
GOTERM_MF_FAT	adenyl ribonucleotide binding	RT	297	8.2E-1	1.0E0
SP_PIR_KEYWORDS	atp-binding	RT	143	8.4E-1	9.9E-1
SP_PIR_KEYWORDS	nucleotide-binding	RT	150	8.5E-1	9.9E-1
INTERPRO	Protein kinase, core	RT	147	8.5E-1	1.0E0
GOTERM_MF_FAT	protein kinase activity	RT	150	8.6E-1	1.0E0
SP_PIR_KEYWORDS	kinase	RT	183	8.7E-1	1.0E0
INTERPRO	Serine/threonine protein kinase-related	RT	126	9.2E-1	1.0E0
Annotation Cluster 41	Enrichment Score: 0.13		Count	P_Value	Benjamini
GOTERM_BP_FAT	carboxylic acid biosynthetic process	RT	5	6.2E-1	1.0E0
GOTERM_BP_FAT	organic acid biosynthetic process	RT	5	6.2E-1	1.0E0
GOTERM_BP_FAT	cellular amino acid biosynthetic process	RT	3	8.8E-1	1.0E0
GOTERM_BP_FAT	amine biosynthetic process	RT	3	9.4E-1	1.0E0
Annotation Cluster 42	Enrichment Score: 0.11		Count	P_Value	Benjamini
GOTERM_CC_FAT	organelle membrane	RT	8	4.8E-1	1.0E0
GOTERM_CC_FAT	membrane coat	RT	9	6.4E-1	1.0E0
GOTERM_CC_FAT	coated membrane	RT	9	6.4E-1	1.0E0
INTERPRO	Clathrin/coatomer adaptor, adaptin-like, N-terminal	RT	4	6.5E-1	1.0E0
GOTERM_CC_FAT	Golgi apparatus	RT	7	7.1E-1	1.0E0
GOTERM_CC_FAT	cytoplasmic vesicle	RT	4	8.0E-1	1.0E0
GOTERM_CC_FAT	coated vesicle membrane	RT	4	8.0E-1	1.0E0
GOTERM_CC_FAT	cytoplasmic vesicle membrane	RT	4	8.0E-1	1.0E0
GOTERM_CC_FAT	coated vesicle	RT	4	8.0E-1	1.0E0
GOTERM_CC_FAT	vesicle coat	RT	4	8.0E-1	1.0E0
GOTERM_CC_FAT	cytoplasmic membrane-bounded vesicle	RT	4	8.0E-1	1.0E0
GOTERM_CC_FAT	cytoplasmic vesicle part	RT	4	8.0E-1	1.0E0
GOTERM_CC_FAT	vesicle membrane	RT	4	8.0E-1	1.0E0
GOTERM_CC_FAT	vesicle	RT	4	8.0E-1	1.0E0
GOTERM_CC_FAT	membrane-bounded vesicle	RT	4	8.0E-1	1.0E0
	Golgi apparatus part	RT	5	8.0E-1	1.0E0

Category	Term		Count	P_Value	Benjamini
GOTERM_CC_FAT					
GOTERM_CC_FAT	transport vesicle membrane	RT	3	9.0E-1	1.0E0
GOTERM_CC_FAT	transport vesicle	RT	3	9.0E-1	1.0E0
GOTERM_CC_FAT	endomembrane system	RT	8	9.3E-1	1.0E0
GOTERM_BP_FAT	ER to Golgi vesicle-mediated transport	RT	3	9.4E-1	1.0E0
GOTERM_BP_FAT	Golgi vesicle transport	RT	3	9.4E-1	1.0E0
Annotation Cluster 43	Enrichment Score: 0.11		Count	P_Value	Benjamini
INTERPRO	Zinc finger, C3HC4 RING-type	RT	22	4.7E-1	1.0E0
INTERPRO	Zinc finger, RING-type	RT	39	6.3E-1	1.0E0
SMART	RING	RT	39	6.7E-1	1.0E0
SP_PIR_KEYWORDS	zinc-finger	RT	34	7.8E-1	1.0E0
SP_PIR_KEYWORDS	metal-binding	RT	55	8.3E-1	1.0E0
SP_PIR_KEYWORDS	zinc	RT	46	8.3E-1	9.9E-1
GOTERM_MF_FAT	metal ion binding	RT	142	8.5E-1	1.0E0
GOTERM_MF_FAT	cation binding	RT	143	8.7E-1	1.0E0
GOTERM_MF_FAT	ion binding	RT	143	8.7E-1	1.0E0
GOTERM_MF_FAT	zinc ion binding	RT	80	9.5E-1	1.0E0
GOTERM_MF_FAT	transition metal ion binding	RT	97	9.8E-1	1.0E0
Annotation Cluster 44	Enrichment Score: 0.11		Count	P_Value	Benjamini
INTERPRO	Like-Sm ribonucleoprotein, eukaryotic and archaea-type, core	RT	5	6.7E-1	1.0E0
SMART	Sm	RT	5	6.8E-1	1.0E0
INTERPRO	Like-Sm ribonucleoprotein, core	RT	6	8.7E-1	1.0E0
SP_PIR_KEYWORDS	viral nucleoprotein	RT	7	9.5E-1	1.0E0
Annotation Cluster 45	Enrichment Score: 0.1		Count	P_Value	Benjamini
GOTERM_MF_FAT	phosphatase activity	RT	25	7.4E-1	1.0E0
GOTERM_BP_FAT	dephosphorylation	RT	9	7.6E-1	1.0E0
GOTERM_MF_FAT	phosphoprotein phosphatase activity	RT	15	8.6E-1	1.0E0
Annotation Cluster 46	Enrichment Score: 0.1		Count	P_Value	Benjamini
KEGG_PATHWAY	Ubiquitin mediated proteolysis	RT	9	2.4E-1	1.0E0
INTERPRO	Ubiquitin-conjugating enzyme/RWD-like	RT	7	8.0E-1	1.0E0
INTERPRO	Ubiquitin-conjugating enzyme, E2	RT	7	8.0E-1	1.0E0
SMART	UBCc	RT	7	8.1E-1	1.0E0
SP_PIR_KEYWORDS	ubl conjugation pathway	RT	7	8.8E-1	1.0E0
GOTERM_MF_FAT	small conjugating protein ligase activity	RT	7	8.8E-1	1.0E0
GOTERM_MF_FAT	ubiquitin-protein ligase activity	RT	3	9.0E-1	1.0E0
PIR_SUPERFAMILY	PIRSF001567:ubiquitin-protein ligase E2	RT	3	9.4E-1	1.0E0
GOTERM_MF_FAT	ligase activity, forming carbon-nitrogen bonds	RT	15	9.7E-1	1.0E0
GOTERM_MF_FAT	acid-amino acid ligase activity	RT	12	9.8E-1	1.0E0
SP_PIR_KEYWORDS	ligase	RT	26	9.8E-1	1.0E0
Annotation Cluster 47	Enrichment Score: 0.1		Count	P_Value	Benjamini
GOTERM_BP_FAT	M phase	RT	4	7.6E-1	1.0E0
GOTERM_BP_FAT	cell cycle process	RT	4	7.6E-1	1.0E0
GOTERM_BP_FAT	cell cycle phase	RT	4	7.6E-1	1.0E0
GOTERM_BP_FAT	cell cycle	RT	4	9.2E-1	1.0E0
Annotation Cluster 48	Enrichment Score: 0.1		Count	P_Value	Benjamini
COG_ONTOLOGY	Defense mechanisms	RT	14	6.2E-1	9.9E-1
INTERPRO	ABC transporter-like	RT	12	8.8E-1	1.0E0
INTERPRO	ABC transporter, conserved site	RT	11	9.2E-1	1.0E0
Annotation Cluster 49	Enrichment Score: 0.09		Count	P_Value	Benjamini
INTERPRO	Myb transcription factor	RT	3	8.0E-1	1.0E0
INTERPRO	Myb-type HTH DNA-binding domain	RT	3	8.0E-1	1.0E0
INTERPRO	SANT, DNA-binding	RT	6	8.0E-1	1.0E0
SMART	SANT	RT	6	8.1E-1	1.0E0
INTERPRO	Myb, DNA-binding	RT	3	9.0E-1	1.0E0
Annotation Cluster 50	Enrichment Score: 0.09		Count	P_Value	Benjamini
INTERPRO	AMP-dependent synthetase and ligase	RT	5	6.7E-1	1.0E0
COG_ONTOLOGY	Lipid metabolism	RT	8	7.7E-1	9.9E-1
GOTERM_MF_FAT	long-chain-fatty-acid-CoA ligase activity	RT	3	9.0E-1	1.0E0

GOTERM_MF_FAT	fatty-acid ligase activity	RT	3	9.0E-1	1.0E0
GOTERM_MF_FAT	ligase activity, forming carbon-sulfur bonds	RT	3	9.0E-1	1.0E0
Annotation Cluster 51	Enrichment Score: 0.08		Count	P_Value	Benjamini
KEGG_PATHWAY	DNA replication	RT	12	7.7E-1	1.0E0
KEGG_PATHWAY	Mismatch repair	RT	8	8.1E-1	9.9E-1
KEGG_PATHWAY	Nucleotide excision repair	RT	8	9.2E-1	1.0E0
Annotation Cluster 52	Enrichment Score: 0.08		Count	P_Value	Benjamini
GOTERM_BP_FAT	ubiquitin-dependent protein catabolic process	RT	9	5.6E-1	1.0E0
INTERPRO	Peptidase C19, ubiquitin carboxyl-terminal hydrolase 2, conserved site	RT	3	8.0E-1	1.0E0
INTERPRO	Peptidase C19, ubiquitin carboxyl-terminal hydrolase 2	RT	3	9.5E-1	1.0E0
GOTERM_MF_FAT	ubiquitin thiolesterase activity	RT	3	9.5E-1	1.0E0
GOTERM_MF_FAT	thiolester hydrolase activity	RT	3	9.9E-1	1.0E0
Annotation Cluster 53	Enrichment Score: 0.07		Count	P_Value	Benjamini
SP_PIR_KEYWORDS	nucleotidyltransferase	RT	15	6.6E-1	1.0E0
KEGG_PATHWAY	Pyrimidine metabolism	RT	19	7.2E-1	1.0E0
KEGG_PATHWAY	Purine metabolism	RT	18	7.7E-1	1.0E0
SP_PIR_KEYWORDS	Transcription	RT	5	8.0E-1	1.0E0
GOTERM_MF_FAT	ribonucleoside binding	RT	3	8.0E-1	1.0E0
SP_PIR_KEYWORDS	dna-directed rna polymerase	RT	7	9.2E-1	1.0E0
GOTERM_BP_FAT	transcription	RT	12	9.4E-1	1.0E0
KEGG_PATHWAY	RNA polymerase	RT	8	9.7E-1	1.0E0
GOTERM_MF_FAT	DNA-directed RNA polymerase activity	RT	10	9.8E-1	1.0E0
GOTERM_MF_FAT	RNA polymerase activity	RT	10	9.9E-1	1.0E0
Annotation Cluster 54	Enrichment Score: 0.07		Count	P_Value	Benjamini
INTERPRO	Ubiquitin supergroup	RT	7	8.0E-1	1.0E0
INTERPRO	Ubiquitin	RT	4	8.8E-1	1.0E0
SMART	UBQ	RT	4	8.9E-1	1.0E0
Annotation Cluster 55	Enrichment Score: 0.06		Count	P_Value	Benjamini
INTERPRO	N-6 adenine-specific DNA methylase, conserved site	RT	4	7.9E-1	1.0E0
GOTERM_BP_FAT	one-carbon metabolic process	RT	6	8.4E-1	1.0E0
GOTERM_BP_FAT	methylation	RT	5	9.1E-1	1.0E0
GOTERM_BP_FAT	biopolymer methylation	RT	3	9.4E-1	1.0E0
Annotation Cluster 56	Enrichment Score: 0.06		Count	P_Value	Benjamini
GOTERM_MF_FAT	protein serine/threonine kinase activity	RT	59	7.3E-1	1.0E0
SP_PIR_KEYWORDS	serine/threonine-protein kinase	RT	34	7.8E-1	1.0E0
INTERPRO	Protein kinase, ATP binding site	RT	43	9.1E-1	1.0E0
INTERPRO	Serine/threonine protein kinase-related	RT	126	9.2E-1	1.0E0
INTERPRO	Serine/threonine protein kinase, active site	RT	46	9.4E-1	1.0E0
INTERPRO	Serine/threonine protein kinase	RT	29	9.5E-1	1.0E0
SMART	S_TKc	RT	29	9.6E-1	1.0E0
Annotation Cluster 57	Enrichment Score: 0.05		Count	P_Value	Benjamini
GOTERM_MF_FAT	antioxidant activity	RT	5	6.7E-1	1.0E0
INTERPRO	Alkyl hydroperoxide reductase/ Thiol specific antioxidant/ Mal allergen	RT	4	7.9E-1	1.0E0
GOTERM_BP_FAT	cell redox homeostasis	RT	9	8.8E-1	1.0E0
INTERPRO	Thioredoxin fold	RT	11	9.2E-1	1.0E0
GOTERM_BP_FAT	cellular homeostasis	RT	9	9.2E-1	1.0E0
INTERPRO	Thioredoxin, conserved site	RT	3	9.5E-1	1.0E0
GOTERM_BP_FAT	homeostatic process	RT	9	9.5E-1	1.0E0
INTERPRO	Thioredoxin-like	RT	6	9.6E-1	1.0E0
INTERPRO	Thioredoxin, core	RT	3	9.8E-1	1.0E0
Annotation Cluster 58	Enrichment Score: 0.05		Count	P_Value	Benjamini
KEGG_PATHWAY	Ribosome	RT	37	8.5E-1	1.0E0
GOTERM_CC_FAT	ribosome	RT	41	8.6E-1	1.0E0
GOTERM_MF_FAT	structural constituent of ribosome	RT	37	8.8E-1	1.0E0
GOTERM_BP_FAT	translation	RT	58	8.8E-1	1.0E0
GOTERM_CC_FAT	intracellular non-membrane-bounded organelle	RT	88	8.8E-1	1.0E0
GOTERM_CC_FAT	non-membrane-bounded organelle	RT	88	8.8E-1	1.0E0

SP_PIR_KEYWORDS	ribosomal protein	RT	38	9.0E-1	1.0E0
GOTERM_MF_FAT	structural molecule activity	RT	44	9.1E-1	1.0E0
GOTERM_CC_FAT	ribonucleoprotein complex	RT	50	9.9E-1	1.0E0
Annotation Cluster 59	Enrichment Score: 0.05		Count	P_Value	Benjamini
GOTERM_MF_FAT	acetyltransferase activity	RT	6	8.0E-1	1.0E0
INTERPRO	GCN5-related N-acetyltransferase	RT	5	8.8E-1	1.0E0
GOTERM_MF_FAT	N-acetyltransferase activity	RT	5	8.8E-1	1.0E0
GOTERM_MF_FAT	N-acyltransferase activity	RT	5	9.3E-1	1.0E0
INTERPRO	Acyl-CoA N-acyltransferase	RT	4	9.8E-1	1.0E0
Annotation Cluster 60	Enrichment Score: 0.05		Count	P_Value	Benjamini
GOTERM_BP_FAT	drug transport	RT	3	8.8E-1	1.0E0
GOTERM_BP_FAT	multidrug transport	RT	3	8.8E-1	1.0E0
GOTERM_BP_FAT	response to drug	RT	3	8.8E-1	1.0E0
INTERPRO	Multi antimicrobial extrusion protein MatE	RT	3	9.0E-1	1.0E0
GOTERM_MF_FAT	drug transporter activity	RT	3	9.0E-1	1.0E0
GOTERM_MF_FAT	antiporter activity	RT	3	9.5E-1	1.0E0
Annotation Cluster 61	Enrichment Score: 0.04		Count	P_Value	Benjamini
PIR_SUPERFAMILY	PIRSF000908:serine/threonine-protein phosphatase, conventional type	RT	5	7.7E-1	1.0E0
GOTERM_MF_FAT	phosphoprotein phosphatase activity	RT	15	8.6E-1	1.0E0
INTERPRO	Metallophosphoesterase	RT	11	8.8E-1	1.0E0
SP_PIR_KEYWORDS	iron	RT	10	9.2E-1	1.0E0
INTERPRO	Serine/threonine-specific protein phosphatase and bis(5-nucleosyl)-tetraphosphatase	RT	7	9.5E-1	1.0E0
SMART	PP2Ac	RT	7	9.6E-1	1.0E0
GOTERM_MF_FAT	iron ion binding	RT	13	9.9E-1	1.0E0
Annotation Cluster 62	Enrichment Score: 0.04		Count	P_Value	Benjamini
GOTERM_MF_FAT	GTPase activity	RT	9	8.8E-1	1.0E0
INTERPRO	Translation elongation factor EFTu/EF1A, domain 2	RT	3	9.0E-1	1.0E0
INTERPRO	Protein synthesis factor, GTP-binding	RT	4	9.4E-1	1.0E0
Annotation Cluster 63	Enrichment Score: 0.04		Count	P_Value	Benjamini
GOTERM_BP_FAT	protein complex biogenesis	RT	7	8.3E-1	1.0E0
GOTERM_BP_FAT	protein complex assembly	RT	7	8.3E-1	1.0E0
GOTERM_BP_FAT	cellular protein complex assembly	RT	6	8.4E-1	1.0E0
GOTERM_BP_FAT	protein polymerization	RT	4	9.2E-1	1.0E0
INTERPRO	Tubulin	RT	4	9.4E-1	1.0E0
INTERPRO	Tubulin, conserved site	RT	4	9.4E-1	1.0E0
INTERPRO	Tubulin/FtsZ, GTPase domain	RT	4	9.4E-1	1.0E0
PIR_SUPERFAMILY	PIRSF002306:tubulin	RT	3	9.7E-1	1.0E0
INTERPRO	Tubulin/FtsZ, 2-layer sandwich domain	RT	3	9.8E-1	1.0E0
Annotation Cluster 64	Enrichment Score: 0.04		Count	P_Value	Benjamini
GOTERM_MF_FAT	electron carrier activity	RT	13	7.9E-1	1.0E0
GOTERM_MF_FAT	metal cluster binding	RT	10	9.2E-1	1.0E0
GOTERM_MF_FAT	iron-sulfur cluster binding	RT	10	9.2E-1	1.0E0
INTERPRO	4Fe-4S ferredoxin, iron-sulphur binding, subgroup	RT	4	9.7E-1	1.0E0
INTERPRO	4Fe-4S ferredoxin, iron-sulpur binding domain	RT	4	9.7E-1	1.0E0
Annotation Cluster 65	Enrichment Score: 0.04		Count	P_Value	Benjamini
INTERPRO	Tyrosine protein kinase	RT	4	8.8E-1	1.0E0
SMART	TyrKc	RT	4	8.9E-1	1.0E0
GOTERM_MF_FAT	protein tyrosine kinase activity	RT	11	9.9E-1	1.0E0
Annotation Cluster 66	Enrichment Score: 0.04		Count	P_Value	Benjamini
GOTERM_MF_FAT	vitamin binding	RT	6	8.8E-1	1.0E0
GOTERM_MF_FAT	vitamin B6 binding	RT	5	9.3E-1	1.0E0
GOTERM_MF_FAT	pyridoxal phosphate binding	RT	5	9.3E-1	1.0E0
INTERPRO	Pyridoxal phosphate-dependent transferase, major region, subdomain 1	RT	4	9.4E-1	1.0E0
Annotation Cluster 67	Enrichment Score: 0.03		Count	P_Value	Benjamini
INTERPRO	Zinc finger, C2H2-like	RT	9	9.2E-1	1.0E0
SMART	ZnF_C2H2	RT	9	9.3E-1	1.0E0
INTERPRO	Zinc finger, C2H2-type	RT	8	9.7E-1	1.0E0

Annotation Cluster 68	Enrichment Score: 0.02			Count	P_Value	Benjamini
GOTERM_MF_FAT	nucleoside-triphosphatase regulator activity	RT		7	8.8E-1	1.0E0
GOTERM_MF_FAT	enzyme activator activity	RT		5	8.8E-1	1.0E0
GOTERM_BP_FAT	regulation of Ras protein signal transduction	RT		4	9.5E-1	1.0E0
GOTERM_BP_FAT	regulation of small GTPase mediated signal transduction	RT		4	9.5E-1	1.0E0
GOTERM_MF_FAT	GTPase regulator activity	RT		5	9.6E-1	1.0E0
GOTERM_MF_FAT	small GTPase regulator activity	RT		5	9.6E-1	1.0E0
GOTERM_BP_FAT	regulation of Ras GTPase activity	RT		3	9.7E-1	1.0E0
GOTERM_MF_FAT	GTPase activator activity	RT		3	9.8E-1	1.0E0
GOTERM_BP_FAT	regulation of GTPase activity	RT		3	9.8E-1	1.0E0
GOTERM_BP_FAT	regulation of hydrolase activity	RT		3	9.8E-1	1.0E0
Annotation Cluster 69	Enrichment Score: 0.02			Count	P_Value	Benjamini
INTERPRO	Anticodon-binding	RT		3	8.0E-1	1.0E0
GOTERM_BP_FAT	tRNA metabolic process	RT		21	8.1E-1	1.0E0
INTERPRO	Aminoacyl-tRNA synthetase, class II (G, H, P and S), conserved region	RT		3	9.0E-1	1.0E0
INTERPRO	Aminoacyl-tRNA synthetase, class II, conserved region	RT		4	9.7E-1	1.0E0
SP_PIR_KEYWORDS	Aminoacyl-tRNA synthetase	RT		9	9.9E-1	1.0E0
GOTERM_BP_FAT	tRNA aminoacylation for protein translation	RT		8	9.9E-1	1.0E0
KEGG_PATHWAY	Aminoacyl-tRNA biosynthesis	RT		8	9.9E-1	1.0E0
GOTERM_BP_FAT	tRNA aminoacylation	RT		8	1.0E0	1.0E0
GOTERM_BP_FAT	amino acid activation	RT		8	1.0E0	1.0E0
GOTERM_MF_FAT	ligase activity, forming carbon-oxygen bonds	RT		9	1.0E0	1.0E0
GOTERM_MF_FAT	aminoacyl-tRNA ligase activity	RT		9	1.0E0	1.0E0
GOTERM_MF_FAT	ligase activity, forming aminoacyl-tRNA and related compounds	RT		9	1.0E0	1.0E0
Annotation Cluster 70	Enrichment Score: 0.02			Count	P_Value	Benjamini
INTERPRO	DNA-dependent ATPase MCM	RT		3	9.5E-1	1.0E0
SMART	MCM	RT		3	9.5E-1	1.0E0
INTERPRO	Nucleic acid-binding, OB-fold	RT		9	9.7E-1	1.0E0
Annotation Cluster 71	Enrichment Score: 0.02			Count	P_Value	Benjamini
INTERPRO	Kelch repeat type 1	RT		3	9.5E-1	1.0E0
SMART	Kelch	RT		3	9.5E-1	1.0E0
INTERPRO	Kelch-type beta propeller	RT		3	9.8E-1	1.0E0
Annotation Cluster 72	Enrichment Score: 0.01			Count	P_Value	Benjamini
INTERPRO	Peptidyl-prolyl cis-trans isomerase, FKBP-type	RT		3	9.5E-1	1.0E0
SP_PIR_KEYWORDS	Rotamase	RT		3	9.9E-1	1.0E0
GOTERM_MF_FAT	cis-trans isomerase activity	RT		3	9.9E-1	1.0E0
GOTERM_MF_FAT	peptidyl-prolyl cis-trans isomerase activity	RT		3	9.9E-1	1.0E0
Annotation Cluster 73	Enrichment Score: 0.01			Count	P_Value	Benjamini
INTERPRO	Chaperonin TCP-1, conserved site	RT		4	9.7E-1	1.0E0
INTERPRO	Chaperonin Cpn60/TCP-1	RT		4	9.7E-1	1.0E0
PIR_SUPERFAMILY	PIRSF002584:molecular chaperone t-complex-type	RT		3	9.9E-1	1.0E0
INTERPRO	Chaperone, tailless complex polypeptide 1	RT		3	9.9E-1	1.0E0
SP_PIR_KEYWORDS	cytoplasm	RT		7	9.9E-1	1.0E0
SP_PIR_KEYWORDS	Chaperone	RT		4	1.0E0	1.0E0
GOTERM_MF_FAT	unfolded protein binding	RT		7	1.0E0	1.0E0
Annotation Cluster 74	Enrichment Score: 0.01			Count	P_Value	Benjamini
INTERPRO	Molecular chaperone, heat shock protein, Hsp40, DnaJ	RT		3	9.8E-1	1.0E0
INTERPRO	Heat shock protein DnaJ, N-terminal	RT		3	9.9E-1	1.0E0
GOTERM_MF_FAT	heat shock protein binding	RT		3	9.9E-1	1.0E0
SMART	DnaJ	RT		3	9.9E-1	1.0E0
Annotation Cluster 75	Enrichment Score: 0			Count	P_Value	Benjamini
GOTERM_BP_FAT	ribonucleoprotein complex biogenesis	RT		8	9.8E-1	1.0E0
GOTERM_BP_FAT	ribosome biogenesis	RT		8	9.8E-1	1.0E0
GOTERM_BP_FAT	rRNA metabolic process	RT		4	1.0E0	1.0E0
GOTERM_BP_FAT	rRNA processing	RT		4	1.0E0	1.0E0

212 terms were not clustered.

Please cite *Nature Protocols 2009; 4(1):44 & Genome Biology 2003; 4(5):P3* within any publication that makes use of any methods inspired by **DAVID**.

SAIC Frederick A Division of Science Applications International Corporation NIAID ABCC NCI-Frederick FIRSTGOV Your First Click to the U.S. Government Department of Health and Human Services

Term of Service | Contact Us | Site Map

DAVID Bioinformatics Resources 6.7
National Institute of Allergy and Infectious Diseases (NIAID), NIH

Functional Annotation Clustering

Help and Manual

Current Gene List: 3_Random4
Current Background: Giardia intestinalis
3000 DAVID IDs

Options Classification Stringency Medium

Rerun using options | Create Sublist

79 Cluster(s)

Download File

Annotation Cluster 1	Enrichment Score: 0.77	G	Count	P_Value	Benjamini
GOTERM_MF_FAT	calcium ion binding	RT	32	2.7E-2	1.0E0
GOTERM_MF_FAT	calcium-dependent phospholipid binding	RT	18	3.8E-2	1.0E0
GOTERM_BP_FAT	cytoskeleton organization	RT	14	3.9E-2	1.0E0
INTERPRO	Alpha giardin	RT	10	1.9E-1	1.0E0
SP_PIR_KEYWORDS	annexin	RT	12	2.4E-1	1.0E0
INTERPRO	Annexin	RT	12	2.8E-1	1.0E0
GOTERM_MF_FAT	phospholipid binding	RT	18	3.3E-1	1.0E0
INTERPRO	Annexin repeat	RT	10	4.3E-1	1.0E0
SMART	ANX	RT	10	4.3E-1	1.0E0
GOTERM_MF_FAT	lipid binding	RT	21	6.3E-1	1.0E0
Annotation Cluster 2	Enrichment Score: 0.74	G	Count	P_Value	Benjamini
GOTERM_MF_FAT	protein kinase regulator activity	RT	7	1.2E-1	1.0E0
GOTERM_MF_FAT	kinase regulator activity	RT	7	1.2E-1	1.0E0
GOTERM_MF_FAT	cAMP-dependent protein kinase regulator activity	RT	4	4.2E-1	1.0E0
Annotation Cluster 3	Enrichment Score: 0.57	G	Count	P_Value	Benjamini
SP_PIR_KEYWORDS	cyclin	RT	9	1.2E-1	1.0E0
INTERPRO	Cyclin, N-terminal	RT	6	2.1E-1	1.0E0
INTERPRO	Cyclin-related	RT	6	2.1E-1	1.0E0
INTERPRO	Cyclin	RT	5	3.1E-1	1.0E0
SMART	CYCLIN	RT	5	3.1E-1	1.0E0
INTERPRO	Cyclin, C-terminal	RT	3	8.0E-1	1.0E0
Annotation Cluster 4	Enrichment Score: 0.54	G	Count	P_Value	Benjamini
INTERPRO	Like-Sm ribonucleoprotein, core	RT	10	9.7E-2	1.0E0
INTERPRO	Like-Sm ribonucleoprotein, eukaryotic and archaea-type, core	RT	6	3.8E-1	1.0E0
SMART	Sm	RT	6	3.8E-1	1.0E0
SP_PIR_KEYWORDS	viral nucleoprotein	RT	10	5.1E-1	1.0E0
Annotation Cluster 5	Enrichment Score: 0.53	G	Count	P_Value	Benjamini
SP_PIR_KEYWORDS	ank repeat	RT	181	2.1E-1	1.0E0
SMART	ANK	RT	258	3.5E-1	1.0E0
INTERPRO	Ankyrin	RT	258	3.5E-1	1.0E0
Annotation Cluster 6	Enrichment Score: 0.48	G	Count	P_Value	Benjamini
GOTERM_BP_FAT	RNA modification	RT	14	3.9E-2	1.0E0
GOTERM_MF_FAT	pseudouridine synthase activity	RT	6	3.5E-1	1.0E0
GOTERM_BP_FAT	pseudouridine synthesis	RT	6	3.6E-1	1.0E0
PIR_SUPERFAMILY	PIRSF001430:tRNA_psdUrid_synth	RT	3	6.1E-1	1.0E0
INTERPRO	Pseudouridine synthase I, TruA	RT	3	6.2E-1	1.0E0
GOTERM_MF_FAT	intramolecular transferase activity	RT	7	6.8E-1	1.0E0
Annotation Cluster 7	Enrichment Score: 0.47	G	Count	P_Value	Benjamini
GOTERM_MF_FAT	protein serine/threonine kinase activity	RT	68	6.3E-2	1.0E0
SP_PIR_KEYWORDS	kinase	RT	200	6.4E-2	1.0E0
INTERPRO	Serine/threonine protein kinase, active site	RT	60	7.2E-2	1.0E0
GOTERM_MF_FAT	protein kinase activity	RT	163	1.2E-1	1.0E0
GOTERM_BP_FAT	protein amino acid phosphorylation	RT	162	1.2E-1	1.0E0
INTERPRO	Protein kinase, core	RT	162	2.1E-1	1.0E0
SP_PIR_KEYWORDS	serine/threonine-protein kinase	RT	39	2.3E-1	1.0E0

GOTERM_BP_FAT	phosphorylation	RT	170	2.3E-1	1.0E0
GOTERM_BP_FAT	phosphate metabolic process	RT	179	2.4E-1	1.0E0
GOTERM_BP_FAT	phosphorus metabolic process	RT	179	2.4E-1	1.0E0
INTERPRO	Protein kinase, ATP binding site	RT	52	2.7E-1	1.0E0
INTERPRO	Serine/threonine protein kinase-related	RT	140	3.2E-1	1.0E0
INTERPRO	Serine/threonine protein kinase	RT	36	4.3E-1	1.0E0
SMART	S_TKc	RT	36	4.3E-1	1.0E0
GOTERM_MF_FAT	nucleoside binding	RT	301	6.4E-1	1.0E0
GOTERM_MF_FAT	purine nucleoside binding	RT	298	6.7E-1	1.0E0
GOTERM_MF_FAT	adenyl nucleotide binding	RT	298	6.7E-1	1.0E0
GOTERM_MF_FAT	adenyl ribonucleotide binding	RT	294	6.7E-1	1.0E0
GOTERM_MF_FAT	ATP binding	RT	294	6.7E-1	1.0E0
GOTERM_MF_FAT	purine nucleotide binding	RT	320	8.4E-1	1.0E0
GOTERM_MF_FAT	ribonucleotide binding	RT	316	8.5E-1	1.0E0
GOTERM_MF_FAT	purine ribonucleotide binding	RT	316	8.5E-1	1.0E0
GOTERM_MF_FAT	nucleotide binding	RT	340	8.5E-1	1.0E0
SP_PIR_KEYWORDS	atp-binding	RT	139	8.6E-1	9.9E-1
SP_PIR_KEYWORDS	nucleotide-binding	RT	142	9.5E-1	1.0E0
Annotation Cluster 8	Enrichment Score: 0.39		Count	P_Value	Benjamini
GOTERM_MF_FAT	nucleotide kinase activity	RT	5	2.8E-1	1.0E0
GOTERM_MF_FAT	nucleobase, nucleoside, nucleotide kinase activity	RT	8	3.1E-1	1.0E0
GOTERM_MF_FAT	phosphotransferase activity, phosphate group as acceptor	RT	6	3.5E-1	1.0E0
GOTERM_MF_FAT	adenylate kinase activity	RT	3	6.0E-1	1.0E0
INTERPRO	Adenylate kinase	RT	3	6.2E-1	1.0E0
Annotation Cluster 9	Enrichment Score: 0.38		Count	P_Value	Benjamini
INTERPRO	Helicase, superfamily 1 and 2, ATP-binding	RT	29	2.2E-1	1.0E0
INTERPRO	DEAD-like helicase, N-terminal	RT	29	2.2E-1	1.0E0
SMART	DEXDc	RT	29	2.2E-1	1.0E0
INTERPRO	DNA/RNA helicase, C-terminal	RT	29	2.9E-1	1.0E0
SMART	HELICc	RT	29	2.9E-1	1.0E0
GOTERM_MF_FAT	helicase activity	RT	34	3.1E-1	1.0E0
SP_PIR_KEYWORDS	helicase	RT	28	3.2E-1	1.0E0
GOTERM_MF_FAT	purine NTP-dependent helicase activity	RT	23	5.2E-1	1.0E0
GOTERM_MF_FAT	ATP-dependent helicase activity	RT	23	5.2E-1	1.0E0
INTERPRO	DNA/RNA helicase, DEAD/DEAH box type, N-terminal	RT	18	6.5E-1	1.0E0
INTERPRO	RNA helicase, DEAD-box type, Q motif	RT	10	7.6E-1	1.0E0
GOTERM_MF_FAT	ATPase activity, coupled	RT	34	9.6E-1	1.0E0
GOTERM_MF_FAT	ATPase activity	RT	45	9.8E-1	1.0E0
Annotation Cluster 10	Enrichment Score: 0.37		Count	P_Value	Benjamini
GOTERM_CC_FAT	microtubule organizing center	RT	4	4.2E-1	1.0E0
GOTERM_CC_FAT	spindle	RT	4	4.2E-1	1.0E0
GOTERM_CC_FAT	spindle pole	RT	4	4.2E-1	1.0E0
GOTERM_BP_FAT	microtubule cytoskeleton organization	RT	4	4.2E-1	1.0E0
INTERPRO	Spc97/Spc98	RT	4	4.5E-1	1.0E0
Annotation Cluster 11	Enrichment Score: 0.36		Count	P_Value	Benjamini
GOTERM_BP_FAT	translational initiation	RT	9	2.3E-1	1.0E0
GOTERM_MF_FAT	translation initiation factor activity	RT	16	4.6E-1	1.0E0
SP_PIR_KEYWORDS	Initiation factor	RT	12	5.6E-1	1.0E0
GOTERM_MF_FAT	translation factor activity, nucleic acid binding	RT	19	6.0E-1	1.0E0
Annotation Cluster 12	Enrichment Score: 0.33		Count	P_Value	Benjamini
GOTERM_BP_FAT	RNA methylation	RT	5	2.9E-1	1.0E0
INTERPRO	Ribosomal RNA methyltransferase RrmJ/FtsJ	RT	4	4.5E-1	1.0E0
GOTERM_BP_FAT	biopolymer methylation	RT	5	4.8E-1	1.0E0
GOTERM_BP_FAT	methylation	RT	7	5.6E-1	1.0E0
GOTERM_BP_FAT	one-carbon metabolic process	RT	7	6.9E-1	1.0E0
Annotation Cluster 13	Enrichment Score: 0.32		Count	P_Value	Benjamini
INTERPRO	EGF	RT	6	3.8E-1	1.0E0

	INTERPRO	EGF, extracellular	RT		15	4.9E-1	1.0E0
	INTERPRO	EGF-like, type 3	RT		20	5.2E-1	1.0E0
	SP_PIR_KEYWORDS	egf-like domain	RT		11	5.4E-1	1.0E0
	Annotation Cluster 14	Enrichment Score: 0.32	G		Count	P_Value	Benjamini
	GOTERM_BP_FAT	RNA capping	RT		5	2.9E-1	1.0E0
	GOTERM_BP_FAT	mRNA metabolic process	RT		5	4.8E-1	1.0E0
	GOTERM_BP_FAT	mRNA capping	RT		3	6.1E-1	1.0E0
	GOTERM_BP_FAT	mRNA processing	RT		4	6.3E-1	1.0E0
	Annotation Cluster 15	Enrichment Score: 0.32	G		Count	P_Value	Benjamini
	GOTERM_BP_FAT	protein amino acid dephosphorylation	RT		10	2.7E-1	1.0E0
	GOTERM_MF_FAT	protein tyrosine/serine/threonine phosphatase activity	RT		5	2.8E-1	1.0E0
	INTERPRO	Dual specificity phosphatase, catalytic domain	RT		5	3.1E-1	1.0E0
	INTERPRO	Protein-tyrosine phosphatase, dual specificity	RT		5	3.1E-1	1.0E0
	GOTERM_BP_FAT	dephosphorylation	RT		10	6.3E-1	1.0E0
	INTERPRO	Protein-tyrosine phosphatase	RT		4	6.5E-1	1.0E0
	INTERPRO	Dual-specific/protein-tyrosine phosphatase, conserved region	RT		4	6.5E-1	1.0E0
	INTERPRO	Protein-tyrosine phosphatase, active site	RT		4	8.0E-1	1.0E0
	GOTERM_MF_FAT	protein tyrosine phosphatase activity	RT		4	8.7E-1	1.0E0
	Annotation Cluster 16	Enrichment Score: 0.3	G		Count	P_Value	Benjamini
	KEGG_PATHWAY	Pyrimidine metabolism	RT		21	3.4E-1	1.0E0
	GOTERM_BP_FAT	transcription	RT		17	4.0E-1	1.0E0
	KEGG_PATHWAY	RNA polymerase	RT		12	4.1E-1	1.0E0
	GOTERM_MF_FAT	DNA-directed RNA polymerase activity	RT		15	4.3E-1	1.0E0
	GOTERM_MF_FAT	RNA polymerase activity	RT		15	5.3E-1	1.0E0
	KEGG_PATHWAY	Purine metabolism	RT		19	5.5E-1	1.0E0
	SP_PIR_KEYWORDS	dna-directed rna polymerase	RT		9	6.0E-1	1.0E0
	SP_PIR_KEYWORDS	nucleotidyltransferase	RT		11	9.7E-1	1.0E0
	Annotation Cluster 17	Enrichment Score: 0.3	G		Count	P_Value	Benjamini
	GOTERM_BP_FAT	protein amino acid dephosphorylation	RT		10	2.7E-1	1.0E0
	GOTERM_MF_FAT	iron ion binding	RT		20	3.8E-1	1.0E0
	GOTERM_MF_FAT	phosphoprotein phosphatase activity	RT		18	4.1E-1	1.0E0
	GOTERM_MF_FAT	phosphatase activity	RT		27	4.4E-1	1.0E0
	INTERPRO	Metallophosphoesterase	RT		14	4.6E-1	1.0E0
	PIR_SUPERFAMILY	PIRSF000908:serine/threonine-protein phosphatase, conventional type	RT		6	5.3E-1	1.0E0
	GOTERM_BP_FAT	dephosphorylation	RT		10	6.3E-1	1.0E0
	SP_PIR_KEYWORDS	iron	RT		12	6.5E-1	9.9E-1
	INTERPRO	Serine/threonine-specific protein phosphatase and bis(5-nucleosyl)-tetraphosphatase	RT		9	7.4E-1	1.0E0
	SMART	PP2Ac	RT		9	7.5E-1	1.0E0
	Annotation Cluster 18	Enrichment Score: 0.29	G		Count	P_Value	Benjamini
	SP_PIR_KEYWORDS	tpr repeat	RT		7	4.0E-1	1.0E0
	INTERPRO	Tetratricopeptide region	RT		16	4.2E-1	1.0E0
	INTERPRO	Tetratricopeptide TPR-1	RT		13	5.3E-1	1.0E0
	INTERPRO	Tetratricopeptide repeat	RT		15	5.8E-1	1.0E0
	INTERPRO	Tetratricopeptide-like helical	RT		19	5.9E-1	1.0E0
	SMART	TPR	RT		15	5.9E-1	1.0E0
	Annotation Cluster 19	Enrichment Score: 0.28	G		Count	P_Value	Benjamini
	INTERPRO	Furin-like repeat	RT		110	4.2E-1	1.0E0
	SMART	FU	RT		110	4.3E-1	1.0E0
	INTERPRO	Giardia variant-specific surface protein	RT		138	5.6E-1	1.0E0
	INTERPRO	EGF-like	RT		110	6.3E-1	1.0E0
	SMART	EGF	RT		110	6.4E-1	1.0E0
	Annotation Cluster 20	Enrichment Score: 0.27	G		Count	P_Value	Benjamini
	GOTERM_BP_FAT	purine nucleoside metabolic process	RT		4	4.2E-1	1.0E0
	GOTERM_BP_FAT	purine ribonucleoside metabolic process	RT		4	4.2E-1	1.0E0
	GOTERM_BP_FAT	ribonucleoside metabolic process	RT		5	6.5E-1	1.0E0
	GOTERM_BP_FAT	nucleoside metabolic process	RT		8	7.0E-1	1.0E0
	Annotation Cluster 21	Enrichment Score: 0.26	G		Count	P_Value	Benjamini

INTERPRO	SANT, DNA-binding	RT	8	3.4E-1	1.0E0
SMART	SANT	RT	8	3.4E-1	1.0E0
INTERPRO	Myb, DNA-binding	RT	4	6.5E-1	1.0E0
INTERPRO	Myb transcription factor	RT	3	8.0E-1	1.0E0
INTERPRO	Myb-type HTH DNA-binding domain	RT	3	8.0E-1	1.0E0
Annotation Cluster 22	Enrichment Score: 0.26		Count	P_Value	Benjamini
GOTERM_BP_FAT	cellular response to stress	RT	14	4.1E-1	1.0E0
GOTERM_BP_FAT	DNA repair	RT	13	4.9E-1	1.0E0
GOTERM_BP_FAT	response to DNA damage stimulus	RT	13	4.9E-1	1.0E0
GOTERM_BP_FAT	DNA metabolic process	RT	30	9.2E-1	1.0E0
Annotation Cluster 23	Enrichment Score: 0.26		Count	P_Value	Benjamini
INTERPRO	Protein of unknown function DUF6, transmembrane	RT	4	4.5E-1	1.0E0
PIR_SUPERFAMILY	PIRSF036436:UCP036436	RT	3	6.1E-1	1.0E0
INTERPRO	Uncharacterised conserved protein UCP036436, nucleotide-sugar transporter-related	RT	3	6.2E-1	1.0E0
Annotation Cluster 24	Enrichment Score: 0.25		Count	P_Value	Benjamini
INTERPRO	Radical SAM	RT	4	4.5E-1	1.0E0
INTERPRO	Elongator protein 3/MiaB/NifB	RT	3	6.2E-1	1.0E0
SMART	Elp3	RT	3	6.2E-1	1.0E0
Annotation Cluster 25	Enrichment Score: 0.25		Count	P_Value	Benjamini
GOTERM_BP_FAT	nucleotide catabolic process	RT	4	4.2E-1	1.0E0
GOTERM_BP_FAT	nitrogen compound catabolic process	RT	5	4.8E-1	1.0E0
GOTERM_BP_FAT	nucleobase, nucleoside and nucleotide catabolic process	RT	5	4.8E-1	1.0E0
GOTERM_BP_FAT	nucleobase, nucleoside, nucleotide and nucleic acid catabolic process	RT	5	4.8E-1	1.0E0
PIR_SUPERFAMILY	PIRSF017316:phosphoesterase, C1039.02 type	RT	3	6.1E-1	1.0E0
PIR_SUPERFAMILY	PIRSF017316:Pesterase_C1039	RT	3	6.1E-1	1.0E0
INTERPRO	5'-Nucleotidase and apyrase	RT	3	6.2E-1	1.0E0
INTERPRO	Predicted phosphoesterase, C1039.02 type	RT	3	6.2E-1	1.0E0
COG_ONTOLOGY	Nucleotide transport and metabolism	RT	5	8.2E-1	1.0E0
Annotation Cluster 26	Enrichment Score: 0.24		Count	P_Value	Benjamini
GOTERM_BP_FAT	ribosome biogenesis	RT	13	4.9E-1	1.0E0
GOTERM_BP_FAT	ribonucleoprotein complex biogenesis	RT	13	4.9E-1	1.0E0
GOTERM_BP_FAT	ncRNA processing	RT	18	5.1E-1	1.0E0
GOTERM_BP_FAT	rRNA modification	RT	3	6.1E-1	1.0E0
GOTERM_BP_FAT	rRNA metabolic process	RT	8	7.0E-1	1.0E0
GOTERM_BP_FAT	rRNA processing	RT	8	7.0E-1	1.0E0
Annotation Cluster 27	Enrichment Score: 0.24		Count	P_Value	Benjamini
INTERPRO	EGF-like, type 3	RT	20	5.2E-1	1.0E0
INTERPRO	EGF-like, laminin	RT	16	6.1E-1	1.0E0
SMART	EGF_Lam	RT	16	6.1E-1	1.0E0
Annotation Cluster 28	Enrichment Score: 0.23		Count	P_Value	Benjamini
GOTERM_BP_FAT	pyridine nucleotide biosynthetic process	RT	4	4.2E-1	1.0E0
GOTERM_BP_FAT	pyridine nucleotide metabolic process	RT	5	4.8E-1	1.0E0
GOTERM_BP_FAT	oxidoreduction coenzyme metabolic process	RT	5	4.8E-1	1.0E0
GOTERM_BP_FAT	coenzyme metabolic process	RT	7	5.6E-1	1.0E0
GOTERM_BP_FAT	cofactor metabolic process	RT	8	5.9E-1	1.0E0
GOTERM_BP_FAT	NAD metabolic process	RT	3	6.1E-1	1.0E0
GOTERM_BP_FAT	NAD biosynthetic process	RT	3	6.1E-1	1.0E0
GOTERM_BP_FAT	nicotinamide nucleotide biosynthetic process	RT	3	6.1E-1	1.0E0
GOTERM_BP_FAT	water-soluble vitamin biosynthetic process	RT	4	6.3E-1	1.0E0
GOTERM_BP_FAT	vitamin metabolic process	RT	4	6.3E-1	1.0E0
GOTERM_BP_FAT	secondary metabolic process	RT	4	6.3E-1	1.0E0
GOTERM_BP_FAT	water-soluble vitamin metabolic process	RT	4	6.3E-1	1.0E0
GOTERM_BP_FAT	nicotinamide metabolic process	RT	4	6.3E-1	1.0E0
GOTERM_BP_FAT	nicotinamide nucleotide metabolic process	RT	4	6.3E-1	1.0E0
GOTERM_BP_FAT	alkaloid metabolic process	RT	4	6.3E-1	1.0E0
GOTERM_BP_FAT	cellular amide metabolic process	RT	4	6.3E-1	1.0E0
GOTERM_BP_FAT	vitamin biosynthetic process	RT	4	6.3E-1	1.0E0

GOTERM_BP_FAT	coenzyme biosynthetic process	RT	5	6.5E-1	1.0E0
GOTERM_BP_FAT	cofactor biosynthetic process	RT	6	6.7E-1	1.0E0
Annotation Cluster 29	Enrichment Score: 0.22		Count	P_Value	Benjamini
SP_PIR_KEYWORDS	transmembrane	RT	18	5.8E-1	1.0E0
INTERPRO	Amino acid transporter, transmembrane	RT	7	5.9E-1	1.0E0
GOTERM_CC_FAT	integral to membrane	RT	38	6.0E-1	1.0E0
GOTERM_CC_FAT	intrinsic to membrane	RT	38	6.0E-1	1.0E0
SP_PIR_KEYWORDS	membrane	RT	16	6.3E-1	9.9E-1
Annotation Cluster 30	Enrichment Score: 0.21		Count	P_Value	Benjamini
INTERPRO	Protein phosphatase 2C, N-terminal	RT	5	5.1E-1	1.0E0
INTERPRO	Protein phosphatase 2C-related	RT	5	5.1E-1	1.0E0
INTERPRO	Protein phosphatase 2C	RT	5	5.1E-1	1.0E0
SMART	PP2C_SIG	RT	5	5.1E-1	1.0E0
SMART	PP2Cc	RT	5	5.1E-1	1.0E0
SP_PIR_KEYWORDS	magnesium	RT	8	5.8E-1	1.0E0
GOTERM_CC_FAT	protein serine/threonine phosphatase complex	RT	5	6.4E-1	1.0E0
GOTERM_MF_FAT	magnesium ion binding	RT	9	7.1E-1	1.0E0
SP_PIR_KEYWORDS	protein phosphatase	RT	3	7.8E-1	9.9E-1
GOTERM_MF_FAT	protein serine/threonine phosphatase activity	RT	3	7.8E-1	1.0E0
INTERPRO	Protein phosphatase 2C, manganese/magnesium aspartate binding site	RT	3	8.0E-1	1.0E0
Annotation Cluster 31	Enrichment Score: 0.21		Count	P_Value	Benjamini
GOTERM_MF_FAT	lipase activity	RT	3	6.0E-1	1.0E0
GOTERM_MF_FAT	triacylglycerol lipase activity	RT	3	6.0E-1	1.0E0
INTERPRO	Lipase, class 3	RT	3	6.2E-1	1.0E0
GOTERM_MF_FAT	carboxylesterase activity	RT	4	6.3E-1	1.0E0
Annotation Cluster 32	Enrichment Score: 0.21		Count	P_Value	Benjamini
SP_PIR_KEYWORDS	Transcription	RT	6	5.2E-1	1.0E0
SP_PIR_KEYWORDS	dna-directed rna polymerase	RT	9	6.0E-1	1.0E0
INTERPRO	DNA-directed RNA polymerase, subunit 2	RT	3	6.2E-1	1.0E0
INTERPRO	RNA polymerase, beta subunit, conserved site	RT	3	6.2E-1	1.0E0
INTERPRO	DNA-directed RNA polymerase, subunit 2, domain 6	RT	3	6.2E-1	1.0E0
INTERPRO	RNA polymerase Rpb2, domain 3	RT	3	6.2E-1	1.0E0
INTERPRO	RNA polymerase, beta subunit, protrusion	RT	3	6.2E-1	1.0E0
INTERPRO	RNA polymerase Rpb2, domain 7	RT	3	6.2E-1	1.0E0
GOTERM_MF_FAT	ribonucleoside binding	RT	3	7.8E-1	1.0E0
Annotation Cluster 33	Enrichment Score: 0.2		Count	P_Value	Benjamini
GOTERM_MF_FAT	enzyme activator activity	RT	7	4.0E-1	1.0E0
GOTERM_BP_FAT	regulation of small GTPase mediated signal transduction	RT	7	4.1E-1	1.0E0
GOTERM_BP_FAT	regulation of Ras protein signal transduction	RT	7	4.1E-1	1.0E0
GOTERM_MF_FAT	nucleoside-triphosphatase regulator activity	RT	9	4.8E-1	1.0E0
GOTERM_BP_FAT	regulation of ARF protein signal transduction	RT	4	6.3E-1	1.0E0
GOTERM_MF_FAT	GTPase activator activity	RT	5	6.5E-1	1.0E0
GOTERM_BP_FAT	regulation of Ras GTPase activity	RT	5	6.5E-1	1.0E0
GOTERM_MF_FAT	GTPase regulator activity	RT	7	6.8E-1	1.0E0
GOTERM_MF_FAT	small GTPase regulator activity	RT	7	6.8E-1	1.0E0
GOTERM_BP_FAT	regulation of GTPase activity	RT	5	7.8E-1	1.0E0
GOTERM_BP_FAT	regulation of hydrolase activity	RT	5	7.8E-1	1.0E0
GOTERM_MF_FAT	Rab GTPase activator activity	RT	3	7.8E-1	1.0E0
GOTERM_MF_FAT	Ras GTPase activator activity	RT	3	7.8E-1	1.0E0
GOTERM_BP_FAT	regulation of Rab protein signal transduction	RT	3	7.9E-1	1.0E0
GOTERM_BP_FAT	regulation of Rab GTPase activity	RT	3	7.9E-1	1.0E0
Annotation Cluster 34	Enrichment Score: 0.19		Count	P_Value	Benjamini
GOTERM_BP_FAT	RNA processing	RT	28	4.7E-1	1.0E0
GOTERM_BP_FAT	ncRNA processing	RT	18	5.1E-1	1.0E0
GOTERM_BP_FAT	tRNA processing	RT	10	7.3E-1	1.0E0
GOTERM_BP_FAT	ncRNA metabolic process	RT	29	7.3E-1	1.0E0

GOTERM_BP_FAT	tRNA metabolic process	RT	21	8.6E-1	1.0E0
Annotation Cluster 35	Enrichment Score: 0.19		Count	P_Value	Benjamini
INTERPRO	RNA recognition motif, RNP-1	RT	9	6.4E-1	1.0E0
SMART	RRM	RT	9	6.5E-1	1.0E0
INTERPRO	Nucleotide-binding, alpha-beta plait	RT	10	6.7E-1	1.0E0
Annotation Cluster 36	Enrichment Score: 0.18		Count	P_Value	Benjamini
GOTERM_MF_FAT	antiporter activity	RT	5	4.8E-1	1.0E0
GOTERM_MF_FAT	drug transporter activity	RT	4	6.3E-1	1.0E0
GOTERM_BP_FAT	drug transport	RT	4	6.3E-1	1.0E0
GOTERM_BP_FAT	response to drug	RT	4	6.3E-1	1.0E0
GOTERM_BP_FAT	multidrug transport	RT	4	6.3E-1	1.0E0
INTERPRO	Multi antimicrobial extrusion protein MatE	RT	4	6.5E-1	1.0E0
INTERPRO	Na+ driven multidrug efflux pump	RT	3	8.0E-1	1.0E0
COG_ONTOLOGY	Defense mechanisms	RT	12	8.7E-1	1.0E0
Annotation Cluster 37	Enrichment Score: 0.18		Count	P_Value	Benjamini
GOTERM_BP_FAT	hexose catabolic process	RT	9	6.1E-1	1.0E0
GOTERM_BP_FAT	alcohol catabolic process	RT	9	6.1E-1	1.0E0
GOTERM_BP_FAT	monosaccharide catabolic process	RT	9	6.1E-1	1.0E0
GOTERM_BP_FAT	cellular carbohydrate catabolic process	RT	9	6.1E-1	1.0E0
GOTERM_BP_FAT	glucose catabolic process	RT	9	6.1E-1	1.0E0
GOTERM_BP_FAT	carbohydrate catabolic process	RT	9	6.1E-1	1.0E0
GOTERM_BP_FAT	hexose metabolic process	RT	12	6.6E-1	1.0E0
GOTERM_BP_FAT	glucose metabolic process	RT	12	6.6E-1	1.0E0
GOTERM_BP_FAT	glycolysis	RT	7	6.9E-1	1.0E0
GOTERM_BP_FAT	generation of precursor metabolites and energy	RT	15	7.1E-1	1.0E0
SP_PIR_KEYWORDS	glycolysis	RT	4	7.7E-1	9.9E-1
GOTERM_BP_FAT	monosaccharide metabolic process	RT	12	8.7E-1	1.0E0
Annotation Cluster 38	Enrichment Score: 0.17		Count	P_Value	Benjamini
GOTERM_BP_FAT	chromatin organization	RT	11	3.1E-1	1.0E0
GOTERM_BP_FAT	chromosome organization	RT	13	3.8E-1	1.0E0
GOTERM_BP_FAT	chromatin assembly or disassembly	RT	9	4.9E-1	1.0E0
GOTERM_CC_FAT	chromatin	RT	8	5.8E-1	1.0E0
PIR_SUPERFAMILY	PIRSF002052:histone H4	RT	3	6.1E-1	1.0E0
INTERPRO	Histone H4	RT	3	6.2E-1	1.0E0
SMART	H4	RT	3	6.2E-1	1.0E0
INTERPRO	Histone-fold	RT	9	6.4E-1	1.0E0
GOTERM_CC_FAT	nucleosome	RT	7	6.8E-1	1.0E0
SP_PIR_KEYWORDS	chromosomal protein	RT	7	6.8E-1	9.9E-1
GOTERM_BP_FAT	protein-DNA complex assembly	RT	7	6.9E-1	1.0E0
GOTERM_BP_FAT	chromatin assembly	RT	7	6.9E-1	1.0E0
GOTERM_BP_FAT	DNA packaging	RT	7	6.9E-1	1.0E0
GOTERM_BP_FAT	nucleosome organization	RT	7	6.9E-1	1.0E0
GOTERM_BP_FAT	nucleosome assembly	RT	7	6.9E-1	1.0E0
GOTERM_CC_FAT	protein-DNA complex	RT	8	6.9E-1	1.0E0
GOTERM_BP_FAT	cellular macromolecular complex assembly	RT	14	6.9E-1	1.0E0
GOTERM_BP_FAT	macromolecular complex assembly	RT	15	7.1E-1	1.0E0
INTERPRO	Histone core	RT	7	7.1E-1	1.0E0
GOTERM_BP_FAT	cellular macromolecular complex subunit organization	RT	14	7.7E-1	1.0E0
GOTERM_CC_FAT	chromosome	RT	15	7.7E-1	1.0E0
GOTERM_BP_FAT	macromolecular complex subunit organization	RT	15	7.7E-1	1.0E0
INTERPRO	Histone H3	RT	3	8.0E-1	1.0E0
SMART	H3	RT	3	8.0E-1	1.0E0
GOTERM_CC_FAT	chromosomal part	RT	9	8.6E-1	1.0E0
SP_PIR_KEYWORDS	nucleosome core	RT	4	8.7E-1	9.9E-1
SP_PIR_KEYWORDS	dna-binding	RT	18	8.8E-1	9.9E-1
SP_PIR_KEYWORDS	nucleus	RT	17	9.1E-1	9.9E-1
Annotation Cluster 39	Enrichment Score: 0.17		Count	P_Value	Benjamini

	Category	Term	RT			Count	P_Value	Benjamini
	INTERPRO	EF-Hand type	RT			12	5.0E-1	1.0E0
	INTERPRO	EF-HAND 2	RT			11	5.8E-1	1.0E0
	COG_ONTOLOGY	Signal transduction mechanisms / Cytoskeleton / Cell division and chromosome partitioning / General function prediction only	RT			5	7.0E-1	1.0E0
	INTERPRO	EF-HAND 1	RT			12	7.0E-1	1.0E0
	SP_PIR_KEYWORDS	calcium	RT			9	7.1E-1	9.9E-1
	INTERPRO	Calcium-binding EF-hand	RT			7	7.1E-1	1.0E0
	SMART	EFh	RT			7	7.2E-1	1.0E0
	INTERPRO	EF hand	RT			6	8.8E-1	1.0E0
	Annotation Cluster 40	Enrichment Score: 0.17	G			Count	P_Value	Benjamini
	SP_PIR_KEYWORDS	protein biosynthesis	RT			7	5.5E-1	1.0E0
	PIR_SUPERFAMILY	PIRSF001525:lysine-tRNA ligase	RT			3	6.1E-1	1.0E0
	INTERPRO	Aminoacyl-tRNA synthetase, class II (D, K and N)	RT			3	6.2E-1	1.0E0
	INTERPRO	Aminoacyl-tRNA synthetase, class II (D, K and N)-like	RT			3	6.2E-1	1.0E0
	INTERPRO	Nucleic acid binding, OB-fold, tRNA/helicase-type	RT			3	8.0E-1	1.0E0
	INTERPRO	Aminoacyl-tRNA synthetase, class II, conserved region	RT			4	9.7E-1	1.0E0
	Annotation Cluster 41	Enrichment Score: 0.17	G			Count	P_Value	Benjamini
	SP_PIR_KEYWORDS	dipeptidase	RT			3	6.0E-1	9.9E-1
	GOTERM_MF_FAT	dipeptidase activity	RT			3	6.0E-1	1.0E0
	GOTERM_MF_FAT	exopeptidase activity	RT			7	6.8E-1	1.0E0
	GOTERM_MF_FAT	metallopeptidase activity	RT			8	6.9E-1	1.0E0
	GOTERM_MF_FAT	metalloexopeptidase activity	RT			4	8.7E-1	1.0E0
	Annotation Cluster 42	Enrichment Score: 0.16	G			Count	P_Value	Benjamini
	GOTERM_MF_FAT	cation binding	RT			149	4.2E-1	1.0E0
	GOTERM_MF_FAT	ion binding	RT			149	4.2E-1	1.0E0
	GOTERM_MF_FAT	metal ion binding	RT			146	4.9E-1	1.0E0
	INTERPRO	Zinc finger, C3HC4 RING-type	RT			21	6.2E-1	1.0E0
	GOTERM_MF_FAT	transition metal ion binding	RT			103	7.7E-1	1.0E0
	SP_PIR_KEYWORDS	zinc-finger	RT			33	8.0E-1	9.9E-1
	INTERPRO	Zinc finger, RING-type	RT			37	8.1E-1	1.0E0
	SMART	RING	RT			37	8.2E-1	1.0E0
	GOTERM_MF_FAT	zinc ion binding	RT			80	9.0E-1	1.0E0
	SP_PIR_KEYWORDS	zinc	RT			42	9.5E-1	1.0E0
	SP_PIR_KEYWORDS	metal-binding	RT			50	9.6E-1	1.0E0
	Annotation Cluster 43	Enrichment Score: 0.16	G			Count	P_Value	Benjamini
	GOTERM_MF_FAT	fatty-acid ligase activity	RT			4	6.3E-1	1.0E0
	GOTERM_MF_FAT	long-chain-fatty-acid-CoA ligase activity	RT			4	6.3E-1	1.0E0
	GOTERM_MF_FAT	ligase activity, forming carbon-sulfur bonds	RT			4	6.3E-1	1.0E0
	COG_ONTOLOGY	Lipid metabolism	RT			8	7.5E-1	1.0E0
	INTERPRO	AMP-dependent synthetase and ligase	RT			4	8.9E-1	1.0E0
	Annotation Cluster 44	Enrichment Score: 0.14	G			Count	P_Value	Benjamini
	SP_PIR_KEYWORDS	heme	RT			4	6.3E-1	9.9E-1
	GOTERM_MF_FAT	heme binding	RT			5	6.5E-1	1.0E0
	GOTERM_MF_FAT	tetrapyrrole binding	RT			5	6.5E-1	1.0E0
	INTERPRO	Cytochrome b5, heme-binding site	RT			3	8.0E-1	1.0E0
	INTERPRO	Cytochrome b5	RT			3	9.0E-1	1.0E0
	Annotation Cluster 45	Enrichment Score: 0.14	G			Count	P_Value	Benjamini
	INTERPRO	Zinc finger, C2H2-like	RT			11	6.9E-1	1.0E0
	SMART	ZnF_C2H2	RT			11	6.9E-1	1.0E0
	INTERPRO	Zinc finger, C2H2-type	RT			10	8.3E-1	1.0E0
	Annotation Cluster 46	Enrichment Score: 0.13	G			Count	P_Value	Benjamini
	GOTERM_MF_FAT	acetyltransferase activity	RT			7	5.5E-1	1.0E0
	GOTERM_MF_FAT	N-acetyltransferase activity	RT			6	6.6E-1	1.0E0
	INTERPRO	GCN5-related N-acetyltransferase	RT			6	7.0E-1	1.0E0
	GOTERM_MF_FAT	N-acyltransferase activity	RT			6	7.8E-1	1.0E0
	SP_PIR_KEYWORDS	Acyltransferase	RT			6	8.6E-1	1.0E0
	INTERPRO	Acyl-CoA N-acyltransferase	RT			5	9.3E-1	1.0E0

				Count	P_Value	Benjamini
Annotation Cluster 47		Enrichment Score: 0.13		Count	P_Value	Benjamini
	INTERPRO	Ubiquitin-conjugating enzyme, E2	RT	9	3.9E-1	1.0E0
	INTERPRO	Ubiquitin-conjugating enzyme/RWD-like	RT	9	3.9E-1	1.0E0
	SMART	UBCc	RT	9	3.9E-1	1.0E0
	SP_PIR_KEYWORDS	ligase	RT	33	4.7E-1	1.0E0
	GOTERM_MF_FAT	acid-amino acid ligase activity	RT	17	4.8E-1	1.0E0
	GOTERM_MF_FAT	small conjugating protein ligase activity	RT	9	4.8E-1	1.0E0
	GOTERM_MF_FAT	ligase activity, forming carbon-nitrogen bonds	RT	19	6.0E-1	1.0E0
	SP_PIR_KEYWORDS	ubl conjugation pathway	RT	8	6.9E-1	9.9E-1
	GOTERM_CC_FAT	proteasome complex	RT	12	7.4E-1	1.0E0
	GOTERM_CC_FAT	cytosol	RT	16	7.8E-1	1.0E0
	PIR_SUPERFAMILY	PIRSF001567:ubiquitin-protein ligase E2	RT	4	7.8E-1	1.0E0
	KEGG_PATHWAY	Proteasome	RT	15	8.0E-1	1.0E0
	SP_PIR_KEYWORDS	proteasome	RT	11	8.0E-1	9.9E-1
	GOTERM_BP_FAT	macromolecule catabolic process	RT	21	8.2E-1	1.0E0
	GOTERM_BP_FAT	modification-dependent protein catabolic process	RT	14	8.3E-1	1.0E0
	GOTERM_BP_FAT	modification-dependent macromolecule catabolic process	RT	14	8.3E-1	1.0E0
	GOTERM_BP_FAT	cellular macromolecule catabolic process	RT	18	8.5E-1	1.0E0
	GOTERM_BP_FAT	protein catabolic process	RT	20	8.6E-1	1.0E0
	INTERPRO	Proteasome, alpha and beta subunits	RT	7	8.8E-1	1.0E0
	GOTERM_BP_FAT	proteolysis involved in cellular protein catabolic process	RT	17	8.8E-1	1.0E0
	GOTERM_BP_FAT	cellular protein catabolic process	RT	17	8.8E-1	1.0E0
	INTERPRO	Proteasome, alpha-subunit, conserved site	RT	4	8.9E-1	1.0E0
	GOTERM_MF_FAT	ubiquitin-protein ligase activity	RT	3	8.9E-1	1.0E0
	GOTERM_MF_FAT	threonine-type peptidase activity	RT	7	9.1E-1	1.0E0
	GOTERM_MF_FAT	threonine-type endopeptidase activity	RT	7	9.1E-1	1.0E0
	GOTERM_CC_FAT	proteasome core complex	RT	7	9.1E-1	1.0E0
	INTERPRO	Proteasome, subunit alpha/beta	RT	7	9.2E-1	1.0E0
	GOTERM_BP_FAT	proteolysis	RT	45	9.3E-1	1.0E0
	SP_PIR_KEYWORDS	threonine protease	RT	3	9.4E-1	1.0E0
	PIR_SUPERFAMILY	PIRSF001212:multicatalytic endopeptidase complex chain C9	RT	3	9.5E-1	1.0E0
	GOTERM_BP_FAT	ubiquitin-dependent protein catabolic process	RT	6	9.7E-1	1.0E0
Annotation Cluster 48		Enrichment Score: 0.13		Count	P_Value	Benjamini
	INTERPRO	von Willebrand factor, type A	RT	5	6.8E-1	1.0E0
	SMART	VWA	RT	5	6.8E-1	1.0E0
	INTERPRO	Copine	RT	3	9.0E-1	1.0E0
Annotation Cluster 49		Enrichment Score: 0.13		Count	P_Value	Benjamini
	GOTERM_BP_FAT	cell redox homeostasis	RT	11	6.5E-1	1.0E0
	GOTERM_BP_FAT	homeostatic process	RT	12	6.6E-1	1.0E0
	INTERPRO	Thioredoxin fold	RT	13	7.2E-1	1.0E0
	GOTERM_BP_FAT	cellular homeostasis	RT	11	7.4E-1	1.0E0
	INTERPRO	Thioredoxin-like	RT	7	8.8E-1	1.0E0
	INTERPRO	Thioredoxin, core	RT	4	8.9E-1	1.0E0
Annotation Cluster 50		Enrichment Score: 0.12		Count	P_Value	Benjamini
	GOTERM_MF_FAT	electron carrier activity	RT	14	6.0E-1	1.0E0
	GOTERM_MF_FAT	iron-sulfur cluster binding	RT	12	6.6E-1	1.0E0
	GOTERM_MF_FAT	metal cluster binding	RT	12	6.6E-1	1.0E0
	GOTERM_MF_FAT	4 iron, 4 sulfur cluster binding	RT	5	7.7E-1	1.0E0
	INTERPRO	4Fe-4S ferredoxin, iron-sulpur binding domain	RT	5	8.8E-1	1.0E0
	INTERPRO	4Fe-4S ferredoxin, iron-sulphur binding, subgroup	RT	5	8.8E-1	1.0E0
	INTERPRO	4Fe-4S ferredoxin, iron-sulphur binding, conserved site	RT	4	8.9E-1	1.0E0
Annotation Cluster 51		Enrichment Score: 0.12		Count	P_Value	Benjamini
	GOTERM_CC_FAT	coated membrane	RT	10	3.8E-1	1.0E0
	GOTERM_CC_FAT	membrane coat	RT	10	3.8E-1	1.0E0
	GOTERM_CC_FAT	transport vesicle membrane	RT	4	6.2E-1	1.0E0
	GOTERM_CC_FAT	transport vesicle	RT	4	6.2E-1	1.0E0

GOTERM_BP_FAT	vesicle-mediated transport	RT	17	6.6E-1	1.0E0
GOTERM_CC_FAT	Golgi apparatus part	RT	5	7.7E-1	1.0E0
GOTERM_CC_FAT	membrane-bounded vesicle	RT	4	7.7E-1	1.0E0
GOTERM_CC_FAT	vesicle	RT	4	7.7E-1	1.0E0
GOTERM_CC_FAT	cytoplasmic vesicle part	RT	4	7.7E-1	1.0E0
GOTERM_CC_FAT	cytoplasmic membrane-bounded vesicle	RT	4	7.7E-1	1.0E0
GOTERM_CC_FAT	vesicle membrane	RT	4	7.7E-1	1.0E0
GOTERM_CC_FAT	cytoplasmic vesicle	RT	4	7.7E-1	1.0E0
GOTERM_CC_FAT	cytoplasmic vesicle membrane	RT	4	7.7E-1	1.0E0
GOTERM_CC_FAT	coated vesicle membrane	RT	4	7.7E-1	1.0E0
GOTERM_CC_FAT	coated vesicle	RT	4	7.7E-1	1.0E0
GOTERM_CC_FAT	vesicle coat	RT	4	7.7E-1	1.0E0
GOTERM_BP_FAT	Golgi vesicle transport	RT	4	7.8E-1	1.0E0
GOTERM_BP_FAT	ER to Golgi vesicle-mediated transport	RT	4	7.8E-1	1.0E0
GOTERM_CC_FAT	ER to Golgi transport vesicle membrane	RT	3	7.8E-1	1.0E0
GOTERM_CC_FAT	ER to Golgi transport vesicle	RT	3	7.8E-1	1.0E0
GOTERM_CC_FAT	COPII vesicle coat	RT	3	7.8E-1	1.0E0
GOTERM_CC_FAT	endomembrane system	RT	9	7.9E-1	1.0E0
GOTERM_BP_FAT	intracellular transport	RT	15	8.3E-1	1.0E0
GOTERM_BP_FAT	protein localization	RT	18	8.5E-1	1.0E0
GOTERM_CC_FAT	organelle membrane	RT	6	8.6E-1	1.0E0
GOTERM_BP_FAT	intracellular protein transport	RT	12	8.7E-1	1.0E0
GOTERM_BP_FAT	cellular macromolecule localization	RT	12	8.7E-1	1.0E0
GOTERM_BP_FAT	cellular protein localization	RT	12	8.7E-1	1.0E0
GOTERM_BP_FAT	establishment of protein localization	RT	17	8.8E-1	1.0E0
GOTERM_BP_FAT	protein transport	RT	17	8.8E-1	1.0E0
GOTERM_CC_FAT	Golgi apparatus	RT	5	9.5E-1	1.0E0
Annotation Cluster 52	Enrichment Score: 0.12	G	Count	P_Value	Benjamini
GOTERM_MF_FAT	endoribonuclease activity	RT	4	6.3E-1	1.0E0
GOTERM_MF_FAT	ribonuclease activity	RT	6	7.8E-1	1.0E0
GOTERM_MF_FAT	endoribonuclease activity, producing 5'-phosphomonoesters	RT	3	7.8E-1	1.0E0
GOTERM_MF_FAT	endonuclease activity, active with either ribo- or deoxyribonucleic acids and producing 5'-phosphomonoesters	RT	4	8.7E-1	1.0E0
Annotation Cluster 53	Enrichment Score: 0.12	G	Count	P_Value	Benjamini
INTERPRO	CDP-alcohol phosphatidyltransferase	RT	3	6.2E-1	1.0E0
GOTERM_BP_FAT	phospholipid biosynthetic process	RT	5	6.5E-1	1.0E0
GOTERM_MF_FAT	phosphotransferase activity, for other substituted phosphate groups	RT	3	7.8E-1	1.0E0
GOTERM_BP_FAT	phospholipid metabolic process	RT	7	7.9E-1	1.0E0
GOTERM_BP_FAT	organophosphate metabolic process	RT	7	8.6E-1	1.0E0
GOTERM_BP_FAT	lipid biosynthetic process	RT	7	9.1E-1	1.0E0
Annotation Cluster 54	Enrichment Score: 0.12	G	Count	P_Value	Benjamini
INTERPRO	Pyridoxal phosphate-dependent transferase, major region, subdomain 1	RT	6	5.5E-1	1.0E0
INTERPRO	Aminotransferase, class V/Cysteine desulfurase	RT	3	6.2E-1	1.0E0
GOTERM_MF_FAT	vitamin binding	RT	7	6.8E-1	1.0E0
GOTERM_MF_FAT	pyridoxal phosphate binding	RT	6	7.8E-1	1.0E0
GOTERM_MF_FAT	vitamin B6 binding	RT	6	7.8E-1	1.0E0
GOTERM_MF_FAT	transferase activity, transferring nitrogenous groups	RT	4	8.7E-1	1.0E0
SP_PIR_KEYWORDS	Aminotransferase	RT	3	8.9E-1	9.9E-1
GOTERM_MF_FAT	transaminase activity	RT	3	8.9E-1	1.0E0
INTERPRO	Aminotransferase, class I and II	RT	3	9.0E-1	1.0E0
Annotation Cluster 55	Enrichment Score: 0.1	G	Count	P_Value	Benjamini
GOTERM_BP_FAT	secretion by cell	RT	3	7.9E-1	1.0E0
GOTERM_BP_FAT	vesicle docking	RT	3	7.9E-1	1.0E0
GOTERM_BP_FAT	membrane docking	RT	3	7.9E-1	1.0E0
GOTERM_BP_FAT	vesicle docking during exocytosis	RT	3	7.9E-1	1.0E0
GOTERM_BP_FAT	secretion	RT	3	7.9E-1	1.0E0
GOTERM_BP_FAT	exocytosis	RT	3	7.9E-1	1.0E0

Category	Term		Count	P_Value	Benjamini
INTERPRO	Sec1-like protein	RT	3	8.0E-1	1.0E0
Annotation Cluster 56	Enrichment Score: 0.09		Count	P_Value	Benjamini
KEGG_PATHWAY	Ribosome	RT	38	6.4E-1	1.0E0
GOTERM_BP_FAT	translation	RT	62	7.6E-1	1.0E0
SP_PIR_KEYWORDS	ribosomal protein	RT	39	8.0E-1	9.9E-1
GOTERM_CC_FAT	ribonucleoprotein complex	RT	54	8.1E-1	1.0E0
GOTERM_MF_FAT	structural constituent of ribosome	RT	37	8.2E-1	1.0E0
GOTERM_CC_FAT	ribosome	RT	40	8.6E-1	1.0E0
GOTERM_MF_FAT	structural molecule activity	RT	44	8.6E-1	1.0E0
GOTERM_CC_FAT	intracellular non-membrane-bounded organelle	RT	85	9.1E-1	1.0E0
GOTERM_CC_FAT	non-membrane-bounded organelle	RT	85	9.1E-1	1.0E0
Annotation Cluster 57	Enrichment Score: 0.08		Count	P_Value	Benjamini
SP_PIR_KEYWORDS	Endonuclease	RT	17	7.2E-1	9.9E-1
GOTERM_MF_FAT	endonuclease activity	RT	19	7.4E-1	1.0E0
SP_PIR_KEYWORDS	rna-directed dna polymerase	RT	13	7.5E-1	9.9E-1
GOTERM_MF_FAT	RNA-directed DNA polymerase activity	RT	13	8.1E-1	1.0E0
GOTERM_MF_FAT	nuclease activity	RT	25	9.2E-1	1.0E0
GOTERM_MF_FAT	nucleotidyltransferase activity	RT	36	9.4E-1	1.0E0
GOTERM_MF_FAT	DNA polymerase activity	RT	15	9.8E-1	1.0E0
Annotation Cluster 58	Enrichment Score: 0.07		Count	P_Value	Benjamini
GOTERM_BP_FAT	transmembrane transport	RT	14	4.1E-1	1.0E0
GOTERM_CC_FAT	proton-transporting two-sector ATPase complex, proton-transporting domain	RT	5	4.8E-1	1.0E0
GOTERM_CC_FAT	proton-transporting V-type ATPase, V0 domain	RT	4	6.2E-1	1.0E0
GOTERM_BP_FAT	monovalent inorganic cation transport	RT	10	6.3E-1	1.0E0
GOTERM_CC_FAT	proton-transporting V-type ATPase complex	RT	6	6.6E-1	1.0E0
KEGG_PATHWAY	Oxidative phosphorylation	RT	8	6.6E-1	9.9E-1
GOTERM_BP_FAT	ion transport	RT	14	6.9E-1	1.0E0
GOTERM_BP_FAT	cation transport	RT	14	6.9E-1	1.0E0
GOTERM_CC_FAT	proton-transporting two-sector ATPase complex	RT	9	7.1E-1	1.0E0
GOTERM_BP_FAT	generation of precursor metabolites and energy	RT	15	7.1E-1	1.0E0
GOTERM_MF_FAT	magnesium ion binding	RT	9	7.1E-1	1.0E0
GOTERM_BP_FAT	hydrogen transport	RT	9	7.1E-1	1.0E0
GOTERM_BP_FAT	proton transport	RT	9	7.1E-1	1.0E0
GOTERM_BP_FAT	nucleobase, nucleoside and nucleotide biosynthetic process	RT	23	7.8E-1	1.0E0
GOTERM_BP_FAT	nucleobase, nucleoside, nucleotide and nucleic acid biosynthetic process	RT	23	7.8E-1	1.0E0
GOTERM_BP_FAT	phospholipid transport	RT	3	7.9E-1	1.0E0
GOTERM_BP_FAT	ion transmembrane transport	RT	8	7.9E-1	1.0E0
INTERPRO	ATPase, P-type, phospholipid-translocating, flippase	RT	3	8.0E-1	1.0E0
GOTERM_BP_FAT	nucleotide biosynthetic process	RT	21	8.2E-1	1.0E0
GOTERM_BP_FAT	purine nucleotide metabolic process	RT	16	8.4E-1	1.0E0
GOTERM_MF_FAT	hydrogen ion transmembrane transporter activity	RT	8	8.6E-1	1.0E0
GOTERM_MF_FAT	monovalent inorganic cation transmembrane transporter activity	RT	8	8.6E-1	1.0E0
GOTERM_BP_FAT	oxidative phosphorylation	RT	7	8.6E-1	1.0E0
GOTERM_BP_FAT	ATP synthesis coupled proton transport	RT	7	8.6E-1	1.0E0
GOTERM_BP_FAT	energy coupled proton transport, down electrochemical gradient	RT	7	8.6E-1	1.0E0
GOTERM_BP_FAT	purine nucleotide biosynthetic process	RT	15	8.8E-1	1.0E0
SP_PIR_KEYWORDS	ion transport	RT	3	8.9E-1	9.9E-1
GOTERM_MF_FAT	phospholipid-translocating ATPase activity	RT	3	8.9E-1	1.0E0
GOTERM_MF_FAT	aminophospholipid transporter activity	RT	3	8.9E-1	1.0E0
GOTERM_MF_FAT	phospholipid transporter activity	RT	3	8.9E-1	1.0E0
GOTERM_BP_FAT	lipid transport	RT	3	8.9E-1	1.0E0
GOTERM_BP_FAT	lipid localization	RT	3	8.9E-1	1.0E0
GOTERM_MF_FAT	inorganic cation transmembrane transporter activity	RT	9	9.0E-1	1.0E0
GOTERM_BP_FAT	nitrogen compound biosynthetic process	RT	25	9.1E-1	1.0E0
GOTERM_BP_FAT	purine ribonucleotide metabolic process	RT	14	9.1E-1	1.0E0

	GOTERM_BP_FAT	purine ribonucleotide biosynthetic process	RT			14	9.1E-1	1.0E0
	GOTERM_BP_FAT	ribonucleotide metabolic process	RT			14	9.1E-1	1.0E0
	GOTERM_BP_FAT	ribonucleotide biosynthetic process	RT			14	9.1E-1	1.0E0
	GOTERM_BP_FAT	purine nucleoside triphosphate metabolic process	RT			13	9.4E-1	1.0E0
	GOTERM_BP_FAT	purine nucleoside triphosphate biosynthetic process	RT			13	9.4E-1	1.0E0
	GOTERM_BP_FAT	nucleoside triphosphate metabolic process	RT			13	9.4E-1	1.0E0
	GOTERM_BP_FAT	nucleoside triphosphate biosynthetic process	RT			13	9.4E-1	1.0E0
	GOTERM_BP_FAT	purine ribonucleoside triphosphate metabolic process	RT			13	9.4E-1	1.0E0
	GOTERM_BP_FAT	purine ribonucleoside triphosphate biosynthetic process	RT			13	9.4E-1	1.0E0
	GOTERM_BP_FAT	ribonucleoside triphosphate metabolic process	RT			13	9.4E-1	1.0E0
	GOTERM_BP_FAT	ribonucleoside triphosphate biosynthetic process	RT			13	9.4E-1	1.0E0
	GOTERM_BP_FAT	ATP metabolic process	RT			12	9.4E-1	1.0E0
	GOTERM_BP_FAT	ATP biosynthetic process	RT			12	9.4E-1	1.0E0
	GOTERM_MF_FAT	lipid transporter activity	RT			3	9.4E-1	1.0E0
	GOTERM_MF_FAT	ATPase activity, coupled to transmembrane movement of ions, phosphorylative mechanism	RT			5	9.7E-1	1.0E0
	INTERPRO	ATPase, P-type, K/Mg/Cd/Cu/Zn/Na/Ca/Na/H-transporter	RT			5	9.8E-1	1.0E0
	GOTERM_CC_FAT	proton-transporting two-sector ATPase complex, catalytic domain	RT			3	9.9E-1	1.0E0
	INTERPRO	ATPase, P-type phosphorylation site	RT			3	9.9E-1	1.0E0
	INTERPRO	ATPase, P-type, ATPase-associated region	RT			4	9.9E-1	1.0E0
	GOTERM_MF_FAT	ATPase activity, coupled to transmembrane movement of ions	RT			7	1.0E0	1.0E0
	GOTERM_MF_FAT	cation-transporting ATPase activity	RT			3	1.0E0	1.0E0
	GOTERM_MF_FAT	hydrolase activity, acting on acid anhydrides, catalyzing transmembrane movement of substances	RT			11	1.0E0	1.0E0
	GOTERM_MF_FAT	primary active transmembrane transporter activity	RT			10	1.0E0	1.0E0
	GOTERM_MF_FAT	P-P-bond-hydrolysis-driven transmembrane transporter activity	RT			10	1.0E0	1.0E0
	GOTERM_MF_FAT	ATPase activity, coupled to movement of substances	RT			9	1.0E0	1.0E0
	GOTERM_MF_FAT	ATPase activity, coupled to transmembrane movement of substances	RT			9	1.0E0	1.0E0
	Annotation Cluster 59	Enrichment Score: 0.07	G			Count	P_Value	Benjamini
	COG_ONTOLOGY	Lipid metabolism	RT			8	7.5E-1	1.0E0
	INTERPRO	Phospholipid/glycerol acyltransferase	RT			3	9.0E-1	1.0E0
	SMART	PlsC	RT			3	9.0E-1	1.0E0
	Annotation Cluster 60	Enrichment Score: 0.06	G			Count	P_Value	Benjamini
	SP_PIR_KEYWORDS	protein biosynthesis	RT			7	5.5E-1	1.0E0
	GOTERM_BP_FAT	ncRNA metabolic process	RT			29	7.3E-1	1.0E0
	GOTERM_BP_FAT	tRNA metabolic process	RT			21	8.6E-1	1.0E0
	SP_PIR_KEYWORDS	Aminoacyl-tRNA synthetase	RT			12	8.6E-1	9.9E-1
	INTERPRO	Aminoacyl-tRNA synthetase, class I, conserved site	RT			5	8.8E-1	1.0E0
	GOTERM_MF_FAT	ligase activity, forming carbon-oxygen bonds	RT			12	9.0E-1	1.0E0
	GOTERM_MF_FAT	aminoacyl-tRNA ligase activity	RT			12	9.0E-1	1.0E0
	GOTERM_MF_FAT	ligase activity, forming aminoacyl-tRNA and related compounds	RT			12	9.0E-1	1.0E0
	KEGG_PATHWAY	Aminoacyl-tRNA biosynthesis	RT			10	9.3E-1	1.0E0
	GOTERM_BP_FAT	tRNA aminoacylation	RT			11	9.4E-1	1.0E0
	GOTERM_BP_FAT	amino acid activation	RT			11	9.4E-1	1.0E0
	GOTERM_BP_FAT	tRNA aminoacylation for protein translation	RT			10	9.6E-1	1.0E0
	INTERPRO	Aminoacyl-tRNA synthetase, class II, conserved region	RT			4	9.7E-1	1.0E0
	Annotation Cluster 61	Enrichment Score: 0.06	G			Count	P_Value	Benjamini
	SP_PIR_KEYWORDS	Protease	RT			20	7.5E-1	9.9E-1
	SP_PIR_KEYWORDS	thiol protease	RT			6	7.8E-1	9.9E-1
	GOTERM_MF_FAT	peptidase activity	RT			42	8.5E-1	1.0E0
	INTERPRO	Peptidase C1A, papain C-terminal	RT			14	9.0E-1	1.0E0
	INTERPRO	Peptidase C1A, papain	RT			14	9.0E-1	1.0E0
	SMART	Pept_C1	RT			14	9.0E-1	1.0E0
	GOTERM_MF_FAT	peptidase activity, acting on L-amino acid peptides	RT			35	9.0E-1	1.0E0

GOTERM_MF_FAT	cysteine-type endopeptidase activity	RT		12	9.0E-1	1.0E0
GOTERM_MF_FAT	cysteine-type peptidase activity	RT		15	9.1E-1	1.0E0
GOTERM_MF_FAT	endopeptidase activity	RT		23	9.2E-1	1.0E0
INTERPRO	Peptidase, cysteine peptidase active site	RT		15	9.3E-1	1.0E0
Annotation Cluster 62	Enrichment Score: 0.06	G		Count	P_Value	Benjamini
GOTERM_BP_FAT	negative regulation of gene expression	RT		4	8.7E-1	1.0E0
GOTERM_BP_FAT	negative regulation of macromolecule metabolic process	RT		4	8.7E-1	1.0E0
GOTERM_BP_FAT	gene silencing	RT		4	8.7E-1	1.0E0
Annotation Cluster 63	Enrichment Score: 0.06	G		Count	P_Value	Benjamini
GOTERM_BP_FAT	cellular carbohydrate biosynthetic process	RT		4	8.7E-1	1.0E0
GOTERM_BP_FAT	carbohydrate biosynthetic process	RT		4	8.7E-1	1.0E0
GOTERM_BP_FAT	alcohol biosynthetic process	RT		3	8.9E-1	1.0E0
Annotation Cluster 64	Enrichment Score: 0.06	G		Count	P_Value	Benjamini
INTERPRO	Thioredoxin, conserved site	RT		4	8.0E-1	1.0E0
INTERPRO	Thioredoxin-like	RT		7	8.8E-1	1.0E0
INTERPRO	Thioredoxin-like subdomain	RT		3	9.0E-1	1.0E0
INTERPRO	Thioredoxin domain	RT		3	9.5E-1	1.0E0
Annotation Cluster 65	Enrichment Score: 0.06	G		Count	P_Value	Benjamini
GOTERM_CC_FAT	cytoskeleton	RT		29	8.2E-1	1.0E0
GOTERM_BP_FAT	microtubule-based process	RT		29	8.3E-1	1.0E0
INTERPRO	Kinesin, motor region, conserved site	RT		10	8.3E-1	1.0E0
INTERPRO	Kinesin, motor region	RT		15	8.6E-1	1.0E0
SMART	KISc	RT		15	8.6E-1	1.0E0
GOTERM_CC_FAT	microtubule	RT		17	8.8E-1	1.0E0
GOTERM_CC_FAT	microtubule cytoskeleton	RT		27	8.9E-1	1.0E0
GOTERM_CC_FAT	cytoskeletal part	RT		27	8.9E-1	1.0E0
SP_PIR_KEYWORDS	motor protein	RT		12	9.0E-1	9.9E-1
SP_PIR_KEYWORDS	microtubule	RT		16	9.1E-1	9.9E-1
GOTERM_MF_FAT	motor activity	RT		21	9.2E-1	1.0E0
GOTERM_MF_FAT	microtubule motor activity	RT		21	9.2E-1	1.0E0
GOTERM_BP_FAT	microtubule-based movement	RT		23	9.5E-1	1.0E0
Annotation Cluster 66	Enrichment Score: 0.05	G		Count	P_Value	Benjamini
GOTERM_CC_FAT	intracellular organelle lumen	RT		3	8.9E-1	1.0E0
GOTERM_CC_FAT	membrane-enclosed lumen	RT		3	8.9E-1	1.0E0
GOTERM_CC_FAT	nuclear lumen	RT		3	8.9E-1	1.0E0
GOTERM_CC_FAT	organelle lumen	RT		3	8.9E-1	1.0E0
Annotation Cluster 67	Enrichment Score: 0.05	G		Count	P_Value	Benjamini
GOTERM_MF_FAT	structure-specific DNA binding	RT		3	8.9E-1	1.0E0
GOTERM_MF_FAT	double-stranded DNA binding	RT		3	8.9E-1	1.0E0
GOTERM_MF_FAT	mismatched DNA binding	RT		3	8.9E-1	1.0E0
GOTERM_BP_FAT	mismatch repair	RT		3	8.9E-1	1.0E0
Annotation Cluster 68	Enrichment Score: 0.05	G		Count	P_Value	Benjamini
INTERPRO	WD40 repeat	RT		41	8.1E-1	1.0E0
SMART	WD40	RT		41	8.1E-1	1.0E0
INTERPRO	WD40/YVTN repeat-like	RT		39	8.6E-1	1.0E0
INTERPRO	WD40 repeat, conserved site	RT		15	9.0E-1	1.0E0
INTERPRO	WD40 repeat, subgroup	RT		29	9.2E-1	1.0E0
SP_PIR_KEYWORDS	wd repeat	RT		24	9.4E-1	1.0E0
INTERPRO	WD40 repeat 2	RT		21	9.6E-1	1.0E0
INTERPRO	WD40 repeat, region	RT		23	9.6E-1	1.0E0
Annotation Cluster 69	Enrichment Score: 0.05	G		Count	P_Value	Benjamini
INTERPRO	ABC transporter, conserved site	RT		12	8.4E-1	1.0E0
COG_ONTOLOGY	Defense mechanisms	RT		12	8.7E-1	1.0E0
INTERPRO	ABC transporter-like	RT		10	9.8E-1	1.0E0
Annotation Cluster 70	Enrichment Score: 0.05	G		Count	P_Value	Benjamini
GOTERM_MF_FAT	antioxidant activity	RT		4	8.7E-1	1.0E0
INTERPRO	Thioredoxin-like	RT		7	8.8E-1	1.0E0
INTERPRO	Alkyl hydroperoxide reductase/ Thiol specific antioxidant/ Mal allergen	RT		3	9.5E-1	1.0E0
Annotation Cluster 71	Enrichment Score: 0.05	G		Count	P_Value	Benjamini

Category	Term	RT	Count	P_Value	Benjamini
GOTERM_BP_FAT	cell cycle	RT	5	7.8E-1	1.0E0
GOTERM_BP_FAT	M phase	RT	3	9.4E-1	1.0E0
GOTERM_BP_FAT	cell cycle phase	RT	3	9.4E-1	1.0E0
GOTERM_BP_FAT	cell cycle process	RT	3	9.4E-1	1.0E0
Annotation Cluster 72	Enrichment Score: 0.04		Count	P_Value	Benjamini
GOTERM_BP_FAT	phospholipid metabolic process	RT	7	7.9E-1	1.0E0
GOTERM_BP_FAT	organophosphate metabolic process	RT	7	8.6E-1	1.0E0
GOTERM_BP_FAT	glycerophospholipid metabolic process	RT	3	9.7E-1	1.0E0
GOTERM_BP_FAT	phosphoinositide metabolic process	RT	3	9.7E-1	1.0E0
GOTERM_BP_FAT	glycerolipid metabolic process	RT	3	9.7E-1	1.0E0
Annotation Cluster 73	Enrichment Score: 0.04		Count	P_Value	Benjamini
GOTERM_BP_FAT	small GTPase mediated signal transduction	RT	9	8.0E-1	1.0E0
INTERPRO	ADP-ribosylation factor	RT	3	8.0E-1	1.0E0
SMART	ARF	RT	3	8.0E-1	1.0E0
GOTERM_BP_FAT	intracellular signaling cascade	RT	11	8.7E-1	1.0E0
INTERPRO	ARF/SAR superfamily	RT	4	8.9E-1	1.0E0
PIR_SUPERFAMILY	PIRSF001711:ADP-ribosylation factor	RT	3	8.9E-1	1.0E0
INTERPRO	Ras GTPase	RT	6	9.6E-1	1.0E0
GOTERM_MF_FAT	guanyl ribonucleotide binding	RT	22	9.8E-1	1.0E0
GOTERM_MF_FAT	GTP binding	RT	22	9.8E-1	1.0E0
GOTERM_MF_FAT	guanyl nucleotide binding	RT	22	9.8E-1	1.0E0
INTERPRO	Ras	RT	5	9.8E-1	1.0E0
SP_PIR_KEYWORDS	gtp-binding	RT	17	9.9E-1	1.0E0
INTERPRO	Small GTP-binding protein	RT	8	9.9E-1	1.0E0
Annotation Cluster 74	Enrichment Score: 0.03		Count	P_Value	Benjamini
PIR_SUPERFAMILY	PIRSF002306:tubulin	RT	4	8.8E-1	1.0E0
INTERPRO	Tubulin/FtsZ, 2-layer sandwich domain	RT	4	8.9E-1	1.0E0
GOTERM_BP_FAT	protein polymerization	RT	4	9.3E-1	1.0E0
INTERPRO	Tubulin	RT	4	9.4E-1	1.0E0
INTERPRO	Tubulin/FtsZ, GTPase domain	RT	4	9.4E-1	1.0E0
INTERPRO	Tubulin, conserved site	RT	4	9.4E-1	1.0E0
GOTERM_BP_FAT	protein complex assembly	RT	6	9.5E-1	1.0E0
GOTERM_BP_FAT	protein complex biogenesis	RT	6	9.5E-1	1.0E0
GOTERM_BP_FAT	cellular protein complex assembly	RT	5	9.6E-1	1.0E0
GOTERM_MF_FAT	GTPase activity	RT	7	9.8E-1	1.0E0
Annotation Cluster 75	Enrichment Score: 0.02		Count	P_Value	Benjamini
INTERPRO	26S proteasome subunit P45	RT	3	9.0E-1	1.0E0
INTERPRO	ATPase, AAA-type, conserved site	RT	6	9.8E-1	1.0E0
INTERPRO	ATPase, AAA-type, core	RT	9	1.0E0	1.0E0
Annotation Cluster 76	Enrichment Score: 0.01		Count	P_Value	Benjamini
INTERPRO	Chaperonin TCP-1, conserved site	RT	4	9.7E-1	1.0E0
INTERPRO	Chaperonin Cpn60/TCP-1	RT	4	9.7E-1	1.0E0
SP_PIR_KEYWORDS	Chaperone	RT	5	9.7E-1	1.0E0
GOTERM_MF_FAT	unfolded protein binding	RT	9	9.8E-1	1.0E0
PIR_SUPERFAMILY	PIRSF002584:molecular chaperone t-complex-type	RT	3	9.9E-1	1.0E0
SP_PIR_KEYWORDS	cytoplasm	RT	7	9.9E-1	1.0E0
INTERPRO	Chaperone, tailless complex polypeptide 1	RT	3	9.9E-1	1.0E0
Annotation Cluster 77	Enrichment Score: 0.01		Count	P_Value	Benjamini
GOTERM_CC_FAT	dynein complex	RT	5	9.7E-1	1.0E0
INTERPRO	Dynein heavy chain	RT	5	9.8E-1	1.0E0
GOTERM_CC_FAT	microtubule associated complex	RT	6	9.8E-1	1.0E0
Annotation Cluster 78	Enrichment Score: 0.01		Count	P_Value	Benjamini
INTERPRO	Molecular chaperone, heat shock protein, Hsp40, DnaJ	RT	3	9.8E-1	1.0E0
GOTERM_MF_FAT	heat shock protein binding	RT	3	9.9E-1	1.0E0
INTERPRO	Heat shock protein DnaJ, N-terminal	RT	3	9.9E-1	1.0E0
SMART	DnaJ	RT	3	9.9E-1	1.0E0
Annotation Cluster 79	Enrichment Score: 0.01		Count	P_Value	Benjamini
SP_PIR_KEYWORDS	Rotamase	RT	3	9.9E-1	1.0E0

DAVID Bioinformatics Resources 6.7
National Institute of Allergy and Infectious Diseases (NIAID), NIH

Functional Annotation Clustering

Help and Manual

Current Gene List: 3_Random5
Current Background: Giardia intestinalis
3000 DAVID IDs

Options | Classification Stringency: Medium

Rerun using options | Create Sublist

84 Cluster(s)

Download File

Annotation Cluster 1	Enrichment Score: 0.8		Count	P_Value	Benjamini
INTERPRO	ATPase, AAA+ type, core	RT	48	1.0E-1	1.0E0
SMART	AAA	RT	48	1.1E-1	1.0E0
INTERPRO	ATPase, AAA-type, core	RT	17	3.5E-1	1.0E0
Annotation Cluster 2	Enrichment Score: 0.67		Count	P_Value	Benjamini
GOTERM_MF_FAT	endonuclease activity	RT	26	5.1E-2	1.0E0
GOTERM_MF_FAT	nuclease activity	RT	34	1.8E-1	1.0E0
GOTERM_MF_FAT	nucleotidyltransferase activity	RT	47	2.1E-1	1.0E0
SP_PIR_KEYWORDS	Endonuclease	RT	21	2.6E-1	1.0E0
GOTERM_MF_FAT	RNA-directed DNA polymerase activity	RT	17	2.8E-1	1.0E0
SP_PIR_KEYWORDS	rna-directed dna polymerase	RT	16	3.6E-1	1.0E0
GOTERM_MF_FAT	DNA polymerase activity	RT	22	4.2E-1	1.0E0
Annotation Cluster 3	Enrichment Score: 0.62		Count	P_Value	Benjamini
INTERPRO	Annexin	RT	13	1.3E-1	1.0E0
SP_PIR_KEYWORDS	annexin	RT	13	1.5E-1	1.0E0
GOTERM_MF_FAT	calcium ion binding	RT	30	1.5E-1	1.0E0
GOTERM_MF_FAT	lipid binding	RT	25	2.1E-1	1.0E0
INTERPRO	Annexin repeat	RT	11	2.3E-1	1.0E0
SMART	ANX	RT	11	2.4E-1	1.0E0
GOTERM_MF_FAT	phospholipid binding	RT	19	2.6E-1	1.0E0
INTERPRO	Alpha giardin	RT	9	3.9E-1	1.0E0
GOTERM_MF_FAT	calcium-dependent phospholipid binding	RT	15	4.0E-1	1.0E0
GOTERM_BP_FAT	cytoskeleton organization	RT	11	4.6E-1	1.0E0
Annotation Cluster 4	Enrichment Score: 0.57		Count	P_Value	Benjamini
INTERPRO	Chaperonin TCP-1, conserved site	RT	8	2.0E-1	1.0E0
INTERPRO	Chaperonin Cpn60/TCP-1	RT	8	2.0E-1	1.0E0
SP_PIR_KEYWORDS	Chaperone	RT	10	2.1E-1	1.0E0
INTERPRO	Chaperone, tailless complex polypeptide 1	RT	7	2.8E-1	1.0E0
GOTERM_MF_FAT	unfolded protein binding	RT	15	3.0E-1	1.0E0
PIR_SUPERFAMILY	PIRSF002584:molecular chaperone t-complex-type	RT	7	3.2E-1	1.0E0
GOTERM_BP_FAT	protein folding	RT	20	3.4E-1	1.0E0
SP_PIR_KEYWORDS	cytoplasm	RT	13	3.5E-1	1.0E0
Annotation Cluster 5	Enrichment Score: 0.55		Count	P_Value	Benjamini
INTERPRO	WD40 repeat, conserved site	RT	21	1.6E-1	1.0E0
INTERPRO	WD40 repeat, subgroup	RT	37	2.1E-1	1.0E0
INTERPRO	WD40 repeat 2	RT	29	2.2E-1	1.0E0
INTERPRO	WD40 repeat	RT	47	2.8E-1	1.0E0
SMART	WD40	RT	47	3.1E-1	1.0E0
INTERPRO	WD40/YVTN repeat-like	RT	45	3.5E-1	1.0E0
INTERPRO	WD40 repeat, region	RT	30	3.7E-1	1.0E0
SP_PIR_KEYWORDS	wd repeat	RT	31	4.4E-1	1.0E0
Annotation Cluster 6	Enrichment Score: 0.51		Count	P_Value	Benjamini
GOTERM_MF_FAT	protein serine/threonine kinase activity	RT	69	8.7E-2	1.0E0
INTERPRO	Serine/threonine protein kinase, active site	RT	58	1.5E-1	1.0E0
INTERPRO	Serine/threonine protein kinase	RT	36	4.2E-1	1.0E0
SMART	S_TKc	RT	36	4.5E-1	1.0E0

Category	Term		Count	P_Value	Benjamini
SP_PIR_KEYWORDS	serine/threonine-protein kinase	RT	38	4.6E-1	1.0E0
INTERPRO	Protein kinase, ATP binding site	RT	46	7.7E-1	1.0E0
Annotation Cluster 7	Enrichment Score: 0.5		Count	P_Value	Benjamini
GOTERM_BP_FAT	negative regulation of macromolecule metabolic process	RT	7	1.3E-1	1.0E0
GOTERM_BP_FAT	negative regulation of gene expression	RT	7	1.3E-1	1.0E0
GOTERM_BP_FAT	gene silencing	RT	7	1.3E-1	1.0E0
GOTERM_BP_FAT	protein amino acid deacetylation	RT	6	2.0E-1	1.0E0
GOTERM_BP_FAT	negative regulation of RNA metabolic process	RT	5	3.0E-1	1.0E0
GOTERM_BP_FAT	chromatin silencing	RT	5	3.0E-1	1.0E0
GOTERM_BP_FAT	negative regulation of cellular biosynthetic process	RT	5	3.0E-1	1.0E0
GOTERM_BP_FAT	negative regulation of nitrogen compound metabolic process	RT	5	3.0E-1	1.0E0
GOTERM_BP_FAT	negative regulation of biosynthetic process	RT	5	3.0E-1	1.0E0
GOTERM_BP_FAT	regulation of gene expression, epigenetic	RT	5	3.0E-1	1.0E0
GOTERM_BP_FAT	negative regulation of macromolecule biosynthetic process	RT	5	3.0E-1	1.0E0
GOTERM_BP_FAT	negative regulation of nucleobase, nucleoside, nucleotide and nucleic acid metabolic process	RT	5	3.0E-1	1.0E0
GOTERM_BP_FAT	negative regulation of transcription	RT	5	3.0E-1	1.0E0
GOTERM_BP_FAT	negative regulation of gene expression, epigenetic	RT	5	3.0E-1	1.0E0
GOTERM_BP_FAT	negative regulation of transcription, DNA-dependent	RT	5	3.0E-1	1.0E0
INTERPRO	NAD-dependent histone deacetylase, silent information regulator Sir2	RT	5	3.1E-1	1.0E0
GOTERM_MF_FAT	NAD binding	RT	5	3.1E-1	1.0E0
GOTERM_BP_FAT	regulation of transcription	RT	20	4.2E-1	1.0E0
GOTERM_MF_FAT	hydrolase activity, acting on carbon-nitrogen (but not peptide) bonds, in linear amides	RT	6	5.6E-1	1.0E0
GOTERM_MF_FAT	NAD or NADH binding	RT	6	5.6E-1	1.0E0
GOTERM_BP_FAT	regulation of transcription, DNA-dependent	RT	8	6.1E-1	1.0E0
GOTERM_BP_FAT	regulation of RNA metabolic process	RT	8	6.1E-1	1.0E0
GOTERM_MF_FAT	coenzyme binding	RT	13	9.0E-1	1.0E0
Annotation Cluster 8	Enrichment Score: 0.45		Count	P_Value	Benjamini
GOTERM_BP_FAT	cellular response to stress	RT	15	2.8E-1	1.0E0
GOTERM_BP_FAT	response to DNA damage stimulus	RT	14	3.4E-1	1.0E0
GOTERM_BP_FAT	DNA repair	RT	14	3.4E-1	1.0E0
GOTERM_BP_FAT	DNA metabolic process	RT	36	4.7E-1	1.0E0
Annotation Cluster 9	Enrichment Score: 0.44		Count	P_Value	Benjamini
SP_PIR_KEYWORDS	ubl conjugation pathway	RT	11	1.5E-1	1.0E0
GOTERM_BP_FAT	modification-dependent macromolecule catabolic process	RT	19	1.6E-1	1.0E0
GOTERM_BP_FAT	modification-dependent protein catabolic process	RT	19	1.6E-1	1.0E0
GOTERM_BP_FAT	proteolysis involved in cellular protein catabolic process	RT	23	2.0E-1	1.0E0
GOTERM_BP_FAT	cellular protein catabolic process	RT	23	2.0E-1	1.0E0
GOTERM_BP_FAT	protein catabolic process	RT	26	2.0E-1	1.0E0
GOTERM_BP_FAT	cellular macromolecule catabolic process	RT	23	2.6E-1	1.0E0
GOTERM_BP_FAT	macromolecule catabolic process	RT	26	2.7E-1	1.0E0
GOTERM_BP_FAT	proteolysis	RT	55	2.9E-1	1.0E0
KEGG_PATHWAY	Ubiquitin mediated proteolysis	RT	8	5.0E-1	9.8E-1
PIR_SUPERFAMILY	PIRSF001567:ubiquitin-protein ligase E2	RT	5	5.5E-1	1.0E0
INTERPRO	Ubiquitin-conjugating enzyme, E2	RT	8	6.2E-1	1.0E0
INTERPRO	Ubiquitin-conjugating enzyme/RWD-like	RT	8	6.2E-1	1.0E0
SMART	UBCc	RT	8	6.3E-1	1.0E0
GOTERM_BP_FAT	ubiquitin-dependent protein catabolic process	RT	9	6.3E-1	1.0E0
GOTERM_MF_FAT	acid-amino acid ligase activity	RT	16	7.0E-1	1.0E0
GOTERM_MF_FAT	small conjugating protein ligase activity	RT	8	7.4E-1	1.0E0
GOTERM_MF_FAT	ligase activity, forming carbon-nitrogen bonds	RT	17	8.8E-1	1.0E0
Annotation Cluster 10	Enrichment Score: 0.43		Count	P_Value	Benjamini
INTERPRO	Ankyrin	RT	260	2.6E-1	1.0E0
SMART	ANK	RT	260	3.3E-1	1.0E0

SP_PIR_KEYWORDS	ank repeat	RT	180	6.0E-1	1.0E0
Annotation Cluster 11	Enrichment Score: 0.4		Count	P_Value	Benjamini
KEGG_PATHWAY	Nucleotide excision repair	RT	12	2.9E-1	1.0E0
KEGG_PATHWAY	Mismatch repair	RT	10	4.5E-1	1.0E0
KEGG_PATHWAY	DNA replication	RT	14	4.8E-1	9.9E-1
Annotation Cluster 12	Enrichment Score: 0.39		Count	P_Value	Benjamini
GOTERM_MF_FAT	small GTPase regulator activity	RT	9	2.6E-1	1.0E0
GOTERM_MF_FAT	GTPase regulator activity	RT	9	2.6E-1	1.0E0
GOTERM_BP_FAT	regulation of GTPase activity	RT	7	2.7E-1	1.0E0
GOTERM_BP_FAT	regulation of hydrolase activity	RT	7	2.7E-1	1.0E0
GOTERM_MF_FAT	nucleoside-triphosphatase regulator activity	RT	10	3.1E-1	1.0E0
GOTERM_BP_FAT	regulation of Ras GTPase activity	RT	6	3.8E-1	1.0E0
GOTERM_MF_FAT	GTPase activator activity	RT	6	3.9E-1	1.0E0
GOTERM_BP_FAT	regulation of Ras protein signal transduction	RT	7	4.3E-1	1.0E0
GOTERM_BP_FAT	regulation of small GTPase mediated signal transduction	RT	7	4.3E-1	1.0E0
GOTERM_BP_FAT	regulation of Rab GTPase activity	RT	4	4.4E-1	1.0E0
GOTERM_BP_FAT	regulation of Rab protein signal transduction	RT	4	4.4E-1	1.0E0
GOTERM_MF_FAT	enzyme activator activity	RT	7	4.5E-1	1.0E0
GOTERM_MF_FAT	Rab GTPase activator activity	RT	4	4.5E-1	1.0E0
GOTERM_MF_FAT	Ras GTPase activator activity	RT	4	4.5E-1	1.0E0
INTERPRO	RabGAP/TBC	RT	3	6.2E-1	1.0E0
SMART	TBC	RT	3	6.3E-1	1.0E0
GOTERM_BP_FAT	regulation of ARF protein signal transduction	RT	3	9.0E-1	1.0E0
Annotation Cluster 13	Enrichment Score: 0.37		Count	P_Value	Benjamini
GOTERM_MF_FAT	endonuclease activity, active with either ribo- or deoxyribonucleic acids and producing 5'-phosphomonoesters	RT	7	1.4E-1	1.0E0
GOTERM_MF_FAT	endoribonuclease activity, producing 5'-phosphomonoesters	RT	4	4.5E-1	1.0E0
GOTERM_MF_FAT	endoribonuclease activity	RT	4	6.6E-1	1.0E0
GOTERM_MF_FAT	ribonuclease activity	RT	6	8.1E-1	1.0E0
Annotation Cluster 14	Enrichment Score: 0.35		Count	P_Value	Benjamini
INTERPRO	Alcohol dehydrogenase, iron-type	RT	5	3.1E-1	1.0E0
COG_ONTOLOGY	Energy production and conversion	RT	7	4.6E-1	1.0E0
PIR_SUPERFAMILY	PIRSF000113:iron-containing alcohol dehydrogenase	RT	3	6.5E-1	1.0E0
Annotation Cluster 15	Enrichment Score: 0.32		Count	P_Value	Benjamini
GOTERM_MF_FAT	vitamin binding	RT	9	2.6E-1	1.0E0
GOTERM_MF_FAT	vitamin B6 binding	RT	8	3.5E-1	1.0E0
GOTERM_MF_FAT	pyridoxal phosphate binding	RT	8	3.5E-1	1.0E0
GOTERM_MF_FAT	transferase activity, transferring nitrogenous groups	RT	6	3.9E-1	1.0E0
INTERPRO	Pyridoxal phosphate-dependent transferase, major region, subdomain 1	RT	6	5.5E-1	1.0E0
GOTERM_MF_FAT	cofactor binding	RT	21	6.4E-1	1.0E0
INTERPRO	Aminotransferase, class I and II	RT	4	6.5E-1	1.0E0
GOTERM_MF_FAT	transaminase activity	RT	4	6.6E-1	1.0E0
SP_PIR_KEYWORDS	Aminotransferase	RT	4	6.7E-1	1.0E0
Annotation Cluster 16	Enrichment Score: 0.31		Count	P_Value	Benjamini
GOTERM_MF_FAT	RNA polymerase activity	RT	17	2.8E-1	1.0E0
GOTERM_MF_FAT	DNA-directed RNA polymerase activity	RT	16	3.4E-1	1.0E0
KEGG_PATHWAY	RNA polymerase	RT	13	3.4E-1	1.0E0
GOTERM_BP_FAT	transcription	RT	17	4.3E-1	1.0E0
KEGG_PATHWAY	Purine metabolism	RT	20	5.6E-1	9.8E-1
SP_PIR_KEYWORDS	nucleotidyltransferase	RT	15	7.0E-1	9.9E-1
KEGG_PATHWAY	Pyrimidine metabolism	RT	19	7.7E-1	9.9E-1
SP_PIR_KEYWORDS	dna-directed rna polymerase	RT	8	8.3E-1	9.9E-1
Annotation Cluster 17	Enrichment Score: 0.29		Count	P_Value	Benjamini
INTERPRO	Small GTP-binding protein	RT	15	2.0E-1	1.0E0
INTERPRO	Ras	RT	9	3.9E-1	1.0E0
GOTERM_BP_FAT	intracellular signaling cascade	RT	14	4.5E-1	1.0E0
GOTERM_MF_FAT	guanyl nucleotide binding	RT	30	4.6E-1	1.0E0
GOTERM_MF_FAT	GTP binding	RT	30	4.6E-1	1.0E0

GOTERM_MF_FAT	guanyl ribonucleotide binding	RT	30	4.6E-1	1.0E0
SP_PIR_KEYWORDS	gtp-binding	RT	25	5.2E-1	1.0E0
GOTERM_BP_FAT	small GTPase mediated signal transduction	RT	10	6.6E-1	1.0E0
INTERPRO	Ras GTPase	RT	8	7.3E-1	1.0E0
GOTERM_MF_FAT	GTPase activity	RT	10	7.7E-1	1.0E0
PIR_SUPERFAMILY	PIRSF001710:Ras-related protein Rab	RT	3	9.6E-1	1.0E0
Annotation Cluster 18	Enrichment Score: 0.28		Count	P_Value	Benjamini
GOTERM_MF_FAT	thiolester hydrolase activity	RT	7	2.9E-1	1.0E0
INTERPRO	Peptidase C19, ubiquitin carboxyl-terminal hydrolase 2	RT	5	5.1E-1	1.0E0
GOTERM_MF_FAT	ubiquitin thiolesterase activity	RT	5	5.2E-1	1.0E0
GOTERM_BP_FAT	ubiquitin-dependent protein catabolic process	RT	9	6.3E-1	1.0E0
INTERPRO	Peptidase C19, ubiquitin carboxyl-terminal hydrolase 2, conserved site	RT	3	8.0E-1	1.0E0
Annotation Cluster 19	Enrichment Score: 0.28		Count	P_Value	Benjamini
GOTERM_MF_FAT	endonuclease activity, active with either ribo- or deoxyribonucleic acids and producing 5'-phosphomonoesters	RT	7	1.4E-1	1.0E0
INTERPRO	Deoxyribonuclease, TatD Mg-dependent	RT	3	6.2E-1	1.0E0
INTERPRO	Deoxyribonuclease, TatD-related	RT	3	6.2E-1	1.0E0
INTERPRO	Deoxyribonuclease, TatD	RT	3	6.2E-1	1.0E0
GOTERM_MF_FAT	deoxyribonuclease activity	RT	3	6.3E-1	1.0E0
GOTERM_MF_FAT	endodeoxyribonuclease activity, producing 5'-phosphomonoesters	RT	3	6.3E-1	1.0E0
GOTERM_MF_FAT	endodeoxyribonuclease activity	RT	3	6.3E-1	1.0E0
PIR_SUPERFAMILY	PIRSF005902:DNase_TatD	RT	3	6.5E-1	1.0E0
Annotation Cluster 20	Enrichment Score: 0.27		Count	P_Value	Benjamini
GOTERM_MF_FAT	protein serine/threonine kinase activity	RT	69	8.7E-2	1.0E0
INTERPRO	Serine/threonine protein kinase, active site	RT	58	1.5E-1	1.0E0
INTERPRO	Serine/threonine protein kinase-related	RT	137	4.7E-1	1.0E0
GOTERM_BP_FAT	phosphorus metabolic process	RT	177	4.7E-1	1.0E0
GOTERM_BP_FAT	phosphate metabolic process	RT	177	4.7E-1	1.0E0
SP_PIR_KEYWORDS	nucleotide-binding	RT	161	4.8E-1	1.0E0
GOTERM_BP_FAT	phosphorylation	RT	167	5.1E-1	1.0E0
SP_PIR_KEYWORDS	atp-binding	RT	152	5.5E-1	1.0E0
GOTERM_BP_FAT	protein amino acid phosphorylation	RT	155	5.7E-1	1.0E0
INTERPRO	Protein kinase, core	RT	153	6.3E-1	1.0E0
GOTERM_MF_FAT	purine ribonucleotide binding	RT	332	6.5E-1	1.0E0
GOTERM_MF_FAT	ribonucleotide binding	RT	332	6.5E-1	1.0E0
GOTERM_MF_FAT	protein kinase activity	RT	156	6.7E-1	1.0E0
GOTERM_MF_FAT	purine nucleotide binding	RT	335	7.0E-1	1.0E0
SP_PIR_KEYWORDS	kinase	RT	191	7.2E-1	9.9E-1
GOTERM_MF_FAT	ATP binding	RT	302	7.2E-1	1.0E0
GOTERM_MF_FAT	adenyl ribonucleotide binding	RT	302	7.2E-1	1.0E0
GOTERM_MF_FAT	nucleotide binding	RT	354	7.6E-1	1.0E0
GOTERM_MF_FAT	purine nucleoside binding	RT	305	7.6E-1	1.0E0
GOTERM_MF_FAT	adenyl nucleotide binding	RT	305	7.6E-1	1.0E0
GOTERM_MF_FAT	nucleoside binding	RT	306	8.1E-1	1.0E0
Annotation Cluster 21	Enrichment Score: 0.26		Count	P_Value	Benjamini
INTERPRO	Ribosomal protein L7A/RS6 family	RT	4	4.4E-1	1.0E0
INTERPRO	Ribosomal protein L7Ae/L30e/S12e/Gadd45	RT	5	5.1E-1	1.0E0
INTERPRO	High mobility group-like nuclear protein	RT	3	6.2E-1	1.0E0
PIR_SUPERFAMILY	PIRSF002165:ribosomal protein, L7Ab/L7Aa/L7Ae/L8e types/H/ACA ribonucleoprotein complex, NHP2 subunit	RT	3	6.5E-1	1.0E0
Annotation Cluster 22	Enrichment Score: 0.24		Count	P_Value	Benjamini
INTERPRO	Dual specificity phosphatase, catalytic domain	RT	5	3.1E-1	1.0E0
INTERPRO	Protein-tyrosine phosphatase, dual specificity	RT	5	3.1E-1	1.0E0
GOTERM_MF_FAT	protein tyrosine/serine/threonine phosphatase activity	RT	5	3.1E-1	1.0E0
GOTERM_BP_FAT	protein amino acid dephosphorylation	RT	9	5.1E-1	1.0E0
GOTERM_BP_FAT	dephosphorylation	RT	10	6.6E-1	1.0E0

GOTERM_MF_FAT	protein tyrosine phosphatase activity	RT	4	8.9E-1	1.0E0
INTERPRO	Dual-specific/protein-tyrosine phosphatase, conserved region	RT	3	9.0E-1	1.0E0
INTERPRO	Protein-tyrosine phosphatase	RT	3	9.0E-1	1.0E0
INTERPRO	Protein-tyrosine phosphatase, active site	RT	3	9.5E-1	1.0E0
Annotation Cluster 23	Enrichment Score: 0.24		Count	P_Value	Benjamini
GOTERM_MF_FAT	transcription regulator activity	RT	8	4.9E-1	1.0E0
INTERPRO	Zinc finger, TFIIS-type	RT	3	6.2E-1	1.0E0
SMART	ZnF_C2C2	RT	3	6.3E-1	1.0E0
Annotation Cluster 24	Enrichment Score: 0.24		Count	P_Value	Benjamini
COG_ONTOLOGY	Defense mechanisms	RT	15	4.2E-1	1.0E0
INTERPRO	ABC transporter-like	RT	14	6.5E-1	1.0E0
INTERPRO	ABC transporter, conserved site	RT	13	7.2E-1	1.0E0
Annotation Cluster 25	Enrichment Score: 0.24		Count	P_Value	Benjamini
SP_PIR_KEYWORDS	Acyltransferase	RT	9	2.8E-1	1.0E0
GOTERM_MF_FAT	acetyltransferase activity	RT	7	6.0E-1	1.0E0
GOTERM_MF_FAT	N-acyltransferase activity	RT	7	6.0E-1	1.0E0
INTERPRO	GCN5-related N-acetyltransferase	RT	6	6.9E-1	1.0E0
GOTERM_MF_FAT	N-acetyltransferase activity	RT	6	7.0E-1	1.0E0
INTERPRO	Acyl-CoA N-acyltransferase	RT	6	8.0E-1	1.0E0
Annotation Cluster 26	Enrichment Score: 0.23		Count	P_Value	Benjamini
GOTERM_CC_FAT	pore complex	RT	4	5.7E-1	1.0E0
GOTERM_CC_FAT	nuclear pore	RT	4	5.7E-1	1.0E0
GOTERM_CC_FAT	nuclear envelope	RT	4	5.7E-1	1.0E0
GOTERM_CC_FAT	envelope	RT	4	5.7E-1	1.0E0
GOTERM_CC_FAT	organelle envelope	RT	4	5.7E-1	1.0E0
GOTERM_CC_FAT	endomembrane system	RT	9	7.1E-1	1.0E0
Annotation Cluster 27	Enrichment Score: 0.23		Count	P_Value	Benjamini
GOTERM_CC_FAT	ribosome	RT	44	2.8E-1	1.0E0
GOTERM_CC_FAT	ribonucleoprotein complex	RT	55	4.7E-1	1.0E0
GOTERM_BP_FAT	translation	RT	66	5.3E-1	1.0E0
KEGG_PATHWAY	Ribosome	RT	40	6.7E-1	9.8E-1
GOTERM_MF_FAT	structural constituent of ribosome	RT	40	6.9E-1	1.0E0
GOTERM_CC_FAT	non-membrane-bounded organelle	RT	84	7.0E-1	1.0E0
GOTERM_CC_FAT	intracellular non-membrane-bounded organelle	RT	84	7.0E-1	1.0E0
SP_PIR_KEYWORDS	ribosomal protein	RT	42	7.0E-1	9.9E-1
GOTERM_MF_FAT	structural molecule activity	RT	46	8.3E-1	1.0E0
Annotation Cluster 28	Enrichment Score: 0.22		Count	P_Value	Benjamini
GOTERM_BP_FAT	coenzyme metabolic process	RT	8	3.3E-1	1.0E0
GOTERM_BP_FAT	coenzyme biosynthetic process	RT	6	3.8E-1	1.0E0
GOTERM_BP_FAT	cofactor metabolic process	RT	9	3.8E-1	1.0E0
GOTERM_BP_FAT	cofactor biosynthetic process	RT	7	4.3E-1	1.0E0
GOTERM_BP_FAT	pyridine nucleotide metabolic process	RT	5	5.0E-1	1.0E0
GOTERM_BP_FAT	oxidoreduction coenzyme metabolic process	RT	5	5.0E-1	1.0E0
GOTERM_BP_FAT	cellular amide metabolic process	RT	4	6.5E-1	1.0E0
GOTERM_BP_FAT	nicotinamide metabolic process	RT	4	6.5E-1	1.0E0
GOTERM_BP_FAT	alkaloid metabolic process	RT	4	6.5E-1	1.0E0
GOTERM_BP_FAT	secondary metabolic process	RT	4	6.5E-1	1.0E0
GOTERM_BP_FAT	nicotinamide nucleotide metabolic process	RT	4	6.5E-1	1.0E0
GOTERM_BP_FAT	pyridine nucleotide biosynthetic process	RT	3	8.0E-1	1.0E0
GOTERM_BP_FAT	water-soluble vitamin metabolic process	RT	3	9.0E-1	1.0E0
GOTERM_BP_FAT	water-soluble vitamin biosynthetic process	RT	3	9.0E-1	1.0E0
GOTERM_BP_FAT	vitamin metabolic process	RT	3	9.0E-1	1.0E0
GOTERM_BP_FAT	vitamin biosynthetic process	RT	3	9.0E-1	1.0E0
Annotation Cluster 29	Enrichment Score: 0.22		Count	P_Value	Benjamini
INTERPRO	Translation elongation factor EFTu/EF1A, domain 2	RT	5	3.1E-1	1.0E0
INTERPRO	Protein synthesis factor, GTP-binding	RT	6	5.5E-1	1.0E0
GOTERM_BP_FAT	translational elongation	RT	5	6.7E-1	1.0E0
SP_PIR_KEYWORDS	elongation factor	RT	5	6.9E-1	1.0E0

	GOTERM_MF_FAT	GTPase activity	RT			10	7.7E-1	1.0E0
	GOTERM_MF_FAT	translation elongation factor activity	RT			5	8.0E-1	1.0E0
	Annotation Cluster 30	Enrichment Score: 0.2	G			Count	P_Value	Benjamini
	GOTERM_MF_FAT	translation factor activity, nucleic acid binding	RT			21	4.0E-1	1.0E0
	GOTERM_MF_FAT	translation initiation factor activity	RT			15	6.8E-1	1.0E0
	GOTERM_BP_FAT	translational initiation	RT			7	7.1E-1	1.0E0
	SP_PIR_KEYWORDS	Initiation factor	RT			11	7.9E-1	9.9E-1
	Annotation Cluster 31	Enrichment Score: 0.2	G			Count	P_Value	Benjamini
	INTERPRO	Lecithin	RT			3	6.2E-1	1.0E0
	GOTERM_MF_FAT	phosphatidylcholine-sterol O-acyltransferase activity	RT			3	6.3E-1	1.0E0
	GOTERM_MF_FAT	O-acyltransferase activity	RT			3	6.3E-1	1.0E0
	Annotation Cluster 32	Enrichment Score: 0.2	G			Count	P_Value	Benjamini
	INTERPRO	Cyclin-related	RT			5	5.1E-1	1.0E0
	SP_PIR_KEYWORDS	cyclin	RT			7	6.1E-1	9.9E-1
	INTERPRO	Cyclin, N-terminal	RT			4	7.9E-1	1.0E0
	Annotation Cluster 33	Enrichment Score: 0.2	G			Count	P_Value	Benjamini
	GOTERM_MF_FAT	phosphotransferase activity, for other substituted phosphate groups	RT			4	4.5E-1	1.0E0
	GOTERM_BP_FAT	organophosphate metabolic process	RT			9	5.1E-1	1.0E0
	GOTERM_BP_FAT	phospholipid metabolic process	RT			8	6.1E-1	1.0E0
	INTERPRO	CDP-alcohol phosphatidyltransferase	RT			3	6.2E-1	1.0E0
	GOTERM_BP_FAT	lipid biosynthetic process	RT			8	8.1E-1	1.0E0
	GOTERM_BP_FAT	phospholipid biosynthetic process	RT			4	8.8E-1	1.0E0
	Annotation Cluster 34	Enrichment Score: 0.19	G			Count	P_Value	Benjamini
	INTERPRO	Thioredoxin, conserved site	RT			6	2.1E-1	1.0E0
	INTERPRO	Thioredoxin-like	RT			9	5.2E-1	1.0E0
	INTERPRO	Thioredoxin-like subdomain	RT			4	6.5E-1	1.0E0
	GOTERM_MF_FAT	antioxidant activity	RT			5	6.8E-1	1.0E0
	INTERPRO	Thioredoxin fold	RT			13	7.2E-1	1.0E0
	INTERPRO	Thioredoxin domain	RT			4	7.9E-1	1.0E0
	INTERPRO	Alkyl hydroperoxide reductase/ Thiol specific antioxidant/ Mal allergen	RT			4	7.9E-1	1.0E0
	GOTERM_BP_FAT	cell redox homeostasis	RT			10	8.2E-1	1.0E0
	GOTERM_BP_FAT	homeostatic process	RT			11	8.3E-1	1.0E0
	GOTERM_BP_FAT	cellular homeostasis	RT			10	8.8E-1	1.0E0
	Annotation Cluster 35	Enrichment Score: 0.19	G			Count	P_Value	Benjamini
	KEGG_PATHWAY	Terpenoid backbone biosynthesis	RT			5	5.2E-1	9.8E-1
	GOTERM_BP_FAT	isoprenoid biosynthetic process	RT			4	6.5E-1	1.0E0
	GOTERM_BP_FAT	isoprenoid metabolic process	RT			4	6.5E-1	1.0E0
	GOTERM_BP_FAT	lipid biosynthetic process	RT			8	8.1E-1	1.0E0
	Annotation Cluster 36	Enrichment Score: 0.18	G			Count	P_Value	Benjamini
	INTERPRO	Phox-like	RT			4	6.5E-1	1.0E0
	GOTERM_MF_FAT	phosphoinositide binding	RT			4	6.6E-1	1.0E0
	SMART	PX	RT			4	6.6E-1	1.0E0
	Annotation Cluster 37	Enrichment Score: 0.18	G			Count	P_Value	Benjamini
	GOTERM_BP_FAT	intracellular transport	RT			18	4.6E-1	1.0E0
	INTERPRO	Clathrin adaptor, sigma subunit/coatomer, zeta subunit	RT			3	6.2E-1	1.0E0
	GOTERM_BP_FAT	cellular protein localization	RT			14	6.4E-1	1.0E0
	GOTERM_BP_FAT	intracellular protein transport	RT			14	6.4E-1	1.0E0
	GOTERM_BP_FAT	cellular macromolecule localization	RT			14	6.4E-1	1.0E0
	GOTERM_BP_FAT	protein localization	RT			20	6.7E-1	1.0E0
	GOTERM_CC_FAT	membrane coat	RT			8	7.1E-1	1.0E0
	GOTERM_CC_FAT	coated membrane	RT			8	7.1E-1	1.0E0
	GOTERM_BP_FAT	protein transport	RT			19	7.2E-1	1.0E0
	GOTERM_BP_FAT	establishment of protein localization	RT			19	7.2E-1	1.0E0
	GOTERM_BP_FAT	vesicle-mediated transport	RT			16	8.1E-1	1.0E0
	Annotation Cluster 38	Enrichment Score: 0.18	G			Count	P_Value	Benjamini
	INTERPRO	EGF-like, laminin	RT			16	6.0E-1	1.0E0
	SMART	EGF_Lam	RT			16	6.2E-1	1.0E0
	INTERPRO	EGF-like, type 3	RT			18	7.8E-1	1.0E0

	Annotation Cluster 39	Enrichment Score: 0.18			Count	P_Value	Benjamini
	GOTERM_MF_FAT	metal ion binding	RT		154	3.0E-1	1.0E0
	GOTERM_MF_FAT	ion binding	RT		154	4.0E-1	1.0E0
	GOTERM_MF_FAT	cation binding	RT		154	4.0E-1	1.0E0
	SP_PIR_KEYWORDS	zinc	RT		49	6.9E-1	1.0E0
	SP_PIR_KEYWORDS	metal-binding	RT		57	7.8E-1	1.0E0
	GOTERM_MF_FAT	transition metal ion binding	RT		106	7.9E-1	1.0E0
	GOTERM_MF_FAT	zinc ion binding	RT		85	8.1E-1	1.0E0
	SP_PIR_KEYWORDS	zinc-finger	RT		34	8.3E-1	9.9E-1
	INTERPRO	Zinc finger, RING-type	RT		35	9.1E-1	1.0E0
	SMART	RING	RT		35	9.3E-1	1.0E0
	INTERPRO	Zinc finger, C3HC4 RING-type	RT		15	9.9E-1	1.0E0

	Annotation Cluster 40	Enrichment Score: 0.17			Count	P_Value	Benjamini
	GOTERM_BP_FAT	lipid transport	RT		5	3.0E-1	1.0E0
	GOTERM_BP_FAT	lipid localization	RT		5	3.0E-1	1.0E0
	GOTERM_CC_FAT	proton-transporting two-sector ATPase complex, proton-transporting domain	RT		5	4.1E-1	1.0E0
	GOTERM_BP_FAT	phospholipid transport	RT		4	4.4E-1	1.0E0
	INTERPRO	ATPase, P-type, phospholipid-translocating, flippase	RT		4	4.4E-1	1.0E0
	GOTERM_MF_FAT	lipid transporter activity	RT		5	5.2E-1	1.0E0
	GOTERM_BP_FAT	nucleoside triphosphate metabolic process	RT		17	5.3E-1	1.0E0
	GOTERM_BP_FAT	purine nucleoside triphosphate metabolic process	RT		17	5.3E-1	1.0E0
	GOTERM_BP_FAT	nucleoside triphosphate biosynthetic process	RT		17	5.3E-1	1.0E0
	GOTERM_BP_FAT	purine ribonucleoside triphosphate metabolic process	RT		17	5.3E-1	1.0E0
	GOTERM_BP_FAT	purine ribonucleoside triphosphate biosynthetic process	RT		17	5.3E-1	1.0E0
	GOTERM_BP_FAT	ribonucleoside triphosphate metabolic process	RT		17	5.3E-1	1.0E0
	GOTERM_BP_FAT	ribonucleoside triphosphate biosynthetic process	RT		17	5.3E-1	1.0E0
	GOTERM_BP_FAT	purine nucleoside triphosphate biosynthetic process	RT		17	5.3E-1	1.0E0
	GOTERM_MF_FAT	magnesium ion binding	RT		10	5.7E-1	1.0E0
	GOTERM_CC_FAT	proton-transporting V-type ATPase, V0 domain	RT		4	5.7E-1	1.0E0
	GOTERM_BP_FAT	ion transport	RT		15	5.7E-1	1.0E0
	GOTERM_BP_FAT	cation transport	RT		15	5.7E-1	1.0E0
	GOTERM_BP_FAT	nucleobase, nucleoside and nucleotide biosynthetic process	RT		25	6.1E-1	1.0E0
	GOTERM_BP_FAT	nucleobase, nucleoside, nucleotide and nucleic acid biosynthetic process	RT		25	6.1E-1	1.0E0
	GOTERM_BP_FAT	purine ribonucleotide biosynthetic process	RT		17	6.1E-1	1.0E0
	GOTERM_BP_FAT	purine ribonucleotide metabolic process	RT		17	6.1E-1	1.0E0
	GOTERM_BP_FAT	ribonucleotide metabolic process	RT		17	6.1E-1	1.0E0
	GOTERM_BP_FAT	ribonucleotide biosynthetic process	RT		17	6.1E-1	1.0E0
	GOTERM_CC_FAT	proton-transporting two-sector ATPase complex	RT		9	6.2E-1	1.0E0
	GOTERM_BP_FAT	transmembrane transport	RT		13	6.2E-1	1.0E0
	GOTERM_BP_FAT	ion transmembrane transport	RT		9	6.3E-1	1.0E0
	GOTERM_BP_FAT	nucleotide biosynthetic process	RT		23	6.4E-1	1.0E0
	GOTERM_BP_FAT	monovalent inorganic cation transport	RT		10	6.6E-1	1.0E0
	GOTERM_MF_FAT	phospholipid-translocating ATPase activity	RT		4	6.6E-1	1.0E0
	GOTERM_MF_FAT	phospholipid transporter activity	RT		4	6.6E-1	1.0E0
	GOTERM_MF_FAT	aminophospholipid transporter activity	RT		4	6.6E-1	1.0E0
	GOTERM_BP_FAT	ATP metabolic process	RT		15	6.6E-1	1.0E0
	GOTERM_BP_FAT	ATP biosynthetic process	RT		15	6.6E-1	1.0E0
	GOTERM_BP_FAT	purine nucleotide biosynthetic process	RT		17	6.9E-1	1.0E0
	INTERPRO	ATPase, P-type, ATPase-associated region	RT		7	7.1E-1	1.0E0
	GOTERM_BP_FAT	oxidative phosphorylation	RT		8	7.2E-1	1.0E0
	GOTERM_BP_FAT	ATP synthesis coupled proton transport	RT		8	7.2E-1	1.0E0
	GOTERM_BP_FAT	energy coupled proton transport, down electrochemical gradient	RT		8	7.2E-1	1.0E0
	GOTERM_BP_FAT	hydrogen transport	RT		9	7.4E-1	1.0E0
	GOTERM_BP_FAT	proton transport	RT		9	7.4E-1	1.0E0

	GOTERM_BP_FAT	generation of precursor metabolites and energy	RT			15	7.4E-1	1.0E0
	GOTERM_MF_FAT	hydrogen ion transmembrane transporter activity	RT			9	7.5E-1	1.0E0
	GOTERM_MF_FAT	monovalent inorganic cation transmembrane transporter activity	RT			9	7.5E-1	1.0E0
	GOTERM_BP_FAT	purine nucleotide metabolic process	RT			17	7.6E-1	1.0E0
	INTERPRO	ATPase, P-type phosphorylation site	RT			5	8.0E-1	1.0E0
	GOTERM_BP_FAT	nitrogen compound biosynthetic process	RT			27	8.0E-1	1.0E0
	INTERPRO	ATPase, P-type, K/Mg/Cd/Cu/Zn/Na/Ca/Na/H-transporter	RT			7	8.1E-1	1.0E0
	GOTERM_CC_FAT	proton-transporting V-type ATPase complex	RT			5	8.2E-1	1.0E0
	GOTERM_MF_FAT	ATPase activity, coupled to transmembrane movement of ions, phosphorylative mechanism	RT			7	8.2E-1	1.0E0
	SP_PIR_KEYWORDS	magnesium	RT			7	8.3E-1	9.9E-1
	GOTERM_MF_FAT	inorganic cation transmembrane transporter activity	RT			10	8.4E-1	1.0E0
	KEGG_PATHWAY	Oxidative phosphorylation	RT			7	8.9E-1	9.9E-1
	GOTERM_CC_FAT	proton-transporting two-sector ATPase complex, catalytic domain	RT			4	9.0E-1	1.0E0
	SP_PIR_KEYWORDS	ion transport	RT			3	9.1E-1	1.0E0
	GOTERM_MF_FAT	ATPase activity, coupled to transmembrane movement of ions	RT			10	9.3E-1	1.0E0
	INTERPRO	Haloacid dehalogenase-like hydrolase	RT			5	9.3E-1	1.0E0
	GOTERM_MF_FAT	ATPase activity, coupled to transmembrane movement of substances	RT			13	9.7E-1	1.0E0
	GOTERM_MF_FAT	ATPase activity, coupled to movement of substances	RT			13	9.7E-1	1.0E0
	GOTERM_MF_FAT	P-P-bond-hydrolysis-driven transmembrane transporter activity	RT			14	9.7E-1	1.0E0
	GOTERM_MF_FAT	primary active transmembrane transporter activity	RT			14	9.7E-1	1.0E0
	GOTERM_MF_FAT	hydrolase activity, acting on acid anhydrides, catalyzing transmembrane movement of substances	RT			14	9.7E-1	1.0E0
	GOTERM_MF_FAT	proton-transporting ATPase activity, rotational mechanism	RT			3	9.8E-1	1.0E0
	GOTERM_MF_FAT	cation-transporting ATPase activity	RT			3	1.0E0	1.0E0
	Annotation Cluster 41	Enrichment Score: 0.17	G			Count	P_Value	Benjamini
	INTERPRO	Giardia variant-specific surface protein	RT			140	4.3E-1	1.0E0
	INTERPRO	EGF-like	RT			109	6.8E-1	1.0E0
	SMART	EGF	RT			109	7.4E-1	1.0E0
	INTERPRO	Furin-like repeat	RT			103	8.0E-1	1.0E0
	SMART	FU	RT			103	8.5E-1	1.0E0
	Annotation Cluster 42	Enrichment Score: 0.16	G			Count	P_Value	Benjamini
	INTERPRO	Uncharacterised conserved protein UCP036436, nucleotide-sugar transporter-related	RT			3	6.2E-1	1.0E0
	PIR_SUPERFAMILY	PIRSF036436:UCP036436	RT			3	6.5E-1	1.0E0
	INTERPRO	Protein of unknown function DUF6, transmembrane	RT			3	8.0E-1	1.0E0
	Annotation Cluster 43	Enrichment Score: 0.16	G			Count	P_Value	Benjamini
	GOTERM_MF_FAT	phosphoprotein phosphatase activity	RT			18	4.9E-1	1.0E0
	INTERPRO	Serine/threonine-specific protein phosphatase and bis(5-nucleosyl)-tetraphosphatase	RT			10	5.5E-1	1.0E0
	SMART	PP2Ac	RT			10	5.7E-1	1.0E0
	INTERPRO	Metallophosphoesterase	RT			13	6.3E-1	1.0E0
	GOTERM_MF_FAT	phosphatase activity	RT			25	7.6E-1	1.0E0
	GOTERM_MF_FAT	iron ion binding	RT			17	8.4E-1	1.0E0
	SP_PIR_KEYWORDS	iron	RT			11	8.6E-1	9.9E-1
	PIR_SUPERFAMILY	PIRSF000908:serine/threonine-protein phosphatase, conventional type	RT			4	9.5E-1	1.0E0
	Annotation Cluster 44	Enrichment Score: 0.16	G			Count	P_Value	Benjamini
	GOTERM_CC_FAT	proteasome complex	RT			14	2.9E-1	1.0E0
	GOTERM_CC_FAT	cytosol	RT			18	3.6E-1	1.0E0
	SP_PIR_KEYWORDS	proteasome	RT			13	5.7E-1	1.0E0
	KEGG_PATHWAY	Proteasome	RT			17	6.6E-1	9.9E-1
	GOTERM_CC_FAT	proteasome core complex	RT			8	7.1E-1	1.0E0
	INTERPRO	Proteasome, beta-type subunit, conserved site	RT			3	8.0E-1	1.0E0
	INTERPRO	Proteasome, subunit alpha/beta	RT			8	8.1E-1	1.0E0
	GOTERM_MF_FAT	threonine-type endopeptidase activity	RT			8	8.2E-1	1.0E0

GOTERM_MF_FAT	threonine-type peptidase activity	RT		8	8.2E-1	1.0E0
INTERPRO	Proteasome, alpha and beta subunits	RT		7	8.8E-1	1.0E0
INTERPRO	Proteasome, alpha-subunit, conserved site	RT		4	8.9E-1	1.0E0
SP_PIR_KEYWORDS	threonine protease	RT		3	9.6E-1	1.0E0
PIR_SUPERFAMILY	PIRSF001212:multicatalytic endopeptidase complex chain C9	RT		3	9.6E-1	1.0E0
Annotation Cluster 45	Enrichment Score: 0.16	G		Count	P_Value	Benjamini
GOTERM_BP_FAT	proteolysis	RT		55	2.9E-1	1.0E0
GOTERM_MF_FAT	peptidase activity, acting on L-amino acid peptides	RT		41	5.4E-1	1.0E0
GOTERM_MF_FAT	peptidase activity	RT		47	5.8E-1	1.0E0
GOTERM_MF_FAT	endopeptidase activity	RT		27	6.7E-1	1.0E0
GOTERM_MF_FAT	cysteine-type endopeptidase activity	RT		14	7.4E-1	1.0E0
GOTERM_MF_FAT	cysteine-type peptidase activity	RT		17	7.8E-1	1.0E0
INTERPRO	Peptidase C1A, papain	RT		15	8.1E-1	1.0E0
INTERPRO	Peptidase C1A, papain C-terminal	RT		15	8.1E-1	1.0E0
SP_PIR_KEYWORDS	thiol protease	RT		6	8.2E-1	9.9E-1
SMART	Pept_C1	RT		15	8.2E-1	1.0E0
SP_PIR_KEYWORDS	Protease	RT		19	9.0E-1	1.0E0
INTERPRO	Peptidase, cysteine peptidase active site	RT		15	9.3E-1	1.0E0
Annotation Cluster 46	Enrichment Score: 0.16	G		Count	P_Value	Benjamini
INTERPRO	EF-Hand type	RT		12	5.0E-1	1.0E0
INTERPRO	EF-HAND 2	RT		11	5.8E-1	1.0E0
SP_PIR_KEYWORDS	calcium	RT		10	5.8E-1	1.0E0
INTERPRO	EF-HAND 1	RT		12	7.0E-1	1.0E0
INTERPRO	Calcium-binding EF-hand	RT		6	8.8E-1	1.0E0
INTERPRO	EF hand	RT		6	8.8E-1	1.0E0
SMART	EFh	RT		6	8.8E-1	1.0E0
Annotation Cluster 47	Enrichment Score: 0.15	G		Count	P_Value	Benjamini
GOTERM_BP_FAT	hexose catabolic process	RT		9	6.3E-1	1.0E0
GOTERM_BP_FAT	alcohol catabolic process	RT		9	6.3E-1	1.0E0
GOTERM_BP_FAT	glucose catabolic process	RT		9	6.3E-1	1.0E0
GOTERM_BP_FAT	monosaccharide catabolic process	RT		9	6.3E-1	1.0E0
GOTERM_BP_FAT	cellular carbohydrate catabolic process	RT		9	6.3E-1	1.0E0
GOTERM_BP_FAT	carbohydrate catabolic process	RT		9	6.3E-1	1.0E0
KEGG_PATHWAY	Glycolysis / Gluconeogenesis	RT		5	6.9E-1	9.7E-1
GOTERM_BP_FAT	generation of precursor metabolites and energy	RT		15	7.4E-1	1.0E0
GOTERM_BP_FAT	monosaccharide metabolic process	RT		13	7.8E-1	1.0E0
GOTERM_BP_FAT	hexose metabolic process	RT		11	8.3E-1	1.0E0
GOTERM_BP_FAT	glucose metabolic process	RT		11	8.3E-1	1.0E0
GOTERM_BP_FAT	glycolysis	RT		6	8.7E-1	1.0E0
Annotation Cluster 48	Enrichment Score: 0.14	G		Count	P_Value	Benjamini
INTERPRO	Prefoldin alpha-like	RT		3	6.2E-1	1.0E0
GOTERM_CC_FAT	prefoldin complex	RT		4	7.2E-1	1.0E0
GOTERM_CC_FAT	cytosolic part	RT		5	8.2E-1	1.0E0
Annotation Cluster 49	Enrichment Score: 0.14	G		Count	P_Value	Benjamini
GOTERM_MF_FAT	dipeptidase activity	RT		3	6.3E-1	1.0E0
SP_PIR_KEYWORDS	dipeptidase	RT		3	6.4E-1	1.0E0
GOTERM_MF_FAT	exopeptidase activity	RT		7	7.2E-1	1.0E0
GOTERM_MF_FAT	metallopeptidase activity	RT		8	7.4E-1	1.0E0
GOTERM_MF_FAT	metalloexopeptidase activity	RT		4	8.9E-1	1.0E0
Annotation Cluster 50	Enrichment Score: 0.14	G		Count	P_Value	Benjamini
GOTERM_BP_FAT	cellular amino acid derivative biosynthetic process	RT		3	6.2E-1	1.0E0
KEGG_PATHWAY	Glutathione metabolism	RT		4	6.7E-1	9.8E-1
GOTERM_BP_FAT	cellular amino acid derivative metabolic process	RT		3	9.0E-1	1.0E0
Annotation Cluster 51	Enrichment Score: 0.14	G		Count	P_Value	Benjamini
GOTERM_BP_FAT	organophosphate metabolic process	RT		9	5.1E-1	1.0E0
GOTERM_BP_FAT	phospholipid metabolic process	RT		8	6.1E-1	1.0E0
GOTERM_MF_FAT	inositol or phosphatidylinositol kinase activity	RT		4	6.6E-1	1.0E0

GOTERM_BP_FAT	glycerolipid metabolic process	RT	4	8.8E-1	1.0E0
GOTERM_BP_FAT	phosphoinositide metabolic process	RT	4	8.8E-1	1.0E0
GOTERM_BP_FAT	glycerophospholipid metabolic process	RT	4	8.8E-1	1.0E0
Annotation Cluster 52	Enrichment Score: 0.13		Count	P_Value	Benjamini
INTERPRO	Cyclin-related	RT	5	5.1E-1	1.0E0
INTERPRO	Cyclin	RT	3	9.0E-1	1.0E0
SMART	CYCLIN	RT	3	9.0E-1	1.0E0
Annotation Cluster 53	Enrichment Score: 0.12		Count	P_Value	Benjamini
INTERPRO	Kelch-type beta propeller	RT	5	6.7E-1	1.0E0
INTERPRO	Kelch repeat type 1	RT	4	7.9E-1	1.0E0
SMART	Kelch	RT	4	8.0E-1	1.0E0
Annotation Cluster 54	Enrichment Score: 0.12		Count	P_Value	Benjamini
GOTERM_CC_FAT	flagellum	RT	3	7.4E-1	1.0E0
GOTERM_CC_FAT	cell projection	RT	3	7.4E-1	1.0E0
SP_PIR_KEYWORDS	flagellum	RT	3	8.1E-1	1.0E0
Annotation Cluster 55	Enrichment Score: 0.11		Count	P_Value	Benjamini
INTERPRO	Molecular chaperone, heat shock protein, Hsp40, DnaJ	RT	5	6.7E-1	1.0E0
INTERPRO	Heat shock protein DnaJ, N-terminal	RT	5	8.0E-1	1.0E0
GOTERM_MF_FAT	heat shock protein binding	RT	5	8.0E-1	1.0E0
SMART	DnaJ	RT	5	8.1E-1	1.0E0
Annotation Cluster 56	Enrichment Score: 0.11		Count	P_Value	Benjamini
GOTERM_MF_FAT	ATP-dependent helicase activity	RT	23	6.0E-1	1.0E0
GOTERM_MF_FAT	purine NTP-dependent helicase activity	RT	23	6.0E-1	1.0E0
INTERPRO	DNA/RNA helicase, DEAD/DEAH box type, N-terminal	RT	18	6.4E-1	1.0E0
GOTERM_MF_FAT	ATPase activity	RT	54	7.1E-1	1.0E0
INTERPRO	RNA helicase, DEAD-box type, Q motif	RT	10	7.5E-1	1.0E0
INTERPRO	DEAD-like helicase, N-terminal	RT	24	7.8E-1	1.0E0
SMART	DEXDc	RT	24	8.0E-1	1.0E0
GOTERM_MF_FAT	ATPase activity, coupled	RT	39	8.1E-1	1.0E0
INTERPRO	Helicase, superfamily 1 and 2, ATP-binding	RT	23	8.6E-1	1.0E0
SP_PIR_KEYWORDS	helicase	RT	24	8.6E-1	9.9E-1
INTERPRO	DNA/RNA helicase, C-terminal	RT	23	9.0E-1	1.0E0
SMART	HELICc	RT	23	9.1E-1	1.0E0
GOTERM_MF_FAT	helicase activity	RT	28	9.3E-1	1.0E0
Annotation Cluster 57	Enrichment Score: 0.11		Count	P_Value	Benjamini
GOTERM_BP_FAT	ribonucleoside metabolic process	RT	5	6.7E-1	1.0E0
GOTERM_BP_FAT	purine ribonucleoside metabolic process	RT	3	8.0E-1	1.0E0
GOTERM_BP_FAT	purine nucleoside metabolic process	RT	3	8.0E-1	1.0E0
GOTERM_BP_FAT	nucleoside metabolic process	RT	7	8.7E-1	1.0E0
Annotation Cluster 58	Enrichment Score: 0.11		Count	P_Value	Benjamini
INTERPRO	EGF-like, type 3	RT	18	7.8E-1	1.0E0
SP_PIR_KEYWORDS	egf-like domain	RT	10	7.8E-1	9.9E-1
INTERPRO	EGF, extracellular	RT	13	7.9E-1	1.0E0
Annotation Cluster 59	Enrichment Score: 0.1		Count	P_Value	Benjamini
SP_PIR_KEYWORDS	membrane	RT	16	7.2E-1	9.9E-1
GOTERM_CC_FAT	integral to membrane	RT	34	7.9E-1	1.0E0
GOTERM_CC_FAT	intrinsic to membrane	RT	34	7.9E-1	1.0E0
INTERPRO	Amino acid transporter, transmembrane	RT	6	8.0E-1	1.0E0
SP_PIR_KEYWORDS	transmembrane	RT	16	8.9E-1	1.0E0
Annotation Cluster 60	Enrichment Score: 0.1		Count	P_Value	Benjamini
INTERPRO	Protein phosphatase 2C	RT	4	7.9E-1	1.0E0
INTERPRO	Protein phosphatase 2C, N-terminal	RT	4	7.9E-1	1.0E0
INTERPRO	Protein phosphatase 2C-related	RT	4	7.9E-1	1.0E0
SMART	PP2Cc	RT	4	8.0E-1	1.0E0
SMART	PP2C_SIG	RT	4	8.0E-1	1.0E0
Annotation Cluster 61	Enrichment Score: 0.09		Count	P_Value	Benjamini
INTERPRO	Peptidyl-prolyl cis-trans isomerase, FKBP-type	RT	4	7.9E-1	1.0E0
GOTERM_MF_FAT	cis-trans isomerase activity	RT	5	8.0E-1	1.0E0

GOTERM_MF_FAT	peptidyl-prolyl cis-trans isomerase activity	RT	5	8.0E-1	1.0E0
SP_PIR_KEYWORDS	Rotamase	RT	5	8.1E-1	9.9E-1
Annotation Cluster 62	Enrichment Score: 0.08		Count	P_Value	Benjamini
INTERPRO	Dynein heavy chain, N-terminal region 2	RT	7	7.1E-1	1.0E0
GOTERM_CC_FAT	microtubule associated complex	RT	8	8.0E-1	1.0E0
GOTERM_CC_FAT	dynein complex	RT	6	8.7E-1	1.0E0
INTERPRO	Dynein heavy chain	RT	6	9.3E-1	1.0E0
Annotation Cluster 63	Enrichment Score: 0.08		Count	P_Value	Benjamini
SP_PIR_KEYWORDS	ligase	RT	32	7.2E-1	9.9E-1
GOTERM_MF_FAT	aminoacyl-tRNA ligase activity	RT	14	7.4E-1	1.0E0
GOTERM_MF_FAT	ligase activity, forming carbon-oxygen bonds	RT	14	7.4E-1	1.0E0
GOTERM_MF_FAT	ligase activity, forming aminoacyl-tRNA and related compounds	RT	14	7.4E-1	1.0E0
INTERPRO	Rossmann-like alpha/beta/alpha sandwich fold	RT	11	7.7E-1	1.0E0
GOTERM_BP_FAT	tRNA aminoacylation	RT	13	7.8E-1	1.0E0
GOTERM_BP_FAT	amino acid activation	RT	13	7.8E-1	1.0E0
SP_PIR_KEYWORDS	Aminoacyl-tRNA synthetase	RT	13	8.2E-1	9.9E-1
GOTERM_BP_FAT	tRNA aminoacylation for protein translation	RT	12	8.4E-1	1.0E0
INTERPRO	Aminoacyl-tRNA synthetase, class I, conserved site	RT	5	8.8E-1	1.0E0
GOTERM_BP_FAT	tRNA metabolic process	RT	21	8.8E-1	1.0E0
KEGG_PATHWAY	Aminoacyl-tRNA biosynthesis	RT	11	9.1E-1	9.9E-1
GOTERM_BP_FAT	ncRNA metabolic process	RT	27	9.1E-1	1.0E0
SP_PIR_KEYWORDS	protein biosynthesis	RT	5	9.4E-1	1.0E0
INTERPRO	Aminoacyl-tRNA synthetase, class II, conserved region	RT	4	9.7E-1	1.0E0
Annotation Cluster 64	Enrichment Score: 0.07		Count	P_Value	Benjamini
INTERPRO	Ubiquitin supergroup	RT	7	8.1E-1	1.0E0
INTERPRO	Ubiquitin	RT	4	8.9E-1	1.0E0
SMART	UBQ	RT	4	8.9E-1	1.0E0
Annotation Cluster 65	Enrichment Score: 0.05		Count	P_Value	Benjamini
GOTERM_MF_FAT	antiporter activity	RT	4	8.0E-1	1.0E0
GOTERM_BP_FAT	multidrug transport	RT	3	9.0E-1	1.0E0
GOTERM_BP_FAT	drug transport	RT	3	9.0E-1	1.0E0
GOTERM_BP_FAT	response to drug	RT	3	9.0E-1	1.0E0
INTERPRO	Multi antimicrobial extrusion protein MatE	RT	3	9.0E-1	1.0E0
GOTERM_MF_FAT	drug transporter activity	RT	3	9.0E-1	1.0E0
Annotation Cluster 66	Enrichment Score: 0.05		Count	P_Value	Benjamini
INTERPRO	Like-Sm ribonucleoprotein, core	RT	6	8.8E-1	1.0E0
INTERPRO	Like-Sm ribonucleoprotein, eukaryotic and archaea-type, core	RT	4	8.9E-1	1.0E0
SMART	Sm	RT	4	8.9E-1	1.0E0
SP_PIR_KEYWORDS	viral nucleoprotein	RT	8	8.9E-1	1.0E0
Annotation Cluster 67	Enrichment Score: 0.05		Count	P_Value	Benjamini
GOTERM_BP_FAT	tRNA modification	RT	3	8.0E-1	1.0E0
GOTERM_BP_FAT	RNA modification	RT	8	9.2E-1	1.0E0
GOTERM_BP_FAT	tRNA processing	RT	8	9.5E-1	1.0E0
Annotation Cluster 68	Enrichment Score: 0.05		Count	P_Value	Benjamini
GOTERM_BP_FAT	cellular carbohydrate biosynthetic process	RT	4	8.8E-1	1.0E0
GOTERM_BP_FAT	carbohydrate biosynthetic process	RT	4	8.8E-1	1.0E0
GOTERM_BP_FAT	alcohol biosynthetic process	RT	3	9.0E-1	1.0E0
Annotation Cluster 69	Enrichment Score: 0.05		Count	P_Value	Benjamini
GOTERM_BP_FAT	ribonucleoprotein complex biogenesis	RT	11	8.3E-1	1.0E0
GOTERM_BP_FAT	ribosome biogenesis	RT	11	8.3E-1	1.0E0
GOTERM_BP_FAT	rRNA processing	RT	6	9.6E-1	1.0E0
GOTERM_BP_FAT	rRNA metabolic process	RT	6	9.6E-1	1.0E0
Annotation Cluster 70	Enrichment Score: 0.05		Count	P_Value	Benjamini
INTERPRO	AMP-dependent synthetase and ligase	RT	4	8.9E-1	1.0E0
GOTERM_MF_FAT	fatty-acid ligase activity	RT	3	9.0E-1	1.0E0
GOTERM_MF_FAT	long-chain-fatty-acid-CoA ligase activity	RT	3	9.0E-1	1.0E0
GOTERM_MF_FAT	ligase activity, forming carbon-sulfur bonds	RT	3	9.0E-1	1.0E0
Annotation Cluster 71	Enrichment Score: 0.05		Count	P_Value	Benjamini

GOTERM_BP_FAT	mismatch repair	RT	3	9.0E-1	1.0E0
GOTERM_MF_FAT	double-stranded DNA binding	RT	3	9.0E-1	1.0E0
GOTERM_MF_FAT	mismatched DNA binding	RT	3	9.0E-1	1.0E0
GOTERM_MF_FAT	structure-specific DNA binding	RT	3	9.0E-1	1.0E0
Annotation Cluster 72	Enrichment Score: 0.04		Count	P_Value	Benjamini
GOTERM_BP_FAT	cell cycle	RT	5	7.9E-1	1.0E0
GOTERM_BP_FAT	cell cycle phase	RT	3	9.5E-1	1.0E0
GOTERM_BP_FAT	cell cycle process	RT	3	9.5E-1	1.0E0
GOTERM_BP_FAT	M phase	RT	3	9.5E-1	1.0E0
Annotation Cluster 73	Enrichment Score: 0.04		Count	P_Value	Benjamini
INTERPRO	Tetratricopeptide TPR-1	RT	11	8.3E-1	1.0E0
INTERPRO	Tetratricopeptide-like helical	RT	16	9.0E-1	1.0E0
INTERPRO	Tetratricopeptide repeat	RT	12	9.2E-1	1.0E0
INTERPRO	Tetratricopeptide region	RT	12	9.2E-1	1.0E0
SMART	TPR	RT	12	9.3E-1	1.0E0
SP_PIR_KEYWORDS	tpr repeat	RT	4	9.7E-1	1.0E0
Annotation Cluster 74	Enrichment Score: 0.04		Count	P_Value	Benjamini
GOTERM_CC_FAT	Golgi apparatus	RT	6	8.1E-1	1.0E0
GOTERM_BP_FAT	ER to Golgi vesicle-mediated transport	RT	3	9.5E-1	1.0E0
GOTERM_BP_FAT	Golgi vesicle transport	RT	3	9.5E-1	1.0E0
GOTERM_CC_FAT	Golgi apparatus part	RT	3	9.8E-1	1.0E0
Annotation Cluster 75	Enrichment Score: 0.04		Count	P_Value	Benjamini
INTERPRO	Myb, DNA-binding	RT	3	9.0E-1	1.0E0
INTERPRO	SANT, DNA-binding	RT	5	9.3E-1	1.0E0
SMART	SANT	RT	5	9.4E-1	1.0E0
Annotation Cluster 76	Enrichment Score: 0.03		Count	P_Value	Benjamini
GOTERM_CC_FAT	microtubule cytoskeleton	RT	26	8.5E-1	1.0E0
GOTERM_CC_FAT	cytoskeletal part	RT	26	8.5E-1	1.0E0
GOTERM_CC_FAT	cytoskeleton	RT	27	8.5E-1	1.0E0
GOTERM_CC_FAT	microtubule	RT	16	8.8E-1	1.0E0
INTERPRO	Kinesin, motor region, conserved site	RT	9	9.2E-1	1.0E0
SP_PIR_KEYWORDS	motor protein	RT	12	9.4E-1	1.0E0
GOTERM_MF_FAT	motor activity	RT	21	9.5E-1	1.0E0
GOTERM_MF_FAT	microtubule motor activity	RT	21	9.5E-1	1.0E0
INTERPRO	Kinesin, motor region	RT	13	9.7E-1	1.0E0
SMART	KISc	RT	13	9.7E-1	1.0E0
GOTERM_BP_FAT	microtubule-based process	RT	26	9.7E-1	1.0E0
SP_PIR_KEYWORDS	microtubule	RT	15	9.7E-1	1.0E0
GOTERM_BP_FAT	microtubule-based movement	RT	22	9.8E-1	1.0E0
Annotation Cluster 77	Enrichment Score: 0.03		Count	P_Value	Benjamini
GOTERM_BP_FAT	one-carbon metabolic process	RT	6	8.7E-1	1.0E0
GOTERM_BP_FAT	methylation	RT	5	9.3E-1	1.0E0
SP_PIR_KEYWORDS	methyltransferase	RT	9	9.9E-1	1.0E0
Annotation Cluster 78	Enrichment Score: 0.03		Count	P_Value	Benjamini
INTERPRO	RNA recognition motif, RNP-1	RT	7	9.2E-1	1.0E0
SMART	RRM	RT	7	9.3E-1	1.0E0
INTERPRO	Nucleotide-binding, alpha-beta plait	RT	7	9.7E-1	1.0E0
Annotation Cluster 79	Enrichment Score: 0.02		Count	P_Value	Benjamini
GOTERM_BP_FAT	ncRNA metabolic process	RT	27	9.1E-1	1.0E0
GOTERM_BP_FAT	ncRNA processing	RT	14	9.5E-1	1.0E0
GOTERM_BP_FAT	tRNA processing	RT	8	9.5E-1	1.0E0
GOTERM_BP_FAT	RNA processing	RT	21	9.9E-1	1.0E0
Annotation Cluster 80	Enrichment Score: 0.02		Count	P_Value	Benjamini
GOTERM_CC_FAT	chromosome	RT	13	8.9E-1	1.0E0
GOTERM_CC_FAT	chromosomal part	RT	8	9.1E-1	1.0E0
GOTERM_CC_FAT	protein-DNA complex	RT	6	9.2E-1	1.0E0
INTERPRO	Histone-fold	RT	7	9.2E-1	1.0E0
GOTERM_CC_FAT	nucleosome	RT	5	9.3E-1	1.0E0
GOTERM_BP_FAT	cellular macromolecular complex subunit organization	RT	12	9.5E-1	1.0E0

GOTERM_BP_FAT	macromolecular complex subunit organization	RT	13	9.5E-1	1.0E0
GOTERM_CC_FAT	chromatin	RT	5	9.6E-1	1.0E0
GOTERM_BP_FAT	nucleosome assembly	RT	5	9.6E-1	1.0E0
GOTERM_BP_FAT	DNA packaging	RT	5	9.6E-1	1.0E0
GOTERM_BP_FAT	nucleosome organization	RT	5	9.6E-1	1.0E0
GOTERM_BP_FAT	chromatin assembly	RT	5	9.6E-1	1.0E0
GOTERM_BP_FAT	protein-DNA complex assembly	RT	5	9.6E-1	1.0E0
INTERPRO	Histone core	RT	5	9.6E-1	1.0E0
GOTERM_BP_FAT	macromolecular complex assembly	RT	12	9.7E-1	1.0E0
GOTERM_BP_FAT	cellular macromolecular complex assembly	RT	11	9.7E-1	1.0E0
SP_PIR_KEYWORDS	chromosomal protein	RT	5	9.7E-1	1.0E0
SP_PIR_KEYWORDS	nucleosome core	RT	3	9.8E-1	1.0E0
GOTERM_BP_FAT	chromosome organization	RT	8	9.8E-1	1.0E0
GOTERM_BP_FAT	chromatin organization	RT	6	9.9E-1	1.0E0
GOTERM_BP_FAT	chromatin assembly or disassembly	RT	5	9.9E-1	1.0E0
Annotation Cluster 81	Enrichment Score: 0.02		Count	P_Value	Benjamini
INTERPRO	Zinc finger, C2H2-type	RT	9	9.2E-1	1.0E0
INTERPRO	Zinc finger, C2H2-like	RT	8	9.7E-1	1.0E0
SMART	ZnF_C2H2	RT	8	9.7E-1	1.0E0
Annotation Cluster 82	Enrichment Score: 0.02		Count	P_Value	Benjamini
GOTERM_BP_FAT	RNA modification	RT	8	9.2E-1	1.0E0
GOTERM_MF_FAT	intramolecular transferase activity	RT	5	9.6E-1	1.0E0
GOTERM_BP_FAT	pseudouridine synthesis	RT	3	9.8E-1	1.0E0
GOTERM_MF_FAT	pseudouridine synthase activity	RT	3	9.8E-1	1.0E0
Annotation Cluster 83	Enrichment Score: 0.02		Count	P_Value	Benjamini
GOTERM_MF_FAT	electron carrier activity	RT	12	9.0E-1	1.0E0
INTERPRO	4Fe-4S ferredoxin, iron-sulpur binding domain	RT	4	9.7E-1	1.0E0
INTERPRO	4Fe-4S ferredoxin, iron-sulphur binding, subgroup	RT	4	9.7E-1	1.0E0
INTERPRO	4Fe-4S ferredoxin, iron-sulphur binding, conserved site	RT	3	9.8E-1	1.0E0
GOTERM_MF_FAT	metal cluster binding	RT	8	9.9E-1	1.0E0
GOTERM_MF_FAT	iron-sulfur cluster binding	RT	8	9.9E-1	1.0E0
Annotation Cluster 84	Enrichment Score: 0.01		Count	P_Value	Benjamini
GOTERM_BP_FAT	protein complex assembly	RT	6	9.6E-1	1.0E0
GOTERM_BP_FAT	protein complex biogenesis	RT	6	9.6E-1	1.0E0
GOTERM_BP_FAT	cellular protein complex assembly	RT	5	9.6E-1	1.0E0
INTERPRO	Tubulin/FtsZ, 2-layer sandwich domain	RT	3	9.8E-1	1.0E0
PIR_SUPERFAMILY	PIRSF002306:tubulin	RT	3	9.8E-1	1.0E0
GOTERM_BP_FAT	protein polymerization	RT	3	9.9E-1	1.0E0
INTERPRO	Tubulin, conserved site	RT	3	9.9E-1	1.0E0
INTERPRO	Tubulin	RT	3	9.9E-1	1.0E0
INTERPRO	Tubulin/FtsZ, GTPase domain	RT	3	9.9E-1	1.0E0

186 terms were not clustered.

Please cite *Nature Protocols 2009; 4(1):44 & Genome Biology 2003; 4(5):P3* within any publication that makes use of any methods inspired by **DAVID**.

Term of Service | Contact Us | Site Map

	GOTERM_MF_FAT	cis-trans isomerase activity	RT		3	9.9E-1	1.0E0
	GOTERM_MF_FAT	peptidyl-prolyl cis-trans isomerase activity	RT		3	9.9E-1	1.0E0

194 terms were not clustered.

Please cite *Nature Protocols 2009; 4(1):44 & Genome Biology 2003; 4(5):P3* within any publication that makes use of any methods inspired by **DAVID**.

Term of Service | Contact Us | Site Map

DAVID Bioinformatics Resources 6.7
National Institute of Allergy and Infectious Diseases (NIAID), NIH

Functional Annotation Clustering

Help and Manual

Current Gene List: 3_Random6
Current Background: Giardia intestinalis
3000 DAVID IDs

Options Classification Stringency Medium
Rerun using options Create Sublist

83 Cluster(s)

Download File

				Count	P_Value	Benjamini
Annotation Cluster 1	Enrichment Score: 1.37	G		Count	P_Value	Benjamini
SP_PIR_KEYWORDS	membrane	RT		23	1.2E-2	7.3E-1
GOTERM_CC_FAT	intrinsic to membrane	RT		45	3.2E-2	9.8E-1
GOTERM_CC_FAT	integral to membrane	RT		45	3.2E-2	9.8E-1
SP_PIR_KEYWORDS	transmembrane	RT		24	3.4E-2	8.5E-1
INTERPRO	Amino acid transporter, transmembrane	RT		8	3.5E-1	1.0E0
Annotation Cluster 2	Enrichment Score: 0.97	G		Count	P_Value	Benjamini
GOTERM_MF_FAT	guanyl ribonucleotide binding	RT		36	2.6E-2	1.0E0
GOTERM_MF_FAT	GTP binding	RT		36	2.6E-2	1.0E0
GOTERM_MF_FAT	guanyl nucleotide binding	RT		36	2.6E-2	1.0E0
INTERPRO	Small GTP-binding protein	RT		16	1.0E-1	1.0E0
SP_PIR_KEYWORDS	gtp-binding	RT		29	1.0E-1	9.8E-1
GOTERM_MF_FAT	GTPase activity	RT		12	4.0E-1	1.0E0
GOTERM_BP_FAT	small GTPase mediated signal transduction	RT		11	4.4E-1	1.0E0
GOTERM_BP_FAT	intracellular signaling cascade	RT		13	6.0E-1	1.0E0
Annotation Cluster 3	Enrichment Score: 0.78	G		Count	P_Value	Benjamini
GOTERM_BP_FAT	cellular homeostasis	RT		16	4.2E-2	1.0E0
GOTERM_BP_FAT	cell redox homeostasis	RT		15	6.0E-2	1.0E0
GOTERM_BP_FAT	homeostatic process	RT		16	8.1E-2	1.0E0
INTERPRO	Thioredoxin-like	RT		11	1.5E-1	1.0E0
INTERPRO	Thioredoxin fold	RT		16	2.5E-1	1.0E0
GOTERM_MF_FAT	antioxidant activity	RT		6	3.9E-1	1.0E0
INTERPRO	Thioredoxin, core	RT		6	3.9E-1	1.0E0
INTERPRO	Alkyl hydroperoxide reductase/ Thiol specific antioxidant/ Mal allergen	RT		5	5.2E-1	1.0E0
Annotation Cluster 4	Enrichment Score: 0.77	G		Count	P_Value	Benjamini
INTERPRO	Ankyrin	RT		268	1.1E-1	1.0E0
SMART	ANK	RT		268	2.1E-1	1.0E0
SP_PIR_KEYWORDS	ank repeat	RT		188	2.2E-1	9.9E-1
Annotation Cluster 5	Enrichment Score: 0.76	G		Count	P_Value	Benjamini
GOTERM_BP_FAT	vesicle-mediated transport	RT		24	1.1E-2	9.9E-1
GOTERM_BP_FAT	intracellular transport	RT		21	9.0E-2	1.0E0
GOTERM_BP_FAT	protein transport	RT		23	1.8E-1	1.0E0
GOTERM_BP_FAT	establishment of protein localization	RT		23	1.8E-1	1.0E0
GOTERM_CC_FAT	Golgi apparatus	RT		9	2.0E-1	1.0E0
GOTERM_BP_FAT	protein localization	RT		23	2.4E-1	1.0E0
GOTERM_BP_FAT	cellular protein localization	RT		16	2.9E-1	1.0E0
GOTERM_BP_FAT	cellular macromolecule localization	RT		16	2.9E-1	1.0E0
GOTERM_BP_FAT	intracellular protein transport	RT		16	2.9E-1	1.0E0
GOTERM_CC_FAT	coated membrane	RT		10	3.6E-1	1.0E0
GOTERM_CC_FAT	membrane coat	RT		10	3.6E-1	1.0E0
Annotation Cluster 6	Enrichment Score: 0.67	G		Count	P_Value	Benjamini
INTERPRO	ATPase, AAA-type, core	RT		19	1.2E-1	1.0E0
INTERPRO	ATPase, AAA+ type, core	RT		46	2.5E-1	1.0E0
SMART	AAA	RT		46	3.1E-1	1.0E0
Annotation Cluster 7	Enrichment Score: 0.63	G		Count	P_Value	Benjamini
INTERPRO	Furin-like repeat	RT		118	9.5E-2	1.0E0

SMART	FU	RT	118	1.5E-1	1.0E0
INTERPRO	EGF-like	RT	116	3.3E-1	1.0E0
INTERPRO	Giardia variant-specific surface protein	RT	143	3.5E-1	1.0E0
SMART	EGF	RT	116	4.4E-1	1.0E0
Annotation Cluster 8	Enrichment Score: 0.53		Count	P_Value	Benjamini
GOTERM_MF_FAT	electron carrier activity	RT	19	3.9E-2	1.0E0
GOTERM_MF_FAT	4 iron, 4 sulfur cluster binding	RT	7	2.8E-1	1.0E0
GOTERM_MF_FAT	iron-sulfur cluster binding	RT	14	3.6E-1	1.0E0
GOTERM_MF_FAT	metal cluster binding	RT	14	3.6E-1	1.0E0
INTERPRO	4Fe-4S ferredoxin, iron-sulphur binding, subgroup	RT	7	4.5E-1	1.0E0
INTERPRO	4Fe-4S ferredoxin, iron-sulpur binding domain	RT	7	4.5E-1	1.0E0
INTERPRO	4Fe-4S ferredoxin, iron-sulphur binding, conserved site	RT	5	6.9E-1	1.0E0
Annotation Cluster 9	Enrichment Score: 0.51		Count	P_Value	Benjamini
GOTERM_BP_FAT	translational elongation	RT	7	1.3E-1	1.0E0
INTERPRO	Translation elongation factor EFTu/EF1A, domain 2	RT	5	3.2E-1	1.0E0
SP_PIR_KEYWORDS	elongation factor	RT	6	4.0E-1	9.9E-1
GOTERM_MF_FAT	translation elongation factor activity	RT	6	5.6E-1	1.0E0
Annotation Cluster 10	Enrichment Score: 0.47		Count	P_Value	Benjamini
GOTERM_MF_FAT	transition metal ion binding	RT	116	2.1E-1	1.0E0
SP_PIR_KEYWORDS	zinc-finger	RT	40	2.6E-1	9.8E-1
GOTERM_MF_FAT	zinc ion binding	RT	93	2.9E-1	1.0E0
INTERPRO	Zinc finger, RING-type	RT	43	3.0E-1	1.0E0
SP_PIR_KEYWORDS	zinc	RT	53	3.4E-1	9.9E-1
SMART	RING	RT	43	3.6E-1	1.0E0
INTERPRO	Zinc finger, C3HC4 RING-type	RT	23	3.8E-1	1.0E0
SP_PIR_KEYWORDS	metal-binding	RT	62	3.9E-1	9.9E-1
GOTERM_MF_FAT	ion binding	RT	153	4.3E-1	1.0E0
GOTERM_MF_FAT	cation binding	RT	153	4.3E-1	1.0E0
GOTERM_MF_FAT	metal ion binding	RT	151	4.4E-1	1.0E0
Annotation Cluster 11	Enrichment Score: 0.43		Count	P_Value	Benjamini
INTERPRO	ATPase, P-type, ATPase-associated region	RT	10	1.0E-1	1.0E0
GOTERM_BP_FAT	purine nucleotide metabolic process	RT	22	1.1E-1	1.0E0
GOTERM_BP_FAT	purine nucleotide biosynthetic process	RT	21	1.4E-1	1.0E0
GOTERM_BP_FAT	nucleobase, nucleoside and nucleotide biosynthetic process	RT	29	1.4E-1	1.0E0
GOTERM_BP_FAT	nucleobase, nucleoside, nucleotide and nucleic acid biosynthetic process	RT	29	1.4E-1	1.0E0
GOTERM_BP_FAT	nucleotide biosynthetic process	RT	27	1.5E-1	1.0E0
GOTERM_BP_FAT	purine ribonucleotide metabolic process	RT	20	1.7E-1	1.0E0
GOTERM_BP_FAT	ribonucleotide metabolic process	RT	20	1.7E-1	1.0E0
GOTERM_BP_FAT	ribonucleotide biosynthetic process	RT	20	1.7E-1	1.0E0
GOTERM_BP_FAT	purine ribonucleotide biosynthetic process	RT	20	1.7E-1	1.0E0
GOTERM_BP_FAT	ATP metabolic process	RT	18	1.9E-1	1.0E0
GOTERM_BP_FAT	ATP biosynthetic process	RT	18	1.9E-1	1.0E0
GOTERM_MF_FAT	ATPase activity, coupled to transmembrane movement of ions, phosphorylative mechanism	RT	10	1.9E-1	1.0E0
INTERPRO	ATPase, P-type, K/Mg/Cd/Cu/Zn/Na/Ca/Na/H-transporter	RT	10	2.0E-1	1.0E0
GOTERM_BP_FAT	purine nucleoside triphosphate biosynthetic process	RT	19	2.1E-1	1.0E0
GOTERM_BP_FAT	nucleoside triphosphate biosynthetic process	RT	19	2.1E-1	1.0E0
GOTERM_BP_FAT	purine nucleoside triphosphate metabolic process	RT	19	2.1E-1	1.0E0
GOTERM_BP_FAT	nucleoside triphosphate metabolic process	RT	19	2.1E-1	1.0E0
GOTERM_BP_FAT	purine ribonucleoside triphosphate metabolic process	RT	19	2.1E-1	1.0E0
GOTERM_BP_FAT	purine ribonucleoside triphosphate biosynthetic process	RT	19	2.1E-1	1.0E0
GOTERM_BP_FAT	ribonucleoside triphosphate metabolic process	RT	19	2.1E-1	1.0E0
GOTERM_BP_FAT	ribonucleoside triphosphate biosynthetic process	RT	19	2.1E-1	1.0E0
GOTERM_BP_FAT	nitrogen compound biosynthetic process	RT	32	2.4E-1	1.0E0
INTERPRO	ATPase, P-type phosphorylation site	RT	7	2.9E-1	1.0E0

GOTERM_MF_FAT	ATPase activity, coupled to transmembrane movement of ions	RT	14	3.6E-1	1.0E0
KEGG_PATHWAY	Oxidative phosphorylation	RT	9	4.3E-1	9.9E-1
GOTERM_CC_FAT	proton-transporting two-sector ATPase complex, proton-transporting domain	RT	5	4.6E-1	1.0E0
SP_PIR_KEYWORDS	phosphoprotein	RT	4	4.6E-1	9.8E-1
GOTERM_MF_FAT	ATPase activity, coupled to transmembrane movement of substances	RT	18	4.8E-1	1.0E0
GOTERM_MF_FAT	ATPase activity, coupled to movement of substances	RT	18	4.8E-1	1.0E0
GOTERM_MF_FAT	hydrolase activity, acting on acid anhydrides, catalyzing transmembrane movement of substances	RT	19	5.1E-1	1.0E0
GOTERM_MF_FAT	primary active transmembrane transporter activity	RT	19	5.1E-1	1.0E0
GOTERM_MF_FAT	P-P-bond-hydrolysis-driven transmembrane transporter activity	RT	19	5.1E-1	1.0E0
GOTERM_MF_FAT	inorganic cation transmembrane transporter activity	RT	12	5.1E-1	1.0E0
GOTERM_BP_FAT	transmembrane transport	RT	13	6.0E-1	1.0E0
GOTERM_CC_FAT	proton-transporting V-type ATPase, V0 domain	RT	4	6.1E-1	1.0E0
PIR_SUPERFAMILY	PIRSF001292:vacuolar H(+)-transporting ATPase 16K chain	RT	3	6.2E-1	1.0E0
INTERPRO	ATPase, F0/V0 complex, subunit C	RT	3	6.3E-1	1.0E0
INTERPRO	ATPase, V0 complex, proteolipid subunit C	RT	3	6.3E-1	1.0E0
GOTERM_CC_FAT	proton-transporting two-sector ATPase complex	RT	9	6.8E-1	1.0E0
GOTERM_BP_FAT	oxidative phosphorylation	RT	8	7.1E-1	1.0E0
GOTERM_BP_FAT	ATP synthesis coupled proton transport	RT	8	7.1E-1	1.0E0
GOTERM_BP_FAT	energy coupled proton transport, down electrochemical gradient	RT	8	7.1E-1	1.0E0
GOTERM_BP_FAT	generation of precursor metabolites and energy	RT	15	7.2E-1	1.0E0
GOTERM_BP_FAT	hydrogen transport	RT	9	7.2E-1	1.0E0
GOTERM_BP_FAT	proton transport	RT	9	7.2E-1	1.0E0
GOTERM_MF_FAT	hydrogen ion transmembrane transporter activity	RT	9	7.5E-1	1.0E0
GOTERM_MF_FAT	monovalent inorganic cation transmembrane transporter activity	RT	9	7.5E-1	1.0E0
GOTERM_BP_FAT	ion transmembrane transport	RT	8	8.0E-1	1.0E0
GOTERM_BP_FAT	monovalent inorganic cation transport	RT	9	8.0E-1	1.0E0
GOTERM_BP_FAT	ion transport	RT	13	8.3E-1	1.0E0
GOTERM_BP_FAT	cation transport	RT	13	8.3E-1	1.0E0
GOTERM_CC_FAT	proton-transporting V-type ATPase complex	RT	5	8.5E-1	1.0E0
SP_PIR_KEYWORDS	ion transport	RT	3	9.1E-1	1.0E0
GOTERM_MF_FAT	proton-transporting ATPase activity, rotational mechanism	RT	3	9.8E-1	1.0E0
GOTERM_CC_FAT	proton-transporting two-sector ATPase complex, catalytic domain	RT	3	9.8E-1	1.0E0
Annotation Cluster 12	Enrichment Score: 0.4	G	Count	P_Value	Benjamini
GOTERM_MF_FAT	translation factor activity, nucleic acid binding	RT	22	2.6E-1	1.0E0
SP_PIR_KEYWORDS	Initiation factor	RT	13	4.6E-1	9.8E-1
GOTERM_MF_FAT	translation initiation factor activity	RT	16	5.2E-1	1.0E0
Annotation Cluster 13	Enrichment Score: 0.4	G	Count	P_Value	Benjamini
INTERPRO	Small GTP-binding protein	RT	16	1.0E-1	1.0E0
INTERPRO	Ras	RT	9	4.0E-1	1.0E0
GOTERM_BP_FAT	small GTPase mediated signal transduction	RT	11	4.4E-1	1.0E0
INTERPRO	Ras small GTPase, Rab type	RT	4	4.5E-1	1.0E0
SMART	RAB	RT	4	4.7E-1	1.0E0
PIR_SUPERFAMILY	PIRSF001710:Ras-related protein Rab	RT	5	5.0E-1	1.0E0
INTERPRO	Ras GTPase	RT	9	5.4E-1	1.0E0
GOTERM_BP_FAT	intracellular signaling cascade	RT	13	6.0E-1	1.0E0
Annotation Cluster 14	Enrichment Score: 0.4	G	Count	P_Value	Benjamini
INTERPRO	Thioredoxin-like	RT	11	1.5E-1	1.0E0
INTERPRO	Thioredoxin domain	RT	5	5.2E-1	1.0E0
INTERPRO	Thioredoxin, conserved site	RT	5	5.2E-1	1.0E0
INTERPRO	Thioredoxin-like subdomain	RT	4	6.6E-1	1.0E0
Annotation Cluster 15	Enrichment Score: 0.36	G	Count	P_Value	Benjamini
GOTERM_CC_FAT	endomembrane system	RT	13	9.0E-2	1.0E0
GOTERM_CC_FAT	Golgi apparatus	RT	9	2.0E-1	1.0E0

Category	Term		Count	P_Value	Benjamini
GOTERM_CC_FAT	Golgi apparatus part	RT	7	2.3E-1	1.0E0
GOTERM_CC_FAT	coated membrane	RT	10	3.6E-1	1.0E0
GOTERM_CC_FAT	membrane coat	RT	10	3.6E-1	1.0E0
GOTERM_CC_FAT	organelle membrane	RT	8	4.2E-1	1.0E0
GOTERM_CC_FAT	cytoplasmic vesicle part	RT	5	4.6E-1	1.0E0
GOTERM_CC_FAT	cytoplasmic membrane-bounded vesicle	RT	5	4.6E-1	1.0E0
GOTERM_CC_FAT	vesicle membrane	RT	5	4.6E-1	1.0E0
GOTERM_CC_FAT	cytoplasmic vesicle	RT	5	4.6E-1	1.0E0
GOTERM_CC_FAT	coated vesicle membrane	RT	5	4.6E-1	1.0E0
GOTERM_CC_FAT	cytoplasmic vesicle membrane	RT	5	4.6E-1	1.0E0
GOTERM_CC_FAT	vesicle	RT	5	4.6E-1	1.0E0
GOTERM_CC_FAT	membrane-bounded vesicle	RT	5	4.6E-1	1.0E0
GOTERM_CC_FAT	coated vesicle	RT	5	4.6E-1	1.0E0
GOTERM_CC_FAT	vesicle coat	RT	5	4.6E-1	1.0E0
GOTERM_BP_FAT	ER to Golgi vesicle-mediated transport	RT	5	4.9E-1	1.0E0
GOTERM_BP_FAT	Golgi vesicle transport	RT	5	4.9E-1	1.0E0
GOTERM_CC_FAT	transport vesicle membrane	RT	4	6.1E-1	1.0E0
GOTERM_CC_FAT	transport vesicle	RT	4	6.1E-1	1.0E0
INTERPRO	Clathrin/coatomer adaptor, adaptin-like, N-terminal	RT	4	6.6E-1	1.0E0
GOTERM_CC_FAT	ER to Golgi transport vesicle membrane	RT	3	7.7E-1	1.0E0
GOTERM_CC_FAT	ER to Golgi transport vesicle	RT	3	7.7E-1	1.0E0
GOTERM_CC_FAT	COPII vesicle coat	RT	3	7.7E-1	1.0E0
Annotation Cluster 16	Enrichment Score: 0.32		Count	P_Value	Benjamini
INTERPRO	ATPase, P-type, ATPase-associated region	RT	10	1.0E-1	1.0E0
GOTERM_MF_FAT	ATPase activity, coupled to transmembrane movement of ions, phosphorylative mechanism	RT	10	1.9E-1	1.0E0
INTERPRO	ATPase, P-type, K/Mg/Cd/Cu/Zn/Na/Ca/Na/H-transporter	RT	10	2.0E-1	1.0E0
INTERPRO	ATPase, P-type phosphorylation site	RT	7	2.9E-1	1.0E0
GOTERM_MF_FAT	lipid transporter activity	RT	5	5.1E-1	1.0E0
GOTERM_BP_FAT	lipid transport	RT	4	6.4E-1	1.0E0
GOTERM_BP_FAT	lipid localization	RT	4	6.4E-1	1.0E0
GOTERM_MF_FAT	aminophospholipid transporter activity	RT	4	6.6E-1	1.0E0
GOTERM_MF_FAT	phospholipid transporter activity	RT	4	6.6E-1	1.0E0
GOTERM_MF_FAT	phospholipid-translocating ATPase activity	RT	4	6.6E-1	1.0E0
GOTERM_BP_FAT	phospholipid transport	RT	3	7.9E-1	1.0E0
INTERPRO	ATPase, P-type, phospholipid-translocating, flippase	RT	3	8.1E-1	1.0E0
SP_PIR_KEYWORDS	magnesium	RT	7	8.3E-1	9.9E-1
GOTERM_MF_FAT	magnesium ion binding	RT	8	8.8E-1	1.0E0
Annotation Cluster 17	Enrichment Score: 0.32		Count	P_Value	Benjamini
INTERPRO	Protein synthesis factor, GTP-binding	RT	7	2.9E-1	1.0E0
PIR_SUPERFAMILY	PIRSF003007:translation elongation factor Tu	RT	3	6.2E-1	1.0E0
INTERPRO	Translation elongation factor EFTu/EF1A, C-terminal	RT	3	6.3E-1	1.0E0
Annotation Cluster 18	Enrichment Score: 0.3		Count	P_Value	Benjamini
GOTERM_MF_FAT	peptidase activity	RT	51	2.2E-1	1.0E0
SP_PIR_KEYWORDS	Protease	RT	25	2.3E-1	9.8E-1
GOTERM_BP_FAT	proteolysis	RT	54	3.2E-1	1.0E0
INTERPRO	Peptidase, cysteine peptidase active site	RT	20	3.8E-1	1.0E0
GOTERM_MF_FAT	peptidase activity, acting on L-amino acid peptides	RT	42	4.3E-1	1.0E0
GOTERM_MF_FAT	endopeptidase activity	RT	29	4.3E-1	1.0E0
GOTERM_MF_FAT	cysteine-type endopeptidase activity	RT	15	5.9E-1	1.0E0
GOTERM_MF_FAT	cysteine-type peptidase activity	RT	17	7.8E-1	1.0E0
SP_PIR_KEYWORDS	thiol protease	RT	6	8.2E-1	9.9E-1
INTERPRO	Peptidase C1A, papain	RT	15	8.2E-1	1.0E0
INTERPRO	Peptidase C1A, papain C-terminal	RT	15	8.2E-1	1.0E0
SMART	Pept_C1	RT	15	8.5E-1	1.0E0
Annotation Cluster 19	Enrichment Score: 0.3		Count	P_Value	Benjamini
GOTERM_MF_FAT	vitamin binding	RT	9	2.6E-1	1.0E0

	INTERPRO	Pyridoxal phosphate-dependent transferase, major region, subdomain 1	RT				7	2.9E-1	1.0E0
	GOTERM_MF_FAT	pyridoxal phosphate binding	RT				8	3.4E-1	1.0E0
	GOTERM_MF_FAT	vitamin B6 binding	RT				8	3.4E-1	1.0E0
	INTERPRO	Aminotransferase, class V/Cysteine desulfurase	RT				3	6.3E-1	1.0E0
	INTERPRO	Aminotransferase, class I and II	RT				4	6.6E-1	1.0E0
	GOTERM_MF_FAT	transferase activity, transferring nitrogenous groups	RT				5	6.8E-1	1.0E0
	GOTERM_MF_FAT	transaminase activity	RT				3	9.0E-1	1.0E0
	SP_PIR_KEYWORDS	Aminotransferase	RT				3	9.1E-1	1.0E0
	Annotation Cluster 20	Enrichment Score: 0.29					Count	P_Value	Benjamini
	KEGG_PATHWAY	Purine metabolism	RT				20	3.8E-1	1.0E0
	SP_PIR_KEYWORDS	nucleotidyltransferase	RT				17	3.9E-1	9.9E-1
	KEGG_PATHWAY	RNA polymerase	RT				12	4.0E-1	9.9E-1
	GOTERM_BP_FAT	transcription	RT				17	4.1E-1	1.0E0
	KEGG_PATHWAY	Pyrimidine metabolism	RT				20	4.7E-1	9.9E-1
	SP_PIR_KEYWORDS	dna-directed rna polymerase	RT				9	6.7E-1	9.9E-1
	GOTERM_MF_FAT	DNA-directed RNA polymerase activity	RT				13	8.0E-1	1.0E0
	GOTERM_MF_FAT	RNA polymerase activity	RT				13	8.5E-1	1.0E0
	Annotation Cluster 21	Enrichment Score: 0.28					Count	P_Value	Benjamini
	INTERPRO	ABC transporter, conserved site	RT				15	4.1E-1	1.0E0
	INTERPRO	ABC transporter-like	RT				15	5.1E-1	1.0E0
	COG_ONTOLOGY	Defense mechanisms	RT				14	6.9E-1	1.0E0
	Annotation Cluster 22	Enrichment Score: 0.27					Count	P_Value	Benjamini
	INTERPRO	AMP-dependent synthetase and ligase	RT				6	3.9E-1	1.0E0
	COG_ONTOLOGY	Lipid metabolism	RT				10	4.0E-1	9.9E-1
	GOTERM_MF_FAT	long-chain-fatty-acid-CoA ligase activity	RT				4	6.6E-1	1.0E0
	GOTERM_MF_FAT	fatty-acid ligase activity	RT				4	6.6E-1	1.0E0
	GOTERM_MF_FAT	ligase activity, forming carbon-sulfur bonds	RT				4	6.6E-1	1.0E0
	Annotation Cluster 23	Enrichment Score: 0.26					Count	P_Value	Benjamini
	KEGG_PATHWAY	Mismatch repair	RT				10	3.4E-1	1.0E0
	KEGG_PATHWAY	Nucleotide excision repair	RT				10	5.7E-1	9.9E-1
	KEGG_PATHWAY	DNA replication	RT				11	8.3E-1	1.0E0
	Annotation Cluster 24	Enrichment Score: 0.25					Count	P_Value	Benjamini
	INTERPRO	Protein of unknown function DUF6, transmembrane	RT				4	4.5E-1	1.0E0
	PIR_SUPERFAMILY	PIRSF036436:UCP036436	RT				3	6.2E-1	1.0E0
	INTERPRO	Uncharacterised conserved protein UCP036436, nucleotide-sugar transporter-related	RT				3	6.3E-1	1.0E0
	Annotation Cluster 25	Enrichment Score: 0.24					Count	P_Value	Benjamini
	GOTERM_MF_FAT	exopeptidase activity	RT				8	4.9E-1	1.0E0
	GOTERM_MF_FAT	dipeptidase activity	RT				3	6.3E-1	1.0E0
	SP_PIR_KEYWORDS	dipeptidase	RT				3	6.4E-1	9.9E-1
	Annotation Cluster 26	Enrichment Score: 0.23					Count	P_Value	Benjamini
	GOTERM_MF_FAT	metallopeptidase activity	RT				10	3.1E-1	1.0E0
	GOTERM_MF_FAT	metalloexopeptidase activity	RT				6	3.9E-1	1.0E0
	INTERPRO	Peptidase M24, structural domain	RT				4	4.5E-1	1.0E0
	GOTERM_MF_FAT	exopeptidase activity	RT				8	4.9E-1	1.0E0
	GOTERM_MF_FAT	carboxypeptidase activity	RT				3	8.0E-1	1.0E0
	GOTERM_MF_FAT	metallocarboxypeptidase activity	RT				3	8.0E-1	1.0E0
	INTERPRO	Peptidase M14, carboxypeptidase A	RT				3	9.0E-1	1.0E0
	SMART	Zn_pept	RT				3	9.1E-1	1.0E0
	Annotation Cluster 27	Enrichment Score: 0.22					Count	P_Value	Benjamini
	SP_PIR_KEYWORDS	egf-like domain	RT				12	4.2E-1	9.9E-1
	INTERPRO	EGF, extracellular	RT				14	6.7E-1	1.0E0
	INTERPRO	EGF-like, type 3	RT				19	6.8E-1	1.0E0
	INTERPRO	EGF	RT				5	6.9E-1	1.0E0
	Annotation Cluster 28	Enrichment Score: 0.22					Count	P_Value	Benjamini
	INTERPRO	Nucleic acid binding, OB-fold, tRNA/helicase-type	RT				4	4.5E-1	1.0E0
	SP_PIR_KEYWORDS	protein biosynthesis	RT				7	6.1E-1	9.9E-1
	PIR_SUPERFAMILY	PIRSF001525:lysine-tRNA ligase	RT				3	6.2E-1	1.0E0

						Count	P_Value	Benjamini
	INTERPRO	Aminoacyl-tRNA synthetase, class II (D, K and N)-like	RT			3	6.3E-1	1.0E0
	INTERPRO	Aminoacyl-tRNA synthetase, class II (D, K and N)	RT			3	6.3E-1	1.0E0
	INTERPRO	Aminoacyl-tRNA synthetase, class II, conserved region	RT			6	7.1E-1	1.0E0
	Annotation Cluster 29	Enrichment Score: 0.22	G			Count	P_Value	Benjamini
	GOTERM_CC_FAT	nuclear pore	RT			4	6.1E-1	1.0E0
	GOTERM_CC_FAT	envelope	RT			4	6.1E-1	1.0E0
	GOTERM_CC_FAT	organelle envelope	RT			4	6.1E-1	1.0E0
	GOTERM_CC_FAT	pore complex	RT			4	6.1E-1	1.0E0
	GOTERM_CC_FAT	nuclear envelope	RT			4	6.1E-1	1.0E0
	Annotation Cluster 30	Enrichment Score: 0.21	G			Count	P_Value	Benjamini
	GOTERM_BP_FAT	carbohydrate biosynthetic process	RT			6	3.6E-1	1.0E0
	GOTERM_BP_FAT	cellular carbohydrate biosynthetic process	RT			6	3.6E-1	1.0E0
	GOTERM_BP_FAT	alcohol biosynthetic process	RT			4	6.4E-1	1.0E0
	GOTERM_BP_FAT	hexose biosynthetic process	RT			3	7.9E-1	1.0E0
	GOTERM_BP_FAT	pyruvate metabolic process	RT			3	7.9E-1	1.0E0
	GOTERM_BP_FAT	monosaccharide biosynthetic process	RT			3	7.9E-1	1.0E0
	GOTERM_BP_FAT	gluconeogenesis	RT			3	7.9E-1	1.0E0
	Annotation Cluster 31	Enrichment Score: 0.21	G			Count	P_Value	Benjamini
	GOTERM_BP_FAT	regulation of RNA metabolic process	RT			9	3.6E-1	1.0E0
	GOTERM_BP_FAT	regulation of transcription, DNA-dependent	RT			9	3.6E-1	1.0E0
	GOTERM_BP_FAT	regulation of transcription	RT			19	5.4E-1	1.0E0
	GOTERM_MF_FAT	NAD or NADH binding	RT			6	5.6E-1	1.0E0
	GOTERM_BP_FAT	negative regulation of RNA metabolic process	RT			4	6.4E-1	1.0E0
	GOTERM_BP_FAT	chromatin silencing	RT			4	6.4E-1	1.0E0
	GOTERM_BP_FAT	negative regulation of nitrogen compound metabolic process	RT			4	6.4E-1	1.0E0
	GOTERM_BP_FAT	regulation of gene expression, epigenetic	RT			4	6.4E-1	1.0E0
	GOTERM_BP_FAT	negative regulation of transcription	RT			4	6.4E-1	1.0E0
	GOTERM_BP_FAT	negative regulation of nucleobase, nucleoside, nucleotide and nucleic acid metabolic process	RT			4	6.4E-1	1.0E0
	GOTERM_BP_FAT	negative regulation of gene expression, epigenetic	RT			4	6.4E-1	1.0E0
	GOTERM_BP_FAT	negative regulation of transcription, DNA-dependent	RT			4	6.4E-1	1.0E0
	GOTERM_BP_FAT	negative regulation of macromolecule biosynthetic process	RT			4	6.4E-1	1.0E0
	GOTERM_BP_FAT	negative regulation of biosynthetic process	RT			4	6.4E-1	1.0E0
	GOTERM_BP_FAT	negative regulation of cellular biosynthetic process	RT			4	6.4E-1	1.0E0
	GOTERM_MF_FAT	NAD binding	RT			4	6.6E-1	1.0E0
	GOTERM_BP_FAT	gene silencing	RT			5	6.6E-1	1.0E0
	GOTERM_BP_FAT	negative regulation of gene expression	RT			5	6.6E-1	1.0E0
	GOTERM_BP_FAT	negative regulation of macromolecule metabolic process	RT			5	6.6E-1	1.0E0
	INTERPRO	NAD-dependent histone deacetylase, silent information regulator Sir2	RT			4	6.6E-1	1.0E0
	GOTERM_BP_FAT	protein amino acid deacetylation	RT			4	7.8E-1	1.0E0
	GOTERM_MF_FAT	hydrolase activity, acting on carbon-nitrogen (but not peptide) bonds, in linear amides	RT			5	8.0E-1	1.0E0
	Annotation Cluster 32	Enrichment Score: 0.2	G			Count	P_Value	Benjamini
	GOTERM_BP_FAT	lipid biosynthetic process	RT			11	2.1E-1	1.0E0
	GOTERM_BP_FAT	phospholipid biosynthetic process	RT			6	3.6E-1	1.0E0
	GOTERM_BP_FAT	organophosphate metabolic process	RT			9	5.0E-1	1.0E0
	GOTERM_BP_FAT	phospholipid metabolic process	RT			8	5.9E-1	1.0E0
	INTERPRO	CDP-alcohol phosphatidyltransferase	RT			3	6.3E-1	1.0E0
	GOTERM_MF_FAT	phosphotransferase activity, for other substituted phosphate groups	RT			3	8.0E-1	1.0E0
	KEGG_PATHWAY	Inositol phosphate metabolism	RT			3	8.7E-1	1.0E0
	GOTERM_BP_FAT	glycerophospholipid metabolic process	RT			3	9.7E-1	1.0E0
	GOTERM_BP_FAT	phosphoinositide metabolic process	RT			3	9.7E-1	1.0E0
	GOTERM_BP_FAT	glycerolipid metabolic process	RT			3	9.7E-1	1.0E0
	Annotation Cluster 33	Enrichment Score: 0.2	G			Count	P_Value	Benjamini
	INTERPRO	SET	RT			5	5.2E-1	1.0E0
	SMART	SET	RT			5	5.4E-1	1.0E0

INTERPRO	Post-SET zinc-binding region	RT	3	6.3E-1	1.0E0
SMART	PostSET	RT	3	6.5E-1	1.0E0
SP_PIR_KEYWORDS	methyltransferase	RT	11	9.0E-1	1.0E0
Annotation Cluster 34	Enrichment Score: 0.2		Count	P_Value	Benjamini
GOTERM_BP_FAT	protein amino acid lipidation	RT	4	6.4E-1	1.0E0
GOTERM_BP_FAT	lipoprotein biosynthetic process	RT	4	6.4E-1	1.0E0
GOTERM_BP_FAT	lipoprotein metabolic process	RT	4	6.4E-1	1.0E0
Annotation Cluster 35	Enrichment Score: 0.2		Count	P_Value	Benjamini
GOTERM_MF_FAT	acetyltransferase activity	RT	7	5.9E-1	1.0E0
GOTERM_MF_FAT	N-acyltransferase activity	RT	7	5.9E-1	1.0E0
INTERPRO	Acyl-CoA N-acyltransferase	RT	7	6.0E-1	1.0E0
GOTERM_MF_FAT	N-acetyltransferase activity	RT	6	7.0E-1	1.0E0
INTERPRO	GCN5-related N-acetyltransferase	RT	6	7.1E-1	1.0E0
Annotation Cluster 36	Enrichment Score: 0.19		Count	P_Value	Benjamini
GOTERM_MF_FAT	FAD binding	RT	5	5.1E-1	1.0E0
INTERPRO	Pyridine nucleotide-disulphide oxidoreductase, NAD-binding region	RT	3	6.3E-1	1.0E0
INTERPRO	FAD-dependent pyridine nucleotide-disulphide oxidoreductase	RT	3	8.1E-1	1.0E0
Annotation Cluster 37	Enrichment Score: 0.19		Count	P_Value	Benjamini
INTERPRO	ARF/SAR superfamily	RT	6	3.9E-1	1.0E0
PIR_SUPERFAMILY	PIRSF001711:ADP-ribosylation factor	RT	4	6.5E-1	1.0E0
INTERPRO	ADP-ribosylation factor	RT	3	8.1E-1	1.0E0
SMART	ARF	RT	3	8.2E-1	1.0E0
Annotation Cluster 38	Enrichment Score: 0.19		Count	P_Value	Benjamini
SP_PIR_KEYWORDS	Transcription	RT	6	5.7E-1	9.9E-1
INTERPRO	RNA polymerase Rpb1, domain 3	RT	3	6.3E-1	1.0E0
INTERPRO	RNA polymerase, N-terminal	RT	3	6.3E-1	1.0E0
INTERPRO	RNA polymerase Rpb1, domain 1	RT	3	6.3E-1	1.0E0
INTERPRO	RNA polymerase Rpb1, domain 5	RT	3	6.3E-1	1.0E0
INTERPRO	RNA polymerase Rpb1, domain 4	RT	3	6.3E-1	1.0E0
INTERPRO	RNA polymerase, alpha subunit	RT	3	6.3E-1	1.0E0
SMART	RPOLA_N	RT	3	6.5E-1	1.0E0
SP_PIR_KEYWORDS	dna-directed rna polymerase	RT	9	6.7E-1	9.9E-1
GOTERM_MF_FAT	ribonucleoside binding	RT	3	8.0E-1	1.0E0
Annotation Cluster 39	Enrichment Score: 0.16		Count	P_Value	Benjamini
GOTERM_BP_FAT	response to drug	RT	4	6.4E-1	1.0E0
GOTERM_BP_FAT	multidrug transport	RT	4	6.4E-1	1.0E0
GOTERM_BP_FAT	drug transport	RT	4	6.4E-1	1.0E0
GOTERM_MF_FAT	drug transporter activity	RT	4	6.6E-1	1.0E0
INTERPRO	Multi antimicrobial extrusion protein MatE	RT	4	6.6E-1	1.0E0
GOTERM_MF_FAT	antiporter activity	RT	4	8.0E-1	1.0E0
INTERPRO	Na+ driven multidrug efflux pump	RT	3	8.1E-1	1.0E0
Annotation Cluster 40	Enrichment Score: 0.16		Count	P_Value	Benjamini
GOTERM_BP_FAT	cellular response to stress	RT	13	6.0E-1	1.0E0
GOTERM_BP_FAT	DNA repair	RT	12	6.7E-1	1.0E0
GOTERM_BP_FAT	response to DNA damage stimulus	RT	12	6.7E-1	1.0E0
GOTERM_BP_FAT	DNA metabolic process	RT	32	8.2E-1	1.0E0
Annotation Cluster 41	Enrichment Score: 0.16		Count	P_Value	Benjamini
GOTERM_BP_FAT	rRNA metabolic process	RT	9	5.0E-1	1.0E0
GOTERM_BP_FAT	rRNA processing	RT	9	5.0E-1	1.0E0
GOTERM_BP_FAT	ribonucleoprotein complex biogenesis	RT	12	6.7E-1	1.0E0
GOTERM_BP_FAT	ribosome biogenesis	RT	12	6.7E-1	1.0E0
GOTERM_BP_FAT	ncRNA processing	RT	16	7.9E-1	1.0E0
GOTERM_BP_FAT	tRNA processing	RT	8	9.5E-1	1.0E0
GOTERM_BP_FAT	RNA processing	RT	22	9.6E-1	1.0E0
Annotation Cluster 42	Enrichment Score: 0.16		Count	P_Value	Benjamini
INTERPRO	Nucleotide-binding, alpha-beta plait	RT	11	4.9E-1	1.0E0
INTERPRO	RNA recognition motif, RNP-1	RT	8	8.3E-1	1.0E0
SMART	RRM	RT	8	8.5E-1	1.0E0
Annotation Cluster 43	Enrichment Score: 0.14		Count	P_Value	Benjamini

GOTERM_MF_FAT	protein kinase regulator activity	RT	5	6.8E-1	1.0E0
GOTERM_MF_FAT	kinase regulator activity	RT	5	6.8E-1	1.0E0
GOTERM_MF_FAT	cAMP-dependent protein kinase regulator activity	RT	3	8.0E-1	1.0E0
Annotation Cluster 44	Enrichment Score: 0.14	G	Count	P_Value	Benjamini
GOTERM_BP_FAT	translation	RT	67	4.0E-1	1.0E0
GOTERM_MF_FAT	structural molecule activity	RT	50	5.0E-1	1.0E0
KEGG_PATHWAY	Ribosome	RT	38	6.2E-1	9.9E-1
GOTERM_CC_FAT	ribonucleoprotein complex	RT	54	7.4E-1	1.0E0
GOTERM_MF_FAT	structural constituent of ribosome	RT	39	7.6E-1	1.0E0
GOTERM_CC_FAT	ribosome	RT	40	8.1E-1	1.0E0
SP_PIR_KEYWORDS	ribosomal protein	RT	37	9.6E-1	1.0E0
GOTERM_CC_FAT	intracellular non-membrane-bounded organelle	RT	80	9.8E-1	1.0E0
GOTERM_CC_FAT	non-membrane-bounded organelle	RT	80	9.8E-1	1.0E0
Annotation Cluster 45	Enrichment Score: 0.14	G	Count	P_Value	Benjamini
GOTERM_MF_FAT	tubulin-tyrosine ligase activity	RT	5	6.8E-1	1.0E0
INTERPRO	Tubulin-tyrosine ligase	RT	5	6.9E-1	1.0E0
GOTERM_MF_FAT	acid-amino acid ligase activity	RT	15	8.2E-1	1.0E0
Annotation Cluster 46	Enrichment Score: 0.14	G	Count	P_Value	Benjamini
GOTERM_MF_FAT	lipid binding	RT	23	4.4E-1	1.0E0
GOTERM_MF_FAT	phospholipid binding	RT	17	5.5E-1	1.0E0
GOTERM_MF_FAT	calcium-dependent phospholipid binding	RT	14	5.7E-1	1.0E0
INTERPRO	Annexin repeat	RT	9	6.6E-1	1.0E0
SMART	ANX	RT	9	6.9E-1	1.0E0
INTERPRO	Annexin	RT	9	8.3E-1	1.0E0
SP_PIR_KEYWORDS	annexin	RT	9	8.4E-1	9.9E-1
GOTERM_MF_FAT	calcium ion binding	RT	20	9.8E-1	1.0E0
INTERPRO	Alpha giardin	RT	5	9.8E-1	1.0E0
GOTERM_BP_FAT	cytoskeleton organization	RT	6	9.9E-1	1.0E0
Annotation Cluster 47	Enrichment Score: 0.13	G	Count	P_Value	Benjamini
INTERPRO	SANT, DNA-binding	RT	7	6.0E-1	1.0E0
SMART	SANT	RT	7	6.3E-1	1.0E0
INTERPRO	Myb-type HTH DNA-binding domain	RT	3	8.1E-1	1.0E0
INTERPRO	Myb transcription factor	RT	3	8.1E-1	1.0E0
INTERPRO	Myb, DNA-binding	RT	3	9.0E-1	1.0E0
Annotation Cluster 48	Enrichment Score: 0.13	G	Count	P_Value	Benjamini
GOTERM_BP_FAT	glucose metabolic process	RT	13	5.0E-1	1.0E0
GOTERM_BP_FAT	hexose metabolic process	RT	13	5.0E-1	1.0E0
GOTERM_BP_FAT	monosaccharide metabolic process	RT	13	7.7E-1	1.0E0
GOTERM_BP_FAT	hexose catabolic process	RT	8	8.0E-1	1.0E0
GOTERM_BP_FAT	glucose catabolic process	RT	8	8.0E-1	1.0E0
GOTERM_BP_FAT	monosaccharide catabolic process	RT	8	8.0E-1	1.0E0
GOTERM_BP_FAT	alcohol catabolic process	RT	8	8.0E-1	1.0E0
GOTERM_BP_FAT	carbohydrate catabolic process	RT	8	8.0E-1	1.0E0
GOTERM_BP_FAT	cellular carbohydrate catabolic process	RT	8	8.0E-1	1.0E0
SP_PIR_KEYWORDS	glycolysis	RT	4	8.1E-1	1.0E0
GOTERM_BP_FAT	glycolysis	RT	5	9.6E-1	1.0E0
Annotation Cluster 49	Enrichment Score: 0.13	G	Count	P_Value	Benjamini
GOTERM_BP_FAT	modification-dependent macromolecule catabolic process	RT	16	5.7E-1	1.0E0
GOTERM_BP_FAT	modification-dependent protein catabolic process	RT	16	5.7E-1	1.0E0
GOTERM_BP_FAT	macromolecule catabolic process	RT	23	6.2E-1	1.0E0
GOTERM_BP_FAT	cellular macromolecule catabolic process	RT	20	6.4E-1	1.0E0
KEGG_PATHWAY	Proteasome	RT	16	6.5E-1	9.9E-1
GOTERM_BP_FAT	protein catabolic process	RT	22	6.7E-1	1.0E0
INTERPRO	Proteasome, alpha-subunit, conserved site	RT	5	6.9E-1	1.0E0
GOTERM_BP_FAT	cellular protein catabolic process	RT	19	7.0E-1	1.0E0
GOTERM_BP_FAT	proteolysis involved in cellular protein catabolic process	RT	19	7.0E-1	1.0E0
INTERPRO	Proteasome, alpha and beta subunits	RT	8	7.4E-1	1.0E0

SP_PIR_KEYWORDS	ubl conjugation pathway	RT		8	7.5E-1	9.9E-1
GOTERM_CC_FAT	proteasome core complex	RT		8	7.7E-1	1.0E0
PIR_SUPERFAMILY	PIRSF001212:multicatalytic endopeptidase complex chain C9	RT		4	7.9E-1	1.0E0
GOTERM_BP_FAT	ubiquitin-dependent protein catabolic process	RT		8	8.0E-1	1.0E0
GOTERM_MF_FAT	threonine-type endopeptidase activity	RT		8	8.2E-1	1.0E0
GOTERM_MF_FAT	threonine-type peptidase activity	RT		8	8.2E-1	1.0E0
INTERPRO	Proteasome, subunit alpha/beta	RT		8	8.3E-1	1.0E0
GOTERM_CC_FAT	proteasome complex	RT		10	9.3E-1	1.0E0
SP_PIR_KEYWORDS	proteasome	RT		10	9.4E-1	1.0E0
SP_PIR_KEYWORDS	threonine protease	RT		3	9.6E-1	1.0E0
GOTERM_CC_FAT	cytosol	RT		13	9.7E-1	1.0E0
Annotation Cluster 50	Enrichment Score: 0.12	G		Count	P_Value	Benjamini
GOTERM_BP_FAT	secondary metabolic process	RT		4	6.4E-1	1.0E0
GOTERM_BP_FAT	alkaloid metabolic process	RT		4	6.4E-1	1.0E0
GOTERM_BP_FAT	cellular amide metabolic process	RT		4	6.4E-1	1.0E0
GOTERM_BP_FAT	nicotinamide metabolic process	RT		4	6.4E-1	1.0E0
GOTERM_BP_FAT	nicotinamide nucleotide metabolic process	RT		4	6.4E-1	1.0E0
GOTERM_BP_FAT	pyridine nucleotide metabolic process	RT		4	7.8E-1	1.0E0
GOTERM_BP_FAT	oxidoreduction coenzyme metabolic process	RT		4	7.8E-1	1.0E0
GOTERM_BP_FAT	coenzyme metabolic process	RT		6	7.9E-1	1.0E0
GOTERM_BP_FAT	cofactor metabolic process	RT		6	9.2E-1	1.0E0
GOTERM_BP_FAT	coenzyme biosynthetic process	RT		3	9.7E-1	1.0E0
GOTERM_BP_FAT	cofactor biosynthetic process	RT		3	9.9E-1	1.0E0
Annotation Cluster 51	Enrichment Score: 0.12	G		Count	P_Value	Benjamini
SP_PIR_KEYWORDS	serine/threonine-protein kinase	RT		38	4.6E-1	9.8E-1
GOTERM_MF_FAT	protein serine/threonine kinase activity	RT		60	6.8E-1	1.0E0
INTERPRO	Protein kinase, ATP binding site	RT		47	7.3E-1	1.0E0
INTERPRO	Serine/threonine protein kinase, active site	RT		51	7.3E-1	1.0E0
INTERPRO	Serine/threonine protein kinase-related	RT		127	9.3E-1	1.0E0
INTERPRO	Serine/threonine protein kinase	RT		29	9.6E-1	1.0E0
SMART	S_TKc	RT		29	9.7E-1	1.0E0
Annotation Cluster 52	Enrichment Score: 0.12	G		Count	P_Value	Benjamini
SP_PIR_KEYWORDS	protein biosynthesis	RT		7	6.1E-1	9.9E-1
INTERPRO	Aminoacyl-tRNA synthetase, class II, conserved region	RT		6	7.1E-1	1.0E0
GOTERM_MF_FAT	ligase activity, forming carbon-oxygen bonds	RT		14	7.4E-1	1.0E0
GOTERM_MF_FAT	aminoacyl-tRNA ligase activity	RT		14	7.4E-1	1.0E0
GOTERM_MF_FAT	ligase activity, forming aminoacyl-tRNA and related compounds	RT		14	7.4E-1	1.0E0
GOTERM_BP_FAT	ncRNA metabolic process	RT		29	7.5E-1	1.0E0
GOTERM_BP_FAT	amino acid activation	RT		13	7.7E-1	1.0E0
GOTERM_BP_FAT	tRNA aminoacylation	RT		13	7.7E-1	1.0E0
SP_PIR_KEYWORDS	Aminoacyl-tRNA synthetase	RT		13	8.2E-1	9.9E-1
GOTERM_BP_FAT	tRNA aminoacylation for protein translation	RT		12	8.2E-1	1.0E0
KEGG_PATHWAY	Aminoacyl-tRNA biosynthesis	RT		11	8.3E-1	1.0E0
GOTERM_BP_FAT	tRNA metabolic process	RT		21	8.7E-1	1.0E0
Annotation Cluster 53	Enrichment Score: 0.12	G		Count	P_Value	Benjamini
PIR_SUPERFAMILY	PIRSF002585:heat shock protein DnaJ	RT		3	6.2E-1	1.0E0
INTERPRO	Heat shock protein DnaJ	RT		3	6.3E-1	1.0E0
INTERPRO	Chaperone DnaJ, C-terminal	RT		3	6.3E-1	1.0E0
INTERPRO	Molecular chaperone, heat shock protein, Hsp40, DnaJ	RT		5	6.9E-1	1.0E0
GOTERM_MF_FAT	heat shock protein binding	RT		5	8.0E-1	1.0E0
INTERPRO	Heat shock protein DnaJ, N-terminal	RT		5	8.1E-1	1.0E0
SMART	DnaJ	RT		5	8.2E-1	1.0E0
SP_PIR_KEYWORDS	Chaperone	RT		5	9.8E-1	1.0E0
GOTERM_MF_FAT	unfolded protein binding	RT		9	9.8E-1	1.0E0
Annotation Cluster 54	Enrichment Score: 0.12	G		Count	P_Value	Benjamini
INTERPRO	Kelch-type beta propeller	RT		5	6.9E-1	1.0E0
INTERPRO	Kelch repeat type 1	RT		4	8.0E-1	1.0E0

Category	Term	RT	Count	P_Value	Benjamini
SMART	Kelch	RT	4	8.2E-1	1.0E0
Annotation Cluster 55	Enrichment Score: 0.11		Count	P_Value	Benjamini
INTERPRO	Tyrosine protein kinase	RT	5	6.9E-1	1.0E0
SMART	TyrKc	RT	5	7.1E-1	1.0E0
GOTERM_MF_FAT	protein tyrosine kinase activity	RT	12	9.7E-1	1.0E0
Annotation Cluster 56	Enrichment Score: 0.11		Count	P_Value	Benjamini
GOTERM_CC_FAT	cell projection	RT	3	7.7E-1	1.0E0
GOTERM_CC_FAT	flagellum	RT	3	7.7E-1	1.0E0
SP_PIR_KEYWORDS	flagellum	RT	3	8.1E-1	9.9E-1
Annotation Cluster 57	Enrichment Score: 0.1		Count	P_Value	Benjamini
INTERPRO	Dynein heavy chain, N-terminal region 2	RT	8	5.0E-1	1.0E0
COG_ONTOLOGY	Cytoskeleton	RT	13	7.5E-1	1.0E0
GOTERM_CC_FAT	dynein complex	RT	7	7.6E-1	1.0E0
INTERPRO	Dynein heavy chain	RT	7	8.2E-1	1.0E0
GOTERM_CC_FAT	microtubule associated complex	RT	8	8.4E-1	1.0E0
GOTERM_MF_FAT	motor activity	RT	20	9.7E-1	1.0E0
GOTERM_MF_FAT	microtubule motor activity	RT	20	9.7E-1	1.0E0
Annotation Cluster 58	Enrichment Score: 0.1		Count	P_Value	Benjamini
GOTERM_BP_FAT	membrane docking	RT	3	7.9E-1	1.0E0
GOTERM_BP_FAT	exocytosis	RT	3	7.9E-1	1.0E0
GOTERM_BP_FAT	secretion	RT	3	7.9E-1	1.0E0
GOTERM_BP_FAT	vesicle docking during exocytosis	RT	3	7.9E-1	1.0E0
GOTERM_BP_FAT	vesicle docking	RT	3	7.9E-1	1.0E0
GOTERM_BP_FAT	secretion by cell	RT	3	7.9E-1	1.0E0
INTERPRO	Sec1-like protein	RT	3	8.1E-1	1.0E0
Annotation Cluster 59	Enrichment Score: 0.1		Count	P_Value	Benjamini
INTERPRO	Zinc finger, C2H2-type	RT	11	7.0E-1	1.0E0
INTERPRO	Zinc finger, C2H2-like	RT	10	8.4E-1	1.0E0
SMART	ZnF_C2H2	RT	10	8.6E-1	1.0E0
Annotation Cluster 60	Enrichment Score: 0.1		Count	P_Value	Benjamini
INTERPRO	Beta tubulin	RT	3	6.3E-1	1.0E0
GOTERM_BP_FAT	protein complex assembly	RT	8	7.1E-1	1.0E0
GOTERM_BP_FAT	protein complex biogenesis	RT	8	7.1E-1	1.0E0
GOTERM_BP_FAT	protein polymerization	RT	5	7.8E-1	1.0E0
GOTERM_BP_FAT	macromolecular complex subunit organization	RT	15	7.8E-1	1.0E0
INTERPRO	Beta tubulin, autoregulation binding site	RT	4	8.0E-1	1.0E0
INTERPRO	Tubulin, conserved site	RT	5	8.1E-1	1.0E0
INTERPRO	Tubulin/FtsZ, GTPase domain	RT	5	8.1E-1	1.0E0
INTERPRO	Tubulin	RT	5	8.1E-1	1.0E0
GOTERM_BP_FAT	macromolecular complex assembly	RT	14	8.3E-1	1.0E0
GOTERM_BP_FAT	cellular protein complex assembly	RT	6	8.7E-1	1.0E0
GOTERM_BP_FAT	cellular macromolecular complex subunit organization	RT	13	8.8E-1	1.0E0
PIR_SUPERFAMILY	PIRSF002306:tubulin	RT	4	8.8E-1	1.0E0
INTERPRO	Tubulin/FtsZ, 2-layer sandwich domain	RT	4	8.9E-1	1.0E0
GOTERM_BP_FAT	cellular macromolecular complex assembly	RT	12	9.1E-1	1.0E0
Annotation Cluster 61	Enrichment Score: 0.09		Count	P_Value	Benjamini
INTERPRO	Copine	RT	4	6.6E-1	1.0E0
INTERPRO	von Willebrand factor, type A	RT	4	8.9E-1	1.0E0
SMART	VWA	RT	4	9.0E-1	1.0E0
Annotation Cluster 62	Enrichment Score: 0.09		Count	P_Value	Benjamini
SP_PIR_KEYWORDS	nucleotide-binding	RT	162	4.3E-1	9.8E-1
GOTERM_BP_FAT	protein amino acid phosphorylation	RT	152	6.5E-1	1.0E0
SP_PIR_KEYWORDS	atp-binding	RT	150	6.6E-1	9.9E-1
GOTERM_BP_FAT	phosphorylation	RT	162	6.9E-1	1.0E0
GOTERM_BP_FAT	phosphorus metabolic process	RT	170	7.4E-1	1.0E0
GOTERM_BP_FAT	phosphate metabolic process	RT	170	7.4E-1	1.0E0
GOTERM_MF_FAT	protein kinase activity	RT	154	7.4E-1	1.0E0
INTERPRO	Protein kinase, core	RT	151	7.9E-1	1.0E0

GOTERM_MF_FAT	nucleotide binding	RT	352	8.1E-1	1.0E0
GOTERM_MF_FAT	purine nucleotide binding	RT	328	8.8E-1	1.0E0
GOTERM_MF_FAT	purine ribonucleotide binding	RT	322	9.2E-1	1.0E0
GOTERM_MF_FAT	ribonucleotide binding	RT	322	9.2E-1	1.0E0
SP_PIR_KEYWORDS	kinase	RT	184	9.3E-1	1.0E0
INTERPRO	Serine/threonine protein kinase-related	RT	127	9.3E-1	1.0E0
GOTERM_MF_FAT	nucleoside binding	RT	295	9.8E-1	1.0E0
GOTERM_MF_FAT	adenyl nucleotide binding	RT	292	9.8E-1	1.0E0
GOTERM_MF_FAT	purine nucleoside binding	RT	292	9.8E-1	1.0E0
GOTERM_MF_FAT	adenyl ribonucleotide binding	RT	286	9.9E-1	1.0E0
GOTERM_MF_FAT	ATP binding	RT	286	9.9E-1	1.0E0
Annotation Cluster 63	Enrichment Score: 0.09		Count	P_Value	Benjamini
GOTERM_MF_FAT	DNA-directed DNA polymerase activity	RT	7	5.9E-1	1.0E0
SP_PIR_KEYWORDS	dna replication	RT	6	8.2E-1	9.9E-1
INTERPRO	DNA-directed DNA polymerase, family B	RT	3	9.0E-1	1.0E0
SP_PIR_KEYWORDS	dna-directed dna polymerase	RT	3	9.1E-1	1.0E0
SMART	POLBc	RT	3	9.1E-1	1.0E0
Annotation Cluster 64	Enrichment Score: 0.08		Count	P_Value	Benjamini
GOTERM_MF_FAT	ATPase activity	RT	55	6.2E-1	1.0E0
GOTERM_MF_FAT	ATPase activity, coupled	RT	41	6.4E-1	1.0E0
INTERPRO	DNA/RNA helicase, DEAD/DEAH box type, N-terminal	RT	18	6.6E-1	1.0E0
GOTERM_MF_FAT	purine NTP-dependent helicase activity	RT	21	8.2E-1	1.0E0
GOTERM_MF_FAT	ATP-dependent helicase activity	RT	21	8.2E-1	1.0E0
INTERPRO	DEAD-like helicase, N-terminal	RT	23	8.8E-1	1.0E0
INTERPRO	Helicase, superfamily 1 and 2, ATP-binding	RT	23	8.8E-1	1.0E0
INTERPRO	RNA helicase, DEAD-box type, Q motif	RT	9	8.9E-1	1.0E0
SMART	DEXDc	RT	23	9.1E-1	1.0E0
INTERPRO	DNA/RNA helicase, C-terminal	RT	23	9.1E-1	1.0E0
SP_PIR_KEYWORDS	helicase	RT	23	9.2E-1	1.0E0
GOTERM_MF_FAT	helicase activity	RT	28	9.2E-1	1.0E0
SMART	HELICc	RT	23	9.3E-1	1.0E0
Annotation Cluster 65	Enrichment Score: 0.08		Count	P_Value	Benjamini
GOTERM_MF_FAT	cis-trans isomerase activity	RT	5	8.0E-1	1.0E0
GOTERM_MF_FAT	peptidyl-prolyl cis-trans isomerase activity	RT	5	8.0E-1	1.0E0
INTERPRO	Peptidyl-prolyl cis-trans isomerase, FKBP-type	RT	4	8.0E-1	1.0E0
SP_PIR_KEYWORDS	Rotamase	RT	5	8.1E-1	9.9E-1
GOTERM_BP_FAT	protein folding	RT	15	9.2E-1	1.0E0
Annotation Cluster 66	Enrichment Score: 0.08		Count	P_Value	Benjamini
GOTERM_MF_FAT	adenylyltransferase activity	RT	4	6.6E-1	1.0E0
GOTERM_BP_FAT	water-soluble vitamin biosynthetic process	RT	3	8.9E-1	1.0E0
GOTERM_BP_FAT	vitamin metabolic process	RT	3	8.9E-1	1.0E0
GOTERM_BP_FAT	water-soluble vitamin metabolic process	RT	3	8.9E-1	1.0E0
GOTERM_BP_FAT	vitamin biosynthetic process	RT	3	8.9E-1	1.0E0
Annotation Cluster 67	Enrichment Score: 0.08		Count	P_Value	Benjamini
SP_PIR_KEYWORDS	ligase	RT	33	6.2E-1	9.9E-1
SP_PIR_KEYWORDS	ubl conjugation pathway	RT	8	7.5E-1	9.9E-1
PIR_SUPERFAMILY	PIRSF001567:ubiquitin-protein ligase E2	RT	4	7.9E-1	1.0E0
GOTERM_MF_FAT	acid-amino acid ligase activity	RT	15	8.2E-1	1.0E0
GOTERM_MF_FAT	ligase activity, forming carbon-nitrogen bonds	RT	17	8.8E-1	1.0E0
INTERPRO	Ubiquitin-conjugating enzyme/RWD-like	RT	6	9.3E-1	1.0E0
INTERPRO	Ubiquitin-conjugating enzyme, E2	RT	6	9.3E-1	1.0E0
SMART	UBCc	RT	6	9.4E-1	1.0E0
GOTERM_MF_FAT	small conjugating protein ligase activity	RT	6	9.6E-1	1.0E0
Annotation Cluster 68	Enrichment Score: 0.07		Count	P_Value	Benjamini
GOTERM_MF_FAT	DNA polymerase activity	RT	20	6.9E-1	1.0E0
GOTERM_MF_FAT	RNA-directed DNA polymerase activity	RT	13	8.5E-1	1.0E0
GOTERM_MF_FAT	nuclease activity	RT	27	8.6E-1	1.0E0
	rna-directed dna polymerase	RT	12	9.1E-1	1.0E0

SP_PIR_KEYWORDS					
GOTERM_MF_FAT	endonuclease activity	RT	17	9.4E-1	1.0E0
SP_PIR_KEYWORDS	Endonuclease	RT	15	9.4E-1	1.0E0
Annotation Cluster 69	Enrichment Score: 0.06		Count	P_Value	Benjamini
GOTERM_MF_FAT	nucleobase, nucleoside, nucleotide kinase activity	RT	6	8.1E-1	1.0E0
GOTERM_MF_FAT	phosphotransferase activity, phosphate group as acceptor	RT	4	8.9E-1	1.0E0
GOTERM_MF_FAT	nucleotide kinase activity	RT	3	9.0E-1	1.0E0
Annotation Cluster 70	Enrichment Score: 0.06		Count	P_Value	Benjamini
GOTERM_MF_FAT	iron ion binding	RT	19	5.9E-1	1.0E0
SP_PIR_KEYWORDS	iron	RT	11	8.6E-1	9.9E-1
INTERPRO	Serine/threonine-specific protein phosphatase and bis(5-nucleosyl)-tetraphosphatase	RT	8	8.9E-1	1.0E0
SMART	PP2Ac	RT	8	9.0E-1	1.0E0
GOTERM_MF_FAT	phosphoprotein phosphatase activity	RT	14	9.3E-1	1.0E0
PIR_SUPERFAMILY	PIRSF000908:serine/threonine-protein phosphatase, conventional type	RT	4	9.4E-1	1.0E0
GOTERM_MF_FAT	phosphatase activity	RT	22	9.5E-1	1.0E0
INTERPRO	Metallophosphoesterase	RT	9	9.8E-1	1.0E0
Annotation Cluster 71	Enrichment Score: 0.06		Count	P_Value	Benjamini
GOTERM_CC_FAT	nuclear lumen	RT	3	8.8E-1	1.0E0
GOTERM_CC_FAT	membrane-enclosed lumen	RT	3	8.8E-1	1.0E0
GOTERM_CC_FAT	intracellular organelle lumen	RT	3	8.8E-1	1.0E0
GOTERM_CC_FAT	organelle lumen	RT	3	8.8E-1	1.0E0
Annotation Cluster 72	Enrichment Score: 0.05		Count	P_Value	Benjamini
GOTERM_MF_FAT	heme binding	RT	4	8.9E-1	1.0E0
GOTERM_MF_FAT	tetrapyrrole binding	RT	4	8.9E-1	1.0E0
INTERPRO	Cytochrome b5	RT	3	9.0E-1	1.0E0
SP_PIR_KEYWORDS	heme	RT	3	9.1E-1	1.0E0
Annotation Cluster 73	Enrichment Score: 0.04		Count	P_Value	Benjamini
GOTERM_MF_FAT	phosphoinositide binding	RT	3	9.0E-1	1.0E0
INTERPRO	Phox-like	RT	3	9.0E-1	1.0E0
SMART	PX	RT	3	9.1E-1	1.0E0
Annotation Cluster 74	Enrichment Score: 0.04		Count	P_Value	Benjamini
INTERPRO	Kinesin, motor region, conserved site	RT	11	7.0E-1	1.0E0
GOTERM_CC_FAT	microtubule	RT	17	8.6E-1	1.0E0
GOTERM_CC_FAT	cytoskeleton	RT	28	8.6E-1	1.0E0
SP_PIR_KEYWORDS	microtubule	RT	17	8.9E-1	1.0E0
GOTERM_BP_FAT	microtubule-based movement	RT	24	9.1E-1	1.0E0
GOTERM_CC_FAT	cytoskeletal part	RT	26	9.1E-1	1.0E0
GOTERM_CC_FAT	microtubule cytoskeleton	RT	26	9.1E-1	1.0E0
SP_PIR_KEYWORDS	motor protein	RT	12	9.4E-1	1.0E0
GOTERM_BP_FAT	microtubule-based process	RT	26	9.7E-1	1.0E0
GOTERM_MF_FAT	motor activity	RT	20	9.7E-1	1.0E0
GOTERM_MF_FAT	microtubule motor activity	RT	20	9.7E-1	1.0E0
INTERPRO	Kinesin, motor region	RT	12	9.9E-1	1.0E0
SMART	KISc	RT	12	9.9E-1	1.0E0
Annotation Cluster 75	Enrichment Score: 0.03		Count	P_Value	Benjamini
INTERPRO	Tetratricopeptide-like helical	RT	17	8.4E-1	1.0E0
INTERPRO	Tetratricopeptide region	RT	13	8.6E-1	1.0E0
INTERPRO	Tetratricopeptide TPR-1	RT	10	9.3E-1	1.0E0
INTERPRO	Tetratricopeptide repeat	RT	11	9.7E-1	1.0E0
SP_PIR_KEYWORDS	tpr repeat	RT	4	9.7E-1	1.0E0
SMART	TPR	RT	11	9.8E-1	1.0E0
Annotation Cluster 76	Enrichment Score: 0.03		Count	P_Value	Benjamini
INTERPRO	WD40 repeat, subgroup	RT	30	8.9E-1	1.0E0
INTERPRO	WD40 repeat, conserved site	RT	15	9.1E-1	1.0E0
INTERPRO	WD40/YVTN repeat-like	RT	38	9.2E-1	1.0E0
INTERPRO	WD40 repeat	RT	39	9.2E-1	1.0E0
INTERPRO	WD40 repeat 2	RT	22	9.3E-1	1.0E0

	Category	Term		Count	P_Value	Benjamini
☐	SP_PIR_KEYWORDS	wd repeat	RT	25	9.5E-1	1.0E0
☐	SMART	WD40	RT	39	9.5E-1	1.0E0
☐	INTERPRO	WD40 repeat, region	RT	23	9.7E-1	1.0E0
	Annotation Cluster 77	Enrichment Score: 0.03		Count	P_Value	Benjamini
☐	GOTERM_MF_FAT	protein tyrosine phosphatase activity	RT	4	8.9E-1	1.0E0
☐	GOTERM_MF_FAT	phosphoprotein phosphatase activity	RT	14	9.3E-1	1.0E0
☐	GOTERM_BP_FAT	protein amino acid dephosphorylation	RT	6	9.5E-1	1.0E0
☐	GOTERM_BP_FAT	dephosphorylation	RT	7	9.7E-1	1.0E0
	Annotation Cluster 78	Enrichment Score: 0.03		Count	P_Value	Benjamini
☐	GOTERM_BP_FAT	macromolecular complex subunit organization	RT	15	7.8E-1	1.0E0
☐	GOTERM_BP_FAT	macromolecular complex assembly	RT	14	8.3E-1	1.0E0
☐	GOTERM_BP_FAT	cellular macromolecular complex subunit organization	RT	13	8.8E-1	1.0E0
☐	SP_PIR_KEYWORDS	nucleosome core	RT	4	9.0E-1	1.0E0
☐	GOTERM_BP_FAT	cellular macromolecular complex assembly	RT	12	9.1E-1	1.0E0
☐	INTERPRO	Histone-fold	RT	7	9.3E-1	1.0E0
☐	GOTERM_CC_FAT	nucleosome	RT	5	9.5E-1	1.0E0
☐	GOTERM_BP_FAT	DNA packaging	RT	5	9.6E-1	1.0E0
☐	GOTERM_BP_FAT	nucleosome assembly	RT	5	9.6E-1	1.0E0
☐	GOTERM_BP_FAT	nucleosome organization	RT	5	9.6E-1	1.0E0
☐	GOTERM_BP_FAT	protein-DNA complex assembly	RT	5	9.6E-1	1.0E0
☐	GOTERM_BP_FAT	chromatin assembly	RT	5	9.6E-1	1.0E0
☐	INTERPRO	Histone core	RT	5	9.6E-1	1.0E0
☐	SP_PIR_KEYWORDS	chromosomal protein	RT	5	9.7E-1	1.0E0
☐	GOTERM_CC_FAT	chromatin	RT	5	9.7E-1	1.0E0
☐	GOTERM_CC_FAT	chromosomal part	RT	7	9.8E-1	1.0E0
☐	GOTERM_BP_FAT	chromosome organization	RT	8	9.8E-1	1.0E0
☐	GOTERM_CC_FAT	protein-DNA complex	RT	5	9.8E-1	1.0E0
☐	GOTERM_BP_FAT	chromatin organization	RT	6	9.8E-1	1.0E0
☐	GOTERM_BP_FAT	chromatin assembly or disassembly	RT	5	9.9E-1	1.0E0
☐	GOTERM_CC_FAT	chromosome	RT	11	9.9E-1	1.0E0
	Annotation Cluster 79	Enrichment Score: 0.02		Count	P_Value	Benjamini
☐	INTERPRO	Protein phosphatase 2C-related	RT	3	9.5E-1	1.0E0
☐	INTERPRO	Protein phosphatase 2C	RT	3	9.5E-1	1.0E0
☐	INTERPRO	Protein phosphatase 2C, N-terminal	RT	3	9.5E-1	1.0E0
☐	SMART	PP2Cc	RT	3	9.6E-1	1.0E0
☐	SMART	PP2C_SIG	RT	3	9.6E-1	1.0E0
	Annotation Cluster 80	Enrichment Score: 0.01		Count	P_Value	Benjamini
☐	GOTERM_CC_FAT	prefoldin complex	RT	3	9.4E-1	1.0E0
☐	GOTERM_MF_FAT	unfolded protein binding	RT	9	9.8E-1	1.0E0
☐	GOTERM_CC_FAT	cytosolic part	RT	3	9.9E-1	1.0E0
	Annotation Cluster 81	Enrichment Score: 0.01		Count	P_Value	Benjamini
☐	INTERPRO	Like-Sm ribonucleoprotein, eukaryotic and archaea-type, core	RT	3	9.8E-1	1.0E0
☐	SMART	Sm	RT	3	9.8E-1	1.0E0
☐	INTERPRO	Like-Sm ribonucleoprotein, core	RT	4	9.9E-1	1.0E0
	Annotation Cluster 82	Enrichment Score: 0.01		Count	P_Value	Benjamini
☐	GOTERM_MF_FAT	nucleoside-triphosphatase regulator activity	RT	6	9.6E-1	1.0E0
☐	GOTERM_MF_FAT	GTPase regulator activity	RT	4	9.9E-1	1.0E0
☐	GOTERM_MF_FAT	small GTPase regulator activity	RT	4	9.9E-1	1.0E0
☐	GOTERM_BP_FAT	regulation of small GTPase mediated signal transduction	RT	3	9.9E-1	1.0E0
☐	GOTERM_BP_FAT	regulation of Ras protein signal transduction	RT	3	9.9E-1	1.0E0
	Annotation Cluster 83	Enrichment Score: 0		Count	P_Value	Benjamini
☐	SP_PIR_KEYWORDS	calcium	RT	5	1.0E0	1.0E0
☐	INTERPRO	EF-HAND 1	RT	7	1.0E0	1.0E0
☐	INTERPRO	EF-HAND 2	RT	5	1.0E0	1.0E0
☐	INTERPRO	EF-Hand type	RT	4	1.0E0	1.0E0

225 terms were not clustered.

Please cite *Nature Protocols 2009; 4(1):44 & Genome Biology 2003; 4(5):P3* within any publication that makes use of any methods inspired by **DAVID**.

Term of Service | Contact Us | Site Map

DAVID Bioinformatics Resources 6.7
National Institute of Allergy and Infectious Diseases (NIAID), NIH

Functional Annotation Clustering

Help and Manual

Current Gene List: 3_Random7
Current Background: Giardia intestinalis
3000 DAVID IDs

Options Classification Stringency Medium
Rerun using options | Create Sublist

79 Cluster(s) Download File

					Count	P_Value	Benjamini
	Annotation Cluster 1	Enrichment Score: 0.98	G		Count	P_Value	Benjamini
☐	GOTERM_MF_FAT	phospholipid binding	RT		22	3.4E-2	9.7E-1
☐	GOTERM_MF_FAT	lipid binding	RT		28	3.5E-2	9.4E-1
☐	GOTERM_MF_FAT	calcium-dependent phospholipid binding	RT		18	6.1E-2	9.9E-1
☐	INTERPRO	Annexin repeat	RT		12	9.8E-2	1.0E0
☐	GOTERM_MF_FAT	calcium ion binding	RT		31	1.0E-1	9.9E-1
☐	SMART	ANX	RT		12	1.0E-1	1.0E0
☐	INTERPRO	Annexin	RT		13	1.3E-1	1.0E0
☐	SP_PIR_KEYWORDS	annexin	RT		13	1.3E-1	1.0E0
☐	GOTERM_BP_FAT	cytoskeleton organization	RT		12	3.0E-1	1.0E0
☐	INTERPRO	Alpha giardin	RT		9	3.9E-1	1.0E0
	Annotation Cluster 2	Enrichment Score: 0.92	G		Count	P_Value	Benjamini
☐	GOTERM_BP_FAT	ncRNA processing	RT		23	4.4E-2	1.0E0
☐	GOTERM_BP_FAT	ncRNA metabolic process	RT		37	8.0E-2	1.0E0
☐	GOTERM_BP_FAT	RNA processing	RT		33	8.9E-2	1.0E0
☐	GOTERM_BP_FAT	ribonucleoprotein complex biogenesis	RT		16	1.1E-1	1.0E0
☐	GOTERM_BP_FAT	ribosome biogenesis	RT		16	1.1E-1	1.0E0
☐	GOTERM_BP_FAT	rRNA metabolic process	RT		11	1.5E-1	1.0E0
☐	GOTERM_BP_FAT	rRNA processing	RT		11	1.5E-1	1.0E0
☐	GOTERM_BP_FAT	tRNA processing	RT		13	2.4E-1	1.0E0
☐	GOTERM_BP_FAT	tRNA metabolic process	RT		27	2.7E-1	1.0E0
	Annotation Cluster 3	Enrichment Score: 0.9	G		Count	P_Value	Benjamini
☐	GOTERM_MF_FAT	metal ion binding	RT		167	1.6E-2	1.0E0
☐	GOTERM_MF_FAT	cation binding	RT		168	2.2E-2	9.9E-1
☐	GOTERM_MF_FAT	ion binding	RT		168	2.2E-2	9.9E-1
☐	GOTERM_MF_FAT	zinc ion binding	RT		99	7.8E-2	9.9E-1
☐	GOTERM_MF_FAT	transition metal ion binding	RT		121	8.0E-2	9.9E-1
☐	INTERPRO	Zinc finger, RING-type	RT		43	2.6E-1	1.0E0
☐	SMART	RING	RT		43	2.9E-1	1.0E0
☐	SP_PIR_KEYWORDS	zinc	RT		53	2.9E-1	1.0E0
☐	SP_PIR_KEYWORDS	metal-binding	RT		61	4.1E-1	1.0E0
☐	SP_PIR_KEYWORDS	zinc-finger	RT		38	4.1E-1	1.0E0
☐	INTERPRO	Zinc finger, C3HC4 RING-type	RT		21	6.2E-1	1.0E0
	Annotation Cluster 4	Enrichment Score: 0.78	G		Count	P_Value	Benjamini
☐	GOTERM_MF_FAT	helicase activity	RT		40	3.1E-2	9.9E-1
☐	INTERPRO	DNA/RNA helicase, C-terminal	RT		32	6.3E-2	1.0E0
☐	SP_PIR_KEYWORDS	helicase	RT		32	6.6E-2	1.0E0
☐	SMART	HELICc	RT		32	6.9E-2	1.0E0
☐	INTERPRO	Helicase, superfamily 1 and 2, ATP-binding	RT		31	7.9E-2	1.0E0
☐	INTERPRO	DNA/RNA helicase, DEAD/DEAH box type, N-terminal	RT		22	1.3E-1	1.0E0
☐	INTERPRO	DEAD-like helicase, N-terminal	RT		30	1.4E-1	1.0E0
☐	SMART	DEXDc	RT		30	1.5E-1	1.0E0
☐	GOTERM_MF_FAT	ATP-dependent helicase activity	RT		26	2.5E-1	1.0E0
☐	GOTERM_MF_FAT	purine NTP-dependent helicase activity	RT		26	2.5E-1	1.0E0
☐	INTERPRO	RNA helicase, DEAD-box type, Q motif	RT		10	7.5E-1	1.0E0
☐	GOTERM_MF_FAT	ATPase activity, coupled	RT		39	8.4E-1	1.0E0

GOTERM_MF_FAT	ATPase activity	RT	50	9.4E-1	1.0E0
Annotation Cluster 5	Enrichment Score: 0.58		Count	P_Value	Benjamini
INTERPRO	Serine/threonine protein kinase	RT	40	9.9E-2	1.0E0
SMART	S_TKc	RT	40	1.1E-1	9.9E-1
GOTERM_MF_FAT	protein serine/threonine kinase activity	RT	68	1.5E-1	1.0E0
INTERPRO	Serine/threonine protein kinase, active site	RT	56	2.8E-1	1.0E0
SP_PIR_KEYWORDS	serine/threonine-protein kinase	RT	34	7.9E-1	9.9E-1
INTERPRO	Protein kinase, ATP binding site	RT	42	9.5E-1	1.0E0
Annotation Cluster 6	Enrichment Score: 0.53		Count	P_Value	Benjamini
INTERPRO	Thioredoxin fold	RT	17	1.2E-1	1.0E0
INTERPRO	Thioredoxin-like	RT	11	1.4E-1	1.0E0
GOTERM_BP_FAT	cell redox homeostasis	RT	14	1.8E-1	1.0E0
INTERPRO	Alkyl hydroperoxide reductase/ Thiol specific antioxidant/ Mal allergen	RT	6	2.1E-1	1.0E0
GOTERM_BP_FAT	cellular homeostasis	RT	14	2.8E-1	1.0E0
GOTERM_BP_FAT	homeostatic process	RT	14	3.8E-1	1.0E0
INTERPRO	Thioredoxin, core	RT	6	3.8E-1	1.0E0
GOTERM_MF_FAT	antioxidant activity	RT	6	4.0E-1	1.0E0
INTERPRO	Thioredoxin domain	RT	5	5.1E-1	1.0E0
INTERPRO	Thioredoxin, conserved site	RT	5	5.1E-1	1.0E0
INTERPRO	Thioredoxin-like subdomain	RT	4	6.5E-1	1.0E0
Annotation Cluster 7	Enrichment Score: 0.49		Count	P_Value	Benjamini
INTERPRO	EGF-like, type 3	RT	23	1.5E-1	1.0E0
INTERPRO	EGF, extracellular	RT	16	3.2E-1	1.0E0
INTERPRO	EGF	RT	6	3.8E-1	1.0E0
SP_PIR_KEYWORDS	egf-like domain	RT	11	5.9E-1	9.9E-1
Annotation Cluster 8	Enrichment Score: 0.45		Count	P_Value	Benjamini
GOTERM_BP_FAT	vesicle-mediated transport	RT	21	1.8E-1	1.0E0
GOTERM_BP_FAT	intracellular transport	RT	20	2.2E-1	1.0E0
GOTERM_CC_FAT	coated membrane	RT	11	2.4E-1	1.0E0
GOTERM_CC_FAT	membrane coat	RT	11	2.4E-1	1.0E0
GOTERM_CC_FAT	endomembrane system	RT	12	2.9E-1	1.0E0
GOTERM_BP_FAT	cellular protein localization	RT	16	3.5E-1	1.0E0
GOTERM_BP_FAT	intracellular protein transport	RT	16	3.5E-1	1.0E0
GOTERM_BP_FAT	cellular macromolecule localization	RT	16	3.5E-1	1.0E0
GOTERM_CC_FAT	Golgi apparatus	RT	8	5.0E-1	1.0E0
GOTERM_BP_FAT	establishment of protein localization	RT	20	6.4E-1	1.0E0
GOTERM_BP_FAT	protein transport	RT	20	6.4E-1	1.0E0
GOTERM_BP_FAT	protein localization	RT	20	7.1E-1	1.0E0
Annotation Cluster 9	Enrichment Score: 0.44		Count	P_Value	Benjamini
INTERPRO	SET	RT	6	2.1E-1	1.0E0
SMART	SET	RT	6	2.1E-1	1.0E0
INTERPRO	Post-SET zinc-binding region	RT	3	6.2E-1	1.0E0
SMART	PostSET	RT	3	6.3E-1	1.0E0
Annotation Cluster 10	Enrichment Score: 0.42		Count	P_Value	Benjamini
INTERPRO	EGF-like, type 3	RT	23	1.5E-1	1.0E0
INTERPRO	EGF-like, laminin	RT	16	6.0E-1	1.0E0
SMART	EGF_Lam	RT	16	6.2E-1	1.0E0
Annotation Cluster 11	Enrichment Score: 0.37		Count	P_Value	Benjamini
GOTERM_BP_FAT	chromosome organization	RT	15	1.4E-1	1.0E0
GOTERM_CC_FAT	chromosome	RT	20	1.4E-1	1.0E0
GOTERM_CC_FAT	chromosomal part	RT	13	2.2E-1	1.0E0
INTERPRO	Histone-fold	RT	11	2.3E-1	1.0E0
GOTERM_CC_FAT	protein-DNA complex	RT	10	3.2E-1	1.0E0
GOTERM_BP_FAT	chromatin assembly or disassembly	RT	10	3.2E-1	1.0E0
GOTERM_BP_FAT	chromatin organization	RT	11	3.7E-1	1.0E0
SP_PIR_KEYWORDS	nucleosome core	RT	6	3.8E-1	1.0E0
GOTERM_CC_FAT	chromatin	RT	9	4.0E-1	1.0E0
GOTERM_BP_FAT	cellular macromolecular complex assembly	RT	16	4.5E-1	1.0E0

INTERPRO	Histone core	RT	8	4.8E-1	1.0E0
SP_PIR_KEYWORDS	chromosomal protein	RT	8	4.9E-1	1.0E0
SP_PIR_KEYWORDS	dna-binding	RT	22	4.9E-1	1.0E0
GOTERM_CC_FAT	nucleosome	RT	8	5.0E-1	1.0E0
GOTERM_BP_FAT	chromatin assembly	RT	8	5.1E-1	1.0E0
GOTERM_BP_FAT	protein-DNA complex assembly	RT	8	5.1E-1	1.0E0
GOTERM_BP_FAT	nucleosome organization	RT	8	5.1E-1	1.0E0
GOTERM_BP_FAT	nucleosome assembly	RT	8	5.1E-1	1.0E0
GOTERM_BP_FAT	DNA packaging	RT	8	5.1E-1	1.0E0
GOTERM_BP_FAT	cellular macromolecular complex subunit organization	RT	16	5.5E-1	1.0E0
PIR_SUPERFAMILY	PIRSF002052:histone H4	RT	3	5.9E-1	1.0E0
INTERPRO	Histone H4	RT	3	6.2E-1	1.0E0
SMART	H4	RT	3	6.3E-1	1.0E0
GOTERM_BP_FAT	macromolecular complex assembly	RT	16	6.4E-1	1.0E0
SP_PIR_KEYWORDS	nucleus	RT	20	6.8E-1	9.9E-1
GOTERM_BP_FAT	macromolecular complex subunit organization	RT	16	7.2E-1	1.0E0
Annotation Cluster 12	Enrichment Score: 0.35		Count	P_Value	Benjamini
INTERPRO	Ubiquitin supergroup	RT	10	1.9E-1	1.0E0
INTERPRO	Ubiquitin	RT	5	6.7E-1	1.0E0
SMART	UBQ	RT	5	6.8E-1	1.0E0
Annotation Cluster 13	Enrichment Score: 0.35		Count	P_Value	Benjamini
GOTERM_CC_FAT	cytosolic part	RT	8	2.1E-1	1.0E0
GOTERM_CC_FAT	prefoldin complex	RT	6	2.1E-1	1.0E0
INTERPRO	Prefoldin alpha-like	RT	3	6.2E-1	1.0E0
INTERPRO	Prefoldin beta-like	RT	3	6.2E-1	1.0E0
GOTERM_MF_FAT	unfolded protein binding	RT	13	6.7E-1	1.0E0
GOTERM_BP_FAT	protein folding	RT	18	6.8E-1	1.0E0
Annotation Cluster 14	Enrichment Score: 0.34		Count	P_Value	Benjamini
GOTERM_MF_FAT	protein serine/threonine kinase activity	RT	68	1.5E-1	1.0E0
INTERPRO	Protein kinase, core	RT	162	2.0E-1	1.0E0
GOTERM_BP_FAT	protein amino acid phosphorylation	RT	164	2.4E-1	1.0E0
INTERPRO	Serine/threonine protein kinase, active site	RT	56	2.8E-1	1.0E0
GOTERM_MF_FAT	protein kinase activity	RT	165	2.8E-1	1.0E0
GOTERM_BP_FAT	phosphorylation	RT	174	3.0E-1	1.0E0
SP_PIR_KEYWORDS	kinase	RT	197	3.0E-1	1.0E0
GOTERM_BP_FAT	phosphate metabolic process	RT	183	3.2E-1	1.0E0
GOTERM_BP_FAT	phosphorus metabolic process	RT	183	3.2E-1	1.0E0
INTERPRO	Serine/threonine protein kinase-related	RT	139	3.6E-1	1.0E0
GOTERM_MF_FAT	adenyl nucleotide binding	RT	311	6.6E-1	1.0E0
GOTERM_MF_FAT	purine nucleoside binding	RT	311	6.6E-1	1.0E0
GOTERM_MF_FAT	ATP binding	RT	306	6.9E-1	1.0E0
GOTERM_MF_FAT	adenyl ribonucleotide binding	RT	306	6.9E-1	1.0E0
GOTERM_MF_FAT	nucleoside binding	RT	312	7.1E-1	1.0E0
GOTERM_MF_FAT	nucleotide binding	RT	359	7.2E-1	1.0E0
GOTERM_MF_FAT	purine nucleotide binding	RT	337	7.5E-1	1.0E0
GOTERM_MF_FAT	purine ribonucleotide binding	RT	332	7.8E-1	1.0E0
GOTERM_MF_FAT	ribonucleotide binding	RT	332	7.8E-1	1.0E0
SP_PIR_KEYWORDS	atp-binding	RT	141	9.1E-1	1.0E0
SP_PIR_KEYWORDS	nucleotide-binding	RT	141	9.9E-1	1.0E0
Annotation Cluster 15	Enrichment Score: 0.32		Count	P_Value	Benjamini
INTERPRO	Peptidase M14, carboxypeptidase A	RT	5	3.1E-1	1.0E0
SMART	Zn_pept	RT	5	3.1E-1	1.0E0
GOTERM_MF_FAT	metalloexopeptidase activity	RT	6	4.0E-1	1.0E0
GOTERM_MF_FAT	metallocarboxypeptidase activity	RT	4	4.6E-1	1.0E0
GOTERM_MF_FAT	carboxypeptidase activity	RT	4	4.6E-1	1.0E0
GOTERM_MF_FAT	exopeptidase activity	RT	7	7.4E-1	1.0E0
GOTERM_MF_FAT	metallopeptidase activity	RT	7	8.9E-1	1.0E0

Category	Term		Count	P_Value	Benjamini
Annotation Cluster 16	Enrichment Score: 0.31		Count	P_Value	Benjamini
INTERPRO	Alkyl hydroperoxide reductase/ Thiol specific antioxidant/ Mal allergen	RT	6	2.1E-1	1.0E0
GOTERM_MF_FAT	antioxidant activity	RT	6	4.0E-1	1.0E0
INTERPRO	Peroxiredoxin, C-terminal	RT	3	6.2E-1	1.0E0
SP_PIR_KEYWORDS	peroxidase	RT	3	6.2E-1	9.9E-1
GOTERM_MF_FAT	peroxidase activity	RT	3	6.4E-1	1.0E0
GOTERM_MF_FAT	oxidoreductase activity, acting on peroxide as acceptor	RT	3	6.4E-1	1.0E0
Annotation Cluster 17	Enrichment Score: 0.3		Count	P_Value	Benjamini
INTERPRO	RNA recognition motif, RNP-1	RT	10	4.3E-1	1.0E0
SMART	RRM	RT	10	4.4E-1	1.0E0
INTERPRO	Nucleotide-binding, alpha-beta plait	RT	10	6.6E-1	1.0E0
Annotation Cluster 18	Enrichment Score: 0.3		Count	P_Value	Benjamini
GOTERM_CC_FAT	coated membrane	RT	11	2.4E-1	1.0E0
GOTERM_CC_FAT	membrane coat	RT	11	2.4E-1	1.0E0
GOTERM_CC_FAT	Golgi apparatus part	RT	7	2.9E-1	1.0E0
GOTERM_CC_FAT	endomembrane system	RT	12	2.9E-1	1.0E0
GOTERM_CC_FAT	Golgi apparatus	RT	8	5.0E-1	1.0E0
GOTERM_CC_FAT	organelle membrane	RT	8	5.0E-1	1.0E0
GOTERM_CC_FAT	cytoplasmic membrane-bounded vesicle	RT	5	5.2E-1	1.0E0
GOTERM_CC_FAT	cytoplasmic vesicle part	RT	5	5.2E-1	1.0E0
GOTERM_CC_FAT	vesicle membrane	RT	5	5.2E-1	1.0E0
GOTERM_CC_FAT	coated vesicle membrane	RT	5	5.2E-1	1.0E0
GOTERM_CC_FAT	cytoplasmic vesicle membrane	RT	5	5.2E-1	1.0E0
GOTERM_CC_FAT	coated vesicle	RT	5	5.2E-1	1.0E0
GOTERM_CC_FAT	vesicle coat	RT	5	5.2E-1	1.0E0
GOTERM_CC_FAT	vesicle	RT	5	5.2E-1	1.0E0
GOTERM_CC_FAT	membrane-bounded vesicle	RT	5	5.2E-1	1.0E0
GOTERM_CC_FAT	cytoplasmic vesicle	RT	5	5.2E-1	1.0E0
GOTERM_BP_FAT	ER to Golgi vesicle-mediated transport	RT	5	5.2E-1	1.0E0
GOTERM_BP_FAT	Golgi vesicle transport	RT	5	5.2E-1	1.0E0
INTERPRO	Clathrin/coatomer adaptor, adaptin-like, N-terminal	RT	4	6.5E-1	1.0E0
GOTERM_CC_FAT	transport vesicle membrane	RT	4	6.6E-1	1.0E0
GOTERM_CC_FAT	transport vesicle	RT	4	6.6E-1	1.0E0
GOTERM_CC_FAT	ER to Golgi transport vesicle membrane	RT	3	8.1E-1	1.0E0
GOTERM_CC_FAT	ER to Golgi transport vesicle	RT	3	8.1E-1	1.0E0
GOTERM_CC_FAT	COPII vesicle coat	RT	3	8.1E-1	1.0E0
Annotation Cluster 19	Enrichment Score: 0.3		Count	P_Value	Benjamini
INTERPRO	EGF-like	RT	116	2.7E-1	1.0E0
SMART	EGF	RT	116	3.1E-1	1.0E0
INTERPRO	Giardia variant-specific surface protein	RT	139	4.9E-1	1.0E0
INTERPRO	Furin-like repeat	RT	101	8.8E-1	1.0E0
SMART	FU	RT	101	9.1E-1	1.0E0
Annotation Cluster 20	Enrichment Score: 0.28		Count	P_Value	Benjamini
GOTERM_MF_FAT	translation factor activity, nucleic acid binding	RT	21	4.2E-1	1.0E0
SP_PIR_KEYWORDS	Initiation factor	RT	13	4.3E-1	1.0E0
GOTERM_MF_FAT	translation initiation factor activity	RT	16	5.5E-1	1.0E0
GOTERM_BP_FAT	translational initiation	RT	7	7.3E-1	1.0E0
Annotation Cluster 21	Enrichment Score: 0.28		Count	P_Value	Benjamini
GOTERM_MF_FAT	phosphatase activity	RT	29	3.3E-1	1.0E0
GOTERM_MF_FAT	iron ion binding	RT	21	3.4E-1	1.0E0
GOTERM_MF_FAT	phosphoprotein phosphatase activity	RT	18	5.2E-1	1.0E0
SP_PIR_KEYWORDS	iron	RT	13	5.4E-1	1.0E0
INTERPRO	Serine/threonine-specific protein phosphatase and bis(5-nucleosyl)-tetraphosphatase	RT	10	5.5E-1	1.0E0
SMART	PP2Ac	RT	10	5.7E-1	1.0E0
PIR_SUPERFAMILY	PIRSF000908:serine/threonine-protein phosphatase, conventional type	RT	5	7.5E-1	1.0E0
INTERPRO	Metallophosphoesterase	RT	12	7.8E-1	1.0E0
Annotation Cluster 22	Enrichment Score: 0.28		Count	P_Value	Benjamini

KEGG_PATHWAY	Ribosome	RT	41	2.1E-1	1.0E0
GOTERM_CC_FAT	ribonucleoprotein complex	RT	61	3.7E-1	1.0E0
GOTERM_CC_FAT	non-membrane-bounded organelle	RT	94	5.3E-1	1.0E0
GOTERM_CC_FAT	intracellular non-membrane-bounded organelle	RT	94	5.3E-1	1.0E0
SP_PIR_KEYWORDS	ribosomal protein	RT	43	5.5E-1	1.0E0
GOTERM_MF_FAT	structural molecule activity	RT	49	6.5E-1	1.0E0
GOTERM_CC_FAT	ribosome	RT	44	6.6E-1	1.0E0
GOTERM_BP_FAT	translation	RT	65	7.0E-1	1.0E0
GOTERM_MF_FAT	structural constituent of ribosome	RT	39	8.0E-1	1.0E0
Annotation Cluster 23	Enrichment Score: 0.27		Count	P_Value	Benjamini
KEGG_PATHWAY	Terpenoid backbone biosynthesis	RT	5	4.2E-1	1.0E0
GOTERM_BP_FAT	lipid biosynthetic process	RT	10	4.5E-1	1.0E0
GOTERM_BP_FAT	isoprenoid metabolic process	RT	4	6.7E-1	1.0E0
GOTERM_BP_FAT	isoprenoid biosynthetic process	RT	4	6.7E-1	1.0E0
Annotation Cluster 24	Enrichment Score: 0.26		Count	P_Value	Benjamini
INTERPRO	Calcium-binding EF-hand	RT	8	4.8E-1	1.0E0
INTERPRO	EF hand	RT	8	4.8E-1	1.0E0
SMART	EFh	RT	8	4.9E-1	1.0E0
INTERPRO	EF-HAND 1	RT	13	5.3E-1	1.0E0
SP_PIR_KEYWORDS	calcium	RT	10	5.6E-1	9.9E-1
INTERPRO	EF-HAND 2	RT	11	5.8E-1	1.0E0
INTERPRO	EF-Hand type	RT	10	8.3E-1	1.0E0
Annotation Cluster 25	Enrichment Score: 0.25		Count	P_Value	Benjamini
GOTERM_BP_FAT	ncRNA metabolic process	RT	37	8.0E-2	1.0E0
GOTERM_BP_FAT	tRNA metabolic process	RT	27	2.7E-1	1.0E0
INTERPRO	Anticodon-binding	RT	4	4.4E-1	1.0E0
KEGG_PATHWAY	Aminoacyl-tRNA biosynthesis	RT	12	6.4E-1	1.0E0
INTERPRO	Aminoacyl-tRNA synthetase, class II (G, H, P and S), conserved region	RT	4	6.5E-1	1.0E0
GOTERM_BP_FAT	amino acid activation	RT	14	6.8E-1	1.0E0
GOTERM_BP_FAT	tRNA aminoacylation	RT	14	6.8E-1	1.0E0
INTERPRO	Aminoacyl-tRNA synthetase, class II, conserved region	RT	6	6.9E-1	1.0E0
GOTERM_BP_FAT	tRNA aminoacylation for protein translation	RT	13	7.4E-1	1.0E0
GOTERM_MF_FAT	ligase activity, forming carbon-oxygen bonds	RT	14	7.6E-1	1.0E0
GOTERM_MF_FAT	ligase activity, forming aminoacyl-tRNA and related compounds	RT	14	7.6E-1	1.0E0
GOTERM_MF_FAT	aminoacyl-tRNA ligase activity	RT	14	7.6E-1	1.0E0
SP_PIR_KEYWORDS	Aminoacyl-tRNA synthetase	RT	13	7.9E-1	9.9E-1
SP_PIR_KEYWORDS	ligase	RT	30	8.4E-1	9.9E-1
Annotation Cluster 26	Enrichment Score: 0.24		Count	P_Value	Benjamini
INTERPRO	Protein phosphatase 2C, manganese/magnesium aspartate binding site	RT	4	4.4E-1	1.0E0
SP_PIR_KEYWORDS	protein phosphatase	RT	4	4.5E-1	1.0E0
GOTERM_MF_FAT	protein serine/threonine phosphatase activity	RT	4	4.6E-1	1.0E0
INTERPRO	Protein phosphatase 2C, N-terminal	RT	5	5.1E-1	1.0E0
INTERPRO	Protein phosphatase 2C-related	RT	5	5.1E-1	1.0E0
INTERPRO	Protein phosphatase 2C	RT	5	5.1E-1	1.0E0
SMART	PP2Cc	RT	5	5.2E-1	1.0E0
SMART	PP2C_SIG	RT	5	5.2E-1	1.0E0
GOTERM_CC_FAT	protein serine/threonine phosphatase complex	RT	5	6.9E-1	1.0E0
GOTERM_BP_FAT	dephosphorylation	RT	10	6.9E-1	1.0E0
GOTERM_BP_FAT	protein amino acid dephosphorylation	RT	8	7.5E-1	1.0E0
GOTERM_MF_FAT	magnesium ion binding	RT	9	7.7E-1	1.0E0
SP_PIR_KEYWORDS	magnesium	RT	7	8.1E-1	9.9E-1
Annotation Cluster 27	Enrichment Score: 0.23		Count	P_Value	Benjamini
GOTERM_BP_FAT	methylation	RT	8	3.6E-1	1.0E0
INTERPRO	Ribosomal RNA methyltransferase RrmJ/FtsJ	RT	4	4.4E-1	1.0E0
GOTERM_BP_FAT	one-carbon metabolic process	RT	8	5.1E-1	1.0E0

GOTERM_BP_FAT	RNA methylation	RT	4	6.7E-1	1.0E0
GOTERM_BP_FAT	biopolymer methylation	RT	4	8.1E-1	1.0E0
GOTERM_BP_FAT	RNA modification	RT	8	9.3E-1	1.0E0
Annotation Cluster 28	Enrichment Score: 0.23		Count	P_Value	Benjamini
GOTERM_BP_FAT	translational elongation	RT	6	4.0E-1	1.0E0
GOTERM_MF_FAT	translation elongation factor activity	RT	6	5.8E-1	1.0E0
SP_PIR_KEYWORDS	elongation factor	RT	5	6.8E-1	9.9E-1
INTERPRO	Protein synthesis factor, GTP-binding	RT	5	8.0E-1	1.0E0
Annotation Cluster 29	Enrichment Score: 0.22		Count	P_Value	Benjamini
INTERPRO	Zinc finger, C2H2-like	RT	12	5.0E-1	1.0E0
SMART	ZnF_C2H2	RT	12	5.1E-1	1.0E0
INTERPRO	Zinc finger, C2H2-type	RT	10	8.3E-1	1.0E0
Annotation Cluster 30	Enrichment Score: 0.21		Count	P_Value	Benjamini
INTERPRO	ATPase, AAA-type, core	RT	16	5.1E-1	1.0E0
INTERPRO	ATPase, AAA+ type, core	RT	41	6.7E-1	1.0E0
SMART	AAA	RT	41	7.0E-1	1.0E0
Annotation Cluster 31	Enrichment Score: 0.2		Count	P_Value	Benjamini
GOTERM_MF_FAT	enzyme activator activity	RT	7	4.6E-1	1.0E0
GOTERM_MF_FAT	nucleoside-triphosphatase regulator activity	RT	9	5.5E-1	1.0E0
GOTERM_BP_FAT	regulation of GTPase activity	RT	6	5.7E-1	1.0E0
GOTERM_BP_FAT	regulation of hydrolase activity	RT	6	5.7E-1	1.0E0
INTERPRO	Arf GTPase activating protein	RT	3	6.2E-1	1.0E0
SMART	ArfGap	RT	3	6.3E-1	1.0E0
GOTERM_BP_FAT	regulation of ARF GTPase activity	RT	3	6.3E-1	1.0E0
GOTERM_MF_FAT	ARF GTPase activator activity	RT	3	6.4E-1	1.0E0
GOTERM_BP_FAT	regulation of ARF protein signal transduction	RT	4	6.7E-1	1.0E0
GOTERM_BP_FAT	regulation of Ras GTPase activity	RT	5	6.9E-1	1.0E0
GOTERM_MF_FAT	GTPase activator activity	RT	5	7.0E-1	1.0E0
GOTERM_BP_FAT	regulation of Ras protein signal transduction	RT	6	7.1E-1	1.0E0
GOTERM_BP_FAT	regulation of small GTPase mediated signal transduction	RT	6	7.1E-1	1.0E0
GOTERM_MF_FAT	small GTPase regulator activity	RT	7	7.4E-1	1.0E0
GOTERM_MF_FAT	GTPase regulator activity	RT	7	7.4E-1	1.0E0
Annotation Cluster 32	Enrichment Score: 0.19		Count	P_Value	Benjamini
GOTERM_MF_FAT	RNA polymerase activity	RT	16	4.6E-1	1.0E0
GOTERM_MF_FAT	nucleotidyltransferase activity	RT	44	5.2E-1	1.0E0
GOTERM_MF_FAT	DNA-directed RNA polymerase activity	RT	15	5.3E-1	1.0E0
KEGG_PATHWAY	Pyrimidine metabolism	RT	19	5.4E-1	1.0E0
KEGG_PATHWAY	RNA polymerase	RT	10	7.2E-1	1.0E0
KEGG_PATHWAY	Purine metabolism	RT	17	7.4E-1	1.0E0
SP_PIR_KEYWORDS	nucleotidyltransferase	RT	14	8.0E-1	9.9E-1
SP_PIR_KEYWORDS	dna-directed rna polymerase	RT	8	8.2E-1	9.9E-1
GOTERM_BP_FAT	transcription	RT	14	8.7E-1	1.0E0
Annotation Cluster 33	Enrichment Score: 0.18		Count	P_Value	Benjamini
INTERPRO	Phox-like	RT	4	6.5E-1	1.0E0
SMART	PX	RT	4	6.6E-1	1.0E0
GOTERM_MF_FAT	phosphoinositide binding	RT	4	6.7E-1	1.0E0
Annotation Cluster 34	Enrichment Score: 0.18		Count	P_Value	Benjamini
INTERPRO	Cyclin, C-terminal	RT	4	4.4E-1	1.0E0
INTERPRO	Cyclin, N-terminal	RT	5	5.1E-1	1.0E0
SP_PIR_KEYWORDS	cyclin	RT	7	5.9E-1	9.9E-1
INTERPRO	Cyclin-related	RT	4	7.9E-1	1.0E0
INTERPRO	Cyclin	RT	3	9.0E-1	1.0E0
SMART	CYCLIN	RT	3	9.0E-1	1.0E0
Annotation Cluster 35	Enrichment Score: 0.18		Count	P_Value	Benjamini
PIR_SUPERFAMILY	PIRSF006621:Dus	RT	3	5.9E-1	1.0E0
INTERPRO	tRNA-dihydrouridine synthase	RT	3	6.2E-1	1.0E0
GOTERM_MF_FAT	tRNA dihydrouridine synthase activity	RT	3	6.4E-1	1.0E0
INTERPRO	Aldolase-type TIM barrel	RT	6	6.9E-1	1.0E0

GOTERM_MF_FAT	FAD binding	RT		4	8.1E-1	1.0E0
Annotation Cluster 36	Enrichment Score: 0.17	G		Count	P_Value	Benjamini
INTERPRO	Copine	RT		4	6.5E-1	1.0E0
INTERPRO	von Willebrand factor, type A	RT		5	6.7E-1	1.0E0
SMART	VWA	RT		5	6.8E-1	1.0E0
Annotation Cluster 37	Enrichment Score: 0.16	G		Count	P_Value	Benjamini
INTERPRO	Like-Sm ribonucleoprotein, eukaryotic and archaea-type, core	RT		5	6.7E-1	1.0E0
SMART	Sm	RT		5	6.8E-1	1.0E0
INTERPRO	Like-Sm ribonucleoprotein, core	RT		7	7.1E-1	1.0E0
Annotation Cluster 38	Enrichment Score: 0.16	G		Count	P_Value	Benjamini
GOTERM_MF_FAT	hydrolase activity, acting on carbon-nitrogen (but not peptide) bonds, in linear amides	RT		7	3.0E-1	1.0E0
GOTERM_BP_FAT	protein amino acid deacetylation	RT		5	5.2E-1	1.0E0
INTERPRO	NAD-dependent histone deacetylase, silent information regulator Sir2	RT		4	6.5E-1	1.0E0
GOTERM_BP_FAT	negative regulation of macromolecule biosynthetic process	RT		4	6.7E-1	1.0E0
GOTERM_BP_FAT	negative regulation of nucleobase, nucleoside, nucleotide and nucleic acid metabolic process	RT		4	6.7E-1	1.0E0
GOTERM_BP_FAT	negative regulation of biosynthetic process	RT		4	6.7E-1	1.0E0
GOTERM_BP_FAT	negative regulation of transcription, DNA-dependent	RT		4	6.7E-1	1.0E0
GOTERM_BP_FAT	chromatin silencing	RT		4	6.7E-1	1.0E0
GOTERM_BP_FAT	negative regulation of cellular biosynthetic process	RT		4	6.7E-1	1.0E0
GOTERM_BP_FAT	negative regulation of gene expression, epigenetic	RT		4	6.7E-1	1.0E0
GOTERM_BP_FAT	negative regulation of RNA metabolic process	RT		4	6.7E-1	1.0E0
GOTERM_BP_FAT	regulation of gene expression, epigenetic	RT		4	6.7E-1	1.0E0
GOTERM_BP_FAT	negative regulation of nitrogen compound metabolic process	RT		4	6.7E-1	1.0E0
GOTERM_BP_FAT	negative regulation of transcription	RT		4	6.7E-1	1.0E0
GOTERM_MF_FAT	NAD binding	RT		4	6.7E-1	1.0E0
GOTERM_BP_FAT	negative regulation of macromolecule metabolic process	RT		4	8.9E-1	1.0E0
GOTERM_BP_FAT	negative regulation of gene expression	RT		4	8.9E-1	1.0E0
GOTERM_BP_FAT	gene silencing	RT		4	8.9E-1	1.0E0
GOTERM_BP_FAT	regulation of transcription, DNA-dependent	RT		6	9.4E-1	1.0E0
GOTERM_BP_FAT	regulation of RNA metabolic process	RT		6	9.4E-1	1.0E0
GOTERM_MF_FAT	NAD or NADH binding	RT		4	9.5E-1	1.0E0
Annotation Cluster 39	Enrichment Score: 0.16	G		Count	P_Value	Benjamini
INTERPRO	Ankyrin	RT		251	6.2E-1	1.0E0
SMART	ANK	RT		251	7.1E-1	1.0E0
SP_PIR_KEYWORDS	ank repeat	RT		174	7.6E-1	9.9E-1
Annotation Cluster 40	Enrichment Score: 0.16	G		Count	P_Value	Benjamini
GOTERM_BP_FAT	lipid biosynthetic process	RT		10	4.5E-1	1.0E0
GOTERM_MF_FAT	phosphotransferase activity, for other substituted phosphate groups	RT		4	4.6E-1	1.0E0
INTERPRO	CDP-alcohol phosphatidyltransferase	RT		3	6.2E-1	1.0E0
GOTERM_BP_FAT	phospholipid metabolic process	RT		8	6.4E-1	1.0E0
GOTERM_BP_FAT	phospholipid biosynthetic process	RT		5	6.9E-1	1.0E0
GOTERM_BP_FAT	organophosphate metabolic process	RT		8	7.5E-1	1.0E0
GOTERM_BP_FAT	phosphoinositide metabolic process	RT		4	8.9E-1	1.0E0
GOTERM_BP_FAT	glycerophospholipid metabolic process	RT		4	8.9E-1	1.0E0
GOTERM_BP_FAT	glycerolipid metabolic process	RT		4	8.9E-1	1.0E0
GOTERM_MF_FAT	inositol or phosphatidylinositol kinase activity	RT		3	9.1E-1	1.0E0
Annotation Cluster 41	Enrichment Score: 0.15	G		Count	P_Value	Benjamini
GOTERM_BP_FAT	cellular amino acid biosynthetic process	RT		4	6.7E-1	1.0E0
GOTERM_BP_FAT	organic acid biosynthetic process	RT		5	6.9E-1	1.0E0
GOTERM_BP_FAT	carboxylic acid biosynthetic process	RT		5	6.9E-1	1.0E0
GOTERM_BP_FAT	amine biosynthetic process	RT		4	8.1E-1	1.0E0
Annotation Cluster 42	Enrichment Score: 0.15	G		Count	P_Value	Benjamini
COG_ONTOLOGY	Defense mechanisms	RT		14	5.8E-1	1.0E0
INTERPRO	ABC transporter, conserved site	RT		13	7.2E-1	1.0E0
	ABC transporter-like	RT		12	8.9E-1	1.0E0

Category	Term	RT	Count	P_Value	Benjamini
INTERPRO					
Annotation Cluster 43	Enrichment Score: 0.14		Count	P_Value	Benjamini
INTERPRO	ADP-ribosylation factor	RT	4	4.4E-1	1.0E0
SMART	ARF	RT	4	4.5E-1	1.0E0
PIR_SUPERFAMILY	PIRSF001711:ADP-ribosylation factor	RT	4	6.1E-1	1.0E0
INTERPRO	ARF/SAR superfamily	RT	5	6.7E-1	1.0E0
PIR_SUPERFAMILY	PIRSF001710:Ras-related protein Rab	RT	4	7.6E-1	1.0E0
GOTERM_BP_FAT	intracellular signaling cascade	RT	12	8.0E-1	1.0E0
GOTERM_BP_FAT	small GTPase mediated signal transduction	RT	9	8.4E-1	1.0E0
INTERPRO	Ras GTPase	RT	6	9.6E-1	1.0E0
INTERPRO	Small GTP-binding protein	RT	9	9.7E-1	1.0E0
INTERPRO	Ras	RT	5	9.8E-1	1.0E0
Annotation Cluster 44	Enrichment Score: 0.14		Count	P_Value	Benjamini
GOTERM_BP_FAT	DNA metabolic process	RT	35	6.4E-1	1.0E0
GOTERM_BP_FAT	response to DNA damage stimulus	RT	12	7.3E-1	1.0E0
GOTERM_BP_FAT	DNA repair	RT	12	7.3E-1	1.0E0
GOTERM_BP_FAT	cellular response to stress	RT	12	8.0E-1	1.0E0
Annotation Cluster 45	Enrichment Score: 0.14		Count	P_Value	Benjamini
INTERPRO	Multi antimicrobial extrusion protein MatE	RT	4	6.5E-1	1.0E0
GOTERM_BP_FAT	multidrug transport	RT	4	6.7E-1	1.0E0
GOTERM_BP_FAT	drug transport	RT	4	6.7E-1	1.0E0
GOTERM_BP_FAT	response to drug	RT	4	6.7E-1	1.0E0
GOTERM_MF_FAT	drug transporter activity	RT	4	6.7E-1	1.0E0
INTERPRO	Na+ driven multidrug efflux pump	RT	3	8.0E-1	1.0E0
GOTERM_MF_FAT	antiporter activity	RT	4	8.1E-1	1.0E0
GOTERM_BP_FAT	transmembrane transport	RT	11	9.0E-1	1.0E0
Annotation Cluster 46	Enrichment Score: 0.14		Count	P_Value	Benjamini
INTERPRO	Kinesin, motor region, conserved site	RT	12	5.0E-1	1.0E0
SP_PIR_KEYWORDS	microtubule	RT	20	5.3E-1	1.0E0
SP_PIR_KEYWORDS	motor protein	RT	15	5.9E-1	9.9E-1
INTERPRO	Kinesin, motor region	RT	17	6.2E-1	1.0E0
GOTERM_CC_FAT	microtubule	RT	20	6.3E-1	1.0E0
SMART	KISc	RT	17	6.4E-1	1.0E0
GOTERM_BP_FAT	microtubule-based movement	RT	27	7.5E-1	1.0E0
GOTERM_BP_FAT	microtubule-based process	RT	30	8.3E-1	1.0E0
GOTERM_CC_FAT	microtubule cytoskeleton	RT	28	8.9E-1	1.0E0
GOTERM_CC_FAT	cytoskeletal part	RT	28	8.9E-1	1.0E0
GOTERM_MF_FAT	microtubule motor activity	RT	22	9.2E-1	1.0E0
GOTERM_MF_FAT	motor activity	RT	22	9.2E-1	1.0E0
GOTERM_CC_FAT	cytoskeleton	RT	28	9.4E-1	1.0E0
Annotation Cluster 47	Enrichment Score: 0.13		Count	P_Value	Benjamini
INTERPRO	Pyridoxal phosphate-dependent transferase, major region, subdomain 1	RT	6	5.5E-1	1.0E0
INTERPRO	Aminotransferase, class I and II	RT	4	6.5E-1	1.0E0
GOTERM_MF_FAT	vitamin binding	RT	7	7.4E-1	1.0E0
GOTERM_MF_FAT	pyridoxal phosphate binding	RT	6	8.2E-1	1.0E0
GOTERM_MF_FAT	vitamin B6 binding	RT	6	8.2E-1	1.0E0
GOTERM_MF_FAT	transferase activity, transferring nitrogenous groups	RT	4	9.0E-1	1.0E0
Annotation Cluster 48	Enrichment Score: 0.12		Count	P_Value	Benjamini
INTERPRO	Amino acid transporter, transmembrane	RT	8	3.4E-1	1.0E0
SP_PIR_KEYWORDS	membrane	RT	14	9.0E-1	1.0E0
SP_PIR_KEYWORDS	transmembrane	RT	15	9.3E-1	1.0E0
GOTERM_CC_FAT	integral to membrane	RT	34	9.6E-1	1.0E0
GOTERM_CC_FAT	intrinsic to membrane	RT	34	9.6E-1	1.0E0
Annotation Cluster 49	Enrichment Score: 0.11		Count	P_Value	Benjamini
INTERPRO	Myb, DNA-binding	RT	4	6.5E-1	1.0E0
INTERPRO	Myb transcription factor	RT	3	8.0E-1	1.0E0
INTERPRO	Myb-type HTH DNA-binding domain	RT	3	8.0E-1	1.0E0
INTERPRO	SANT, DNA-binding	RT	6	8.0E-1	1.0E0

SMART	SANT	RT	6	8.1E-1	1.0E0
Annotation Cluster 50	Enrichment Score: 0.1		Count	P_Value	Benjamini
GOTERM_MF_FAT	tetrapyrrole binding	RT	5	7.0E-1	1.0E0
GOTERM_MF_FAT	heme binding	RT	5	7.0E-1	1.0E0
INTERPRO	Cytochrome b5	RT	3	9.0E-1	1.0E0
SP_PIR_KEYWORDS	heme	RT	3	9.0E-1	1.0E0
Annotation Cluster 51	Enrichment Score: 0.1		Count	P_Value	Benjamini
INTERPRO	RNA-directed DNA polymerase (reverse transcriptase), related	RT	4	6.5E-1	1.0E0
INTERPRO	RNA-directed DNA polymerase (reverse transcriptase)	RT	5	6.7E-1	1.0E0
GOTERM_BP_FAT	RNA-dependent DNA replication	RT	5	6.9E-1	1.0E0
GOTERM_MF_FAT	nuclease activity	RT	29	7.3E-1	1.0E0
GOTERM_MF_FAT	DNA polymerase activity	RT	19	8.2E-1	1.0E0
SP_PIR_KEYWORDS	rna-directed dna polymerase	RT	12	8.9E-1	1.0E0
SP_PIR_KEYWORDS	Endonuclease	RT	15	9.3E-1	1.0E0
GOTERM_MF_FAT	RNA-directed DNA polymerase activity	RT	12	9.4E-1	1.0E0
GOTERM_MF_FAT	endonuclease activity	RT	16	9.8E-1	1.0E0
Annotation Cluster 52	Enrichment Score: 0.09		Count	P_Value	Benjamini
INTERPRO	Spc97/Spc98	RT	3	8.0E-1	1.0E0
GOTERM_CC_FAT	microtubule organizing center	RT	3	8.1E-1	1.0E0
GOTERM_CC_FAT	spindle pole	RT	3	8.1E-1	1.0E0
GOTERM_CC_FAT	spindle	RT	3	8.1E-1	1.0E0
GOTERM_BP_FAT	microtubule cytoskeleton organization	RT	3	8.1E-1	1.0E0
Annotation Cluster 53	Enrichment Score: 0.09		Count	P_Value	Benjamini
GOTERM_BP_FAT	nucleoside metabolic process	RT	8	7.5E-1	1.0E0
GOTERM_BP_FAT	purine ribonucleoside metabolic process	RT	3	8.1E-1	1.0E0
GOTERM_BP_FAT	purine nucleoside metabolic process	RT	3	8.1E-1	1.0E0
GOTERM_BP_FAT	ribonucleoside metabolic process	RT	4	8.9E-1	1.0E0
Annotation Cluster 54	Enrichment Score: 0.09		Count	P_Value	Benjamini
GOTERM_MF_FAT	GTPase activity	RT	11	6.2E-1	1.0E0
SP_PIR_KEYWORDS	gtp-binding	RT	23	7.3E-1	9.9E-1
GOTERM_BP_FAT	small GTPase mediated signal transduction	RT	9	8.4E-1	1.0E0
GOTERM_MF_FAT	guanyl nucleotide binding	RT	26	8.8E-1	1.0E0
GOTERM_MF_FAT	GTP binding	RT	26	8.8E-1	1.0E0
GOTERM_MF_FAT	guanyl ribonucleotide binding	RT	26	8.8E-1	1.0E0
INTERPRO	Small GTP-binding protein	RT	9	9.7E-1	1.0E0
Annotation Cluster 55	Enrichment Score: 0.09		Count	P_Value	Benjamini
SP_PIR_KEYWORDS	ubl conjugation pathway	RT	8	7.3E-1	9.9E-1
GOTERM_MF_FAT	small conjugating protein ligase activity	RT	8	7.5E-1	1.0E0
PIR_SUPERFAMILY	PIRSF001567:ubiquitin-protein ligase E2	RT	4	7.6E-1	1.0E0
INTERPRO	Ubiquitin-conjugating enzyme/RWD-like	RT	7	8.1E-1	1.0E0
INTERPRO	Ubiquitin-conjugating enzyme, E2	RT	7	8.1E-1	1.0E0
SMART	UBCc	RT	7	8.2E-1	1.0E0
GOTERM_MF_FAT	ligase activity, forming carbon-nitrogen bonds	RT	17	8.9E-1	1.0E0
GOTERM_MF_FAT	acid-amino acid ligase activity	RT	14	9.1E-1	1.0E0
KEGG_PATHWAY	Ubiquitin mediated proteolysis	RT	5	9.3E-1	1.0E0
Annotation Cluster 56	Enrichment Score: 0.08		Count	P_Value	Benjamini
GOTERM_CC_FAT	cytosol	RT	18	5.9E-1	1.0E0
GOTERM_BP_FAT	macromolecule catabolic process	RT	23	6.9E-1	1.0E0
SP_PIR_KEYWORDS	ubl conjugation pathway	RT	8	7.3E-1	9.9E-1
KEGG_PATHWAY	Proteasome	RT	15	7.3E-1	1.0E0
GOTERM_BP_FAT	protein catabolic process	RT	22	7.4E-1	1.0E0
PIR_SUPERFAMILY	PIRSF001212:multicatalytic endopeptidase complex chain C9	RT	4	7.6E-1	1.0E0
GOTERM_BP_FAT	modification-dependent macromolecule catabolic process	RT	15	7.7E-1	1.0E0
GOTERM_BP_FAT	modification-dependent protein catabolic process	RT	15	7.7E-1	1.0E0
GOTERM_BP_FAT	cellular macromolecule catabolic process	RT	19	8.2E-1	1.0E0
GOTERM_BP_FAT	proteolysis	RT	49	8.4E-1	1.0E0
GOTERM_BP_FAT	cellular protein catabolic process	RT	18	8.6E-1	1.0E0

	Category	Term	RT			Count	P_Value	Benjamini
	GOTERM_BP_FAT	proteolysis involved in cellular protein catabolic process	RT			18	8.6E-1	1.0E0
	INTERPRO	Proteasome, alpha and beta subunits	RT			7	8.8E-1	1.0E0
	INTERPRO	Proteasome, alpha-subunit, conserved site	RT			4	8.9E-1	1.0E0
	GOTERM_CC_FAT	proteasome complex	RT			11	9.0E-1	1.0E0
	INTERPRO	Proteasome, subunit alpha/beta	RT			7	9.2E-1	1.0E0
	SP_PIR_KEYWORDS	proteasome	RT			10	9.3E-1	1.0E0
	GOTERM_BP_FAT	ubiquitin-dependent protein catabolic process	RT			7	9.3E-1	1.0E0
	GOTERM_CC_FAT	proteasome core complex	RT			7	9.3E-1	1.0E0
	GOTERM_MF_FAT	threonine-type endopeptidase activity	RT			7	9.4E-1	1.0E0
	GOTERM_MF_FAT	threonine-type peptidase activity	RT			7	9.4E-1	1.0E0
	SP_PIR_KEYWORDS	threonine protease	RT			3	9.5E-1	1.0E0
	Annotation Cluster 57	Enrichment Score: 0.07	G			Count	P_Value	Benjamini
	GOTERM_MF_FAT	GTPase activity	RT			11	6.2E-1	1.0E0
	INTERPRO	Tubulin/FtsZ, GTPase domain	RT			5	8.0E-1	1.0E0
	INTERPRO	Tubulin	RT			5	8.0E-1	1.0E0
	INTERPRO	Tubulin, conserved site	RT			5	8.0E-1	1.0E0
	GOTERM_BP_FAT	protein polymerization	RT			5	8.1E-1	1.0E0
	PIR_SUPERFAMILY	PIRSF002306:tubulin	RT			4	8.6E-1	1.0E0
	INTERPRO	Tubulin/FtsZ, 2-layer sandwich domain	RT			4	8.9E-1	1.0E0
	GOTERM_BP_FAT	cellular protein complex assembly	RT			6	8.9E-1	1.0E0
	INTERPRO	Beta tubulin, autoregulation binding site	RT			3	9.5E-1	1.0E0
	GOTERM_BP_FAT	protein complex assembly	RT			6	9.6E-1	1.0E0
	GOTERM_BP_FAT	protein complex biogenesis	RT			6	9.6E-1	1.0E0
	Annotation Cluster 58	Enrichment Score: 0.07	G			Count	P_Value	Benjamini
	SP_PIR_KEYWORDS	Protease	RT			21	7.0E-1	9.9E-1
	INTERPRO	Peptidase, cysteine peptidase active site	RT			17	7.7E-1	1.0E0
	INTERPRO	Peptidase C1A, papain	RT			15	8.1E-1	1.0E0
	INTERPRO	Peptidase C1A, papain C-terminal	RT			15	8.1E-1	1.0E0
	SMART	Pept_C1	RT			15	8.2E-1	1.0E0
	GOTERM_MF_FAT	cysteine-type endopeptidase activity	RT			13	8.7E-1	1.0E0
	GOTERM_MF_FAT	cysteine-type peptidase activity	RT			16	8.9E-1	1.0E0
	GOTERM_MF_FAT	peptidase activity	RT			43	8.9E-1	1.0E0
	GOTERM_MF_FAT	peptidase activity, acting on L-amino acid peptides	RT			35	9.6E-1	1.0E0
	GOTERM_MF_FAT	endopeptidase activity	RT			23	9.6E-1	1.0E0
	Annotation Cluster 59	Enrichment Score: 0.07	G			Count	P_Value	Benjamini
	GOTERM_BP_FAT	pyruvate metabolic process	RT			3	8.1E-1	1.0E0
	GOTERM_BP_FAT	gluconeogenesis	RT			3	8.1E-1	1.0E0
	GOTERM_BP_FAT	monosaccharide biosynthetic process	RT			3	8.1E-1	1.0E0
	GOTERM_BP_FAT	hexose biosynthetic process	RT			3	8.1E-1	1.0E0
	GOTERM_BP_FAT	cellular carbohydrate biosynthetic process	RT			4	8.9E-1	1.0E0
	GOTERM_BP_FAT	carbohydrate biosynthetic process	RT			4	8.9E-1	1.0E0
	GOTERM_BP_FAT	alcohol biosynthetic process	RT			3	9.1E-1	1.0E0
	Annotation Cluster 60	Enrichment Score: 0.07	G			Count	P_Value	Benjamini
	GOTERM_MF_FAT	carbon-nitrogen lyase activity	RT			3	8.1E-1	1.0E0
	GOTERM_MF_FAT	ammonia-lyase activity	RT			3	8.1E-1	1.0E0
	SP_PIR_KEYWORDS	lyase	RT			9	9.2E-1	1.0E0
	Annotation Cluster 61	Enrichment Score: 0.07	G			Count	P_Value	Benjamini
	GOTERM_MF_FAT	DNA-directed DNA polymerase activity	RT			7	6.1E-1	1.0E0
	INTERPRO	DNA-directed DNA polymerase, family B	RT			3	9.0E-1	1.0E0
	SP_PIR_KEYWORDS	dna-directed dna polymerase	RT			3	9.0E-1	1.0E0
	SMART	POLBc	RT			3	9.0E-1	1.0E0
	SP_PIR_KEYWORDS	dna replication	RT			4	9.8E-1	1.0E0
	Annotation Cluster 62	Enrichment Score: 0.07	G			Count	P_Value	Benjamini
	GOTERM_MF_FAT	metal cluster binding	RT			12	7.3E-1	1.0E0
	GOTERM_MF_FAT	iron-sulfur cluster binding	RT			12	7.3E-1	1.0E0
	GOTERM_MF_FAT	4 iron, 4 sulfur cluster binding	RT			5	8.1E-1	1.0E0
	INTERPRO	4Fe-4S ferredoxin, iron-sulphur binding, subgroup	RT			4	9.7E-1	1.0E0

INTERPRO	4Fe-4S ferredoxin, iron-sulpur binding domain	RT	4	9.7E-1	1.0E0
INTERPRO	4Fe-4S ferredoxin, iron-sulphur binding, conserved site	RT	3	9.8E-1	1.0E0
Annotation Cluster 63	Enrichment Score: 0.07		Count	P_Value	Benjamini
GOTERM_BP_FAT	protein amino acid dephosphorylation	RT	8	7.5E-1	1.0E0
INTERPRO	Protein-tyrosine phosphatase, active site	RT	4	7.9E-1	1.0E0
INTERPRO	Dual-specific/protein-tyrosine phosphatase, conserved region	RT	3	9.0E-1	1.0E0
INTERPRO	Protein-tyrosine phosphatase	RT	3	9.0E-1	1.0E0
GOTERM_MF_FAT	protein tyrosine phosphatase activity	RT	3	9.8E-1	1.0E0
Annotation Cluster 64	Enrichment Score: 0.07		Count	P_Value	Benjamini
GOTERM_BP_FAT	protein amino acid dephosphorylation	RT	8	7.5E-1	1.0E0
INTERPRO	Dual specificity phosphatase, catalytic domain	RT	3	9.0E-1	1.0E0
INTERPRO	Protein-tyrosine phosphatase, dual specificity	RT	3	9.0E-1	1.0E0
GOTERM_MF_FAT	protein tyrosine/serine/threonine phosphatase activity	RT	3	9.1E-1	1.0E0
Annotation Cluster 65	Enrichment Score: 0.06		Count	P_Value	Benjamini
INTERPRO	WD40 repeat, conserved site	RT	19	4.1E-1	1.0E0
SP_PIR_KEYWORDS	wd repeat	RT	25	9.3E-1	1.0E0
INTERPRO	WD40 repeat 2	RT	21	9.6E-1	1.0E0
INTERPRO	WD40 repeat, region	RT	23	9.6E-1	1.0E0
INTERPRO	WD40 repeat, subgroup	RT	27	9.7E-1	1.0E0
INTERPRO	WD40 repeat	RT	36	9.8E-1	1.0E0
SMART	WD40	RT	36	9.8E-1	1.0E0
INTERPRO	WD40/YVTN repeat-like	RT	34	9.9E-1	1.0E0
Annotation Cluster 66	Enrichment Score: 0.05		Count	P_Value	Benjamini
INTERPRO	AMP-dependent synthetase and ligase	RT	4	8.9E-1	1.0E0
COG_ONTOLOGY	Lipid metabolism	RT	7	8.9E-1	1.0E0
GOTERM_MF_FAT	fatty-acid ligase activity	RT	3	9.1E-1	1.0E0
GOTERM_MF_FAT	long-chain-fatty-acid-CoA ligase activity	RT	3	9.1E-1	1.0E0
GOTERM_MF_FAT	ligase activity, forming carbon-sulfur bonds	RT	3	9.1E-1	1.0E0
Annotation Cluster 67	Enrichment Score: 0.04		Count	P_Value	Benjamini
GOTERM_CC_FAT	nuclear envelope	RT	3	9.0E-1	1.0E0
GOTERM_CC_FAT	pore complex	RT	3	9.0E-1	1.0E0
GOTERM_CC_FAT	nuclear pore	RT	3	9.0E-1	1.0E0
GOTERM_CC_FAT	envelope	RT	3	9.0E-1	1.0E0
GOTERM_CC_FAT	organelle envelope	RT	3	9.0E-1	1.0E0
Annotation Cluster 68	Enrichment Score: 0.04		Count	P_Value	Benjamini
GOTERM_CC_FAT	organelle lumen	RT	3	9.0E-1	1.0E0
GOTERM_CC_FAT	intracellular organelle lumen	RT	3	9.0E-1	1.0E0
GOTERM_CC_FAT	nuclear lumen	RT	3	9.0E-1	1.0E0
GOTERM_CC_FAT	membrane-enclosed lumen	RT	3	9.0E-1	1.0E0
Annotation Cluster 69	Enrichment Score: 0.04		Count	P_Value	Benjamini
KEGG_PATHWAY	Nucleotide excision repair	RT	8	8.6E-1	1.0E0
KEGG_PATHWAY	Mismatch repair	RT	6	9.5E-1	1.0E0
KEGG_PATHWAY	DNA replication	RT	9	9.6E-1	1.0E0
Annotation Cluster 70	Enrichment Score: 0.03		Count	P_Value	Benjamini
INTERPRO	Kelch-type beta propeller	RT	4	8.9E-1	1.0E0
INTERPRO	Kelch repeat type 1	RT	3	9.5E-1	1.0E0
SMART	Kelch	RT	3	9.5E-1	1.0E0
Annotation Cluster 71	Enrichment Score: 0.02		Count	P_Value	Benjamini
INTERPRO	Heat shock protein DnaJ, N-terminal	RT	4	9.4E-1	1.0E0
SMART	DnaJ	RT	4	9.4E-1	1.0E0
GOTERM_MF_FAT	heat shock protein binding	RT	4	9.5E-1	1.0E0
INTERPRO	Molecular chaperone, heat shock protein, Hsp40, DnaJ	RT	3	9.8E-1	1.0E0
Annotation Cluster 72	Enrichment Score: 0.02		Count	P_Value	Benjamini
INTERPRO	DNA-dependent ATPase MCM	RT	3	9.5E-1	1.0E0
SMART	MCM	RT	3	9.5E-1	1.0E0
KEGG_PATHWAY	DNA replication	RT	9	9.6E-1	1.0E0
Annotation Cluster 73	Enrichment Score: 0.02		Count	P_Value	Benjamini
GOTERM_MF_FAT	N-acyltransferase activity	RT	5	9.4E-1	1.0E0

GOTERM_MF_FAT	acetyltransferase activity	RT	5	9.4E-1	1.0E0
INTERPRO	GCN5-related N-acetyltransferase	RT	4	9.7E-1	1.0E0
GOTERM_MF_FAT	N-acetyltransferase activity	RT	4	9.7E-1	1.0E0
Annotation Cluster 74	Enrichment Score: 0.02		Count	P_Value	Benjamini
GOTERM_BP_FAT	monosaccharide metabolic process	RT	12	9.1E-1	1.0E0
GOTERM_BP_FAT	glucose metabolic process	RT	10	9.3E-1	1.0E0
GOTERM_BP_FAT	hexose metabolic process	RT	10	9.3E-1	1.0E0
SP_PIR_KEYWORDS	glycolysis	RT	3	9.5E-1	1.0E0
GOTERM_BP_FAT	glycolysis	RT	5	9.7E-1	1.0E0
GOTERM_BP_FAT	generation of precursor metabolites and energy	RT	12	9.7E-1	1.0E0
GOTERM_BP_FAT	monosaccharide catabolic process	RT	6	9.8E-1	1.0E0
GOTERM_BP_FAT	alcohol catabolic process	RT	6	9.8E-1	1.0E0
GOTERM_BP_FAT	cellular carbohydrate catabolic process	RT	6	9.8E-1	1.0E0
GOTERM_BP_FAT	glucose catabolic process	RT	6	9.8E-1	1.0E0
GOTERM_BP_FAT	carbohydrate catabolic process	RT	6	9.8E-1	1.0E0
GOTERM_BP_FAT	hexose catabolic process	RT	6	9.8E-1	1.0E0
Annotation Cluster 75	Enrichment Score: 0.01		Count	P_Value	Benjamini
GOTERM_CC_FAT	proton-transporting two-sector ATPase complex, proton-transporting domain	RT	4	8.1E-1	1.0E0
GOTERM_BP_FAT	transmembrane transport	RT	11	9.0E-1	1.0E0
GOTERM_CC_FAT	proton-transporting V-type ATPase, V0 domain	RT	3	9.0E-1	1.0E0
KEGG_PATHWAY	Oxidative phosphorylation	RT	6	9.2E-1	1.0E0
GOTERM_BP_FAT	ion transmembrane transport	RT	7	9.3E-1	1.0E0
GOTERM_MF_FAT	inorganic cation transmembrane transporter activity	RT	9	9.3E-1	1.0E0
GOTERM_BP_FAT	hydrogen transport	RT	7	9.6E-1	1.0E0
GOTERM_BP_FAT	proton transport	RT	7	9.6E-1	1.0E0
GOTERM_CC_FAT	proton-transporting two-sector ATPase complex	RT	7	9.6E-1	1.0E0
INTERPRO	ATPase, P-type, ATPase-associated region	RT	5	9.6E-1	1.0E0
GOTERM_MF_FAT	hydrogen ion transmembrane transporter activity	RT	7	9.6E-1	1.0E0
GOTERM_MF_FAT	monovalent inorganic cation transmembrane transporter activity	RT	7	9.6E-1	1.0E0
GOTERM_BP_FAT	oxidative phosphorylation	RT	6	9.6E-1	1.0E0
GOTERM_BP_FAT	energy coupled proton transport, down electrochemical gradient	RT	6	9.6E-1	1.0E0
GOTERM_BP_FAT	ATP synthesis coupled proton transport	RT	6	9.6E-1	1.0E0
GOTERM_CC_FAT	proton-transporting V-type ATPase complex	RT	4	9.7E-1	1.0E0
GOTERM_BP_FAT	generation of precursor metabolites and energy	RT	12	9.7E-1	1.0E0
GOTERM_BP_FAT	ion transport	RT	11	9.7E-1	1.0E0
GOTERM_BP_FAT	cation transport	RT	11	9.7E-1	1.0E0
GOTERM_BP_FAT	monovalent inorganic cation transport	RT	7	9.8E-1	1.0E0
INTERPRO	ATPase, P-type, K/Mg/Cd/Cu/Zn/Na/Ca/Na/H-transporter	RT	5	9.8E-1	1.0E0
GOTERM_MF_FAT	proton-transporting ATPase activity, rotational mechanism	RT	3	9.8E-1	1.0E0
GOTERM_MF_FAT	ATPase activity, coupled to transmembrane movement of ions, phosphorylative mechanism	RT	5	9.8E-1	1.0E0
GOTERM_BP_FAT	ATP metabolic process	RT	11	9.8E-1	1.0E0
GOTERM_BP_FAT	ATP biosynthetic process	RT	11	9.8E-1	1.0E0
GOTERM_BP_FAT	nitrogen compound biosynthetic process	RT	23	9.9E-1	1.0E0
GOTERM_MF_FAT	cation-transporting ATPase activity	RT	4	9.9E-1	1.0E0
GOTERM_BP_FAT	nucleobase, nucleoside and nucleotide biosynthetic process	RT	19	9.9E-1	1.0E0
GOTERM_BP_FAT	nucleobase, nucleoside, nucleotide and nucleic acid biosynthetic process	RT	19	9.9E-1	1.0E0
GOTERM_BP_FAT	purine ribonucleotide biosynthetic process	RT	12	9.9E-1	1.0E0
GOTERM_BP_FAT	purine ribonucleotide metabolic process	RT	12	9.9E-1	1.0E0
GOTERM_BP_FAT	ribonucleotide biosynthetic process	RT	12	9.9E-1	1.0E0
GOTERM_BP_FAT	ribonucleotide metabolic process	RT	12	9.9E-1	1.0E0
GOTERM_CC_FAT	proton-transporting two-sector ATPase complex, catalytic domain	RT	3	9.9E-1	1.0E0
GOTERM_MF_FAT	ATPase activity, coupled to transmembrane movement of ions	RT	8	9.9E-1	1.0E0
GOTERM_BP_FAT	purine nucleotide biosynthetic process	RT	12	9.9E-1	1.0E0

	GOTERM_BP_FAT	nucleoside triphosphate biosynthetic process	RT		11	9.9E-1	1.0E0
	GOTERM_BP_FAT	nucleoside triphosphate metabolic process	RT		11	9.9E-1	1.0E0
	GOTERM_BP_FAT	ribonucleoside triphosphate metabolic process	RT		11	9.9E-1	1.0E0
	GOTERM_BP_FAT	purine ribonucleoside triphosphate metabolic process	RT		11	9.9E-1	1.0E0
	GOTERM_BP_FAT	ribonucleoside triphosphate biosynthetic process	RT		11	9.9E-1	1.0E0
	GOTERM_BP_FAT	purine nucleoside triphosphate biosynthetic process	RT		11	9.9E-1	1.0E0
	GOTERM_BP_FAT	purine nucleoside triphosphate metabolic process	RT		11	9.9E-1	1.0E0
	GOTERM_BP_FAT	purine ribonucleoside triphosphate biosynthetic process	RT		11	9.9E-1	1.0E0
	GOTERM_BP_FAT	purine nucleotide metabolic process	RT		12	1.0E0	1.0E0
	GOTERM_MF_FAT	ATPase activity, coupled to transmembrane movement of substances	RT		11	1.0E0	1.0E0
	GOTERM_MF_FAT	ATPase activity, coupled to movement of substances	RT		11	1.0E0	1.0E0
	GOTERM_BP_FAT	nucleotide biosynthetic process	RT		16	1.0E0	1.0E0
	GOTERM_MF_FAT	P-P-bond-hydrolysis-driven transmembrane transporter activity	RT		11	1.0E0	1.0E0
	GOTERM_MF_FAT	primary active transmembrane transporter activity	RT		11	1.0E0	1.0E0
	GOTERM_MF_FAT	hydrolase activity, acting on acid anhydrides, catalyzing transmembrane movement of substances	RT		11	1.0E0	1.0E0
	Annotation Cluster 76	Enrichment Score: 0.01	G		Count	P_Value	Benjamini
	INTERPRO	Tetratricopeptide region	RT		11	9.7E-1	1.0E0
	INTERPRO	Tetratricopeptide repeat	RT		11	9.7E-1	1.0E0
	SP_PIR_KEYWORDS	tpr repeat	RT		4	9.7E-1	1.0E0
	SMART	TPR	RT		11	9.7E-1	1.0E0
	INTERPRO	Tetratricopeptide-like helical	RT		14	9.8E-1	1.0E0
	INTERPRO	Tetratricopeptide TPR-1	RT		8	9.9E-1	1.0E0
	Annotation Cluster 77	Enrichment Score: 0.01	G		Count	P_Value	Benjamini
	INTERPRO	Dynein heavy chain, N-terminal region 2	RT		5	9.6E-1	1.0E0
	INTERPRO	Dynein heavy chain	RT		5	9.8E-1	1.0E0
	GOTERM_CC_FAT	dynein complex	RT		5	9.8E-1	1.0E0
	COG_ONTOLOGY	Cytoskeleton	RT		8	1.0E0	1.0E0
	GOTERM_CC_FAT	microtubule associated complex	RT		5	1.0E0	1.0E0
	Annotation Cluster 78	Enrichment Score: 0.01	G		Count	P_Value	Benjamini
	INTERPRO	Chaperonin TCP-1, conserved site	RT		4	9.7E-1	1.0E0
	PIR_SUPERFAMILY	PIRSF002584:molecular chaperone t-complex-type	RT		3	9.8E-1	1.0E0
	INTERPRO	Chaperone, tailless complex polypeptide 1	RT		3	9.9E-1	1.0E0
	SP_PIR_KEYWORDS	cytoplasm	RT		7	9.9E-1	1.0E0
	INTERPRO	Chaperonin Cpn60/TCP-1	RT		3	9.9E-1	1.0E0
	SP_PIR_KEYWORDS	Chaperone	RT		4	1.0E0	1.0E0
	Annotation Cluster 79	Enrichment Score: 0	G		Count	P_Value	Benjamini
	SP_PIR_KEYWORDS	Rotamase	RT		3	9.9E-1	1.0E0
	GOTERM_MF_FAT	cis-trans isomerase activity	RT		3	9.9E-1	1.0E0
	GOTERM_MF_FAT	peptidyl-prolyl cis-trans isomerase activity	RT		3	9.9E-1	1.0E0

192 terms were not clustered.

Please cite Nature Protocols 2009; 4(1):44 & Genome Biology 2003; 4(5):P3 within any publication that makes use of any methods inspired by **DAVID**.

Term of Service | Contact Us | Site Map

DAVID Bioinformatics Resources 6.7
National Institute of Allergy and Infectious Diseases (NIAID), NIH

Functional Annotation Clustering

Help and Manual

Current Gene List: 3_Random8
Current Background: Giardia intestinalis
3000 DAVID IDs

Options Classification Stringency Medium
Rerun using options | Create Sublist

75 Cluster(s)

Download File

Annotation Cluster 1	Enrichment Score: 0.74		Count	P_Value	Benjamini
INTERPRO	EF-HAND 1	RT	17	3.6E-2	1.0E0
SP_PIR_KEYWORDS	calcium	RT	13	6.5E-2	1.0E0
INTERPRO	EF-HAND 2	RT	13	2.2E-1	1.0E0
SMART	EFh	RT	9	2.4E-1	1.0E0
INTERPRO	EF hand	RT	9	2.6E-1	1.0E0
INTERPRO	Calcium-binding EF-hand	RT	9	2.6E-1	1.0E0
INTERPRO	EF-Hand type	RT	13	3.2E-1	1.0E0
GOTERM_MF_FAT	calcium ion binding	RT	27	4.5E-1	1.0E0
Annotation Cluster 2	Enrichment Score: 0.67		Count	P_Value	Benjamini
GOTERM_BP_FAT	microtubule-based process	RT	38	7.9E-2	1.0E0
GOTERM_CC_FAT	cytoskeletal part	RT	35	1.0E-1	1.0E0
GOTERM_CC_FAT	microtubule cytoskeleton	RT	35	1.0E-1	1.0E0
GOTERM_CC_FAT	cytoskeleton	RT	36	1.1E-1	1.0E0
GOTERM_MF_FAT	motor activity	RT	29	1.7E-1	1.0E0
GOTERM_MF_FAT	microtubule motor activity	RT	29	1.7E-1	1.0E0
GOTERM_BP_FAT	microtubule-based movement	RT	31	2.7E-1	1.0E0
SMART	KISc	RT	19	3.0E-1	1.0E0
INTERPRO	Kinesin, motor region	RT	19	3.3E-1	1.0E0
SP_PIR_KEYWORDS	microtubule	RT	21	3.6E-1	9.7E-1
GOTERM_CC_FAT	microtubule	RT	21	3.7E-1	1.0E0
SP_PIR_KEYWORDS	motor protein	RT	16	4.0E-1	9.6E-1
INTERPRO	Kinesin, motor region, conserved site	RT	12	5.0E-1	1.0E0
Annotation Cluster 3	Enrichment Score: 0.51		Count	P_Value	Benjamini
GOTERM_BP_FAT	lipoprotein biosynthetic process	RT	5	3.1E-1	1.0E0
GOTERM_BP_FAT	protein amino acid lipidation	RT	5	3.1E-1	1.0E0
GOTERM_BP_FAT	lipoprotein metabolic process	RT	5	3.1E-1	1.0E0
Annotation Cluster 4	Enrichment Score: 0.51		Count	P_Value	Benjamini
GOTERM_MF_FAT	pseudouridine synthase activity	RT	7	1.4E-1	1.0E0
GOTERM_BP_FAT	pseudouridine synthesis	RT	7	1.4E-1	1.0E0
GOTERM_MF_FAT	intramolecular transferase activity	RT	9	2.6E-1	1.0E0
GOTERM_BP_FAT	RNA modification	RT	11	4.7E-1	1.0E0
PIR_SUPERFAMILY	PIRSF001430:tRNA_psdUrid_synth	RT	3	6.1E-1	1.0E0
INTERPRO	Pseudouridine synthase I, TruA	RT	3	6.2E-1	1.0E0
Annotation Cluster 5	Enrichment Score: 0.45		Count	P_Value	Benjamini
GOTERM_MF_FAT	enzyme activator activity	RT	8	2.0E-1	1.0E0
GOTERM_BP_FAT	regulation of Ras protein signal transduction	RT	8	2.0E-1	1.0E0
GOTERM_BP_FAT	regulation of small GTPase mediated signal transduction	RT	8	2.0E-1	1.0E0
GOTERM_MF_FAT	GTPase regulator activity	RT	9	2.6E-1	1.0E0
GOTERM_MF_FAT	small GTPase regulator activity	RT	9	2.6E-1	1.0E0
GOTERM_BP_FAT	regulation of GTPase activity	RT	7	2.8E-1	1.0E0
GOTERM_BP_FAT	regulation of hydrolase activity	RT	7	2.8E-1	1.0E0
GOTERM_MF_FAT	nucleoside-triphosphatase regulator activity	RT	10	3.1E-1	1.0E0
GOTERM_MF_FAT	GTPase activator activity	RT	6	3.8E-1	1.0E0
GOTERM_BP_FAT	regulation of Ras GTPase activity	RT	6	3.9E-1	1.0E0
GOTERM_MF_FAT	Rab GTPase activator activity	RT	4	4.5E-1	1.0E0

	GOTERM_MF_FAT	Ras GTPase activator activity	RT			4	4.5E-1	1.0E0
	GOTERM_BP_FAT	regulation of Rab protein signal transduction	RT			4	4.5E-1	1.0E0
	GOTERM_BP_FAT	regulation of Rab GTPase activity	RT			4	4.5E-1	1.0E0
	SMART	TBC	RT			3	6.1E-1	1.0E0
	INTERPRO	RabGAP/TBC	RT			3	6.2E-1	1.0E0
	GOTERM_BP_FAT	regulation of ARF protein signal transduction	RT			4	6.6E-1	1.0E0
	Annotation Cluster 6	Enrichment Score: 0.39	G			Count	P_Value	Benjamini
	GOTERM_BP_FAT	translational initiation	RT			9	2.6E-1	1.0E0
	GOTERM_MF_FAT	translation initiation factor activity	RT			17	3.6E-1	1.0E0
	GOTERM_MF_FAT	translation factor activity, nucleic acid binding	RT			21	3.9E-1	1.0E0
	SP_PIR_KEYWORDS	Initiation factor	RT			11	7.6E-1	9.9E-1
	Annotation Cluster 7	Enrichment Score: 0.38	G			Count	P_Value	Benjamini
	SMART	CYCLIN	RT			5	3.0E-1	1.0E0
	INTERPRO	Cyclin	RT			5	3.1E-1	1.0E0
	SP_PIR_KEYWORDS	cyclin	RT			8	3.3E-1	9.8E-1
	INTERPRO	Cyclin-related	RT			5	5.1E-1	1.0E0
	INTERPRO	Cyclin, N-terminal	RT			4	8.0E-1	1.0E0
	Annotation Cluster 8	Enrichment Score: 0.38	G			Count	P_Value	Benjamini
	SP_PIR_KEYWORDS	atp-binding	RT			158	1.2E-1	1.0E0
	INTERPRO	Protein kinase, ATP binding site	RT			54	1.4E-1	1.0E0
	INTERPRO	Serine/threonine protein kinase, active site	RT			58	1.5E-1	1.0E0
	SP_PIR_KEYWORDS	kinase	RT			199	1.7E-1	1.0E0
	GOTERM_MF_FAT	protein serine/threonine kinase activity	RT			66	2.2E-1	1.0E0
	SP_PIR_KEYWORDS	nucleotide-binding	RT			162	2.6E-1	9.9E-1
	SMART	S_TKc	RT			37	2.9E-1	1.0E0
	INTERPRO	Serine/threonine protein kinase-related	RT			140	3.2E-1	1.0E0
	INTERPRO	Serine/threonine protein kinase	RT			37	3.2E-1	1.0E0
	GOTERM_MF_FAT	protein kinase activity	RT			161	3.7E-1	1.0E0
	SP_PIR_KEYWORDS	serine/threonine-protein kinase	RT			38	3.8E-1	9.5E-1
	INTERPRO	Protein kinase, core	RT			158	3.9E-1	1.0E0
	GOTERM_BP_FAT	protein amino acid phosphorylation	RT			159	4.1E-1	1.0E0
	GOTERM_BP_FAT	phosphorylation	RT			170	4.2E-1	1.0E0
	GOTERM_BP_FAT	phosphorus metabolic process	RT			175	6.6E-1	1.0E0
	GOTERM_BP_FAT	phosphate metabolic process	RT			175	6.6E-1	1.0E0
	GOTERM_MF_FAT	adenyl ribonucleotide binding	RT			301	7.2E-1	1.0E0
	GOTERM_MF_FAT	ATP binding	RT			301	7.2E-1	1.0E0
	GOTERM_MF_FAT	purine nucleoside binding	RT			304	7.6E-1	1.0E0
	GOTERM_MF_FAT	adenyl nucleotide binding	RT			304	7.6E-1	1.0E0
	GOTERM_MF_FAT	nucleoside binding	RT			305	8.0E-1	1.0E0
	GOTERM_MF_FAT	purine ribonucleotide binding	RT			325	8.5E-1	1.0E0
	GOTERM_MF_FAT	ribonucleotide binding	RT			325	8.5E-1	1.0E0
	GOTERM_MF_FAT	nucleotide binding	RT			349	8.7E-1	1.0E0
	GOTERM_MF_FAT	purine nucleotide binding	RT			328	8.7E-1	1.0E0
	Annotation Cluster 9	Enrichment Score: 0.37	G			Count	P_Value	Benjamini
	SMART	SET	RT			6	2.0E-1	1.0E0
	INTERPRO	SET	RT			6	2.1E-1	1.0E0
	SMART	PostSET	RT			3	6.1E-1	1.0E0
	INTERPRO	Post-SET zinc-binding region	RT			3	6.2E-1	1.0E0
	SP_PIR_KEYWORDS	methyltransferase	RT			10	9.5E-1	1.0E0
	Annotation Cluster 10	Enrichment Score: 0.36	G			Count	P_Value	Benjamini
	SMART	FU	RT			111	3.0E-1	1.0E0
	INTERPRO	Giardia variant-specific surface protein	RT			142	3.4E-1	1.0E0
	INTERPRO	Furin-like repeat	RT			111	3.6E-1	1.0E0
	SMART	EGF	RT			109	6.3E-1	1.0E0
	INTERPRO	EGF-like	RT			109	6.8E-1	1.0E0
	Annotation Cluster 11	Enrichment Score: 0.36	G			Count	P_Value	Benjamini
	SP_PIR_KEYWORDS	egf-like domain	RT			13	2.1E-1	9.9E-1
	INTERPRO	EGF-like, type 3	RT			20	5.2E-1	1.0E0

INTERPRO	EGF, extracellular	RT			13	7.9E-1	1.0E0
Annotation Cluster 12	Enrichment Score: 0.36				Count	P_Value	Benjamini
INTERPRO	Alcohol dehydrogenase, iron-type	RT			5	3.1E-1	1.0E0
COG_ONTOLOGY	Energy production and conversion	RT			7	4.6E-1	1.0E0
PIR_SUPERFAMILY	PIRSF000113:iron-containing alcohol dehydrogenase	RT			3	6.1E-1	1.0E0
Annotation Cluster 13	Enrichment Score: 0.33				Count	P_Value	Benjamini
GOTERM_MF_FAT	cofactor binding	RT			24	2.4E-1	1.0E0
GOTERM_MF_FAT	vitamin binding	RT			9	2.6E-1	1.0E0
INTERPRO	Aminotransferase, class I and II	RT			5	3.1E-1	1.0E0
GOTERM_MF_FAT	vitamin B6 binding	RT			8	3.4E-1	1.0E0
GOTERM_MF_FAT	pyridoxal phosphate binding	RT			8	3.4E-1	1.0E0
INTERPRO	Pyridoxal phosphate-dependent transferase, major region, subdomain 1	RT			6	5.5E-1	1.0E0
KEGG_PATHWAY	Alanine, aspartate and glutamate metabolism	RT			3	6.1E-1	1.0E0
INTERPRO	Pyridoxal phosphate-dependent transferase, major region, subdomain 2	RT			3	6.2E-1	1.0E0
GOTERM_MF_FAT	transferase activity, transferring nitrogenous groups	RT			5	6.8E-1	1.0E0
SP_PIR_KEYWORDS	Aminotransferase	RT			3	9.0E-1	9.9E-1
GOTERM_MF_FAT	transaminase activity	RT			3	9.0E-1	1.0E0
Annotation Cluster 14	Enrichment Score: 0.32				Count	P_Value	Benjamini
GOTERM_CC_FAT	spindle	RT			4	4.1E-1	1.0E0
GOTERM_CC_FAT	microtubule organizing center	RT			4	4.1E-1	1.0E0
GOTERM_CC_FAT	spindle pole	RT			4	4.1E-1	1.0E0
INTERPRO	Spc97/Spc98	RT			4	4.4E-1	1.0E0
GOTERM_BP_FAT	microtubule cytoskeleton organization	RT			4	4.5E-1	1.0E0
GOTERM_BP_FAT	cytoskeleton organization	RT			9	8.3E-1	1.0E0
Annotation Cluster 15	Enrichment Score: 0.31				Count	P_Value	Benjamini
GOTERM_CC_FAT	proton-transporting V-type ATPase complex	RT			8	1.7E-1	1.0E0
GOTERM_BP_FAT	nitrogen compound biosynthetic process	RT			33	2.0E-1	1.0E0
GOTERM_BP_FAT	purine nucleotide metabolic process	RT			21	2.3E-1	1.0E0
INTERPRO	ATPase, P-type, ATPase-associated region	RT			9	2.6E-1	1.0E0
GOTERM_CC_FAT	proton-transporting V-type ATPase, V0 domain	RT			5	2.8E-1	1.0E0
GOTERM_BP_FAT	purine nucleotide biosynthetic process	RT			20	2.8E-1	1.0E0
INTERPRO	ATPase, P-type phosphorylation site	RT			7	2.8E-1	1.0E0
GOTERM_BP_FAT	purine ribonucleotide metabolic process	RT			19	3.3E-1	1.0E0
GOTERM_BP_FAT	ribonucleotide metabolic process	RT			19	3.3E-1	1.0E0
GOTERM_BP_FAT	ribonucleotide biosynthetic process	RT			19	3.3E-1	1.0E0
GOTERM_BP_FAT	purine ribonucleotide biosynthetic process	RT			19	3.3E-1	1.0E0
GOTERM_BP_FAT	ATP metabolic process	RT			17	3.6E-1	1.0E0
GOTERM_BP_FAT	ATP biosynthetic process	RT			17	3.6E-1	1.0E0
GOTERM_BP_FAT	nucleobase, nucleoside and nucleotide biosynthetic process	RT			27	3.8E-1	1.0E0
GOTERM_BP_FAT	nucleobase, nucleoside, nucleotide and nucleic acid biosynthetic process	RT			27	3.8E-1	1.0E0
INTERPRO	ATPase, P-type, K/Mg/Cd/Cu/Zn/Na/Ca/Na/H-transporter	RT			9	3.9E-1	1.0E0
GOTERM_BP_FAT	purine nucleoside triphosphate metabolic process	RT			18	3.9E-1	1.0E0
GOTERM_BP_FAT	nucleoside triphosphate biosynthetic process	RT			18	3.9E-1	1.0E0
GOTERM_BP_FAT	purine nucleoside triphosphate biosynthetic process	RT			18	3.9E-1	1.0E0
GOTERM_BP_FAT	nucleoside triphosphate metabolic process	RT			18	3.9E-1	1.0E0
GOTERM_BP_FAT	purine ribonucleoside triphosphate metabolic process	RT			18	3.9E-1	1.0E0
GOTERM_BP_FAT	purine ribonucleoside triphosphate biosynthetic process	RT			18	3.9E-1	1.0E0
GOTERM_BP_FAT	ribonucleoside triphosphate metabolic process	RT			18	3.9E-1	1.0E0
GOTERM_BP_FAT	ribonucleoside triphosphate biosynthetic process	RT			18	3.9E-1	1.0E0
GOTERM_MF_FAT	ATPase activity, coupled to transmembrane movement of ions, phosphorylative mechanism	RT			9	3.9E-1	1.0E0
GOTERM_BP_FAT	nucleotide biosynthetic process	RT			25	4.1E-1	1.0E0
SP_PIR_KEYWORDS	phosphoprotein	RT			4	4.4E-1	9.6E-1
GOTERM_BP_FAT	generation of precursor metabolites and energy	RT			17	4.5E-1	1.0E0

☐	GOTERM_CC_FAT	proton-transporting two-sector ATPase complex, proton-transporting domain	RT			5	4.7E-1	1.0E0
☐	GOTERM_CC_FAT	proton-transporting two-sector ATPase complex	RT			10	5.0E-1	1.0E0
☐	GOTERM_MF_FAT	ATPase activity, coupled to transmembrane movement of ions	RT			13	5.4E-1	1.0E0
☐	GOTERM_BP_FAT	hydrogen transport	RT			10	5.6E-1	1.0E0
☐	GOTERM_BP_FAT	proton transport	RT			10	5.6E-1	1.0E0
☐	GOTERM_BP_FAT	cation transport	RT			15	5.9E-1	1.0E0
☐	GOTERM_BP_FAT	ion transport	RT			15	5.9E-1	1.0E0
☐	PIR_SUPERFAMILY	PIRSF001292:vacuolar H(+)-transporting ATPase 16K chain	RT			3	6.1E-1	1.0E0
☐	INTERPRO	ATPase, P-type cation-transporter, C-terminal	RT			3	6.2E-1	1.0E0
☐	INTERPRO	ATPase, V0 complex, proteolipid subunit C	RT			3	6.2E-1	1.0E0
☐	INTERPRO	ATPase, P-type cation-transporter, N-terminal	RT			3	6.2E-1	1.0E0
☐	INTERPRO	ATPase, F0/V0 complex, subunit C	RT			3	6.2E-1	1.0E0
☐	GOTERM_BP_FAT	transmembrane transport	RT			13	6.4E-1	1.0E0
☐	GOTERM_BP_FAT	ion transmembrane transport	RT			9	6.5E-1	1.0E0
☐	GOTERM_BP_FAT	monovalent inorganic cation transport	RT			10	6.7E-1	1.0E0
☐	GOTERM_MF_FAT	inorganic cation transmembrane transporter activity	RT			11	6.9E-1	1.0E0
☐	KEGG_PATHWAY	Oxidative phosphorylation	RT			8	7.0E-1	1.0E0
☐	GOTERM_BP_FAT	oxidative phosphorylation	RT			8	7.3E-1	1.0E0
☐	GOTERM_BP_FAT	energy coupled proton transport, down electrochemical gradient	RT			8	7.3E-1	1.0E0
☐	GOTERM_BP_FAT	ATP synthesis coupled proton transport	RT			8	7.3E-1	1.0E0
☐	GOTERM_MF_FAT	hydrogen ion transmembrane transporter activity	RT			9	7.5E-1	1.0E0
☐	GOTERM_MF_FAT	monovalent inorganic cation transmembrane transporter activity	RT			9	7.5E-1	1.0E0
☐	GOTERM_MF_FAT	hydrolase activity, acting on acid anhydrides, catalyzing transmembrane movement of substances	RT			17	7.8E-1	1.0E0
☐	INTERPRO	Haloacid dehalogenase-like hydrolase	RT			6	8.0E-1	1.0E0
☐	GOTERM_MF_FAT	cation-transporting ATPase activity	RT			6	8.1E-1	1.0E0
☐	GOTERM_MF_FAT	ATPase activity, coupled to movement of substances	RT			15	8.6E-1	1.0E0
☐	GOTERM_MF_FAT	ATPase activity, coupled to transmembrane movement of substances	RT			15	8.6E-1	1.0E0
☐	GOTERM_MF_FAT	primary active transmembrane transporter activity	RT			16	8.7E-1	1.0E0
☐	GOTERM_MF_FAT	P-P-bond-hydrolysis-driven transmembrane transporter activity	RT			16	8.7E-1	1.0E0
☐	GOTERM_CC_FAT	proton-transporting two-sector ATPase complex, catalytic domain	RT			4	9.3E-1	1.0E0
☐	GOTERM_MF_FAT	proton-transporting ATPase activity, rotational mechanism	RT			3	9.8E-1	1.0E0
	Annotation Cluster 16	Enrichment Score: 0.29	G			Count	P_Value	Benjamini
☐	GOTERM_BP_FAT	alcohol catabolic process	RT			10	4.4E-1	1.0E0
☐	GOTERM_BP_FAT	glucose catabolic process	RT			10	4.4E-1	1.0E0
☐	GOTERM_BP_FAT	carbohydrate catabolic process	RT			10	4.4E-1	1.0E0
☐	GOTERM_BP_FAT	monosaccharide catabolic process	RT			10	4.4E-1	1.0E0
☐	GOTERM_BP_FAT	hexose catabolic process	RT			10	4.4E-1	1.0E0
☐	GOTERM_BP_FAT	cellular carbohydrate catabolic process	RT			10	4.4E-1	1.0E0
☐	GOTERM_BP_FAT	generation of precursor metabolites and energy	RT			17	4.5E-1	1.0E0
☐	GOTERM_BP_FAT	glycolysis	RT			8	4.9E-1	1.0E0
☐	SP_PIR_KEYWORDS	glycolysis	RT			5	5.0E-1	9.6E-1
☐	GOTERM_BP_FAT	monosaccharide metabolic process	RT			14	6.6E-1	1.0E0
☐	GOTERM_BP_FAT	glucose metabolic process	RT			11	8.4E-1	1.0E0
☐	GOTERM_BP_FAT	hexose metabolic process	RT			11	8.4E-1	1.0E0
	Annotation Cluster 17	Enrichment Score: 0.29	G			Count	P_Value	Benjamini
☐	SMART	Pept_C1	RT			18	3.6E-1	1.0E0
☐	GOTERM_MF_FAT	peptidase activity	RT			49	3.8E-1	1.0E0
☐	INTERPRO	Peptidase C1A, papain C-terminal	RT			18	3.8E-1	1.0E0
☐	INTERPRO	Peptidase C1A, papain	RT			18	3.8E-1	1.0E0
☐	GOTERM_MF_FAT	cysteine-type peptidase activity	RT			19	5.1E-1	1.0E0
☐	GOTERM_MF_FAT	cysteine-type endopeptidase activity	RT			15	5.9E-1	1.0E0
☐	GOTERM_MF_FAT	peptidase activity, acting on L-amino acid peptides	RT			40	6.2E-1	1.0E0
		Peptidase, cysteine peptidase active site	RT			18	6.4E-1	1.0E0

INTERPRO					
SP_PIR_KEYWORDS	Protease	RT	21	6.8E-1	9.8E-1
GOTERM_MF_FAT	endopeptidase activity	RT	25	8.5E-1	1.0E0
Annotation Cluster 18	Enrichment Score: 0.28		Count	P_Value	Benjamini
GOTERM_CC_FAT	cytosolic part	RT	7	3.9E-1	1.0E0
GOTERM_CC_FAT	prefoldin complex	RT	5	4.7E-1	1.0E0
INTERPRO	Prefoldin beta-like	RT	3	6.2E-1	1.0E0
GOTERM_MF_FAT	unfolded protein binding	RT	13	6.4E-1	1.0E0
Annotation Cluster 19	Enrichment Score: 0.28		Count	P_Value	Benjamini
INTERPRO	Cytochrome b5	RT	5	3.1E-1	1.0E0
INTERPRO	Cytochrome b5, heme-binding site	RT	4	4.4E-1	1.0E0
SP_PIR_KEYWORDS	heme	RT	4	6.5E-1	9.8E-1
GOTERM_MF_FAT	heme binding	RT	5	6.8E-1	1.0E0
GOTERM_MF_FAT	tetrapyrrole binding	RT	5	6.8E-1	1.0E0
Annotation Cluster 20	Enrichment Score: 0.27		Count	P_Value	Benjamini
SP_PIR_KEYWORDS	membrane	RT	18	3.7E-1	9.7E-1
SP_PIR_KEYWORDS	transmembrane	RT	19	4.8E-1	9.6E-1
GOTERM_CC_FAT	integral to membrane	RT	37	6.8E-1	1.0E0
GOTERM_CC_FAT	intrinsic to membrane	RT	37	6.8E-1	1.0E0
Annotation Cluster 21	Enrichment Score: 0.26		Count	P_Value	Benjamini
INTERPRO	ATPase, P-type, ATPase-associated region	RT	9	2.6E-1	1.0E0
INTERPRO	ATPase, P-type phosphorylation site	RT	7	2.8E-1	1.0E0
INTERPRO	ATPase, P-type, K/Mg/Cd/Cu/Zn/Na/Ca/Na/H-transporter	RT	9	3.9E-1	1.0E0
GOTERM_MF_FAT	ATPase activity, coupled to transmembrane movement of ions, phosphorylative mechanism	RT	9	3.9E-1	1.0E0
GOTERM_MF_FAT	lipid transporter activity	RT	5	5.1E-1	1.0E0
GOTERM_MF_FAT	phospholipid-translocating ATPase activity	RT	4	6.5E-1	1.0E0
GOTERM_MF_FAT	phospholipid transporter activity	RT	4	6.5E-1	1.0E0
GOTERM_MF_FAT	aminophospholipid transporter activity	RT	4	6.5E-1	1.0E0
GOTERM_BP_FAT	lipid transport	RT	4	6.6E-1	1.0E0
GOTERM_BP_FAT	lipid localization	RT	4	6.6E-1	1.0E0
INTERPRO	ATPase, P-type, phospholipid-translocating, flippase	RT	3	8.0E-1	1.0E0
GOTERM_BP_FAT	phospholipid transport	RT	3	8.0E-1	1.0E0
GOTERM_MF_FAT	magnesium ion binding	RT	8	8.8E-1	1.0E0
Annotation Cluster 22	Enrichment Score: 0.26		Count	P_Value	Benjamini
INTERPRO	DNA/RNA helicase, DEAD/DEAH box type, N-terminal	RT	20	3.5E-1	1.0E0
SP_PIR_KEYWORDS	helicase	RT	28	3.8E-1	9.6E-1
GOTERM_MF_FAT	purine NTP-dependent helicase activity	RT	24	4.6E-1	1.0E0
GOTERM_MF_FAT	ATP-dependent helicase activity	RT	24	4.6E-1	1.0E0
GOTERM_MF_FAT	ATPase activity	RT	56	5.4E-1	1.0E0
GOTERM_MF_FAT	ATPase activity, coupled	RT	42	5.4E-1	1.0E0
INTERPRO	RNA helicase, DEAD-box type, Q motif	RT	11	5.8E-1	1.0E0
SMART	HELICc	RT	26	6.1E-1	1.0E0
GOTERM_MF_FAT	helicase activity	RT	32	6.2E-1	1.0E0
INTERPRO	DNA/RNA helicase, C-terminal	RT	26	6.4E-1	1.0E0
INTERPRO	Helicase, superfamily 1 and 2, ATP-binding	RT	25	6.9E-1	1.0E0
SMART	DEXDc	RT	24	7.7E-1	1.0E0
INTERPRO	DEAD-like helicase, N-terminal	RT	24	7.9E-1	1.0E0
Annotation Cluster 23	Enrichment Score: 0.25		Count	P_Value	Benjamini
INTERPRO	Thioredoxin-like	RT	10	3.1E-1	1.0E0
GOTERM_BP_FAT	cellular homeostasis	RT	13	4.3E-1	1.0E0
INTERPRO	Thioredoxin domain	RT	5	5.1E-1	1.0E0
INTERPRO	Thioredoxin, conserved site	RT	5	5.1E-1	1.0E0
GOTERM_BP_FAT	cell redox homeostasis	RT	12	5.1E-1	1.0E0
GOTERM_BP_FAT	homeostatic process	RT	13	5.4E-1	1.0E0
INTERPRO	Thioredoxin fold	RT	14	5.6E-1	1.0E0
INTERPRO	Thioredoxin-like subdomain	RT	4	6.5E-1	1.0E0
INTERPRO	Thioredoxin, core	RT	5	6.8E-1	1.0E0

Category	Term		Count	P_Value	Benjamini
INTERPRO	Alkyl hydroperoxide reductase/ Thiol specific antioxidant/ Mal allergen	RT	4	8.0E-1	1.0E0
GOTERM_MF_FAT	antioxidant activity	RT	4	8.9E-1	1.0E0
Annotation Cluster 24	Enrichment Score: 0.25		Count	P_Value	Benjamini
GOTERM_CC_FAT	microtubule associated complex	RT	10	5.0E-1	1.0E0
GOTERM_CC_FAT	dynein complex	RT	8	5.7E-1	1.0E0
INTERPRO	Dynein heavy chain	RT	8	6.2E-1	1.0E0
Annotation Cluster 25	Enrichment Score: 0.24		Count	P_Value	Benjamini
GOTERM_CC_FAT	endomembrane system	RT	11	4.1E-1	1.0E0
GOTERM_CC_FAT	pore complex	RT	4	6.2E-1	1.0E0
GOTERM_CC_FAT	envelope	RT	4	6.2E-1	1.0E0
GOTERM_CC_FAT	organelle envelope	RT	4	6.2E-1	1.0E0
GOTERM_CC_FAT	nuclear pore	RT	4	6.2E-1	1.0E0
GOTERM_CC_FAT	nuclear envelope	RT	4	6.2E-1	1.0E0
Annotation Cluster 26	Enrichment Score: 0.24		Count	P_Value	Benjamini
GOTERM_MF_FAT	acid-amino acid ligase activity	RT	19	2.5E-1	1.0E0
GOTERM_MF_FAT	ligase activity, forming carbon-nitrogen bonds	RT	22	2.6E-1	1.0E0
SP_PIR_KEYWORDS	ubl conjugation pathway	RT	10	3.0E-1	9.9E-1
GOTERM_MF_FAT	small conjugating protein ligase activity	RT	10	3.1E-1	1.0E0
GOTERM_BP_FAT	proteolysis	RT	55	3.2E-1	1.0E0
SMART	UBCc	RT	9	3.7E-1	1.0E0
INTERPRO	Ubiquitin-conjugating enzyme, E2	RT	9	3.9E-1	1.0E0
INTERPRO	Ubiquitin-conjugating enzyme/RWD-like	RT	9	3.9E-1	1.0E0
GOTERM_BP_FAT	modification-dependent macromolecule catabolic process	RT	17	4.5E-1	1.0E0
GOTERM_BP_FAT	modification-dependent protein catabolic process	RT	17	4.5E-1	1.0E0
GOTERM_BP_FAT	macromolecule catabolic process	RT	24	5.4E-1	1.0E0
GOTERM_BP_FAT	cellular macromolecule catabolic process	RT	21	5.5E-1	1.0E0
GOTERM_BP_FAT	protein catabolic process	RT	23	6.0E-1	1.0E0
GOTERM_BP_FAT	cellular protein catabolic process	RT	20	6.1E-1	1.0E0
GOTERM_BP_FAT	proteolysis involved in cellular protein catabolic process	RT	20	6.1E-1	1.0E0
GOTERM_CC_FAT	cytosol	RT	17	6.3E-1	1.0E0
PIR_SUPERFAMILY	PIRSF001567:ubiquitin-protein ligase E2	RT	4	7.9E-1	1.0E0
GOTERM_BP_FAT	ubiquitin-dependent protein catabolic process	RT	8	8.2E-1	1.0E0
INTERPRO	Proteasome, alpha and beta subunits	RT	7	8.8E-1	1.0E0
INTERPRO	Proteasome, alpha-subunit, conserved site	RT	4	8.9E-1	1.0E0
GOTERM_CC_FAT	proteasome core complex	RT	7	9.1E-1	1.0E0
KEGG_PATHWAY	Proteasome	RT	14	9.2E-1	1.0E0
SP_PIR_KEYWORDS	proteasome	RT	10	9.2E-1	1.0E0
INTERPRO	Proteasome, subunit alpha/beta	RT	7	9.2E-1	1.0E0
GOTERM_MF_FAT	threonine-type peptidase activity	RT	7	9.3E-1	1.0E0
GOTERM_MF_FAT	threonine-type endopeptidase activity	RT	7	9.3E-1	1.0E0
GOTERM_CC_FAT	proteasome complex	RT	10	9.3E-1	1.0E0
PIR_SUPERFAMILY	PIRSF001212:multicatalytic endopeptidase complex chain C9	RT	3	9.5E-1	1.0E0
Annotation Cluster 27	Enrichment Score: 0.23		Count	P_Value	Benjamini
INTERPRO	DNA-directed DNA polymerase, family B, conserved site	RT	4	4.4E-1	1.0E0
INTERPRO	DNA-directed DNA polymerase, family B, conserved region2	RT	4	4.4E-1	1.0E0
SP_PIR_KEYWORDS	dna replication	RT	7	5.8E-1	9.8E-1
SMART	POLBc	RT	4	6.4E-1	1.0E0
SP_PIR_KEYWORDS	dna-directed dna polymerase	RT	4	6.5E-1	9.8E-1
INTERPRO	DNA-directed DNA polymerase, family B	RT	4	6.5E-1	1.0E0
GOTERM_MF_FAT	DNA-directed DNA polymerase activity	RT	6	8.1E-1	1.0E0
Annotation Cluster 28	Enrichment Score: 0.22		Count	P_Value	Benjamini
SMART	ARF	RT	4	4.4E-1	1.0E0
INTERPRO	ADP-ribosylation factor	RT	4	4.4E-1	1.0E0
PIR_SUPERFAMILY	PIRSF001711:ADP-ribosylation factor	RT	4	6.4E-1	1.0E0
GOTERM_BP_FAT	small GTPase mediated signal transduction	RT	10	6.7E-1	1.0E0
INTERPRO	ARF/SAR superfamily	RT	5	6.8E-1	1.0E0

INTERPRO	Small GTP-binding protein	RT	11	8.4E-1	1.0E0
Annotation Cluster 29	Enrichment Score: 0.22		Count	P_Value	Benjamini
GOTERM_BP_FAT	regulation of transcription	RT	21	3.1E-1	1.0E0
GOTERM_CC_FAT	nucleoplasm part	RT	3	6.0E-1	1.0E0
GOTERM_CC_FAT	nucleoplasm	RT	3	6.0E-1	1.0E0
GOTERM_CC_FAT	transcription factor complex	RT	3	6.0E-1	1.0E0
GOTERM_CC_FAT	intracellular organelle lumen	RT	4	6.2E-1	1.0E0
GOTERM_CC_FAT	nuclear lumen	RT	4	6.2E-1	1.0E0
GOTERM_CC_FAT	membrane-enclosed lumen	RT	4	6.2E-1	1.0E0
GOTERM_CC_FAT	organelle lumen	RT	4	6.2E-1	1.0E0
GOTERM_BP_FAT	regulation of transcription, DNA-dependent	RT	7	8.1E-1	1.0E0
GOTERM_BP_FAT	regulation of RNA metabolic process	RT	7	8.1E-1	1.0E0
Annotation Cluster 30	Enrichment Score: 0.21		Count	P_Value	Benjamini
GOTERM_CC_FAT	organelle membrane	RT	8	4.3E-1	1.0E0
GOTERM_CC_FAT	endoplasmic reticulum membrane	RT	3	6.0E-1	1.0E0
GOTERM_CC_FAT	endoplasmic reticulum part	RT	3	6.0E-1	1.0E0
GOTERM_CC_FAT	nuclear envelope-endoplasmic reticulum network	RT	3	6.0E-1	1.0E0
GOTERM_CC_FAT	endoplasmic reticulum	RT	3	9.4E-1	1.0E0
Annotation Cluster 31	Enrichment Score: 0.21		Count	P_Value	Benjamini
GOTERM_CC_FAT	ribonucleoprotein complex	RT	58	4.4E-1	1.0E0
GOTERM_CC_FAT	intracellular non-membrane-bounded organelle	RT	90	5.5E-1	1.0E0
GOTERM_CC_FAT	non-membrane-bounded organelle	RT	90	5.5E-1	1.0E0
GOTERM_CC_FAT	ribosome	RT	43	5.8E-1	1.0E0
SP_PIR_KEYWORDS	ribosomal protein	RT	42	6.1E-1	9.8E-1
KEGG_PATHWAY	Ribosome	RT	39	6.5E-1	1.0E0
GOTERM_BP_FAT	translation	RT	64	7.3E-1	1.0E0
GOTERM_MF_FAT	structural molecule activity	RT	47	7.5E-1	1.0E0
GOTERM_MF_FAT	structural constituent of ribosome	RT	38	8.3E-1	1.0E0
Annotation Cluster 32	Enrichment Score: 0.19		Count	P_Value	Benjamini
PIR_SUPERFAMILY	PIRSF005902:DNase_TatD	RT	3	6.1E-1	1.0E0
INTERPRO	Deoxyribonuclease, TatD Mg-dependent	RT	3	6.2E-1	1.0E0
INTERPRO	Deoxyribonuclease, TatD-related	RT	3	6.2E-1	1.0E0
INTERPRO	Deoxyribonuclease, TatD	RT	3	6.2E-1	1.0E0
GOTERM_MF_FAT	endodeoxyribonuclease activity, producing 5'-phosphomonoesters	RT	3	6.2E-1	1.0E0
GOTERM_MF_FAT	deoxyribonuclease activity	RT	3	6.2E-1	1.0E0
GOTERM_MF_FAT	endodeoxyribonuclease activity	RT	3	6.2E-1	1.0E0
GOTERM_MF_FAT	endonuclease activity, active with either ribo- or deoxyribonucleic acids and producing 5'-phosphomonoesters	RT	4	8.9E-1	1.0E0
Annotation Cluster 33	Enrichment Score: 0.18		Count	P_Value	Benjamini
INTERPRO	4Fe-4S ferredoxin, iron-sulpur binding domain	RT	7	4.4E-1	1.0E0
INTERPRO	4Fe-4S ferredoxin, iron-sulphur binding, subgroup	RT	7	4.4E-1	1.0E0
GOTERM_MF_FAT	electron carrier activity	RT	14	6.6E-1	1.0E0
INTERPRO	4Fe-4S ferredoxin, iron-sulphur binding, conserved site	RT	5	6.8E-1	1.0E0
GOTERM_MF_FAT	metal cluster binding	RT	11	8.4E-1	1.0E0
GOTERM_MF_FAT	iron-sulfur cluster binding	RT	11	8.4E-1	1.0E0
GOTERM_MF_FAT	4 iron, 4 sulfur cluster binding	RT	4	9.4E-1	1.0E0
Annotation Cluster 34	Enrichment Score: 0.16		Count	P_Value	Benjamini
SMART	Sm	RT	5	6.6E-1	1.0E0
INTERPRO	Like-Sm ribonucleoprotein, eukaryotic and archaea-type, core	RT	5	6.8E-1	1.0E0
INTERPRO	Like-Sm ribonucleoprotein, core	RT	7	7.1E-1	1.0E0
Annotation Cluster 35	Enrichment Score: 0.16		Count	P_Value	Benjamini
GOTERM_BP_FAT	phospholipid biosynthetic process	RT	6	3.9E-1	1.0E0
GOTERM_BP_FAT	organophosphate metabolic process	RT	9	5.3E-1	1.0E0
GOTERM_BP_FAT	phospholipid metabolic process	RT	8	6.2E-1	1.0E0
GOTERM_BP_FAT	lipid biosynthetic process	RT	8	8.2E-1	1.0E0
GOTERM_BP_FAT	glycerolipid metabolic process	RT	4	8.9E-1	1.0E0
GOTERM_BP_FAT	glycerophospholipid metabolic process	RT	4	8.9E-1	1.0E0

GOTERM_BP_FAT	phosphoinositide metabolic process	RT	4	8.9E-1	1.0E0
Annotation Cluster 36	Enrichment Score: 0.16		Count	P_Value	Benjamini
SMART	ANK	RT	250	5.9E-1	1.0E0
INTERPRO	Ankyrin	RT	250	6.7E-1	1.0E0
SP_PIR_KEYWORDS	ank repeat	RT	170	8.5E-1	9.9E-1
Annotation Cluster 37	Enrichment Score: 0.16		Count	P_Value	Benjamini
INTERPRO	Adenylate kinase	RT	3	6.2E-1	1.0E0
GOTERM_MF_FAT	adenylate kinase activity	RT	3	6.2E-1	1.0E0
GOTERM_MF_FAT	nucleotide kinase activity	RT	4	6.5E-1	1.0E0
GOTERM_MF_FAT	phosphotransferase activity, phosphate group as acceptor	RT	5	6.8E-1	1.0E0
GOTERM_MF_FAT	nucleobase, nucleoside, nucleotide kinase activity	RT	5	9.3E-1	1.0E0
Annotation Cluster 38	Enrichment Score: 0.15		Count	P_Value	Benjamini
INTERPRO	WD40 repeat, conserved site	RT	18	5.6E-1	1.0E0
SP_PIR_KEYWORDS	wd repeat	RT	29	6.0E-1	9.8E-1
INTERPRO	WD40 repeat, subgroup	RT	33	6.2E-1	1.0E0
INTERPRO	WD40 repeat 2	RT	25	6.9E-1	1.0E0
INTERPRO	WD40 repeat, region	RT	27	7.1E-1	1.0E0
SMART	WD40	RT	40	8.4E-1	1.0E0
INTERPRO	WD40 repeat	RT	40	8.6E-1	1.0E0
INTERPRO	WD40/YVTN repeat-like	RT	38	9.0E-1	1.0E0
Annotation Cluster 39	Enrichment Score: 0.14		Count	P_Value	Benjamini
GOTERM_BP_FAT	amine biosynthetic process	RT	5	5.1E-1	1.0E0
GOTERM_BP_FAT	cellular amino acid biosynthetic process	RT	4	6.6E-1	1.0E0
GOTERM_BP_FAT	organic acid biosynthetic process	RT	4	8.9E-1	1.0E0
GOTERM_BP_FAT	carboxylic acid biosynthetic process	RT	4	8.9E-1	1.0E0
Annotation Cluster 40	Enrichment Score: 0.13		Count	P_Value	Benjamini
GOTERM_MF_FAT	thiolester hydrolase activity	RT	6	5.6E-1	1.0E0
INTERPRO	Peptidase C19, ubiquitin carboxyl-terminal hydrolase 2	RT	4	8.0E-1	1.0E0
GOTERM_MF_FAT	ubiquitin thiolesterase activity	RT	4	8.0E-1	1.0E0
GOTERM_BP_FAT	ubiquitin-dependent protein catabolic process	RT	8	8.2E-1	1.0E0
Annotation Cluster 41	Enrichment Score: 0.13		Count	P_Value	Benjamini
GOTERM_MF_FAT	unfolded protein binding	RT	13	6.4E-1	1.0E0
GOTERM_BP_FAT	protein folding	RT	17	7.8E-1	1.0E0
SP_PIR_KEYWORDS	Chaperone	RT	7	8.0E-1	9.9E-1
Annotation Cluster 42	Enrichment Score: 0.13		Count	P_Value	Benjamini
SMART	VWA	RT	5	6.6E-1	1.0E0
INTERPRO	von Willebrand factor, type A	RT	5	6.8E-1	1.0E0
INTERPRO	Copine	RT	3	9.0E-1	1.0E0
Annotation Cluster 43	Enrichment Score: 0.12		Count	P_Value	Benjamini
SP_PIR_KEYWORDS	Acyltransferase	RT	8	4.7E-1	9.6E-1
INTERPRO	Acyl-CoA N-acyltransferase	RT	6	8.0E-1	1.0E0
GOTERM_MF_FAT	N-acyltransferase activity	RT	6	8.1E-1	1.0E0
GOTERM_MF_FAT	acetyltransferase activity	RT	6	8.1E-1	1.0E0
INTERPRO	GCN5-related N-acetyltransferase	RT	5	8.8E-1	1.0E0
GOTERM_MF_FAT	N-acetyltransferase activity	RT	5	8.8E-1	1.0E0
Annotation Cluster 44	Enrichment Score: 0.12		Count	P_Value	Benjamini
INTERPRO	EGF-like, type 3	RT	20	5.2E-1	1.0E0
SMART	EGF_Lam	RT	13	9.2E-1	1.0E0
INTERPRO	EGF-like, laminin	RT	13	9.3E-1	1.0E0
Annotation Cluster 45	Enrichment Score: 0.12		Count	P_Value	Benjamini
INTERPRO	Molecular chaperone, heat shock protein, Hsp40, DnaJ	RT	5	6.8E-1	1.0E0
SMART	DnaJ	RT	5	7.9E-1	1.0E0
INTERPRO	Heat shock protein DnaJ, N-terminal	RT	5	8.0E-1	1.0E0
GOTERM_MF_FAT	heat shock protein binding	RT	5	8.0E-1	1.0E0
Annotation Cluster 46	Enrichment Score: 0.11		Count	P_Value	Benjamini
GOTERM_BP_FAT	translational elongation	RT	5	6.8E-1	1.0E0
GOTERM_MF_FAT	translation elongation factor activity	RT	5	8.0E-1	1.0E0
SP_PIR_KEYWORDS	elongation factor	RT	4	8.8E-1	9.9E-1

						Count	P_Value	Benjamini
	Annotation Cluster 47	Enrichment Score: 0.1				Count	P_Value	Benjamini
	PIR_SUPERFAMILY	PIRSF002306:tubulin	RT			5	6.6E-1	1.0E0
	INTERPRO	Tubulin/FtsZ, 2-layer sandwich domain	RT			5	6.8E-1	1.0E0
	GOTERM_BP_FAT	protein complex assembly	RT			8	7.3E-1	1.0E0
	GOTERM_BP_FAT	protein complex biogenesis	RT			8	7.3E-1	1.0E0
	INTERPRO	Beta tubulin, autoregulation binding site	RT			4	8.0E-1	1.0E0
	INTERPRO	Tubulin/FtsZ, GTPase domain	RT			5	8.0E-1	1.0E0
	INTERPRO	Tubulin, conserved site	RT			5	8.0E-1	1.0E0
	INTERPRO	Tubulin	RT			5	8.0E-1	1.0E0
	GOTERM_BP_FAT	protein polymerization	RT			5	8.0E-1	1.0E0
	GOTERM_BP_FAT	macromolecular complex subunit organization	RT			15	8.2E-1	1.0E0
	GOTERM_BP_FAT	macromolecular complex assembly	RT			14	8.6E-1	1.0E0
	GOTERM_BP_FAT	cellular protein complex assembly	RT			6	8.8E-1	1.0E0
	GOTERM_MF_FAT	GTPase activity	RT			9	8.8E-1	1.0E0
	GOTERM_BP_FAT	cellular macromolecular complex subunit organization	RT			13	9.0E-1	1.0E0
	GOTERM_BP_FAT	cellular macromolecular complex assembly	RT			12	9.3E-1	1.0E0
	Annotation Cluster 48	Enrichment Score: 0.08				Count	P_Value	Benjamini
	GOTERM_MF_FAT	exopeptidase activity	RT			7	7.2E-1	1.0E0
	GOTERM_MF_FAT	metallopeptidase activity	RT			7	8.8E-1	1.0E0
	GOTERM_MF_FAT	metalloexopeptidase activity	RT			4	8.9E-1	1.0E0
	Annotation Cluster 49	Enrichment Score: 0.08				Count	P_Value	Benjamini
	INTERPRO	RNA-directed DNA polymerase (reverse transcriptase)	RT			5	6.8E-1	1.0E0
	GOTERM_BP_FAT	RNA-dependent DNA replication	RT			5	6.8E-1	1.0E0
	INTERPRO	RNA-directed DNA polymerase (reverse transcriptase), related	RT			3	9.0E-1	1.0E0
	GOTERM_MF_FAT	RNA-directed DNA polymerase activity	RT			11	9.7E-1	1.0E0
	SP_PIR_KEYWORDS	rna-directed dna polymerase	RT			10	9.8E-1	1.0E0
	Annotation Cluster 50	Enrichment Score: 0.07				Count	P_Value	Benjamini
	SMART	RRM	RT			8	8.0E-1	1.0E0
	INTERPRO	RNA recognition motif, RNP-1	RT			8	8.2E-1	1.0E0
	INTERPRO	Nucleotide-binding, alpha-beta plait	RT			8	9.2E-1	1.0E0
	Annotation Cluster 51	Enrichment Score: 0.07				Count	P_Value	Benjamini
	GOTERM_BP_FAT	intracellular signaling cascade	RT			13	6.4E-1	1.0E0
	GOTERM_BP_FAT	small GTPase mediated signal transduction	RT			10	6.7E-1	1.0E0
	SP_PIR_KEYWORDS	gtp-binding	RT			22	8.0E-1	9.9E-1
	INTERPRO	Small GTP-binding protein	RT			11	8.4E-1	1.0E0
	INTERPRO	Ras GTPase	RT			7	8.8E-1	1.0E0
	GOTERM_MF_FAT	GTPase activity	RT			9	8.8E-1	1.0E0
	PIR_SUPERFAMILY	PIRSF001710:Ras-related protein Rab	RT			3	9.5E-1	1.0E0
	GOTERM_MF_FAT	GTP binding	RT			24	9.5E-1	1.0E0
	GOTERM_MF_FAT	guanyl ribonucleotide binding	RT			24	9.5E-1	1.0E0
	GOTERM_MF_FAT	guanyl nucleotide binding	RT			24	9.5E-1	1.0E0
	INTERPRO	Ras	RT			5	9.8E-1	1.0E0
	Annotation Cluster 52	Enrichment Score: 0.07				Count	P_Value	Benjamini
	KEGG_PATHWAY	DNA replication	RT			12	7.5E-1	1.0E0
	KEGG_PATHWAY	Nucleotide excision repair	RT			8	9.1E-1	1.0E0
	KEGG_PATHWAY	Mismatch repair	RT			7	9.2E-1	1.0E0
	Annotation Cluster 53	Enrichment Score: 0.06				Count	P_Value	Benjamini
	SP_PIR_KEYWORDS	iron	RT			12	6.9E-1	9.8E-1
	GOTERM_MF_FAT	iron ion binding	RT			18	7.2E-1	1.0E0
	SMART	PP2Ac	RT			8	8.7E-1	1.0E0
	INTERPRO	Serine/threonine-specific protein phosphatase and bis(5-nucleosyl)-tetraphosphatase	RT			8	8.8E-1	1.0E0
	INTERPRO	Metallophosphoesterase	RT			11	8.9E-1	1.0E0
	PIR_SUPERFAMILY	PIRSF000908:serine/threonine-protein phosphatase, conventional type	RT			4	9.4E-1	1.0E0
	GOTERM_MF_FAT	phosphoprotein phosphatase activity	RT			11	1.0E0	1.0E0
	GOTERM_MF_FAT	phosphatase activity	RT			18	1.0E0	1.0E0
	Annotation Cluster 54	Enrichment Score: 0.06				Count	P_Value	Benjamini
	GOTERM_CC_FAT	organelle membrane	RT			8	4.3E-1	1.0E0

	GOTERM_CC_FAT	membrane coat	RT			8	7.8E-1	1.0E0
	GOTERM_CC_FAT	coated membrane	RT			8	7.8E-1	1.0E0
	GOTERM_CC_FAT	transport vesicle membrane	RT			3	8.8E-1	1.0E0
	GOTERM_CC_FAT	transport vesicle	RT			3	8.8E-1	1.0E0
	INTERPRO	Clathrin/coatomer adaptor, adaptin-like, N-terminal	RT			3	9.0E-1	1.0E0
	GOTERM_CC_FAT	membrane-bounded vesicle	RT			3	9.4E-1	1.0E0
	GOTERM_CC_FAT	vesicle	RT			3	9.4E-1	1.0E0
	GOTERM_CC_FAT	cytoplasmic vesicle	RT			3	9.4E-1	1.0E0
	GOTERM_CC_FAT	coated vesicle membrane	RT			3	9.4E-1	1.0E0
	GOTERM_CC_FAT	cytoplasmic vesicle membrane	RT			3	9.4E-1	1.0E0
	GOTERM_CC_FAT	vesicle coat	RT			3	9.4E-1	1.0E0
	GOTERM_CC_FAT	cytoplasmic vesicle part	RT			3	9.4E-1	1.0E0
	GOTERM_CC_FAT	coated vesicle	RT			3	9.4E-1	1.0E0
	GOTERM_CC_FAT	cytoplasmic membrane-bounded vesicle	RT			3	9.4E-1	1.0E0
	GOTERM_CC_FAT	vesicle membrane	RT			3	9.4E-1	1.0E0
	GOTERM_CC_FAT	Golgi apparatus part	RT			3	9.9E-1	1.0E0
	GOTERM_CC_FAT	Golgi apparatus	RT			3	1.0E0	1.0E0
	Annotation Cluster 55	Enrichment Score: 0.05	G			Count	P_Value	Benjamini
	GOTERM_CC_FAT	membrane coat	RT			8	7.8E-1	1.0E0
	GOTERM_CC_FAT	coated membrane	RT			8	7.8E-1	1.0E0
	GOTERM_BP_FAT	establishment of protein localization	RT			18	8.4E-1	1.0E0
	GOTERM_BP_FAT	protein transport	RT			18	8.4E-1	1.0E0
	GOTERM_BP_FAT	protein localization	RT			18	8.8E-1	1.0E0
	GOTERM_BP_FAT	intracellular transport	RT			14	9.3E-1	1.0E0
	GOTERM_BP_FAT	intracellular protein transport	RT			11	9.5E-1	1.0E0
	GOTERM_BP_FAT	cellular macromolecule localization	RT			11	9.5E-1	1.0E0
	GOTERM_BP_FAT	cellular protein localization	RT			11	9.5E-1	1.0E0
	GOTERM_BP_FAT	vesicle-mediated transport	RT			14	9.5E-1	1.0E0
	Annotation Cluster 56	Enrichment Score: 0.05	G			Count	P_Value	Benjamini
	GOTERM_MF_FAT	cation binding	RT			146	7.8E-1	1.0E0
	GOTERM_MF_FAT	ion binding	RT			146	7.8E-1	1.0E0
	GOTERM_MF_FAT	metal ion binding	RT			144	7.9E-1	1.0E0
	SP_PIR_KEYWORDS	metal-binding	RT			54	8.7E-1	1.0E0
	SP_PIR_KEYWORDS	zinc	RT			45	8.8E-1	9.9E-1
	GOTERM_MF_FAT	transition metal ion binding	RT			101	9.4E-1	1.0E0
	INTERPRO	Zinc finger, C3HC4 RING-type	RT			17	9.5E-1	1.0E0
	GOTERM_MF_FAT	zinc ion binding	RT			80	9.5E-1	1.0E0
	SP_PIR_KEYWORDS	zinc-finger	RT			30	9.6E-1	1.0E0
	SMART	RING	RT			33	9.6E-1	1.0E0
	INTERPRO	Zinc finger, RING-type	RT			33	9.7E-1	1.0E0
	Annotation Cluster 57	Enrichment Score: 0.05	G			Count	P_Value	Benjamini
	INTERPRO	ATPase, AAA-type, core	RT			14	8.0E-1	1.0E0
	SMART	AAA	RT			36	9.4E-1	1.0E0
	INTERPRO	ATPase, AAA+ type, core	RT			36	9.5E-1	1.0E0
	Annotation Cluster 58	Enrichment Score: 0.05	G			Count	P_Value	Benjamini
	INTERPRO	Aminoacyl-tRNA synthetase, class I, conserved site	RT			6	7.0E-1	1.0E0
	SP_PIR_KEYWORDS	ligase	RT			31	7.4E-1	9.9E-1
	SP_PIR_KEYWORDS	protein biosynthesis	RT			6	8.0E-1	9.9E-1
	GOTERM_BP_FAT	tRNA aminoacylation for protein translation	RT			11	9.3E-1	1.0E0
	GOTERM_MF_FAT	aminoacyl-tRNA ligase activity	RT			12	9.3E-1	1.0E0
	GOTERM_MF_FAT	ligase activity, forming carbon-oxygen bonds	RT			12	9.3E-1	1.0E0
	GOTERM_MF_FAT	ligase activity, forming aminoacyl-tRNA and related compounds	RT			12	9.3E-1	1.0E0
	KEGG_PATHWAY	Aminoacyl-tRNA biosynthesis	RT			10	9.4E-1	1.0E0
	SP_PIR_KEYWORDS	Aminoacyl-tRNA synthetase	RT			11	9.5E-1	1.0E0
	INTERPRO	Rossmann-like alpha/beta/alpha sandwich fold	RT			9	9.5E-1	1.0E0
	GOTERM_BP_FAT	amino acid activation	RT			11	9.5E-1	1.0E0
	GOTERM_BP_FAT	tRNA aminoacylation	RT			11	9.5E-1	1.0E0

GOTERM_BP_FAT	tRNA metabolic process	RT	19	9.7E-1	1.0E0
GOTERM_BP_FAT	ncRNA metabolic process	RT	24	9.9E-1	1.0E0
Annotation Cluster 59	Enrichment Score: 0.05		Count	P_Value	Benjamini
GOTERM_BP_FAT	water-soluble vitamin biosynthetic process	RT	3	9.0E-1	1.0E0
GOTERM_BP_FAT	vitamin biosynthetic process	RT	3	9.0E-1	1.0E0
GOTERM_BP_FAT	water-soluble vitamin metabolic process	RT	3	9.0E-1	1.0E0
GOTERM_BP_FAT	vitamin metabolic process	RT	3	9.0E-1	1.0E0
Annotation Cluster 60	Enrichment Score: 0.04		Count	P_Value	Benjamini
SP_PIR_KEYWORDS	Chaperone	RT	7	8.0E-1	9.9E-1
INTERPRO	Chaperonin Cpn60/TCP-1	RT	5	8.8E-1	1.0E0
SP_PIR_KEYWORDS	cytoplasm	RT	9	9.2E-1	1.0E0
PIR_SUPERFAMILY	PIRSF002584:molecular chaperone t-complex-type	RT	4	9.4E-1	1.0E0
INTERPRO	Chaperone, tailless complex polypeptide 1	RT	4	9.4E-1	1.0E0
INTERPRO	Chaperonin TCP-1, conserved site	RT	4	9.7E-1	1.0E0
Annotation Cluster 61	Enrichment Score: 0.04		Count	P_Value	Benjamini
SMART	UBQ	RT	4	8.8E-1	1.0E0
INTERPRO	Ubiquitin	RT	4	8.9E-1	1.0E0
INTERPRO	Ubiquitin supergroup	RT	5	9.8E-1	1.0E0
Annotation Cluster 62	Enrichment Score: 0.04		Count	P_Value	Benjamini
INTERPRO	ABC transporter-like	RT	12	8.9E-1	1.0E0
INTERPRO	ABC transporter, conserved site	RT	11	9.2E-1	1.0E0
COG_ONTOLOGY	Defense mechanisms	RT	11	9.4E-1	1.0E0
Annotation Cluster 63	Enrichment Score: 0.03		Count	P_Value	Benjamini
GOTERM_BP_FAT	cytoskeleton organization	RT	9	8.3E-1	1.0E0
GOTERM_MF_FAT	lipid binding	RT	19	8.9E-1	1.0E0
SMART	ANX	RT	7	9.2E-1	1.0E0
INTERPRO	Annexin repeat	RT	7	9.2E-1	1.0E0
GOTERM_MF_FAT	calcium-dependent phospholipid binding	RT	11	9.3E-1	1.0E0
SP_PIR_KEYWORDS	annexin	RT	7	9.7E-1	1.0E0
INTERPRO	Annexin	RT	7	9.7E-1	1.0E0
INTERPRO	Alpha giardin	RT	5	9.8E-1	1.0E0
GOTERM_MF_FAT	phospholipid binding	RT	12	9.8E-1	1.0E0
Annotation Cluster 64	Enrichment Score: 0.03		Count	P_Value	Benjamini
SP_PIR_KEYWORDS	tpr repeat	RT	5	8.8E-1	1.0E0
INTERPRO	Tetratricopeptide TPR-1	RT	10	9.2E-1	1.0E0
INTERPRO	Tetratricopeptide region	RT	12	9.2E-1	1.0E0
INTERPRO	Tetratricopeptide-like helical	RT	15	9.5E-1	1.0E0
SMART	TPR	RT	11	9.6E-1	1.0E0
INTERPRO	Tetratricopeptide repeat	RT	11	9.7E-1	1.0E0
Annotation Cluster 65	Enrichment Score: 0.03		Count	P_Value	Benjamini
SP_PIR_KEYWORDS	Rotamase	RT	4	9.4E-1	1.0E0
GOTERM_MF_FAT	peptidyl-prolyl cis-trans isomerase activity	RT	4	9.4E-1	1.0E0
GOTERM_MF_FAT	cis-trans isomerase activity	RT	4	9.4E-1	1.0E0
Annotation Cluster 66	Enrichment Score: 0.02		Count	P_Value	Benjamini
GOTERM_BP_FAT	ribosome biogenesis	RT	10	9.3E-1	1.0E0
GOTERM_BP_FAT	ribonucleoprotein complex biogenesis	RT	10	9.3E-1	1.0E0
GOTERM_BP_FAT	rRNA metabolic process	RT	6	9.6E-1	1.0E0
GOTERM_BP_FAT	rRNA processing	RT	6	9.6E-1	1.0E0
GOTERM_BP_FAT	ncRNA processing	RT	13	9.8E-1	1.0E0
Annotation Cluster 67	Enrichment Score: 0.02		Count	P_Value	Benjamini
GOTERM_BP_FAT	nucleobase, nucleoside, nucleotide and nucleic acid catabolic process	RT	3	9.5E-1	1.0E0
GOTERM_BP_FAT	nucleobase, nucleoside and nucleotide catabolic process	RT	3	9.5E-1	1.0E0
GOTERM_BP_FAT	nitrogen compound catabolic process	RT	3	9.5E-1	1.0E0
Annotation Cluster 68	Enrichment Score: 0.02		Count	P_Value	Benjamini
GOTERM_BP_FAT	cofactor metabolic process	RT	6	9.3E-1	1.0E0
GOTERM_BP_FAT	coenzyme metabolic process	RT	5	9.3E-1	1.0E0
GOTERM_BP_FAT	pyridine nucleotide metabolic process	RT	3	9.5E-1	1.0E0
GOTERM_BP_FAT	oxidoreduction coenzyme metabolic process	RT	3	9.5E-1	1.0E0

GOTERM_BP_FAT	cofactor biosynthetic process	RT	4	9.7E-1	1.0E0
GOTERM_BP_FAT	coenzyme biosynthetic process	RT	3	9.8E-1	1.0E0
Annotation Cluster 69	Enrichment Score: 0.01		Count	P_Value	Benjamini
GOTERM_BP_FAT	RNA processing	RT	23	9.5E-1	1.0E0
GOTERM_BP_FAT	tRNA processing	RT	8	9.5E-1	1.0E0
GOTERM_BP_FAT	tRNA metabolic process	RT	19	9.7E-1	1.0E0
GOTERM_BP_FAT	ncRNA processing	RT	13	9.8E-1	1.0E0
GOTERM_BP_FAT	ncRNA metabolic process	RT	24	9.9E-1	1.0E0
Annotation Cluster 70	Enrichment Score: 0.01		Count	P_Value	Benjamini
GOTERM_BP_FAT	transcription	RT	14	8.6E-1	1.0E0
KEGG_PATHWAY	Purine metabolism	RT	15	9.6E-1	1.0E0
GOTERM_MF_FAT	RNA polymerase activity	RT	10	9.9E-1	1.0E0
KEGG_PATHWAY	RNA polymerase	RT	7	9.9E-1	1.0E0
GOTERM_MF_FAT	DNA-directed RNA polymerase activity	RT	9	9.9E-1	1.0E0
KEGG_PATHWAY	Pyrimidine metabolism	RT	13	1.0E0	1.0E0
SP_PIR_KEYWORDS	dna-directed rna polymerase	RT	4	1.0E0	1.0E0
Annotation Cluster 71	Enrichment Score: 0.01		Count	P_Value	Benjamini
GOTERM_MF_FAT	DNA polymerase activity	RT	17	9.4E-1	1.0E0
GOTERM_MF_FAT	RNA-directed DNA polymerase activity	RT	11	9.7E-1	1.0E0
SP_PIR_KEYWORDS	rna-directed dna polymerase	RT	10	9.8E-1	1.0E0
GOTERM_MF_FAT	endonuclease activity	RT	15	9.9E-1	1.0E0
GOTERM_MF_FAT	nuclease activity	RT	23	9.9E-1	1.0E0
SP_PIR_KEYWORDS	Endonuclease	RT	12	9.9E-1	1.0E0
Annotation Cluster 72	Enrichment Score: 0.01		Count	P_Value	Benjamini
GOTERM_BP_FAT	cellular macromolecular complex assembly	RT	12	9.3E-1	1.0E0
GOTERM_BP_FAT	chromosome organization	RT	8	9.8E-1	1.0E0
GOTERM_BP_FAT	chromatin organization	RT	6	9.9E-1	1.0E0
GOTERM_CC_FAT	nucleosome	RT	4	9.9E-1	1.0E0
GOTERM_BP_FAT	chromatin assembly or disassembly	RT	5	9.9E-1	1.0E0
SP_PIR_KEYWORDS	chromosomal protein	RT	4	9.9E-1	1.0E0
INTERPRO	Histone core	RT	4	9.9E-1	1.0E0
GOTERM_BP_FAT	nucleosome assembly	RT	4	9.9E-1	1.0E0
GOTERM_BP_FAT	DNA packaging	RT	4	9.9E-1	1.0E0
GOTERM_BP_FAT	chromatin assembly	RT	4	9.9E-1	1.0E0
GOTERM_BP_FAT	protein-DNA complex assembly	RT	4	9.9E-1	1.0E0
GOTERM_BP_FAT	nucleosome organization	RT	4	9.9E-1	1.0E0
INTERPRO	Histone-fold	RT	5	9.9E-1	1.0E0
GOTERM_CC_FAT	chromatin	RT	4	9.9E-1	1.0E0
GOTERM_CC_FAT	chromosomal part	RT	6	9.9E-1	1.0E0
GOTERM_CC_FAT	chromosome	RT	10	1.0E0	1.0E0
GOTERM_CC_FAT	protein-DNA complex	RT	4	1.0E0	1.0E0
Annotation Cluster 73	Enrichment Score: 0		Count	P_Value	Benjamini
INTERPRO	Zinc finger, C2H2-type	RT	7	9.9E-1	1.0E0
SMART	ZnF_C2H2	RT	6	1.0E0	1.0E0
INTERPRO	Zinc finger, C2H2-like	RT	6	1.0E0	1.0E0
Annotation Cluster 74	Enrichment Score: 0		Count	P_Value	Benjamini
GOTERM_MF_FAT	phosphoprotein phosphatase activity	RT	11	1.0E0	1.0E0
GOTERM_BP_FAT	protein amino acid dephosphorylation	RT	4	1.0E0	1.0E0
GOTERM_BP_FAT	dephosphorylation	RT	4	1.0E0	1.0E0
Annotation Cluster 75	Enrichment Score: 0		Count	P_Value	Benjamini
GOTERM_BP_FAT	response to DNA damage stimulus	RT	7	1.0E0	1.0E0
GOTERM_BP_FAT	DNA repair	RT	7	1.0E0	1.0E0
GOTERM_BP_FAT	cellular response to stress	RT	7	1.0E0	1.0E0

236 terms were not clustered.

Please cite Nature Protocols 2009; 4(1):44 & Genome Biology 2003; 4(5):P3 within any publication that makes use of any methods inspired by **DAVID**.

Department of Health and Human Services

Term of Service | Contact Us | Site Map

DAVID Bioinformatics Resources 6.7
National Institute of Allergy and Infectious Diseases (NIAID), NIH

Functional Annotation Clustering

Help and Manual

Current Gene List: 3_Random9
Current Background: Giardia intestinalis
3000 DAVID IDs

Options **Classification Stringency** Medium

Rerun using options | Create Sublist

79 Cluster(s)

Download File

Category	Term		Count	P_Value	Benjamini
Annotation Cluster 1	Enrichment Score: 0.85		Count	P_Value	Benjamini
INTERPRO	EGF-like, type 3	RT	24	8.2E-2	1.0E0
INTERPRO	EGF-like, laminin	RT	19	1.8E-1	1.0E0
SMART	EGF_Lam	RT	19	1.9E-1	1.0E0
Annotation Cluster 2	Enrichment Score: 0.81		Count	P_Value	Benjamini
SP_PIR_KEYWORDS	nucleotidyltransferase	RT	20	7.8E-2	1.0E0
KEGG_PATHWAY	Purine metabolism	RT	25	1.0E-1	9.2E-1
KEGG_PATHWAY	RNA polymerase	RT	15	1.4E-1	9.1E-1
GOTERM_MF_FAT	RNA polymerase activity	RT	18	1.4E-1	1.0E0
GOTERM_BP_FAT	transcription	RT	19	1.5E-1	1.0E0
KEGG_PATHWAY	Pyrimidine metabolism	RT	25	1.6E-1	8.7E-1
GOTERM_MF_FAT	DNA-directed RNA polymerase activity	RT	17	1.8E-1	1.0E0
GOTERM_MF_FAT	nucleotidyltransferase activity	RT	46	2.6E-1	1.0E0
SP_PIR_KEYWORDS	dna-directed rna polymerase	RT	11	2.9E-1	1.0E0
Annotation Cluster 3	Enrichment Score: 0.78		Count	P_Value	Benjamini
INTERPRO	Giardia variant-specific surface protein	RT	151	5.1E-2	1.0E0
INTERPRO	EGF-like	RT	119	1.5E-1	1.0E0
SMART	EGF	RT	119	1.9E-1	1.0E0
INTERPRO	Furin-like repeat	RT	113	2.6E-1	1.0E0
SMART	FU	RT	113	3.1E-1	1.0E0
Annotation Cluster 4	Enrichment Score: 0.75		Count	P_Value	Benjamini
GOTERM_BP_FAT	cellular response to stress	RT	16	1.4E-1	1.0E0
GOTERM_BP_FAT	response to DNA damage stimulus	RT	15	1.8E-1	1.0E0
GOTERM_BP_FAT	DNA repair	RT	15	1.8E-1	1.0E0
GOTERM_BP_FAT	DNA metabolic process	RT	38	2.4E-1	1.0E0
Annotation Cluster 5	Enrichment Score: 0.69		Count	P_Value	Benjamini
INTERPRO	EGF-like, type 3	RT	24	8.2E-2	1.0E0
INTERPRO	EGF	RT	7	1.4E-1	1.0E0
INTERPRO	EGF, extracellular	RT	16	3.3E-1	1.0E0
SP_PIR_KEYWORDS	egf-like domain	RT	12	4.6E-1	9.9E-1
Annotation Cluster 6	Enrichment Score: 0.67		Count	P_Value	Benjamini
GOTERM_CC_FAT	ribosome	RT	49	2.5E-2	9.6E-1
GOTERM_CC_FAT	ribonucleoprotein complex	RT	60	8.9E-2	9.8E-1
GOTERM_CC_FAT	intracellular non-membrane-bounded organelle	RT	90	1.8E-1	1.0E0
GOTERM_CC_FAT	non-membrane-bounded organelle	RT	90	1.8E-1	1.0E0
GOTERM_MF_FAT	structural constituent of ribosome	RT	45	2.0E-1	1.0E0
GOTERM_MF_FAT	structural molecule activity	RT	52	3.0E-1	1.0E0
SP_PIR_KEYWORDS	ribosomal protein	RT	46	4.0E-1	1.0E0
KEGG_PATHWAY	Ribosome	RT	43	6.7E-1	9.8E-1
GOTERM_BP_FAT	translation	RT	63	7.1E-1	1.0E0
Annotation Cluster 7	Enrichment Score: 0.62		Count	P_Value	Benjamini
GOTERM_MF_FAT	nucleoside binding	RT	326	5.2E-2	1.0E0
GOTERM_MF_FAT	adenyl nucleotide binding	RT	322	7.5E-2	1.0E0
GOTERM_MF_FAT	purine nucleoside binding	RT	322	7.5E-2	1.0E0
GOTERM_MF_FAT	ATP binding	RT	317	9.0E-2	1.0E0
GOTERM_MF_FAT	adenyl ribonucleotide binding	RT	317	9.0E-2	1.0E0

GOTERM_MF_FAT	nucleotide binding	RT	369	1.1E-1	1.0E0
GOTERM_MF_FAT	purine nucleotide binding	RT	347	1.4E-1	1.0E0
GOTERM_MF_FAT	purine ribonucleotide binding	RT	342	1.6E-1	1.0E0
GOTERM_MF_FAT	ribonucleotide binding	RT	342	1.6E-1	1.0E0
GOTERM_BP_FAT	protein amino acid phosphorylation	RT	157	3.7E-1	1.0E0
GOTERM_BP_FAT	phosphorylation	RT	167	4.2E-1	1.0E0
SP_PIR_KEYWORDS	kinase	RT	200	4.8E-1	9.9E-1
GOTERM_BP_FAT	phosphate metabolic process	RT	175	4.8E-1	1.0E0
GOTERM_BP_FAT	phosphorus metabolic process	RT	175	4.8E-1	1.0E0
INTERPRO	Serine/threonine protein kinase-related	RT	137	4.9E-1	1.0E0
GOTERM_MF_FAT	protein kinase activity	RT	157	5.5E-1	1.0E0
INTERPRO	Protein kinase, core	RT	155	5.6E-1	1.0E0
SP_PIR_KEYWORDS	atp-binding	RT	155	5.6E-1	9.9E-1
GOTERM_MF_FAT	protein serine/threonine kinase activity	RT	61	5.8E-1	1.0E0
SP_PIR_KEYWORDS	nucleotide-binding	RT	162	6.1E-1	9.9E-1
Annotation Cluster 8	Enrichment Score: 0.56		Count	P_Value	Benjamini
INTERPRO	Tetratricopeptide region	RT	19	7.0E-2	1.0E0
INTERPRO	Tetratricopeptide repeat	RT	17	2.7E-1	1.0E0
SMART	TPR	RT	17	2.9E-1	1.0E0
INTERPRO	Tetratricopeptide-like helical	RT	21	3.0E-1	1.0E0
INTERPRO	Tetratricopeptide TPR-1	RT	14	3.6E-1	1.0E0
SP_PIR_KEYWORDS	tpr repeat	RT	6	7.4E-1	9.9E-1
Annotation Cluster 9	Enrichment Score: 0.49		Count	P_Value	Benjamini
INTERPRO	WD40 repeat, subgroup	RT	37	2.2E-1	1.0E0
INTERPRO	WD40 repeat 2	RT	29	2.3E-1	1.0E0
INTERPRO	WD40 repeat, region	RT	31	2.7E-1	1.0E0
INTERPRO	WD40 repeat	RT	47	2.9E-1	1.0E0
SMART	WD40	RT	47	3.3E-1	1.0E0
INTERPRO	WD40/YVTN repeat-like	RT	45	3.6E-1	1.0E0
SP_PIR_KEYWORDS	wd repeat	RT	32	4.0E-1	1.0E0
INTERPRO	WD40 repeat, conserved site	RT	17	7.1E-1	1.0E0
Annotation Cluster 10	Enrichment Score: 0.47		Count	P_Value	Benjamini
GOTERM_MF_FAT	peptidyl-prolyl cis-trans isomerase activity	RT	7	2.8E-1	1.0E0
GOTERM_MF_FAT	cis-trans isomerase activity	RT	7	2.8E-1	1.0E0
SP_PIR_KEYWORDS	Rotamase	RT	7	3.2E-1	1.0E0
INTERPRO	Peptidyl-prolyl cis-trans isomerase, FKBP-type	RT	5	5.1E-1	1.0E0
Annotation Cluster 11	Enrichment Score: 0.46		Count	P_Value	Benjamini
GOTERM_BP_FAT	homeostatic process	RT	15	1.8E-1	1.0E0
GOTERM_BP_FAT	cellular homeostasis	RT	14	2.3E-1	1.0E0
GOTERM_BP_FAT	cell redox homeostasis	RT	13	2.9E-1	1.0E0
INTERPRO	Thioredoxin fold	RT	15	3.9E-1	1.0E0
INTERPRO	Thioredoxin-like	RT	9	5.3E-1	1.0E0
INTERPRO	Thioredoxin, core	RT	5	6.8E-1	1.0E0
Annotation Cluster 12	Enrichment Score: 0.45		Count	P_Value	Benjamini
INTERPRO	Ankyrin	RT	262	2.2E-1	1.0E0
SMART	ANK	RT	262	2.9E-1	1.0E0
SP_PIR_KEYWORDS	ank repeat	RT	182	6.9E-1	9.9E-1
Annotation Cluster 13	Enrichment Score: 0.4		Count	P_Value	Benjamini
KEGG_PATHWAY	Nucleotide excision repair	RT	13	2.3E-1	9.2E-1
KEGG_PATHWAY	DNA replication	RT	15	4.7E-1	9.9E-1
KEGG_PATHWAY	Mismatch repair	RT	10	5.9E-1	9.8E-1
Annotation Cluster 14	Enrichment Score: 0.36		Count	P_Value	Benjamini
GOTERM_CC_FAT	cytosolic part	RT	7	3.2E-1	1.0E0
GOTERM_CC_FAT	prefoldin complex	RT	5	4.1E-1	1.0E0
INTERPRO	Prefoldin alpha-like	RT	3	6.2E-1	1.0E0
Annotation Cluster 15	Enrichment Score: 0.35		Count	P_Value	Benjamini
GOTERM_CC_FAT	cytosol	RT	21	5.8E-2	9.8E-1
GOTERM_CC_FAT	proteasome complex	RT	14	2.8E-1	1.0E0

GOTERM_CC_FAT	proteasome core complex	RT			10	2.9E-1	1.0E0
INTERPRO	Proteasome, alpha-subunit, conserved site	RT			6	3.8E-1	1.0E0
GOTERM_BP_FAT	ubiquitin-dependent protein catabolic process	RT			10	4.0E-1	1.0E0
GOTERM_MF_FAT	threonine-type peptidase activity	RT			10	4.3E-1	1.0E0
GOTERM_MF_FAT	threonine-type endopeptidase activity	RT			10	4.3E-1	1.0E0
SP_PIR_KEYWORDS	proteasome	RT			14	4.3E-1	1.0E0
INTERPRO	Proteasome, subunit alpha/beta	RT			10	4.3E-1	1.0E0
GOTERM_BP_FAT	cellular macromolecule catabolic process	RT			21	5.0E-1	1.0E0
PIR_SUPERFAMILY	PIRSF001212:multicatalytic endopeptidase complex chain C9	RT			5	5.1E-1	1.0E0
INTERPRO	Proteasome, alpha and beta subunits	RT			9	5.3E-1	1.0E0
KEGG_PATHWAY	Proteasome	RT			19	5.4E-1	9.7E-1
GOTERM_BP_FAT	proteolysis involved in cellular protein catabolic process	RT			20	5.6E-1	1.0E0
GOTERM_BP_FAT	cellular protein catabolic process	RT			20	5.6E-1	1.0E0
GOTERM_BP_FAT	modification-dependent protein catabolic process	RT			16	5.7E-1	1.0E0
GOTERM_BP_FAT	modification-dependent macromolecule catabolic process	RT			16	5.7E-1	1.0E0
GOTERM_BP_FAT	macromolecule catabolic process	RT			22	7.3E-1	1.0E0
GOTERM_BP_FAT	protein catabolic process	RT			21	7.8E-1	1.0E0
SP_PIR_KEYWORDS	threonine protease	RT			4	8.3E-1	9.9E-1
Annotation Cluster 16	Enrichment Score: 0.34	G			Count	P_Value	Benjamini
GOTERM_MF_FAT	endopeptidase activity	RT			30	3.0E-1	1.0E0
GOTERM_MF_FAT	cysteine-type peptidase activity	RT			20	3.5E-1	1.0E0
INTERPRO	Peptidase, cysteine peptidase active site	RT			20	3.6E-1	1.0E0
INTERPRO	Peptidase C1A, papain	RT			18	3.9E-1	1.0E0
INTERPRO	Peptidase C1A, papain C-terminal	RT			18	3.9E-1	1.0E0
SMART	Pept_C1	RT			18	4.1E-1	1.0E0
GOTERM_MF_FAT	cysteine-type endopeptidase activity	RT			16	4.1E-1	1.0E0
GOTERM_BP_FAT	proteolysis	RT			51	5.9E-1	1.0E0
GOTERM_MF_FAT	peptidase activity, acting on L-amino acid peptides	RT			40	6.0E-1	1.0E0
GOTERM_MF_FAT	peptidase activity	RT			46	6.3E-1	1.0E0
SP_PIR_KEYWORDS	thiol protease	RT			7	6.4E-1	9.9E-1
SP_PIR_KEYWORDS	Protease	RT			22	6.7E-1	9.8E-1
Annotation Cluster 17	Enrichment Score: 0.31	G			Count	P_Value	Benjamini
COG_ONTOLOGY	Lipid metabolism	RT			11	1.5E-1	1.0E0
GOTERM_MF_FAT	ligase activity, forming carbon-sulfur bonds	RT			4	6.5E-1	1.0E0
GOTERM_MF_FAT	fatty-acid ligase activity	RT			4	6.5E-1	1.0E0
GOTERM_MF_FAT	long-chain-fatty-acid-CoA ligase activity	RT			4	6.5E-1	1.0E0
INTERPRO	AMP-dependent synthetase and ligase	RT			5	6.8E-1	1.0E0
Annotation Cluster 18	Enrichment Score: 0.31	G			Count	P_Value	Benjamini
INTERPRO	Amino acid transporter, transmembrane	RT			8	3.4E-1	1.0E0
GOTERM_CC_FAT	intrinsic to membrane	RT			37	4.7E-1	1.0E0
GOTERM_CC_FAT	integral to membrane	RT			37	4.7E-1	1.0E0
SP_PIR_KEYWORDS	transmembrane	RT			19	6.0E-1	9.9E-1
SP_PIR_KEYWORDS	membrane	RT			17	6.3E-1	9.9E-1
Annotation Cluster 19	Enrichment Score: 0.3	G			Count	P_Value	Benjamini
SP_PIR_KEYWORDS	cytoplasm	RT			14	2.2E-1	1.0E0
GOTERM_MF_FAT	unfolded protein binding	RT			15	2.9E-1	1.0E0
PIR_SUPERFAMILY	PIRSF002584:molecular chaperone t-complex-type	RT			6	5.5E-1	1.0E0
INTERPRO	Chaperone, tailless complex polypeptide 1	RT			6	5.6E-1	1.0E0
INTERPRO	Chaperonin TCP-1, conserved site	RT			6	7.0E-1	1.0E0
INTERPRO	Chaperonin Cpn60/TCP-1	RT			6	7.0E-1	1.0E0
SP_PIR_KEYWORDS	Chaperone	RT			7	8.5E-1	9.9E-1
Annotation Cluster 20	Enrichment Score: 0.3	G			Count	P_Value	Benjamini
COG_ONTOLOGY	Nucleotide transport and metabolism	RT			7	2.9E-1	9.8E-1
GOTERM_BP_FAT	nucleotide catabolic process	RT			4	4.3E-1	1.0E0
GOTERM_BP_FAT	nucleobase, nucleoside and nucleotide catabolic process	RT			5	4.9E-1	1.0E0
GOTERM_BP_FAT	nitrogen compound catabolic process	RT			5	4.9E-1	1.0E0

GOTERM_BP_FAT	nucleobase, nucleoside, nucleotide and nucleic acid catabolic process	RT			5	4.9E-1	1.0E0
PIR_SUPERFAMILY	PIRSF017316:Pesterase_C1039	RT			3	6.2E-1	1.0E0
PIR_SUPERFAMILY	PIRSF017316:phosphoesterase, C1039.02 type	RT			3	6.2E-1	1.0E0
INTERPRO	5'-Nucleotidase and apyrase	RT			3	6.2E-1	1.0E0
INTERPRO	Predicted phosphoesterase, C1039.02 type	RT			3	6.2E-1	1.0E0
Annotation Cluster 21	Enrichment Score: 0.29				Count	P_Value	Benjamini
SP_PIR_KEYWORDS	zinc-finger	RT			41	2.4E-1	1.0E0
INTERPRO	Zinc finger, RING-type	RT			43	2.7E-1	1.0E0
SMART	RING	RT			43	3.1E-1	1.0E0
INTERPRO	Zinc finger, C3HC4 RING-type	RT			23	3.6E-1	1.0E0
SP_PIR_KEYWORDS	zinc	RT			51	6.1E-1	9.9E-1
GOTERM_MF_FAT	cation binding	RT			148	6.6E-1	1.0E0
GOTERM_MF_FAT	ion binding	RT			148	6.6E-1	1.0E0
GOTERM_MF_FAT	metal ion binding	RT			146	6.7E-1	1.0E0
SP_PIR_KEYWORDS	metal-binding	RT			59	7.3E-1	9.9E-1
GOTERM_MF_FAT	zinc ion binding	RT			85	7.7E-1	1.0E0
GOTERM_MF_FAT	transition metal ion binding	RT			105	7.9E-1	1.0E0
Annotation Cluster 22	Enrichment Score: 0.29				Count	P_Value	Benjamini
INTERPRO	DEAD-like helicase, N-terminal	RT			28	3.3E-1	1.0E0
GOTERM_MF_FAT	ATPase activity	RT			58	3.4E-1	1.0E0
SMART	DEXDc	RT			28	3.6E-1	1.0E0
GOTERM_MF_FAT	helicase activity	RT			34	3.8E-1	1.0E0
INTERPRO	Helicase, superfamily 1 and 2, ATP-binding	RT			27	4.5E-1	1.0E0
GOTERM_MF_FAT	ATPase activity, coupled	RT			42	5.2E-1	1.0E0
INTERPRO	DNA/RNA helicase, C-terminal	RT			27	5.2E-1	1.0E0
SMART	HELICc	RT			27	5.6E-1	1.0E0
GOTERM_MF_FAT	purine NTP-dependent helicase activity	RT			23	5.8E-1	1.0E0
GOTERM_MF_FAT	ATP-dependent helicase activity	RT			23	5.8E-1	1.0E0
INTERPRO	DNA/RNA helicase, DEAD/DEAH box type, N-terminal	RT			18	6.5E-1	1.0E0
SP_PIR_KEYWORDS	helicase	RT			26	7.4E-1	9.9E-1
INTERPRO	RNA helicase, DEAD-box type, Q motif	RT			8	9.5E-1	1.0E0
Annotation Cluster 23	Enrichment Score: 0.27				Count	P_Value	Benjamini
INTERPRO	Metallophosphoesterase	RT			16	1.6E-1	1.0E0
GOTERM_MF_FAT	phosphatase activity	RT			28	3.9E-1	1.0E0
INTERPRO	Serine/threonine-specific protein phosphatase and bis(5-nucleosyl)-tetraphosphatase	RT			10	5.6E-1	1.0E0
SMART	PP2Ac	RT			10	5.8E-1	1.0E0
GOTERM_MF_FAT	iron ion binding	RT			19	5.8E-1	1.0E0
SP_PIR_KEYWORDS	iron	RT			12	7.7E-1	9.8E-1
GOTERM_MF_FAT	phosphoprotein phosphatase activity	RT			15	8.6E-1	1.0E0
PIR_SUPERFAMILY	PIRSF000908:serine/threonine-protein phosphatase, conventional type	RT			4	9.4E-1	1.0E0
Annotation Cluster 24	Enrichment Score: 0.27				Count	P_Value	Benjamini
INTERPRO	Aminotransferase, class I and II	RT			5	3.1E-1	1.0E0
GOTERM_MF_FAT	vitamin B6 binding	RT			8	3.4E-1	1.0E0
GOTERM_MF_FAT	pyridoxal phosphate binding	RT			8	3.4E-1	1.0E0
GOTERM_MF_FAT	vitamin binding	RT			8	4.8E-1	1.0E0
INTERPRO	Pyridoxal phosphate-dependent transferase, major region, subdomain 1	RT			6	5.6E-1	1.0E0
INTERPRO	Pyridoxal phosphate-dependent transferase, major region, subdomain 2	RT			3	6.2E-1	1.0E0
GOTERM_MF_FAT	transferase activity, transferring nitrogenous groups	RT			5	6.7E-1	1.0E0
KEGG_PATHWAY	Alanine, aspartate and glutamate metabolism	RT			3	6.9E-1	9.8E-1
GOTERM_MF_FAT	transaminase activity	RT			3	9.0E-1	1.0E0
SP_PIR_KEYWORDS	Aminotransferase	RT			3	9.2E-1	1.0E0
Annotation Cluster 25	Enrichment Score: 0.26				Count	P_Value	Benjamini
GOTERM_BP_FAT	monosaccharide metabolic process	RT			16	2.9E-1	1.0E0
GOTERM_BP_FAT	generation of precursor metabolites and energy	RT			17	4.1E-1	1.0E0
GOTERM_BP_FAT	glycolysis	RT			8	4.6E-1	1.0E0

GOTERM_BP_FAT	glucose metabolic process	RT	13	5.0E-1	1.0E0
GOTERM_BP_FAT	hexose metabolic process	RT	13	5.0E-1	1.0E0
SP_PIR_KEYWORDS	glycolysis	RT	5	5.5E-1	9.9E-1
GOTERM_BP_FAT	cellular carbohydrate catabolic process	RT	9	6.2E-1	1.0E0
GOTERM_BP_FAT	carbohydrate catabolic process	RT	9	6.2E-1	1.0E0
GOTERM_BP_FAT	glucose catabolic process	RT	9	6.2E-1	1.0E0
GOTERM_BP_FAT	hexose catabolic process	RT	9	6.2E-1	1.0E0
GOTERM_BP_FAT	monosaccharide catabolic process	RT	9	6.2E-1	1.0E0
GOTERM_BP_FAT	alcohol catabolic process	RT	9	6.2E-1	1.0E0
KEGG_PATHWAY	Glycolysis / Gluconeogenesis	RT	3	9.9E-1	1.0E0
Annotation Cluster 26	Enrichment Score: 0.26		Count	P_Value	Benjamini
SP_PIR_KEYWORDS	Transcription	RT	7	3.2E-1	1.0E0
GOTERM_MF_FAT	ribonucleoside binding	RT	4	4.4E-1	1.0E0
INTERPRO	RNA polymerase Rpb2, domain 3	RT	3	6.2E-1	1.0E0
INTERPRO	RNA polymerase, beta subunit, protrusion	RT	3	6.2E-1	1.0E0
INTERPRO	RNA polymerase, beta subunit, conserved site	RT	3	6.2E-1	1.0E0
INTERPRO	DNA-directed RNA polymerase, subunit 2, domain 6	RT	3	6.2E-1	1.0E0
INTERPRO	RNA polymerase Rpb2, domain 7	RT	3	6.2E-1	1.0E0
INTERPRO	DNA-directed RNA polymerase, subunit 2	RT	3	6.2E-1	1.0E0
Annotation Cluster 27	Enrichment Score: 0.24		Count	P_Value	Benjamini
INTERPRO	Kinesin, motor region, conserved site	RT	14	1.7E-1	1.0E0
GOTERM_CC_FAT	microtubule	RT	19	5.2E-1	1.0E0
GOTERM_CC_FAT	microtubule cytoskeleton	RT	29	5.3E-1	1.0E0
GOTERM_CC_FAT	cytoskeletal part	RT	29	5.3E-1	1.0E0
GOTERM_MF_FAT	microtubule motor activity	RT	25	6.2E-1	1.0E0
GOTERM_MF_FAT	motor activity	RT	25	6.2E-1	1.0E0
INTERPRO	Kinesin, motor region	RT	17	6.3E-1	1.0E0
SMART	KISc	RT	17	6.5E-1	1.0E0
GOTERM_CC_FAT	cytoskeleton	RT	29	6.6E-1	1.0E0
SP_PIR_KEYWORDS	motor protein	RT	15	6.7E-1	9.9E-1
GOTERM_BP_FAT	microtubule-based movement	RT	27	6.7E-1	1.0E0
SP_PIR_KEYWORDS	microtubule	RT	19	7.5E-1	9.8E-1
GOTERM_BP_FAT	microtubule-based process	RT	30	7.6E-1	1.0E0
Annotation Cluster 28	Enrichment Score: 0.24		Count	P_Value	Benjamini
INTERPRO	DNA-directed DNA polymerase, family B, conserved region2	RT	4	4.5E-1	1.0E0
INTERPRO	DNA-directed DNA polymerase, family B, conserved site	RT	4	4.5E-1	1.0E0
GOTERM_MF_FAT	DNA-directed DNA polymerase activity	RT	7	5.9E-1	1.0E0
SP_PIR_KEYWORDS	dna replication	RT	7	6.4E-1	9.9E-1
INTERPRO	DNA-directed DNA polymerase, family B	RT	4	6.5E-1	1.0E0
SMART	POLBc	RT	4	6.6E-1	1.0E0
SP_PIR_KEYWORDS	dna-directed dna polymerase	RT	4	6.9E-1	9.8E-1
Annotation Cluster 29	Enrichment Score: 0.22		Count	P_Value	Benjamini
SP_PIR_KEYWORDS	cyclin	RT	9	1.7E-1	1.0E0
INTERPRO	Cyclin, N-terminal	RT	5	5.1E-1	1.0E0
INTERPRO	Cyclin-related	RT	4	8.0E-1	1.0E0
INTERPRO	Cyclin, C-terminal	RT	3	8.0E-1	1.0E0
INTERPRO	Cyclin	RT	3	9.0E-1	1.0E0
SMART	CYCLIN	RT	3	9.1E-1	1.0E0
Annotation Cluster 30	Enrichment Score: 0.22		Count	P_Value	Benjamini
GOTERM_CC_FAT	organelle membrane	RT	8	3.5E-1	1.0E0
GOTERM_CC_FAT	endoplasmic reticulum part	RT	3	5.5E-1	1.0E0
GOTERM_CC_FAT	nuclear envelope-endoplasmic reticulum network	RT	3	5.5E-1	1.0E0
GOTERM_CC_FAT	endoplasmic reticulum membrane	RT	3	5.5E-1	1.0E0
GOTERM_CC_FAT	endomembrane system	RT	8	8.5E-1	1.0E0
GOTERM_CC_FAT	endoplasmic reticulum	RT	3	9.2E-1	1.0E0
Annotation Cluster 31	Enrichment Score: 0.22		Count	P_Value	Benjamini
GOTERM_MF_FAT	magnesium ion binding	RT	11	3.5E-1	1.0E0

GOTERM_BP_FAT	phospholipid transport	RT	4	4.3E-1	1.0E0
INTERPRO	ATPase, P-type, phospholipid-translocating, flippase	RT	4	4.5E-1	1.0E0
INTERPRO	ATPase, P-type phosphorylation site	RT	6	5.6E-1	1.0E0
GOTERM_BP_FAT	lipid transport	RT	4	6.4E-1	1.0E0
GOTERM_BP_FAT	lipid localization	RT	4	6.4E-1	1.0E0
GOTERM_MF_FAT	phospholipid transporter activity	RT	4	6.5E-1	1.0E0
GOTERM_MF_FAT	phospholipid-translocating ATPase activity	RT	4	6.5E-1	1.0E0
GOTERM_MF_FAT	aminophospholipid transporter activity	RT	4	6.5E-1	1.0E0
INTERPRO	ATPase, P-type, ATPase-associated region	RT	7	7.2E-1	1.0E0
GOTERM_MF_FAT	lipid transporter activity	RT	4	7.9E-1	1.0E0
GOTERM_MF_FAT	ATPase activity, coupled to transmembrane movement of ions, phosphorylative mechanism	RT	7	8.1E-1	1.0E0
INTERPRO	ATPase, P-type, K/Mg/Cd/Cu/Zn/Na/Ca/Na/H-transporter	RT	7	8.1E-1	1.0E0
Annotation Cluster 32	Enrichment Score: 0.21		Count	P_Value	Benjamini
INTERPRO	Haloacid dehalogenase-like hydrolase	RT	7	5.9E-1	1.0E0
GOTERM_MF_FAT	phosphoglycolate phosphatase activity	RT	3	6.2E-1	1.0E0
INTERPRO	HAD-superfamily hydrolase, subfamily IA, variant 1	RT	3	6.2E-1	1.0E0
Annotation Cluster 33	Enrichment Score: 0.2		Count	P_Value	Benjamini
GOTERM_BP_FAT	lipid biosynthetic process	RT	10	4.0E-1	1.0E0
GOTERM_BP_FAT	isoprenoid biosynthetic process	RT	4	6.4E-1	1.0E0
GOTERM_BP_FAT	isoprenoid metabolic process	RT	4	6.4E-1	1.0E0
KEGG_PATHWAY	Terpenoid backbone biosynthesis	RT	3	9.7E-1	1.0E0
Annotation Cluster 34	Enrichment Score: 0.2		Count	P_Value	Benjamini
GOTERM_MF_FAT	nucleobase, nucleoside, nucleotide kinase activity	RT	7	5.9E-1	1.0E0
GOTERM_MF_FAT	nucleotide kinase activity	RT	4	6.5E-1	1.0E0
GOTERM_MF_FAT	phosphotransferase activity, phosphate group as acceptor	RT	5	6.7E-1	1.0E0
Annotation Cluster 35	Enrichment Score: 0.2		Count	P_Value	Benjamini
GOTERM_BP_FAT	phospholipid biosynthetic process	RT	6	3.6E-1	1.0E0
GOTERM_BP_FAT	lipid biosynthetic process	RT	10	4.0E-1	1.0E0
GOTERM_BP_FAT	organophosphate metabolic process	RT	9	5.0E-1	1.0E0
GOTERM_BP_FAT	phospholipid metabolic process	RT	8	5.9E-1	1.0E0
GOTERM_BP_FAT	phosphoinositide metabolic process	RT	4	8.8E-1	1.0E0
GOTERM_BP_FAT	glycerolipid metabolic process	RT	4	8.8E-1	1.0E0
GOTERM_BP_FAT	glycerophospholipid metabolic process	RT	4	8.8E-1	1.0E0
KEGG_PATHWAY	Inositol phosphate metabolism	RT	3	9.4E-1	1.0E0
Annotation Cluster 36	Enrichment Score: 0.18		Count	P_Value	Benjamini
INTERPRO	Thioredoxin-like	RT	9	5.3E-1	1.0E0
GOTERM_MF_FAT	antioxidant activity	RT	5	6.7E-1	1.0E0
INTERPRO	Alkyl hydroperoxide reductase/ Thiol specific antioxidant/ Mal allergen	RT	4	8.0E-1	1.0E0
Annotation Cluster 37	Enrichment Score: 0.17		Count	P_Value	Benjamini
INTERPRO	ATPase associated with various cellular activities, AAA-5	RT	5	5.1E-1	1.0E0
INTERPRO	Dynein heavy chain, N-terminal region 2	RT	7	7.2E-1	1.0E0
SP_PIR_KEYWORDS	coiled coil	RT	3	8.3E-1	9.9E-1
Annotation Cluster 38	Enrichment Score: 0.15		Count	P_Value	Benjamini
GOTERM_MF_FAT	DNA-dependent ATPase activity	RT	6	5.5E-1	1.0E0
GOTERM_MF_FAT	DNA helicase activity	RT	4	7.9E-1	1.0E0
GOTERM_MF_FAT	ATP-dependent DNA helicase activity	RT	3	8.0E-1	1.0E0
Annotation Cluster 39	Enrichment Score: 0.15		Count	P_Value	Benjamini
GOTERM_CC_FAT	microtubule associated complex	RT	9	6.1E-1	1.0E0
GOTERM_CC_FAT	dynein complex	RT	7	7.0E-1	1.0E0
INTERPRO	Dynein heavy chain, N-terminal region 2	RT	7	7.2E-1	1.0E0
INTERPRO	Dynein heavy chain	RT	7	8.1E-1	1.0E0
Annotation Cluster 40	Enrichment Score: 0.15		Count	P_Value	Benjamini
GOTERM_BP_FAT	cell cycle	RT	6	5.3E-1	1.0E0
GOTERM_BP_FAT	cell cycle phase	RT	4	7.8E-1	1.0E0
GOTERM_BP_FAT	cell cycle process	RT	4	7.8E-1	1.0E0
GOTERM_BP_FAT	M phase	RT	4	7.8E-1	1.0E0
Annotation Cluster 41	Enrichment Score: 0.15		Count	P_Value	Benjamini

Category	Term		Count	P_Value	Benjamini
GOTERM_MF_FAT	electron carrier activity	RT	16	3.2E-1	1.0E0
INTERPRO	4Fe-4S ferredoxin, iron-sulpur binding domain	RT	6	7.0E-1	1.0E0
INTERPRO	4Fe-4S ferredoxin, iron-sulphur binding, subgroup	RT	6	7.0E-1	1.0E0
GOTERM_MF_FAT	4 iron, 4 sulfur cluster binding	RT	5	8.0E-1	1.0E0
INTERPRO	4Fe-4S ferredoxin, iron-sulphur binding, conserved site	RT	4	8.9E-1	1.0E0
GOTERM_MF_FAT	iron-sulfur cluster binding	RT	10	9.2E-1	1.0E0
GOTERM_MF_FAT	metal cluster binding	RT	10	9.2E-1	1.0E0
Annotation Cluster 42	Enrichment Score: 0.15		Count	P_Value	Benjamini
GOTERM_MF_FAT	protein kinase regulator activity	RT	5	6.7E-1	1.0E0
GOTERM_MF_FAT	kinase regulator activity	RT	5	6.7E-1	1.0E0
GOTERM_MF_FAT	cAMP-dependent protein kinase regulator activity	RT	3	8.0E-1	1.0E0
Annotation Cluster 43	Enrichment Score: 0.14		Count	P_Value	Benjamini
GOTERM_BP_FAT	carbohydrate biosynthetic process	RT	5	6.6E-1	1.0E0
GOTERM_BP_FAT	cellular carbohydrate biosynthetic process	RT	5	6.6E-1	1.0E0
GOTERM_BP_FAT	alcohol biosynthetic process	RT	3	8.9E-1	1.0E0
Annotation Cluster 44	Enrichment Score: 0.13		Count	P_Value	Benjamini
PIR_SUPERFAMILY	PIRSF002165:ribosomal protein, L7Ab/L7Aa/L7Ae/L8e types/H/ACA ribonucleoprotein complex, NHP2 subunit	RT	3	6.2E-1	1.0E0
INTERPRO	High mobility group-like nuclear protein	RT	3	6.2E-1	1.0E0
INTERPRO	Ribosomal protein L7A/RS6 family	RT	3	8.0E-1	1.0E0
INTERPRO	Ribosomal protein L7Ae/L30e/S12e/Gadd45	RT	3	9.5E-1	1.0E0
Annotation Cluster 45	Enrichment Score: 0.13		Count	P_Value	Benjamini
INTERPRO	EF hand	RT	8	4.9E-1	1.0E0
INTERPRO	EF-HAND 1	RT	13	5.4E-1	1.0E0
INTERPRO	EF-Hand type	RT	11	6.9E-1	1.0E0
GOTERM_MF_FAT	calcium ion binding	RT	24	7.8E-1	1.0E0
INTERPRO	Calcium-binding EF-hand	RT	6	8.8E-1	1.0E0
INTERPRO	EF-HAND 2	RT	9	8.8E-1	1.0E0
SMART	EFh	RT	6	8.9E-1	1.0E0
SP_PIR_KEYWORDS	calcium	RT	8	9.1E-1	1.0E0
Annotation Cluster 46	Enrichment Score: 0.13		Count	P_Value	Benjamini
INTERPRO	Thioredoxin-like	RT	9	5.3E-1	1.0E0
INTERPRO	Thioredoxin, conserved site	RT	4	8.0E-1	1.0E0
INTERPRO	Thioredoxin domain	RT	4	8.0E-1	1.0E0
INTERPRO	Thioredoxin-like subdomain	RT	3	9.0E-1	1.0E0
Annotation Cluster 47	Enrichment Score: 0.13		Count	P_Value	Benjamini
GOTERM_MF_FAT	protein serine/threonine kinase activity	RT	61	5.8E-1	1.0E0
INTERPRO	Serine/threonine protein kinase, active site	RT	52	6.2E-1	1.0E0
INTERPRO	Protein kinase, ATP binding site	RT	46	7.8E-1	1.0E0
INTERPRO	Serine/threonine protein kinase	RT	32	8.2E-1	1.0E0
SMART	S_TKc	RT	32	8.4E-1	1.0E0
SP_PIR_KEYWORDS	serine/threonine-protein kinase	RT	34	8.8E-1	9.9E-1
Annotation Cluster 48	Enrichment Score: 0.13		Count	P_Value	Benjamini
INTERPRO	Pumilio RNA-binding region	RT	4	6.5E-1	1.0E0
SMART	Pumilio	RT	4	6.6E-1	1.0E0
INTERPRO	Armadillo-like helical	RT	10	9.5E-1	1.0E0
Annotation Cluster 49	Enrichment Score: 0.13		Count	P_Value	Benjamini
INTERPRO	von Willebrand factor, type A	RT	5	6.8E-1	1.0E0
SMART	VWA	RT	5	6.9E-1	1.0E0
INTERPRO	Copine	RT	3	9.0E-1	1.0E0
Annotation Cluster 50	Enrichment Score: 0.1		Count	P_Value	Benjamini
GOTERM_BP_FAT	cellular amino acid biosynthetic process	RT	4	6.4E-1	1.0E0
GOTERM_BP_FAT	amine biosynthetic process	RT	4	7.8E-1	1.0E0
GOTERM_BP_FAT	organic acid biosynthetic process	RT	4	8.8E-1	1.0E0
GOTERM_BP_FAT	carboxylic acid biosynthetic process	RT	4	8.8E-1	1.0E0
Annotation Cluster 51	Enrichment Score: 0.1		Count	P_Value	Benjamini
GOTERM_BP_FAT	generation of precursor metabolites and energy	RT	17	4.1E-1	1.0E0
GOTERM_MF_FAT	ATPase activity, coupled to transmembrane movement of ions	RT	13	5.3E-1	1.0E0

INTERPRO	ATPase, P-type phosphorylation site	RT	6	5.6E-1	1.0E0
GOTERM_MF_FAT	proton-transporting ATPase activity, rotational mechanism	RT	5	6.7E-1	1.0E0
GOTERM_BP_FAT	ion transport	RT	14	7.0E-1	1.0E0
GOTERM_BP_FAT	cation transport	RT	14	7.0E-1	1.0E0
GOTERM_CC_FAT	proton-transporting two-sector ATPase complex, catalytic domain	RT	5	7.1E-1	1.0E0
INTERPRO	ATPase, P-type, ATPase-associated region	RT	7	7.2E-1	1.0E0
GOTERM_MF_FAT	monovalent inorganic cation transmembrane transporter activity	RT	9	7.4E-1	1.0E0
GOTERM_MF_FAT	hydrogen ion transmembrane transporter activity	RT	9	7.4E-1	1.0E0
GOTERM_MF_FAT	ATPase activity, coupled to transmembrane movement of substances	RT	16	7.5E-1	1.0E0
GOTERM_MF_FAT	ATPase activity, coupled to movement of substances	RT	16	7.5E-1	1.0E0
GOTERM_MF_FAT	primary active transmembrane transporter activity	RT	17	7.7E-1	1.0E0
GOTERM_MF_FAT	P-P-bond-hydrolysis-driven transmembrane transporter activity	RT	17	7.7E-1	1.0E0
GOTERM_BP_FAT	ATP metabolic process	RT	14	7.8E-1	1.0E0
GOTERM_BP_FAT	ATP biosynthetic process	RT	14	7.8E-1	1.0E0
GOTERM_BP_FAT	nucleoside triphosphate biosynthetic process	RT	15	7.8E-1	1.0E0
GOTERM_BP_FAT	nucleoside triphosphate metabolic process	RT	15	7.8E-1	1.0E0
GOTERM_BP_FAT	purine nucleoside triphosphate biosynthetic process	RT	15	7.8E-1	1.0E0
GOTERM_BP_FAT	purine nucleoside triphosphate metabolic process	RT	15	7.8E-1	1.0E0
GOTERM_BP_FAT	purine ribonucleoside triphosphate metabolic process	RT	15	7.8E-1	1.0E0
GOTERM_BP_FAT	purine ribonucleoside triphosphate biosynthetic process	RT	15	7.8E-1	1.0E0
GOTERM_BP_FAT	ribonucleoside triphosphate metabolic process	RT	15	7.8E-1	1.0E0
GOTERM_BP_FAT	ribonucleoside triphosphate biosynthetic process	RT	15	7.8E-1	1.0E0
GOTERM_MF_FAT	cation-transporting ATPase activity	RT	6	8.0E-1	1.0E0
GOTERM_MF_FAT	ATPase activity, coupled to transmembrane movement of ions, phosphorylative mechanism	RT	7	8.1E-1	1.0E0
INTERPRO	ATPase, P-type, K/Mg/Cd/Cu/Zn/Na/Ca/Na/H-transporter	RT	7	8.1E-1	1.0E0
GOTERM_MF_FAT	inorganic cation transmembrane transporter activity	RT	10	8.3E-1	1.0E0
GOTERM_BP_FAT	purine ribonucleotide biosynthetic process	RT	15	8.4E-1	1.0E0
GOTERM_BP_FAT	purine ribonucleotide metabolic process	RT	15	8.4E-1	1.0E0
GOTERM_BP_FAT	ribonucleotide metabolic process	RT	15	8.4E-1	1.0E0
GOTERM_BP_FAT	ribonucleotide biosynthetic process	RT	15	8.4E-1	1.0E0
GOTERM_BP_FAT	purine nucleotide metabolic process	RT	16	8.4E-1	1.0E0
GOTERM_BP_FAT	nitrogen compound biosynthetic process	RT	26	8.6E-1	1.0E0
GOTERM_MF_FAT	hydrolase activity, acting on acid anhydrides, catalyzing transmembrane movement of substances	RT	16	8.6E-1	1.0E0
GOTERM_BP_FAT	oxidative phosphorylation	RT	7	8.6E-1	1.0E0
GOTERM_BP_FAT	ATP synthesis coupled proton transport	RT	7	8.6E-1	1.0E0
GOTERM_BP_FAT	energy coupled proton transport, down electrochemical gradient	RT	7	8.6E-1	1.0E0
GOTERM_BP_FAT	purine nucleotide biosynthetic process	RT	15	8.8E-1	1.0E0
GOTERM_BP_FAT	nucleotide biosynthetic process	RT	20	9.0E-1	1.0E0
GOTERM_CC_FAT	proton-transporting two-sector ATPase complex	RT	7	9.1E-1	1.0E0
GOTERM_BP_FAT	monovalent inorganic cation transport	RT	8	9.1E-1	1.0E0
GOTERM_BP_FAT	ion transmembrane transport	RT	7	9.1E-1	1.0E0
SP_PIR_KEYWORDS	ion transport	RT	3	9.2E-1	1.0E0
GOTERM_BP_FAT	nucleobase, nucleoside and nucleotide biosynthetic process	RT	21	9.3E-1	1.0E0
GOTERM_BP_FAT	nucleobase, nucleoside, nucleotide and nucleic acid biosynthetic process	RT	21	9.3E-1	1.0E0
GOTERM_BP_FAT	transmembrane transport	RT	10	9.4E-1	1.0E0
GOTERM_BP_FAT	hydrogen transport	RT	7	9.5E-1	1.0E0
GOTERM_BP_FAT	proton transport	RT	7	9.5E-1	1.0E0
KEGG_PATHWAY	Oxidative phosphorylation	RT	6	9.8E-1	1.0E0
GOTERM_CC_FAT	proton-transporting V-type ATPase complex	RT	3	9.9E-1	1.0E0
Annotation Cluster 52	Enrichment Score: 0.1		Count	P_Value	Benjamini
GOTERM_CC_FAT	organelle membrane	RT	8	3.5E-1	1.0E0

GOTERM_CC_FAT	transport vesicle membrane	RT	4	5.6E-1	1.0E0
GOTERM_CC_FAT	transport vesicle	RT	4	5.6E-1	1.0E0
GOTERM_CC_FAT	cytoplasmic vesicle part	RT	4	7.2E-1	1.0E0
GOTERM_CC_FAT	vesicle membrane	RT	4	7.2E-1	1.0E0
GOTERM_CC_FAT	coated vesicle membrane	RT	4	7.2E-1	1.0E0
GOTERM_CC_FAT	cytoplasmic vesicle membrane	RT	4	7.2E-1	1.0E0
GOTERM_CC_FAT	coated vesicle	RT	4	7.2E-1	1.0E0
GOTERM_CC_FAT	vesicle coat	RT	4	7.2E-1	1.0E0
GOTERM_CC_FAT	cytoplasmic membrane-bounded vesicle	RT	4	7.2E-1	1.0E0
GOTERM_CC_FAT	vesicle	RT	4	7.2E-1	1.0E0
GOTERM_CC_FAT	membrane-bounded vesicle	RT	4	7.2E-1	1.0E0
GOTERM_CC_FAT	cytoplasmic vesicle	RT	4	7.2E-1	1.0E0
GOTERM_CC_FAT	ER to Golgi transport vesicle membrane	RT	3	7.4E-1	1.0E0
GOTERM_CC_FAT	ER to Golgi transport vesicle	RT	3	7.4E-1	1.0E0
GOTERM_CC_FAT	COPII vesicle coat	RT	3	7.4E-1	1.0E0
GOTERM_CC_FAT	endomembrane system	RT	8	8.5E-1	1.0E0
GOTERM_CC_FAT	Golgi apparatus part	RT	4	9.0E-1	1.0E0
GOTERM_BP_FAT	ER to Golgi vesicle-mediated transport	RT	3	9.5E-1	1.0E0
GOTERM_BP_FAT	Golgi vesicle transport	RT	3	9.5E-1	1.0E0
GOTERM_CC_FAT	coated membrane	RT	6	9.5E-1	1.0E0
GOTERM_CC_FAT	membrane coat	RT	6	9.5E-1	1.0E0
GOTERM_CC_FAT	Golgi apparatus	RT	4	9.8E-1	1.0E0
GOTERM_BP_FAT	establishment of protein localization	RT	14	9.9E-1	1.0E0
GOTERM_BP_FAT	protein transport	RT	14	9.9E-1	1.0E0
GOTERM_BP_FAT	intracellular protein transport	RT	9	9.9E-1	1.0E0
GOTERM_BP_FAT	cellular macromolecule localization	RT	9	9.9E-1	1.0E0
GOTERM_BP_FAT	cellular protein localization	RT	9	9.9E-1	1.0E0
GOTERM_BP_FAT	protein localization	RT	14	9.9E-1	1.0E0
GOTERM_BP_FAT	intracellular transport	RT	10	1.0E0	1.0E0
GOTERM_BP_FAT	vesicle-mediated transport	RT	10	1.0E0	1.0E0
Annotation Cluster 53	Enrichment Score: 0.09		Count	P_Value	Benjamini
INTERPRO	Annexin	RT	10	6.7E-1	1.0E0
GOTERM_MF_FAT	calcium-dependent phospholipid binding	RT	13	7.1E-1	1.0E0
SP_PIR_KEYWORDS	annexin	RT	10	7.3E-1	9.9E-1
GOTERM_MF_FAT	calcium ion binding	RT	24	7.8E-1	1.0E0
GOTERM_MF_FAT	phospholipid binding	RT	15	8.1E-1	1.0E0
INTERPRO	Annexin repeat	RT	8	8.2E-1	1.0E0
SMART	ANX	RT	8	8.3E-1	1.0E0
GOTERM_MF_FAT	lipid binding	RT	19	8.8E-1	1.0E0
INTERPRO	Alpha giardin	RT	6	9.3E-1	1.0E0
GOTERM_BP_FAT	cytoskeleton organization	RT	7	9.7E-1	1.0E0
Annotation Cluster 54	Enrichment Score: 0.09		Count	P_Value	Benjamini
GOTERM_BP_FAT	cofactor biosynthetic process	RT	6	6.8E-1	1.0E0
GOTERM_BP_FAT	cofactor metabolic process	RT	7	7.9E-1	1.0E0
GOTERM_BP_FAT	coenzyme biosynthetic process	RT	4	8.8E-1	1.0E0
GOTERM_BP_FAT	coenzyme metabolic process	RT	5	9.2E-1	1.0E0
Annotation Cluster 55	Enrichment Score: 0.09		Count	P_Value	Benjamini
INTERPRO	DNA-dependent ATPase MCM	RT	4	8.0E-1	1.0E0
SMART	MCM	RT	4	8.1E-1	1.0E0
INTERPRO	Nucleic acid-binding, OB-fold	RT	11	8.4E-1	1.0E0
Annotation Cluster 56	Enrichment Score: 0.09		Count	P_Value	Benjamini
INTERPRO	Ras	RT	8	6.2E-1	1.0E0
INTERPRO	Ras GTPase	RT	8	7.3E-1	1.0E0
PIR_SUPERFAMILY	PIRSF001710:Ras-related protein Rab	RT	4	8.0E-1	1.0E0
GOTERM_BP_FAT	small GTPase mediated signal transduction	RT	9	8.0E-1	1.0E0
SP_PIR_KEYWORDS	gtp-binding	RT	23	8.1E-1	9.9E-1
INTERPRO	Small GTP-binding protein	RT	11	8.4E-1	1.0E0
GOTERM_BP_FAT	intracellular signaling cascade	RT	11	8.7E-1	1.0E0

	GOTERM_MF_FAT	GTPase activity	RT			9	8.8E-1	1.0E0
	GOTERM_MF_FAT	GTP binding	RT			25	9.0E-1	1.0E0
	GOTERM_MF_FAT	guanyl ribonucleotide binding	RT			25	9.0E-1	1.0E0
	GOTERM_MF_FAT	guanyl nucleotide binding	RT			25	9.0E-1	1.0E0
	Annotation Cluster 57	Enrichment Score: 0.09	G			Count	P_Value	Benjamini
	INTERPRO	Aminoacyl-tRNA synthetase, class I, conserved site	RT			7	4.4E-1	1.0E0
	INTERPRO	Rossmann-like alpha/beta/alpha sandwich fold	RT			12	6.1E-1	1.0E0
	GOTERM_MF_FAT	ligase activity, forming carbon-oxygen bonds	RT			13	8.5E-1	1.0E0
	GOTERM_MF_FAT	ligase activity, forming aminoacyl-tRNA and related compounds	RT			13	8.5E-1	1.0E0
	GOTERM_MF_FAT	aminoacyl-tRNA ligase activity	RT			13	8.5E-1	1.0E0
	GOTERM_BP_FAT	tRNA metabolic process	RT			21	8.7E-1	1.0E0
	GOTERM_BP_FAT	tRNA aminoacylation	RT			12	8.7E-1	1.0E0
	GOTERM_BP_FAT	amino acid activation	RT			12	8.7E-1	1.0E0
	GOTERM_BP_FAT	ncRNA metabolic process	RT			27	8.9E-1	1.0E0
	GOTERM_BP_FAT	tRNA aminoacylation for protein translation	RT			11	9.1E-1	1.0E0
	SP_PIR_KEYWORDS	Aminoacyl-tRNA synthetase	RT			12	9.3E-1	1.0E0
	SP_PIR_KEYWORDS	protein biosynthesis	RT			5	9.5E-1	1.0E0
	KEGG_PATHWAY	Aminoacyl-tRNA biosynthesis	RT			10	9.9E-1	1.0E0
	Annotation Cluster 58	Enrichment Score: 0.07	G			Count	P_Value	Benjamini
	GOTERM_CC_FAT	organelle lumen	RT			3	8.5E-1	1.0E0
	GOTERM_CC_FAT	intracellular organelle lumen	RT			3	8.5E-1	1.0E0
	GOTERM_CC_FAT	membrane-enclosed lumen	RT			3	8.5E-1	1.0E0
	GOTERM_CC_FAT	nuclear lumen	RT			3	8.5E-1	1.0E0
	Annotation Cluster 59	Enrichment Score: 0.07	G			Count	P_Value	Benjamini
	GOTERM_MF_FAT	NAD or NADH binding	RT			6	5.5E-1	1.0E0
	GOTERM_BP_FAT	regulation of transcription, DNA-dependent	RT			7	7.9E-1	1.0E0
	GOTERM_BP_FAT	regulation of RNA metabolic process	RT			7	7.9E-1	1.0E0
	GOTERM_MF_FAT	hydrolase activity, acting on carbon-nitrogen (but not peptide) bonds, in linear amides	RT			5	8.0E-1	1.0E0
	GOTERM_BP_FAT	regulation of transcription	RT			17	8.0E-1	1.0E0
	GOTERM_BP_FAT	negative regulation of gene expression	RT			4	8.8E-1	1.0E0
	GOTERM_BP_FAT	negative regulation of macromolecule metabolic process	RT			4	8.8E-1	1.0E0
	GOTERM_BP_FAT	gene silencing	RT			4	8.8E-1	1.0E0
	GOTERM_BP_FAT	chromatin silencing	RT			3	8.9E-1	1.0E0
	GOTERM_BP_FAT	negative regulation of cellular biosynthetic process	RT			3	8.9E-1	1.0E0
	GOTERM_BP_FAT	negative regulation of macromolecule biosynthetic process	RT			3	8.9E-1	1.0E0
	GOTERM_BP_FAT	negative regulation of RNA metabolic process	RT			3	8.9E-1	1.0E0
	GOTERM_BP_FAT	negative regulation of biosynthetic process	RT			3	8.9E-1	1.0E0
	GOTERM_BP_FAT	regulation of gene expression, epigenetic	RT			3	8.9E-1	1.0E0
	GOTERM_BP_FAT	negative regulation of nitrogen compound metabolic process	RT			3	8.9E-1	1.0E0
	GOTERM_BP_FAT	negative regulation of nucleobase, nucleoside, nucleotide and nucleic acid metabolic process	RT			3	8.9E-1	1.0E0
	GOTERM_BP_FAT	negative regulation of transcription	RT			3	8.9E-1	1.0E0
	GOTERM_BP_FAT	negative regulation of gene expression, epigenetic	RT			3	8.9E-1	1.0E0
	GOTERM_BP_FAT	negative regulation of transcription, DNA-dependent	RT			3	8.9E-1	1.0E0
	GOTERM_MF_FAT	NAD binding	RT			3	9.0E-1	1.0E0
	INTERPRO	NAD-dependent histone deacetylase, silent information regulator Sir2	RT			3	9.0E-1	1.0E0
	GOTERM_BP_FAT	protein amino acid deacetylation	RT			3	9.5E-1	1.0E0
	Annotation Cluster 60	Enrichment Score: 0.06	G			Count	P_Value	Benjamini
	SP_PIR_KEYWORDS	Acyltransferase	RT			7	7.6E-1	9.9E-1
	INTERPRO	Acyl-CoA N-acyltransferase	RT			6	8.1E-1	1.0E0
	GOTERM_MF_FAT	N-acetyltransferase activity	RT			5	8.8E-1	1.0E0
	INTERPRO	GCN5-related N-acetyltransferase	RT			5	8.8E-1	1.0E0
	GOTERM_MF_FAT	N-acyltransferase activity	RT			5	9.3E-1	1.0E0
	GOTERM_MF_FAT	acetyltransferase activity	RT			5	9.3E-1	1.0E0
	Annotation Cluster 61	Enrichment Score: 0.06	G			Count	P_Value	Benjamini

GOTERM_BP_FAT	tRNA processing	RT	9	8.7E-1	1.0E0
GOTERM_BP_FAT	tRNA metabolic process	RT	21	8.7E-1	1.0E0
GOTERM_BP_FAT	RNA processing	RT	24	8.8E-1	1.0E0
GOTERM_BP_FAT	ncRNA processing	RT	15	8.8E-1	1.0E0
GOTERM_BP_FAT	ncRNA metabolic process	RT	27	8.9E-1	1.0E0
Annotation Cluster 62	Enrichment Score: 0.05		Count	P_Value	Benjamini
GOTERM_BP_FAT	rRNA processing	RT	7	8.6E-1	1.0E0
GOTERM_BP_FAT	rRNA metabolic process	RT	7	8.6E-1	1.0E0
GOTERM_BP_FAT	ncRNA processing	RT	15	8.8E-1	1.0E0
GOTERM_BP_FAT	ribosome biogenesis	RT	10	9.1E-1	1.0E0
GOTERM_BP_FAT	ribonucleoprotein complex biogenesis	RT	10	9.1E-1	1.0E0
Annotation Cluster 63	Enrichment Score: 0.05		Count	P_Value	Benjamini
GOTERM_BP_FAT	protein amino acid lipidation	RT	3	8.9E-1	1.0E0
GOTERM_BP_FAT	lipoprotein metabolic process	RT	3	8.9E-1	1.0E0
GOTERM_BP_FAT	lipoprotein biosynthetic process	RT	3	8.9E-1	1.0E0
Annotation Cluster 64	Enrichment Score: 0.05		Count	P_Value	Benjamini
GOTERM_BP_FAT	mismatch repair	RT	3	8.9E-1	1.0E0
GOTERM_MF_FAT	structure-specific DNA binding	RT	3	9.0E-1	1.0E0
GOTERM_MF_FAT	mismatched DNA binding	RT	3	9.0E-1	1.0E0
GOTERM_MF_FAT	double-stranded DNA binding	RT	3	9.0E-1	1.0E0
Annotation Cluster 65	Enrichment Score: 0.05		Count	P_Value	Benjamini
GOTERM_MF_FAT	intramolecular transferase activity	RT	6	8.8E-1	1.0E0
GOTERM_BP_FAT	pseudouridine synthesis	RT	4	8.8E-1	1.0E0
GOTERM_MF_FAT	pseudouridine synthase activity	RT	4	8.8E-1	1.0E0
GOTERM_BP_FAT	RNA modification	RT	7	9.7E-1	1.0E0
Annotation Cluster 66	Enrichment Score: 0.04		Count	P_Value	Benjamini
GOTERM_MF_FAT	GTPase activity	RT	9	8.8E-1	1.0E0
INTERPRO	Translation elongation factor EFTu/EF1A, domain 2	RT	3	9.0E-1	1.0E0
INTERPRO	Protein synthesis factor, GTP-binding	RT	4	9.4E-1	1.0E0
Annotation Cluster 67	Enrichment Score: 0.04		Count	P_Value	Benjamini
PIR_SUPERFAMILY	PIRSF001567:ubiquitin-protein ligase E2	RT	4	8.0E-1	1.0E0
KEGG_PATHWAY	Ubiquitin mediated proteolysis	RT	7	8.2E-1	9.9E-1
GOTERM_MF_FAT	small conjugating protein ligase activity	RT	7	8.8E-1	1.0E0
INTERPRO	Ubiquitin-conjugating enzyme, E2	RT	6	9.3E-1	1.0E0
INTERPRO	Ubiquitin-conjugating enzyme/RWD-like	RT	6	9.3E-1	1.0E0
GOTERM_MF_FAT	ligase activity, forming carbon-nitrogen bonds	RT	16	9.3E-1	1.0E0
SMART	UBCc	RT	6	9.3E-1	1.0E0
SP_PIR_KEYWORDS	ligase	RT	29	9.4E-1	1.0E0
GOTERM_MF_FAT	acid-amino acid ligase activity	RT	13	9.5E-1	1.0E0
SP_PIR_KEYWORDS	ubl conjugation pathway	RT	6	9.7E-1	1.0E0
Annotation Cluster 68	Enrichment Score: 0.04		Count	P_Value	Benjamini
GOTERM_MF_FAT	phosphoprotein phosphatase activity	RT	15	8.6E-1	1.0E0
GOTERM_BP_FAT	dephosphorylation	RT	8	9.1E-1	1.0E0
GOTERM_BP_FAT	protein amino acid dephosphorylation	RT	6	9.5E-1	1.0E0
Annotation Cluster 69	Enrichment Score: 0.04		Count	P_Value	Benjamini
GOTERM_BP_FAT	one-carbon metabolic process	RT	6	8.7E-1	1.0E0
GOTERM_BP_FAT	methylation	RT	5	9.2E-1	1.0E0
GOTERM_BP_FAT	biopolymer methylation	RT	3	9.5E-1	1.0E0
Annotation Cluster 70	Enrichment Score: 0.04		Count	P_Value	Benjamini
INTERPRO	RecF/RecN/SMC protein, N-terminal	RT	4	8.0E-1	1.0E0
GOTERM_CC_FAT	chromosome	RT	11	9.8E-1	1.0E0
GOTERM_BP_FAT	chromosome organization	RT	8	9.8E-1	1.0E0
Annotation Cluster 71	Enrichment Score: 0.04		Count	P_Value	Benjamini
GOTERM_MF_FAT	nucleoside-triphosphatase regulator activity	RT	8	7.3E-1	1.0E0
GOTERM_MF_FAT	small GTPase regulator activity	RT	6	8.8E-1	1.0E0
GOTERM_MF_FAT	GTPase regulator activity	RT	6	8.8E-1	1.0E0
GOTERM_BP_FAT	regulation of hydrolase activity	RT	4	9.3E-1	1.0E0
GOTERM_BP_FAT	regulation of GTPase activity	RT	4	9.3E-1	1.0E0
GOTERM_BP_FAT	regulation of Ras protein signal transduction	RT	4	9.6E-1	1.0E0

GOTERM_BP_FAT	regulation of small GTPase mediated signal transduction	RT	4	9.6E-1	1.0E0
GOTERM_MF_FAT	enzyme activator activity	RT	4	9.7E-1	1.0E0
GOTERM_BP_FAT	regulation of Ras GTPase activity	RT	3	9.7E-1	1.0E0
GOTERM_MF_FAT	GTPase activator activity	RT	3	9.8E-1	1.0E0
Annotation Cluster 72	Enrichment Score: 0.03		Count	P_Value	Benjamini
INTERPRO	RNA recognition motif, RNP-1	RT	7	9.3E-1	1.0E0
SMART	RRM	RT	7	9.3E-1	1.0E0
INTERPRO	Nucleotide-binding, alpha-beta plait	RT	7	9.7E-1	1.0E0
Annotation Cluster 73	Enrichment Score: 0.02		Count	P_Value	Benjamini
GOTERM_MF_FAT	thiolester hydrolase activity	RT	4	9.4E-1	1.0E0
GOTERM_MF_FAT	ubiquitin thiolesterase activity	RT	3	9.5E-1	1.0E0
INTERPRO	Peptidase C19, ubiquitin carboxyl-terminal hydrolase 2	RT	3	9.5E-1	1.0E0
Annotation Cluster 74	Enrichment Score: 0.02		Count	P_Value	Benjamini
GOTERM_MF_FAT	nuclease activity	RT	27	8.5E-1	1.0E0
GOTERM_MF_FAT	DNA polymerase activity	RT	17	9.3E-1	1.0E0
SP_PIR_KEYWORDS	Endonuclease	RT	15	9.6E-1	1.0E0
GOTERM_MF_FAT	endonuclease activity	RT	15	9.9E-1	1.0E0
GOTERM_MF_FAT	RNA-directed DNA polymerase activity	RT	10	9.9E-1	1.0E0
SP_PIR_KEYWORDS	rna-directed dna polymerase	RT	10	9.9E-1	1.0E0
Annotation Cluster 75	Enrichment Score: 0.02		Count	P_Value	Benjamini
INTERPRO	Protein phosphatase 2C	RT	3	9.5E-1	1.0E0
INTERPRO	Protein phosphatase 2C, N-terminal	RT	3	9.5E-1	1.0E0
INTERPRO	Protein phosphatase 2C-related	RT	3	9.5E-1	1.0E0
SMART	PP2C_SIG	RT	3	9.5E-1	1.0E0
SMART	PP2Cc	RT	3	9.5E-1	1.0E0
Annotation Cluster 76	Enrichment Score: 0.01		Count	P_Value	Benjamini
INTERPRO	Like-Sm ribonucleoprotein, core	RT	5	9.6E-1	1.0E0
INTERPRO	Like-Sm ribonucleoprotein, eukaryotic and archaea-type, core	RT	3	9.8E-1	1.0E0
SMART	Sm	RT	3	9.8E-1	1.0E0
Annotation Cluster 77	Enrichment Score: 0.01		Count	P_Value	Benjamini
SP_PIR_KEYWORDS	Initiation factor	RT	9	9.7E-1	1.0E0
GOTERM_MF_FAT	translation initiation factor activity	RT	11	9.8E-1	1.0E0
GOTERM_BP_FAT	translational initiation	RT	4	9.9E-1	1.0E0
GOTERM_MF_FAT	translation factor activity, nucleic acid binding	RT	12	1.0E0	1.0E0
Annotation Cluster 78	Enrichment Score: 0.01		Count	P_Value	Benjamini
GOTERM_BP_FAT	protein polymerization	RT	4	9.3E-1	1.0E0
INTERPRO	Tubulin/FtsZ, GTPase domain	RT	4	9.4E-1	1.0E0
INTERPRO	Tubulin	RT	4	9.4E-1	1.0E0
INTERPRO	Tubulin, conserved site	RT	4	9.4E-1	1.0E0
PIR_SUPERFAMILY	PIRSF002306:tubulin	RT	3	9.8E-1	1.0E0
INTERPRO	Tubulin/FtsZ, 2-layer sandwich domain	RT	3	9.8E-1	1.0E0
GOTERM_BP_FAT	chromosome organization	RT	8	9.8E-1	1.0E0
GOTERM_BP_FAT	protein complex biogenesis	RT	5	9.9E-1	1.0E0
GOTERM_BP_FAT	protein complex assembly	RT	5	9.9E-1	1.0E0
GOTERM_BP_FAT	cellular protein complex assembly	RT	4	9.9E-1	1.0E0
GOTERM_BP_FAT	chromatin organization	RT	5	1.0E0	1.0E0
GOTERM_CC_FAT	nucleosome	RT	3	1.0E0	1.0E0
GOTERM_BP_FAT	chromatin assembly or disassembly	RT	4	1.0E0	1.0E0
GOTERM_CC_FAT	chromosomal part	RT	5	1.0E0	1.0E0
GOTERM_BP_FAT	macromolecular complex assembly	RT	9	1.0E0	1.0E0
GOTERM_CC_FAT	chromatin	RT	3	1.0E0	1.0E0
GOTERM_BP_FAT	chromatin assembly	RT	3	1.0E0	1.0E0
GOTERM_BP_FAT	protein-DNA complex assembly	RT	3	1.0E0	1.0E0
GOTERM_BP_FAT	nucleosome organization	RT	3	1.0E0	1.0E0
GOTERM_BP_FAT	DNA packaging	RT	3	1.0E0	1.0E0
GOTERM_BP_FAT	nucleosome assembly	RT	3	1.0E0	1.0E0
GOTERM_BP_FAT	cellular macromolecular complex assembly	RT	8	1.0E0	1.0E0
INTERPRO	Histone core	RT	3	1.0E0	1.0E0

	GOTERM_BP_FAT	macromolecular complex subunit organization	RT		9	1.0E0	1.0E0
	GOTERM_BP_FAT	cellular macromolecular complex subunit organization	RT		8	1.0E0	1.0E0
	SP_PIR_KEYWORDS	chromosomal protein	RT		3	1.0E0	1.0E0
	GOTERM_CC_FAT	protein-DNA complex	RT		3	1.0E0	1.0E0
	INTERPRO	Histone-fold	RT		3	1.0E0	1.0E0
	Annotation Cluster 79	Enrichment Score: 0	G		Count	P_Value	Benjamini
	INTERPRO	Zinc finger, C2H2-like	RT		7	9.9E-1	1.0E0
	INTERPRO	Zinc finger, C2H2-type	RT		7	9.9E-1	1.0E0
	SMART	ZnF_C2H2	RT		7	9.9E-1	1.0E0

201 terms were not clustered.

Please cite *Nature Protocols 2009; 4(1):44 & Genome Biology 2003; 4(5):P3* within any publication that makes use of any methods inspired by **DAVID**.

SAIC SAIC Frederick NIAID ABCC NCI-Frederick NATIONAL INSTITUTES OF HEALTH FIRSTGOV Your First Click to the U.S. Government Department of Health and Human Services

DAVID Bioinformatics Resources 6.7
National Institute of Allergy and Infectious Diseases (NIAID), NIH

Functional Annotation Clustering

Help and Manual

Current Gene List: 3_Random10
Current Background: Giardia intestinalis
3000 DAVID IDs

Options   Classification Stringency Medium

Rerun using options   Create Sublist

83 Cluster(s)   Download File

Category	Term		Count	P_Value	Benjamini
Annotation Cluster 1	Enrichment Score: 1.03		Count	P_Value	Benjamini
GOTERM_BP_FAT	cytoskeleton organization	RT	14	4.3E-2	1.0E0
SP_PIR_KEYWORDS	annexin	RT	14	4.3E-2	9.9E-1
GOTERM_MF_FAT	calcium-dependent phospholipid binding	RT	18	4.6E-2	1.0E0
INTERPRO	Annexin	RT	14	5.0E-2	1.0E0
INTERPRO	Alpha giardin	RT	11	6.8E-2	1.0E0
SMART	ANX	RT	12	9.9E-2	1.0E0
INTERPRO	Annexin repeat	RT	12	1.0E-1	1.0E0
GOTERM_MF_FAT	calcium ion binding	RT	30	1.2E-1	1.0E0
GOTERM_MF_FAT	phospholipid binding	RT	18	3.6E-1	1.0E0
GOTERM_MF_FAT	lipid binding	RT	23	4.1E-1	1.0E0
Annotation Cluster 2	Enrichment Score: 0.66		Count	P_Value	Benjamini
GOTERM_BP_FAT	protein localization	RT	25	8.1E-2	1.0E0
GOTERM_BP_FAT	intracellular transport	RT	21	9.3E-2	1.0E0
GOTERM_BP_FAT	protein transport	RT	24	1.0E-1	1.0E0
GOTERM_BP_FAT	establishment of protein localization	RT	24	1.0E-1	1.0E0
GOTERM_BP_FAT	intracellular protein transport	RT	17	1.7E-1	1.0E0
GOTERM_BP_FAT	cellular protein localization	RT	17	1.7E-1	1.0E0
GOTERM_BP_FAT	cellular macromolecule localization	RT	17	1.7E-1	1.0E0
GOTERM_CC_FAT	Golgi apparatus	RT	9	2.7E-1	1.0E0
GOTERM_BP_FAT	vesicle-mediated transport	RT	19	3.8E-1	1.0E0
INTERPRO	Clathrin/coatomer adaptor, adaptin-like, N-terminal	RT	4	6.6E-1	1.0E0
GOTERM_CC_FAT	coated membrane	RT	9	6.7E-1	1.0E0
GOTERM_CC_FAT	membrane coat	RT	9	6.7E-1	1.0E0
Annotation Cluster 3	Enrichment Score: 0.63		Count	P_Value	Benjamini
GOTERM_CC_FAT	integral to membrane	RT	44	1.6E-1	1.0E0
GOTERM_CC_FAT	intrinsic to membrane	RT	44	1.6E-1	1.0E0
SP_PIR_KEYWORDS	transmembrane	RT	20	3.3E-1	1.0E0
SP_PIR_KEYWORDS	membrane	RT	18	3.6E-1	1.0E0
Annotation Cluster 4	Enrichment Score: 0.62		Count	P_Value	Benjamini
SMART	AAA	RT	47	1.6E-1	1.0E0
INTERPRO	ATPase, AAA+ type, core	RT	47	1.6E-1	1.0E0
INTERPRO	ATPase, AAA-type, core	RT	16	5.2E-1	1.0E0
Annotation Cluster 5	Enrichment Score: 0.51		Count	P_Value	Benjamini
INTERPRO	AMP-dependent synthetase and ligase	RT	7	1.4E-1	1.0E0
GOTERM_MF_FAT	fatty-acid ligase activity	RT	5	3.0E-1	1.0E0
GOTERM_MF_FAT	long-chain-fatty-acid-CoA ligase activity	RT	5	3.0E-1	1.0E0
GOTERM_MF_FAT	ligase activity, forming carbon-sulfur bonds	RT	5	3.0E-1	1.0E0
COG_ONTOLOGY	Lipid metabolism	RT	8	7.3E-1	9.9E-1
Annotation Cluster 6	Enrichment Score: 0.43		Count	P_Value	Benjamini
GOTERM_BP_FAT	chromatin assembly	RT	9	2.4E-1	1.0E0
GOTERM_BP_FAT	protein-DNA complex assembly	RT	9	2.4E-1	1.0E0
GOTERM_BP_FAT	DNA packaging	RT	9	2.4E-1	1.0E0
GOTERM_BP_FAT	nucleosome assembly	RT	9	2.4E-1	1.0E0
GOTERM_BP_FAT	nucleosome organization	RT	9	2.4E-1	1.0E0
INTERPRO	Histone-fold	RT	11	2.4E-1	1.0E0

Category	Term		Count	P_Value	Benjamini
SP_PIR_KEYWORDS	chromosomal protein	RT	9	2.4E-1	1.0E0
SP_PIR_KEYWORDS	nucleus	RT	23	2.5E-1	1.0E0
INTERPRO	Histone core	RT	9	2.6E-1	1.0E0
GOTERM_CC_FAT	nucleosome	RT	9	2.7E-1	1.0E0
GOTERM_BP_FAT	chromatin assembly or disassembly	RT	10	2.9E-1	1.0E0
SP_PIR_KEYWORDS	dna-binding	RT	23	3.2E-1	1.0E0
GOTERM_CC_FAT	protein-DNA complex	RT	10	3.2E-1	1.0E0
GOTERM_BP_FAT	cellular macromolecular complex subunit organization	RT	17	3.3E-1	1.0E0
GOTERM_BP_FAT	chromatin organization	RT	11	3.3E-1	1.0E0
GOTERM_BP_FAT	macromolecular complex subunit organization	RT	18	3.6E-1	1.0E0
SP_PIR_KEYWORDS	nucleosome core	RT	6	3.7E-1	1.0E0
GOTERM_BP_FAT	cellular macromolecular complex assembly	RT	16	3.9E-1	1.0E0
GOTERM_CC_FAT	chromatin	RT	9	4.1E-1	1.0E0
GOTERM_CC_FAT	chromosomal part	RT	12	4.1E-1	1.0E0
GOTERM_BP_FAT	macromolecular complex assembly	RT	17	4.2E-1	1.0E0
GOTERM_BP_FAT	chromosome organization	RT	12	5.8E-1	1.0E0
SMART	H4	RT	3	6.2E-1	1.0E0
INTERPRO	Histone H4	RT	3	6.3E-1	1.0E0
PIR_SUPERFAMILY	PIRSF002052:histone H4	RT	3	6.3E-1	1.0E0
SMART	H3	RT	3	8.0E-1	1.0E0
INTERPRO	Histone H3	RT	3	8.0E-1	1.0E0
GOTERM_CC_FAT	chromosome	RT	14	9.2E-1	1.0E0
Annotation Cluster 7	Enrichment Score: 0.43		Count	P_Value	Benjamini
SMART	ANK	RT	259	3.0E-1	1.0E0
INTERPRO	Ankyrin	RT	259	3.4E-1	1.0E0
SP_PIR_KEYWORDS	ank repeat	RT	177	5.0E-1	1.0E0
Annotation Cluster 8	Enrichment Score: 0.41		Count	P_Value	Benjamini
SMART	UBQ	RT	6	3.8E-1	1.0E0
INTERPRO	Ubiquitin	RT	6	3.9E-1	1.0E0
INTERPRO	Ubiquitin supergroup	RT	9	3.9E-1	1.0E0
Annotation Cluster 9	Enrichment Score: 0.41		Count	P_Value	Benjamini
GOTERM_MF_FAT	protein kinase regulator activity	RT	6	3.7E-1	1.0E0
GOTERM_MF_FAT	kinase regulator activity	RT	6	3.7E-1	1.0E0
GOTERM_MF_FAT	cAMP-dependent protein kinase regulator activity	RT	4	4.4E-1	1.0E0
Annotation Cluster 10	Enrichment Score: 0.4		Count	P_Value	Benjamini
PIR_SUPERFAMILY	PIRSF001711:ADP-ribosylation factor	RT	5	3.2E-1	1.0E0
INTERPRO	ARF/SAR superfamily	RT	6	3.9E-1	1.0E0
SMART	ARF	RT	4	4.5E-1	1.0E0
INTERPRO	ADP-ribosylation factor	RT	4	4.5E-1	1.0E0
Annotation Cluster 11	Enrichment Score: 0.39		Count	P_Value	Benjamini
GOTERM_BP_FAT	intracellular signaling cascade	RT	16	1.4E-1	1.0E0
GOTERM_BP_FAT	small GTPase mediated signal transduction	RT	12	2.6E-1	1.0E0
GOTERM_MF_FAT	guanyl ribonucleotide binding	RT	31	3.0E-1	1.0E0
GOTERM_MF_FAT	GTP binding	RT	31	3.0E-1	1.0E0
GOTERM_MF_FAT	guanyl nucleotide binding	RT	31	3.0E-1	1.0E0
INTERPRO	Small GTP-binding protein	RT	14	3.6E-1	1.0E0
INTERPRO	Ras	RT	9	3.9E-1	1.0E0
INTERPRO	Ras GTPase	RT	9	5.3E-1	1.0E0
SP_PIR_KEYWORDS	gtp-binding	RT	24	5.7E-1	9.9E-1
SMART	RAB	RT	3	8.0E-1	1.0E0
INTERPRO	Ras small GTPase, Rab type	RT	3	8.0E-1	1.0E0
PIR_SUPERFAMILY	PIRSF001710:Ras-related protein Rab	RT	4	8.1E-1	1.0E0
Annotation Cluster 12	Enrichment Score: 0.35		Count	P_Value	Benjamini
COG_ONTOLOGY	Nucleotide transport and metabolism	RT	8	8.7E-2	9.5E-1
GOTERM_BP_FAT	nucleotide catabolic process	RT	4	4.3E-1	1.0E0
GOTERM_BP_FAT	nucleobase, nucleoside, nucleotide and nucleic acid catabolic process	RT	5	4.9E-1	1.0E0
GOTERM_BP_FAT	nucleobase, nucleoside and nucleotide catabolic process	RT	5	4.9E-1	1.0E0

GOTERM_BP_FAT	nitrogen compound catabolic process	RT	5	4.9E-1	1.0E0
INTERPRO	5'-Nucleotidase and apyrase	RT	3	6.3E-1	1.0E0
INTERPRO	Predicted phosphoesterase, C1039.02 type	RT	3	6.3E-1	1.0E0
PIR_SUPERFAMILY	PIRSF017316:phosphoesterase, C1039.02 type	RT	3	6.3E-1	1.0E0
PIR_SUPERFAMILY	PIRSF017316:Pesterase_C1039	RT	3	6.3E-1	1.0E0
Annotation Cluster 13	Enrichment Score: 0.35	G	Count	P_Value	Benjamini
SMART	RRM	RT	10	4.3E-1	1.0E0
INTERPRO	RNA recognition motif, RNP-1	RT	10	4.4E-1	1.0E0
INTERPRO	Nucleotide-binding, alpha-beta plait	RT	11	4.8E-1	1.0E0
Annotation Cluster 14	Enrichment Score: 0.34	G	Count	P_Value	Benjamini
SMART	VWA	RT	6	3.8E-1	1.0E0
INTERPRO	von Willebrand factor, type A	RT	6	3.9E-1	1.0E0
INTERPRO	Copine	RT	4	6.6E-1	1.0E0
Annotation Cluster 15	Enrichment Score: 0.31	G	Count	P_Value	Benjamini
GOTERM_CC_FAT	organelle membrane	RT	9	2.7E-1	1.0E0
GOTERM_CC_FAT	Golgi apparatus	RT	9	2.7E-1	1.0E0
GOTERM_CC_FAT	endomembrane system	RT	12	3.0E-1	1.0E0
GOTERM_CC_FAT	ER to Golgi transport vesicle membrane	RT	4	4.6E-1	1.0E0
GOTERM_CC_FAT	ER to Golgi transport vesicle	RT	4	4.6E-1	1.0E0
GOTERM_CC_FAT	COPII vesicle coat	RT	4	4.6E-1	1.0E0
GOTERM_BP_FAT	ER to Golgi vesicle-mediated transport	RT	5	4.9E-1	1.0E0
GOTERM_BP_FAT	Golgi vesicle transport	RT	5	4.9E-1	1.0E0
GOTERM_CC_FAT	cytoplasmic membrane-bounded vesicle	RT	5	5.2E-1	1.0E0
GOTERM_CC_FAT	vesicle membrane	RT	5	5.2E-1	1.0E0
GOTERM_CC_FAT	membrane-bounded vesicle	RT	5	5.2E-1	1.0E0
GOTERM_CC_FAT	cytoplasmic vesicle	RT	5	5.2E-1	1.0E0
GOTERM_CC_FAT	vesicle	RT	5	5.2E-1	1.0E0
GOTERM_CC_FAT	cytoplasmic vesicle membrane	RT	5	5.2E-1	1.0E0
GOTERM_CC_FAT	coated vesicle membrane	RT	5	5.2E-1	1.0E0
GOTERM_CC_FAT	coated vesicle	RT	5	5.2E-1	1.0E0
GOTERM_CC_FAT	cytoplasmic vesicle part	RT	5	5.2E-1	1.0E0
GOTERM_CC_FAT	vesicle coat	RT	5	5.2E-1	1.0E0
GOTERM_CC_FAT	Golgi apparatus part	RT	6	5.7E-1	1.0E0
GOTERM_CC_FAT	transport vesicle membrane	RT	4	6.7E-1	1.0E0
GOTERM_CC_FAT	transport vesicle	RT	4	6.7E-1	1.0E0
GOTERM_CC_FAT	coated membrane	RT	9	6.7E-1	1.0E0
GOTERM_CC_FAT	membrane coat	RT	9	6.7E-1	1.0E0
Annotation Cluster 16	Enrichment Score: 0.29	G	Count	P_Value	Benjamini
GOTERM_MF_FAT	nucleobase, nucleoside, nucleotide kinase activity	RT	8	3.3E-1	1.0E0
GOTERM_MF_FAT	nucleotide kinase activity	RT	4	6.4E-1	1.0E0
GOTERM_MF_FAT	phosphotransferase activity, phosphate group as acceptor	RT	5	6.6E-1	1.0E0
Annotation Cluster 17	Enrichment Score: 0.27	G	Count	P_Value	Benjamini
GOTERM_BP_FAT	purine nucleotide metabolic process	RT	20	3.2E-1	1.0E0
GOTERM_BP_FAT	ATP metabolic process	RT	17	3.3E-1	1.0E0
GOTERM_BP_FAT	ATP biosynthetic process	RT	17	3.3E-1	1.0E0
GOTERM_BP_FAT	nucleoside triphosphate biosynthetic process	RT	18	3.6E-1	1.0E0
GOTERM_BP_FAT	nucleoside triphosphate metabolic process	RT	18	3.6E-1	1.0E0
GOTERM_BP_FAT	purine nucleoside triphosphate biosynthetic process	RT	18	3.6E-1	1.0E0
GOTERM_BP_FAT	purine nucleoside triphosphate metabolic process	RT	18	3.6E-1	1.0E0
GOTERM_BP_FAT	ribonucleoside triphosphate metabolic process	RT	18	3.6E-1	1.0E0
GOTERM_BP_FAT	ribonucleoside triphosphate biosynthetic process	RT	18	3.6E-1	1.0E0
GOTERM_BP_FAT	purine ribonucleoside triphosphate metabolic process	RT	18	3.6E-1	1.0E0
GOTERM_BP_FAT	purine ribonucleoside triphosphate biosynthetic process	RT	18	3.6E-1	1.0E0
GOTERM_MF_FAT	ATPase activity, coupled to transmembrane movement of ions, phosphorylative mechanism	RT	9	3.7E-1	1.0E0
GOTERM_BP_FAT	purine nucleotide biosynthetic process	RT	19	3.8E-1	1.0E0

GOTERM_BP_FAT	ion transport	RT	16	3.9E-1	1.0E0
GOTERM_BP_FAT	cation transport	RT	16	3.9E-1	1.0E0
INTERPRO	ATPase, P-type, K/Mg/Cd/Cu/Zn/Na/Ca/Na/H-transporter	RT	9	3.9E-1	1.0E0
GOTERM_BP_FAT	purine ribonucleotide metabolic process	RT	18	4.4E-1	1.0E0
GOTERM_BP_FAT	purine ribonucleotide biosynthetic process	RT	18	4.4E-1	1.0E0
GOTERM_BP_FAT	ribonucleotide metabolic process	RT	18	4.4E-1	1.0E0
GOTERM_BP_FAT	ribonucleotide biosynthetic process	RT	18	4.4E-1	1.0E0
GOTERM_CC_FAT	proton-transporting V-type ATPase complex	RT	7	4.6E-1	1.0E0
GOTERM_MF_FAT	inorganic cation transmembrane transporter activity	RT	12	4.8E-1	1.0E0
INTERPRO	ATPase, P-type, ATPase-associated region	RT	8	4.9E-1	1.0E0
GOTERM_BP_FAT	nucleotide biosynthetic process	RT	24	5.0E-1	1.0E0
GOTERM_CC_FAT	proton-transporting two-sector ATPase complex, proton-transporting domain	RT	5	5.2E-1	1.0E0
GOTERM_BP_FAT	hydrogen transport	RT	10	5.3E-1	1.0E0
GOTERM_BP_FAT	proton transport	RT	10	5.3E-1	1.0E0
KEGG_PATHWAY	Oxidative phosphorylation	RT	9	5.3E-1	1.0E0
INTERPRO	ATPase, P-type phosphorylation site	RT	6	5.6E-1	1.0E0
GOTERM_BP_FAT	generation of precursor metabolites and energy	RT	16	5.8E-1	1.0E0
GOTERM_CC_FAT	proton-transporting two-sector ATPase complex	RT	10	5.8E-1	1.0E0
GOTERM_BP_FAT	nitrogen compound biosynthetic process	RT	29	5.8E-1	1.0E0
GOTERM_BP_FAT	nucleobase, nucleoside, nucleotide and nucleic acid biosynthetic process	RT	25	5.9E-1	1.0E0
GOTERM_BP_FAT	nucleobase, nucleoside and nucleotide biosynthetic process	RT	25	5.9E-1	1.0E0
GOTERM_MF_FAT	di-, tri-valent inorganic cation transmembrane transporter activity	RT	3	6.1E-1	1.0E0
GOTERM_MF_FAT	hydrolase activity, acting on acid anhydrides, catalyzing transmembrane movement of substances	RT	18	6.2E-1	1.0E0
GOTERM_BP_FAT	ion transmembrane transport	RT	9	6.2E-1	1.0E0
INTERPRO	ATPase, P-type cation-transporter, C-terminal	RT	3	6.3E-1	1.0E0
INTERPRO	ATPase, P-type cation-transporter, N-terminal	RT	3	6.3E-1	1.0E0
GOTERM_MF_FAT	metal ion transmembrane transporter activity	RT	4	6.4E-1	1.0E0
GOTERM_BP_FAT	monovalent inorganic cation transport	RT	10	6.4E-1	1.0E0
GOTERM_CC_FAT	proton-transporting V-type ATPase, V0 domain	RT	4	6.7E-1	1.0E0
GOTERM_MF_FAT	ATPase activity, coupled to transmembrane movement of ions	RT	12	6.9E-1	1.0E0
GOTERM_BP_FAT	oxidative phosphorylation	RT	8	7.1E-1	1.0E0
GOTERM_BP_FAT	ATP synthesis coupled proton transport	RT	8	7.1E-1	1.0E0
GOTERM_BP_FAT	energy coupled proton transport, down electrochemical gradient	RT	8	7.1E-1	1.0E0
GOTERM_MF_FAT	hydrogen ion transmembrane transporter activity	RT	9	7.3E-1	1.0E0
GOTERM_MF_FAT	monovalent inorganic cation transmembrane transporter activity	RT	9	7.3E-1	1.0E0
GOTERM_MF_FAT	ATPase activity, coupled to transmembrane movement of substances	RT	16	7.4E-1	1.0E0
GOTERM_MF_FAT	ATPase activity, coupled to movement of substances	RT	16	7.4E-1	1.0E0
GOTERM_MF_FAT	primary active transmembrane transporter activity	RT	17	7.5E-1	1.0E0
GOTERM_MF_FAT	P-P-bond-hydrolysis-driven transmembrane transporter activity	RT	17	7.5E-1	1.0E0
GOTERM_BP_FAT	transmembrane transport	RT	12	7.6E-1	1.0E0
SP_PIR_KEYWORDS	phosphoprotein	RT	3	7.9E-1	9.9E-1
SP_PIR_KEYWORDS	ion transport	RT	3	8.9E-1	9.9E-1
GOTERM_CC_FAT	proton-transporting two-sector ATPase complex, catalytic domain	RT	4	9.5E-1	1.0E0
GOTERM_MF_FAT	proton-transporting ATPase activity, rotational mechanism	RT	3	9.7E-1	1.0E0
Annotation Cluster 18	Enrichment Score: 0.27		Count	P_Value	Benjamini
GOTERM_BP_FAT	cellular response to stress	RT	14	4.3E-1	1.0E0
GOTERM_BP_FAT	DNA repair	RT	13	5.1E-1	1.0E0
GOTERM_BP_FAT	response to DNA damage stimulus	RT	13	5.1E-1	1.0E0
GOTERM_BP_FAT	DNA metabolic process	RT	33	7.5E-1	1.0E0
Annotation Cluster 19	Enrichment Score: 0.27		Count	P_Value	Benjamini
GOTERM_CC_FAT	endomembrane system	RT	12	3.0E-1	1.0E0
GOTERM_BP_FAT	nuclear transport	RT	4	4.3E-1	1.0E0

GOTERM_BP_FAT	nucleocytoplasmic transport	RT		4	4.3E-1	1.0E0
GOTERM_CC_FAT	organelle envelope	RT		4	6.7E-1	1.0E0
GOTERM_CC_FAT	envelope	RT		4	6.7E-1	1.0E0
GOTERM_CC_FAT	pore complex	RT		4	6.7E-1	1.0E0
GOTERM_CC_FAT	nuclear envelope	RT		4	6.7E-1	1.0E0
GOTERM_CC_FAT	nuclear pore	RT		4	6.7E-1	1.0E0
Annotation Cluster 20	Enrichment Score: 0.26	G		Count	P_Value	Benjamini
SMART	t_SNARE	RT		4	4.5E-1	1.0E0
INTERPRO	Target SNARE coiled-coil region	RT		4	4.5E-1	1.0E0
KEGG_PATHWAY	SNARE interactions in vesicular transport	RT		3	8.1E-1	1.0E0
Annotation Cluster 21	Enrichment Score: 0.26	G		Count	P_Value	Benjamini
GOTERM_MF_FAT	ion binding	RT		155	2.4E-1	1.0E0
GOTERM_MF_FAT	cation binding	RT		155	2.4E-1	1.0E0
GOTERM_MF_FAT	metal ion binding	RT		152	2.9E-1	1.0E0
GOTERM_MF_FAT	zinc ion binding	RT		90	4.1E-1	1.0E0
GOTERM_MF_FAT	transition metal ion binding	RT		107	6.5E-1	1.0E0
SP_PIR_KEYWORDS	metal-binding	RT		57	6.7E-1	9.9E-1
SP_PIR_KEYWORDS	zinc	RT		46	8.1E-1	9.9E-1
SP_PIR_KEYWORDS	zinc-finger	RT		33	8.3E-1	9.9E-1
SMART	RING	RT		36	8.8E-1	1.0E0
INTERPRO	Zinc finger, RING-type	RT		36	8.8E-1	1.0E0
INTERPRO	Zinc finger, C3HC4 RING-type	RT		18	9.1E-1	1.0E0
Annotation Cluster 22	Enrichment Score: 0.26	G		Count	P_Value	Benjamini
GOTERM_BP_FAT	rRNA processing	RT		9	5.0E-1	1.0E0
GOTERM_BP_FAT	rRNA metabolic process	RT		9	5.0E-1	1.0E0
GOTERM_BP_FAT	ribonucleoprotein complex biogenesis	RT		13	5.1E-1	1.0E0
GOTERM_BP_FAT	ribosome biogenesis	RT		13	5.1E-1	1.0E0
GOTERM_BP_FAT	ncRNA processing	RT		18	5.3E-1	1.0E0
GOTERM_BP_FAT	rRNA modification	RT		3	6.1E-1	1.0E0
GOTERM_BP_FAT	RNA processing	RT		26	7.3E-1	1.0E0
Annotation Cluster 23	Enrichment Score: 0.26	G		Count	P_Value	Benjamini
GOTERM_MF_FAT	protein phosphatase regulator activity	RT		4	4.4E-1	1.0E0
GOTERM_MF_FAT	phosphatase regulator activity	RT		4	4.4E-1	1.0E0
GOTERM_MF_FAT	protein phosphatase type 2A regulator activity	RT		3	6.1E-1	1.0E0
GOTERM_CC_FAT	protein phosphatase type 2A complex	RT		3	6.3E-1	1.0E0
GOTERM_CC_FAT	protein serine/threonine phosphatase complex	RT		5	6.9E-1	1.0E0
Annotation Cluster 24	Enrichment Score: 0.26	G		Count	P_Value	Benjamini
SMART	HATPase_c	RT		6	3.8E-1	1.0E0
INTERPRO	ATP-binding region, ATPase-like	RT		6	3.9E-1	1.0E0
INTERPRO	DNA mismatch repair protein	RT		3	6.3E-1	1.0E0
GOTERM_BP_FAT	mismatch repair	RT		4	6.4E-1	1.0E0
GOTERM_MF_FAT	double-stranded DNA binding	RT		4	6.4E-1	1.0E0
GOTERM_MF_FAT	structure-specific DNA binding	RT		4	6.4E-1	1.0E0
GOTERM_MF_FAT	mismatched DNA binding	RT		4	6.4E-1	1.0E0
Annotation Cluster 25	Enrichment Score: 0.25	G		Count	P_Value	Benjamini
GOTERM_MF_FAT	transcription regulator activity	RT		8	4.7E-1	1.0E0
SMART	ZnF_C2C2	RT		3	6.2E-1	1.0E0
INTERPRO	Zinc finger, TFIIS-type	RT		3	6.3E-1	1.0E0
Annotation Cluster 26	Enrichment Score: 0.24	G		Count	P_Value	Benjamini
GOTERM_MF_FAT	heat shock protein binding	RT		6	5.4E-1	1.0E0
SMART	DnaJ	RT		6	5.5E-1	1.0E0
INTERPRO	Heat shock protein DnaJ, N-terminal	RT		6	5.6E-1	1.0E0
INTERPRO	Molecular chaperone, heat shock protein, Hsp40, DnaJ	RT		5	6.8E-1	1.0E0
Annotation Cluster 27	Enrichment Score: 0.23	G		Count	P_Value	Benjamini
GOTERM_MF_FAT	structural constituent of ribosome	RT		42	4.3E-1	1.0E0
KEGG_PATHWAY	Ribosome	RT		42	4.3E-1	1.0E0
SP_PIR_KEYWORDS	ribosomal protein	RT		43	5.0E-1	1.0E0

	Category	Term				Count	P_Value	Benjamini
☐	GOTERM_BP_FAT	translation	RT			66	5.0E-1	1.0E0
☐	GOTERM_MF_FAT	structural molecule activity	RT			49	5.4E-1	1.0E0
☐	GOTERM_CC_FAT	ribonucleoprotein complex	RT			59	5.9E-1	1.0E0
☐	GOTERM_CC_FAT	ribosome	RT			44	6.8E-1	1.0E0
☐	GOTERM_CC_FAT	intracellular non-membrane-bounded organelle	RT			87	9.6E-1	1.0E0
☐	GOTERM_CC_FAT	non-membrane-bounded organelle	RT			87	9.6E-1	1.0E0
	Annotation Cluster 28	Enrichment Score: 0.21	G			Count	P_Value	Benjamini
☐	SP_PIR_KEYWORDS	ligase	RT			34	4.1E-1	1.0E0
☐	GOTERM_MF_FAT	small conjugating protein ligase activity	RT			9	5.1E-1	1.0E0
☐	SMART	UBCc	RT			8	6.2E-1	1.0E0
☐	INTERPRO	Ubiquitin-conjugating enzyme/RWD-like	RT			8	6.2E-1	1.0E0
☐	INTERPRO	Ubiquitin-conjugating enzyme, E2	RT			8	6.2E-1	1.0E0
☐	GOTERM_MF_FAT	ubiquitin-protein ligase activity	RT			4	6.4E-1	1.0E0
☐	GOTERM_MF_FAT	acid-amino acid ligase activity	RT			16	6.7E-1	1.0E0
☐	SP_PIR_KEYWORDS	ubl conjugation pathway	RT			8	7.1E-1	9.9E-1
☐	GOTERM_MF_FAT	ligase activity, forming carbon-nitrogen bonds	RT			18	7.6E-1	1.0E0
	Annotation Cluster 29	Enrichment Score: 0.2	G			Count	P_Value	Benjamini
☐	GOTERM_MF_FAT	exopeptidase activity	RT			8	4.7E-1	1.0E0
☐	GOTERM_MF_FAT	metallopeptidase activity	RT			9	5.1E-1	1.0E0
☐	SP_PIR_KEYWORDS	dipeptidase	RT			3	6.1E-1	9.9E-1
☐	GOTERM_MF_FAT	dipeptidase activity	RT			3	6.1E-1	1.0E0
☐	SMART	Zn_pept	RT			4	6.5E-1	1.0E0
☐	INTERPRO	Peptidase M14, carboxypeptidase A	RT			4	6.6E-1	1.0E0
☐	GOTERM_MF_FAT	metalloexopeptidase activity	RT			5	6.6E-1	1.0E0
☐	GOTERM_MF_FAT	carboxypeptidase activity	RT			3	7.9E-1	1.0E0
☐	GOTERM_MF_FAT	metallocarboxypeptidase activity	RT			3	7.9E-1	1.0E0
	Annotation Cluster 30	Enrichment Score: 0.2	G			Count	P_Value	Benjamini
☐	INTERPRO	WD40/YVTN repeat-like	RT			45	3.7E-1	1.0E0
☐	SMART	WD40	RT			45	4.7E-1	1.0E0
☐	INTERPRO	WD40 repeat	RT			45	4.8E-1	1.0E0
☐	INTERPRO	WD40 repeat, conserved site	RT			17	7.1E-1	1.0E0
☐	INTERPRO	WD40 repeat, subgroup	RT			32	7.3E-1	1.0E0
☐	SP_PIR_KEYWORDS	wd repeat	RT			27	7.9E-1	9.9E-1
☐	INTERPRO	WD40 repeat 2	RT			23	8.7E-1	1.0E0
☐	INTERPRO	WD40 repeat, region	RT			25	8.8E-1	1.0E0
	Annotation Cluster 31	Enrichment Score: 0.2	G			Count	P_Value	Benjamini
☐	SMART	FU	RT			110	4.3E-1	1.0E0
☐	INTERPRO	Furin-like repeat	RT			110	4.4E-1	1.0E0
☐	INTERPRO	Giardia variant-specific surface protein	RT			135	7.3E-1	1.0E0
☐	INTERPRO	EGF-like	RT			105	8.7E-1	1.0E0
☐	SMART	EGF	RT			105	8.8E-1	1.0E0
	Annotation Cluster 32	Enrichment Score: 0.19	G			Count	P_Value	Benjamini
☐	GOTERM_BP_FAT	protein amino acid lipidation	RT			4	6.4E-1	1.0E0
☐	GOTERM_BP_FAT	lipoprotein metabolic process	RT			4	6.4E-1	1.0E0
☐	GOTERM_BP_FAT	lipoprotein biosynthetic process	RT			4	6.4E-1	1.0E0
	Annotation Cluster 33	Enrichment Score: 0.18	G			Count	P_Value	Benjamini
☐	GOTERM_MF_FAT	ATPase activity, coupled to transmembrane movement of ions, phosphorylative mechanism	RT			9	3.7E-1	1.0E0
☐	INTERPRO	ATPase, P-type, K/Mg/Cd/Cu/Zn/Na/Ca/Na/H-transporter	RT			9	3.9E-1	1.0E0
☐	INTERPRO	ATPase, P-type, ATPase-associated region	RT			8	4.9E-1	1.0E0
☐	INTERPRO	ATPase, P-type phosphorylation site	RT			6	5.6E-1	1.0E0
☐	SP_PIR_KEYWORDS	magnesium	RT			8	6.0E-1	9.9E-1
☐	GOTERM_BP_FAT	lipid transport	RT			4	6.4E-1	1.0E0
☐	GOTERM_BP_FAT	lipid localization	RT			4	6.4E-1	1.0E0
☐	GOTERM_MF_FAT	magnesium ion binding	RT			9	7.3E-1	1.0E0
☐	GOTERM_MF_FAT	lipid transporter activity	RT			4	7.9E-1	1.0E0
☐	GOTERM_BP_FAT	phospholipid transport	RT			3	7.9E-1	1.0E0
☐	INTERPRO	ATPase, P-type, phospholipid-translocating,	RT			3	8.0E-1	1.0E0

Category	Term	RT	Count	P_Value	Benjamini
	flippase				
GOTERM_MF_FAT	aminophospholipid transporter activity	RT	3	8.9E-1	1.0E0
GOTERM_MF_FAT	phospholipid-translocating ATPase activity	RT	3	8.9E-1	1.0E0
GOTERM_MF_FAT	phospholipid transporter activity	RT	3	8.9E-1	1.0E0
Annotation Cluster 34	Enrichment Score: 0.18		Count	P_Value	Benjamini
GOTERM_MF_FAT	GTPase regulator activity	RT	8	4.7E-1	1.0E0
GOTERM_MF_FAT	small GTPase regulator activity	RT	8	4.7E-1	1.0E0
GOTERM_MF_FAT	nucleoside-triphosphatase regulator activity	RT	9	5.1E-1	1.0E0
GOTERM_BP_FAT	regulation of ARF protein signal transduction	RT	4	6.4E-1	1.0E0
GOTERM_BP_FAT	regulation of Ras protein signal transduction	RT	6	6.8E-1	1.0E0
GOTERM_BP_FAT	regulation of small GTPase mediated signal transduction	RT	6	6.8E-1	1.0E0
GOTERM_MF_FAT	enzyme activator activity	RT	6	6.8E-1	1.0E0
GOTERM_BP_FAT	regulation of hydrolase activity	RT	5	7.9E-1	1.0E0
GOTERM_BP_FAT	regulation of GTPase activity	RT	5	7.9E-1	1.0E0
GOTERM_BP_FAT	regulation of Ras GTPase activity	RT	4	8.8E-1	1.0E0
GOTERM_MF_FAT	GTPase activator activity	RT	4	8.8E-1	1.0E0
Annotation Cluster 35	Enrichment Score: 0.17		Count	P_Value	Benjamini
GOTERM_BP_FAT	cyclic nucleotide metabolic process	RT	3	6.1E-1	1.0E0
GOTERM_BP_FAT	cyclic nucleotide biosynthetic process	RT	3	6.1E-1	1.0E0
GOTERM_MF_FAT	phosphorus-oxygen lyase activity	RT	3	6.1E-1	1.0E0
GOTERM_BP_FAT	nucleoside monophosphate biosynthetic process	RT	3	7.9E-1	1.0E0
GOTERM_BP_FAT	nucleoside monophosphate metabolic process	RT	3	7.9E-1	1.0E0
Annotation Cluster 36	Enrichment Score: 0.16		Count	P_Value	Benjamini
SMART	ZnF_C2H2	RT	11	6.9E-1	1.0E0
INTERPRO	Zinc finger, C2H2-type	RT	11	6.9E-1	1.0E0
INTERPRO	Zinc finger, C2H2-like	RT	11	6.9E-1	1.0E0
Annotation Cluster 37	Enrichment Score: 0.14		Count	P_Value	Benjamini
GOTERM_BP_FAT	hexose metabolic process	RT	13	5.1E-1	1.0E0
GOTERM_BP_FAT	glucose metabolic process	RT	13	5.1E-1	1.0E0
GOTERM_BP_FAT	generation of precursor metabolites and energy	RT	16	5.8E-1	1.0E0
GOTERM_BP_FAT	monosaccharide metabolic process	RT	14	6.3E-1	1.0E0
GOTERM_BP_FAT	hexose catabolic process	RT	8	8.0E-1	1.0E0
GOTERM_BP_FAT	alcohol catabolic process	RT	8	8.0E-1	1.0E0
GOTERM_BP_FAT	glucose catabolic process	RT	8	8.0E-1	1.0E0
GOTERM_BP_FAT	monosaccharide catabolic process	RT	8	8.0E-1	1.0E0
GOTERM_BP_FAT	cellular carbohydrate catabolic process	RT	8	8.0E-1	1.0E0
GOTERM_BP_FAT	carbohydrate catabolic process	RT	8	8.0E-1	1.0E0
GOTERM_BP_FAT	glycolysis	RT	6	8.7E-1	1.0E0
SP_PIR_KEYWORDS	glycolysis	RT	3	9.5E-1	1.0E0
Annotation Cluster 38	Enrichment Score: 0.14		Count	P_Value	Benjamini
GOTERM_BP_FAT	organophosphate metabolic process	RT	9	5.0E-1	1.0E0
GOTERM_BP_FAT	phospholipid metabolic process	RT	8	6.0E-1	1.0E0
GOTERM_BP_FAT	lipid biosynthetic process	RT	9	6.2E-1	1.0E0
GOTERM_BP_FAT	phospholipid biosynthetic process	RT	5	6.6E-1	1.0E0
GOTERM_BP_FAT	glycerolipid metabolic process	RT	4	8.8E-1	1.0E0
GOTERM_BP_FAT	phosphoinositide metabolic process	RT	4	8.8E-1	1.0E0
GOTERM_BP_FAT	glycerophospholipid metabolic process	RT	4	8.8E-1	1.0E0
GOTERM_MF_FAT	inositol or phosphatidylinositol kinase activity	RT	3	8.9E-1	1.0E0
Annotation Cluster 39	Enrichment Score: 0.14		Count	P_Value	Benjamini
GOTERM_BP_FAT	dephosphorylation	RT	10	6.4E-1	1.0E0
GOTERM_BP_FAT	protein amino acid dephosphorylation	RT	8	7.1E-1	1.0E0
GOTERM_MF_FAT	phosphatase activity	RT	25	7.2E-1	1.0E0
GOTERM_MF_FAT	phosphoprotein phosphatase activity	RT	15	8.5E-1	1.0E0
Annotation Cluster 40	Enrichment Score: 0.14		Count	P_Value	Benjamini
GOTERM_BP_FAT	cellular carbohydrate biosynthetic process	RT	5	6.6E-1	1.0E0
GOTERM_BP_FAT	carbohydrate biosynthetic process	RT	5	6.6E-1	1.0E0
GOTERM_BP_FAT	alcohol biosynthetic process	RT	3	8.9E-1	1.0E0
Annotation Cluster 41	Enrichment Score: 0.13		Count	P_Value	Benjamini

INTERPRO	ABC transporter-like	RT	14	6.6E-1	1.0E0
INTERPRO	ABC transporter, conserved site	RT	13	7.2E-1	1.0E0
COG_ONTOLOGY	Defense mechanisms	RT	12	8.5E-1	1.0E0
Annotation Cluster 42	Enrichment Score: 0.13		Count	P_Value	Benjamini
SP_PIR_KEYWORDS	viral nucleoprotein	RT	10	5.3E-1	1.0E0
INTERPRO	Like-Sm ribonucleoprotein, core	RT	7	7.2E-1	1.0E0
SMART	Sm	RT	4	8.9E-1	1.0E0
INTERPRO	Like-Sm ribonucleoprotein, eukaryotic and archaea-type, core	RT	4	8.9E-1	1.0E0
Annotation Cluster 43	Enrichment Score: 0.13		Count	P_Value	Benjamini
INTERPRO	Cyclin-related	RT	5	5.1E-1	1.0E0
SMART	CYCLIN	RT	3	9.0E-1	1.0E0
INTERPRO	Cyclin	RT	3	9.0E-1	1.0E0
Annotation Cluster 44	Enrichment Score: 0.13		Count	P_Value	Benjamini
INTERPRO	Pseudouridine synthase I, TruA	RT	3	6.3E-1	1.0E0
PIR_SUPERFAMILY	PIRSF001430:tRNA_psdUrid_synth	RT	3	6.3E-1	1.0E0
GOTERM_MF_FAT	intramolecular transferase activity	RT	7	7.0E-1	1.0E0
GOTERM_BP_FAT	RNA modification	RT	9	8.1E-1	1.0E0
GOTERM_BP_FAT	pseudouridine synthesis	RT	4	8.8E-1	1.0E0
GOTERM_MF_FAT	pseudouridine synthase activity	RT	4	8.8E-1	1.0E0
Annotation Cluster 45	Enrichment Score: 0.13		Count	P_Value	Benjamini
GOTERM_MF_FAT	thiolester hydrolase activity	RT	6	5.4E-1	1.0E0
GOTERM_MF_FAT	ubiquitin thiolesterase activity	RT	4	7.9E-1	1.0E0
INTERPRO	Peptidase C19, ubiquitin carboxyl-terminal hydrolase 2	RT	4	8.0E-1	1.0E0
GOTERM_BP_FAT	ubiquitin-dependent protein catabolic process	RT	7	9.2E-1	1.0E0
Annotation Cluster 46	Enrichment Score: 0.12		Count	P_Value	Benjamini
INTERPRO	Kelch-type beta propeller	RT	5	6.8E-1	1.0E0
SMART	Kelch	RT	4	8.0E-1	1.0E0
INTERPRO	Kelch repeat type 1	RT	4	8.0E-1	1.0E0
Annotation Cluster 47	Enrichment Score: 0.12		Count	P_Value	Benjamini
INTERPRO	Cyclin-related	RT	5	5.1E-1	1.0E0
SP_PIR_KEYWORDS	cyclin	RT	5	9.3E-1	1.0E0
INTERPRO	Cyclin, N-terminal	RT	3	9.5E-1	1.0E0
Annotation Cluster 48	Enrichment Score: 0.12		Count	P_Value	Benjamini
KEGG_PATHWAY	Valine, leucine and isoleucine biosynthesis	RT	5	3.1E-1	1.0E0
INTERPRO	Aminoacyl-tRNA synthetase, class I, conserved site	RT	7	4.4E-1	1.0E0
GOTERM_BP_FAT	ncRNA metabolic process	RT	30	6.6E-1	1.0E0
GOTERM_BP_FAT	tRNA aminoacylation for protein translation	RT	12	8.3E-1	1.0E0
GOTERM_MF_FAT	ligase activity, forming carbon-oxygen bonds	RT	13	8.4E-1	1.0E0
GOTERM_MF_FAT	aminoacyl-tRNA ligase activity	RT	13	8.4E-1	1.0E0
GOTERM_MF_FAT	ligase activity, forming aminoacyl-tRNA and related compounds	RT	13	8.4E-1	1.0E0
GOTERM_BP_FAT	tRNA metabolic process	RT	21	8.7E-1	1.0E0
SP_PIR_KEYWORDS	Aminoacyl-tRNA synthetase	RT	12	8.8E-1	9.9E-1
GOTERM_BP_FAT	tRNA aminoacylation	RT	12	8.8E-1	1.0E0
GOTERM_BP_FAT	amino acid activation	RT	12	8.8E-1	1.0E0
INTERPRO	Rossmann-like alpha/beta/alpha sandwich fold	RT	10	8.9E-1	1.0E0
KEGG_PATHWAY	Aminoacyl-tRNA biosynthesis	RT	11	9.0E-1	1.0E0
SP_PIR_KEYWORDS	protein biosynthesis	RT	5	9.3E-1	1.0E0
INTERPRO	Aminoacyl-tRNA synthetase, class II, conserved region	RT	4	9.7E-1	1.0E0
Annotation Cluster 49	Enrichment Score: 0.11		Count	P_Value	Benjamini
GOTERM_MF_FAT	endoribonuclease activity	RT	4	6.4E-1	1.0E0
GOTERM_MF_FAT	ribonuclease activity	RT	6	7.9E-1	1.0E0
GOTERM_MF_FAT	endoribonuclease activity, producing 5'-phosphomonoesters	RT	3	7.9E-1	1.0E0
GOTERM_MF_FAT	endonuclease activity, active with either ribo- or deoxyribonucleic acids and producing 5'-phosphomonoesters	RT	4	8.8E-1	1.0E0
Annotation Cluster 50	Enrichment Score: 0.11		Count	P_Value	Benjamini
GOTERM_MF_FAT	peptidase activity, acting on L-amino acid peptides	RT	40	5.8E-1	1.0E0
GOTERM_MF_FAT	peptidase activity	RT	46	6.0E-1	1.0E0

GOTERM_BP_FAT	proteolysis	RT	51	6.0E-1	1.0E0
GOTERM_MF_FAT	endopeptidase activity	RT	26	7.3E-1	1.0E0
GOTERM_MF_FAT	cysteine-type peptidase activity	RT	17	7.5E-1	1.0E0
SMART	Pept_C1	RT	14	9.0E-1	1.0E0
INTERPRO	Peptidase C1A, papain	RT	14	9.0E-1	1.0E0
INTERPRO	Peptidase C1A, papain C-terminal	RT	14	9.0E-1	1.0E0
GOTERM_MF_FAT	cysteine-type endopeptidase activity	RT	12	9.2E-1	1.0E0
SP_PIR_KEYWORDS	Protease	RT	18	9.2E-1	1.0E0
INTERPRO	Peptidase, cysteine peptidase active site	RT	14	9.7E-1	1.0E0
Annotation Cluster 51	Enrichment Score: 0.11		Count	P_Value	Benjamini
SP_PIR_KEYWORDS	nucleotide-binding	RT	158	4.1E-1	9.9E-1
SP_PIR_KEYWORDS	atp-binding	RT	148	5.5E-1	9.9E-1
GOTERM_BP_FAT	phosphorus metabolic process	RT	171	7.2E-1	1.0E0
GOTERM_BP_FAT	phosphate metabolic process	RT	171	7.2E-1	1.0E0
GOTERM_MF_FAT	nucleotide binding	RT	349	7.5E-1	1.0E0
GOTERM_BP_FAT	phosphorylation	RT	161	7.6E-1	1.0E0
SP_PIR_KEYWORDS	kinase	RT	184	7.8E-1	9.9E-1
GOTERM_BP_FAT	protein amino acid phosphorylation	RT	149	8.1E-1	1.0E0
GOTERM_MF_FAT	ribonucleotide binding	RT	322	8.2E-1	1.0E0
GOTERM_MF_FAT	purine ribonucleotide binding	RT	322	8.2E-1	1.0E0
GOTERM_MF_FAT	protein kinase activity	RT	150	8.2E-1	1.0E0
GOTERM_MF_FAT	purine nucleotide binding	RT	325	8.5E-1	1.0E0
INTERPRO	Protein kinase, core	RT	148	8.6E-1	1.0E0
GOTERM_MF_FAT	adenyl ribonucleotide binding	RT	291	9.0E-1	1.0E0
GOTERM_MF_FAT	ATP binding	RT	291	9.0E-1	1.0E0
GOTERM_MF_FAT	nucleoside binding	RT	297	9.1E-1	1.0E0
INTERPRO	Serine/threonine protein kinase-related	RT	127	9.2E-1	1.0E0
GOTERM_MF_FAT	purine nucleoside binding	RT	294	9.2E-1	1.0E0
GOTERM_MF_FAT	adenyl nucleotide binding	RT	294	9.2E-1	1.0E0
Annotation Cluster 52	Enrichment Score: 0.1		Count	P_Value	Benjamini
GOTERM_BP_FAT	regulation of transcription	RT	20	4.1E-1	1.0E0
GOTERM_MF_FAT	NAD or NADH binding	RT	6	5.4E-1	1.0E0
GOTERM_BP_FAT	regulation of RNA metabolic process	RT	8	6.0E-1	1.0E0
GOTERM_BP_FAT	regulation of transcription, DNA-dependent	RT	8	6.0E-1	1.0E0
GOTERM_BP_FAT	gene silencing	RT	5	6.6E-1	1.0E0
GOTERM_BP_FAT	negative regulation of gene expression	RT	5	6.6E-1	1.0E0
GOTERM_BP_FAT	negative regulation of macromolecule metabolic process	RT	5	6.6E-1	1.0E0
GOTERM_BP_FAT	chromatin silencing	RT	3	8.9E-1	1.0E0
GOTERM_BP_FAT	negative regulation of cellular biosynthetic process	RT	3	8.9E-1	1.0E0
GOTERM_BP_FAT	negative regulation of RNA metabolic process	RT	3	8.9E-1	1.0E0
GOTERM_BP_FAT	negative regulation of biosynthetic process	RT	3	8.9E-1	1.0E0
GOTERM_BP_FAT	negative regulation of macromolecule biosynthetic process	RT	3	8.9E-1	1.0E0
GOTERM_BP_FAT	regulation of gene expression, epigenetic	RT	3	8.9E-1	1.0E0
GOTERM_BP_FAT	negative regulation of transcription	RT	3	8.9E-1	1.0E0
GOTERM_BP_FAT	negative regulation of nitrogen compound metabolic process	RT	3	8.9E-1	1.0E0
GOTERM_BP_FAT	negative regulation of nucleobase, nucleoside, nucleotide and nucleic acid metabolic process	RT	3	8.9E-1	1.0E0
GOTERM_BP_FAT	negative regulation of gene expression, epigenetic	RT	3	8.9E-1	1.0E0
GOTERM_BP_FAT	negative regulation of transcription, DNA-dependent	RT	3	8.9E-1	1.0E0
GOTERM_MF_FAT	NAD binding	RT	3	8.9E-1	1.0E0
INTERPRO	NAD-dependent histone deacetylase, silent information regulator Sir2	RT	3	9.0E-1	1.0E0
GOTERM_BP_FAT	protein amino acid deacetylation	RT	3	9.5E-1	1.0E0
GOTERM_MF_FAT	hydrolase activity, acting on carbon-nitrogen (but not peptide) bonds, in linear amides	RT	3	9.9E-1	1.0E0
Annotation Cluster 53	Enrichment Score: 0.1		Count	P_Value	Benjamini
GOTERM_BP_FAT	lipid biosynthetic process	RT	9	6.2E-1	1.0E0

GOTERM_BP_FAT	isoprenoid biosynthetic process	RT	3	8.9E-1	1.0E0
GOTERM_BP_FAT	isoprenoid metabolic process	RT	3	8.9E-1	1.0E0
Annotation Cluster 54	Enrichment Score: 0.1		Count	P_Value	Benjamini
GOTERM_BP_FAT	membrane docking	RT	3	7.9E-1	1.0E0
GOTERM_BP_FAT	exocytosis	RT	3	7.9E-1	1.0E0
GOTERM_BP_FAT	secretion by cell	RT	3	7.9E-1	1.0E0
GOTERM_BP_FAT	secretion	RT	3	7.9E-1	1.0E0
GOTERM_BP_FAT	vesicle docking during exocytosis	RT	3	7.9E-1	1.0E0
GOTERM_BP_FAT	vesicle docking	RT	3	7.9E-1	1.0E0
INTERPRO	Sec1-like protein	RT	3	8.0E-1	1.0E0
Annotation Cluster 55	Enrichment Score: 0.1		Count	P_Value	Benjamini
SP_PIR_KEYWORDS	motor protein	RT	15	5.6E-1	9.9E-1
INTERPRO	Kinesin, motor region, conserved site	RT	11	6.9E-1	1.0E0
SP_PIR_KEYWORDS	microtubule	RT	18	7.6E-1	1.0E0
SMART	KISc	RT	16	7.6E-1	1.0E0
INTERPRO	Kinesin, motor region	RT	16	7.7E-1	1.0E0
GOTERM_MF_FAT	motor activity	RT	23	8.1E-1	1.0E0
GOTERM_MF_FAT	microtubule motor activity	RT	23	8.1E-1	1.0E0
GOTERM_BP_FAT	microtubule-based process	RT	29	8.4E-1	1.0E0
GOTERM_BP_FAT	microtubule-based movement	RT	25	8.6E-1	1.0E0
GOTERM_CC_FAT	microtubule	RT	18	8.7E-1	1.0E0
GOTERM_CC_FAT	microtubule cytoskeleton	RT	28	9.0E-1	1.0E0
GOTERM_CC_FAT	cytoskeletal part	RT	28	9.0E-1	1.0E0
GOTERM_CC_FAT	cytoskeleton	RT	29	9.1E-1	1.0E0
Annotation Cluster 56	Enrichment Score: 0.09		Count	P_Value	Benjamini
GOTERM_BP_FAT	microtubule cytoskeleton organization	RT	3	7.9E-1	1.0E0
INTERPRO	Spc97/Spc98	RT	3	8.0E-1	1.0E0
GOTERM_CC_FAT	spindle pole	RT	3	8.1E-1	1.0E0
GOTERM_CC_FAT	spindle	RT	3	8.1E-1	1.0E0
GOTERM_CC_FAT	microtubule organizing center	RT	3	8.1E-1	1.0E0
Annotation Cluster 57	Enrichment Score: 0.09		Count	P_Value	Benjamini
INTERPRO	RNA helicase, DEAD-box type, Q motif	RT	12	4.0E-1	1.0E0
GOTERM_MF_FAT	ATPase activity	RT	54	6.5E-1	1.0E0
GOTERM_MF_FAT	ATPase activity, coupled	RT	40	6.8E-1	1.0E0
GOTERM_MF_FAT	helicase activity	RT	29	8.5E-1	1.0E0
INTERPRO	DNA/RNA helicase, DEAD/DEAH box type, N-terminal	RT	16	8.7E-1	1.0E0
GOTERM_MF_FAT	purine NTP-dependent helicase activity	RT	20	8.7E-1	1.0E0
GOTERM_MF_FAT	ATP-dependent helicase activity	RT	20	8.7E-1	1.0E0
SP_PIR_KEYWORDS	helicase	RT	23	8.8E-1	9.9E-1
SMART	HELICc	RT	22	9.4E-1	1.0E0
INTERPRO	DNA/RNA helicase, C-terminal	RT	22	9.5E-1	1.0E0
SMART	DEXDc	RT	21	9.6E-1	1.0E0
INTERPRO	Helicase, superfamily 1 and 2, ATP-binding	RT	21	9.6E-1	1.0E0
INTERPRO	DEAD-like helicase, N-terminal	RT	21	9.6E-1	1.0E0
Annotation Cluster 58	Enrichment Score: 0.09		Count	P_Value	Benjamini
SP_PIR_KEYWORDS	Rotamase	RT	5	7.9E-1	9.9E-1
GOTERM_MF_FAT	cis-trans isomerase activity	RT	5	7.9E-1	1.0E0
GOTERM_MF_FAT	peptidyl-prolyl cis-trans isomerase activity	RT	5	7.9E-1	1.0E0
INTERPRO	Peptidyl-prolyl cis-trans isomerase, FKBP-type	RT	4	8.0E-1	1.0E0
GOTERM_BP_FAT	protein folding	RT	15	9.2E-1	1.0E0
Annotation Cluster 59	Enrichment Score: 0.09		Count	P_Value	Benjamini
SP_PIR_KEYWORDS	Endonuclease	RT	17	7.5E-1	1.0E0
SP_PIR_KEYWORDS	rna-directed dna polymerase	RT	13	7.7E-1	9.9E-1
GOTERM_MF_FAT	endonuclease activity	RT	19	7.7E-1	1.0E0
GOTERM_MF_FAT	nuclease activity	RT	27	8.4E-1	1.0E0
GOTERM_MF_FAT	RNA-directed DNA polymerase activity	RT	13	8.4E-1	1.0E0
GOTERM_MF_FAT	nucleotidyltransferase activity	RT	39	8.4E-1	1.0E0
GOTERM_MF_FAT	DNA polymerase activity	RT	16	9.6E-1	1.0E0

Annotation Cluster 60	Enrichment Score: 0.08		Count	P_Value	Benjamini
GOTERM_BP_FAT	proteolysis	RT	51	6.0E-1	1.0E0
GOTERM_BP_FAT	proteolysis involved in cellular protein catabolic process	RT	19	7.0E-1	1.0E0
GOTERM_BP_FAT	cellular protein catabolic process	RT	19	7.0E-1	1.0E0
SP_PIR_KEYWORDS	ubl conjugation pathway	RT	8	7.1E-1	9.9E-1
INTERPRO	Proteasome, alpha and beta subunits	RT	8	7.3E-1	1.0E0
GOTERM_BP_FAT	cellular macromolecule catabolic process	RT	19	7.7E-1	1.0E0
SP_PIR_KEYWORDS	threonine protease	RT	4	7.9E-1	9.9E-1
GOTERM_BP_FAT	protein catabolic process	RT	21	7.9E-1	1.0E0
GOTERM_MF_FAT	threonine-type peptidase activity	RT	8	8.1E-1	1.0E0
GOTERM_MF_FAT	threonine-type endopeptidase activity	RT	8	8.1E-1	1.0E0
INTERPRO	Proteasome, subunit alpha/beta	RT	8	8.2E-1	1.0E0
GOTERM_BP_FAT	macromolecule catabolic process	RT	21	8.3E-1	1.0E0
GOTERM_CC_FAT	proteasome core complex	RT	8	8.4E-1	1.0E0
GOTERM_BP_FAT	modification-dependent protein catabolic process	RT	14	8.4E-1	1.0E0
GOTERM_BP_FAT	modification-dependent macromolecule catabolic process	RT	14	8.4E-1	1.0E0
SP_PIR_KEYWORDS	proteasome	RT	10	9.1E-1	1.0E0
GOTERM_BP_FAT	ubiquitin-dependent protein catabolic process	RT	7	9.2E-1	1.0E0
PIR_SUPERFAMILY	PIRSF001212:multicatalytic endopeptidase complex chain C9	RT	3	9.6E-1	1.0E0
GOTERM_CC_FAT	proteasome complex	RT	10	9.6E-1	1.0E0
INTERPRO	Proteasome, alpha-subunit, conserved site	RT	3	9.8E-1	1.0E0
GOTERM_CC_FAT	cytosol	RT	13	9.9E-1	1.0E0
KEGG_PATHWAY	Proteasome	RT	12	9.9E-1	1.0E0
Annotation Cluster 61	Enrichment Score: 0.08		Count	P_Value	Benjamini
SP_PIR_KEYWORDS	tpr repeat	RT	6	6.8E-1	9.9E-1
INTERPRO	Tetratricopeptide region	RT	14	7.4E-1	1.0E0
INTERPRO	Tetratricopeptide-like helical	RT	17	8.3E-1	1.0E0
INTERPRO	Tetratricopeptide TPR-1	RT	10	9.3E-1	1.0E0
SMART	TPR	RT	12	9.3E-1	1.0E0
INTERPRO	Tetratricopeptide repeat	RT	12	9.3E-1	1.0E0
Annotation Cluster 62	Enrichment Score: 0.08		Count	P_Value	Benjamini
GOTERM_BP_FAT	translational initiation	RT	7	7.0E-1	1.0E0
GOTERM_MF_FAT	translation factor activity, nucleic acid binding	RT	17	8.6E-1	1.0E0
GOTERM_MF_FAT	translation initiation factor activity	RT	13	8.8E-1	1.0E0
SP_PIR_KEYWORDS	Initiation factor	RT	9	9.4E-1	1.0E0
Annotation Cluster 63	Enrichment Score: 0.07		Count	P_Value	Benjamini
GOTERM_BP_FAT	protein amino acid dephosphorylation	RT	8	7.1E-1	1.0E0
GOTERM_MF_FAT	protein tyrosine/serine/threonine phosphatase activity	RT	3	8.9E-1	1.0E0
INTERPRO	Protein-tyrosine phosphatase, dual specificity	RT	3	9.0E-1	1.0E0
INTERPRO	Dual specificity phosphatase, catalytic domain	RT	3	9.0E-1	1.0E0
Annotation Cluster 64	Enrichment Score: 0.07		Count	P_Value	Benjamini
INTERPRO	Metallophosphoesterase	RT	13	6.4E-1	1.0E0
SP_PIR_KEYWORDS	iron	RT	11	8.2E-1	9.9E-1
GOTERM_MF_FAT	phosphoprotein phosphatase activity	RT	15	8.5E-1	1.0E0
SMART	PP2Ac	RT	8	8.8E-1	1.0E0
INTERPRO	Serine/threonine-specific protein phosphatase and bis(5-nucleosyl)-tetraphosphatase	RT	8	8.8E-1	1.0E0
PIR_SUPERFAMILY	PIRSF000908:serine/threonine-protein phosphatase, conventional type	RT	4	9.5E-1	1.0E0
GOTERM_MF_FAT	iron ion binding	RT	14	9.8E-1	1.0E0
Annotation Cluster 65	Enrichment Score: 0.07		Count	P_Value	Benjamini
GOTERM_MF_FAT	acetyltransferase activity	RT	6	7.9E-1	1.0E0
GOTERM_MF_FAT	N-acyltransferase activity	RT	6	7.9E-1	1.0E0
GOTERM_MF_FAT	N-acetyltransferase activity	RT	5	8.7E-1	1.0E0
INTERPRO	GCN5-related N-acetyltransferase	RT	5	8.8E-1	1.0E0
INTERPRO	Acyl-CoA N-acyltransferase	RT	5	9.3E-1	1.0E0
Annotation Cluster 66	Enrichment Score: 0.07		Count	P_Value	Benjamini

SP_PIR_KEYWORDS	dna replication	RT	6	7.9E-1	9.9E-1
GOTERM_BP_FAT	DNA replication	RT	17	8.9E-1	1.0E0
KEGG_PATHWAY	DNA replication	RT	11	9.0E-1	1.0E0
Annotation Cluster 67	Enrichment Score: 0.06		Count	P_Value	Benjamini
SMART	MCM	RT	4	8.0E-1	1.0E0
INTERPRO	DNA-dependent ATPase MCM	RT	4	8.0E-1	1.0E0
KEGG_PATHWAY	DNA replication	RT	11	9.0E-1	1.0E0
INTERPRO	Nucleic acid-binding, OB-fold	RT	8	9.9E-1	1.0E0
Annotation Cluster 68	Enrichment Score: 0.06		Count	P_Value	Benjamini
KEGG_PATHWAY	Pyrimidine metabolism	RT	19	7.6E-1	1.0E0
SP_PIR_KEYWORDS	Transcription	RT	5	7.9E-1	9.9E-1
GOTERM_MF_FAT	ribonucleoside binding	RT	3	7.9E-1	1.0E0
GOTERM_BP_FAT	transcription	RT	14	8.4E-1	1.0E0
GOTERM_MF_FAT	DNA-directed RNA polymerase activity	RT	12	8.8E-1	1.0E0
GOTERM_MF_FAT	RNA polymerase activity	RT	12	9.2E-1	1.0E0
KEGG_PATHWAY	RNA polymerase	RT	9	9.3E-1	1.0E0
SP_PIR_KEYWORDS	nucleotidyltransferase	RT	12	9.4E-1	1.0E0
SP_PIR_KEYWORDS	dna-directed rna polymerase	RT	6	9.7E-1	1.0E0
KEGG_PATHWAY	Purine metabolism	RT	15	9.8E-1	1.0E0
Annotation Cluster 69	Enrichment Score: 0.05		Count	P_Value	Benjamini
GOTERM_BP_FAT	carboxylic acid biosynthetic process	RT	4	8.8E-1	1.0E0
GOTERM_BP_FAT	organic acid biosynthetic process	RT	4	8.8E-1	1.0E0
GOTERM_BP_FAT	cellular amino acid biosynthetic process	RT	3	8.9E-1	1.0E0
GOTERM_BP_FAT	amine biosynthetic process	RT	3	9.5E-1	1.0E0
Annotation Cluster 70	Enrichment Score: 0.05		Count	P_Value	Benjamini
KEGG_PATHWAY	Mismatch repair	RT	8	8.3E-1	1.0E0
KEGG_PATHWAY	DNA replication	RT	11	9.0E-1	1.0E0
KEGG_PATHWAY	Nucleotide excision repair	RT	7	9.8E-1	1.0E0
Annotation Cluster 71	Enrichment Score: 0.05		Count	P_Value	Benjamini
INTERPRO	EF-Hand type	RT	10	8.3E-1	1.0E0
INTERPRO	EF-HAND 1	RT	11	8.4E-1	1.0E0
SP_PIR_KEYWORDS	calcium	RT	8	8.7E-1	9.9E-1
INTERPRO	EF-HAND 2	RT	9	8.8E-1	1.0E0
SMART	EFh	RT	5	9.6E-1	1.0E0
INTERPRO	Calcium-binding EF-hand	RT	5	9.6E-1	1.0E0
INTERPRO	EF hand	RT	5	9.6E-1	1.0E0
Annotation Cluster 72	Enrichment Score: 0.05		Count	P_Value	Benjamini
SP_PIR_KEYWORDS	Chaperone	RT	7	8.0E-1	9.9E-1
SP_PIR_KEYWORDS	cytoplasm	RT	10	8.1E-1	9.9E-1
INTERPRO	Chaperonin Cpn60/TCP-1	RT	5	8.8E-1	1.0E0
INTERPRO	Chaperone, tailless complex polypeptide 1	RT	4	9.4E-1	1.0E0
PIR_SUPERFAMILY	PIRSF002584:molecular chaperone t-complex-type	RT	4	9.5E-1	1.0E0
INTERPRO	Chaperonin TCP-1, conserved site	RT	4	9.7E-1	1.0E0
GOTERM_MF_FAT	unfolded protein binding	RT	9	9.8E-1	1.0E0
Annotation Cluster 73	Enrichment Score: 0.04		Count	P_Value	Benjamini
INTERPRO	Ribosomal RNA methyltransferase RrmJ/FtsJ	RT	3	8.0E-1	1.0E0
GOTERM_BP_FAT	RNA methylation	RT	3	8.9E-1	1.0E0
GOTERM_BP_FAT	methylation	RT	5	9.3E-1	1.0E0
GOTERM_BP_FAT	biopolymer methylation	RT	3	9.5E-1	1.0E0
GOTERM_BP_FAT	one-carbon metabolic process	RT	5	9.6E-1	1.0E0
Annotation Cluster 74	Enrichment Score: 0.04		Count	P_Value	Benjamini
SMART	EGF_Lam	RT	14	8.6E-1	1.0E0
INTERPRO	EGF-like, laminin	RT	14	8.6E-1	1.0E0
INTERPRO	EGF	RT	4	8.9E-1	1.0E0
INTERPRO	EGF, extracellular	RT	12	8.9E-1	1.0E0
INTERPRO	EGF-like, type 3	RT	14	9.9E-1	1.0E0
SP_PIR_KEYWORDS	egf-like domain	RT	6	1.0E0	1.0E0
Annotation Cluster 75	Enrichment Score: 0.04		Count	P_Value	Benjamini
SP_PIR_KEYWORDS	Aminotransferase	RT	3	8.9E-1	9.9E-1

GOTERM_MF_FAT	transaminase activity	RT		3	8.9E-1	1.0E0
GOTERM_MF_FAT	transferase activity, transferring nitrogenous groups	RT		3	9.7E-1	1.0E0
Annotation Cluster 76	Enrichment Score: 0.04	G		Count	P_Value	Benjamini
INTERPRO	Myb, DNA-binding	RT		3	9.0E-1	1.0E0
SMART	SANT	RT		5	9.3E-1	1.0E0
INTERPRO	SANT, DNA-binding	RT		5	9.3E-1	1.0E0
Annotation Cluster 77	Enrichment Score: 0.03	G		Count	P_Value	Benjamini
INTERPRO	Serine/threonine protein kinase, active site	RT		48	8.9E-1	1.0E0
GOTERM_MF_FAT	protein serine/threonine kinase activity	RT		55	9.1E-1	1.0E0
INTERPRO	Serine/threonine protein kinase-related	RT		127	9.2E-1	1.0E0
INTERPRO	Protein kinase, ATP binding site	RT		42	9.5E-1	1.0E0
SMART	S_TKc	RT		29	9.6E-1	1.0E0
INTERPRO	Serine/threonine protein kinase	RT		29	9.6E-1	1.0E0
SP_PIR_KEYWORDS	serine/threonine-protein kinase	RT		29	9.8E-1	1.0E0
Annotation Cluster 78	Enrichment Score: 0.03	G		Count	P_Value	Benjamini
GOTERM_MF_FAT	pyridoxal phosphate binding	RT		5	9.3E-1	1.0E0
GOTERM_MF_FAT	vitamin B6 binding	RT		5	9.3E-1	1.0E0
INTERPRO	Pyridoxal phosphate-dependent transferase, major region, subdomain 1	RT		4	9.4E-1	1.0E0
GOTERM_MF_FAT	vitamin binding	RT		5	9.6E-1	1.0E0
Annotation Cluster 79	Enrichment Score: 0.03	G		Count	P_Value	Benjamini
INTERPRO	Dynein heavy chain	RT		6	9.3E-1	1.0E0
GOTERM_CC_FAT	dynein complex	RT		6	9.4E-1	1.0E0
GOTERM_CC_FAT	microtubule associated complex	RT		7	9.6E-1	1.0E0
Annotation Cluster 80	Enrichment Score: 0.02	G		Count	P_Value	Benjamini
INTERPRO	RNA-directed DNA polymerase (reverse transcriptase), related	RT		3	9.0E-1	1.0E0
GOTERM_BP_FAT	RNA-dependent DNA replication	RT		3	9.7E-1	1.0E0
INTERPRO	RNA-directed DNA polymerase (reverse transcriptase)	RT		3	9.8E-1	1.0E0
Annotation Cluster 81	Enrichment Score: 0.02	G		Count	P_Value	Benjamini
INTERPRO	Thioredoxin, conserved site	RT		4	8.0E-1	1.0E0
INTERPRO	Thioredoxin-like subdomain	RT		3	9.0E-1	1.0E0
INTERPRO	Thioredoxin domain	RT		3	9.5E-1	1.0E0
INTERPRO	Thioredoxin, core	RT		3	9.8E-1	1.0E0
INTERPRO	Thioredoxin-like	RT		5	9.9E-1	1.0E0
INTERPRO	Thioredoxin fold	RT		9	9.9E-1	1.0E0
GOTERM_BP_FAT	cell redox homeostasis	RT		7	9.9E-1	1.0E0
GOTERM_BP_FAT	cellular homeostasis	RT		7	9.9E-1	1.0E0
GOTERM_BP_FAT	homeostatic process	RT		7	1.0E0	1.0E0
Annotation Cluster 82	Enrichment Score: 0.01	G		Count	P_Value	Benjamini
GOTERM_BP_FAT	protein complex assembly	RT		6	9.5E-1	1.0E0
GOTERM_BP_FAT	protein complex biogenesis	RT		6	9.5E-1	1.0E0
GOTERM_BP_FAT	cellular protein complex assembly	RT		5	9.6E-1	1.0E0
GOTERM_BP_FAT	protein polymerization	RT		3	9.9E-1	1.0E0
INTERPRO	Tubulin	RT		3	9.9E-1	1.0E0
INTERPRO	Tubulin/FtsZ, GTPase domain	RT		3	9.9E-1	1.0E0
INTERPRO	Tubulin, conserved site	RT		3	9.9E-1	1.0E0
Annotation Cluster 83	Enrichment Score: 0.01	G		Count	P_Value	Benjamini
GOTERM_MF_FAT	4 iron, 4 sulfur cluster binding	RT		4	9.3E-1	1.0E0
INTERPRO	4Fe-4S ferredoxin, iron-sulpur binding domain	RT		4	9.7E-1	1.0E0
INTERPRO	4Fe-4S ferredoxin, iron-sulphur binding, subgroup	RT		4	9.7E-1	1.0E0
INTERPRO	4Fe-4S ferredoxin, iron-sulphur binding, conserved site	RT		3	9.8E-1	1.0E0
GOTERM_MF_FAT	iron-sulfur cluster binding	RT		8	9.9E-1	1.0E0
GOTERM_MF_FAT	metal cluster binding	RT		8	9.9E-1	1.0E0
GOTERM_MF_FAT	electron carrier activity	RT		9	9.9E-1	1.0E0

201 terms were not clustered.

Please cite *Nature Protocols 2009; 4(1):44 & Genome Biology 2003; 4(5):P3* within any publication that makes use of any methods inspired by **DAVID**.

Term of Service | Contact Us | Site Map
